# A manual collection of *Syt*, *Esyt*, *Rph3a*, *Rph3al*, *Doc2*, and *Dblc2 *genes from 46 metazoan genomes - an open access resource for neuroscience and evolutionary biology

**DOI:** 10.1186/1471-2164-11-37

**Published:** 2010-01-15

**Authors:** Molly Craxton

**Affiliations:** 1 Medical Research Council Laboratory of Molecular Biology, Hills Road, Cambridge, CB2 0QH, UK

## Abstract

**Background:**

Synaptotagmin proteins were first identified in nervous tissue, residing in synaptic vesicles. Synaptotagmins were subsequently found to form a large family, some members of which play important roles in calcium triggered exocytic events. These members have been investigated intensively, but other family members are not well understood, making it difficult to grasp the meaning of family membership in functional terms. Further difficulty arises as families are defined quite legitimately in different ways: by common descent or by common possession of distinguishing features. One definition does not necessarily imply the other. The evolutionary range of genome sequences now available, can shed more light on synaptotagmin gene phylogeny and clarify family relationships. The aim of compiling this open access collection of synaptotagmin and synaptotagmin-like sequences, is that its use may lead to greater understanding of the biological function of these proteins in an evolutionary context.

**Results:**

46 metazoan genomes were examined and their complement of *Syt*, *Esyt*, *Rph3a*, *Rph3al*, *Doc2 *and *Dblc2 *genes identified. All of the sequences were compared, named, then examined in detail. *Esyt *genes were formerly named *Fam62*. The species in this collection are *Trichoplax*, *Nematostella*, *Capitella*, *Helobdella*, *Lottia*, *Ciona*, *Strongylocentrotus*, *Branchiostoma*, *Ixodes*, *Daphnia*, *Acyrthosiphon*, *Tribolium*, *Nasonia*, *Apis*, *Anopheles*, *Drosophila*, *Caenorhabditis*, *Takifugu*, *Tetraodon*, *Gasterosteus*, *Oryzias*, *Danio*, *Xenopus*, *Anolis*, *Gallus*, *Taeniopygia*,*Ornithorhynchus*, *Monodelphis*, *Mus *and *Homo*. All of the data described in this paper is available as additional files.

**Conclusions:**

Only a subset of synaptotagmin proteins appear able to function as calcium triggers. Syt1, Syt7 and Syt9 are ancient conserved synaptotagmins of this type. Some animals carry extensive repertoires of synaptotagmin genes. Other animals of no less complexity, carry only a small repertoire. Current understanding does not explain why this is so. The biological roles of many synaptotagmins remain to be understood. This collection of genes offers prospects for fruitful speculation about the functional roles of the synaptotagmin repertoires of different animals and includes a great range of biological complexity. With reference to this gene collection, functional relationships among *Syt*, *Esyt*, *Rph3a*, *Rph3al*, *Doc2 *and *Dblc2 *genes, which encode similar proteins, can better be assessed in future.

## Background

Synaptotagmin (Syt) proteins participate in regulated membrane fusion events in multicellular organisms. Syt research dates back to 1981, when the first Syt was identified as an integral protein of synaptic vesicles [1]. Since then, much effort has gone into discovering the function and the detailed mechanism of action of this protein, Syt1. Numerous experimental approaches have shown that Syt1 is crucially involved in fast neurotransmitter release at synapses [reviewed in [2-6]]. Syt1 binds calcium, serving as the calcium sensor which triggers synaptic vesicle exocytosis. Syt1 also binds to the neuronal SNARE proteins which are required for membrane fusion, as well as to membranes directly. The primary structure of Syt1 [7] revealed three important features: an N-terminal transmembrane (TM) domain, which serves to anchor the protein in the synaptic vesicle, plus two, tandem, C-terminal, cytoplasmic C2 domains (C2A and C2B) which specify the calcium, SNARE and membrane binding properties. Crystallographic studies of some Syt C2 domains [8,9] have revealed the exact nature of their calcium binding abilities. Structural bioinformatics [10-12] has shown that C2 domains are very common in the eukaryotic protein repertoire, but not all act to bind calcium. C2 domains can occur singly or as multiple copies in a given protein [13]. The particular domain organisation of Syt1 is important for its role in synaptic vesicle exocytosis. The TM domain tethers the protein to the membrane. The C2A and C2B domains, endowed with individual capacities to bind calcium, SNARE proteins, other accessory proteins and phospholipids, act both independently and synergistically, at different stages during the life cycle of the synaptic vesicle, to promote or inhibit fusion [14-16].

After the genetic code for Syt1 was discovered [7] genes similar to *Syt1 *were sought and found. Biochemical methods which were employed initially, led to an increase in the size of the rodent *Syt *family, from one member to thirteen members and beyond. *Syt *relatives were also identified and studied in *D. melanogaster *and *C. elegans*. All of these homologous *Syt *genes were found to encode proteins with a common domain architecture: an N-terminal TM domain connected by a variable length, poorly conserved linker sequence, to well conserved, tandem, C-terminal C2A and C2B domains and this domain architecture was taken to define the family. Functional studies with the proteins *in vivo *and *in vitro*, indicate that some respond to calcium but others do not. Whether or not they respond to calcium, Syt proteins are able to regulate membrane fusion due to common properties of their C2 domains [17-21]. Studies of the anatomical expression patterns of *Syt *genes show that *Syt1 *is abundantly expressed in nervous tissue [1,22-26]. In *D. melanogaster, Syt1 *expression is neuron specific but other *Syt *genes are expressed elsewhere [27]. In rodents, most *Syt *genes are expressed in the brain [28].

With the publication of whole genome sequences from multicellular organisms, it became possible to identify and compare complete genome complements of *Syt *genes using computational methods [29,30]. The phylogeny of these genes could be examined by comparing sequences from different organisms. Seventeen *Syt *homologues were found in each of the complete *H. sapiens *and *M. musculus *genome sequences. These were named *SYT1 *(*Syt1*) to *SYT17 *(*Syt17*) according to the nomenclature conventions of the HUGO Gene Nomenclature Committee [31] (and the Mouse Genome Informatics Database [32]). Some of these genes however, encode Syt proteins which lack the requisite domain architecture for inclusion in a Syt family defined by domain structure. The complete genome sequences of *C. elegans*, *D. melanogaster *and *A. thaliana *were analysed, together with draft genome sequences from other eukaryotes, to assess their Syt coding potential [30]. Proteins which share the stereotypical Syt domain architecture, are encoded by distinct (not phylogenetically homologous) gene families in plants and in animals [33] further confounding the notion of a homologous Syt family defined in terms of domain architecture. Functional similarity between plant and animal Syt proteins, at the level of calcium and membrane binding, has been demonstrated [34,35] in keeping with the notion that protein form specifies function. Proteins have been classified as Syt family members by the presence or absence of suitable characteristics in terms of protein form and function [eg. [6,36,37]] without studious regard to gene ancestry. Legitimately but confusingly, family membership can be defined in different ways: in terms of descent from a common ancestor (homology) or in terms of the common possession of distinguishing features. Since it is not straightforward to demonstrate that homology is the cause of the similarity between biological entities such as proteins, it is often just assumed, leading to the confusion of two different notions of a protein family. Please see [38,39] for explanations of the terminology of homology. Examination of the genomic specification of eukaryotic proteins can help to reveal their phylogenetic relationships because the evolutionary conservation of gene structure provides additional information on which to base an inference of homology. Families may thereby be resolved as those related by homology and those otherwise related. Ambiguity still remains however, because depending on the exact biological context, members of a family, however defined, can sometimes be considered as functionally interchangeable and thus worthy of a common functional identity. Thus, genes and proteins can acquire multiple identities depending on the perspective from which they are considered. Universal agreement about nomenclature for genes and proteins which is suitably clear and meaningful is a challenge for the future, but norms for gene nomenclature according to phylogenetic relationships are established [31,32].

It may be the case (as is often assumed for an orthologous family group) that members of a gene family related by heredity, share a common functional role [38-41]. However, due to the dynamics of eukaryotic genome evolution [41-45] the functional attributes of gene relatives can diverge. It is also possible, that separate start points, through advantageous gain of function, can lead through selection, to convergence upon a common form and function. While it is practicable to assign family membership to genes or proteins based on their apparent hereditary relationships, the functional implications of family membership cannot simply be deduced. More than a decade of research effort has been aimed at understanding how Syt1 works. Considerable effort has also been directed at understanding the functions of other Syt proteins. Current understanding however, is insufficient to predict the functions of a whole genome complement of Syt proteins, or indeed to predict what a genome complement of Syt proteins might consist of.

To help improve this lack of understanding, I have put together an open access resource intended to serve future research aimed at understanding the biological meaning of the hereditary and functional relationships among some of the metazoan Syt-like proteins. I have collected and compiled manually, information about homologous *Syt*, *Esyt*, *Rph3a, Rph3al*, *Doc2 *and *Dblc2 *genes from 46 metazoan genome sequences spanning a wide evolutionary range. I have not attempted to collect all genes capable of encoding proteins with similarity on any level to Syt proteins. Such a collection would ultimately include perhaps, all eukaryotic C2 domain proteins. My collection is restricted to homologous *Syt *genes plus a small group of genes already identified as similar but not homologous to *Syt *genes [33]. The collection does include a new group of homologous genes (*Dblc2*) which are present in the genomes of marine invertebrates and encode proteins similar to Syt proteins. The information in this collection may interest investigators in the field of evolutionary biology, with examples of dynamic genome evolution including whole gene duplication, partial duplication, gene fission, acquisition of novel coding exons, gene inactivation, extreme sequence divergence, intron mobilization and a variety of routes to the expression of altered gene products. For experimentalists who want to understand how Syt proteins act to effect the biology of animal nervous systems, the collection provides a utility with great evolutionary depth. In order to make progress in neuroscience, appropriate model organisms must be chosen. The choice of model organism is fundamental to the type of science which can be pursued. The range of organisms and genes in this collection, offer novel possibilities for future descriptive, comparative and hypothesis driven research.

In order to make the large amount of information described by this paper available for public scrutiny, it is available as 50 additional files which can be accessed online.

## Results and Discussion

### Origin of this gene collection

The origin of this manual gene curation project lay in an attempt to annotate some of the plant *NTMC2 *genes I had identified previously [33]. I chose the unique *NTMC2 *nomenclature to emphasise the phylogenetic relationships among these plant genes and to distinguish them from animal *Syt *genes. Plant *NTMC2T1*, *NTMC2T2 *and *NTMC2T3 *genes resemble animal *Syt *genes in having the same domain architecture. That this shared domain architecture implies functions in common, has now been demonstrated experimentally [34,35]. The *A. thaliana *NTMC2T1.1 protein (also known as SYT1 [34,35,37]) possesses calcium and membrane binding activities which allow it to function in a plasma membrane repair pathway induced by stress. While using the annotation facilities at DOE-JGI [46] I realised that the evolutionary range of genome sequences available for inspection at DOE-JGI, could be used to search for earlier origins of the embryophyte *NTMC2 *genes and metazoan *Syt *genes. The genome sequence of *M. brevicollis *[47] has demonstrated that, as theorised [48,49] the evolutionary transition from unicellularity to multicellularity involved abundant domain shuffling in proteins. *M. brevicollis*, a unicellular protist, closely related but basal to metazoans, does not possess *Syt *genes. It does have a gene with a degree of gene structure similarity, domain architecture similarity and amino acid sequence similarity to plant NTMC2 and animal Esyt proteins XM_001748216. Proteins with similarity to NTMC2 and Esyt proteins (but without similarity at the gene structure level) are present in other unicellular eukaryotes [33]. It seems reasonable therefore, to propose that while *NTMC2 *and *Esyt *genes could ultimately be homologous, having attained their current forms through descent from a unicellular ancestor, *Syt *genes, distinguished by their conserved gene structure, more likely represent a metazoan novelty.

**Figure 1:**
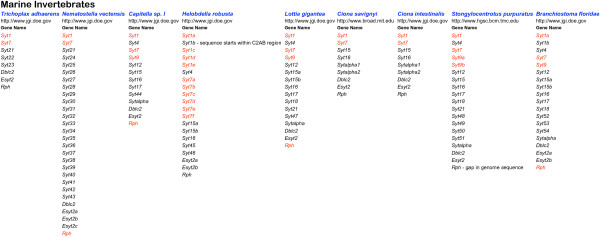
**Summary of the genes collected from marine invertebrate genomes**. The website of the organisation which sequenced the genome is listed below the organism name. Underneath the Gene Name heading, gene symbols are listed. Red symbols indicate sequences containing all ten acidic amino acid positions required for function as a calcium trigger for exocytosis.

**Figure 2:**
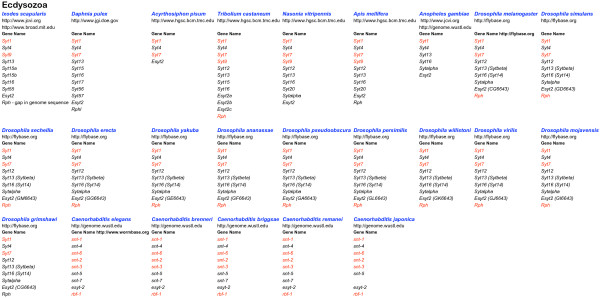
**Summary of the genes collected from ecdysozoan genomes**. The websites of the organisations which sequenced the genome or which provide access to multiple genomes within a single genus, are listed below the organism name. Underneath the Gene Name heading, gene symbols are listed. Red symbols indicate sequences containing all ten acidic amino acid positions required for function as a calcium trigger for exocytosis. Websites for the relevant nomenclature authorities are listed alongside the Gene Name heading. Gene symbols within brackets are currently officially approved, but in conflict with the nomenclature proposed here.

**Figure 3:**
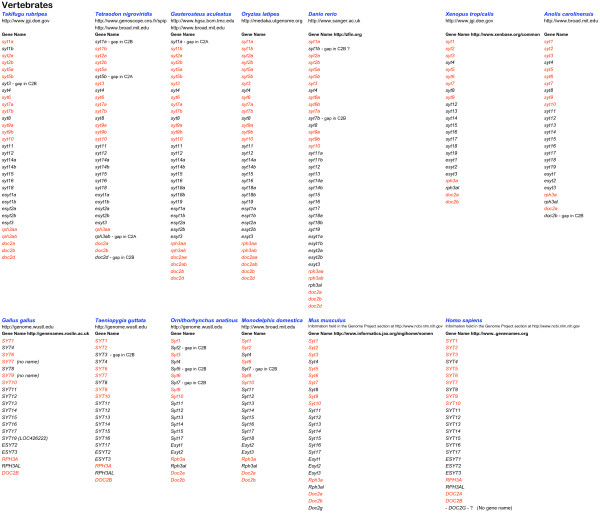
**Summary of the genes collected from Vertebrate genomes**. The websites of the organisations which sequenced the genome or which provide this information, are listed below the organism name. Underneath the Gene Name heading, gene symbols are listed. Red symbols indicate sequences containing all ten acidic amino acid positions required for function as a calcium trigger for exocytosis. Websites for the relevant nomenclature authorities are listed alongside the Gene Name heading. Gene symbols within brackets are currently officially approved, but in conflict with the nomenclature proposed here.

On finding uniquely metazoan *Syt *homologues, with their characteristic gene structures, in the genomes of *T. adhaerens *and *N. vectensis *[50,51] I decided to compile afresh, the *Syt *repertoire of the currently available genomes of evolutionary significance. In addition to *Syt *genes, I set out to gather *Esyt*, *Rph3a*, *Rph3al *and *Doc2 *homologues. These gene families, while clearly distinct from the *Syt *family at the gene structure level, encode proteins with high level sequence similarity to the tandem C2 domain region of Syt (*Rph3a *and *Doc2*) or with similarity, in common with NTMC2 proteins, at a more crude domain architecture level (*Esyt*). Because of these similarities in form, there will be common functions. A collection like this can be used to further define these functions, by revealing the patterns of sequence conservation (indicating selection and functional importance) and sequence divergence, apparent in: 1. the individual domains of an orthologous group of proteins, 2. the equivalent domain in different groups, not necessarily homologous. Such knowledge can provide clues about how these proteins function as molecular machines in cells and about how these functions have developed over evolutionary time. I used the annotation facilities at DOE-JGI to create and annotate gene models where possible. Full details of all genes are listed in additional files 1, 2, 3 and 4. Figures 1, 2 and 3 summarise the genes in additional files 1, 2 and 3 respectively.

### Gene identification

I searched for homologues among the metazoan genome sequences at DOE-JGI [46] NCBI [52] and UCSC [53]. I employed a variety of methods, first to identify, then to piece together, gene sequences for homologues of *Syt*, *Esyt*, *Rph3a*,*Rph3al *and *Doc2*. These are generally large, multi-exon genes which encompass regions of high (the C2 domain region of Syt proteins for example) and low (the N-terminal portion of Syt proteins for example) levels of sequence conservation. As a first step, I used blatp [54] or tblastn [55] with amino acid sequence probes representing already known homologues, to identify genomic loci with potential to encode a similar protein. I then examined a 3-frame translation [56] of the genomic region to look at the possible exon-intron structure. Intron positions and phases are generally well conserved among these genes and serve as a useful diagnostic tool. In cases where the draft genome sequence was highly fragmented, homologues were pieced together exon by exon. Where sequence conservation between amino acid probe and novel genomic locus was high, it was straightforward to identify exons and introns. Where sequence conservation was low, the conservation of intron position and phase could serve as a guide to judge possible exon boundaries. In all cases, when transcript sequences from the locus under examination, or from a homologous locus in a closely related organism, were available, these were used to help identify exons. Occasionally, trace archive reads were sought to confirm exon sequences, where there was a gap or where there appeared to be frameshift errors in a section of draft sequence for example. Details of such instances are noted in additional files 1, 2 and 3.

Where transcript sequences indicated the expression of alternative variants, I collected the variants. In the case of *M. musculus *and *H. sapiens*, where there is abundant transcript variation, particularly at certain genomic loci, I collected only those variants indicated by two or more transcript sequences. Where evidence of alternative splicing exists but is not represented by full length transcripts (the majority of cases) the resultant gene predictions require validation, as do all gene predictions for which no transcript evidence exists. For genomic loci with conserved variant exons, which could be employed to extend the length of the encoded protein but where transcript evidence was lacking, the gene prediction includes all of the conserved exons. Transcript evidence for the gene predictions is listed in additional files 1, 2, 3 and 4.

Once I had gathered a set of crude gene predictions, I compared the sequences in order to refine the predictions. Exon-intron junctions, gaps, novel regions of sequence conservation useful as probes to fill gaps, were all carefully examined using multiple alignments [57]. In this way, it was possible to extend and improve the gene predictions significantly. Many of the gene predictions in this collection are still incomplete, at the N-terminal region of Syt proteins in particular. When there was no transcript coverage and no detectable sequence conservation or very short coding exons, it was not possible to make a prediction. All incomplete predictions are noted in additional files 1, 2, 3 and 4.

### Sequence comparison: global comparison and nomenclature

Having collected 711 genes from 46 genomes, I used clustalw2 at EBI [58,59] to compare subsets of amino acid sequences, in order to examine their relationships of similarity overall. I compared sets which excluded expressed variants but which included one sequence per gene, whether complete or not. Comparisons of invertebrate sequences (additional file 5) vertebrate sequences (additional file 6) invertebrate (one representative per genus) plus mammalian sequences (additional file 7) and invertebrate (one representative per genus) plus a subset of vertebrate sequences (additional file 8) were used to examine relationships and assign a suitable name to each gene. I assigned a common name to those genes which clustered together in conserved groups. When a conserved group included a homologue from *M. musculus*/*H. sapiens*, the assigned name was chosen to be consistent with that in *M. musculus*/*H. sapiens*. For those *Syt *genes which did not consistently cluster together and are not conserved among different organisms, I assigned them numbers upward from the last conserved group. This process led to the identification of 22 conserved groups of *Syt *genes plus a further 35 unique *Syt *genes named with the *Syt *stem symbol plus a number identifier, in order to provide a recognisable name (albeit provisional) for each. This naming system allows further sampling of the tree of life by gene sequencing to reveal: 1. more unique *Syt *genes, from *Syt58 *upwards, all members of a recognisably homologous family and 2. previously unrecognisable patterns of conservation among *Syt *genes, from *Syt22 *upwards. I contacted the relevant organism nomenclature authorities [31,60-66] to discuss this nomenclature. This wide consultation led to agreement to change *FAM62*, a temporary HGNC symbol for this poorly characterised gene family, to *Esyt *[67]. Where current approved nomenclature is discordant with that proposed here, the approved nomenclature is indicated within brackets, in additional files 1, 2, 3 and figures 1, 2 and 3. There is inconsistency in the application of my naming rationale in *Drosophila *and *Caenorhabditis*, where it was more practical to stick with the approved nomenclature than propose new names. As yet, and in comparison to vertebrates, invertebrates are poorly represented by organised gene nomenclature authorities. My proposed nomenclature for a large number of genes in this collection, therefore, awaits sanction. Note that due to differing nomenclature conventions, the gene symbols in different organisms, range from having no capital letters to all capital letters. The generic form, which I have used to label the genes from organisms outside the purview of nomenclature authorities, is first letter in capitals. This designation will be used here, to describe the gene in general, without reference to a specific organism.

**Figure 4:**
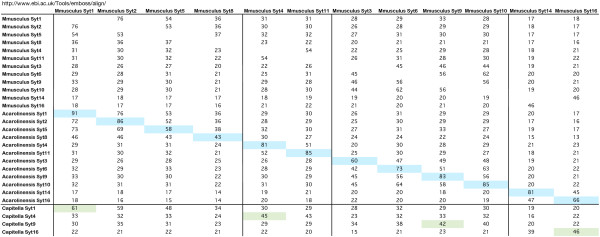
***Syt *orthologues and paralogues in *M. musculus***. Percent identity scores produced by the align facility at EBI, of pairwise comparisons of full length protein sequences, are listed. Top scores from mouse versus lizard comparisons are highlighted in blue, indicating an orthologous relationship between the mouse gene and the evolutionarily more ancient lizard gene. Top scores from comparisons between mouse and the much more evolutionarily ancient polychaete worm, *Capitella*, are highlighted in green, indicating that of these mouse genes, *Syt1*, *Syt4*, *Syt9 *and *Syt16 *are orthologous to genes in *Capitella*.

Deciding whether relationships of orthology or some other kind exist among a group of similar genes, can be complicated. The processes of duplication, deletion, and rearrangement of genes, plus the action of selection or absence of selection on gene sequences, can obscure relationships. Whether absence from a genome assembly reflects true absence, or incompleteness of the assembly, presents a further problem of some seriousness [68]. In *M. musculus *and *H. sapiens *there are several groups of duplicated *Syt *genes: group 1 [*Syt1*, *Syt2*, *Syt5*, *Syt8*] group 2 [*Syt4*, *Syt11*] group 3 [*Syt3*, *Syt6*, *Syt9*, *Syt10*] and group 4 [*Syt14*, *Syt16*]. Each group contains genes which share a distinct gene structure. Additional files 5, 6, 7, 8 and figure 4, indicate that the parental gene of group 1 is *Syt1*. The parental gene of group 2 is *Syt4*, that of group 3 is *Syt9 *and that of group 4 is *Syt16*. In each of these cases, the parental gene is more similar to a gene present in an evolutionarily more ancient genome than it is to another member of the mammalian group, which is to say, orthologous [39]. Orthologous genes in different organisms, are related by vertical descent from a common ancestor. Relevant pairwise comparison scores are listed in figure 4. It is worthwhile trying to determine the phylogenetic relationships between the *Syt *genes of mammals and those of other animals because it is the rodent Syt proteins which are best characterised functionally. However, because mammalian organisms and their brains in particular, represent biological systems of such complexity, modelling these systems by using simpler systems is essential. The relationships and nomenclature proposed here, are in good general agreement with the *Syt *data at Treefam [69,70] which does not include many of the invertebrate *Syt *genes in this collection.

This gene collection includes several previously unrecognised *Syt *groups which are absent from *M. musculus*/*H. sapiens*. Members of the *Syt18 *group appear in vertebrates and invertebrates, but the *Syt19 *group has yet to be found beyond vertebrates. Neither of these groups are well conserved, both genes having diverged significantly over time. The *Syt18 *group ranges from members encoding: tandem C2 domains (*S. purpuratus*, *B. floridae*, *A. carolinensis*) a single C2B domain (*L. gigantea*, *X. tropicalis*, the fish genomes) or no C2 domains at all (the *Syt18b *duplicates in the fish genomes). *Syt20 *appears in only two hexapod species in this collection. The other *Syt *groups absent in vertebrates, are *Sytalpha *and *Syt21*. Of these, the *Sytalpha *group has a higher level of sequence conservation. The *Sytalpha *designation applies to all members of the conserved group which contains the *D. melanogaster Sytalpha *gene (see additional file 5). There are many invertebrate *Syt *genes, related to some degree, at the gene structure level, which are not conserved among different phyla (*Syt22 *to *Syt57 *in this collection). *N. vectensis*, for example, has a very large number ofunique and divergent *Syt *genes (*Syt24 *to *Syt43*). Most of these encode stereotypical Syt proteins, but some have only one C2 domain and some lack a TM domain (as noted in additional file 1). The *Caenorhabditis Syt *repertoire, in common with many others in this collection, demonstrates that variant Syt proteins which lack the TM domain, are usually included. They are encoded either by separate genes or by alternative splicing. *Caenorhabditis snt-3 *is very similar to *snt-1 *at the amino acid sequence level, but does not encode a TM domain. In the *Ciona Syt *repertoire, it is *Syt16 *which produces a variant lacking the TM domain, but by alternative splicing. In mammals, *Syt17 *does not encode a TM domain, but a number of other mammalian *Syt *genes which do encode a TM domain, express splice variants which lack the TM domain (*Syt5*,*Syt6*,*Syt8*,*Syt9*,*Syt10*,*Syt12*,*Syt13*,*Syt15*,*Syt16*). Transcript variants are listed in additional files 1, 2, and 3.

### Sequence comparison: gene duplications

The duplicate genes in the fish genomes presented a nomenclature challenge and I am very grateful for help from the Zfin nomenclature coordinator [62,63] in choosing the most appropriate names for these genes based on gene structure similarity and synteny. A number of genes present as a single copy in mammals (*Syt7*, *Syt9*, *Syt14*, *Esyt2*, *Rph3a*) are present as duplicates in the fish genomes. In addition, *Syt6 *and *Syt11 *are duplicated in *D. rerio *(figure 3). It is notable that the single *Rph3a *of tetrapods appears to have undergone duplication and fission, giving rise to *Rph3al *and *Doc2b *genes [as noted in 33]. *Rph3al *and *Doc2b *are linked as a tandem pair in tetrapod genomes (noted in additional file 3). Invertebrates have a single, equivalent *Rph*, but no *Doc2 *genes. It is possible that *D. pulex Rphl *represents an N-terminal *Rph *fission product. In the fish genomes, there are duplicate *Rph3a *genes, but only in the lineage leading to *D. rerio*, has a supposed *Rph3a *duplication led to the retention of linked *rph3al *and *doc2b *fission products (noted in additional file 3). In the other fish genomes, *doc2b *genes are present, along with the paralogous *doc2a *and *doc2d *genes, as well as duplicate *rph3aa *and *rph3ab *genes, so *Doc2b *genes are not always linked to a *Rph3al *fission product. Apart from the gene duplications already noted, other paralogous gene expansions are evident in this collection (figures 1, 2 and 3). In the case of *Esyt *gene duplications, retroposition of processed transcripts has occasionally led to gene duplication (*N. vectensis Esyt2a *and *X. troplicalis Esyt2b*, as noted in additional files 1 and 3).

### Sequence comparison: multiple alignments

In order to illustrate the conserved features of the proteins in this collection, I used Multalin [57] to construct multiple alignments of subsets of sequences, including expressed variants (additional files 9, 10, 11, 12, 13, 14, 15, 16, 17, 18, 19, 20, 21, 22, 23, 24, 25, 26, 27, 28, 29, 30, 31, 32, 33, 34, 35, 36, 37, 38, 39, 40, 41, 42, 43, 44, 45, 46, 47, 48, 49 and 50). The conserved groups of vertebrates and invertebrates are compared separately but the *Syt18 *group (additional file 37) contains members from vertebrates and invertebrates. All of the Syt sequences from *N. vectensis *(additional file 41) are compared. These multiple alignments illustrate the patterns of sequence conservation (indicating the action of selection) distinctive of different groups. Common patterns of alternative splicing which serve to alter particular domains, are also illustrated. Where calcium coordinating residues are absolutely conserved (indicating the action of purifying selection on calcium binding activity) these are indicated by arrows. These absolutely conserved sequences are highlighted in red in figures 1, 2 and 3. In the many more cases where calcium coordinating positions are not absolutely conserved, there is a range, down to zero conservation, of the ten sites present in the absolutely conserved, calcium responsive Syt proteins. A conserved region 20-50 residues upstream of the C2A domain, often recognisable as a variant of this motif: GRIKPELY is indicated where present. Conservation of this motif was noticed previously [30] but it is obvious here, that this region is much more widely conserved. Its function is yet to be investigated. The position and phase of introns are also illustrated, in order to show the gene structures distinctive of different family groups, as well as instances of intron mobilization. Intron mobilization within a genus is specifically noted in additional file 2. Intron phase is indicated by colour: black for phase 0, red for phase +1 and blue for phase +2.

### Syt1 homologues

Additional file 9 shows a multiple alignment of the invertebrate Syt1 protein sequences in this collection. The ten conserved calcium coordinating positions are indicated by arrows. The C2AB region is highly conserved whereas the N-terminal portion has much more sequence variation. Expressed variants are included to demonstrate the regions altered by alternative splicing and RNA editing. The region between the TM domain and the C2A domain is commonly altered, for instance in *S. purpuratus*, *B. floridae*, *A. gambiae *and *D. melanogaster*. The next region expressed as alternative forms, is the region between C2A and C2B, as indicated by *D. pulex *and *A. gambiae *variants. The third region to be altered, by alternative splicing or by RNA editing, is the central portion of the C2B domain. In *Caenorhabditis*, this recoding alters not only the identity of positions within this exon of 45 amino acids length in all other cases, but extends its length by 2 or 3 amino acids. Additional file 9 also includes the snt-3 sequences from *Caenorhabditis*. The *snt-3 *gene encodes a protein very similar to the snt-1 protein which is the Syt1 orthologue. The snt-3 proteins however, lack the N-terminal membrane anchoring portion. The snt-3 proteins also differ in the region between C2A and C2B.

Additional file 10 shows a multiple alignment of the vertebrate Syt1 protein sequences in this collection. The ten conserved calcium coordinating positions are indicated by arrows. The C2AB region is highly conserved whereas the N-terminal portion has a little more sequence variation. Expressed variants are included to demonstrate the regions altered by alternative splicing. This time, the alteration is restricted to the region between the TM and C2A domain. Conserved N-glycosylation consensus sites are indicated by blue boxes. In the first coding exon, the N-glycosylation site and the conserved upstream O-glycosylated threonine, are known to be important functional sites *in vivo *[25,71,72].

**Figure 5:**
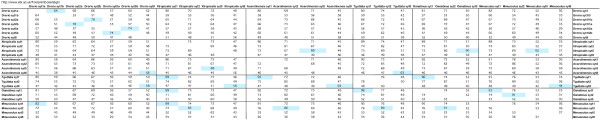
**Pairwise comparisons of *Syt1 *paralogues in vertebrates**. Percent identity scores produced by the align facility at EBI, of pairwise comparisons of full length protein sequences, are listed. Top scores are highlighted in blue, indicating an orthologous relationship between the compared genes.

**Figure 6:**
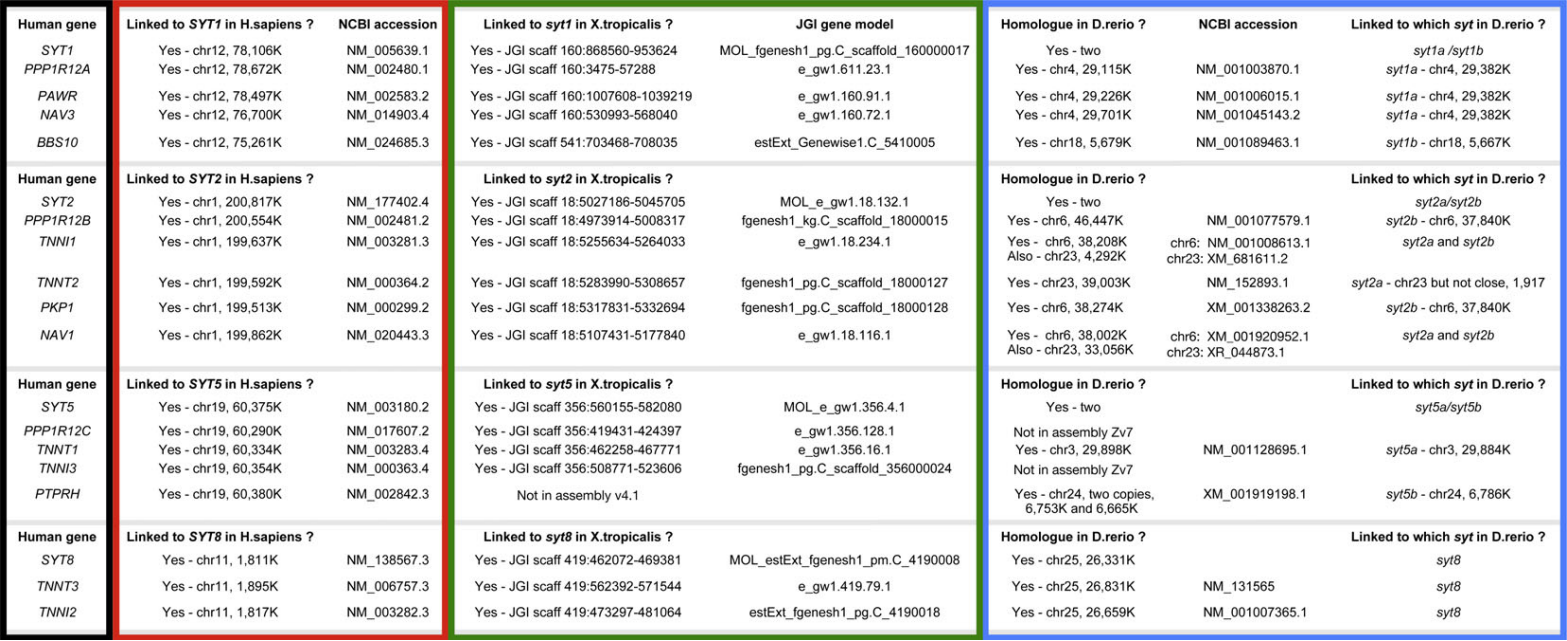
**Synteny of *Syt1 *paralogues in *D. rerio*, *X. tropicalis *and *H. sapiens***. Gene symbols for four groups of neighbouring genes in *H. sapiens*, are enclosed by a black box. Within the red box, the chromosomal locations of each gene in the current human genome reference sequence, are indicated along with a reference transcript sequence. The genomic locations and transcript sequences for the *X. tropicalis *gene relatives are within the green box and those for *D. rerio *are within the blue box.

Additional files 11, 12 and 13 show multiple alignments of the vertebrate Syt2, Syt5 and Syt8 protein sequences, respectively. For *Syt2*, *Syt5 *and *Syt8 *genes, there is transcript evidence of alternatively expressed forms which lack the TM domain. *Syt1*, *Syt2*, *Syt5 *and *Syt8 *are all related by duplication, with *Syt1 *being the parental gene (figure 4). In the fish genomes, there are yet more duplicates. In order to distinguish the relationships among these duplicates, pairwise comparison scores (figure 5) and syntenic relationships (figure 6) were examined. The top scores highlighted in blue in figure 5, indicate that *D. rerio syt1a *is orthologous to the *Syt1 *gene of other vertebrates and that *D. rerio syt8 *is orthologous to the *Syt8 *gene of other vertebrates. The pairwise comparison scores do not however, indicate orthology for the other duplicates. Figure 6 shows that relationships of orthology, are indeed present. The neighbouring genes of *Syt1*, *Syt2*, *Syt5 *and *Syt8*, in the genomes of *H. sapiens*, *X. tropicalis *and *D. rerio*, reveal conserved syntenic blocks in each genome. This synteny indicates that the four genomic segments share ancestry. Orthologous relationships exist between: *H. sapiens SYT1 *and the duplicated *syt1a*/*syt1b *in *D. rerio*, between *SYT2 *and *syt2*/*syt2b *and between *SYT5 *and *syt5a*/*syt5b*, respectively. Since there is so much interest in the mechanism of Syt1 function, close examination of the Syt1 orthologues and paralogues in this collection, to see how they have been shaped by evolution, may prove productive.

### Syt4 homologues

Additional file 14 shows a multiple alignment of the invertebrate Syt4 protein sequences in this collection. The variants of *D. pulex *differ at the C-terminal end and those of *A. gambiae *differ at the N-terminal end. Additional file 15 shows a multiple alignment of the vertebrate Syt4 protein sequences. The variants of *G. gallus *differ in the region between C2A and C2B. Additional file 16 shows a multiple alignment of the protein sequences of *Syt11*, the vertebrate paralogue of *Syt4*. Here, the variants in *G. gallus*, *M. musculus *and *H. sapiens *differ in the C2B domain. Recent functional studies of mouse *Syt4 *[20,21] have built on initial gene knockout results [73] which suggested that SYT4 dysfunction could be responsible for some human psychiatric disease. SYT11 dysfunction has also been implicated in human psychiatric disease [74].

### Syt7 homologues

Additional file 17 shows a multiple alignment of the invertebrate Syt7 protein sequences in this collection. Here, there is evidence from *D. melanogaster*, of variant forms lacking the TM domain. Additional file 18 shows the vertebrate Syt7 sequences. In comparison to the invertebrate *Syt7 *genes, the vertebrate *Syt7 *genes contain additional coding capacity in the region between the TM and C2A domains. Several additional exons which can be alternatively spliced, are available to extend the length of the region linking the TM and C2A domains. A study of constitutive versus alternative splicing, has examined the SYT7 alternative exons [75]. Recent gene knockout studies in mice [76,77] appear to confirm a role for *Syt7 *in glucose homeostasis.

### Syt9 homologues

Additional file 19 shows a multiple alignment of the invertebrate Syt9 protein sequences in this collection. The conserved motif upstream of the C2A domain is indicated. Additional file 20 shows the vertebrate Syt9 sequences. The variants expressed by *M. musculus *and *H. sapiens *differ at one or other end. Additional files 21, 22 and 23 show the protein sequences of the vertebrate *Syt9 *paralogues, *Syt3*, *Syt6 *and *Syt10*, respectively. All of these proteins retain all ten calcium coordinating residues and are thus likely to have been selected to act as calcium sensors. They also retain the motif of unknown function upstream of the C2A domain. Transcript evidence from *X. tropicalis*, *M. musculus *and *H. sapiens *indicates the expression of alternative forms of *Syt6 *which lack the TM domain. The C-terminal region of *Syt6 *is also expressed as alternative forms. Functional studies have examined the conserved cysteine residues of the N-terminal region which are unique to this paralogous family in vertebrates [78]. Syt6 is thought to play a role in sperm acrosomal exocytosis [79]. Mouse Syt9 has been identified as upregulated during meiosis in fetal ovaries [80]. Note that some investigators working with Syt proteins, use different synonyms for the protein products of certain *Syt *genes. The officially approved gene nomenclature is used throughout this paper. In the functional literature, the protein products of *Syt5 *and *Syt9 *have most often been referred to differently [81]. *Syt16 *and *Syt17*, so far poorly characterised, have also been prone to a similar lack of recognition [6,81].

### Syt12 homologues

Additional file 24 shows a multiple alignment of the invertebrate Syt12 protein sequences in this collection. While there is some variation in length, of sequences from different organisms in the C2B region, the more pronounced difference is a trend towards increased length of the region between TM and C2A domains in insects. Additional file 25 shows the vertebrate Syt12 sequences. Transcript evidence indicates the expression of forms varying in the N-terminal region in *H. sapiens*. Little functional work has been done so far with *Syt12 *[19,82].

### Syt13 homologues

Additional file 26 shows a multiple alignment of the invertebrate Syt13 protein sequences in this collection. In most of these genes, there are two alternative N-terminal coding exons, only one of which encodes a TM domain. These sequences also retain the motif of unknown function just upstream of the C2A domain. Additional file 27 shows the vertebrate Syt13 sequences. Transcript sequences from *H. sapiens *indicate the expression of a form lacking the TM domain. A recent study suggests that human *SYT13 *may function as a tumour supressor [83].

### Syt15 homologues

Additional file 28 shows a multiple alignment of the invertebrate Syt15 protein sequences in this collection. In these sequences, it is the C2A domain which is more highly conserved than the C2B domain. The motif upstream of the C2A domain is present. There is a large amount of variation in amino acid sequence, intron position and intron phase in the N-terminal portion of these sequences. Additional file 29 shows the vertebrate Syt15 sequences. Transcript evidence from *M. musculus *and *H. sapiens *indicates the expression of variants differing at the C-terminal end. Transcripts from *H. sapiens *indicate variant forms at the N-terminal end, which lack the TM domain. Very little is known about the function of *Syt15 *[19].

### Syt16 homologues

Additional file 30 shows a multiple alignment of the invertebrate Syt16 protein sequences in this collection. Transcript evidence indicates the expression of variants in *C. savignyi*, *C. intestinalis *and *S. purpuratus *which differ at the N-terminal end and lack the TM domain. Additional file 31 shows the vertebrate Syt16 sequences which contain a TM domain. Additional file 32 shows the vertebrate Syt16 sequences which lack a TM domain. Additional file 33 shows vertebrate Syt14 sequences. Transcript evidence in *X. tropicalis*, *M. musculus *and *H. sapiens *indicates the expression of variant forms of Syt14 with altered C2B domains. Human *SYT14 *transcripts also differ at the N-terminal region, resulting in forms which lack the TM domain. Loss of function of human *SYT14 *has been associated with neurodevelopmental abnormalities [84]. In the human genome, there is a repeat of a portion of the *SYT14 *gene (which is on chromosome 1) on chromosome 4, apparently the result of retroposition of a *SYT14 *transcript. The chromosome 4 locus has been named *SYT14L *by HGNC [31]. *SYT14L *is classed as a pseudogene by NCBI (GeneID: NR_027094) and is not included in this collection.

### Syt17 homologues

Additional file 34 shows a multiple alignment of the invertebrate Syt17 protein sequences in this collection. A possible motif upstream of the C2A domain is indicated. Additional file 35 shows the vertebrate Syt17 sequences. Transcript evidence in *M. musculus *and *H. sapiens *indicates the expression of variants altered at N-terminal and C-terminal ends. A possible motif upstream of the C2A domain is indicated. The protein product of Syt17 has been referred to as B/K protein, but not much is known about its function [85].

### Sytalpha homologues

Additional file 36 shows the Sytalpha sequences in this collection. A possible motif upstream of the C2A domain is indicated. The only functional study relevant to Sytalpha is its anatomical localisation in *D. melanogaster *[27].

### Syt18 homologues

Additional file 37 shows the Syt18 sequences in this collection. These proteins are so far completely uncharacterised.

### Syt19 homologues

Additional file 38 shows the vertebrate Syt19 sequences in this collection. These proteins are so far completely uncharacterised.

### Syt21 homologues

Additional file 39 shows the invertebrate Syt21 sequences in this collection. These proteins are so far completely uncharacterised.

### Syt homologues in *N. vectensis*

Additional file 40 shows all of the Syt proteins of *N. vectensis*, demonstrating enormous sequence variation. These proteins are so far completely uncharacterised.

### Dblc2 homologues

Additional file 41 shows the Dblc2 sequences in this collection. Transcript variants are indicated. These proteins have not previously been reported and are so far completely uncharacterised. Dblc2 sequences are detectably similar to Syt sequences at the amino acid sequence level and occur in the genomes of marine invertebrates in this collection. While they lack an N-terminal TM domain, they have a tandem C2 domain architecture in common with Syt proteins. *Dblc2 *genes lack similarity to *Syt *genes at the gene structure level, so a new gene symbol was chosen to represent these genes. The Dblc2 designation represents a fuller description: double C2 domain protein. Dblc2 is thus similar but recognisably different from Doc2, the designation for the homologous family of double C2 domain proteins of vertebrates.

### Esyt homologues

Additional file 42 shows the invertebrate Esyt2 sequences in this collection. Transcript variants are indicated. The arthropod *Esyt2 *genes include mutually exclusive alternative exons which serve to alter the specification of the first C2 domain. Additional file 42 shows the sequence of the first of these alternatives only. Additional file 43 shows all versions of this alternatively coded region. Additional file 44 shows the vertebrate Esyt1 sequences. Transcript variants are indicated. In the fish genomes, *Esyt1 *has duplicated, resulting in *esyt1a *and *esyt1b*. The *esyt1b *duplicate has acquired an internal duplication which contributes an additional four C2 domains to the product of this gene [33]. Within this duplicated section, the pink dot marks the position of an apparent phase 0 intron loss in the tetraodontiform fish. In each of the three other occurrences (two prior, one subsequent) of this portion of the *esyt1b *gene, the phase 0 intron is present. This intron is also present in each of the two repeats of this sequence, in the *esyt1a *genes. Additional file 45 shows the vertebrate Esyt2 sequences. Transcript variants are indicated. In the fish genomes, *Esyt2 *has duplicated, resulting in *esyt2a *and *esyt2b*. Additional file 46 shows the vertebrate Esyt3 sequences. Transcript variants are indicated.

Similarity between *Syt *genes and what are now named *Esyt *genes, was first evident from genome sequence comparisons [29,30]. In trying to classify and annotate the genes within the human genome, HGNC [31] noticed that these genes formed a distinct gene family, separate from *Syt *genes. As there was little functional data associated with these genes, a temporary nomenclature with no functional implication was provided: *FAM62A*, *FAM62B *and *FAM62C*. The functional data which did exist, was in the form of a cDNA representing Rat *Esyt1*, which had been cloned during an investigation of adipocyte proteins and found to encode a membrane bound C2 domain protein with similarity to proteins in plants [86]. Further genome sequence comparisons indicated that *Syt*, *FAM62 *and plant *NTMC2 *genes encode proteins with amino acid sequence similarity and domain architecture similarity, but belong to distinct gene families [33]. Apart from [86] and until recently, functional data associated with plant *NTMC2 *and animal *FAM62 *gene families had been lacking. An initial investigation of proteins encoded by the Human *FAM62A*, *FAM62B *and *FAM62C *genes, has now been published and the authors named these proteins Esyt1, Esyt2 and Esyt3, to stand for extended synaptotagmin-like proteins [67]. Consultation with the nomenclature committees during the preparation of this paper, led to the decision to implement the *Esyt *nomenclature in place of *Fam62*. In an initial pair of studies on one member of the plant *NTMC2 *gene family [34,35] the authors put forward the name SYT1 for this particular gene and gene product. As discussed in the background section of this paper, when different communities make naming decisions based on the criteria most relevant to them (gene phylogeny, protein structure, biological function) it is inevitable that multiple names will be used to identify the same entities.

### Rph3a homologues

Additional file 47 shows the invertebrate Rabphilin sequences in this collection. In *Caenorhabditis*, alternative N-terminal exons can be used. Sequence conservation among Rabphilins is high in the N-terminal, Rab binding portion [87] and the C-terminal tandem C2 domain portion, but a large middle portion is very poorly conserved. This means that where transcript confirmation is absent, the gene predictions across this portion are unlikely to be accurate. Additional file 48 shows the vertebrate Rph3a sequences. Transcript variants are indicated. In vertebrates, all 10 calcium coordinating positions are absolutely conserved, but not all are conserved in invertebrates. In additional file 47, the positions of these 10 amino acids are indicated by pink dots. The position of the fifth calcium coordinating residue in the C2A domain is not strictly conserved, but in most cases a suitable aspartate or glutamate residue is present one residue earlier. I am not aware of biochemical evidence for calcium binding by invertebrate Rabphilin proteins, but genetic evidence [88] suggests that *C. elegans rbf-1 *at least, appears to function similarly to its mammalian homologues. For this reason, in figures 1, and 2, I have counted the fifth position as present when a suitable aspartate or glutamate residue occurs at the usual spacing or one residue earlier.

### Rph3al homologues

Additional file 49 shows vertebrate Rph3al sequences. Transcript variants are indicated. In the functional literature, products of *Rph3al *genes have been referred to as Noc2 [89,90]. As outlined above, *Rph3al *genes appear to be the result of duplication and fission of an ancestral *Rph3a *gene, producing linked *Rph3al *and *Doc2b *genes in tetrapods and in *D. rerio*. *Rph3al *represents the N-terminal portion of the ancestral *Rph3a *gene and *Doc2b *represents the C-terminal portion, as seems clear from a comparison of the gene structures of *Rph3a*, *Rph3al *and the linked *Doc2b *genes (additional files 48, 49, and 50). As also indicated above, *Doc2b *genes do not always accompany *Rph3al *genes, as they exist in other fish genomes where *Rph3al *genes do not occur (see figure 3).

### Doc2 homologues

Additional file 50 shows the vertebrate Doc2 protein sequences. Transcript variants are indicated as well as the positions of the calcium coordinating residues, which are conserved in all of the Doc2 proteins except Doc2g. I have included potential Doc2g products from the human gene locus. Although spliced transcripts are produced from this locus, they cannot encode full length proteins and it is not known whether protein products are translated from these transcripts. It is clear from the shared gene structure of *Doc2 *genes and *Rph3a *genes, that *Doc2 *genes represent the C-terminal portion of a *Rph3a *gene. Since *Rph *genes exist in invertebrates, but *Doc2 *genes do not, it seems reasonable to suggest that *Doc2 *genes arose via duplication and fission of a *Rph3a *gene. Evidence for this, in the form of the two linked fission products (*Rph3al *and *Doc2b*) is present in the genomes of *D. rerio*, *X. tropicalis*, *A. carolinensis*, *G. gallus*, *T. guttata*, *O. anatinus*, *M. domestica*, *M. musculus *and *H. sapiens*. In this collection, the fish genomes reveal a novel lineage of *Doc2 *genes, which in consultation with Zfin staff [62,63] were given the gene symbol *Doc2d*, to stand for Doc2 delta. The first *Doc2 *gene, cloned in 1995 [91] was soon found to represent the first member of a family of three genes in rodents. The protein products of these three genes were named Doc2 alpha, Doc2 beta and Doc2 gamma [92]. The conserved region present in the N-terminal portion of Doc2 sequences, specifies the Munc13 binding capacity of Doc2 proteins [93].

## Conclusions

This paper describes a manually curated collection of genes pertinent to intercellular trafficking in multicellular animals. The collection includes 141 genes from the genome sequences of 9 marine invertebrates (additional file 1) 215 genes from the genome sequences of 24 ecdysozoans, including the 12 *Drosophila *and 5 *Caenorhabditis *genomes (additional file 2) plus 355 genes from 13 vertebrate genomes (additional file 3). When viewed overall (figures 1, 2 and 3) the collection implies that particular homologues which are always present in the genome repertoire, serve to specify functions required for animal life. The basis for this view is that these genes have not been lost, but have been retained and conserved, over the long period of metazoan evolution. Such a view about the essentiality of gene function, differs from a synthetic biology view, which considers the genes necessary to specify an operable system which functions outside the natural world. It differs too, from a reductionist molecular biology view where, if a gene can be deleted in the laboratory setting, without killing the organism, it is not considered essential. Such gene deletion experiments have been done with *Syt1 *in different organisms [94-96] and according this view, *Syt1 *is not strictly essential to animal life because *snt-1 *null mutants are not lethal in *Caenorhabditis*. In *Drosophila *and *Mus *however, *Syt1 *null mutants are indeed lethal as they cannot survive to reproduce as adults. While my previous genomic comparison of *Syt *genes [30] could have been interpreted as indicating a correlation between increased numbers of *Syt *genes and increased organism complexity [33] the analysis here, which is much more comprehensive, definitively rules that conclusion out. This collection of genes offers prospects for fruitful speculation about the functional roles of the *Syt *repertoires of different animals and includes a great range of biological complexity. The conclusions drawn from evolutionary genomics approaches are always provisional, because they depend on the quality of information available (draft or 'complete' genome sequence, availability of transcript information) and are thus subject to review when better information becomes available. Conclusions drawn from reassembled and reannotated genomes will inevitably be more accurate than those drawn from the first draft. The *D. rerio *genome being repeat rich, is taking time to assemble into a complete version. Among the genomes in this collection, the draft *D. rerio *genome ranks first in terms of gene number. *D. rerio *surely presents a useful model system with which to investigate the functional divergence of close paralogues [41].

Currently, it is the rodent Syt proteins and, with the advent of transgenic mouse studies, their respective genes, which garner most interest and have been best characterised. Work with *Syt *genes in other animal species has provided information supporting hypotheses about how *Syt *genes fulfil their functional roles [94,95,97,98]. To date, these studies indicate a primary role for *Syt1 *in specifying a neuron specific synaptic vesicle protein which serves to trigger exocytosis. The genomic specification of *Syt1 *expression patterns, has been investigated and utilised in ascidian species [99-102]. In *C. elegans*, the anatomical expression patterns of the mutually exclusive alternative exon variants of Syt1 have been mapped [103]. This gene collection shows that such mutually exclusive exon deployment has arisen on a number of occasions in invertebrates, altering C2 domains in Syt1, Syt34 and Esyt2 proteins (see additional file 5). The repeated evolution of this type of C2 domain alteration, suggests that careful comparison of the altered forms might prove a useful focus for investigations aimed at understanding how these C2 domains operate as molecular machines. The feature most widely shared among the great diversity of Syt proteins in this collection, is sequence conservation of the C-terminal C2B region, which suggests that a defining function of Syt proteins is located here.

The regulatory machinery controlling variant expression can be illuminated by comparative analyses of genomic sequences, as was done to investigate the RNA editing of *Syt1 *which occurs in hexapods [104]. Another comparative analysis [105] examining the genomic specification of alternative splicing of *Syt1*, concluded that splice variants expressed in *D. melanogaster*, *C. pipiens*, *B. mori*, *T. castaneum *and *P. humanus *depend on correctly coded intronic, *cis*-acting regulatory sequences acting on the requisite splice donor/acceptor sequences. In this collection, there is evidence from *A. gambiae*, *D. melanogaster*, *D. pulex*, *S. purpuratus*, *B. floridae*, *D. rerio*, *X. tropicalis*, *A. carolinensis*, *G. gallus*, *M. musculus *and *H. sapiens *of RNA editing and alternative splicing of *Syt1 *(additional files 1, 2, 3, 9 and 10). These sequences may aid future efforts to identify the *cis*-sequences involved in transcript processing. The biochemical understanding of transcript production and processing, acquired at great pains over many years [see [43]] is not quite up to the task of extracting the full meaning from the flood of new transcript data from high throughput sequencing. An enormous amount of experimental work will need to be done to understand the varied roles of transcripts in complex organisms. The problem of identifying and annotating different kinds of transcript sequences is discussed in [106]. Work to assess the functional significance of alternatively expressed *Syt *genes has barely begun [103,107-112] so it is difficult at present, to judge the importance of the transcript variants listed in this collection. Evolutionary conservation of variant production, probably argues for functional significance. New developments in mass spectrometry [113] are bound to aid attempts to examine complex mixtures of proteins such as synaptic vesicle preparations [114,115]. It is more likely however, that the functional impact of Syt protein variants will eventually be revealed by research focussed on Syt proteins of known importance.

The significance of the conservation of intron position and phase within *Syt *genes could be investigated by gene targeting in transgenic mice, now a common route to investigate *Syt *roles [6,21]. The best conserved intron, a signature feature of *Syt *genes, is the phase +1 intron at the start of the third beta strand of the C2B domain [29,33,36] (additional files 9, 10, 11, 12, 13, 14, 15, 16, 17, 18, 19, 20, 21, 22, 23, 24, 25, 26, 27, 28, 29, 30, 31, 32, 33, 34, 35, 36, 37, 38, 39, 40 and 41). A single point mutation to inactivate this intron acceptor dinucleotide would be predicted to have devastating consequences for the expression of the host gene. In contrast to the approach of disabling a gene by creating a large deletion, the production of a null mutant by such a small intervention would be unlikely to cause collateral damage. The chances of cryptic splice acceptors being deployed to restore normal function to such a targeted *Syt *splice site point mutant, seem remote, based on the importance of this region to Syt protein function. The presence of this intron in so many *Syt *genes could be interpreted as evidence that evolutionary alteration of this site is hazardous.

**Figure 7:**
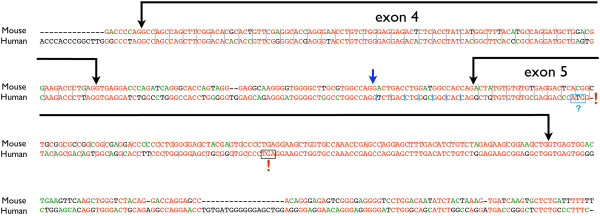
**Genomic sequence surrounding coding exons 4 and 5 in mouse *Doc2g *and the equivalent human region**. Genomic sequence surrounding coding exons 4 and 5 of mouse *Doc2g *and the equivalent region in human, are shown. Exonic and intronic sequences are indicated. The reading frame is indicated by grey bars between codons. The single nucleotide deletion in the human exon 5 region is highlighted with a red exclamation mark. The deletion in human exon 5 disrupts the reading frame, leading to a premature termination codon which is boxed and highlighted with a red exclamation mark. An alternative splice acceptor dinucleotide which could restore the correct reading frame to human exon 5 is indicated by a blue arrow. Blue bars between the first few codons indicate the reading frame were the alternative splice acceptor to be functional. Human exon 5 has an alternative start codon, which is boxed and highlighted with a question mark. There is a possibility that this start codon could allow normally spliced transcripts to be translated into the N-terminal and C-terminal protein products listed in additional file 50.

An example of a gene inactivating mutation which is not rescued by altered splicing, is present in this gene collection. The *H. sapiens *gene which is orthologous to *M. musculus Doc2g *has acquired a frameshift mutation in the fifth coding exon which introduces a stop codon. Human transcript sequences indicate that an alternative intron acceptor dinucleotide capable of restoring the reading frame, which exists 20 nucleotides upstream of the exon 5 acceptor, is not used. The regulatory sequences necessary to select this alternative acceptor are not present within this short intron (figure 7). It is not known what protein products, if any, are produced by this gene in *H. sapiens *and it appears likely that the human gene is non functional. This observation underscores the importance of splicing signals which are poorly understood at present, as well as the importance of transcript analysis to verify gene predictions.

In sea urchin species, the Syt1 protein has been shown to act in cortical granule exocytosis [98]. *Syt1 *products are used as pan neuronal markers in echinoderms, hemichordates and urochordates [25] and the role of *Syt1 *in synaptic vesicle exocytosis is assumed. Few comparative studies have attempted to map the expression patterns of the *Syt *repertoire of a particular organism but all of the *Syt *genes of *D. melanogaster *[27] and a large fraction of those of *R. norvegicus *[28] have been examined in this way. *T. adhaerens *is thought to lack cells equivalent to neurons, but it has a well conserved *Syt1 *as well as genes encoding the requisite neuronal SNAREs and complexin for fast neurotransmission (additional file 4). Although never observed in the laboratory, *T. adhaerens *is expected to produce gametes in the wild [116,117] so a role for the *T. adhaerens Syt1 *similar to that of sea urchin *Syt1 *in cortical granule exocytosis is possible, alongside any yet to be discovered 'neural' role. *N. vectensis*, which has a bona fide nervous system, also has *Syt1 *and the requisite neuronal SNAREs and complexin (additional file 4).

*N. vectensis *is quite unusual among the metazoans collected here, in having so many highly divergent *Syt *genes (additional file 41). Of all the *N. vectensis Syt *genes, only *Syt1 *and *Syt7 *encode all ten calcium coordinating positions, indicating their likely role in calcium triggered membrane fusion. Future studies to determine the anatomical expression patterns of the *N. vectensis Syt *genes *in vivo*, will be of great interest to further understand the evolutionary development of animal nervous systems. Initial study of the molecular anatomy of the *N. vectensis *nervous system has examined the expression patterns of neurotransmitters, neuropeptides, transcription factor and cell adhesion genes [118]. Synaptotagmin gene expression was used to label differentiated neurons in *Platynereis*, in a study of the evolutionary development of the central nervous system of animals [119]. *Capitella*, like *Platynereis*, is an annelid member of the Lophotrochozoa, but has the advantage as a model organism, of a whole genome sequence [120]. Study of neurogenesis in *Capitella *is now underway [121]. *In situ *hybridization analysis of *Syt1 *expression in *Capitella*, shows a broad neural pattern (Elaine Seaver, personal communication).

It will be important in future, to describe and compare anatomical expression patterns and functions of *Syt *genes, both conserved and unique, in vertebrates and invertebrates. Such comparisons will be essential to improve, or reduce confidence in functional annotation of genomes based on phylogenetic homology, or similarity otherwise defined. Good annotation of genomes is a necessary step in the process of biocuration [122] a new field aimed at using twenty first century sequencing and information technology to make accessible to bench scientists, the vast amount of information they require, distilled, in order to take forward wisely informed hypothesis driven research. Community deposition of a variety of types of biological data into appropriate databases, database efforts to curate, update and integrate this data with relevant data in other databases, will if pursued successfully, lead to great progress in biology in the years ahead. For the time being, the manual gene collection made available here, can be used immediately, by bench scientists engaged in hypothesis driven investigation of membrane trafficking by these proteins, to widen the bounds of a field already stretching from biophysical study *in vitro *at the atomic level, to phenotypic assessment of gene function in model organisms.

## Authors' contributions

The author had sole responsibility for the design and execution of this work.

## Supplementary Material

Additional file 1 **Comprehensive information about the marine invertebrate genes in this collection**. This spreadsheet file (MarineInvertebrates.xls) contains full details of each gene identified in the marine invertebrate genomes in this collection. Click here for file

Additional file 2 **Comprehensive information about the ecdysozoan genes in this collection**. This spreadsheet file (Ecdysozoa.xls) contains full details of each gene identified in the ecdysozoan genomes in this collection. Click here for file

Additional file 3 **Comprehensive information about the vertebrate genes in this collection**. This spreadsheet file (Vertebrates.xls) contains full details of each gene identified in the vertebrate genomes in this collection. Click here for file

Additional file 4 **Comprehensive information about the SNARE and complexin genes identified in *T. adhaerens *and *N. vectensis***. This spreadsheet file (SNAREs.xls) contains full details of the genes identified in *T. adhaerens *and *N. vectensis *which are similar to neuronal SNAREs and complexin. Click here for file

Additional file 5 **Dendrogram of relationships among the invertebrate sequences in this collection**. Additional file 5 displays the guide tree of the clustalw2 comparison of the invertebrate sequences in this collection, excluding variants, totalling 356 sequences. Genes which encode mutually exclusive alternative exons are highlighted with a green box. Conserved groups of *Syt *genes which have not previously been described, are highlighted with a blue box. Click here for file

Additional file 6 **Dendrogram of relationships among the vertebrate sequences in this collection**. Additional file 6 displays the guide tree of the clustalw2 comparison of the vertebrate sequences in this collection, excluding variants, totalling 355 sequences. Mouse genes are highlighted with a red box. Conserved groups of *Syt *genes which have not previously been described, are highlighted with a blue box. Click here for file

Additional file 7 **Dendrogram of relationships among the invertebrate and mammalian sequences in this collection**. Additional file 7 displays the guide tree of the clustalw2 comparison of the invertebrate sequences (one representative per genus) excluding variants, plus mammalian sequences, excluding variants, totalling 289 sequences. Mouse genes are highlighted with a red box. Conserved groups of *Syt *genes which have not previously been described, are highlighted with a blue box. Click here for file

Additional file 8 **Dendrogram of relationships among the invertebrate and vertebrate sequences in this collection**. Additional file 8 displays the guide tree of the clustalw2 comparison of the invertebrate sequences (one representative per genus) excluding variants, plus a subset of vertebrate sequences, excluding variants, totalling 454 sequences. Mouse genes are highlighted with a red box. Conserved groups of *Syt *genes which have not previously been described, are highlighted with a blue box. Click here for file

Additional file 9 **Alignment of the invertebrate Syt1 sequences, plus *Caenorhabditis *snt-3**. Amino acid position is marked every hundred amino acids approximately, at the top of each page of the alignment. Splice variants are included and highlighted with black dots where they differ. Intron position and phase is indicated with a coloured bar between amino acids. Black bars indicate phase 0 introns. Red bars indicate phase +1 introns. Blue bars indicate phase +2 introns. The five conserved acidic amino acids in each C2 domain are indicated by black arrows at the top of the alignment. X residues indicate where a portion of sequence is missing. Click here for file

Additional file 10 **Alignment of the vertebrate Syt1 sequences**. Amino acid position is marked every hundred amino acids approximately, at the top of each page of the alignment. Splice variants are included and highlighted with black dots where they differ. Intron position and phase is indicated with a coloured bar between amino acids. Black bars indicate phase 0 introns. Red bars indicate phase +1 introns. The five conserved acidic amino acids in each C2 domain are indicated by black arrows at the top of the alignment. Conserved N-glycosylation consensus sites are indicated by blue boxes. The conserved threonine, which can be O-glycosylated is also indicated by a blue box. X residues indicate where a portion of sequence is missing. Click here for file

Additional file 11 **Alignment of the vertebrate Syt2 sequences**. Amino acid position is marked every hundred amino acids approximately, at the top of each page of the alignment. The *X. tropicalis *splice variant is included and highlighted with a black dot where it differs. Intron position and phase is indicated with a coloured bar between amino acids. Black bars indicate phase 0 introns. Red bars indicate phase +1 introns. The five conserved acidic amino acids in each C2 domain are indicated by black arrows at the top of the alignment. X residues indicate where a portion of sequence is missing. Click here for file

Additional file 12 **Alignment of the vertebrate Syt5 sequences**. Amino acid position is marked every hundred amino acids approximately, at the top of each page of the alignment. Splice variants are included and highlighted with black dots where they differ. Intron position and phase is indicated with a coloured bar between amino acids. Black bars indicate phase 0 introns. Red bars indicate phase +1 introns. The five conserved acidic amino acids in each C2 domain are indicated by black arrows at the top of the alignment. X residues indicate where a portion of sequence is missing. Click here for file

Additional file 13 **Alignment of the vertebrate Syt8 sequences**. Amino acid position is marked every hundred amino acids approximately, at the top of each page of the alignment. Splice variants are included and highlighted with black dots where they differ. Intron position and phase is indicated with a coloured bar between amino acids. Black bars indicate phase 0 introns. Red bars indicate phase +1 introns. X residues indicate where a portion of sequence is missing. Click here for file

Additional file 14 **Alignment of the invertebrate Syt4 sequences**. Amino acid position is marked every hundred amino acids approximately, at the top of each page of the alignment. Splice variants are included and highlighted with black dots where they differ. Intron position and phase is indicated with a coloured bar between amino acids. Black bars indicate phase 0 introns. Red bars indicate phase +1 introns. Blue bars indicate phase +2 introns. X residues indicate where a portion of sequence is missing. Click here for file

Additional file 15 **Alignment of the vertebrate Syt4 sequences**. Amino acid position is marked every hundred amino acids approximately, at the top of each page of the alignment. The *G. gallus *splice variant is included and highlighted with a black dot where it differs. Intron position and phase is indicated with a coloured bar between amino acids. Black bars indicate phase 0 introns. Red bars indicate phase +1 introns. X residues indicate where a portion of sequence is missing. Click here for file

Additional file 16 **Alignment of the vertebrate Syt11 sequences**. Amino acid position is marked every hundred amino acids approximately, at the top of each page of the alignment. Splice variants are included and highlighted with black dots where they differ. Intron position and phase is indicated with a coloured bar between amino acids. Black bars indicate phase 0 introns. Red bars indicate phase +1 introns. X residues indicate where a portion of sequence is missing. Click here for file

Additional file 17 **Alignment of the invertebrate Syt7 sequences**. Amino acid position is marked every hundred amino acids approximately, at the top of each page of the alignment. The *D. melanogaster *splice variant is included and highlighted with a black dot where it differs. Intron position and phase is indicated with a coloured bar between amino acids. Black bars indicate phase 0 introns. Red bars indicate phase +1 introns. Blue bars indicate phase +2 introns. The five conserved acidic amino acids in each C2 domain are indicated by black arrows at the top of the alignment. X residues indicate where a portion of sequence is missing. Click here for file

Additional file 18 **Alignment of the vertebrate Syt7 sequences**. Amino acid position is marked every hundred amino acids approximately, at the top of each page of the alignment. Splice variants are included and highlighted with black dots where they differ. Intron position and phase is indicated with a coloured bar between amino acids. Black bars indicate phase 0 introns. Red bars indicate phase +1 introns. Blue bars indicate phase +2 introns. The five conserved acidic amino acids in each C2 domain are indicated by black arrows at the top of the alignment. X residues indicate where a portion of sequence is missing. Click here for file

Additional file 19 **Alignment of the invertebrate Syt9 sequences**. Amino acid position is marked every hundred amino acids approximately, at the top of each page of the alignment. Intron position and phase is indicated with a coloured bar between amino acids. Black bars indicate phase 0 introns. Red bars indicate phase +1 introns. Blue bars indicate phase +2 introns. The widely conserved motif of unknown function, just upstream of the C2A domain, is indicated. The five conserved acidic amino acids in each C2 domain are indicated by black arrows at the top of the alignment. X residues indicate where a portion of sequence is missing. Click here for file

Additional file 20 **Alignment of the vertebrate Syt9 sequences**. Amino acid position is marked every hundred amino acids approximately, at the top of each page of the alignment. Splice variants are included and highlighted with black dots where they differ. Intron position and phase is indicated with a coloured bar between amino acids. Black bars indicate phase 0 introns. Red bars indicate phase +1 introns. Blue bars indicate phase +2 introns. The widely conserved motif of unknown function, just upstream of the C2A domain, is indicated. The five conserved acidic amino acids in each C2 domain are indicated by black arrows at the top of the alignment. X residues indicate where a portion of sequence is missing. Click here for file

Additional file 21 **Alignment of the vertebrate Syt3 sequences**. Amino acid position is marked every hundred amino acids approximately, at the top of each page of the alignment. Intron position and phase is indicated with a coloured bar between amino acids. Black bars indicate phase 0 introns. Red bars indicate phase +1 introns. Blue bars indicate phase +2 introns. The widely conserved motif of unknown function, just upstream of the C2A domain, is indicated. The five conserved acidic amino acids in each C2 domain are indicated by black arrows at the top of the alignment. X residues indicate where a portion of sequence is missing. Click here for file

Additional file 22 **Alignment of the vertebrate Syt6 sequences**. Amino acid position is marked every hundred amino acids approximately, at the top of each page of the alignment. Splice variants are included and highlighted with black dots where they differ. Intron position and phase is indicated with a coloured bar between amino acids. Black bars indicate phase 0 introns. Red bars indicate phase +1 introns. Blue bars indicate phase +2 introns. The widely conserved motif of unknown function, just upstream of the C2A domain, is indicated. The five conserved acidic amino acids in each C2 domain are indicated by black arrows at the top of the alignment. X residues indicate where a portion of sequence is missing. Click here for file

Additional file 23 **Alignment of the vertebrate Syt10 sequences**. Amino acid position is marked every hundred amino acids approximately, at the top of each page of the alignment. Intron position and phase is indicated with a coloured bar between amino acids. Black bars indicate phase 0 introns. Red bars indicate phase +1 introns. Blue bars indicate phase +2 introns. The widely conserved motif of unknown function, just upstream of the C2A domain, is indicated. The five conserved acidic amino acids in each C2 domain are indicated by black arrows at the top of the alignment. Click here for file

Additional file 24 **Alignment of the invertebrate Syt12 sequences**. Amino acid position is marked every hundred amino acids approximately, at the top of each page of the alignment. Intron position and phase is indicated with a coloured bar between amino acids. Black bars indicate phase 0 introns. Red bars indicate phase +1 introns. Blue bars indicate phase +2 introns. Because of their differing positions, TM domains are highlighted in blue. X residues indicate where a portion of sequence is missing. Click here for file

Additional file 25 **Alignment of the vertebrate Syt12 sequences**. Amino acid position is marked every hundred amino acids approximately, at the top of each page of the alignment. The *H. sapiens *splice variant is included and highlighted with a black dot where it differs. Intron position and phase is indicated with a coloured bar between amino acids. Black bars indicate phase 0 introns. Red bars indicate phase +1 introns. X residues indicate where a portion of sequence is missing. Click here for file

Additional file 26 **Alignment of the invertebrate Syt13 sequences**. Amino acid position is marked every hundred amino acids approximately, at the top of each page of the alignment. Splice variants which specify an alternative N-terminus lacking a TM domain, are indicated on top, with a dotted line indicating where they join the common C-terminal portion. Intron position and phase is indicated with a coloured bar between amino acids. Black bars indicate phase 0 introns. Red bars indicate phase +1 introns. Blue bars indicate phase +2 introns. The widely conserved motif of unknown function, upstream of the C2A domain, is indicated. X residues indicate where a portion of sequence is missing. Click here for file

Additional file 27 **Alignment of the vertebrate Syt13 sequences**. Amino acid position is marked every hundred amino acids approximately, at the top of each page of the alignment. The *H. sapiens *splice variant is included and highlighted with a black dot where it differs. Intron position and phase is indicated with a coloured bar between amino acids. Black bars indicate phase 0 introns. Red bars indicate phase +1 introns. The widely conserved motif of unknown function, just upstream of the C2A domain, is indicated. X residues indicate where a portion of sequence is missing. Click here for file

Additional file 28 **Alignment of the invertebrate Syt15 sequences**. Amino acid position is marked every hundred amino acids approximately, at the top of each page of the alignment. Intron position and phase is indicated with a coloured bar between amino acids. Black bars indicate phase 0 introns. Red bars indicate phase +1 introns. Blue bars indicate phase +2 introns. Because of their differing positions, TM domains are highlighted in blue. The widely conserved motif of unknown function, just upstream of the C2A domain, is indicated. X residues indicate where a portion of sequence is missing. Click here for file

Additional file 29 **Alignment of the vertebrate Syt15 sequences**. Amino acid position is marked every hundred amino acids approximately, at the top of each page of the alignment. Splice variants are included and highlighted with black dots where they differ. Intron position and phase is indicated with a coloured bar between amino acids. Black bars indicate phase 0 introns. Red bars indicate phase +1 introns. Blue bars indicate phase +2 introns. The widely conserved motif of unknown function, just upstream of the C2A domain, is indicated. X residues indicate where a portion of sequence is missing. Click here for file

Additional file 30 **Alignment of the invertebrate Syt16 sequences**. Amino acid position is marked every hundred amino acids approximately, at the top of each page of the alignment. Splice variants which specify alternative N-termini lacking TM domains, are included and highlighted with black dots where their sequences differ. TM domains are highlighted in blue. Intron position and phase is indicated with a coloured bar between amino acids. Black bars indicate phase 0 introns. Red bars indicate phase +1 introns. Blue bars indicate phase +2 introns. X residues indicate where a portion of sequence is missing. Click here for file

Additional file 31 **Alignment of the vertebrate Syt16 sequences which have TM domains**. Amino acid position is marked every hundred amino acids approximately, at the top of each page of the alignment. Splice variants are included and highlighted with black dots where they differ. Intron position and phase is indicated with a coloured bar between amino acids. Black bars indicate phase 0 introns. Red bars indicate phase +1 introns. X residues indicate where a portion of sequence is missing. Click here for file

Additional file 32 **Alignment of the vertebrate Syt16 sequences which lack TM domains**. Amino acid position is marked every hundred amino acids approximately, at the top of each page of the alignment. Splice variants are included and highlighted with black dots where they differ. Intron position and phase is indicated with a coloured bar between amino acids. Black bars indicate phase 0 introns. Red bars indicate phase +1 introns. X residues indicate where a portion of sequence is missing. Click here for file

Additional file 33 **Alignment of the vertebrate Syt14 sequences**. Amino acid position is marked every hundred amino acids approximately, at the top of each page of the alignment. Splice variants are included and highlighted with black dots where they differ. Intron position and phase is indicated with a coloured bar between amino acids. Black bars indicate phase 0 introns. Red bars indicate phase +1 introns. X residues indicate where a portion of sequence is missing. Click here for file

Additional file 34 **Alignment of the invertebrate Syt17 sequences**. Amino acid position is marked every hundred amino acids approximately, at the top of each page of the alignment. Intron position and phase is indicated with a coloured bar between amino acids. Black bars indicate phase 0 introns. Red bars indicate phase +1 introns. Blue bars indicate phase +2 introns. The widely conserved motif of unknown function, upstream of the C2A domain, is indicated. X residues indicate where a portion of sequence is missing. Click here for file

Additional file 35 **Alignment of the vertebrate Syt17 sequences**. Amino acid position is marked every hundred amino acids approximately, at the top of each page of the alignment. Splice variants are included and highlighted with black dots where they differ. Intron position and phase is indicated with a coloured bar between amino acids. Black bars indicate phase 0 introns. Red bars indicate phase +1 introns. Blue bars indicate phase +2 introns. A possible motif just upstream of the C2A domain, is indicated. X residues indicate where a portion of sequence is missing. Click here for file

Additional file 36 **Alignment of the invertebrate Sytalpha sequences**. Amino acid position is marked every hundred amino acids approximately, at the top of each page of the alignment. Intron position and phase is indicated with a coloured bar between amino acids. Black bars indicate phase 0 introns. Red bars indicate phase +1 introns. Blue bars indicate phase +2 introns. The widely conserved motif of unknown function, just upstream of the C2A domain, is indicated. X residues indicate where a portion of sequence is missing. Click here for file

Additional file 37 **Alignment of the Syt18 sequences**. Amino acid position is marked every hundred amino acids approximately, at the top of each page of the alignment. Intron position and phase is indicated with a coloured bar between amino acids. Black bars indicate phase 0 introns. Red bars indicate phase +1 introns. X residues indicate where a portion of sequence is missing. Click here for file

Additional file 38 **Alignment of the vertebrate Syt19 sequences**. Amino acid position is marked every hundred amino acids approximately, at the top of each page of the alignment. Intron position and phase is indicated with a coloured bar between amino acids. Black bars indicate phase 0 introns. Red bars indicate phase +1 introns. The X residue indicates where a portion of sequence is missing. Click here for file

Additional file 39 **Alignment of the invertebrate Syt21 sequences**. Amino acid position is marked every hundred amino acids approximately, at the top of each page of the alignment. TM sequences are highlighted in blue. Intron position and phase is indicated with a coloured bar between amino acids. Black bars indicate phase 0 introns. Red bars indicate phase +1 introns. X residues indicate where a portion of sequence is missing. Click here for file

Additional file 40 **Alignment of the *N. vectensis *Syt sequences**. Amino acid position is marked every hundred amino acids approximately, at the top of each page of the alignment. Splice variants are included and highlighted with black dots where they differ. TM sequences are highlighted in blue. Intron position and phase is indicated with a coloured bar between amino acids. Black bars indicate phase 0 introns. Red bars indicate phase +1 introns. Blue bars indicate phase +2 introns. X residues indicate where a portion of sequence is missing. Click here for file

Additional file 41 **Alignment of the invertebrate Dblc2 sequences**. Amino acid position is marked every hundred amino acids approximately, at the top of each page of the alignment. Splice variants are included and highlighted with black dots where they differ. Intron position and phase is indicated with a coloured bar between amino acids. Black bars indicate phase 0 introns. Red bars indicate phase +1 introns. Blue bars indicate phase +2 introns. Click here for file

Additional file 42 **Alignment of the invertebrate Esyt2 sequences**. Amino acid position is marked every hundred amino acids approximately, at the top of each page of the alignment. Splice variants are included and highlighted with black dots where they differ. Intron position and phase is indicated with a coloured bar between amino acids. Black bars indicate phase 0 introns. Red bars indicate phase +1 introns. Blue bars indicate phase +2 introns. X residues indicate where a portion of sequence is missing. Click here for file

Additional file 43 **Alignment of the mutually exclusive alternative Esyt2 exons**. In addition to the alternatively coded exon which is highlighted, the exon upstream and the exon downstream are shown. Intron position and phase is indicated with a coloured bar between amino acids. Black bars indicate phase 0 introns. Blue bars indicate phase +2 introns. Click here for file

Additional file 44 **Alignment of the vertebrate Esyt1 sequences**. Amino acid position is marked every hundred amino acids approximately, at the top of each page of the alignment. Splice variants are included and highlighted with black dots where they differ. The middle portion is only present in the fish esyt1b sequences. A pink dot in this portion, marks an intron loss in *T. rubripes *and *T. nigroviridis*. Intron position and phase is indicated with a coloured bar between amino acids. Black bars indicate phase 0 introns. Blue bars indicate phase +2 introns. X residues indicate where a portion of sequence is missing. Click here for file

Additional file 45 **Alignment of the vertebrate Esyt2 sequences**. Amino acid position is marked every hundred amino acids approximately, at the top of each page of the alignment. Splice variants are included and highlighted with black dots where they differ. Intron position and phase is indicated with a coloured bar between amino acids. Black bars indicate phase 0 introns. Blue bars indicate phase +2 introns. X residues indicate where a portion of sequence is missing. Click here for file

Additional file 46 **Alignment of the vertebrate Esyt3 sequences**. Amino acid position is marked every hundred amino acids approximately, at the top of each page of the alignment. Splice variants are included and highlighted with black dots where they differ. Intron position and phase is indicated with a coloured bar between amino acids. Black bars indicate phase 0 introns. Red bars indicate phase +1 introns. Blue bars indicate phase +2 introns. Some intron positions are marked with dotted lines. In these cases, transcript sequence covers a gap in the genomic sequence and the intron presence is assumed. X residues indicate where a portion of sequence is missing. Click here for file

Additional file 47 **Alignment of the invertebrate Rabphilin sequences**. Amino acid position is marked every hundred amino acids approximately, at the top of each page of the alignment. Splice variants are indicated. Intron position and phase is indicated with a coloured bar between amino acids. Black bars indicate phase 0 introns. Red bars indicate phase +1 introns. Blue bars indicate phase +2 introns. The five conserved acidic amino acids in each C2 domain are indicated by pink dots at the top of the alignment. X residues indicate where a portion of sequence is missing. Click here for file

Additional file 48 **Alignment of the vertebrate Rabphilin (Rph3a) sequences**. Amino acid position is marked every hundred amino acids approximately, at the top of each page of the alignment. Splice variants are included and highlighted with black dots where they differ. Intron position and phase is indicated with a coloured bar between amino acids. Black bars indicate phase 0 introns. Red bars indicate phase +1 introns. Blue bars indicate phase +2 introns. The five conserved acidic amino acids in each C2 domain are indicated by black arrows at the top of the alignment. X residues indicate where a portion of sequence is missing. Click here for file

Additional file 49 **Alignment of the vertebrate Rph3al sequences**. Amino acid position is marked every hundred amino acids approximately, at the top of each page of the alignment. Splice variants are included and highlighted with black dots where they differ. Intron position and phase is indicated with a coloured bar between amino acids. Black bars indicate phase 0 introns. Red bars indicate phase +1 introns. Blue bars indicate phase +2 introns. X residues indicate where a portion of sequence is missing. Click here for file

Additional file 50 **Alignment of the vertebrate Doc2 sequences**. Amino acid position is marked every hundred amino acids approximately, at the top of each page of the alignment. Splice variants are included and highlighted with black dots where they differ. Intron position and phase is indicated with a coloured bar between amino acids. Black bars indicate phase 0 introns. Red bars indicate phase +1 introns. Blue bars indicate phase +2 introns. Some intron positions are marked with dotted lines. In these cases, transcript sequence covers a gap in the genomic sequence and the intron presence is assumed. The five conserved acidic amino acids in each C2 domain are indicated by black arrows at the top of the alignment. X residues indicate where a portion of sequence is missing. Click here for file
