# Supplementary figures and images for: A manual collection of Syt, Esyt, Rph3a, Rph3al, Doc2, and Dblc2 genes from 46 metazoan genomes - an open access resource for neuroscience and evolutionary biology
Source: BMC Genomics. 2010 Jan 15;11:37. doi: 10.1186/1471-2164-11-37 (PMC2823689; doi:10.1186/1471-2164-11-37)

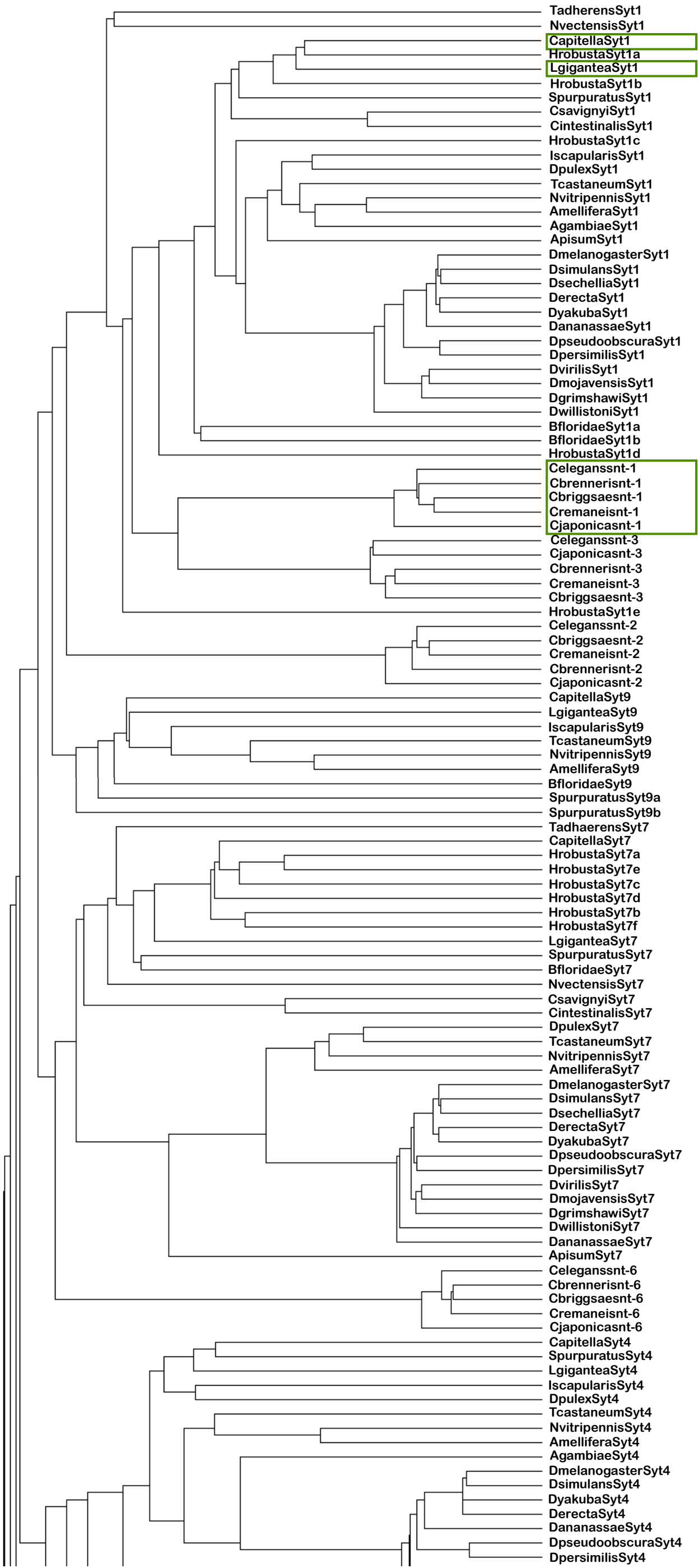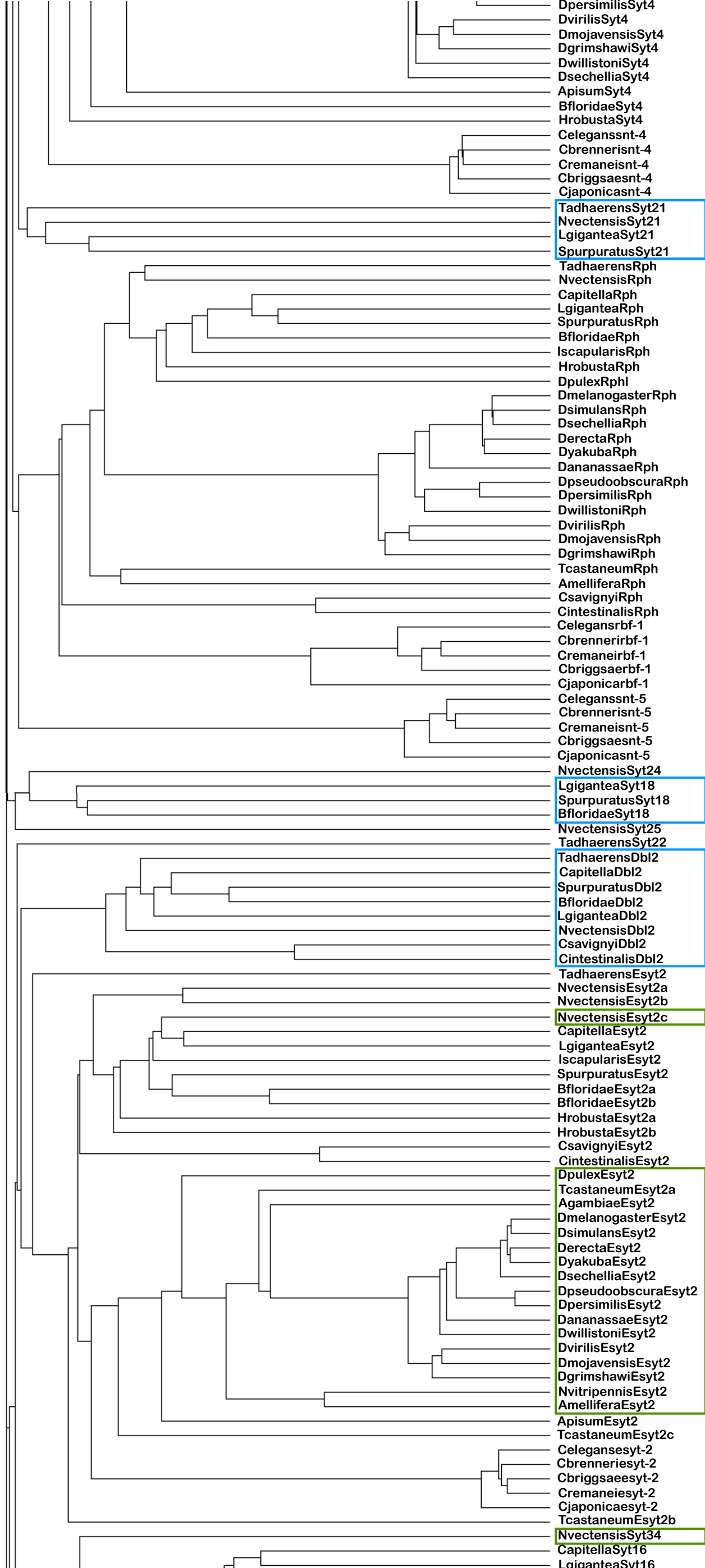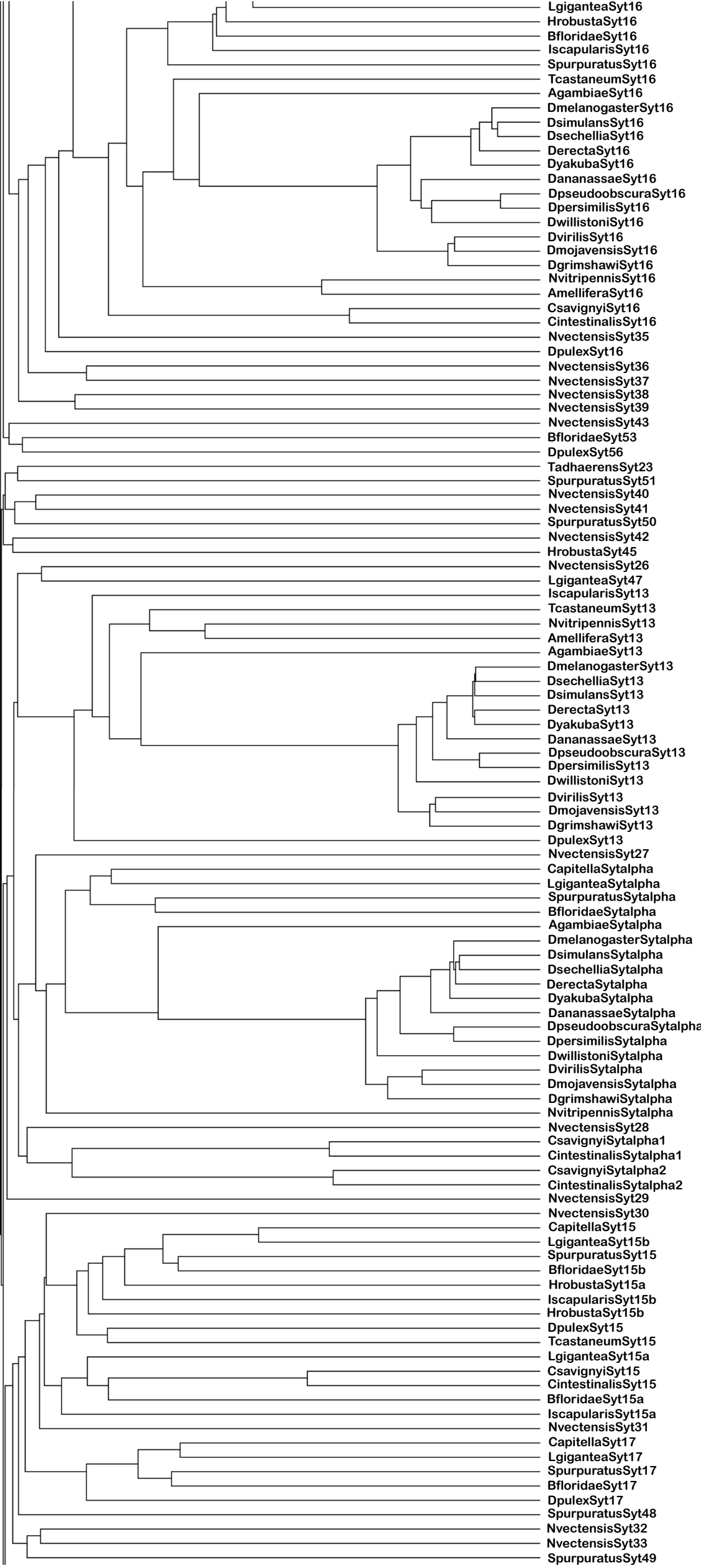

Supplement: Additional file 5 — Dendrogram of relationships among the invertebrate sequences in this collection. Additional file 5 displays the guide tree of the clustalw2 comparison of the invertebrate sequences in this collection, excluding variants, totalling 356 sequences. Genes which encode mutually exclusive alternative exons are highlighted with a green box. Conserved groups of Syt genes which have not previously been described, are highlighted with a blue box. [file 1471-2164-11-37-S5.PDF]

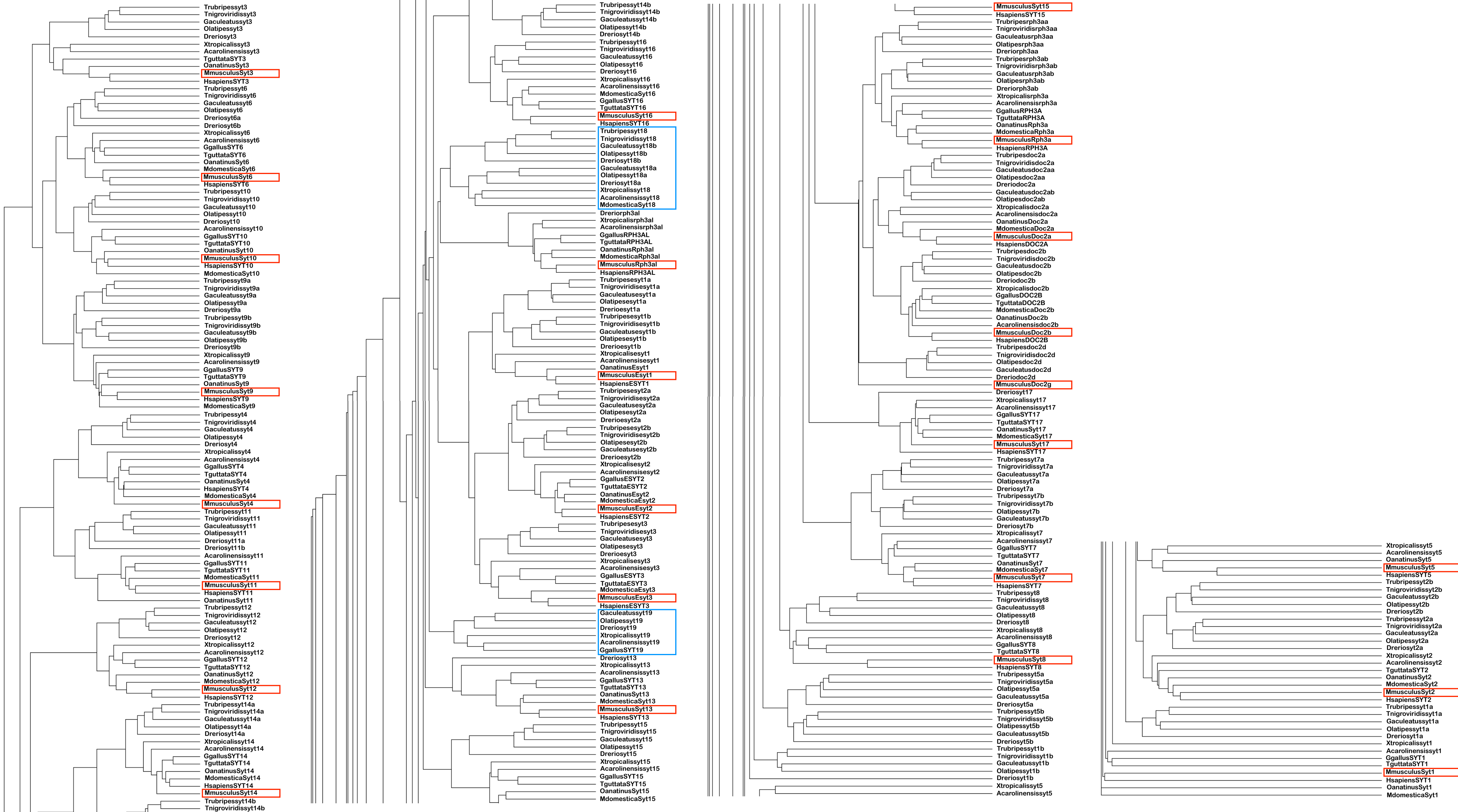

Supplement: Additional file 6 — Dendrogram of relationships among the vertebrate sequences in this collection. Additional file 6 displays the guide tree of the clustalw2 comparison of the vertebrate sequences in this collection, excluding variants, totalling 355 sequences. Mouse genes are highlighted with a red box. Conserved groups of Syt genes which have not previously been described, are highlighted with a blue box. [file 1471-2164-11-37-S6.PDF]

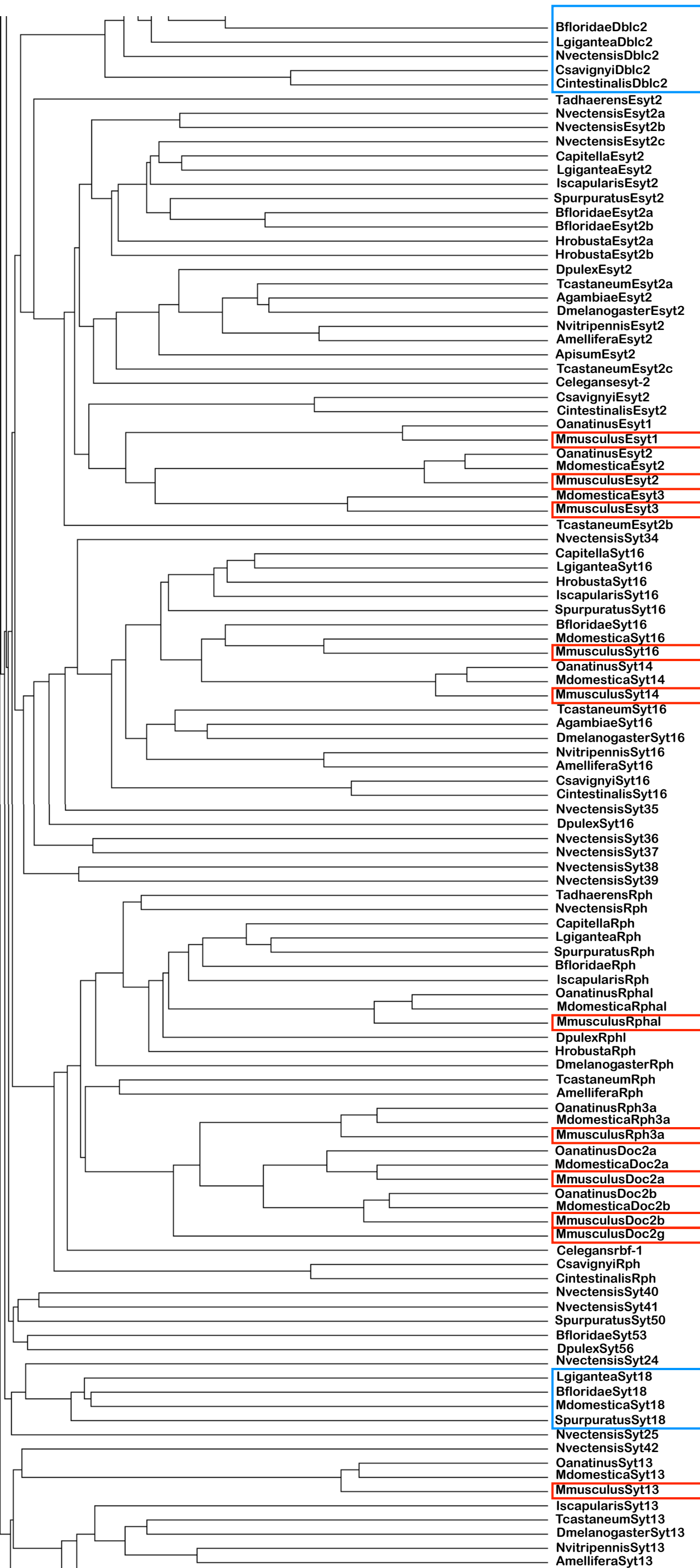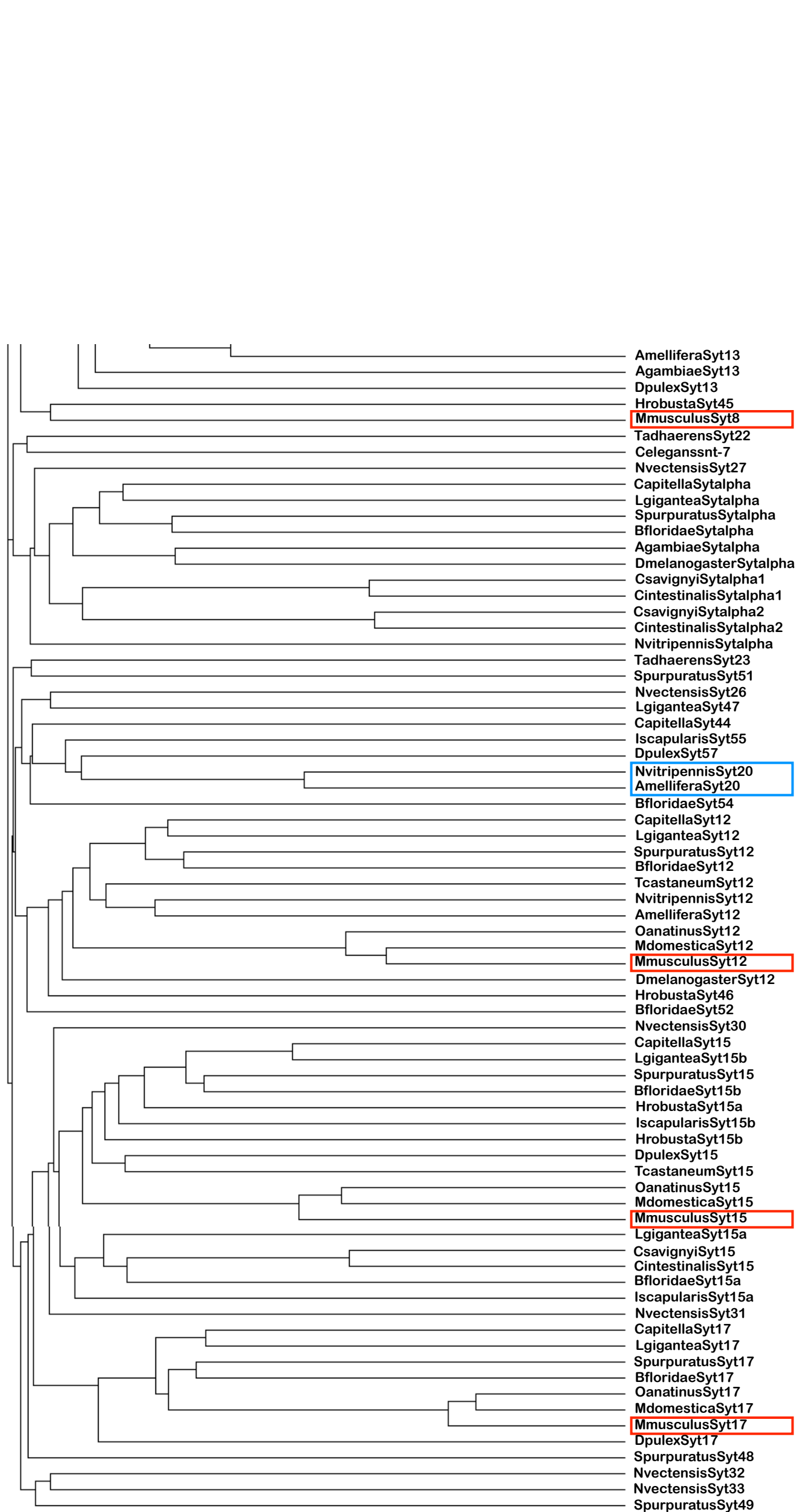

Supplement: Additional file 7 — Dendrogram of relationships among the invertebrate and mammalian sequences in this collection. Additional file 7 displays the guide tree of the clustalw2 comparison of the invertebrate sequences (one representative per genus) excluding variants, plus mammalian sequences, excluding variants, totalling 289 sequences. Mouse genes are highlighted with a red box. Conserved groups of Syt genes which have not previously been described, are highlighted with a blue box. [file 1471-2164-11-37-S7.PDF]

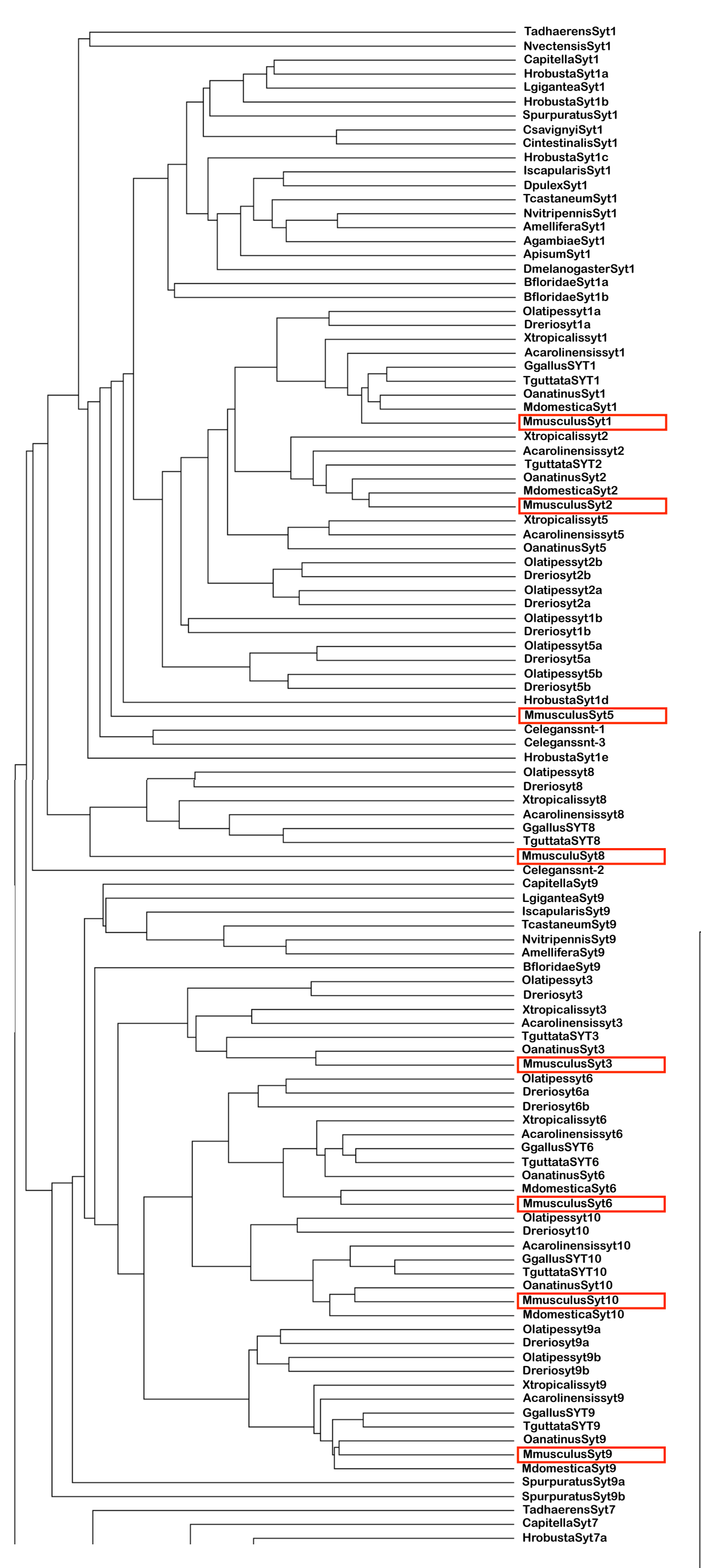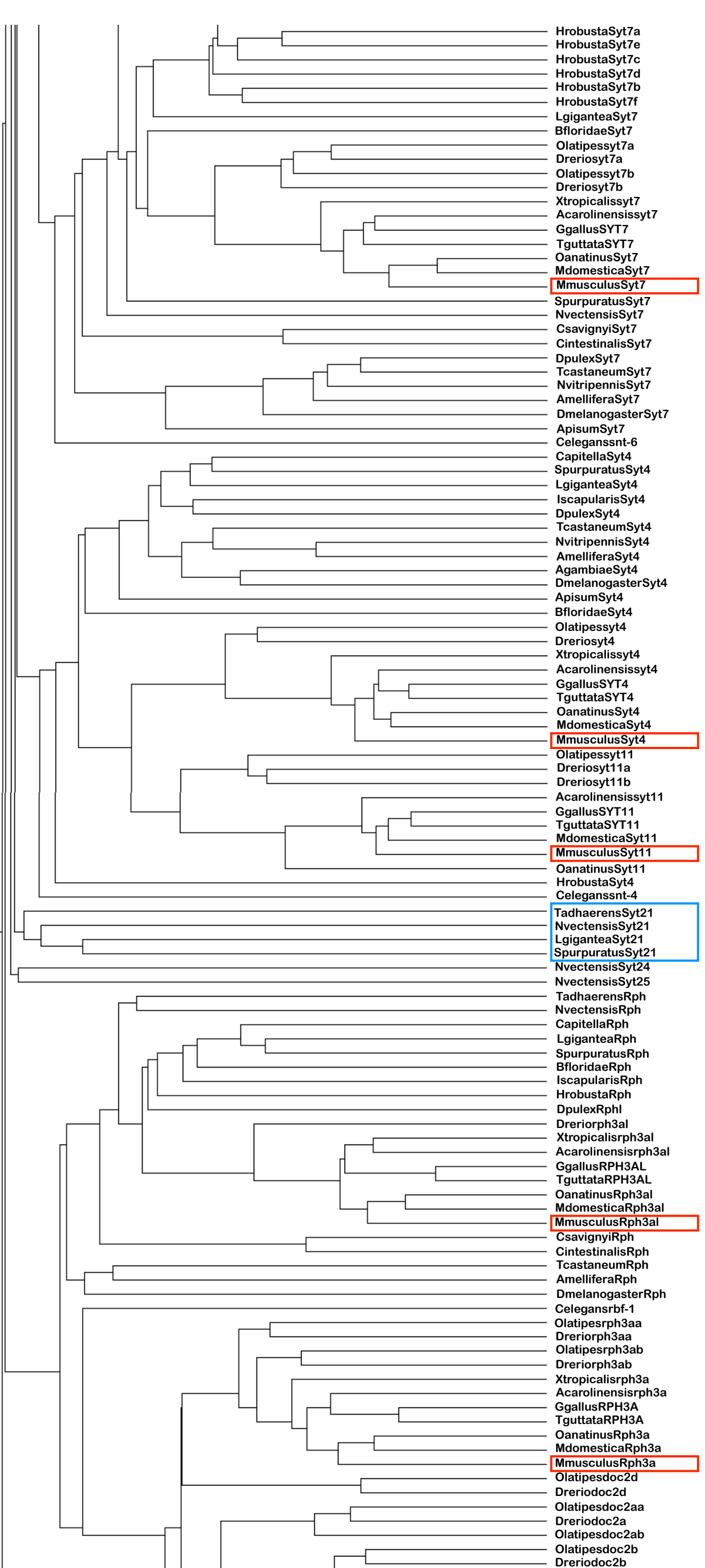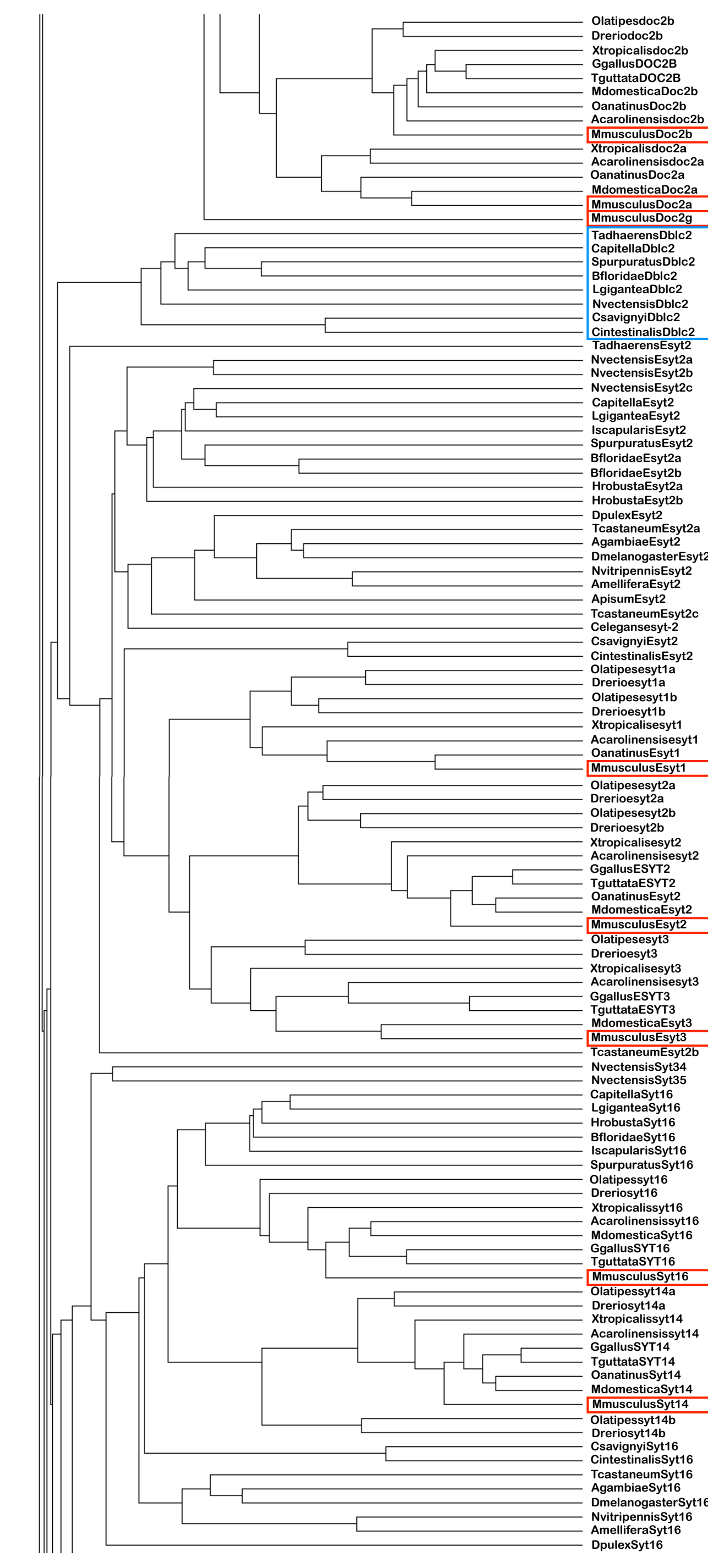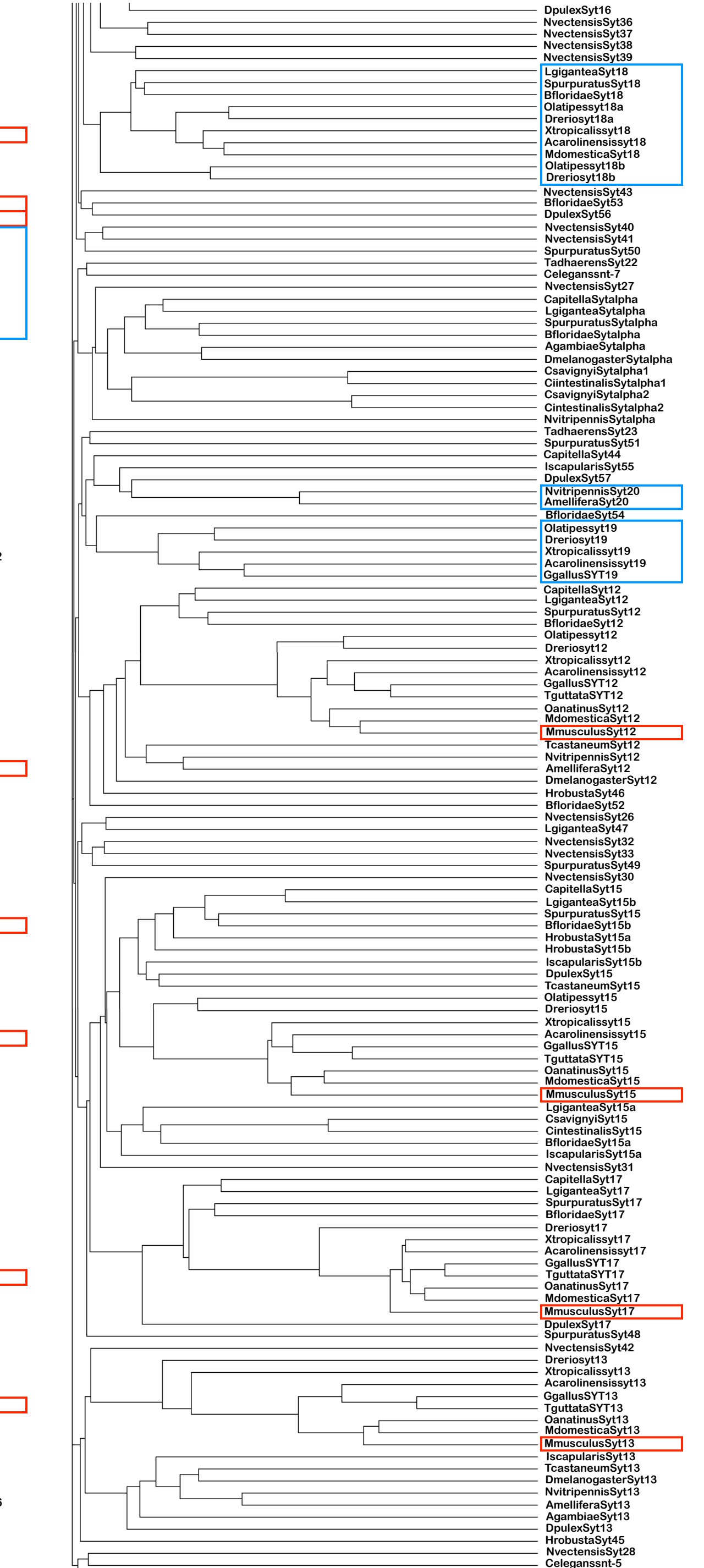

Supplement: Additional file 8 — Dendrogram of relationships among the invertebrate and vertebrate sequences in this collection. Additional file 8 displays the guide tree of the clustalw2 comparison of the invertebrate sequences (one representative per genus) excluding variants, plus a subset of vertebrate sequences, excluding variants, totalling 454 sequences. Mouse genes are highlighted with a red box. Conserved groups of Syt genes which have not previously been described, are highlighted with a blue box. [file 1471-2164-11-37-S8.PDF]

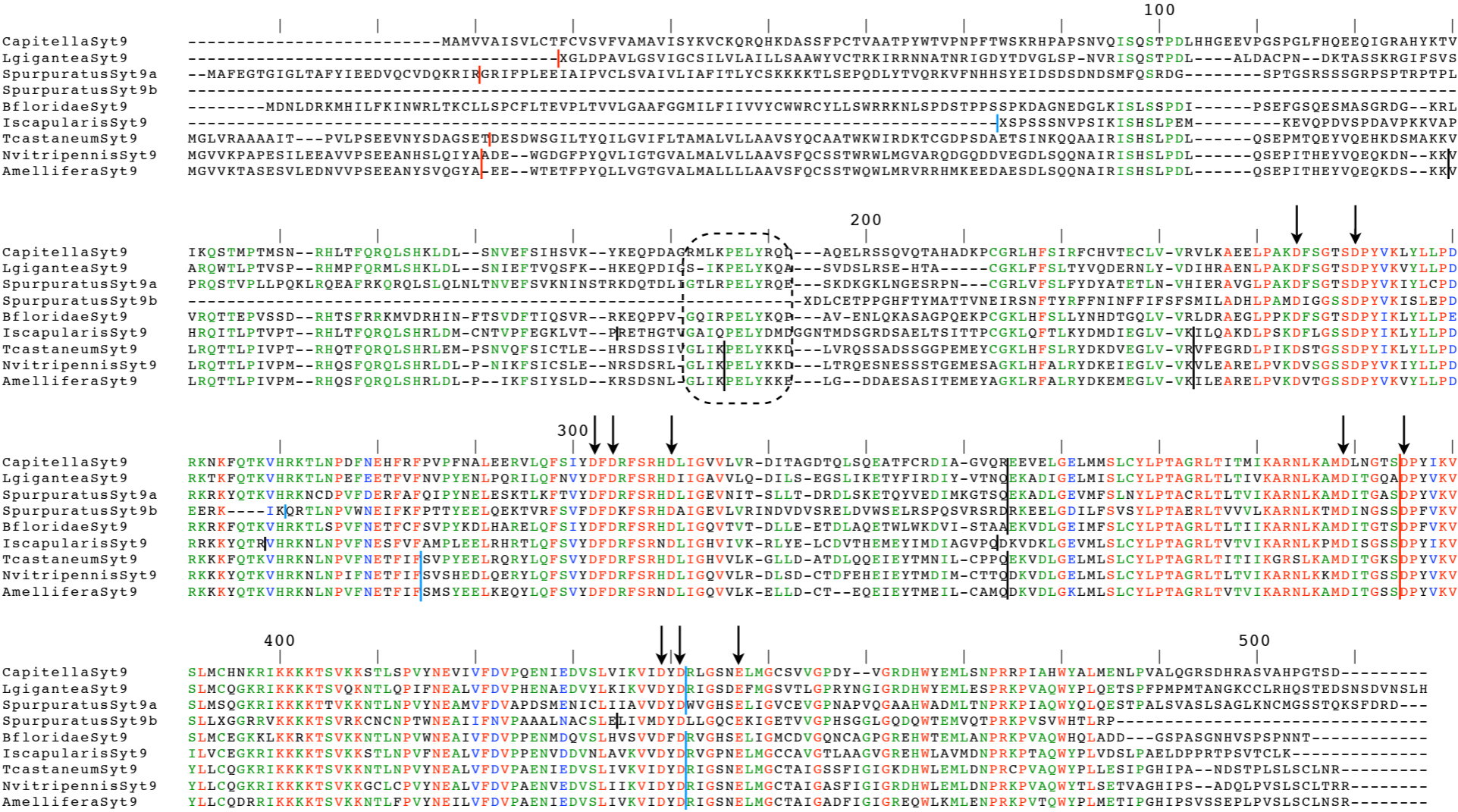

Supplement: Additional file 19 — Alignment of the invertebrate Syt9 sequences. Amino acid position is marked every hundred amino acids approximately, at the top of each page of the alignment. Intron position and phase is indicated with a coloured bar between amino acids. Black bars indicate phase 0 introns. Red bars indicate phase +1 introns. Blue bars indicate phase +2 introns. The widely conserved motif of unknown function, just upstream of the C2A domain, is indicated. The five conserved acidic amino acids in each C2 domain are indicated by black arrows at the top of the alignment. X residues indicate where a portion of sequence is missing. [file 1471-2164-11-37-S19.PDF]

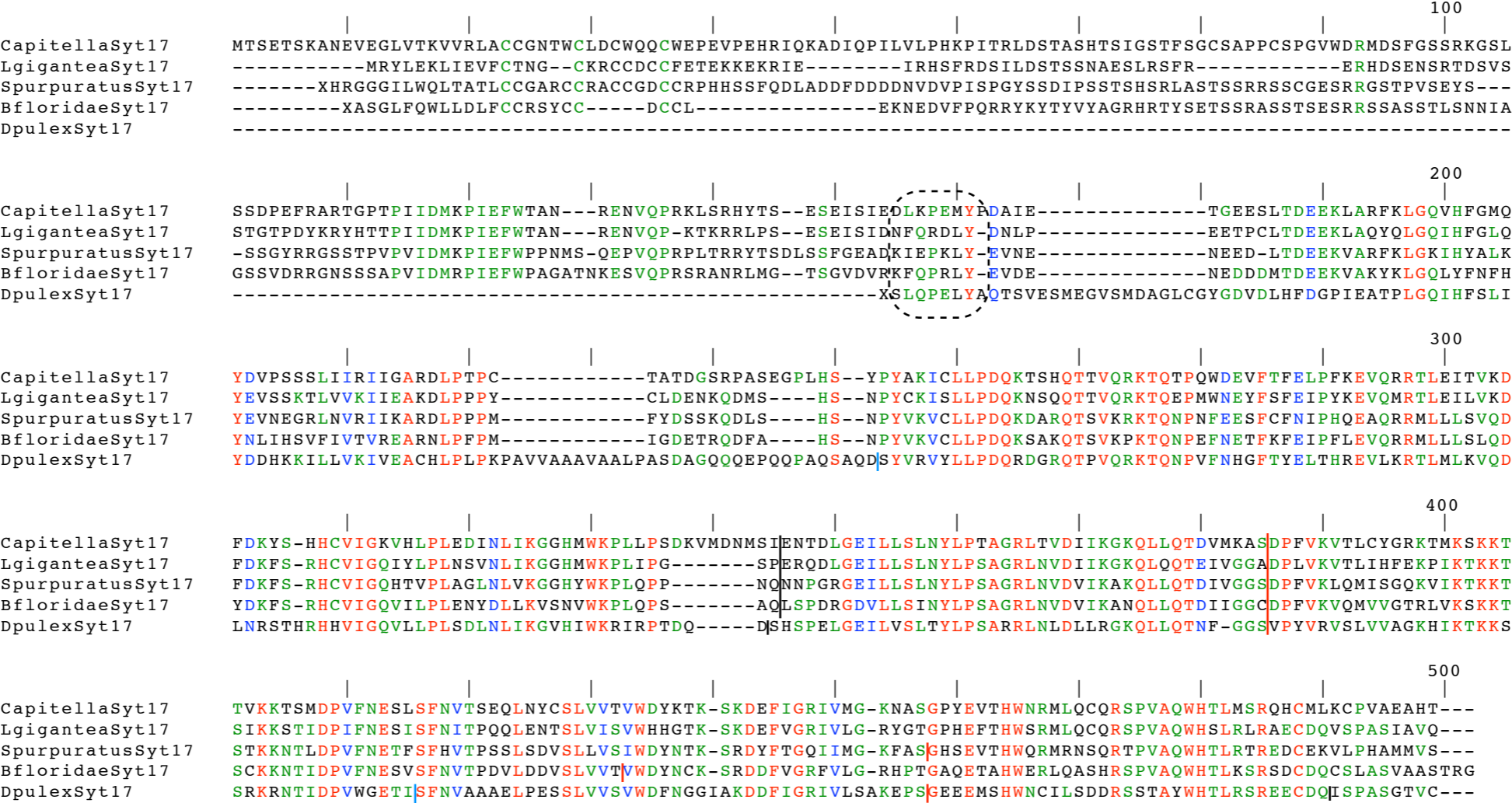

Supplement: Additional file 34 — Alignment of the invertebrate Syt17 sequences. Amino acid position is marked every hundred amino acids approximately, at the top of each page of the alignment. Intron position and phase is indicated with a coloured bar between amino acids. Black bars indicate phase 0 introns. Red bars indicate phase +1 introns. Blue bars indicate phase +2 introns. The widely conserved motif of unknown function, upstream of the C2A domain, is indicated. X residues indicate where a portion of sequence is missing. [file 1471-2164-11-37-S34.PDF]

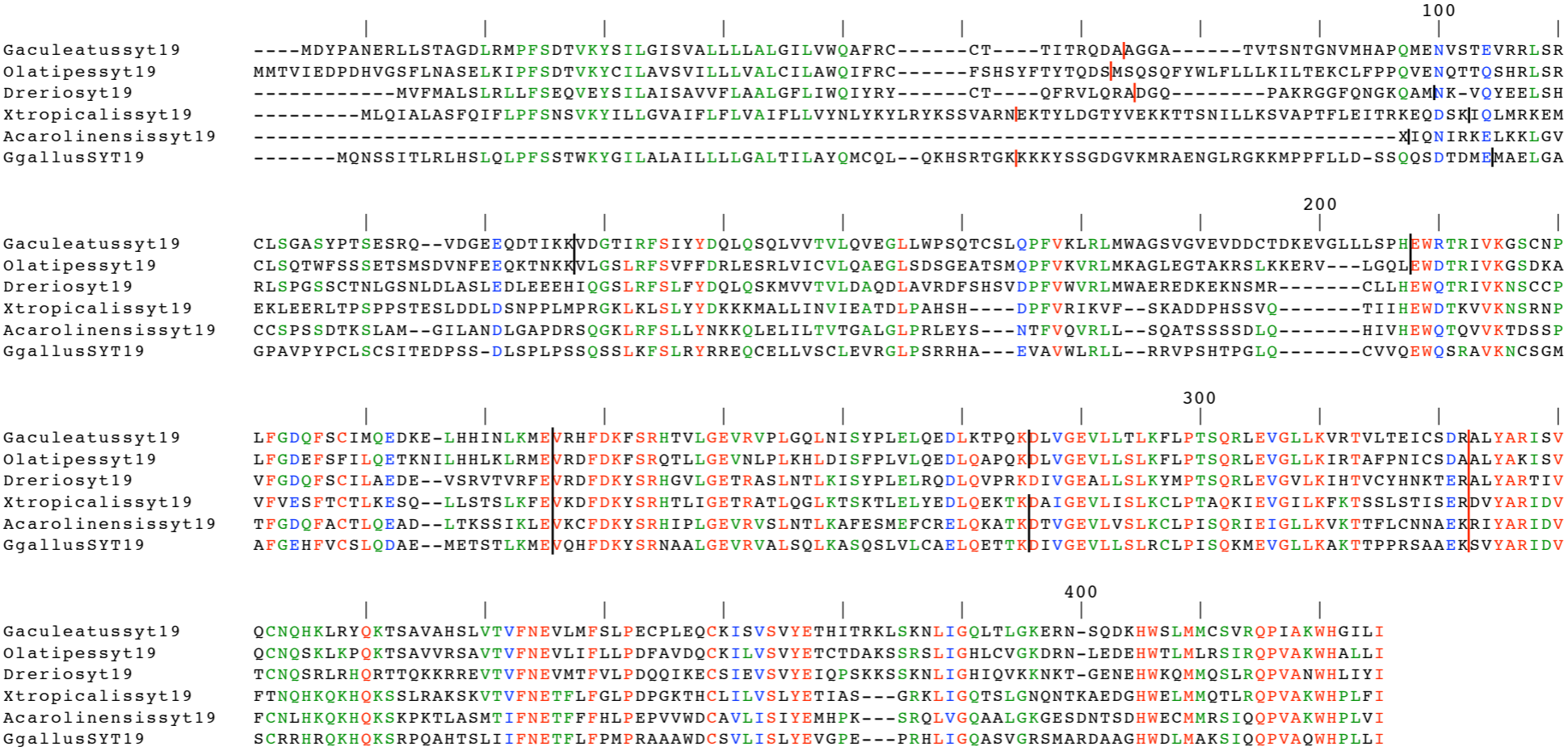

Supplement: Additional file 38 — Alignment of the vertebrate Syt19 sequences. Amino acid position is marked every hundred amino acids approximately, at the top of each page of the alignment. Intron position and phase is indicated with a coloured bar between amino acids. Black bars indicate phase 0 introns. Red bars indicate phase +1 introns. The X residue indicates where a portion of sequence is missing. [file 1471-2164-11-37-S38.PDF]

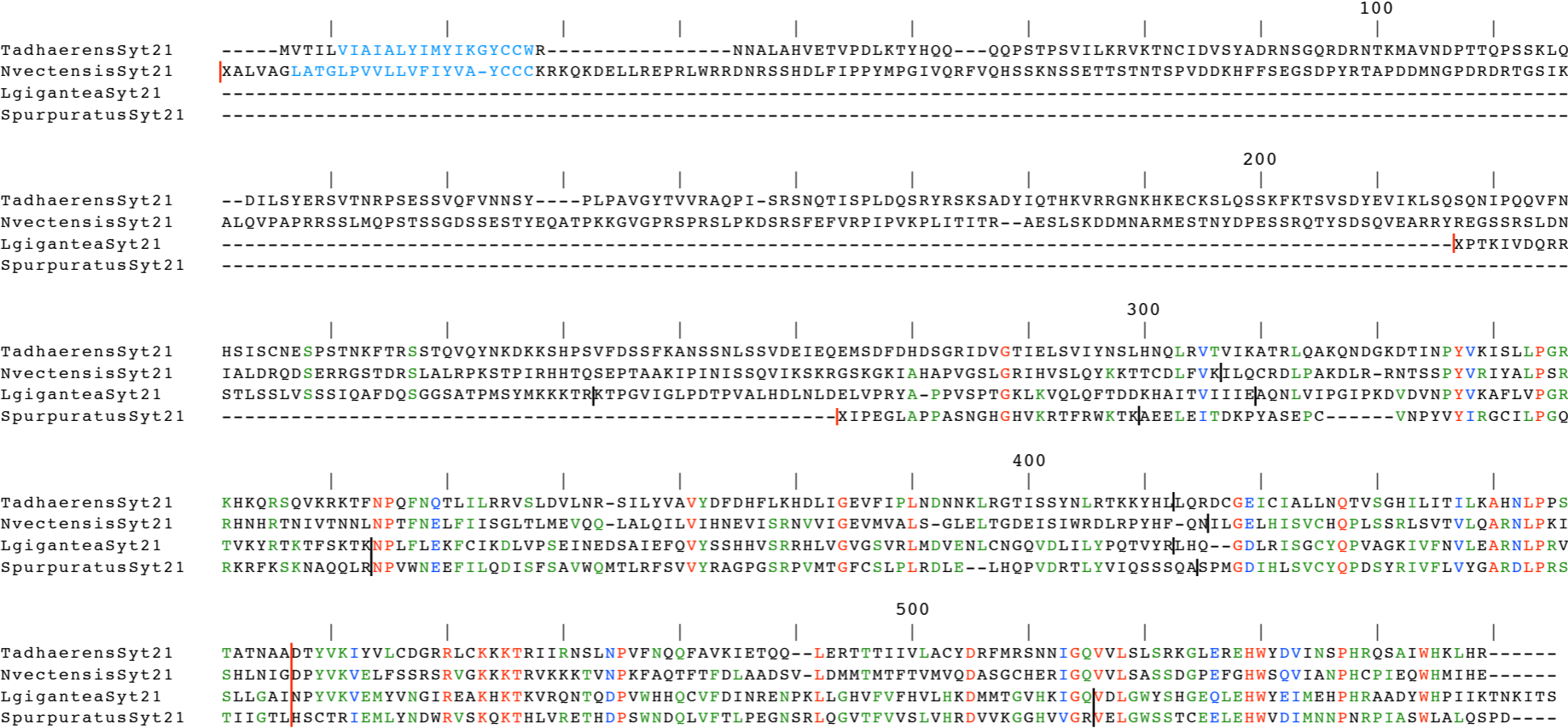

Supplement: Additional file 39 — Alignment of the invertebrate Syt21 sequences. Amino acid position is marked every hundred amino acids approximately, at the top of each page of the alignment. TM sequences are highlighted in blue. Intron position and phase is indicated with a coloured bar between amino acids. Black bars indicate phase 0 introns. Red bars indicate phase +1 introns. X residues indicate where a portion of sequence is missing. [file 1471-2164-11-37-S39.PDF]
