# Supplementary material for: A manual collection of Syt, Esyt, Rph3a, Rph3al, Doc2, and Dblc2 genes from 46 metazoan genomes - an open access resource for neuroscience and evolutionary biology
Source: BMC Genomics. 2010 Jan 15;11:37. doi: 10.1186/1471-2164-11-37 (PMC2823689; doi:10.1186/1471-2164-11-37)
Supplement: Additional file 9 — Alignment of the invertebrate Syt1 sequences, plus Caenorhabditis snt-3. Amino acid position is marked every hundred amino acids approximately, at the top of each page of the alignment. Splice variants are included and highlighted with black dots where they differ. Intron position and phase is indicated with a coloured bar between amino acids. Black bars indicate phase 0 introns. Red bars indicate phase +1 introns. Blue bars indicate phase +2 introns. The five conserved acidic amino acids in each C2 domain are indicated by black arrows at the top of the alignment. X residues indicate where a portion of sequence is missing. [file 1471-2164-11-37-S9.PDF]

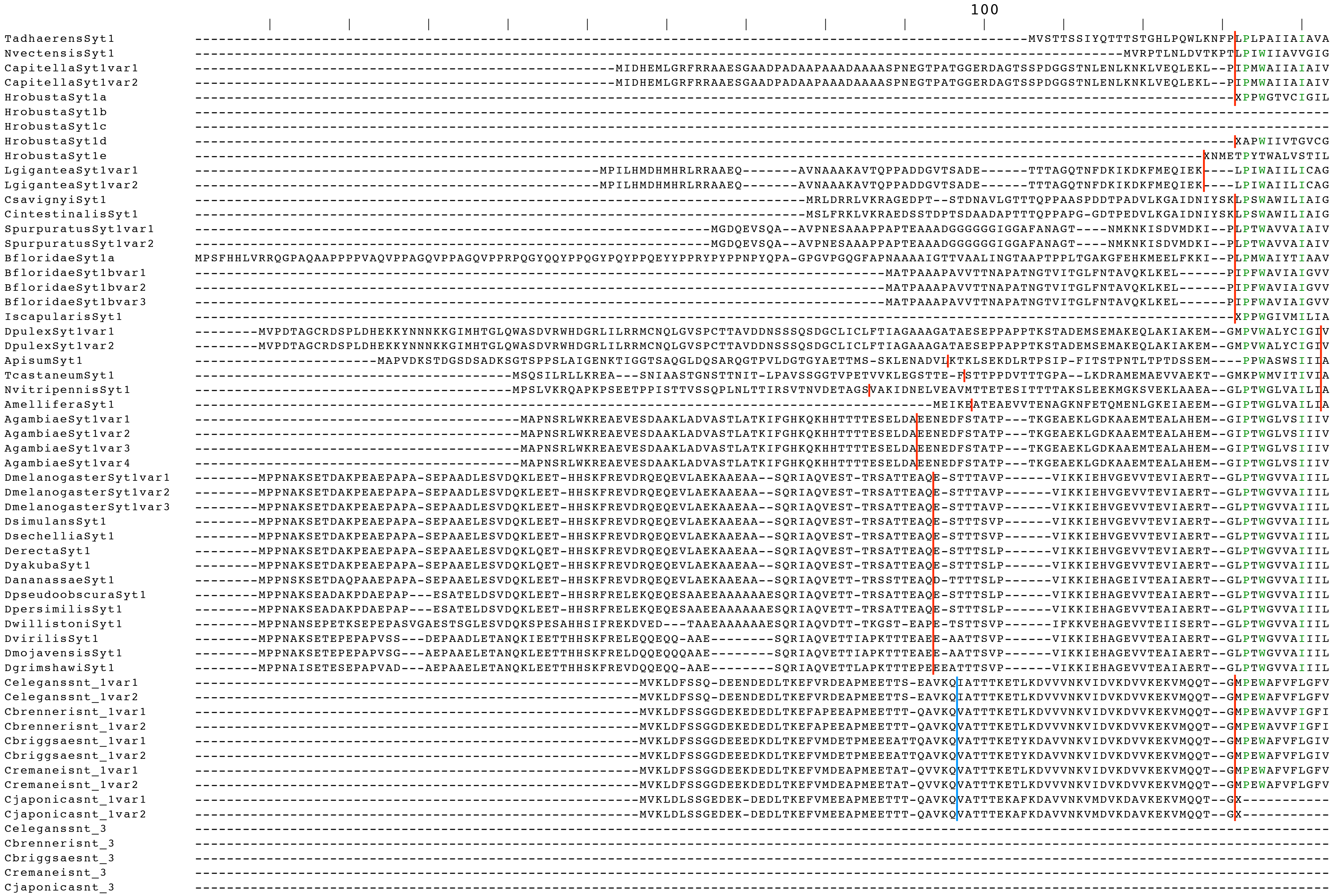
100
TadhaerensSyt1
NvectensisSyt1
CapitellaSyt1var1
CapitellaSyt1var2
HrobustaSyt1a
HrobustaSyt1b
HrobustaSyt1c
HrobustaSyt1d
HrobustaSyt1e
LgiganteaSyt1var1
LgiganteaSyt1var2
CsavignyiSyt1
CintestinalisSyt1
SpurpuratusSyt1var1
SpurpuratusSyt1var2
BfloridaeSyt1a
BfloridaeSyt1bvar1
BfloridaeSyt1bvar2
BfloridaeSyt1bvar3
IscapularisSyt1
DpulexSyt1var1
DpulexSyt1var2
ApisumSyt1
TcastaneumSyt1
NvitripennisSyt1
AmelliferaSyt1
AgambiaeSyt1var1
AgambiaeSyt1var2
AgambiaeSyt1var3
AgambiaeSyt1var4
DmelanogasterSyt1var1
DmelanogasterSyt1var2
DmelanogasterSyt1var3
DsimulansSyt1
DsechelliaSyt1
DerectaSyt1
DyakubaSyt1
DananassaeSyt1
DpseudoobscuraSyt1
DpersimilisSyt1
DwillistoniSyt1
DvirilisSyt1
DmojavensisSyt1
DgrimshawiSyt1
Celeganssnt_1var1
Celeganssnt_1var2
Cbrennerisnt_1var1
Cbrennerisnt_1var2
Cbriggsaesnt_1var1
Cbriggsaesnt_1var2
Cremaneisnt_1var1
Cremaneisnt_1var2
Cjaponicasnt_1var1
Cjaponicasnt_1var2
Celeganssnt_3
Cbrennerisnt_3
Cbriggsaesnt_3
Cremaneisnt_3
Cjaponicasnt_3

```
                                                                          200
TadhaerensSyt1             CAVLVFALVLCICCKCCCRRKKPKNDKTKQGFKEKVSLQSAQSLGVSLKER------------------------------------------------------------VQPEVEGVNKEME---LESKQIDPSEYVGELK
NvectensisSyt1             AAVIVIFCIYCCCCRKCGKKKK-KDDK--RGGKERVDFRAVQ-IGASYQEK------------------------------------------------------------VQPSMDELDYNSE---DYHSDLSSGVKIGRIN
CapitellaSyt1var1          ALLLLLCCCFCICKRCCKRKKKDKDKK--KGLKGAVDLGSVKILGNSYKEK------------------------------------------------------------VQPDLEELEVNME-DNEDAESTKSEVKLGKLQ
CapitellaSyt1var2          ALLLLLCCCFCICKRCCKRKKKDKDKK--KGLKGAVDLGSVKILGNSYKEK------------------------------------------------------------VQPDLEELEVNME-DNEDAESTKSEVKLGKLQ
HrobustaSyt1a              ALLILLCCCGCLL-RMCWKRRKGKEGK--KGLKGAVDLKNVQLLGSSMKEK------------------------------------------------------------VQPDLESLEGNME-DNEGVENAKEEVKLGKLQ
HrobustaSyt1b              -----------------------------------------------------------------------------------------------------------------------------------------------
HrobustaSyt1c              -----------------------------------------------------------------------------------------------------------------------------------------------
HrobustaSyt1d              SVVLLLFLMWCLCRCCTGKKKASSK----KGAKGYIDISQVQQLGLSYKEK------------------------------------------------------------VQPDLNELKENME-------ENEDAQHRGRLH
HrobustaSyt1e              IVIVVIFVIFCLKAKFCRKLKFGTEAS---TLGGKVDVDAVKALGHSYKER------------------------------------------------------------VQPELNDLKIDDCVANKKEKDDGGDVKLGRVH
LgiganteaSyt1var1          ALLFLVCCTYCMCKRCCRKKKK-KEGK--KGLKGAVDLKSVQLLGNAYKEK------------------------------------------------------------VQPDLDELQVNME-DNEDAESTKSEVKLGKLQ
LgiganteaSyt1var2          ALLFLVCCTYCMCKRCCRKKKK-KEGK--KGLKGAVDLKSVQLLGNAYKEK------------------------------------------------------------VQPDLDELQVNME-DNEDAESTKSEVKLGKLQ
CsavignyiSyt1              AGLILLCILYCCCKRCCCKKKKKKQEK--KGLKNAIDLKSVQNMGDSYREK------------------------------------------------------------VQPDVDDLDTGAEKEGEE------EKKLGKLQ
CintestinalisSyt1          AGLILLCVLYCCCKRCCCKKKKKKQEK--KGLKNAIDLKSVKNMGDSYREK------------------------------------------------------------VQPDVDEVDIG-EKEDEE------EKKLGKLQ
SpurpuratusSyt1var1        AGLILLCCCFCICKKCCCKKRKKKEGK--KGLKGAVDLKSVQMLGNSYKEK------------------------------------------------------------IQPDVDDLDNGGD-EEGDTDSVKSEIKLGKLQ
SpurpuratusSyt1var2      ● AGLILLCCCFCICKKCCCKKRKKKEGK--KGLKGAVDLKSVQMLGNSYKEK--------------------------------------------------------------PDVDDLDNGGD-EEGDTDSVKSEIKLGKLQ
BfloridaeSyt1a             IALILLACIACICKKCVFKKKKKGKGE--KGGKNVIDLKDVKMLGNSYKEK------------------------------------------------------------VQPDVEEIDAGLEGEGGDNESVQEKVNLGKLQ
BfloridaeSyt1bvar1         AALLLLCCMVCICKKCCCKKKKAKQGK--KGLKDSVDLQEVKMLGNQYKEK-------------------------------------------------------------------VQPDMDDLDSYGDEEDGAPKLGQLQ
BfloridaeSyt1bvar2       ● AALLLLCCMVCICKKCCCKKKKAKQGK--KGLKDSVDLQEVKMLGNQYKEKSDSEDDLEDIEVEKPGIWNPNMEGGGSGSDADGSDNEEDGKKKKKKKKKRSFFGKLGNPDYVVSREKVQPDMDDLDSYGDEEDGAPKLGQLQ
BfloridaeSyt1bvar3       ● AALLLLCCMVCICKKCCCKKKKAKQGK--KGLKDSVDLQE-----------SDSEDDLEDIEVEKPGIWNPNMEGGGSGSDADGSDNEEDGKKKKKKKKKRSFFGKLGNPDYVVSREKVQPDMDDLDSYGDEEDGAPKLGQLQ
IscapularisSyt1            VALLLLCICFCCLRRWC-R-RRKKDAK--KGLKGAVDLKGVQLLGNAYKEK------------------------------------------------------------VQPDMEDLQDNMENNEGDGESKKSEEKLGRLQ
DpulexSyt1var1             VALVILLIAFCCIRRCC-RKRRPKDAK--KGLKGAVDLKSVQLLGNAYKEK------------------------------------------------------------VQPDMDELTDNVEDIAVEGE-KKEEQKLGRLQ
DpulexSyt1var2             VALVILLIAFCCIRRCC-RKRRPKDAK--KGLKGAVDLKSVQLLGNAYKEK------------------------------------------------------------VQPDMDELTDNVEDIAVEGE-KKEEQKLGRLQ
ApisumSyt1                 VVLLAVGFVGFCIHRFF-RKRRTKDGK--KG-KGVVDLKAVQLLGSAYKEK------------------------------------------------------------IQPDMEELADNAEEIAEEGDSTKDEKKLGRLQ
TcastaneumSyt1             ICAIILGICFCCIRRCF-RKRRAKDGK--KGMKG-VDLKSVQLLGSSYKEK------------------------------------------------------------VQPDMDELTENAEE-PDEAE-KPEVQKLGKLQ
NvitripennisSyt1           VAIIVLGFCGFCIRRCF-RKRRSKDGK--KGLKGAVDLKSVQLLGSTYKDK------------------------------------------------------------VQPDMEELTDNAEE-PDEAESKQSEVKLGRLQ
AmelliferaSyt1             VSVVVLGICFCCIRRCC-RKRRSKDGK--KGLKGAVDLKSVQLLGSTYKDK------------------------------------------------------------VQPDMEELTDNAEE-PDEAESKQSEVKLGKLQ
AgambiaeSyt1var1           VVLIILGICGFCIRRCF-RKRRSKDGK--KGMKG-VDLKSVQLLGSAYKEK------------------------------------------------------------VQPDMEELTENAEE-GDEGESKQSEQKLGKLQ
AgambiaeSyt1var2         ● VVLIILGICGFCIRRCF-RKRRSKDGK--KGMKG-VDLKS-----------------------------------------------------------------------VQPDMEELTENAEE-GDEGESKQSEQKLGKLQ
AgambiaeSyt1var3           VVLIILGICGFCIRRCF-RKRRSKDGK--KGMKG-VDLKSVQLLGSAYKEK------------------------------------------------------------VQPDMEELTENAEE-GDEGESKQSEQKLGKLQ
AgambiaeSyt1var4           VVLIILGICGFCIRRCF-RKRRSKDGK--KGMKG-VDLKSVQLLGSAYKEK------------------------------------------------------------VQPDMEELTENAEE-GDEGESKQSEQKLGKLQ
DmelanogasterSyt1var1      VFLVVFGIIFFCVRRFL-KKRRTKDGK---GKKG-VDMKSVQLLGSAYKEK------------------------------------------------------------VQPDMEELTENAEE--GDEEDKQSEQKLGRLN
DmelanogasterSyt1var2    ● VFLVVFGIIFFCVRRFL-KKRRTKDGK---GKKG-VDMKSVQLLGSAYKEK--------------------------------------------------------------PDMEELTENAEE--GDEEDKQSEQKLGRLN
DmelanogasterSyt1var3      VFLVVFGIIFFCVRRFL-KKRRTKDGK---GKKG-VDMKSVQLLGSAYKEK------------------------------------------------------------VQPDMEELTENAEE--GDEEDKQSEQKLGRLN
DsimulansSyt1              VFLVVFGIIFFCVRRFL-KKRRTKDGK---GKKG-VDMKSVQLLGSAYKEK------------------------------------------------------------VQPDMEELTENAEE--GDEEDKQSEQKLGRLN
DsechelliaSyt1             VFLVVFGIIFFCVRRFL-KKRRTKDGK---GKKG-VDMKSVQLLGSAYKEK------------------------------------------------------------VQPDMEELTENAEE--GDEEDKQSEQKLGRLN
DerectaSyt1                VFLVVFGIIFFCVRRFL-KKRRTKDGK---GKKG-VDMKSVQLLGSAYKEK------------------------------------------------------------VQPDMEELTENAEE--GDEEDKQSEQKLGRLN
DyakubaSyt1                VFLVVFGIIFFCVRRFL-KKRRTKDGK---GKKG-VDMKSVQLLGSAYKEK------------------------------------------------------------VQPDMEELTENAEE--GDEEDKQSEQKLGRLN
DananassaeSyt1             VFLVVFGIIFFCVRRFL-KKRRTKDGK---GKKG-VDMKSVQLLGSAYKEK------------------------------------------------------------VQPDMEELTENAEE--GDEEDKQSEQKLGRLN
DpseudoobscuraSyt1         VFLVVFGIIFFCVRRFL-KKRRTKDGK---GKKG-VDMKSVQLLGSAYKEK------------------------------------------------------------VQPDMEELAENAEE---DEEDKQSEQKLGRLN
DpersimilisSyt1            VFLVVFGIIFFCVRRFL-KKRRTKDGK---GKKG-VDMKSVQLLGSAYKEK------------------------------------------------------------VQPDMEELAENAEE---DEEDKQSEQKLGRLN
DwillistoniSyt1            VFLVVFGIIFFCVRRFL-KKRRTKDGK---GKKG-VDMKSVQLLGSAYKEK------------------------------------------------------------VQPDMEELTENAEE--GDEEDKQSEQKLGRLN
DvirilisSyt1               VFLVVFGIIFFCVRRFL-KKRRTKDGK---GKKG-VDMKSVQLLGSAYKEK------------------------------------------------------------VQPDMEELTENAEE--GDEEDKQSEQKLGRLN
DmojavensisSyt1            VFLVVFGIIFFCVRRFL-KKRRTKDGK---GKKG-VDMKSVQLLGSAYKEK------------------------------------------------------------VQPDMEELTENAEE--GDEEDKQSEQKLGRLN
DgrimshawiSyt1             VFLVVFGIIFFCVRRFL-KKRRTKDGK---GKKG-VDMKSVQLLGSAYKEK------------------------------------------------------------VQPDMEELTENAEE--GDEEDKQSEQKLGRLN
Celeganssnt_1var1          FILLVLACAFCLIRKLFGKKRHGEKNK--KGGLKGFFGKGQDVVDGKNIQG------------------------------------------------------------MAQDLEELGDAMEQNEKEQAEEKEEVKLGRIQ
Celeganssnt_1var2          FILLVLACAFCLIRKLFGKKRHGEKNK--KGGLKGFFGKGQDVVDGKNIQG------------------------------------------------------------MAQDLEELGDAMEQNEKEQAEEKEEVKLGRIQ
Cbrennerisnt_1var1         FILLLLVCGFLLIRKLFGKKRHGEKNK--KGGLKGFFGKGQDVVDGKNIQG------------------------------------------------------------MAQDLEELGDAMEQNEKAQAEEKEEVKLGRIQ
Cbrennerisnt_1var2         FILLLLVCGFLLIRKLFGKKRHGEKNK--KGGLKGFFGKGQDVVDGKNIQG------------------------------------------------------------MAQDLEELGDAMEQNEKAQAEEKEEVKLGRIQ
Cbriggsaesnt_1var1         FIFFVLACAFFIIRKLFGKKRHGEKNK--KGGLKGFFGKGQDVVDGKNIQG------------------------------------------------------------MAQDLEELGDAMEQNEKAQAEEKEEVKLGRIQ
Cbriggsaesnt_1var2         FIFFVLACAFFIIRKLFGKKRHGEKNK--KGGLKGFFGKGQDVVDGKNIQG------------------------------------------------------------MAQDLEELGDAMEQNEKAQAEEKEEVKLGRIQ
Cremaneisnt_1var1          FILLVLACAFFIIRKLFGKKRHGEKNK--KGGLKGFFGKGQDVVDGKNIQG------------------------------------------------------------MAQDLEELGDAMEQNEKAQAEEKEEVKLGRIQ
Cremaneisnt_1var2          FILLVLACAFFIIRKLFGKKRHGEKNK--KGGLKGFFGKGQDVVDGKNIQG------------------------------------------------------------MAQDLEELGDAMEQNEKAQAEEKEEVKLGRIQ
Cjaponicasnt_1var1         ---------------------------------------------------------------------------------------------------------------MAQDLEELGDAMEQNEKAQADEKEEVKLGRIQ
Cjaponicasnt_1var2         ---------------------------------------------------------------------------------------------------------------MAQDLEELGDAMEQNEKAQADEKEEVKLGRIQ
Celeganssnt_3              ---------------------------------------------------------------------------------------------------------------------------MSVSKKKDEDKEKKFLGRLQ
Cbrennerisnt_3             ---------------------------------------------------------------------------------------------------------------------------MSVSKKKDEDKEKKFLGRLQ
Cbriggsaesnt_3             ---------------------------------------------------------------------------------------------------------------------------MSVSKKKDEDKEKKFLGRLQ
Cremaneisnt_3              ---------------------------------------------------------------------------------------------------------------------------MSVSKKKDEDKEKKFLGRLQ
Cjaponicasnt_3             ---------------------------------------------------------------------------------------------------------------------------MSVSKKKDDDKEKKFLGRLQ
```

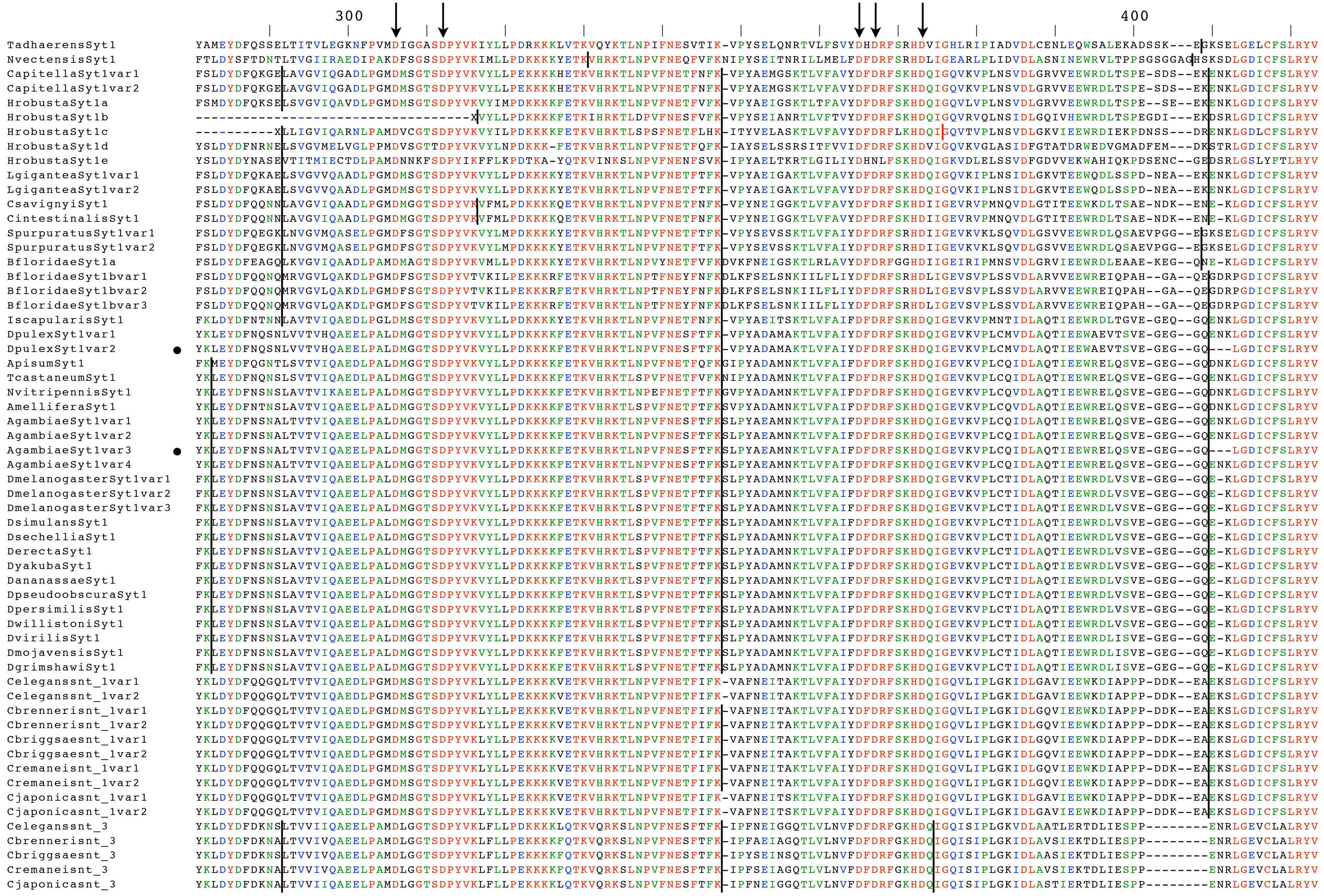
300
400
TadhaerensSyt1
NvectensisSyt1
CapitellaSyt1var1
CapitellaSyt1var2
HrobustaSyt1a
HrobustaSyt1b
HrobustaSyt1c
HrobustaSyt1d
HrobustaSyt1e
LgiganteaSyt1var1
LgiganteaSyt1var2
CsavignyiSyt1
CintestinalisSyt1
SpurpuratusSyt1var1
SpurpuratusSyt1var2
BfloridaeSyt1a
BfloridaeSyt1bvar1
BfloridaeSyt1bvar2
BfloridaeSyt1bvar3
IscapularisSyt1
DpulexSyt1var1
DpulexSyt1var2
ApisumSyt1
TcastaneumSyt1
NvitripennisSyt1
AmelliferaSyt1
AgambiaeSyt1var1
AgambiaeSyt1var2
AgambiaeSyt1var3
AgambiaeSyt1var4
DmelanogasterSyt1var1
DmelanogasterSyt1var2
DmelanogasterSyt1var3
DsimulansSyt1
DsecheliaSyt1
DerectaSyt1
DyakubaSyt1
DananassaeSyt1
DpseudoobscuraSyt1
DpersimilisSyt1
DwillistoniSyt1
DvirilisSyt1
DmojavensisSyt1
DgrimshawiSyt1
Celeganssnt_1var1
Celeganssnt_1var2
Cbrennerisnt_1var1
Cbrennerisnt_1var2
Cbriggsaesnt_1var1
Cbriggsaesnt_1var2
Cremaneisnt_1var1
Cremaneisnt_1var2
Cjaponicasnt_1var1
Cjaponicasnt_1var2
Celeganssnt_3
Cbrennerisnt_3
Cbriggsaesnt_3
Cremaneisnt_3
Cjaponicasnt_3

```
                                500
TadhaerensSyt1            PTGGKLTIGIMEAKGLKKMDVGGSSDPYVKISLI--IDGKRIKKKKTIVKKRTLDPVWNEAYMFK-VPFEKIKDTKIVLAVMDHDRLGKSDLIGQVVVGGSESGAELRHWSDMLSSPRRPIAQWHVLVD------------
NvectensisSyt1            PSSGKLQITIVEAKSLKSMDLTGYSDPYVKIALV--QEGRKIKKKKTTVKKRTLNPYYNETFTFT-VAFEKIEQTSLIISVLDYDRVGKSEMIGKCVVGELSSGADLRHWADMLASPRRSVAQWHTLHN------------
CapitellaSyt1var1         PTAGKLTVVILEAKNLKKMDVGGLSDPYVKLALY--QGTKRLKKKKTTIKKRTLNPYYNESFTFE-VPFEQIQKVTMIITVVDYDRIGTSEPIGRVVLGCNSTGTELRHWSDMLANPRRPIAQWHTLQEMPEK--------
CapitellaSyt1var2       ● PTAGKLTVVILEAKNLKKMDVGGLSDPYVKLSLM--LNGKRIKKKKTTIKKCTLNPYYNESFTFE-VPFEQIQKVTMIITVVDYDRIGTSEPIGRVVLGCNSTGTELRHWSDMLANPRRPIAQWHTLQEMPEK--------
HrobustaSyt1a             PTAGKLTIVILEAKNLKKMDVGGLSDPYVKLALL--MNGKRIKKKKSTIKKCTLNPYWNESFSFE-VAFEQIQKISLDITVVDYDRIGTSDPIGHVLLGCNATGTELRHWSDMLANPRRPIAQWHTLQEVPEKN-------
HrobustaSyt1b             PTAGKLTVVILEAKNLKKMDVGGLSDPYVKLCLT--MNGKRLKKKKTTIRKCTLNPYFNESFSFE-VPFEQIQKITLILTVVDYDRIGSSDPIGRVILGCNSSGTELRHWSDMLANPRRPIAQWHALKELPDKNQGGDDD-
HrobustaSyt1c             PTSGKLTVVILEAKNLKKMDVGGLSDPYVKLSLL--QNGKRIKKKKTTIKKCTLNPYYNEAFTFE-VPFETIQQTMLVVTVVDYDRISCSDPIGQVVLGCHSSGTELRHWSDTLANPRRPIAQWHTLREISEKYD------
HrobustaSyt1d             PTAAKLVIVILEAKNLKKMDISGLSDPYVKVTLY--IGGKKVKKKKSTVKKRTLNPYFNESFTFE-VTFDQIQKASLVITVMDYDKIGFSDPMGQVVVSSKASGTGLRHWSDMLANPRRPIAQWHELQEVTEKK-------
HrobustaSyt1e             PTAGKLTVVILEAKNLKKMDSIGLSDPYVKISLM--MGSKRIKKKKTSVKKKTLNPYYNEEFIFD-VTFDQIQKTSLIISVIDYDLIGKSETMGAITVGCNSTGSPLKHWSEMLSNPRRPIAQWHELEMPKDERKK-----
LgiganteaSyt1var1         PTAGKLTVVILEAKNLKKMDVGGLSDPYVKIALY--QGSKRLKKKKTTIKKRTLNPYFNESFTYE-VPFEQIQKVQLIITVVDYDRIGTSEPIGRVLLGCNSTGTELRHWSDMLANPRRPIAQWHTLQEVPEKN-------
LgiganteaSyt1var2       ● PTAGKLTVVILEAKNLKKMDVGGLSDPYVKISMN--LNGKRIKKKKTTIKKCTLNPYYNESFTFE-VPFEQIQKVQLIITVVDYDRIGTSEPIGRVLLGCNSTGTELRHWSDMLANPRRPIAQWHTLQEVPEKN-------
CsavignyiSyt1             PTAGKLTVVILEAKNLKKMDVGGLSDPYVKITLM--QGGKRLKKKKTTIKKNTLNPYFNESFSFE-VPFEQIQKVSLVVSVFDYDRMGKNDPIGKLVLGCNASGTELRHWSDMLASPRRPIAQWHTLQLAEEG--------
CintestinalisSyt1         PTAGKLTVVILEAKNLKKMDVGGLSDPYVKITLM--QGGKRLKKKKTTIKKNTLNPYFNESFSFE-VPFEQIQKVALVVSVFDYDRMGKNDPIGKLVLGCNASGTELRHWSDMLASPRRPIAQWHTLQMAEEG--------
SpurpuratusSyt1var1       PTAGKLTVVILEAKNLKKMDVGGLSDPYVKISLY--MNNKRMKKKKTTIKKRTLNPYYNESFGFE-VPFEQIQKVTLVVTVVDYDRMGSSEPIGKVVLGCNATGAGLRHWSDMLASPRRPIAQWHTLQEPEEGK-------
SpurpuratusSyt1var2       PTAGKLTVVILEAKNLKKMDVGGLSDPYVKISLY--MNNKRMKKKKTTIKKRTLNPYYNESFGFE-VPFEQIQKVTLVVTVVDYDRMGSSEPIGKVVLGCNATGAGLRHWSDMLASPRRPIAQWHTLQEPEEGK-------
BfloridaeSyt1a            PTKGQLTVVILECKQLKKMDLGGASDPYVKIYLM--MNGKRLKKKKTTVKKCTLNPYYNESFKFD-IPFDQIQKVELVITVLDWDAIGGSDPIGRTVCGCNATGAELRHWSDMLANPRRPICQWHTLQEVPEDK-------
BfloridaeSyt1bvar1        PTAGKLTIVILEAKNLKKCDVGGLSDPYVKMQLY--SNNKRLKKKKTTIKKRTLNPYYNESFTFE-VPFEQIQRVELKLTVMDWDRTSKNDPIGELRLGPNAAGAELRHWSDMLANPRRPIAQWHTLTELEK---------
BfloridaeSyt1bvar2        PTAGKLTIVILEAKNLKKCDVGGLSDPYVKMQLY--SNNKRLKKKKTTIKKRTLNPYYNESFTFE-VPFEQIQRVELKLTVMDWDRTSKNDPIGELRLGPNAAGAELRHWSDMLANPRRPIAQWHTLTELEK---------
BfloridaeSyt1bvar3        PTAGKLTIVILEAKNLKKCDVGGLSDPYVKMQLY--SNNKRLKKKKTTIKKRTLNPYYNESFTFE-VPFEQIQRVELKLTVMDWDRTSKNDPIGELRLGPNAAGAELRHWSDMLANPRRPIAQWHTLTELEK---------
IscapularisSyt1           PTAGKLTVVILEAKNLKKMDVGGLSDPYVKIALM--MNGKRLKKKKTSIKKCTLNPYYNESFTFE-VPFEQIQKVQLVVTVVDYDRIGTSEPIGKVVLGCNATGTELRHWMDMLASPRRPIAQWHSLKDPSDGQDN-----
DpulexSyt1var1            PTAGKLTVVILEAKNLKKMDVGGLSDPYVKIALM--QNGKRLKKKKTSIKKCTLNPYYNESFTFE-VPFEQIQKVQLVVTVVDYDRIGTSDPIGKAILGCNATGTELRHWSDMLASPRRPIAQWHTLKEPDDGDKKD----
DpulexSyt1var2            PTAGKLTVVILEAKNLKKMDVGGLSDPYVKIALM--QNGKRLKKKKTSIKKCTLNPYYNESFTFE-VPFEQIQKVQLVVTVVDYDRIGTSDPIGKAILGCNATGTELRHWSDMLASPRRPIAQWHTLKEPDDGDKKD----
ApisumSyt1                PTAGKLTVVILEAKNLKKMDVGGLSDPYVKIALM--QNGKRLKKKKTSIKKCTLNPYYNESFTFE-VPFEQIQKVNLVVTVVDYDRIGTSEPIGKVVLGYNATGTELRHWSDMLASPRRPIAQWHTLKDPDDDAKN-----
TcastaneumSyt1            PTAGKLTVVILEAKNLKKMDVGGLSDPYVKIALV--QNGKRLKKKKTSIKKCTLNPYYNESFTFE-VPFEQIQKVNLVVTVVDYDRIGTSEPIGKVVLGYNASGTELRHWSDMLASPRRPIAQWHTLKDPED-EKKD----
NvitripennisSyt1          PTAGKLTVVILEAKNLKKMDVGGLSDPYVKIALM--QNGKRLKKKKTSIKKCTLNPYYNESFTFE-VPFEQIQKVNLMVTVVDYDRIGTSEPIGKVILGYNASGTELRHWSDMLASPRRPIAQWHTLKDPEDGDKKD----
AmelliferaSyt1            PTAGKLTVVILEAKNLKKMDVGGLSDPYVKIALM--QNGKRLKKKKTSIKKCTLNPYYNESFTFE-VPFEQIQKVQLVVTVVDYDRIGTSEPIGKVVLGYNASGTELRHWSDMLASPRRPIAQWHTLKDPEDGDKKD----
AgambiaeSyt1var1          PTAGKLTVVILEAKNLKKMDVGGLSDPYVKIALM--QNGKRLKKKKTSIKKCTLNPYYNESFSFE-VPFEQIQKVNLVVTVVDYDRIGTSEPIGKVVLGYNASGTELRHWSDMLASPRRPIAQWHTLKDPDD-DKKD----
AgambiaeSyt1var2          PTAGKLTVVILEAKNLKKMDVGGLSDPYVKIALM--QNGKRLKKKKTSIKKCTLNPYYNESFSFE-VPFEQIQKVNLVVTVVDYDRIGTSEPIGKVVLGYNASGTELRHWSDMLASPRRPIAQWHTLKDPDD-DKKD----
AgambiaeSyt1var3          PTAGKLTVVILEAKNLKKMDVGGLSDPYVKIALM--QNGKRLKKKKTSIKKCTLNPYYNESFSFE-VPFEQIQKVNLVVTVVDYDRIGTSEPIGKVVLGYNASGTELRHWSDMLASPRRPIAQWHTLKDPDD-DKKD----
AgambiaeSyt1var4        ● PTAGKLTVVILEAKNLKKMDVGGLSDPYVKIALM--QNGKRLKKKKTSVRKCTLNPYYNESFSFE-VPFEQMQKVNLVVTVVDYDRIGTSEPIGKVVLGYNASGTELRHWSDMLASPRRPIAQWHTLKDPDD-DKKD----
DmelanogasterSyt1var1     PTAGKLTVVILEAKNLKKMDVGGLSDPYVKIAIM--QNGKRLKKKKTSIKKCTLNPYYNESFSFE-VPFEQIQKICLVVTVVDYDRIGTSEPIGRCILGCMGTGTELRHWSDMLASPRRPIAQWHTLKDPEETDEILKNMK
DmelanogasterSyt1var2     PTAGKLTVVILEAKNLKKMDVGGLSDPYVKIAIM--QNGKRLKKKKTSIKKCTLNPYYNESFSFE-VPFEQIQKICLVVTVVDYDRIGTSEPIGRCILGCMGTGTELRHWSDMLASPRRPIAQWHTLKDPEETDEILKNMK
DmelanogasterSyt1var3   ● PTAGKLTVVILEAKNLKKMDVGGLSDPYVKVAIM--QNGKRLKKRKTSVRKCTLNPYYNESFSFE-VPFEQMQKICLVVTVVDYDRIGTSEPIGRCILGCMGTGTELRHWSDMLASPRRPIAQWHTLKDPEETDEILKNMK
DsimulansSyt1             PTAGKLTVVILEAKNLKKMDVGGLSDPYVKIAIM--QNGKRLKKKKTSIKKCTLNPYYNESFSFE-VPFEQIQKICLVVTVVDYDRIGTSEPIGRCILGCMGTGTELRHWSDMLASPRRPIAQWHTLKDPEETDEILKNMK
DsechelliaSyt1            PTAGKLTVVILEAKNLKKMDVGGLSDPYVKIAIM--QNGKRLKKKKTSIKKCTLNPYYNESFSFE-VPFEQIQKICLVVTVVDYDRIGTSEPIGRCILGCMGTGTELRHWSDMLASPRRPIAQWHTLKDPEETDEILKNMK
DerectaSyt1               PTAGKLTVVILEAKNLKKMDVGGLSDPYVKIAIM--QNGKRLKKKKTSIKKCTLNPYYNESFSFE-VPFEQIQKICLVVTVVDYDRIGTSEPIGRCILGCMGTGTELRHWSDMLASPRRPIAQWHTLKDPEETDEILKNMK
DyakubaSyt1               PTAGKLTVVILEAKNLKKMDVGGLSDPYVKIAIM--QNGKRLKKKKTSIKKCTLNPYYNESFSFE-VPFEQIQKICLVVTVVDYDRIGTSEPIGRCILGCMGTGTELRHWSDMLASPRRPIAQWHTLKDPEETDEILKNMK
DananassaeSyt1            PTAGKLTVVILEAKNLKKMDVGGLSDPYVKIAIM--QNGKRLKKKKTSIKKCTLNPYYNESFSFE-VPFEQIQKICLVVTVVDYDRIGTSEPIGRCILGCMGTGTELRHWSDMLASPRRPIAQWHTLKDPEETDEILKNMK
DpseudoobscuraSyt1        PTAGKLTVVILEAKNLKKMDVGGLSDPYVKIAIM--QNGKRLKKKKTSIKKCTLNPYYNESFSFE-VPFEQIQKICLVVTVVDYDRIGTSEPIGRCILGCMGTGTELRHWSDMLASPRRPIAQWHTLKDPEETDEILKNMK
DpersimilisSyt1           PTAGKLTVVILEAKNLKKMDVGGLSDPYVKIAIM--QNGKRLKKKKTSIKKCTLNPYYNESFSFE-VPFEQIQKICLVVTVVDYDRIGTSEPIGRCILGCMGTGTELRHWSDMLASPRRPIAQWHTLKDPEETDEILKNMK
DwillistoniSyt1           PTAGKLTVVILEAKNLKKMDVGGLSDPYVKIAIM--QNGKRLKKKKTSIKKCTLNPYYNESFSFE-VPFEQIQKICLVVTVVDYDRIGTSEPIGRCILGCMGTGTELRHWSDMLASPRRPIAQWHTLKDPEETDEILKNMK
DvirilisSyt1              PTAGKLTVVILEAKNLKKMDVGGLSDPYVKIAIM--QNGKRLKKKKTSIKKCTLNPYYNESFSFE-VPFEQIQKICLVVTVVDYDRIGTSEPIGRCILGCMGTGTELRHWSDMLASPRRPIAQWHTLKDPEETDEILKNMK
DmojavensisSyt1           PTAGKLTVVILEAKNLKKMDVGGLSDPYVKIAIM--QNGKRLKKKKTSIKKCTLNPYYNESFSFE-VPFEQIQKICLVVTVVDYDRIGTSEPIGRCILGCMATGTELRHWSDMLASPRRPIAQWHTLKDPEETDEILKNMK
DgrimshawiSyt1            PTAGKLTVVILEAKNLKKMDVGGLSDPYVKIAIM--QNGKRLKKKKTSIKKCTLNPYYNESFSFE-VPFEQIQKICLVVTVVDYDRIGTSEPIGRCILGCMGTGTELRHWSDMLASPRRPIAQWHTLKDPEETDEILKNMK
Celeganssnt_1var1         PTAGKLTVVILEAKNLKKMDVGGLSDPYVKLEMFGEDR-KRLKKKKTNIKRKTLNPYFNESFVFDKLPLHKMKKVSLMITVMDYDKLGSNDAIGRCLLGCNGTGAELRHWMDMLASPRRPIAQWHTLGPVEEEGDKKDDKK
Celeganssnt_1var2       ● PTAGKLTVVILEAKNLKKMDVGGLSDPYVKIVLM--QGGKRLKKKKTSIKKCTLNPYYNESFSFE-VPFEQIQKVSLMITVMDYDKLGSNDAIGRCLLGCNGTGAELRHWMDMLASPRRPIAQWHTLGPVEEEGDKKDDKK
Cbrennerisnt_1var1        PTAGKLTVVVLEAKNLKKMDVGGLSDPYVKLEMFGEDR-KRLKKKKTSIKRKTLNPYYNESFVFDKLQLHKIKKVSLMITVMDYDKLGSNDAIGRCLLGCNGTGAELRHWMDMLASPRRPIAQWHTLGPVEEEGDKKDDKK
Cbrennerisnt_1var2      ● PTAGKLTVVVLEAKNLKKMDVGGLSDPYVKIVLM--QGGKRLKKKKTSIKKCTLNPYYNESFSFE-VPFEQIQKVSLMITVMDYDKLGSNDAIGRCLLGCNGTGAELRHWMDMLASPRRPIAQWHTLGPVEEEGDKKDDKK
Cbriggsaesnt_1var1        PTAGKLTVVVLEAKNLKKMDVGGLSDPYVKLEMFGEDKKKRLKKKKTSIKRNTLNPYYNESFVFDKLPLHKMKKVSLMITVMDYDKLGSNDAIGRCLLGCNGTGAELRHWMDMLASPRRPIAQWHTLGPVEEEGDKKDEKK
Cbriggsaesnt_1var2      ● PTAGKLTVVVLEAKNLKKMDVGGLSDPYVKIVLM--QGGKRLKKKKTSIKKCTLNPYYNESFSFE-VPFEQIQKVSLMITVMDYDKLGSNDAIGRCLLGCNGTGAELRHWMDMLASPRRPIAQWHTLGPVEEEGDKKDEKK
Cremaneisnt_1var1         PTAGKLTVVVLEAKNLKKMDVGGLSDPYVKLEMFGEDKGKRLKKKKTSIKRKTLNPYYNESFVFDKLPLHKMKKVSLMITVMDYDKLGSNDAIGRCLLGCNGTGAELRHWMDMLASPRRPIAQWHTLGPVEEEGDKKDEKK
Cremaneisnt_1var2       ● PTAGKLTVVVLEAKNLKKMDVGGLSDPYVKIVLM--QGGKRLKKKKTSIKKCTLNPYYNESFSFE-VPFEQIQKVSLMITVMDYDKLGSNDAIGRCLLGCNGTGAELRHWMDMLASPRRPIAQWHTLGPVEEEGDKKDEKK
Cjaponicasnt_1var1        PTAGKLTVVILEAKNLKKMDVGGLSDPYVKLEMFG-DDGKRLKKKKTNIKRNTLNPYYNESFVFDKLQLHKIKKVSLMITVMDYDKLGSNDAIGRCLLGCNGAGAELRHWMDMLASPRRPIAQWHTLQPVEEEGEKKEEKK
Cjaponicasnt_1var2      ● PTAGKLTVVILEAKNLKKMDVGGLSDPYVKIVLM--QGGKRLKKKKTSIKKCTLNPYYNESFSFE-VPFEQIQKVSLMITVMDYDKLGSNDAIGRCLLGCNGAGAELRHWMDMLASPRRPIAQWHTLQPVEEEGEKKEEKK
Celeganssnt_3             PNKNKLSVVVMECKNLKKMDVLGLSDPYVKIYLM--MGTKRLEKKKTTIKMKTLNPYYNESFSFD-VTSEKMQRVHLHVTVSDYDRVGSNERIGQVIIGTCATGVALKQWNDMLATPRRSVAQWHTLVPFNDD--------
Cbrennerisnt_3            PNKNKLSVVVMECKNLKKMDVLGLSDPYVKIYSM--MGTKRLEKKKTTIKMKTLNPYYNESFSFD-VTPEKMMRVHLHVTVSDYDRVGSNERIGQVIIGTCATGVALKQWNDMLATPRRSVAQWHTLVPFNDD--------
Cbriggsaesnt_3            PNKNKLSVVVMECKNLKKMDVLGLSDPYVKIYLM--MGTKRLEKKKTTIKMKTLNPYYNESFSFD-VTPEKMMRVHLHVTVSDYDRVGSNERIGQVIIGTCATGVALKQWNDMLATPRRSVAQWHTLVPFNDD--------
Cremaneisnt_3             PNKNKLSVVVMECKNLKKMDVLGLSDPYVKIYLM--MGTKRLEKKKTTIKMKTLNPYYNESFSFD-VTPEKMMRVHLHVTVSDYDRVGSNERIGQVIIGTCATGVALKQWNDMLATPRRSVAQWHTLVPFNDD--------
Cjaponicasnt_3            PNKNKLSVVVMECKNLKKMDVLGLSDPYVKIYLM--MGTKRLEKKKTTIKMKTLNPYYNESFSFD-VTPEKMMRVHLHVTVSDYDRVGSNERIGQVLIGTCATGIALKQWNDMLATPRRSVAQWHTLLPFNDD--------
```
