# Supplementary material for: A manual collection of Syt, Esyt, Rph3a, Rph3al, Doc2, and Dblc2 genes from 46 metazoan genomes - an open access resource for neuroscience and evolutionary biology
Source: BMC Genomics. 2010 Jan 15;11:37. doi: 10.1186/1471-2164-11-37 (PMC2823689; doi:10.1186/1471-2164-11-37)
Supplement: Additional file 10 — Alignment of the vertebrate Syt1 sequences. Amino acid position is marked every hundred amino acids approximately, at the top of each page of the alignment. Splice variants are included and highlighted with black dots where they differ. Intron position and phase is indicated with a coloured bar between amino acids. Black bars indicate phase 0 introns. Red bars indicate phase +1 introns. The five conserved acidic amino acids in each C2 domain are indicated by black arrows at the top of the alignment. Conserved N-glycosylation consensus sites are indicated by blue boxes. The conserved threonine, which can be O-glycosylated is also indicated by a blue box. X residues indicate where a portion of sequence is missing. [file 1471-2164-11-37-S10.PDF]

```
                                                                                                      100
Trubripessyt1a               MSESRREALAAAAPESATASSAVPGSNTTNIAGPGTGE----KDEAFSKLKDKFMNQLDKIPLPSWAIVSIAFVAIILVLACCFCICKKWIFKKKNKKKGKDKG-KNA
Trubripessyt1b               ------------------------------------------------------------------------------------------------------------
Tnigroviridissyt1a           MSESRREALAAAAPESATASSVAAGTNTTNVAGQGTGE----KEEAFSKLKDKFMNELNKIPLPSWAIVSIAFVAIILVLACCFCICKKWIFKKKNKKKGKDKG-KNA
Tnigroviridissyt1b           --------MTGNHHQATTTS--NHLFNLTIA-----------PEEKVFDGYDPK------KLMMSLSALGAVCTVSLCMVLSTVVCIWKKCWRKVKDKEENRKKEKESN
Gaculeatussyt1a              MTEGRREALAAAAPEPTTASSSTAGSNTTNVAGRGAGE----NDEAFSKLKDKFMNELNKIPLPSWAIVSIAFVAIILVLACCFCVCKKWIFKKKNKKKGKDKG-KNA
Gaculeatussyt1b              -------------------------------------------------------------XPPWAVAALCVVSLCVVLSFAVCVGKKCLKK-KDKEKDKKKGKDKT
Olatipessyt1a                MNESRREALAAAAPVPTTASSSMAGTNTTNGAGQGTGE----KDEAFSKVKNKFMNELNKIPLPSWAIVSIAFVAIILVLACCFCICKKWIFKKKNKKKGKDKG-KNA
Olatipessyt1b                -------------------------------------------------------------XPSWTAAALCFVSLCLVLSCVICVWKKCIKKDKDKEKEKKKGKEKS
Dreriosyt1avar1              --MSRREA-RVGNPAP-TAAPEVPG-NSTEAAGPGPRE---TKDEMFSKVKNKFMNELHKIPLPSWAIVAIAFVAVVLVVSCCFCICKKWIFKKKNKKKGKDKG-KNA
Dreriosyt1avar2              --MSRREA-RVGNPAP-TAAPEVPG-NSTEAAGPGPRE---TKDEMFSKVKNKFMNELHKIPLPSWAIVAIAFVAVVLVVSCCFCICKKWIFKKKNKKKGKDKG-KNA
Dreriosyt1b                  MHEDDAPLAASSSSPASLAQKESKIPGSTISSPTAVHPQTTTADSLVHSIRSKFLNELHKVPLPSWATAALGFITVFIVLSCCLCIFRKMIFKKKKEKGGKEKNEKND
Xtropicalissyt1var1          MKLSESRR-EVLEELQTTVAAPALPNNATETVAPGGG-----KDNHFSKLREKFMNELNKIPLPPWALIAIAIVAVLLILTCCFCVCKKCLFKKKNKKKGKEKGGKNA
Xtropicalissyt1var2          MKLSESRRPEVLEELQTTVAAPALPNNATETVAPGGG-----KDNHFSKLREKFMNELNKIPLPPWALIAIAIVAVLLILTCCFCVCKKCLFKKKNKKKGKEKGGKNA
Acarolinensissyt1var1        --MAKESQREALAAPPGTT---AIINNVTEPSIPEGGGKGGPKEDAFSKLKEKFMNELNKIPLPPWALIAIAIVAILLVLTCCFCLCKKCLFKKKNKKKGKEKGGKNA
Acarolinensissyt1var2        --MAKESQREALAAPPGTT---AIINNVTEPSIPEGGGKGGPKEDAFSKLKEKFMNELNKIPLPPWALIAIAIVAILLVLTCCFCLCKKCLFKKKNKKKGKEKGGKNA
GgallusSYT1var1              --MVSESHHEALAAPPATTVAAALPSNVTEPAAPGGGG--G-KEDAFSNLKKKFMNELNKIPLPPWALIAIAIVAVLLILTCCFCLCKKCLFKKKNKKKGKEKGGKNA
GgallusSYT1var2              --MVSESHHEALAAPPATTVAAALPSNVTEPAAPGGGG--G-KEDAFSNLKKKFMNELNKIPLPPWALIAIAIVAVLLILTCCFCLCKKCLFKKKNKKKGKEKGGKNA
TguttataSYT1                 --MVSESHHEALAAPPATTVAAALPSNVTEPASPGGGG--GGKEDAFSKLKEKFMNELNKIPLPPWALIAIAIVAVLLILTCCFCLCKKCLFKKKNKKKGKEKGGKNA
OanatinusSyt1                --MVGESHHEALAAPPATTTAAILSSNVTEPASPGEG-----KEDAFSKLKEKFMNGLNKIPLPPWALIAIAIVAVLLILTCCFCICKKCLFKKKNKKKGKEKGGKNA
MdomesticaSyt1               MVSESESHHEALAAPPVTTTAPVLPHNVTEPATPGEG-----KEDAFSKIKEKFMNELNKIPLPPWALIAIAIVAVLLILTCCFCICKKCLFKKKNKKKGKEKGGKNA
MmusculusSyt1var1            --MVSASRPEALAAP-VTTVATLVPHNATEPASPGEG-----KEDAFSKLKQKFMNELHKIPLPPWALIAIAIVAVLLVVTCCFCVCKKCLFKKKNKKKGKEKGGKNA
MmusculusSyt1var2            --MVSASRPEALAAP-VTTVATLVPHNATEPASPGEG-----KEDAFSKLKQKFMNELHKIPLPPWALIAIAIVAVLLVVTCCFCVCKKCLFKKKNKKKGKEKGGKNA
HsapiensSYT1var1             --MVSESHHEALAAPPVTTVATVLPSNATEPASPGEG-----KEDAFSKLKEKFMNELHKIPLPPWALIAIAIVAVLLVLTCCFCICKKCLFKKKNKKKGKEKGGKNA
HsapiensSYT1var2             --MVSESHHEALAAPPVTTVATVLPSNATEPASPGEG-----KEDAFSKLKEKFMNELHKIPLPPWALIAIAIVAVLLVLTCCFCICKKCLFKKKNKKKGKEKGGKNA

                                                                                                      200
Trubripessyt1a                 INMKDVIDGGKP---EALKDEED-AETGLTETEKEAEPKEDQKLGKLQYSLDYNFTENTLIVGIIQAAELPAMDMGGTSDPYVKVYLLPDKKKKFETKVHRKTLNPVF
Trubripessyt1b                 --------------------------------------XETEKMGRLHFTLDYNFTDNMLVVGILQAAELPAMDVGGSSDPYVKLYLLLDKKKKFETKVHMKNFDPNF
Tnigroviridissyt1a             INMKDVIDGAKT---EALKDEED-AETGLTETEKEAEPKEDQKLGKLQYSLDYNFTENTLIVGIIQAAELPAMDMGGTSDPYVKVYLLPDKKKKFETKVHRKTLNPVF
Tnigroviridissyt1b             KSHFDTEMDEGY--KKPPTDERN-QETVLSK----KAPKEMENLGRLHFTLDYNFTDSLLVVGILQAAELPAMDVGGSSDPYVKLYLLPDKKKKFETKVQRKTLDPNF
Gaculeatussyt1a                INMMDVKDGAKT---EALKDEEE-AETGLTETEKEEEPKREQKLGKLQYSLDYNFTENTX------------------------------------------------
Gaculeatussyt1b                SGGSDTEMDGGY--SEPLKDEGN-EKTELSD----IEPKDEGKLGRLHFTLDYNFTDNTLIVGILQGAELPAMDVGGSSDPYVKLYLLPDKKKKFETKVHRKTLEPNF
Olatipessyt1a                  INMKDVKDGAKT---EALKDEED-AETGLTETEKEAEPKESEKLGKLQYSLDYNFTENTLMVGIIQAAELPAMDMGGTSDPYVKVYLLPDKKKKFETKVHRKTLNPVF
Olatipessyt1b                  KGDFDTEMDGSY--NEPLKEEIK-KETALSE----NELKEDEKLGRLHFTLDYNFTENTLVVGVLEASELPAMDVGGSSDPYVKLYLLPDKKKRFETKVHRKTLEPTF
Dreriosyt1avar1                INMKDVKDGIKT---EALKDEDD-AETGLTDTEKEVEPKEEEKLGKLQYSMDYNFTENTLIVGIIQAAELPAMDMGGTSDPYVKVYLLPDKKKKFETKVHRKTLNPVF
Dreriosyt1avar2              ● INMKDVKDGIKT---E---DEDD-AETGLTDTEKEVEPKEEEKLGKLQYSMDYNFTENTLIVGIIQAAELPAMDMGGTSDPYVKVYLLPDKKKKFETKVHRKTLNPVF
Dreriosyt1b                    INMSNVKEEG-T-K-QAVHHTTD-SDAKEED----SESKEAQKLGKLLYTLDYNFTDSTLIVGVIRAEGLAAMDMSGTSDPYVKVYLLPDKKKKFETKVHRKTLEPTF
Xtropicalissyt1var1            INMKDVKDLGKTMKDQALKDDDDDAETGLTDGEDKEQPKEVEKLGKLQYSLDYDFQNNQLMVGIIQAAELPALDMGGTSDPYVKLFLMPDKKKKFETKVHRKTLNPVF
Xtropicalissyt1var2          ● INMKDVKDLGKTMKDQ---DDDDDAETGLTDGEDKEQPKEVEKLGKLQYSLDYDFQNNQLMVGIIQAAELPALDMGGTSDPYVKLFLMPDKKKKFETKVHRKTLNPVF
Acarolinensissyt1var1          INMKDVKDLGKNLKDQALKDDD--AETGLTDGEEKEETKEEEKLGKIQYSLDYDFTNNQLLVGIIQAAELPALDMGGTSDPYVKVFLLPDKKKKYETKVHRKTLNPVF
Acarolinensissyt1var2          INMKDVKDLGKNLKDQ---DDD--AETGLTDGEEKEETKEEEKLGKIQYSLDYDFTNNQLLVGIIQAAELPALDMGGTSDPYVKVFLLPDKKKKYETKVHRKTLNPVF
GgallusSYT1var1                INMKDVKDLGKTMKDQALKDDD--AETGLTDGEEKEEPKEVEKLGKIQYSLDYDFQNNQLLVGIIQAAELPALDMGGTSDPYVKVFLLPDKKKKYETKVHRKTLNPVF
GgallusSYT1var2              ● INMKDVKDLGKTMKDQ---DDD--AETGLTDGEEKEEPKEVEKLGKIQYSLDYDFQNNQLLVGIIQAAELPALDMGGTSDPYVKVFLLPDKKKKYETKVHRKTLNPVF
TguttataSYT1                   INMKDVKDLGKSMKDQALKDDD--AETGLTDGEEKEEAKEVEKLGKIQYSLDYDFQNNQLLVGIIQAAELPALDMGGTSDPYVKVFLLPDKKKKYETKVHRKTLNPVF
OanatinusSyt1                  INMKDVKDLGKTMKDQALKDDD--AETGLTDGEEKEEPKEEEKLGKLQYSLDYDFQNNQLMVGIIQAAELPALDMGGTSDPYVKVFLLPDKKKKFETKVHRKTLNPVF
MdomesticaSyt1                 INMKDVKDLGKTMKDQALKDDD--AETGLTDGEEKEEPKEEEKLGKLQYSLDYDFQNNQLLVGIIQAAELPALDMGGTSDPYVKVFLLPDKKKKFETKVHRKTLNPVF
MmusculusSyt1var1              INMKDVKDLGKTMKDQALKDDD--AETGLTDGEEKEEPKEEEKLGKLQYSLDYDFQNNQLLVGIIQAAELPALDMGGTSDPYVKVFLLPDKKKKFETKVHRKTLNPVF
MmusculusSyt1var2            ● INMKDVKDLGKTMKDQ---DDD--AETGLTDGEEKEEPKEEEKLGKLQYSLDYDFQNNQLLVGIIQAAELPALDMGGTSDPYVKVFLLPDKKKKFETKVHRKTLNPVF
HsapiensSYT1var1               INMKDVKDLGKTMKDQALKDDD--AETGLTDGEEKEEPKEEEKLGKLQYSLDYDFQNNQLLVGIIQAAELPALDMGGTSDPYVKVFLLPDKKKKFETKVHRKTLNPVF
HsapiensSYT1var2             ● INMKDVKDLGKTMKDQ---DDD--AETGLTDGEEKEEPKEEEKLGKLQYSLDYDFQNNQLLVGIIQAAELPALDMGGTSDPYVKVFLLPDKKKKFETKVHRKTLNPVF
```

```
                                                                                                      300
Trubripessyt1a            NEQFTFKVPYVELGGKTLVMTVYDFDRFSKHDAIGDIKVPMNKVDFSHITEEWRDLQSAEKEEQEKLGDICFSLRYVPTAGKLTVVVLEAKNLKKMDVGGLSDPYVKI
Trubripessyt1b            NETFSFKVPYTELGGRTLVMTVYDFGRFSKHDAIGAVKIPMSTADFSQSLEEWRDLQKAEKEESEKLGDVCLSLRYVPTAGKLTVMILEAKNLKKMDVGGLSDPYVKI
Tnigroviridissyt1a        NEQFTFKVPYVELGGKTLVMTVYDFDRFSKHDAIGDIKVPMNKVDFSHITEEWRDLQSAEKEEQEKLGDICFSLRYVPTAGKLTVVILEAKNLKKMDVGGLSX-----
Tnigroviridissyt1b        NESFSFKVPHTELGGRTLVMTVYDFDRFSKHDAIGAVKIPMSTVDFSRSLQDWRDLQKAEKEESEKLGDVCLSLRYVPTAGKLTVVILEAKNLKKMDVGGLSDPYVKI
Gaculeatussyt1a           ------XVPYVELGGKTLIMTVYDFDRFSKHDAIGDIKVPMNKVDFSHVTEEWRDLLSAEKEEQEKLGDICFSLRYVPTAGKLTVVILEAKNLKKMDVGGLSDPYAKI
Gaculeatussyt1b           NETFTFKVPYTELGGKTLVMTVYDFDRFSKHDAIGAVKIPMSGVDFSQSLQEWRDLQKAEKEESERLGDICLSLRYVPSAGKLTVVILEAKNLKKMDVGGLSDPYVKL
Olatipessyt1a             NEQFTFKVPYVELGGKTLVMTVYDFDRFSKHDAIGDIKVPMNKVDFSHVTEEWRDLQSAEKEEQEKLGDICFSLRYVPTAGKLTVVVLEAKNLKKMDVGGLSDPYVKI
Olatipessyt1b             NETFTFKVPYTEMGGRTLVMTVYDFDRFSKHDAIGAVKIPMRGVDFSQSMQEWRDLQKAEKEESERLGDICLSLRYVPTAGKLTVVILEAKNLKKMDVGGLSDPYVKI
Dreriosyt1avar1           NEQFTFKVPYTELGGKTLVMTVYDFDRFSKHDAIGDVKVPMNKVDFSHVTEEWRDLQSAEKEEQEKLGDICFSLRYVPTAGKLTVVVLEAKNLKKMDVGGLSDPYVKI
Dreriosyt1avar2           NEQFTFKVPYTELGGKTLVMTVYDFDRFSKHDAIGDVKVPMNKVDFSHVTEEWRDLQSAEKEEQEKLGDICFSLRYVPTAGKLTVVVLEAKNLKKMDVGGLSDPYVKI
Dreriosyt1b               NEHFTFKVPYAELGGKTLVMTVYDFDRFSKHDAIGDVRLQMNKVDFSHLTEEWRDLQKAEKEEQERLGDICLSLRYVPTAGKLTVVVLEAKNLKKMDVGGLSDPYVKI
Xtropicalissyt1var1       NEQFTFKVPYAELGGKTLVLTVYDFDRFSKHDVIGDVKVPMNTVDFGHVTEEWRDLVSAEKEEQEKLGDICFSLRYVPTAGKLTVVILEAKNLKKMDVGGLSDPYVKI
Xtropicalissyt1var2       NEQFTFKVPYAELGGKTLVLTVYDFDRFSKHDVIGDVKVPMNTVDFGHVTEEWRDLVSAEKEEQEKLGDICFSLRYVPTAGKLTVVILEAKNLKKMDVGGLSDPYVKI
Acarolinensissyt1var1     NEQFTFKVPYAELGGKTLVMAVYDFDRFSKHDIIGEYKVAMNTVDFGHVTEEWRDLQSAEKEEQEKLGDICFSLRYVPTAGKLTVVILEAKNLKKMDVGGLSDPYVKI
Acarolinensissyt1var2     NEQFTFKVPYAELGGKTLVMAVYDFDRFSKHDIIGEYKVAMNTVDFGHVTEEWRDLQSAEKEEQEKLGDICFSLRYVPTAGKLTVVILEAKNLKKMDVGGLSDPYVKI
GgallusSYT1var1           NEQFTFKVPYSELGGKTLVMAVYDFDRFSKHDIIGEYKVAMNTVDFGHVTEEWRDLQSAEKEEQEKLGDICFSLRYVPTAGKLTVVILEAKNLKKMDVGGLSDPYVKI
GgallusSYT1var2           NEQFTFKVPYSELGGKTLVMAVYDFDRFSKHDIIGEYKVAMNTVDFGHVTEEWRDLQSAEKEEQEKLGDICFSLRYVPTAGKLTVVILEAKNLKKMDVGGLSDPYVKI
TguttataSYT1              NEQFTFKVPYSELGGKTLVMAVYDFDRFSKHDIIGEYKVAMNTVDFGHVTEEWRDLQSAEKEEQEKLGDICFSLRYVPTAGKLTVVILEAKNLKKMDVGGLSDPYVKI
OanatinusSyt1             NEQFTFKVPYSELGGKTLVMAVYDFDRFSKHDIIGEFKVAMNTVDFGHVTEEWRDLQSAEKEEQEKLGDICFSLRYVPTAGKLTVVILEAKNLKKMDVGGLSDPYVKI
MdomesticaSyt1            NEQFTFKVPYSELGGKTLVMAVYDFDRFSKHDIIGEFKVAMNTVDFGHVTEEWRDLQSAEKEEQEKLGDICFSLRYVPTAGKLTVVILEAKNLKKMDVGGLSDPYVKI
MmusculusSyt1var1         NEQFTFKVPYSELGGKTLVMAVYDFDRFSKHDIIGEFKVPMNTVDFGHVTEEWRDLQSAEKEEQEKLGDICFSLRYVPTAGKLTVVILEAKNLKKMDVGGLSDPYVKI
MmusculusSyt1var2         NEQFTFKVPYSELGGKTLVMAVYDFDRFSKHDIIGEFKVPMNTVDFGHVTEEWRDLQSAEKEEQEKLGDICFSLRYVPTAGKLTVVILEAKNLKKMDVGGLSDPYVKI
HsapiensSYT1var1          NEQFTFKVPYSELGGKTLVMAVYDFDRFSKHDIIGEFKVPMNTVDFGHVTEEWRDLQSAEKEEQEKLGDICFSLRYVPTAGKLTVVILEAKNLKKMDVGGLSDPYVKI
HsapiensSYT1var2          NEQFTFKVPYSELGGKTLVMAVYDFDRFSKHDIIGEFKVPMNTVDFGHVTEEWRDLQSAEKEEQEKLGDICFSLRYVPTAGKLTVVILEAKNLKKMDVGGLSDPYVKI

                                                                                                      400
Trubripessyt1a            HLMQNGKRLKKKKTTIKKNTLNPYYNESFSFEVPFEQIQKVQVVITVLDYDKIGKNDAIGKVFVGLNSSGTELRHWSDMLANPRRPIAQWHVLKPEEEVEAQLSVKK-
Trubripessyt1b            HFMQNGKRLKKKKTTIKKNSLNPYYNESFSFEVPCEQIEKVQIAVTVLDYDKIGKNDAIGKVVLGASNTGTEQRHWSDMLANPRRPIAQWHSLRHQEEVNSLLFSKK-
Tnigroviridissyt1a        ------------------------------------------------------------------------------------------------------------
Tnigroviridissyt1b        HLMQNGKRLKKKKTTIKKNTLNPYYNESFSFEVPCDQIEKVKIAVTVLDYDKIGKNDVIGKVLLGTNSTGTEQRHWSDMLANPRRPIAQWHSLQRQEEVNSQLSNKK-
Gaculeatussyt1a           HLMQNGKRLKKKKTTIKKNTLNPYYNESFSFEVPFEQIQKVQVVITVLDYDKIGKNDAIGKVFVGLNSSGTELRHWSDMLANPRRPIAQWHVLKPEEEVDAQLSTKK-
Gaculeatussyt1b           HLMQNGKRLKKKKTTTKKNTLNPYYNESFSFEVTCEQIEKVQVGVTVLDYDKIGKNDAIGKVLLGLNGTGTEQRHWEDMLANPRRPIAQWHSLKPEDEIKALLSNKK-
Olatipessyt1a             HLMQNGKRLKKKKTTIKKNTLNPYYNESFSFEVPFEQIQKVQIVITVLDYDKIGKNDAIGKVFVGLNSSGTELRHWSDMLANPRRPIAQWHVLKPEEEVDAQLSVKK-
Olatipessyt1b             HLMQNGKRLKKKKTTIKKNTLNPYYNESFSFEVPCEQIEKVQVAVTVLDYDKIGKNDAIGKVLLGGNSSGTEQRHWSDMLANPRRPIAQWHSLKPEDEVNLLISNKK-
Dreriosyt1avar1           HLMQNGKRLKKKKTTIKKNTLNPYYNESFSFEVPFEQIQKVQVVITVLDYDKIGKNDAIGKVFVGLNSTGTELRHWSDMLANPRRPIAQWHVLKPEEEVDALLAAQKK
Dreriosyt1avar2           HLMQNGKRLKKKKTTIKKNTLNPYYNESFSFEVPFEQIQKVQVVITVLDYDKIGKNDAIGKVFVGLNSTGTELRHWSDMLANPRRPIAQWHVLKPEEEVDALLAAQKK
Dreriosyt1b               HLMQNGKRLKKKKTTIKKNTLNPYYNESFSFEVPSEQIQ----------------------------------RHWSDMLANPRRPIAQWHALKPEEDVDAELIKK--
Xtropicalissyt1var1       HLMQNGKRLKKKKTTIKKNTLNPYYNESFSFEVPFEQIQKVQVVVTVLDYDKIGKNDAIGKVFVGYNSTGAELRHWSDMLANPRRPIAQWHTLTPEEEVDAMLGVKK-
Xtropicalissyt1var2       HLMQNGKRLKKKKTTIKKNTLNPYYNESFSFEVPFEQIQKVQVVVTVLDYDKIGKNDAIGKVFVGYNSTGAELRHWSDMLANPRRPIAQWHTLTPEEEVDAMLGVKK-
Acarolinensissyt1var1     HLMQNGKRLKKKKTTIKKNTLNPYYNESFSFEVPFEQIQKVQVVVTVLDYDKIGKNDAIGKVFVGYNSTGAELRHWSDMLANPRRPIAQWHTLQQEEEVDAMLASKK-
Acarolinensissyt1var2     HLMQNGKRLKKKKTTIKKNTLNPYYNESFSFEVPFEQIQKVQVVVTVLDYDKIGKNDAIGKVFVGYNSTGAELRHWSDMLANPRRPIAQWHTLQQEEEVDAMLASKK-
GgallusSYT1var1           HLMQNGKRLKKKKTTIKKNTLNPYYNESFSFEVPFEQIQKVQIVVTVLDYDKIGKNDAIGKVFVGYNSTGAELRHWSDMLANPRRPIAQWHTLQPEEEVDAMLAVKK-
GgallusSYT1var2           HLMQNGKRLKKKKTTIKKNTLNPYYNESFSFEVPFEQIQKVQIVVTVLDYDKIGKNDAIGKVFVGYNSTGAELRHWSDMLANPRRPIAQWHTLQPEEEVDAMLAVKK-
TguttataSYT1              HLMQNGKRLKKKKTTIKKNTLNPYYNESFSFEVPFEQIQKVQIVVTVLDYDKIGKNDAIGKVFVGYNSTGAELRHWSDMLANPRRPIAQWHTLQPEEEVDAMLAVKK-
OanatinusSyt1             HLMQNGKRLKKKKTTIKKNTLNPYYNESFSFEVPFEQIQKVQVVVTVLDYDKIGKNDAIGKVFVGYNSTGAELRHWSDMLANPRRPIAQWHTLQVEEEVDAMLAVKK-
MdomesticaSyt1            HLMQNGKRLKKKKTTIKKNTLNPYYNESFSFEVPFEQIQKVQVVVTVLDYDKIGKNDAIGKVFVGYNSTGAELRHWSDMLANPRRPIAQWHTLQVEEEVDAMLAVKK-
MmusculusSyt1var1         HLMQNGKRLKKKKTTIKKNTLNPYYNESFSFEVPFEQIQKVQVVVTVLDYDKIGKNDAIGKVFVGYNSTGAELRHWSDMLANPRRPIAQWHTLQVEEEVDAMLAVKK-
MmusculusSyt1var2         HLMQNGKRLKKKKTTIKKNTLNPYYNESFSFEVPFEQIQKVQVVVTVLDYDKIGKNDAIGKVFVGYNSTGAELRHWSDMLANPRRPIAQWHTLQVEEEVDAMLAVKK-
HsapiensSYT1var1          HLMQNGKRLKKKKTTIKKNTLNPYYNESFSFEVPFEQIQKVQVVVTVLDYDKIGKNDAIGKVFVGYNSTGAELRHWSDMLANPRRPIAQWHTLQVEEEVDAMLAVKK-
HsapiensSYT1var2          HLMQNGKRLKKKKTTIKKNTLNPYYNESFSFEVPFEQIQKVQVVVTVLDYDKIGKNDAIGKVFVGYNSTGAELRHWSDMLANPRRPIAQWHTLQVEEEVDAMLAVKK-
```
