# Supplementary material for: A manual collection of Syt, Esyt, Rph3a, Rph3al, Doc2, and Dblc2 genes from 46 metazoan genomes - an open access resource for neuroscience and evolutionary biology
Source: BMC Genomics. 2010 Jan 15;11:37. doi: 10.1186/1471-2164-11-37 (PMC2823689; doi:10.1186/1471-2164-11-37)
Supplement: Additional file 11 — Alignment of the vertebrate Syt2 sequences. Amino acid position is marked every hundred amino acids approximately, at the top of each page of the alignment. The X. tropicalis splice variant is included and highlighted with a black dot where it differs. Intron position and phase is indicated with a coloured bar between amino acids. Black bars indicate phase 0 introns. Red bars indicate phase +1 introns. The five conserved acidic amino acids in each C2 domain are indicated by black arrows at the top of the alignment. X residues indicate where a portion of sequence is missing. [file 1471-2164-11-37-S11.PDF]

```
                                                                                          100
                      |         |         |         |         |         |         |         |         |         |
Trubripessyt2a        MPKWNLFKHQTTPMVAAKPTG----ASALTVTPAPVALVSAENSTE---PVNKNDAFDEIKNKFLNEIDKIPLPSWAIIAIAVVAALLIVTCCFCIVKKCCCKKKKNKK--
Trubripessyt2b        -MKFNFFK-NSQATVATEPIS--ATSVTTAPTAGVIPTTAAETPGPNNTEISKNDMFEEIKSKFLNEIDKIPLPPWALIAIAVVAALLMVTCCFCIIKKCCCKKKKNKK--
Tnigroviridissyt2a    MTKWNLFKHQTTPMVAAKPTG----ASDLTVTPAPVALVSAENSTE---SVKKNDAFDEIKNKFLNEIDKIPLPSWAIIAIAVVAALLIVTCCFCIVKKCCCKKKKNKK--
Tnigroviridissyt2b    -MKFNFFK-KSQAAAATEPVG--ASTATVAPTEGVIPTSAADTPGSNNTEISKNDMFEEIKSKFLNEIDKIPLPPWALIAIAVVAALLMVTCCFCIIKKCCCKKKKNKK--
Gaculeatussyt2a       MTKWNLFKHQTTPMVVANPTG----ASALTVTPAPVALVSTDNSTE---PVEKNDGFDEIKNKFLNEIDKIPLPPWAIIAIAVVAALLILTCCFCIIKKCCCKKKKNKK--
Gaculeatussyt2b       -MKFNLFR-KAEAVAAAEPTG--VTAATAAPTQAFVSTSVPDTSGSNNTEISKNDMFEEIKSKFLNEIDKIPLPPWALIAIAVVAALLFLTCCFCIIKKCCCKKKKNKK--
Olatipessyt2a         MKKFNVLKPQTAPMVAAKPTG----AGDSSATPAPVAVVSNDNSTE---PVNKNDAFDEIKNKFLNEIDKIPLPSWAIIAIAVVAALLILTCCFCIIKKCCCKKKKNKK--
Olatipessyt2b         -MKFNLFK-TPQAVAA-EPTS--GTTVTMATSPAVVSTTAPDISGSNNTEISKNDMFEEIKSKFLNEIDKIPLPPWALIAIAVVAALLVLTCCFCIIKKCCCKKKKNKK--
Dreriosyt2a           -MKWNVLKKKPAAMVGPEPTG----ASALTMAPA--VSTPADNSTEHQGPVSKNDVFGDIKNKFLNEMDKLPLPPWAIIAIAIVAVVLILTCCFCIVKKTCLKKKKGKK--
Dreriosyt2b           -------------MVGPEPTGPGGTHPSHTMTMAPAVSTPADNSTESEGTVNKNDVFEEIKSKFLNEIDKIPLPPWALIAIAVVAALLILTCCFCIIKKCCCKKKKNKK--
Xtropicalissyt2var1   -MHFNLFKKAEAPPP--------ETHTMPTSTEPPVVSQDNSTAVAGSGD-KSADTFRKIQQELMNQINKIPLPPWALIAIAVVAGLLIITCCFCICKKCCCKKKKGKKEK
Xtropicalissyt2var2   --------------------------------------------------------------------------------------------------------------
Acarolinensissyt2     -MKWNLFKKTANPEPIIAATTGGPAASPTTTTAPPSLSTDNTTQISGPVE-SKEDVFAKIKEKFMNEINKIPLPPWALIAIAVVAGLLILTCCFCICKKCCCKKKKNKKDK
TguttataSYT2          ----------------MTFKQASMMAPEATASTLPMSTMENSTEAAGPGE-SKEDMFTKLRDKFMNELNKIPLPPWALIAIAVVAGLLILTCCFCICKKCCCKKKKNKKEK
OanatinusSyt2         -------------MAAVP-----PGPPGAPGPV---GPADNSTETT-AGE-SKGDMFAKLKEKFFNEINKIPLPPWALIAIAVVAGLLILTCCFCICKKCCCKKKKNKKEK
MdomesticaSyt2        --MRNIFKR--NQEPIVA-----PAATTATMPVVPAGPPDNSTESGGAGP-TGEDMFTQMKDKFFNEIQKIPLPPWALIAIAVVAGLLLLTCCFCICKKCCCKKKKNKKEK
MmusculusSyt2         --MRNIFKR--NQEPIVA-----PATTTATMPI---GPVDNSTESGGAGE-SQEDMFAKLKEKLFNEINKIPLPPWALIAIAVVAGLLLLTCCFCICKKCCCKKKKNKKEK
HsapiensSYT2          --MRNIFKR--NQEPIVA-----PATTTATMPI---GPVDNSTESGGAGE-SQEDMFAKLKEKLFNEINKIPLPPWALIAIAVVAGLLLLTCCFCICKKCCCKKKKNKKEK

                                                                                                    ↓     ↓
                                                                                                            200
                      |         |         |         |         |         |         |         |         |         |
Trubripessyt2a        GKKGKGDMGMTNLKGGEKQQEDDDEEDDVEPGLTGDEKE-EEVKEKEKLGKLQYSIDYDFQENKLAVGILQAADLLSMDSGGTSDPYVKVFVLPDKKKKYDTKVHKKTLNP
Trubripessyt2b        GKKGKDGFNMKDMQGGEKLQDDDDEEG--ETGLTEEEKE-EEDKEKEKLGKLQYSIDYDFENTKLTVGILQAADLISMDSGGTSDPYVKVLLLPDKKKKYDTKVHKKTLNP
Tnigroviridissyt2a    GKKGKGDMGMKNLKGGEKQQEDDDEEDDVEPGLTGDEKE-EEVKEKEKLGKLQYSIDYDFQENKLTVGILQAADLLSMDSGGTSDPYVKVFVLPDKKKKFDTKVHKKTLNP
Tnigroviridissyt2b    GKKGKDGFNMKNMQGGEKHQDDDDEEG--ETGLTEEEKE-EEEKEKEKLGKLQYSIDYDFENTKLTVGILQAADLISMDSGGTSDPYVKVLLLPDKKKKYDTKVHKKTLNP
Gaculeatussyt2a       GKKGKGEMGMKNLKGGE---DEDDEEDDVEPGLTGDEKE-EEVKEKEKLGKLQYSIDYDFQENKLTVGILQAADLLSMDSGGTSDPYVKVFVLPDKKKKYDTKVHKKTLNP
Gaculeatussyt2b       GKKGKDGFNMKNMQGEIHQDDEDDEEG--ETGLTEEEKE-EEEKEEEKLGKLQYSIDYDFENAKLTVGILQAADLISMDSGGTSDPYVKVQLLPEKKKKYDTKVHKKTLNP
Olatipessyt2a         GKKGKGEMGMKNLKGGENQKDDDDEEEDMEPGITGDEKE-EEVKEKEKLGKLQYSIDYDFQENKLTVGILQAADLLSMDSGGTSDPYVKVFVLPDKKKKFDTKVHKKTLNP
Olatipessyt2b         GKKGKDGFNMKNMQGGE--DDDDDDEG--ETGMTEEEKE-EEEKEQEKLGKLQYSIEYDFENTKLTVGILQAADLMSMDSGGTSDPYVRVLLLPDKKKKFDTKVHKKTLNP
Dreriosyt2a           GKKGKGDMGMKNMKGGEKQEEDDDEE-DIDMGIT-EEKE-EEPKEKEKLGKLQFSLDYDFSASQLTVGILQAADLLSMDSGGTSDPYVKVFILPDKKKKYDTKVHKKTLNP
Dreriosyt2b           GKKGKDGFNMKNMQGGD---DDDDEEG--ETGLTEEEKE-EEEKEVEKLGKLQYSLDYDFQDNKLTVGILQCADLISMDSGGTSDPYVKVFILPDKKKKYDTKVHKKTLNP
Xtropicalissyt2var1   GKGMKNALNMKDMKGSGGQDDDDDAE----TGLTEGEDKEEEEKEEEKLGKLQFSLDYDFQNNQMQVGIIQATELPALDMGGTSDPYVKVFFLPDKKKKHETKVHRKTLNP
Xtropicalissyt2var2 ● -------------------------------------------------------------MQVGIIQATELPALDMGGTSDPYVKVFFLPDKKKKHETKVHRKTLNP
Acarolinensissyt2     -KGMKNAMNMKDMKG--QDDDDDDGD-----GDEEGGEE-EEEKEPENLGKLQFSLDYDFQANQLTVGILQAAELPALDMGGTSDPYVKVFLLPDKKKKYETKVQKKTLNP
TguttataSYT2          GKGMKNAMNMKDMKS--GNQDDDDAE----TGLTEGEGE-EEEKEPENLGKLQFSLDYDFQANQLTVGILQAAELPALDMGGTSDPYVKVFLLPDKKKKYETKVQKKTLNP
OanatinusSyt2         GKGMKNAMNMKDMKG--G-QDDDDAE----MGLTEGEGEGEEEKEPENLGKLQFSLDYDFQANQLTVGVLQAAELPALDMGGTSDPYVKVFLLPDKKKKYETKVHRKTLNP
MdomesticaSyt2        GKGMKNAMNMKDMKG----QDDDDAE----TGLTEGEGEGEEEKEPENLGKLQFSLDYDFQANQLTVGVLQAAELPALDMGGTSDPYVKVFLLPDKKKKYETKVHRKTLNP
MmusculusSyt2         GKGMKNAMNMKDMKG--G-QDDDDAE----TGLTEGEGEGEEEKEPENLGKLQFSLDYDFQANQLTVGVLQAAELPALDMGGTSDPYVKVFLLPDKKKKYETKVHRKTLNP
HsapiensSYT2          GKGMKNAMNMKDMKG--G-QDDDDAE----TGLTEGEGEGEEEKEPENLGKLQFSLDYDFQANQLTVGVLQAAELPALDMGGTSDPYVKVFLLPDKKKKYETKVHRKTLNP
```

```
                                                                                         300
Trubripessyt2a          VFNETFSFKIPFQEMGGKTLVMSVYDFDRFSKHDVIGEIKIPMNTLDLAKPIEEWRDLDSADQEEPEKLGDICISLRYVPTAGKLTICILEAKNLKKMDVGGLSDPYVKIN
Trubripessyt2b          VFNETFVFKVPYEELGGKTLVMSVYDYDRFSKHDVIGEVKIPMNTIDLGXPIEEWRDLESADQEEPEKLGDICISLRYVPTAGKLTVCILEAKNLKKMDACGLSDPYVKIQ
Tnigroviridissyt2a      VFNETFSFKIPFQEMGGKTLVMSVYDFDRFSKHDVIGEIKIPMNTLDLAKPIEEWRDLDSADQEEPEKLGDICISLRYVPTAGKLTICILEAKNLKKMDVGGLSDPYVKIN
Tnigroviridissyt2b      VFNETFVFKVPYEELGGKTLVMSVYDYDRFSKHDVIGEVKIPMNTIDLGRPIEEWRDLESADQEEPEKLGDICISLRYVPTAGKLTVCILEAKNLKKMDACGLSDPYVKIQ
Gaculeatussyt2a         TFHETFYFQIPFQEMGGKTLVMSVYDFDRFSKHDVIGEIKIPMNTLDLAKPIEEWKDLDSADQEEPEKLGDICISLRYVPTAGKLTICILEAKNLKKMDVGGLSDPYVKIN
Gaculeatussyt2b         VFNETFVFKVPYEELGGKILSMSVYDYDRFSKHDVIGEVKIPMNTIDLGRPIEEWKDLESADQEEPEKLGDICISLRYVPTAGKLTVCILEAKNLKKMDACGLSDPYVKIQ
Olatipessyt2a           VFNETFTFKIPFQEMGGKTLVMSVYDFDRFSKHDVIGEIKIPMNTLDLAKPIEEWRDLDSADQEEPEKLGDICISLRYVPTAGKLTICILEAKNLKKMDVGGLSDPYVKIN
Olatipessyt2b           VFNETFVFKVPYEELGGKTLCLSVYDYDRFSKHDVIGEVKLAMNTIDLGRPIEEWRDLESADQEEPEKLGDICISLRYVPTAGKLTVCILEAKNLKKMDACGLSDPYVKIQ
Dreriosyt2a             VFNETFHFKIPFTEMGGKTLVMSVYDFDRFSKHDVIGEVKIPMNTLDLAQPIEQWRDLDSAEKEEPEKLGDICISLRYVPTAGKLTICILEAKNLKKMDVGGLSDPYVKIH
Dreriosyt2b             VFNESFVFKIPYQELGGKTLVMSVYDYDRFSKHDIIGEVKLPMNTLDLGQPIEEWRDLDSAEKEEPEKLGDICISLRYVPTAGKLTVCILEAKNLKKMDVGGLSDPYVKIQ
Xtropicalissyt2var1     IFNETFTFKIPYQELGGKTLCMSIYDFDRFSKHDIIGEVKVPMNTVDLGQPIEEWRDLQSAEKEEPEKLGDICISLRYVPTAGKLTVCILEAKNLKKMDVGGLSDPYVKIH
Xtropicalissyt2var2     IFNETFTFKIPYQELGGKTLCMSIYDFDRFSKHDIIGEVKVPMNTVDLGQPIEEWRDLQSAEKEEPEKLGDICISLRYVPTAGKLTVCILEAKNLKKMDVGGLSDPYVKIH
Acarolinensissyt2       TYNETFVFKIPYQELGGKTLVMAIYDFDRFSKHDIIGEVKVPMNTVDLGQPIEEWRDLESAEKEEPEKLGDICISLRYVPTAGKLTVCILEAKNLKKMDVGGLSDPYVKIH
TguttataSYT2            AFNETFTFKVPYQELGGKTLVMAIYDFDRFSKHDIIGEVKVPMNTVDLGQPIEEWRDLQSGEKEEPEKLGDICISLRYVPTAGKLTVCILEAKNLKKMDVGGLSDPYVKIH
OanatinusSyt2           AFNETFTFKVPYQELGGKTLVMAIYDFDRFSKHDIIGEVKVPMNTVDLGQPIEEWRDLQGGEKEEPEKLGDICTSLRYVPTAGKLTVCILEAKNLKKMX-----XPYVKIH
MdomesticaSyt2          AFNETFTFKVPYQELGGKTLVMAIYDFDRFSKHDIIGEVKVPMNTVDLGQPIEEWRDLQGGEKEEPEKLGDICTSLRYVPTAGKLTVCILEAKNLKKMDVGGLSDPYVKIH
MmusculusSyt2           AFNETFTFKVPYQELGGKTLVMAIYDFDRFSKHDIIGEVKVPMNTVDLGQPIEEWRDLQGGEKEEPEKLGDICTSLRYVPTAGKLTVCILEAKNLKKMDVGGLSDPYVKIH
HsapiensSYT2            AFNETFTFKVPYQELGGKTLVMAIYDFDRFSKHDIIGEVKVPMNTVDLGQPIEEWRDLQGGEKEEPEKLGDICTSLRYVPTAGKLTVCILEAKNLKKMDVGGLSDPYVKIH
```

```
                                                                              400
Trubripessyt2a          LLQNGKRLKKKKTTVKKNTLNPYYNESFSFEIPLEQMQKIHAVITVLDYDKIGKNDAIGKILVGSKATGAGLKHWSDMLANPRRPIAQWHPLQPEEEVDAAVAAINAKK
Trubripessyt2b          LLQGGKRLKKKKTTVKKNTLNPYYNESFSFEIPLEQMQKIIVVVTVFDYDKIGKNDAIGKIFVGSKATGLGLKHWSDMLANPRRPIAQWHPLQPEEDIDGQLASLAAKK
Tnigroviridissyt2a      LLQNGKRLKKKKTTVKKNTLNPYYNESFSFEIPLEQMQKIQAVITVLDYDKIGKNDAIGKILVGSKATGAGLKHWSDMLANPRRPIAQWHPLQPEEEVDAAVAAINAKK
Tnigroviridissyt2b      LLQGGKRLKKKKTTVKKNTLNPYYNESFSFEIPLEQMQKIIVVVTVFDYDKIGKNDAIGKIFVGSKATGLGLKHWSDMLANPRRPIAQWHPLQPEEDIDGQLASLAAKK
Gaculeatussyt2a         LLQNGKRLKKKKTTVKKNTLNPYYNESFSFEIPIDQMQKILAVITVLDYDKIGKNDAIGKILVGSKSTGAGLKHWSDMLANPRRPIAQWHPLQPEEEVDAALAALNAKK
Gaculeatussyt2b         LLQGGKRLKKKKTTVKKNTLNPYYNESFSFEIPLEQMQKILVAVTVFDYDKIGKNDAIGKIFVGSKASGLGLKHWSDMLSNPRRPIAQWHPLQPEEDIDGQLASLNAKK
Olatipessyt2a           LLQNGKRLKKKKTTVKKNTLNPYYNESFSFEIPLEQMQKIQAVITVLDYDKIGKNDAIGKIWVGSKSTGAGLKHWSDMLANPRRPIAQWHPLQPEEEVDASLAALNAKK
Olatipessyt2b           LLQGGKRLKKKKTTVKKNTLNPYYNESFSFEIPLEQMQKILVAVTVFDYDKIGKNDAIGKIFVGSKATGLGLKHWSDMLANPRRPIAQWHVLQPEEDIDGQLAALFAKK
Dreriosyt2a             LLQNGKRLKKKKTTVKKNTLNPYYNESFSFEIPQDQMQKIQAVITVLDYDKIGKNDAIGKIWVGSKAQGTELRHWSDMLANPRRPIAQWHPLKPEEEVDATLAALTAKK
Dreriosyt2b             LLQNGKRLKKKKTTVKKNTLNPYYNESFSFEIPLEQMQKIIVVVTVFDYDKIGKNDAIGKIFIGSKASGTSQKHWSDMLANPRRPIAQWHPLQQEEDIDAALAALNAKK
Xtropicalissyt2var1     LMQNGKRLKKKKTTVKKKTLNPYFNESFSFEIPFEQIQKVQVVITVLDYDKLGKNEAIGKIFVGCNASGTELRHWSDMLANPRRPIAQWHPLKPEEEVDIALGSKK---
Xtropicalissyt2var2     LMQNGKRLKKKKTTVKKKTLNPYFNESFSFEIPFEQIQKVQVVITVLDYDKLGKNEAIGKIFVGCNASGTELRHWSDMLANPRRPIAQWHPLKPEEEVDIALGSKK---
Acarolinensissyt2       LLQNGKRLKKKKTTVKKKTLNPYFNESFSFEIPFEQIQKVQVVITVLDYDKLGKNEAIGKVFVGNNATGTELRHWSDMLANPRRPIAQWHSLKPEEEVDAALGKNK---
TguttataSYT2            LLQNGKRLKKKKTTVKKKTLNPYFNESFSFEIPFEQIQKVQVVITVLDYDKLGKNEAIGKIFTGLNSTGTELRHWSDMLANPRRPIAQWHSLKPEEEVDAALGKNK---
OanatinusSyt2           LMQNGKRLKKKKTTVKKKTLNPYFNESFSFEIPFEQIQKVQVVVTVLDYDKLGKNEAIGKIFVGCNATGTELRHWSDMLANPRRPIAQWHSLKPEEEVDALLGKNK---
MdomesticaSyt2          LMQNGKRLKKKKTTVKKKTLNPYFNESFSFEIPFEQIQKVQVVVTVLDYDKLGKNEAIGKIFVGCNATGTELRHWSDMLANPRRPIAQWHSLKPEEEVDALLGKNK---
MmusculusSyt2           LMQNGKRLKKKKTTVKKKTLNPYFNESFSFEIPFEQIQKVQVVVTVLDYDKLGKNEAIGKIFVGSNATGTELRHWSDMLANPRRPIAQWHSLKPEEEVDALLGKNK---
HsapiensSYT2            LMQNGKRLKKKKTTVKKKTLNPYFNESFSFEIPFEQIQKVQVVVTVLDYDKLGKNEAIGKIFVGSNATGTELRHWSDMLANPRRPIAQWHSLKPEEEVDALLGKNK---
```
