# Supplementary material for: A manual collection of Syt, Esyt, Rph3a, Rph3al, Doc2, and Dblc2 genes from 46 metazoan genomes - an open access resource for neuroscience and evolutionary biology
Source: BMC Genomics. 2010 Jan 15;11:37. doi: 10.1186/1471-2164-11-37 (PMC2823689; doi:10.1186/1471-2164-11-37)
Supplement: Additional file 12 — Alignment of the vertebrate Syt5 sequences. Amino acid position is marked every hundred amino acids approximately, at the top of each page of the alignment. Splice variants are included and highlighted with black dots where they differ. Intron position and phase is indicated with a coloured bar between amino acids. Black bars indicate phase 0 introns. Red bars indicate phase +1 introns. The five conserved acidic amino acids in each C2 domain are indicated by black arrows at the top of the alignment. X residues indicate where a portion of sequence is missing. [file 1471-2164-11-37-S12.PDF]

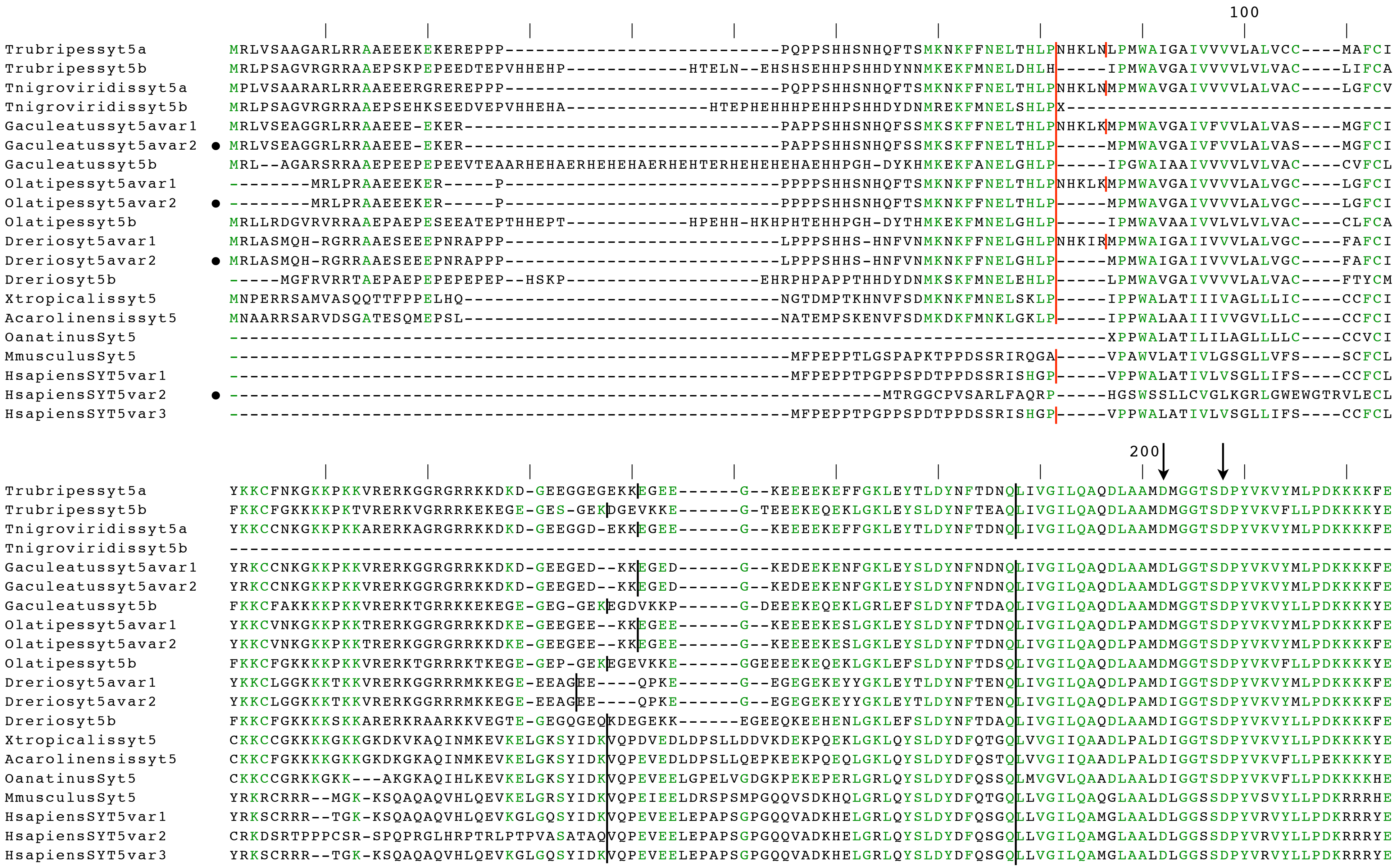
100
Trubripessyt5a
Trubripessyt5b
Tnigroviridissyt5a
Tnigroviridissyt5b
Gaculeatussyt5avar1
Gaculeatussyt5avar2
Gaculeatussyt5b
Olatipessyt5avar1
Olatipessyt5avar2
Olatipessyt5b
Dreriosyt5avar1
Dreriosyt5avar2
Dreriosyt5b
Xtropicalissyt5
Acarolinensissyt5
OanatinusSyt5
MmusculusSyt5
HsapiensSYT5var1
HsapiensSYT5var2
HsapiensSYT5var3
200
Trubripessyt5a
Trubripessyt5b
Tnigroviridissyt5a
Tnigroviridissyt5b
Gaculeatussyt5avar1
Gaculeatussyt5avar2
Gaculeatussyt5b
Olatipessyt5avar1
Olatipessyt5avar2
Olatipessyt5b
Dreriosyt5avar1
Dreriosyt5avar2
Dreriosyt5b
Xtropicalissyt5
Acarolinensissyt5
OanatinusSyt5
MmusculusSyt5
HsapiensSYT5var1
HsapiensSYT5var2
HsapiensSYT5var3

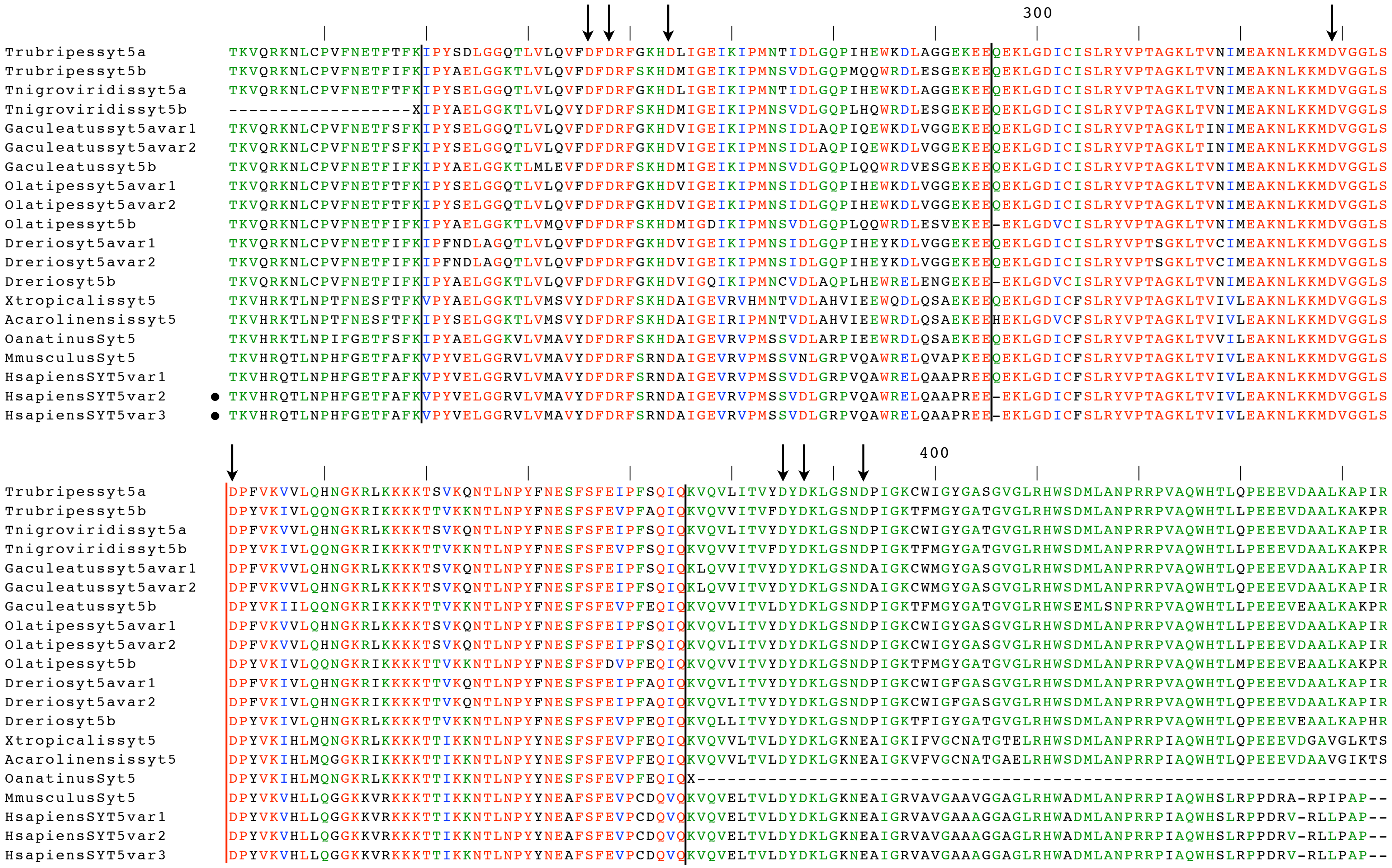
300
Trubripessyt5a
Trubripessyt5b
Tnigroviridissyt5a
Tnigroviridissyt5b
Gaculeatussyt5avar1
Gaculeatussyt5avar2
Gaculeatussyt5b
Olatipessyt5avar1
Olatipessyt5avar2
Olatipessyt5b
Dreriosyt5avar1
Dreriosyt5avar2
Dreriosyt5b
Xtropicalissyt5
Acarolinensissyt5
OanatinusSyt5
MmusculusSyt5
HsapiensSYT5var1
HsapiensSYT5var2
HsapiensSYT5var3
400
Trubripessyt5a
Trubripessyt5b
Tnigroviridissyt5a
Tnigroviridissyt5b
Gaculeatussyt5avar1
Gaculeatussyt5avar2
Gaculeatussyt5b
Olatipessyt5avar1
Olatipessyt5avar2
Olatipessyt5b
Dreriosyt5avar1
Dreriosyt5avar2
Dreriosyt5b
Xtropicalissyt5
Acarolinensissyt5
OanatinusSyt5
MmusculusSyt5
HsapiensSYT5var1
HsapiensSYT5var2
HsapiensSYT5var3
