# Supplementary material for: A manual collection of Syt, Esyt, Rph3a, Rph3al, Doc2, and Dblc2 genes from 46 metazoan genomes - an open access resource for neuroscience and evolutionary biology
Source: BMC Genomics. 2010 Jan 15;11:37. doi: 10.1186/1471-2164-11-37 (PMC2823689; doi:10.1186/1471-2164-11-37)
Supplement: Additional file 13 — Alignment of the vertebrate Syt8 sequences. Amino acid position is marked every hundred amino acids approximately, at the top of each page of the alignment. Splice variants are included and highlighted with black dots where they differ. Intron position and phase is indicated with a coloured bar between amino acids. Black bars indicate phase 0 introns. Red bars indicate phase +1 introns. X residues indicate where a portion of sequence is missing. [file 1471-2164-11-37-S13.PDF]

```
                                                                                                                      100
Trubripessyt8          -MPSTHWSPDSSSRSSAPSTSPLPWTIEPT---------TTTFTSTNTTLDPAEAAAAAAASAFINDVLDKIP------LPRWVIYSIFAVGGLLILICCLCICIKCGCRRKKK
Tnigroviridissyt8      MMAPTYLPPTSTSISSVPGPSPSPWTTEPTFVTTSISANTTLNPVAAAAAAAAAAAAAAAASGFFNDLLAKIP------LPRWAIYSIFAVGGLLIMICCLCICIKCCCKNRKK
Gaculeatussyt8         ----------MSSFHSTPNSSHPFTTALPV---------TNS--TSNSTLNPV---AAAAANLVNDLLNKIP------LPRWAIYAIFVAGALLILICCICICAKCCCKGKKK
Olatipessyt8           -------------------------------------------------------------------------------XPQWAIYTIFVSGGLLILLCLLCICIKCCCKKKQK
Dreriosyt8             ----------------------------------------MTPRPSVKPTKTPHTNTTTAAPGFLPSLLDRIP------LPRWAIYAIFAAIVLLILICVICCVKCCCKGKKK
Xtropicalissyt8        ---------------------------------------MARNSTNTTAATTIYATTTKEPESWIDSILNQIP------LPRWAIYALAGLALFIILLFIICIC-CCCCKSKKN
Acarolinensissyt8      -------------------------------------------------------------------------------XPRWALIAIVIAVGLVLLLLICII-KCCC-CRKK
GgallusSYT8            -----------------------------------MAKAQRNGQATASPPPASPVITTTGPAGFLDGFVNNIP------LPRWALIAVAVTVAVLLLFLICII-RCFC-GKKK
TguttataSYT8           -----------------------------------MAVAARKGRTTASPHTS---ITTTAWPGSLDSWLSWIP------LPKWALITVAVAGAILLLLFLVCII-KCCC-TKKK
MmusculusSyt8var1      ----------------------------------MQADRSMKMGHALNPFSTSAPLDATAGPSLIPDLITRIP------WPRWTLFIAILAAGVLLVSCLLCVI-CCYCHRHRH
MmusculusSyt8var2      ------------------------------------------------------------------------------------------------------------------
MmusculusSyt8var3 ●    ----------------------------------------MKMGHALNPFSTSAPLDATAGPSLIPDLITRIP------WPRWTLFIAILAAGVLLVSCLLCVI-CCYCHRHRH
MmusculusSyt8var4 ●    ----------------------------------------MKMGHALNPFSTSAPLDATAGPSLIPDLITRIPSCAPGLGPRWTLFIAILAAGVLLVSCLLCVI-CCYCHRHRH
MmusculusSyt8var5      ------------------------------------------------------------------------------------------------------------------
HsapiensSYT8var1       ----------------------------MLHLHGWQTMQGRKMGHPPVSPSAPAPAGTTAIPGLIPDLVAGTP------WPRWALIAGALAAGVLLVSCLLCAA-CCCCRRHR-
HsapiensSYT8var2 ●     ------------------------------------------MGHPPVSPSAPAPAGTTAIPGLIPDLVAGTP------WPRWALIAGALAAGVLLVSCLLCAA-CCCCRRHR-

                                                                                                      200
Trubripessyt8          QRQKKDDKVKLIDMDG-KSAAALVQSDVMDADYGSTNQEKRGKLLYSLEYKTVQSELIVGIKKADGLKAMDLGGSSDPYVKVYILPDKTKTCETKVFRHTLQPIFNEHFIFQI
Tnigroviridissyt8      QRQKKDDKVKLMEVNG-KSPAALAQSDAMGADYGATKQEKRGKLLYSLEYKSAQSELIVGIKKADALKAMDLGGSSDPYVKVYILPDKSKTCETKVFRHTLQPIFNEQFTFQI
Gaculeatussyt8         -KQKKNEKIDLKGVNG-KTTTALVQPDVSDVGYGSTKRP-RGKLLYSLDYNAAESVLTVGIKQADSLRAMDLGGSSDPYVKVYTCPDKSKTFETKVFRNTLSPTFNERFSFQI
Olatipessyt8           KKKQKDQ-LNLKGVNG-QTTTTLVQPDVEDVDYGSTKQH-RGKLLYSLEYNTTKSELSVGIKQANGLKSVNVGRSADPYAKVYILPDKSKTFETKVFKNTLNPTFNELFTFQL
Dreriosyt8             RKKKPDEKINMKGISG-TTTTALVQPETEDTEYSSSDQQ-RGKLQYSLEFNASRSELTVGIKEAAALKAMDSGGTSDPYVKVYILPNKSKTFETKVFRKTLNPVFNENFKYQI
Xtropicalissyt8        -KKKKDKKIDMDKVPG-NLTTHLVQPGAGNLQKG-EKVEYRGRVQYSLEYNFQTEELTVGVKQAAALKAMDLGGTSDPYAIVYVTNDTRKKFETKVNRKTLNPVFNESFVFKV
Acarolinensissyt8      -RKKKEKAVDMTSINGPSTTKRLVQPDLEDIECGNEEEEKRGRLQYSLEYDFRSQEMKVGVKQAADLKAMDSGGTSDPYVIVYLTSDIRKKYETKVYRKTLNPVFNESFIFHI
GgallusSYT8            -TKKKEK-VGLLPFSG-STTASLVQPEMEDLEQG-PQETGRGRLQYSLEYNFRAQELKVGVKQAADLKAMDNGGTSDPYVIVYLTSDMRKKYETKVYRKTLNPIFNESFTFQV
TguttataSYT8           -HKKKER-IGLCAISN-STTINLVQPEMEDLEQE-VEQKRRGKLQYSLEYNFRMQELKVGVKQAVKLRAMDSGSTSDPYVIVYLTSDIKKRYETKVYRKTLNPIFNETFTFQV
MmusculusSyt8var1      RKQPKDKETVGLGSARNSTTTHLVQPDVDCLEPCSGGDQQWGRLLLSLEYDFGSQEIRVGLRQAGNLKA---EGTADPYAWVSVSTQSGRRHETKVHRGTLSPMFEETCCFLV
MmusculusSyt8var2 ●    -------------------------------------------------------------------------------------------------------MFEETCCFLV
MmusculusSyt8var3      RKQPKDKETVGLGSARNSTTTHLVQPDVDCLEPCSGGDQQWGRLLLSLEYDFGSQEIRVGLRQAGNLKA---EGTADPYAWVSVSTQSGRRHETKVHRGTLSPMFEETCCFLV
MmusculusSyt8var4      RKQPKDKETVGLGSARNSTTTHLVQPDVDCLEPCSGGDQQWGRLLLSLEYDFGSQEIRVGLRQAGNLKA---EGTADPYAWVSVSTQSGRRHETKVHRGTLSPMFEETCCFLV
MmusculusSyt8var5      -----------------------------------------------------------------------------------------------------------------
HsapiensSYT8var1       -KKPRDKESVGLGSARGTTTTHLVQPDVDGLESSPGDAQQWGRLQLSLEFDFGSQEIRVGLRQAADLRP---GGTVDPYARVSVSTQAGHRHETKVHRGTLCPVFDETCCFHI
HsapiensSYT8var2       -KKPRDKESVGLGSARGTTTTHLVQPDVDGLESSPGDAQQWGRLQLSLEFDFGSQEIRVGLRQAADLRP---GGTVDPYARVSVSTQAGHRHETKVHRGTLCPVFDETCCFHI

                                                                                        300
Trubripessyt8          SKSTLLNSTAVMQVFDFNRFHKHNIIGELRLQLCNVDWNHVIEEWRDLAEPAKFEEE-NLGEICFSMRYVPSAGKLTVVILEARNLKSMDVGGSSDPYVKVQLALDKRKWKKK
Tnigroviridissyt8      SKSSLLNSTVVMQVFDFNRFHKHNIIGELRLQLSDVDWNHVIEEWRDX-----------------------------------------------------------------
Gaculeatussyt8         SKSSLLKSTAVMQIFDFNRFTKHEIMGEIRVDLCSVDWNRVIEEWQDLAEPAKFEEE-NLGEICFSLRYVPTTSKLTVVVLEAKDLKSMDTGGSSDPYVKVQLALDKRKWKKR
Olatipessyt8           SKSKLLKSTVVIKVFDFSRFTTHTIIGELRVQLCDINWNHVREEWGGLEEPAMFEQEVDFGEICFSLRYVPTTSKLTVVILEAKNLRSMDFGGSSDPYVKVQLALDKTKWKKR
Dreriosyt8             PQKELTESTLVMQVYDFNRFSKHDIIGEIRLNLSTVDWNHVIEEWRDLSEASKHEQE-HLGEICFSLRYVPTSSKLTVIILEAKNLKKMDQVGSSDPYVKVQLILEKKKWKKK
Xtropicalissyt8        TQEEVPRTTAVVQIFDFNRFLKHDVIGEMVIPLGEVNLQHVIEDWKDLGPAGKTEHE-HLGDICFSLRYVPSSGKLTIIILEAKNLKRMDSDGFSDPYVKVHLALNRKKWKRK
Acarolinensissyt8      PQGDVAENTLVMQVYDFNRFSKHDIIGEMRLPLGDVDLQHVMEQWHELTAASKIEQE-HLGEICISLRYVPSTSKLTVVILEAKKLKRMDPNGLSDPYVKVQLILNKKKWKKK
GgallusSYT8            PPAEVSEATLVMQIYDFNRFAKHDIIGEVRLPLASVSLQHIIEQWSDLVVASKVEQE-QLGEICFSLRYVPSTGKLTVLILEAKKLKRMDSHGLSDPFVKVHLILNKRKWKKK
TguttataSYT8           PQAEVSKCTLVMQIYDFNRFSKHDIIGEVWLPLASVNLQHVIEQWSDLAVANKVEEE-HLGEICFSLCYVPSTGKLTVLILEAKQLKRMDSDGLSDPFVKVHLILNRKKWKKK
MmusculusSyt8var1      PPAELPKATLKVQLWDFKRFSEHEPLGELQLPLGTVDLQHVLESWYQLGPPGTTEPE-QMGELCFSLRYVPSSGSLTVVVLEARGLNP----GLAEAYVKIQLMLNQRKWKKS
MmusculusSyt8var2      PPAELPKATLKVQLWDFKRFSEHEPLGELQLPLGTVDLQHVLESWYQLGPPGTTEPE-QMGELCFSLRYVPSSGSLTVVVLEARGLNP----GLAEAYVKIQLMLNQRKWKKS
MmusculusSyt8var3      PPAELPKATLKVQLWDFKRFSEHEPLGELQLPLGTVDLQHVLESWYQLGPPGTTEPE-QMGELCFSLRYVPSSGSLTVVVLEARGLNP----GLAEAYVKIQLMLNQRKWKKS
MmusculusSyt8var4      PPAELPKATLKVQLWDFKRFSEHEPLGELQLPLGTVDLQHVLESWYQLGPPGTTEPE-QMGELCFSLRYVPSSGSLTVVVLEARGLNP----GLAEAYVKIQLMLNQRKWKKS
MmusculusSyt8var5 ●    -------------------------------------------------------------------------------------------------------MLNQRKWKKS
HsapiensSYT8var1       PQAELPGATLQVQLFNFKRFSGHEPLGELRLPLGTVDLQHVLEHWYLLGPPAATQPE-QVGELCFSLRYVPSSGRLTVVVLEARGLRP----GLAEPYVKVQLMLNQRKWKKR
HsapiensSYT8var2       PQAELPGATLQVQLFNFKRFSGHEPLGELRLPLGTVDLQHVLEHWYLLGPPAATQPE-QVGELCFSLRYVPSSGRLTVVVLEARGLRP----GLAEPYVKVQLMLNQRKWKKR

                                                                              400
Trubripessyt8          KTCIKKKTLNPYYNESFVFDVSFDQIQRVNLVISVWDHDAVTRNDAIGKIFLGCDAAGNQLRHWADMLSNPRRPVAQWHSLLSSQQINSSLTLKK--KIPLASKLPL-----
Tnigroviridissyt8      --------------------------------XIAVWDHDAVTRNDAIGKIFLGCDAAGNQLRHWADMLSNPRRPVAQWHSLLSSQQINSSLTLKK--KIPLASKLPL-----
Gaculeatussyt8         KTSIKKKTLNPYYNESFTFDVSFDQIQRVNLVISVWDHDTVSRNDPIGKLFLGGDASGNQLRHWADMLANPRRPVAQWHSLLSAQQVNATLSLKR--KLPLHNKLPF-----
Olatipessyt8           KTSVKKKTLNPYFNESFTFNVMFDQIKRVNLVISVWDYDAMTRNDAIGKVFLGCDASGNQLKHWADMLSNPRRPVVQWHSLLSAEQVNSTLGLKK--KTPLSNAQLFDKYIK
Dreriosyt8             KTSVKKRTLNPYFNESFTFDVSFEQIQKVQLVISVWDHDKMSRNDAIGKIYLGCDATGNQLRHWADMLSNPRKPVAQWHTLLSAEQVDTTLALKHTLKIPFTNKNF------
Xtropicalissyt8        KTAVKKSTLKPYFNESFTFDVSLEQMKNLDLIISVWDHDKVGKNEQIGKLFLGCRASGNALRHWSDMLAHPRRPIAQWHKLQEADEVDKVLELKRNL--------------
Acarolinensissyt8      KTSVKKNTLSPYFNEAFIFDVPFHLIQNIDLVISVWDSDKVTKDDQIGKLFLGCRATGNQLRHWSDMLANARRPVAQWHNLQPVEDIDKTLGLKTRFKLPLPGS--------
GgallusSYT8            KTSVKKNTLSPYFNEAFVFEVPFNQIQNVDVVISVWDYDKMTKNEPIGKLFLGCRATGNQLRHWSDMLSNPRRPLAQWHSLQPPDVVDKALGLKSHIKLPMTTR--------
TguttataSYT8           RTSVKKNTLSPYFNEVFVFEMPFSQIQNVDMVISVWDHDKVTKNEPTGKLFLGCRATGSQLRHWSDTLSNPRRPLAQWHILQPPGMVVKALGLKSHLKLPLHSR--------
MmusculusSyt8var1      KTSSKKGTTTPYFNEAFVFLVPVSQLQSVDLVLAVWARGLQLRTEPVGKVLLGSRASGQPLQHWADMLAHARRPIAQWHHLRSPREVDRVLALQPRLPLLRPRS--------
MmusculusSyt8var2      KTSSKKGTTTPYFNEAFVFLVPVSQLQSVDLVLAVWARGLQLRTEPVGKVLLGSRASGQPLQHWADMLAHARRPIAQWHHLRSPREVDRVLALQPRLPLLRPRS--------
MmusculusSyt8var3      KTSSKKGTTTPYFNEAFVFLVPVSQLQSVDLVLAVWARGLQLRTEPVGKVLLGSRASGQPLQHWADMLAHARRPIAQWHHLRSPREVDRVLALQPRLPLLRPRS--------
MmusculusSyt8var4      KTSSKKGTTTPYFNEAFVFLVPVSQLQSVDLVLAVWARGLQLRTEPVGKVLLGSRASGQPLQHWADMLAHARRPIAQWHHLRSPREVDRVLALQPRLPLLRPRS--------
MmusculusSyt8var5      KTSSKKGTTTPYFNEAFVFLVPVSQLQSVDLVLAVWARGLQLRTEPVGKVLLGSRASGQPLQHWADMLAHARRPIAQWHHLRSPREVDRVLALQPRLPLLRPRS--------
HsapiensSYT8var1       KTATKKGMAAPYFNEAFTFLVPFSQVQNVDLVLAVWDRSLPLRTEPVGKVHLGARASGQPLQHWADMLAHARRPIAQRHPLRPAREVDRMLALQPRLRLRLPLPHS------
HsapiensSYT8var2       KTATKKGMAAPYFNEAFTFLVPFSQVQNVDLVLAVWDRSLPLRTEPVGKVHLGARASGQPLQHWADMLAHARRPIAQRHPLRPAREVDRMLALQPRLRLRLPLPHS------
```
