# Supplementary material for: A manual collection of Syt, Esyt, Rph3a, Rph3al, Doc2, and Dblc2 genes from 46 metazoan genomes - an open access resource for neuroscience and evolutionary biology
Source: BMC Genomics. 2010 Jan 15;11:37. doi: 10.1186/1471-2164-11-37 (PMC2823689; doi:10.1186/1471-2164-11-37)
Supplement: Additional file 14 — Alignment of the invertebrate Syt4 sequences. Amino acid position is marked every hundred amino acids approximately, at the top of each page of the alignment. Splice variants are included and highlighted with black dots where they differ. Intron position and phase is indicated with a coloured bar between amino acids. Black bars indicate phase 0 introns. Red bars indicate phase +1 introns. Blue bars indicate phase +2 introns. X residues indicate where a portion of sequence is missing. [file 1471-2164-11-37-S14.PDF]

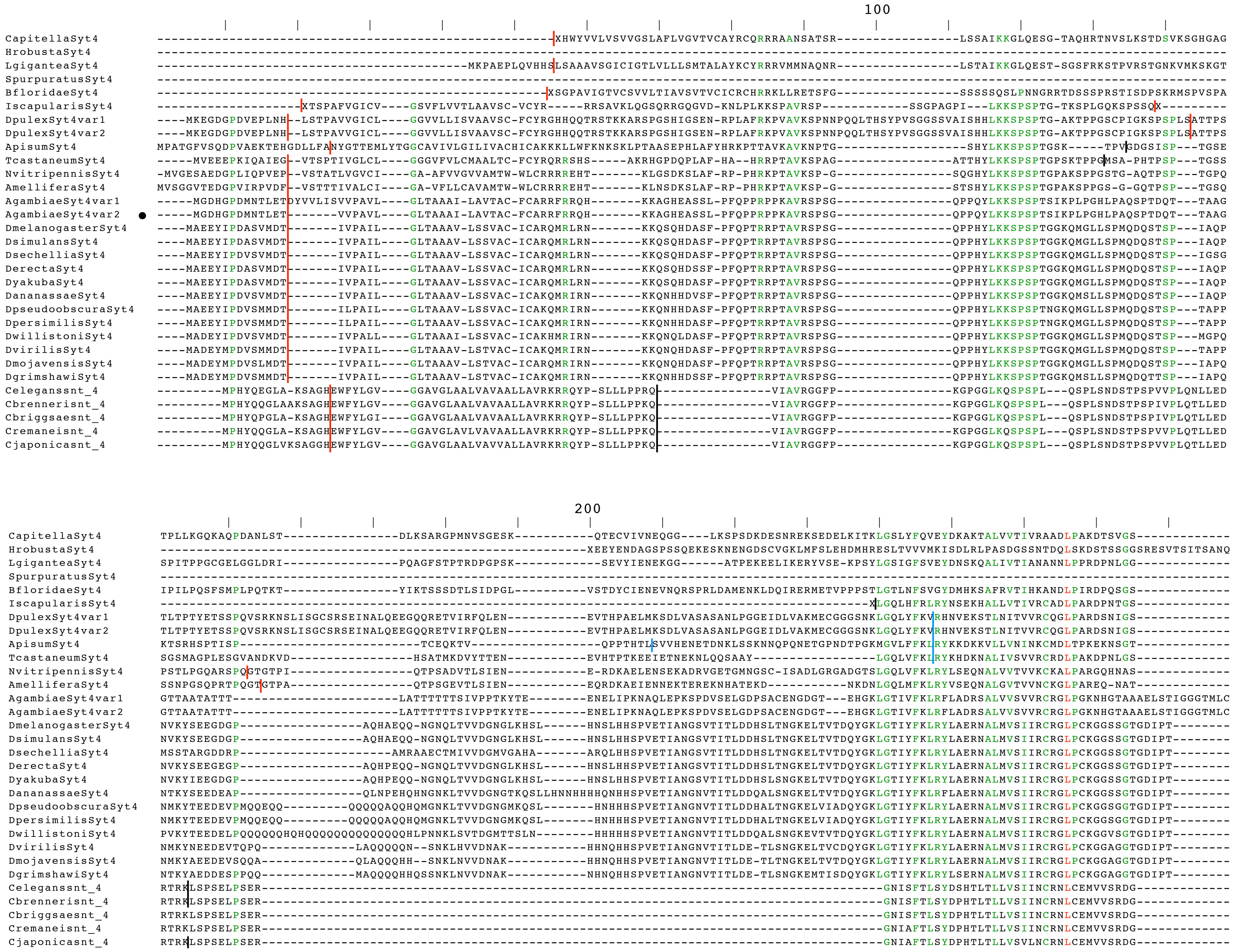
100
CapitellaSyt4
HrobustaSyt4
LgiganteaSyt4
SpurpuratusSyt4
BfloridaeSyt4
IscapularisSyt4
DpulexSyt4var1
DpulexSyt4var2
ApisumSyt4
TcastaneumSyt4
NvitripennisSyt4
AmelliferaSyt4
AgambiaeSyt4var1
AgambiaeSyt4var2
DmelanogasterSyt4
DsimulansSyt4
DsechelliaSyt4
DerectaSyt4
DyakubaSyt4
DananassaeSyt4
DpseudoobscuraSyt4
DpersimilisSyt4
DwillistoniSyt4
DvirilisSyt4
DmojavensisSyt4
DgrimshawiSyt4
Celeganssnt_4
Cbrennerisnt_4
Cbriggsaesnt_4
Cremaneisnt_4
Cjaponicasnt_4
200

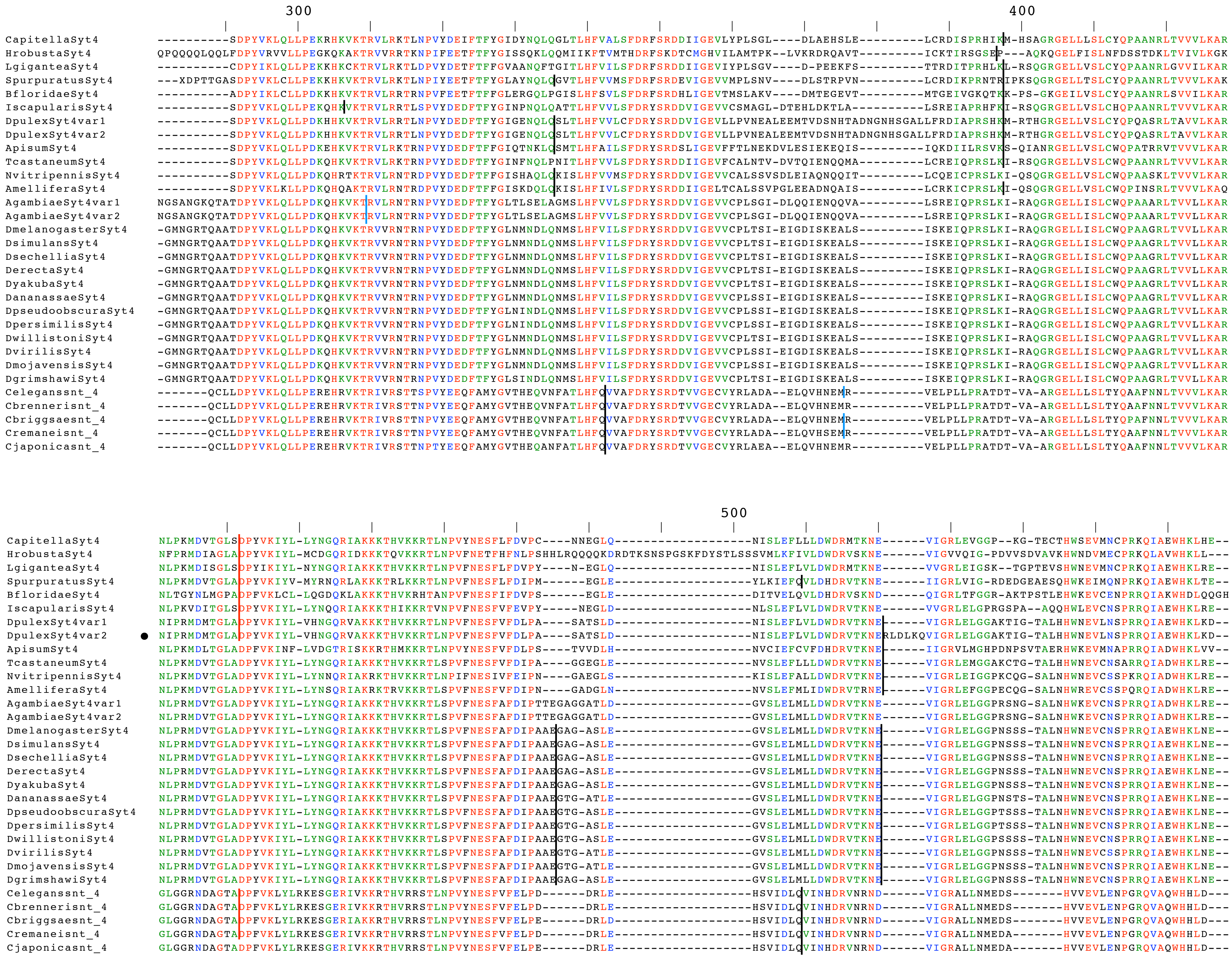

300
400
CapitellaSyt4
HrobustaSyt4
LgiganteaSyt4
SpurpuratusSyt4
BfloridaeSyt4
IscapularisSyt4
DpulexSyt4var1
DpulexSyt4var2
ApisumSyt4
TcastaneumSyt4
NvitripennisSyt4
AmelliferaSyt4
AgambiaeSyt4var1
AgambiaeSyt4var2
DmelanogasterSyt4
DsimulansSyt4
DsechelliaSyt4
DerectaSyt4
DyakubaSyt4
DananassaeSyt4
DpseudoobscuraSyt4
DpersimilisSyt4
DwillistoniSyt4
DvirilisSyt4
DmojavensisSyt4
DgrimshawiSyt4
Celeganssnt_4
Cbrennerisnt_4
Cbriggsaesnt_4
Cremaneisnt_4
Cjaponicasnt_4
500
CapitellaSyt4
HrobustaSyt4
LgiganteaSyt4
SpurpuratusSyt4
BfloridaeSyt4
IscapularisSyt4
DpulexSyt4var1
DpulexSyt4var2
ApisumSyt4
TcastaneumSyt4
NvitripennisSyt4
AmelliferaSyt4
AgambiaeSyt4var1
AgambiaeSyt4var2
DmelanogasterSyt4
DsimulansSyt4
DsechelliaSyt4
DerectaSyt4
DyakubaSyt4
DananassaeSyt4
DpseudoobscuraSyt4
DpersimilisSyt4
DwillistoniSyt4
DvirilisSyt4
DmojavensisSyt4
DgrimshawiSyt4
Celeganssnt_4
Cbrennerisnt_4
Cbriggsaesnt_4
Cremaneisnt_4
Cjaponicasnt_4
