# Supplementary material for: A manual collection of Syt, Esyt, Rph3a, Rph3al, Doc2, and Dblc2 genes from 46 metazoan genomes - an open access resource for neuroscience and evolutionary biology
Source: BMC Genomics. 2010 Jan 15;11:37. doi: 10.1186/1471-2164-11-37 (PMC2823689; doi:10.1186/1471-2164-11-37)
Supplement: Additional file 17 — Alignment of the invertebrate Syt7 sequences. Amino acid position is marked every hundred amino acids approximately, at the top of each page of the alignment. The D. melanogaster splice variant is included and highlighted with a black dot where it differs. Intron position and phase is indicated with a coloured bar between amino acids. Black bars indicate phase 0 introns. Red bars indicate phase +1 introns. Blue bars indicate phase +2 introns. The five conserved acidic amino acids in each C2 domain are indicated by black arrows at the top of the alignment. X residues indicate where a portion of sequence is missing. [file 1471-2164-11-37-S17.PDF]

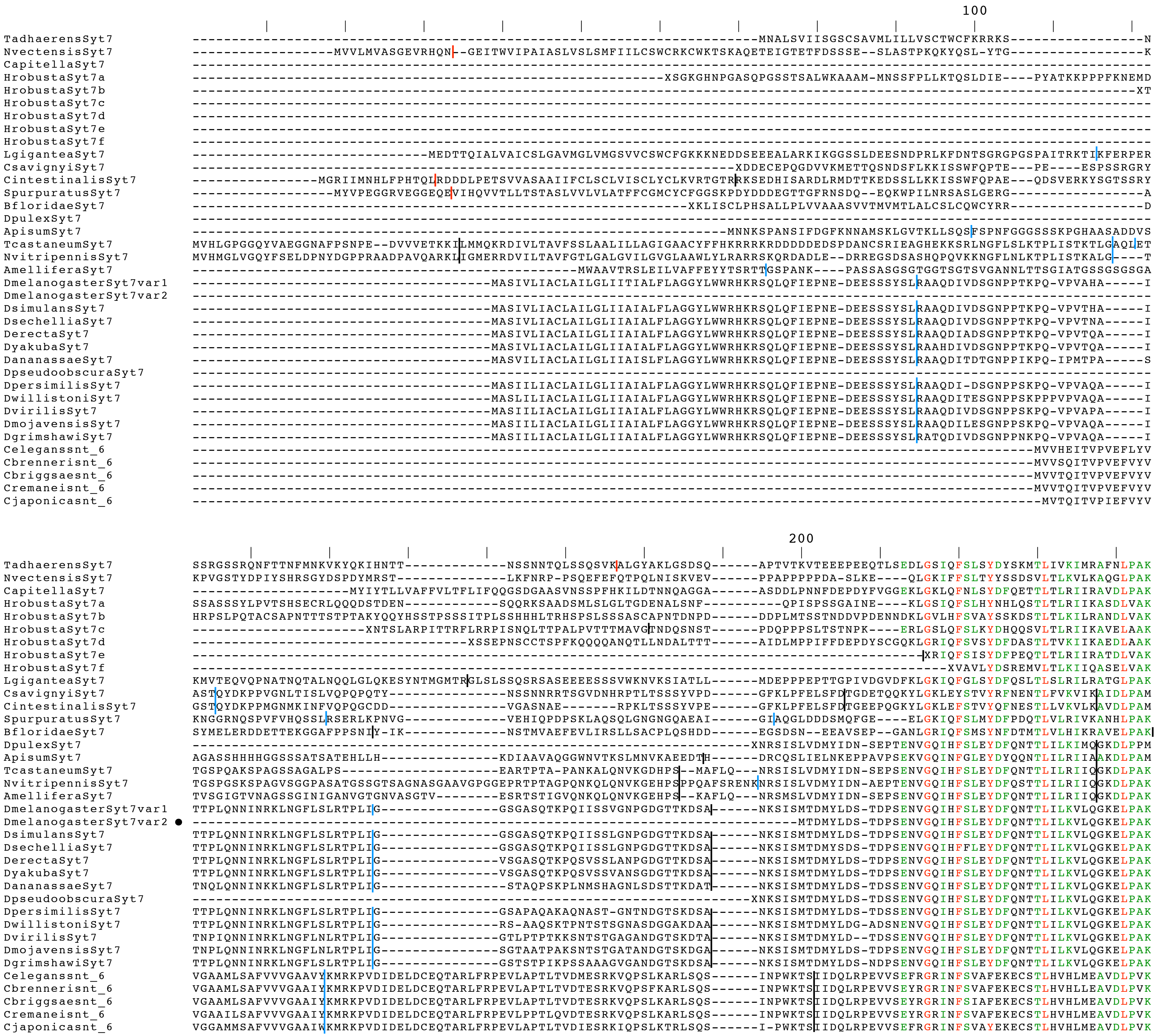
100
TadhaerensSyt7
NvectensisSyt7
CapitellaSyt7
HrobustaSyt7a
HrobustaSyt7b
HrobustaSyt7c
HrobustaSyt7d
HrobustaSyt7e
HrobustaSyt7f
LgiganteaSyt7
CsavignyiSyt7
CintestinalisSyt7
SpurpuratusSyt7
BfloridaeSyt7
DpulexSyt7
ApisumSyt7
TcastaneumSyt7
NvitripennisSyt7
AmelliferaSyt7
DmelanogasterSyt7var1
DmelanogasterSyt7var2
DsimulansSyt7
DsechelliaSyt7
DerectaSyt7
DyakubaSyt7
DananassaeSyt7
DpseudoobscuraSyt7
DpersimilisSyt7
DwillistoniSyt7
DvirilisSyt7
DmojavensisSyt7
DgrimshawiSyt7
Celeganssnt_6
Cbrennerisnt_6
Cbriggsaesnt_6
Cremaneisnt_6
Cjaponicasnt_6
200

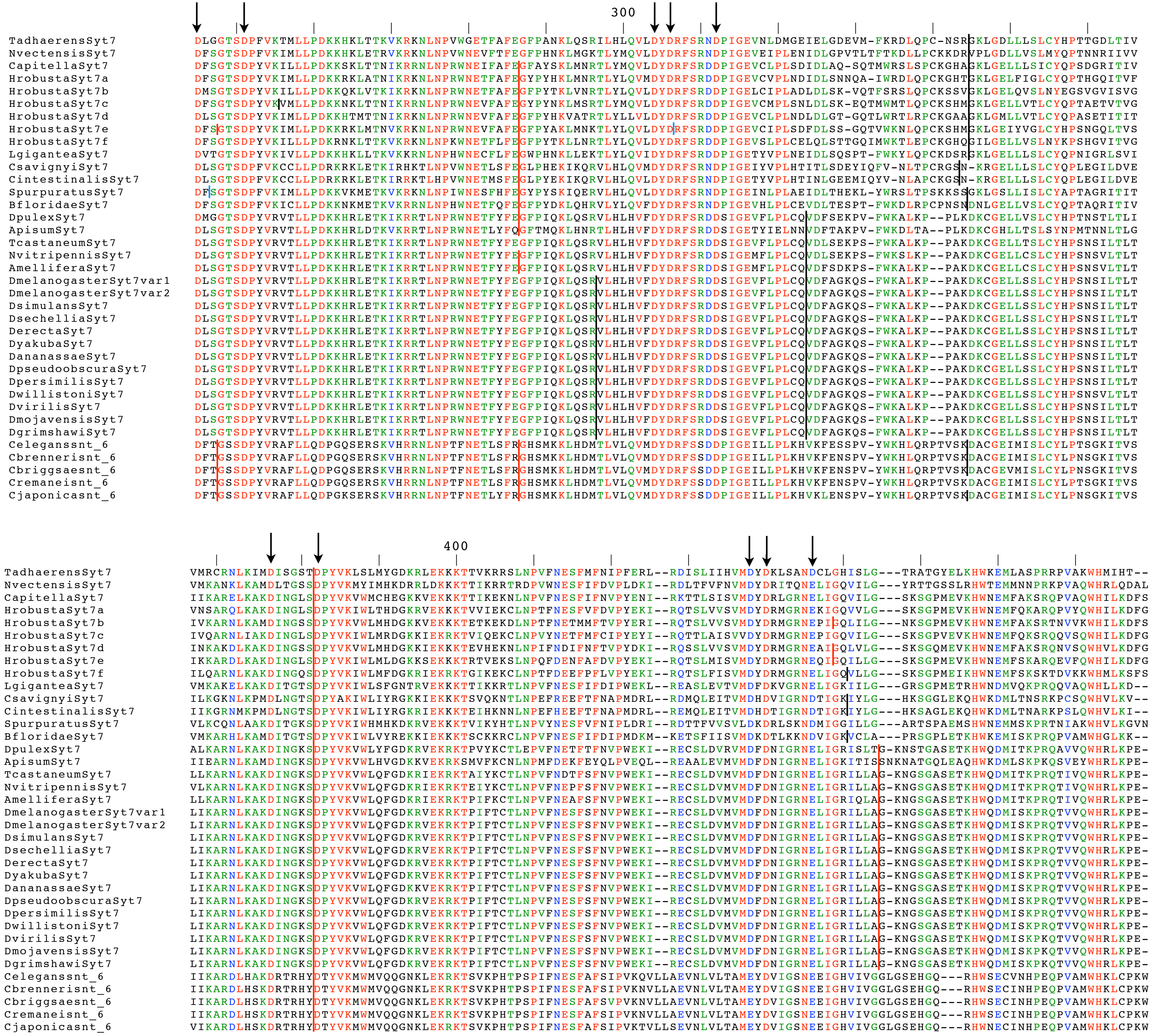
300
400
TadhaerensSyt7
NvectensisSyt7
CapitellaSyt7
HrobustaSyt7a
HrobustaSyt7b
HrobustaSyt7c
HrobustaSyt7d
HrobustaSyt7e
HrobustaSyt7f
LgiganteaSyt7
CsavignyiSyt7
CintestinalisSyt7
SpurpuratusSyt7
BfloridaeSyt7
DpulexSyt7
ApisumSyt7
TcastaneumSyt7
NvitripennisSyt7
AmelliferaSyt7
DmelanogasterSyt7var1
DmelanogasterSyt7var2
DsimulansSyt7
DsechelliaSyt7
DerectaSyt7
DyakubaSyt7
DananassaeSyt7
DpseudoobscuraSyt7
DpersimilisSyt7
DwillistoniSyt7
DvirilisSyt7
DmojavensisSyt7
DgrimshawiSyt7
Celeganssnt_6
Cbrennerisnt_6
Cbriggsaesnt_6
Cremaneisnt_6
Cjaponicasnt_6
