# Supplementary material for: A manual collection of Syt, Esyt, Rph3a, Rph3al, Doc2, and Dblc2 genes from 46 metazoan genomes - an open access resource for neuroscience and evolutionary biology
Source: BMC Genomics. 2010 Jan 15;11:37. doi: 10.1186/1471-2164-11-37 (PMC2823689; doi:10.1186/1471-2164-11-37)
Supplement: Additional file 18 — Alignment of the vertebrate Syt7 sequences. Amino acid position is marked every hundred amino acids approximately, at the top of each page of the alignment. Splice variants are included and highlighted with black dots where they differ. Intron position and phase is indicated with a coloured bar between amino acids. Black bars indicate phase 0 introns. Red bars indicate phase +1 introns. Blue bars indicate phase +2 introns. The five conserved acidic amino acids in each C2 domain are indicated by black arrows at the top of the alignment. X residues indicate where a portion of sequence is missing. [file 1471-2164-11-37-S18.PDF]

```
                                                                                                                     100
Trubripessyt7a               XDASVLICP-GLLCLCAGSVSLSVFLVSLAVTVCAVWLVALCGVCGWCQRKLGKRNKPGVEAADTPDSA----RARGEKKAIN--DLDRDFWNNNDSSTVQQRWSSYPPKEFVLNISPYAPYGD
Trubripessyt7b               -----------------XSVSLSVFLVSVASVVCAVWLVALCGVCTWCQRKMGKRNKPGVEAASSPDSV----RVRGENKAIN--DLDRDFWNNNDSSNVQQRWSSYPPKEFLLNMSPYAPYGD
Tnigroviridissyt7a           XIGGALICSLSCLCLCTGSVSLSVFLVSLAVTVCAVWLVALCGVCGWCQRKLGKRNKPGVEAADTPDSA----RARGEKKAIN--DLDRDFWNNNDSSTVQQRWSSYPPKEFVLNISPYAPYGD
Tnigroviridissyt7b           -----------------XSVSLSVFLVSVASVVCAVWLVALCGVCTWCQRKMGKRNKPGVEAASSPDSV----RGRGENKAIN--DLDRDFWNNNDSSNVQQRWSSYPPKEFLLNMSPYAPYGD
Gaculeatussyt7a              -----------------XSVSLSVFLVSLAVTVCAVWLVALCGVCGWCQRKLGKRNKPGVETADTPDSA----RGRGEKKAIN--DLDRDFWNNNDSSTVQQRWSSYPPKEFVLNISPYAPYGD
Gaculeatussyt7b              -----------------XSVSLSVFLVSVLSVICAVWLVALCGVCTWCQRKLGKRNKPGVEAASSPDSV----RGRGENKAINHNDLDRDFWNNNDSSNVQQRWTSYPPKEFLLNMSPYAPYGD
Olatipessyt7a                -----------------XSVSLSVFLVSLAVTVCAVWLVALCGVCGWCQRKLGKRNKPGVEAADTPDSA----RGRGEKKAIN--DLDRDFWNNNDSSTVQQRWSSYPPKEFVLNISPYAPYGD
Olatipessyt7b                -----------------XSESLSVFLLSVAFVICTIWLVALCGVCTWCQRKLGMRNKPGVEATGSPDSV----RGRGENKAIN--DLDRDFWNNNDSSNVQQRWSSYPPKEFLLNMSPYAPYGD
Dreriosyt7a                  -----------------XSISLSVLLVSLAVTVCGVWLVALCGVCGWCQRKLGKRNKPGVESVGSPDSG----RGRGEKKAIN--DLDRDFWNNNDSSSVQQRWSSYPPKEFVLNISPYAPYGD
Dreriosyt7b                  ------MHLNREDEDGKGSLSLSVFLVSVGLTVCVVWLVALCGVCGWCQRKLGMRNKPGVETAGTPDSVSVSGRGRGENKAINHNDLDRDFWNNNDSCSVQQRWSSYPPKEFLLNMSPYAPYGD
Xtropicalissyt7var1          ------MHINQEASNTKGAPTRDVILVSTVLTLSLSITIIMCGLCQWCQRKLGKRYKNSLETAGTPDSTR---GIRSEKKTLN--DLDKDFWNNNDS-TAHQKWSSYPPKEFILNISPYAPYGD
Xtropicalissyt7var2        ● ------MHINQEASNTKGAPTRDVILVSTVLTLSLSITIIMCGLCQWCQRKLGKRYKNSLETAGTPDSTR---GIRSEKKTL------------------------------------------
Acarolinensissyt7            -----------------XAPSRDVLLVSAIITISLSVTIVLCGICQWCQRKLGKRYKNSLETVGTPDSSR---G-RSEKKAIN--DLDRDFWNNNDN-TVQQKWSSYPPKEFILNISPYAPYGD
GgallusSYT7                  ---------------------------------------------------XGKRYKTSLETVGTPDSSR---G-RSEKKTIK-----RDFWNNNDN-TVQQKWSSYPPKEFILNISPYAPYGD
TguttataSYT7                 -----------------XTPSRDVLLVSAIITVSLSVTIVLCGICQWCQRKMGKRYKTSLETVGTPDSSR---G-RSEKKTIN--DLDRDFWNNNDN-TVQQKWSSYPPKEFILNISPYAPYGD
OanatinusSyt7                -----------------XAPSRDVLLVSAIITVSLSVTIVLCGICQWCQRKLX-----------------------------X--DLDRDFWNNNES-TVQQKWSSYPPKEFILNISPYAPYGD
MdomesticaSyt7               ----------------------------------------------------------------------------------------------------------------------------
MmusculusSyt7var1            ------MYRDPEAASP-GAPTRDVLLVSAIITVSLSVTIVLCGLCHWCQRKLGKRYKNSLETVGTPDSGR---G-RGEKKAIN--DLDRDFWNNNES-TVQQKWSSYPPKEFILNISPYAPYGD
MmusculusSyt7var2            ------MYRDPEAASP-GAPTRDVLLVSAIITVSLSVTIVLCGLCHWCQRKLGKRYKNSLETVGTPDSG----RGRGEKKAIN--DLDRDFWNNNES-TVQQKWSSYPPKEFILNISPYAPYGD
MmusculusSyt7var3            ------MYRDPEAASP-GAPTRDVLLVSAIITVSLSVTIVLCGLCHWCQRKLGKRYKNSLETVGTPDSGR---G-RGEKKAIN--DLDRDFWNNNES-TVQQKWSSYPPKEFILNISPYAPYGD
MmusculusSyt7var4          ● ------MYRDPEAASP-GAPTRDVLLVSAIITVSLSVTIVLCGLCHWCQRKLGKRYKNSLETVGTPDSG----RGRGEKKAI------------------------------------------
HsapiensSYT7var1             ------MYRDPEAASP-GAPSRDVLLVSAIITVSLSVTVVLCGLCHWCQRKLGKRYKNSLETVGTPDSGR---G-RSEKKAIN--DLDRDFWNNNES-TVQQKWSSYPPKEFILNISPYAPYGD
HsapiensSYT7var2             ------MYRDPEAASP-GAPSRDVLLVSAIITVSLSVTVVLCGLCHWCQRKLGKRYKNSLETVGTPDSGR---G-RSEKKAIN--DLDRDFWNNNES-TVQQKWSSYPPKEFILNISPYAPYGD
HsapiensSYT7var3             ------MYRDPEAASP-GAPSRDVLLVSAIITVSLSVTVVLCGLCHWCQRKLGKRYKNSLETVGTPDSG----RGRSEKKAIN--DLDRDFWNNNES-TVQQKWSSYPPKEFILNISPYAPYGD
HsapiensSYT7var4           ● ------MYRDPEAASP-GAPSRDVLLVSAIITVSLSVTVVLCGLCHWCQRKLGKRYKNSLETVGTPDSG----RGRSEKKAI------------------------------------------

                                                                                               200
Trubripessyt7a               PRLTLNGAVSGSQKGAAT---G---------------HGDNGGG--------PYHSDSMKSMVT--------EGAKVGRWTTVQGHVQSGRLRPSDFGDPVLSYASTLEHIPSGPA-----RPR
Trubripessyt7b               PRLTPNGAADKGGQGGAGPSPG---------------ASDGGGGACSGAGAGPSRSDSVRSMVT--------GGSKAGRWQTIQSHMHAGGLRFSNFGDASLSSASTLEHIPSSAVA----RPR
Tnigroviridissyt7a           PRLTLNGAVSGSQKGAAT---G---------------HGDNGGG--------PYRSDSMKSMVT--------EGAKAGRWQTVQGHVQSGRLRPSDFGDPVLSYASTLEHISSGPA-----RPR
Tnigroviridissyt7b           PRLTPNGAADKGGQGGAGPLPG---------------ASDSGGGACSGAGEGPSRSDSVRSMVT--------GGSKAGRWQTVQSHMHAGGLRFTNFGDASLSSASTLEHIPSSAVA----RPR
Gaculeatussyt7a              PRLTLNGAVSGGQKGAAT---G---------------HGDDGGV--------PYRSDSVKSMVT--------EGVKAGRWQTVQGHMQSGGLRPSDFGDPVLSYTSTLEHI---PT-----RPR
Gaculeatussyt7b              PRLTPNGAVDKGGQGGAGPSPG---------------ASDSGGGACSGAG--PSRSDSVRSMLM--------GGGKAGRWQTVQSHMQAGGLRFGNFGDASLSSASTLEHIPSSAVA----RPR
Olatipessyt7a                PRLTLNGAVSGGQKGAAT---G---------------HGDAGGA--------PYRSDSVKSMVT--------EGVKAGKWQTVQGHVQSGGLRPSNFGDPVLSYTSTLEHIHSGPT-----RPR
Olatipessyt7b                PRLTPNGAVDKGGQGGAGPSPG---------------ASDSGGGACSGTGAGPSRSDSVRSMVS--------GGSKAGRWQTVQSHMHAGGLRFGNFGDASLSSASTLEHIPSSAVA----RPR
Dreriosyt7a                  PRLTLNGTVHGGQKGAAG---C---------------VLGDGGG--------PSRSDSVRSMMT--------GGIKAGRWQTVQSRLHSGDFKNGNLSDHTLSSVETLDYVPSAGATP---RPR
Dreriosyt7b                  PRLTPNGSLEKGEAGG----------------------GAVGG---------PSRSDSC--IGA--------GTSRSGRWQTLQSHLHSGSLRPSNFGDPSLSTTSTLEHIPSSAA-----RPR
Xtropicalissyt7var1          PRLALNGTVLSSSKSAACGTSG-------------LL-DKDGRLGDKQFR---SDSMKISMSSQSHHSEPVLGKSGRGRWHTVQSHLASGNLSRTNFEDPTLTAATTLDHIPISAGDMKCQRPR
Xtropicalissyt7var2        ● -----NGTVLSSSKSAACGTSG-------------LL-DKDGRLGDKQFR---SDSMKISMSSQSHHSEPVLGKSGRGRWHTVQSHLASGNLSRTNFEDPTLTAATTLDHIPISAGDMKCQRPR
Acarolinensissyt7            PRLSLNGSLLSGAKLAASAT-G-------------LAGGREGRHGERPQL----REDGMKSSVSAHSEP-SIGKAGRGRWQMVQSHLAAGKLSLSNFEDSTLSTATTLEYIPTSAGDPKCQRPR
GgallusSYT7                  PRLSLNGSLLXGAKLTASATAG-------------LAGDRDGRPGDKQQC----AEDGMRSSISAHSEPGA-GKAARGRWHTVQSHLFAGKLTLSNFEDSTLSTATTLEYIPTSAGDPKCQRPR
TguttataSYT7                 PRLSLX-----------------------------------------------------------------------------------------XFEDSTLSTATTLEYIPTSAGDPKFQRPR
OanatinusSyt7                PRLSLNPPLLGPSGPAALGTSGTLLSGAKVAAAAGLAAEREGRPGEKPPL---PGEDAMRSSVSAHSEP-SNGKAGRGRWHTVQSHLAAGKLSLSNFEDSTLSTATTLEYIPSSVGEPKCQRPR
MdomesticaSyt7               ----------------------------------------------------------------------------------------------------------------------------
MmusculusSyt7var1            PRLSLNGTLLSGAKVATAAA-G-------------LAVEREGRLGEKPAPVPPPGEDALRSGGAAPSEPGSSGKAGRGRWRMVQSHLAAGKLNLSNFEDSTLSTATTLESIPSSAGEPKCQRPR
MmusculusSyt7var2          ● PRLSL-----------------------------------------------------------------------------------------------------------------------
MmusculusSyt7var3          ● PRLSL------------------------------------------------------------------------------------------NFEDSTLSTATTLESIPSSAGEPKCQRPR
MmusculusSyt7var4          ● ----------------------------------------------------------------------------------------------------------------------------
HsapiensSYT7var1             PRLSLNGTLLSGAKVA-AAA-G-------------LAVEREGRLGEKPAPVPPPGEDALRSGGAAPSEPGSGGKAGRGRWRTVQSHLAAGKLNLSNFEDSTLSTATTLESIPSSTGEPKCQRPR
HsapiensSYT7var2           ● PRLSL------------------------------------------------------------------------------------------NFEDSTLSTATTLESIPSSTGEPKCQRPR
HsapiensSYT7var3           ● PRLSL-----------------------------------------------------------------------------------------------------------------------
HsapiensSYT7var4           ● ----------------------------------------------------------------------------------------------------------------------------

                                                                           300
Trubripessyt7a               TLLRQQSLQQPLIRPPGPGLSHPPTTSQSLGQLNTATGQPGGGGGAGKGEGGTGSGE--GGGTRGGPRGARGSPTGAGASR-YRGGGAGGRSRANPGSWDYMMDQIRKRGLDVKSFLEGKMVVL
Trubripessyt7b               PLVRQQSLQQPLTHQPPPGPNDPPVTSQSLGQLHTGPGGGGHRGG---------------------PRGVRGSA--AGASR-YRGGGA-GRSRSNPGSWDHMVEQIRHRGLDVKSFLEGKMVVL
Tnigroviridissyt7a           TLLRQQSLQQPLIRPPGPGLSHPPTTSQSLGELNTASGQPGLGGGAGKGEGGSGSGE--GGGTRGAPRGARGSPAGAGASR-YRGGGAGGRSRANPGSWDYMMDQIRKRGLDVKSFLEGKMVVL
Tnigroviridissyt7b           PLVRQQSLQQPLTHQPPPGPNDPPVTSQSLGQLHTGPGGGGHRGG---------------------PRGVRGSA--AGASR-YRGGGA-GRSRSNPGSWDHMVEQIRHRGLDVKSFLEGKMVVL
Gaculeatussyt7a              TLLRQQSLQQPLIHPPVPVLVHPPTTSQSLGQLHTAPGQPGGGGGAGRGEAGGGSGEGGLAGTRGGPRGVRGAP-----SR-HRGGGGAGRSRGNPGSWDYTMDQMRSRGLDVKSFLEGKLVVL
Gaculeatussyt7b              PLVRQQSLQQPLTHQPPPGPNDPPVTSQSLGQLHTGPGGGGHRGG---------------------PRGVRGSP--AGASR-HRGGGA-GRSRSNPGSWDHMMEQIRHRGLDVKSFFEGKMVVL
Olatipessyt7a                TLLRQQSLQQPLIHPSGPGLGHPPTTSQSLGQLHTTPGHPGGGGGVGRGQGGGGTGDSRGAGTQVASQGAQGSQAGAGASR-CRGGGAGGHTRANPGSWDYMMDQMRNRGLDVKSFLEGKLVVL
Olatipessyt7b                PLVRQQSLQQPLTHQPPPGPNDPPVTSQSLGQLHTGPGGGGHRGG---------------------PRGVRGSP--AGASR-YRTG-A-GRSRSNPGSWDHMMEQIRHRGLDVKSFLEGKMVVL
Dreriosyt7a                  TLLRQQSLQQPLIQAPPPNLASRPPISQSLGQLHTQPGGAGPGGGGGAG-----------------SRNSRGEP-----AQ-HRSAGAGGRGSGNPGSVDHMMGQIKKRGLDVKSFLEGKMVVL
Dreriosyt7b                  PLVRQQSLQQPLTHQPPPGPNDPPATSQSLGQLHTGPGGGGHRGG---------------------PRGVRGSPAGAGATR-YRSGGG-ARSRSNPGSWDHMMGQIRTRGMDVKSFLEGKMVVL
Xtropicalissyt7var1          TLLRQQSLQQPLSLHQKHNH-CQPTTSQSLGQLQLQHQPPSTSSSNTSN-----------------TRPQRTGQNRPPTSAGSKHRPAGIRNRTNAGSWDYVVGQIRNRGLDMKSFLEGRMVVL
Xtropicalissyt7var2          TLLRQQSLQQPLSLHQKHNH-CQPTTSQSLGQLQLQHQPPSTSSSNTSN-----------------TRPQRTGQNRPPTSAGSKHRPAGIRNRTNAGSWDYVVGQIRNRGLDMKSFLEGRMVVL
Acarolinensissyt7            TLLRQQSLQQPLSQHQKPNH-SQPTTSQSLGHLQSH----SSSTSG--N-----------------PRGCRSGQSRQGTTAGSKQRTAGGRSRSNPGSWDHMVGQIRNRGLDMKSFLEGRMVVL
GgallusSYT7                  TLMRQQSLQQPLSQHQRSNH-SQPTTSQSLGHLQAH----SSSSGGAGN-----------------PRGSRGGQARQGIAAGSKQRT----SRSNPGSWDHVVGQIRNRGLDMKSFLEGRMVVL
TguttataSYT7                 TLVRQQSLQQPLSQHQRANH-SQPTTSQSLGHLQAH----SGSSAAAAN-----------------SRGSRGGPARQGTAAGSKQRMAGGRSRSNPGSWDHVVGQIRNRGLDMKSFLEGRMVVL
OanatinusSyt7                TLLRQQSLQQPLSQHQRAQQPGQPTTSQSLGQLQCH----TGSGPGA-N-----------------PRGYPRGQARSGSTAGAKHRGAGGRSRSNPGSWDHVVGQIRNRGLDMKSFLEGRMVVL
MdomesticaSyt7               ----------------------------------------------------------------------------------------------------------------------------
MmusculusSyt7var1            TLMRQQSLQQPLSQNQQGRQPSQPTTSQSLGQLQAH----AASAPGS-N-----------------PRAYGRGQARQGTSAGSKYRAAGGRSRSNPGSWDHVVGQIRNRGLDMKSFLEGRMVVL
MmusculusSyt7var2          ● ----------------------------------------------------------------------------------------------------------------------------
MmusculusSyt7var3            TLMRQQSLQQPLSQNQQGRQPSQPTTSQSLGQLQAH----AASAPGS-N-----------------PRAYGRGQARQGTSAGSKYRAAGGRSRSNPGSWDHVVGQIRNRGLDMKSFLEGRMVVL
MmusculusSyt7var4          ● ----------------------------------------------------------------------------------------------------------------------------
HsapiensSYT7var1             TLMRQQSLQQPLSQHQRGRQPSQPTTSQSLGQLQAH----MASAPGP-N-----------------PRAYGRGQARQGTSAGSKYRAAGGRSRSNPGSWDHVVGQIRNRGLDMKSFLEGRMVVL
HsapiensSYT7var2             TLMRQQSLQQPLSQHQRGRQPSQPTTSQSLGQLQAH----MASAPGP-N-----------------PRAYGRGQARQGTSAGSKYRAAGGRSRSNPGSWDHVVGQIRNRGLDMKSFLEGRMVVL
HsapiensSYT7var3           ● ----------------------------------------------------------------------------------------------------------------------------
HsapiensSYT7var4           ● ----------------------------------------------------------------------------------------------------------------------------
```

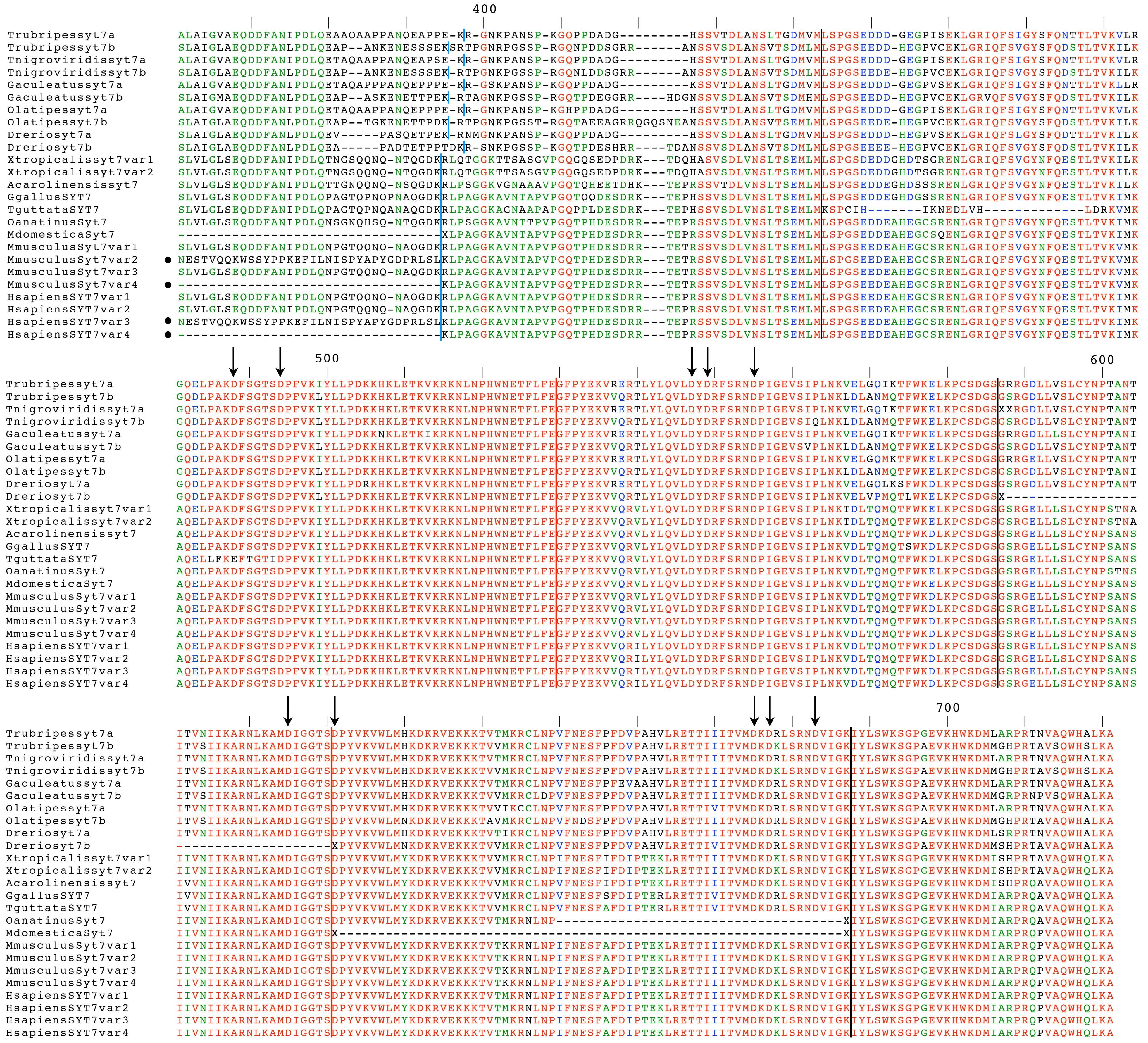

400
Trubripessyt7a ALAIGVAEQDDFANIPDLQEAAQAAPPANQEAPPE-KR-GNKPANSP-KGQPPDADG---------HSSVTDLANSLTGDMVMLSPGSEDDD-GEGPISEKLGRIQFSIGYSFQNTTLTVKVLR
Trubripessyt7b SLAIGLAEQDDFANLPDLQEAP--ANKENESSSEKSRTPGNRPGSSP-RGQNPDDSGRR------ANSSVSDLANSVTSDMLMLSPGSEEDD-HEGPVCEKLGRIQFSVGYSFQDSTLTVKILK
Tnigroviridissyt7a ALAIGVAEQDDFANIPDLQETAQAAPPANQEAPSE-KR-GNKPANSP-KGQPPDADG---------HSSVTDLANSLTGDMVMLSPGSEDDD-GEGPISEKLGRIQFSIGYSFQNTTLTVKVLR
Tnigroviridissyt7b SLAIGLAEQDDFANLPDLQEAP--ANKENESSSEK-RTPGNKPGSSP-RGQNLDDSGRR------ANSSVSDLANSVTSDMLMLSPGSEEDE-HEGPVCEKLGRIQFSVGYSFQDSTLTVKILK
Gaculeatussyt7a ALAIGVAEQDDFANIPDLQETAQAPPPANQEPPPE-KR-GSKPANSP-KGQPPDADG---------KSSVTDLANSLTGDMVMLSPGSEDDD-GEGPVSEKLGRIQFSIGYSFQNTTLTVKLLR
Gaculeatussyt7b SLAIGMAEQDDFANLPDLQEAP--ASKENETTPEK-RTAGNKPGSSP-RGQTPDEGGRR----HDGNSSVSDLANSVTSDMHMLSPGSEEDD-HEGPVCEKLGRVQFSVGYSFQDSTLTVKILK
Olatipessyt7a ALAIGVAEQDDFANIPDLQETAQAAPPANQEPPPE-KR-GNKPANSP-KGHPPDADG---------HSSVTDLANSLTGDMVMLSPGSEDDD-GEGPISEKLGRIQFSIGYSFQNTTLTVKVLK
Olatipessyt7b SLAIGLAEQDDFANLPDLQEAP--TGKENETTPDK-RTPGNKPGSST-RGQTAEEAGRRQGQSNEANSSVSDLANSVTSEMLMLSPGSEEDE-HEGPVCEKLGRIQFSVGYSFQDSTLTVKILK
Dreriosyt7a SLAIGLAEQDDFANLPDLQEV-----PASQETPEK-RNMGNKPANSP-KGQPPDADG---------HSSVSDLANSLTGDMVMLSPGSEDDD-HEGPVSEKLGRIQFSLGYSFQDTTLTVKILK
Dreriosyt7b SLAIGLAEQDDFANLPDLQEA-----PADTETPPTDKR-SNKPGSSP-KGQTPDESHRR----TDANSSVSDLANSVTSDMLMLSPGSEEEE-HEGPVCEKLGRIQFSVGYSFQDSTLTVKILK
Xtropicalissyt7var1 SLVLGLSEQDDFANIPDLQTNGSQQNQ-NTQGDKRLQTGGKTTSASGVPGQGQSEDPDRK---TDQHASVSDLVNSLTSEMLMLSPGSDEDDGHDTSGRENLGRIQFSVGYNFQESTLTVKILK
Xtropicalissyt7var2 SLVLGLSEQDDFANIPDLQTNGSQQNQ-NTQGDKRLQTGGKTTSASGVPGQGQSEDPDRK---TDQHASVSDLVNSLTSEMLMLSPGSDEDDGHDTSGRENLGRIQFSVGYNFQESTLTVKILK
Acarolinensissyt7 SLVLGLSEQDDFANIPDLQTTGNQQNQ-NSQGDKRLPSGGKVGNAAAVPGQTQHEETDHK---TEPRSSVTDLVNSLTSEMLMLSPGSEDDEGHDSSSRENLGRIQFSVGYNFQESTLTVKILK
GgallusSYT7 SLVLGLSEQDDFANIPDLQPAGTQPNQPNAQGDKRLPAGGKAVNTAPVPGQTQQDESDRK---TEPHSSVSDLVNSLTSEMLMLSPGSEDDEGHDGSSRENLGRIQFSVGYNFQESTLTVKIMK
TguttataSYT7 SLVLGLSEQDDFANIPDLQPAGTQPNQANAQGDKRLPAGGKAGNAAPAPGQPPLDESDRK---TEPHSSVSDLVNSLTSEMLMKSPCIH-------IKNEDLVH-------------LDRKVMK
OanatinusSyt7 SLVLGLSEQDDFANIPDLQNSGNQHSQ-NTQGDKRLPSGGKAVNTTPVPGQTPHDESDRR---TEPRSSVSDLVNSLTSEMLMLSPGSEDDEAHEGCSRENLGRIQFSVGYNFQESTLTVKIMK
MdomesticaSyt7 ----------------------------------XLPAGGKAVNTAPVPGQTPHDESDRR---TEPRSSVSDLVNSLTSEMLMLSPGSEDDEAHEGCSQENLGRIQFSVGYNFQESTLTVKIMK
MmusculusSyt7var1 SLVLGLSEQDDFANIPDLQNPGTQQNQ-NAQGDKRLPAGGKAVNTAPVPGQTPHDESDRR---TETRSSVSDLVNSLTSEMLMLSPGSEEDEAHEGCSRENLGRIQFSVGYNFQESTLTVKVMK
MmusculusSyt7var2 ● NESTVQQKWSSYPPKEFILNISPYAPYGDPRLSLKLPAGGKAVNTAPVPGQTPHDESDRR---TETRSSVSDLVNSLTSEMLMLSPGSEEDEAHEGCSRENLGRIQFSVGYNFQESTLTVKVMK
MmusculusSyt7var3 SLVLGLSEQDDFANIPDLQNPGTQQNQ-NAQGDKRLPAGGKAVNTAPVPGQTPHDESDRR---TETRSSVSDLVNSLTSEMLMLSPGSEEDEAHEGCSRENLGRIQFSVGYNFQESTLTVKVMK
MmusculusSyt7var4 ● ----------------------------------KLPAGGKAVNTAPVPGQTPHDESDRR---TETRSSVSDLVNSLTSEMLMLSPGSEEDEAHEGCSRENLGRIQFSVGYNFQESTLTVKVMK
HsapiensSYT7var1 SLVLGLSEQDDFANIPDLQNPGTQQNQ-NAQGDKRLPAGGKAVNTAPVPGQTPHDESDRR---TEPRSSVSDLVNSLTSEMLMLSPGSEEDEAHEGCSRENLGRIQFSVGYNFQESTLTVKIMK
HsapiensSYT7var2 SLVLGLSEQDDFANIPDLQNPGTQQNQ-NAQGDKRLPAGGKAVNTAPVPGQTPHDESDRR---TEPRSSVSDLVNSLTSEMLMLSPGSEEDEAHEGCSRENLGRIQFSVGYNFQESTLTVKIMK
HsapiensSYT7var3 ● NESTVQQKWSSYPPKEFILNISPYAPYGDPRLSLKLPAGGKAVNTAPVPGQTPHDESDRR---TEPRSSVSDLVNSLTSEMLMLSPGSEEDEAHEGCSRENLGRIQFSVGYNFQESTLTVKIMK
HsapiensSYT7var4 ● ----------------------------------KLPAGGKAVNTAPVPGQTPHDESDRR---TEPRSSVSDLVNSLTSEMLMLSPGSEEDEAHEGCSRENLGRIQFSVGYNFQESTLTVKIMK
500
600
Trubripessyt7a GQELPAKDFSGTSDPFVKIYLLPDKKHKLETKVKRKNLNPHWNETFLFEGFPYEKVRERTLYLQVLDYDRFSRNDPIGEVSIPLNKVELGQIKTFWKELKPCSDGSGRRGDLLVSLCYNPTANT
Trubripessyt7b GQDLPAKDFSGTSDPFVKLYLLPDKKHKLETKVKRKNLNPHWNETFLFEGFPYEKVVQRTLYLQVLDYDRFSRNDPIGEVSIPLNKLDLANMQTFWKELKPCSDGSGSRGDLLVSLCYNPTANT
Tnigroviridissyt7a GQELPAKDFSGTSDPFVKIYLLPDKKHKLETKVKRKNLNPHWNETFLFEGFPYEKVRERTLYLQVLDYDRFSRNDPIGEVSIPLNKVELGQIKTFWKELKPCSDGSXXRGDLLVSLCYNPTANT
Tnigroviridissyt7b GQDLPAKDFSGTSDPFVKLYLLPDKKHKLETKVKRKNLNPHWNETFLFEGFPYEKVVQRTLYLQVLDYDRFSRNDPIGEVSIQLNKLDLANMQTFWKELKPCSDGSGSRGDLLVSLCYNPTANT
Gaculeatussyt7a GQELPAKDFSGTSDPFVKIYLLPDKKNKLETKIKRKNLNPHWNETFLFEGFPYEKVRERTLYLQVLDYDRFSRNDPIGEVSIPLNKVELGQIKTFWKELKPCSDGSGRRGDLLLSLCYNPTANI
Gaculeatussyt7b GQDLPAKDFSGTSDPFVKLYLLPDKKHKLETKVKRKNLNPHWNETFLFEGFPYEKVVQRTLYLQVLDYDRFSRNDPIGEVSVPLNKLDLANMQTFWKELKPCSDGSGSRGDLLVSLCYNPTANT
Olatipessyt7a GQDLPAKDFSGTSDPFVKIYLLPDKKHKLETKVKRKNLNPHWNETFLFEGFPYEKVRERTLYLQVLDYDRFSRNDPIGEVSIPLNKVELGQMKTFWKELKPCSDGSGRRGELLVSLCYNPTANT
Olatipessyt7b GQELPAKDFSGTSDPFVKLYLLPDKKHKLETKVKRKNLNPHWNETFLFEGFPYEKVVQRTLYLQVLDYDRFSRNDPIGEVSIPLNKLDLANMQTFWKELKPCSDGSGSRGDLLVSLCYNPTANI
Dreriosyt7a GQDLPAKDFSGTSDPFVKIYLLPDRKHKLETKVKRKNLNPHWNETFLFEGFPYEKVRERTLYLQVLDYDRFSRNDPIGEVSIPLNKVELGQLKSFWKDLKPCSDGSGSRGDLLVSLCYNPTANT
Dreriosyt7b GQDLPAKDFSGTSDPFVKLYLLPDKKHKLETKVKRKNLNPHWNETFLFEGFPYEKVVQRTLYLQVLDYDRFSRNDPIGEVSIPLNKVELVPMQTLWKELKPCSDGSX-----------------
Xtropicalissyt7var1 AQELPAKDFSGTSDPFVKIYLLPDKKHKLETKVKRKNLNPHWNETFLFEGFPYEKVVQRVLYLQVLDYDRFSRNDPIGEVSIPLNKTDLTQMQTFWKELKPCSDGSGSRGELLLSLCYNPSTNA
Xtropicalissyt7var2 AQELPAKDFSGTSDPFVKIYLLPDKKHKLETKVKRKNLNPHWNETFLFEGFPYEKVVQRVLYLQVLDYDRFSRNDPIGEVSIPLNKTDLTQMQTFWKELKPCSDGSGSRGELLLSLCYNPSTNA
Acarolinensissyt7 AQELPAKDFSGTSDPFVKIYLLPDKKHKLETKVKRKNLNPHWNETFLFEGFPYEKVVQRVLYLQVLDYDRFSRNDPIGEVSIPLNKVDLTQMQTFWKDLKPCSDGSGSRGELLLSLCYNPSANS
GgallusSYT7 AQELPAKDFSGTSDPFVKIYLLPDKKHKLETKVKRKNLNPHWNETFLFEGFPYEKVVQRVLYLQVLDYDRFSRNDPIGEVSIPLNKVDLTQMQTSWKDLKPCSDGSGSRGELLLSLCYNPSANS
TguttataSYT7 AQELLFKEFTGTIDPFVKIYLLPDKKHKLETKVKRKNLNPHWNETFLFEGFPYEKVVQRVLYLQVLDYDRFSRNDPIGEVSIPLNKVDLTQMQTFWKDLKPCSDGSGSRGELLLSLCYNPSANS
OanatinusSyt7 AQELPAKDFSGTSDPFVKIYLLPDKKHKLETKVKRKNLNPHWNETFLFEGFPYEKVVQRVLYLQVLDYDRFSRNDPIGEVSIPLNKVDLTQMQTFWKELKPCSDGSGSRGELLLSLCYNPSTNS
MdomesticaSyt7 AQELPAKDFSGTSDPFVKIYLLPDKKHKLETKVKRKNLNPHWNETFLFEGFPYEKVVQRVLYLQVLDYDRFSRNDPIGEVSIPLNKVDLTQMQTFWKELKPCSDGSGSRGELLLSLCYNPSANS
MmusculusSyt7var1 AQELPAKDFSGTSDPFVKIYLLPDKKHKLETKVKRKNLNPHWNETFLFEGFPYEKVVQRVLYLQVLDYDRFSRNDPIGEVSIPLNKVDLTQMQTFWKDLKPCSDGSGSRGELLLSLCYNPSANS
MmusculusSyt7var2 AQELPAKDFSGTSDPFVKIYLLPDKKHKLETKVKRKNLNPHWNETFLFEGFPYEKVVQRVLYLQVLDYDRFSRNDPIGEVSIPLNKVDLTQMQTFWKDLKPCSDGSGSRGELLLSLCYNPSANS
MmusculusSyt7var3 AQELPAKDFSGTSDPFVKIYLLPDKKHKLETKVKRKNLNPHWNETFLFEGFPYEKVVQRVLYLQVLDYDRFSRNDPIGEVSIPLNKVDLTQMQTFWKDLKPCSDGSGSRGELLLSLCYNPSANS
MmusculusSyt7var4 AQELPAKDFSGTSDPFVKIYLLPDKKHKLETKVKRKNLNPHWNETFLFEGFPYEKVVQRVLYLQVLDYDRFSRNDPIGEVSIPLNKVDLTQMQTFWKDLKPCSDGSGSRGELLLSLCYNPSANS
HsapiensSYT7var1 AQELPAKDFSGTSDPFVKIYLLPDKKHKLETKVKRKNLNPHWNETFLFEGFPYEKVVQRILYLQVLDYDRFSRNDPIGEVSIPLNKVDLTQMQTFWKDLKPCSDGSGSRGELLLSLCYNPSANS
HsapiensSYT7var2 AQELPAKDFSGTSDPFVKIYLLPDKKHKLETKVKRKNLNPHWNETFLFEGFPYEKVVQRILYLQVLDYDRFSRNDPIGEVSIPLNKVDLTQMQTFWKDLKPCSDGSGSRGELLLSLCYNPSANS
HsapiensSYT7var3 AQELPAKDFSGTSDPFVKIYLLPDKKHKLETKVKRKNLNPHWNETFLFEGFPYEKVVQRILYLQVLDYDRFSRNDPIGEVSIPLNKVDLTQMQTFWKDLKPCSDGSGSRGELLLSLCYNPSANS
HsapiensSYT7var4 AQELPAKDFSGTSDPFVKIYLLPDKKHKLETKVKRKNLNPHWNETFLFEGFPYEKVVQRILYLQVLDYDRFSRNDPIGEVSIPLNKVDLTQMQTFWKDLKPCSDGSGSRGELLLSLCYNPSANS
700
Trubripessyt7a ITVNIIKARNLKAMDIGGTSDPYVKVWLMHKDKRVEKKKTVTMKRCLNPVFNESFPFDVPAHVLRETTIIITVMDKDRLSRNDVIGKIYLSWKSGPGEVKHWKDMLARPRTNVAQWHALKA
Trubripessyt7b ITVSIIKARNLKAMDIGGTSDPYVKVWLMHKDKRVEKKKTVVMKRCLNPVFNESFPFDVPAHVLRETTIIITVMDKDKLSRNDVIGKIYLSWKSGPAEVKHWKDMMGHPRTAVSQWHSLKA
Tnigroviridissyt7a ITVNIIKARNLKAMDIGGTSDPYVKVWLMHKDKRVEKKKTVTMKRCLNPVFNESFPFDVPAHVLRETTIIITVMDKDRLSRNDVIGKIYLSWKSGPGEVKHWKDMLARPRTNVAQWHALKA
Tnigroviridissyt7b ITVSIIKARNLKAMDIGGTSDPYVKVWLMHKDKRVEKKKTVVMKRCLNPVFNESFPFDVPAHVLRETTIIITVMDKDKLSRNDVIGKIYLSWKSGPAEVKHWKDMMGHPRTAVSQWHSLKA
Gaculeatussyt7a ITVNIIKARNLKAMDIGGTSDPYVKVWLMHKDKRVEKKKTVTMKRCLNPVFNESFPFEVAAHVLRETTIIITVMDKDRLSRNDVIGKIYLSWKSGPAEVKHWKDMLARPRTNVAQWHALKA
Gaculeatussyt7b ITVSIIKARNLKAMDIGGTSDPYVKVWLMHKDKRVEKKKTVVMKRCLDPVFNESFPFDVPAHVLRETTIIITVMDKDRLSRNDVIGKIYLSWKSGPAEVKHWKDMMGRPRNPVSQWHALKA
Olatipessyt7a ITVNIIKARNLKAMDIGGTSDPYVKVWLMHKDKRVEKKKTVVIKCCLNPVFNESFPFDVPAHVLRETTIVITVMDKDRLSRNDVIGKIYLSWKSGPAEVKHWKDMLARPRTNVAQWHALKA
Olatipessyt7b ITVSIIKARNLKAMDIGGTSDPYVKVWLMNKDKRVEKKKTAVMKRCLNPVFNDSFPFDVPAHVLRETTIIITVMDKDRLSRNDVIGKIYLSWKSGPAEVKHWKDMMGHPRTAVAQWHALKA
Dreriosyt7a ITVNIIKARNLKAMDIGGTSDPYVKVWLMHKDKRVEKKKTVTIKRCLNPVFNESFPFDVPAHVLRETTIIITVMDKDRLSRNDVIGKIYLSWKSGPGEVKHWKDMLSRPRTNVAQWHALKA
Dreriosyt7b --------------------XPYVKVWLMNKDKRVEKKKTVVMKRCLNPVFNESFPFDVPAHVLRETTIVITVMDKDRLSRNDVIGKIYLSWKSGPAEVKHWKDMMSHPRTAVAQWHALKA
Xtropicalissyt7var1 IIVNIIKARNLKAMDIGGTSDPYVKVWLMYKDKRVEKKKTVVMKRCLNPIFNESFIFDIPTEKLRETTIIITVMDKDKLSRNDVIGKIYLSWKSGPGEVKHWKDMISHPRTAVAQWHQLKA
Xtropicalissyt7var2 IIVNIIKARNLKAMDIGGTSDPYVKVWLMYKDKRVEKKKTVVMKRCLNPIFNESFIFDIPTEKLRETTIIITVMDKDKLSRNDVIGKIYLSWKSGPGEVKHWKDMISHPRTAVAQWHQLKA
Acarolinensissyt7 IVVNIIKARNLKAMDIGGTSDPYVKVWLMYKDKRVEKKKTVVMKRCLNPVFNESFIFDIPTEKLRETTIIITVMDKDKLSRNDVIGKIYLSWKSGPGEVKHWKDMISHPRQAVAQWHQLKA
GgallusSYT7 IVVNIIKARNLKAMDIGGTSDPYVKVWLMYKDKRVEKKKTVVMKRCLNPVFNESFSFDIPTERLRETTIVITVMDKDRLSRNDVIGKIYLSWKSGPGEVKHWKDMIARPRQAVAQWHQLKA
TguttataSYT7 IVVNIIKARNLKAMDIGGTSDPYVKVWLMYKDKRVEKKKTVVMKRCLNPVFNESFAFDIPTERLRETTIVITVMDKDRLSRNDVIGKIYLSWKSGPGEVKHWKDMIARPRQAVAQWHQLKA
OanatinusSyt7 IIVNIIKARNLKAMDIGGTSDPYVKVWLMYKDKRVEKKKTVTMKRNLNP-------------------------------------XIYLSWKSGPGEVKHWKDMIARPRQAVAQWHQLKA
MdomesticaSyt7 IIVNIIKARNLKAMDIGGTSX---XIYLSWKSGPGEVKHWKDMIARPRQPVAQWHQLKA
MmusculusSyt7var1 IIVNIIKARNLKAMDIGGTSDPYVKVWLMYKDKRVEKKKTVTKKRNLNPIFNESFAFDIPTEKLRETTIIITVMDKDKLSRNDVIGKIYLSWKSGPGEVKHWKDMIARPRQPVAQWHQLKA
MmusculusSyt7var2 IIVNIIKARNLKAMDIGGTSDPYVKVWLMYKDKRVEKKKTVTKKRNLNPIFNESFAFDIPTEKLRETTIIITVMDKDKLSRNDVIGKIYLSWKSGPGEVKHWKDMIARPRQPVAQWHQLKA
MmusculusSyt7var3 IIVNIIKARNLKAMDIGGTSDPYVKVWLMYKDKRVEKKKTVTKKRNLNPIFNESFAFDIPTEKLRETTIIITVMDKDKLSRNDVIGKIYLSWKSGPGEVKHWKDMIARPRQPVAQWHQLKA
MmusculusSyt7var4 IIVNIIKARNLKAMDIGGTSDPYVKVWLMYKDKRVEKKKTVTKKRNLNPIFNESFAFDIPTEKLRETTIIITVMDKDKLSRNDVIGKIYLSWKSGPGEVKHWKDMIARPRQPVAQWHQLKA
HsapiensSYT7var1 IIVNIIKARNLKAMDIGGTSDPYVKVWLMYKDKRVEKKKTVTMKRNLNPIFNESFAFDIPTEKLRETTIIITVMDKDKLSRNDVIGKIYLSWKSGPGEVKHWKDMIARPRQPVAQWHQLKA
HsapiensSYT7var2 IIVNIIKARNLKAMDIGGTSDPYVKVWLMYKDKRVEKKKTVTMKRNLNPIFNESFAFDIPTEKLRETTIIITVMDKDKLSRNDVIGKIYLSWKSGPGEVKHWKDMIARPRQPVAQWHQLKA
HsapiensSYT7var3 IIVNIIKARNLKAMDIGGTSDPYVKVWLMYKDKRVEKKKTVTMKRNLNPIFNESFAFDIPTEKLRETTIIITVMDKDKLSRNDVIGKIYLSWKSGPGEVKHWKDMIARPRQPVAQWHQLKA
HsapiensSYT7var4 IIVNIIKARNLKAMDIGGTSDPYVKVWLMYKDKRVEKKKTVTMKRNLNPIFNESFAFDIPTEKLRETTIIITVMDKDKLSRNDVIGKIYLSWKSGPGEVKHWKDMIARPRQPVAQWHQLKA
