# Supplementary material for: A manual collection of Syt, Esyt, Rph3a, Rph3al, Doc2, and Dblc2 genes from 46 metazoan genomes - an open access resource for neuroscience and evolutionary biology
Source: BMC Genomics. 2010 Jan 15;11:37. doi: 10.1186/1471-2164-11-37 (PMC2823689; doi:10.1186/1471-2164-11-37)
Supplement: Additional file 20 — Alignment of the vertebrate Syt9 sequences. Amino acid position is marked every hundred amino acids approximately, at the top of each page of the alignment. Splice variants are included and highlighted with black dots where they differ. Intron position and phase is indicated with a coloured bar between amino acids. Black bars indicate phase 0 introns. Red bars indicate phase +1 introns. Blue bars indicate phase +2 introns. The widely conserved motif of unknown function, just upstream of the C2A domain, is indicated. The five conserved acidic amino acids in each C2 domain are indicated by black arrows at the top of the alignment. X residues indicate where a portion of sequence is missing. [file 1471-2164-11-37-S20.PDF]

```
                                                                                                                  100
Trubripessyt9a          MPGDRDDEICQKALELLSDLCSKGEVQNEHCLDFIYHFRDLARPRYSDSDVSVSLLSLVVTACGLALFGVSLFVSWKLCWIPWRERGLSPTTKEAQGHLNPTMSPLSSQLAAEQP
Trubripessyt9b          MPAEREDEICQKALELLSDLCSKGEVQDDNCLDFIYYFRDLARPGYADTDVSVSLLSLLVTTCGLALFGVSLFVSWKLCWVPLSGRFLSDILKRAHFL-----------SGDQQP
Tnigroviridissyt9a      MPGDRDDEICQKALELLSDLCSKGEVQNEHCLDFIYYFRDIARPRYSDSDVSVSLLSLVVTACGLALFGVSLFVSWKLCWIPWRERGLSPTTKEAQGHLNPTMSPLPSQPGADQP
Tnigroviridissyt9b      MPAESVDEICEKALELLSDLCSRGEVHDDNCLDFIYYFRDLARPRYADTX-----------------------------------------------------------------
Gaculeatussyt9a         MPGDRDDEICQKALELLSDLCSKGEVQNEHCLDFIYYFRDLARPRYTDSDISVSLLSLVVTACGLALFGVSLFVSWKLCWIPWRERVLSPTTKEAHGHLNPIMSPLHSHLSPRQP
Gaculeatussyt9b         MPTEREDEICRKALELLSDLCSKGEVQDDHCLDYIYYFRDLARPRYTDSDVSVSLLSLLVTTCGLALFSVSLFVSWKLCWVPLSGRFLPDLLKGVHFL-----------GGDQQP
Olatipessyt9a           MPGDRDDEICQKALELLSDLCSKGDVQNENCLDFIYYFRDLARPRYSDSDISVSLLSLVVTACGLALFGVSLFVSWKLCWIPWRERGLSPTTKEPQGHINPTMSPLPSQPTPREP
Olatipessyt9b           MPAEREDEICRKALELLSDLCSKGEVQDDNCLDYIYHFRDLARPRYTDSDVSVSLLSLLVTTCGLALFSVSLFVSWKLCWVPLSGRFLPDALKGAHFL-----------GGDQQP
Dreriosyt9a             MPVDREDEICQKALELLSDLCSKGEVQNENCLDFIYYFRDLARPRYSDSDISISLLSLVVTACGLALFGVSLFVSWKLCWIPWRERGLSPGTKE--GHPDPPHPPPP--PLQPQP
Dreriosyt9b             MPADREDEICGKALELLSDLCSRGEVHNENCLDFTYYFRDLGRPRYSDSDVSVSLLSLLVTTCGLALFAVSLFVSWKLFWVPWRDRFLPSLKDQ--------------QGEQQP
Xtropicalissyt9         MPGDREQELCLKAVRLLAELCYSGIVEDESCLDFIYYLRDRARPRLSDSDVSVSLLSLLVTACGLALFGVSLFVSWKLCWIPWREHGLK--GIT---------------E-HEQL
Acarolinensissyt9       MPGGREDAICQKALKLLVELCSDGTLEHEGCLEFNYYLRDSGRPRSPDADITVSLLSLVVTACGLALFGVSLFVSWKLCWVPWRERGL---SGI---------------K-LESM
GgallusSYT9             MPGDREDEICQKALQLLAELCSVGAVENENCRDFIYYLRDRARPRLTDSDISISLLSLVVTACGLALFGVSLFVSWKLCWIPWRERGLPFGNKD---------------K-QESL
TguttataSYT9            -------------------------------------------------XISISLLSLVVTACGLALFGVSLFVSWKLCWIPWRERGLPFGNKD---------------SKQEPL
OanatinusSyt9           -------------------------------------------------XISVSLLSLMVTACGLALFGVSLFVSWKLCWLPWRDRGFFSGPKD---------------DNQEPL
MdomesticaSyt9          MPGDR-DEICQKALQLLAELCSSGAVEHDSCHDFIHYLRDRARPRLTDSDISVSLLSLVVTACGLALFSVSLFVSWKLCWIPWRERGLFSGSKD---------------N-QEPL
MmusculusSyt9var1       MPGAR-DALCHQALQLLAELCARGALEHDSCQDFIYHLRDRARPRLRDPDISVSLLTLVVTACGLALFGVSLFVSWKLCWVPWRERGLFSGSKD---------------NNQEPL
MmusculusSyt9var2       MPGAR-DALCHQALQLLAELCARGALEHDSCQDFIYHLRDRARPRLRDPDISVSLLTLVVTACGLALFGVSLFVSWKLCWVPWRERGLFSGSKD---------------NNQEPL
MmusculusSyt9var3       -------------------------------------------------------------------------------------------------------------------
MmusculusSyt9var4       -------------------------------------------------------------------------------------------------------------------
HsapiensSYT9var1        MPGAR-DALCHQALQLLAELCARGALEHDSCQDFIYHLRDRARPRLRDPDISVSLLTLVVTACGLALFGVSLFVSWKLCWVPWRERGLPSGSKD---------------NNQEPL
HsapiensSYT9var2      ● ---------------------------------MLAKVVEGDLAFKGGRDISVSLLTLVVTACGLALFGVSLFVSWKLCWVPWRERGLPSGSKD---------------NNQEPL

                                                                                                      200
Trubripessyt9a          VYTAVDPPAHDPRDLSQCSMAREPTPMASVVSVEAPVTPMSPPPVVLAAPEAAMKISHTSPDIPLDAQSKAQE-NGVHTTP--RMQRQTTEPP---------PQGSIRRHMNLSN
Trubripessyt9b          VLNEVDG---DEREDSEDDCMKEA----------------SCPPLAVPIVESGLKISHTSPDISLEVQAKS-EKNHLHTLARDRVQRQITEPTS------SVRHNSIRRQMNLSN
Tnigroviridissyt9a      VYTAVDPPTHGRRESSQCYMAREPTPMASVVSVEAPVTPVSPPPVVLATPEAAMKISHTSPDIPLDAQSKTQE-NGVHTTP--RMQRQTTEPP---------PQGSIRRHMNLSN
Tnigroviridissyt9b      ---XVDG---EEKEDSEDECVKEP----------------TFPTLAVPILESGLKISHTSPDISLEVQAKVQK-KHVHTFARDRVKRQMTEPTS------SVRHNSIRRQMNLSD
Gaculeatussyt9a         VYTAVDPLPLNRRESSHCSIAREPTPVASVVSVEAPSTPVSPPPVVVATPEAAMKISHTSPDIPLDAQTKSRE-NGVNTNP--RMQRQTTDPQTSGNLTSEIGQGSIRRHMNLSN
Gaculeatussyt9b         VLTEVDG---DEREYSEDGCVKEP----------------SGPLIAVAVPESGLKISHTSPDIPLEIQTKV-EKSQVHTLARDRVQRQITEPTS------SVRHNSIRRQMNLSN
Olatipessyt9a           VYTAVDPPMHNRRKSSHCSITKEPTPVASVVSVEAPGTPVSPPPVVMATPEAAMKISHTSPDIALDAQSKSRE-SGIHTNP--RMQRQTTDPAPGGCSISEIGQGSIRRHMNLSN
Olatipessyt9b           VLTEVDG---EEREYSEDGCMKDT----------------SGPPSAVAVPESALKISHTSPDIPLESQTKL-EKNQVHTLARDRVQRQITEPTS------SVRHNSIRRQMNLSN
Dreriosyt9a             IYTEVDA-TLDRRSNARSSVVKETTVPPTPVS---PAVPGSPPAVPV--PEAALKISHTSPDIPLEVESKTQE-NGVHGNT--RMQRQITDPSS-----TCVRQSSIRRQMNQSN
Dreriosyt9b             VLAEVEG---LEREYSED-FEKDP----------------NAP---LVLPESAMKISHTSPDIQLEAQTKAKELNHIHIA---RVQRQVTEPTS------SVRHNSIRRQMNVSN
Xtropicalissyt9         NYTDSDT---NERDYSDNYLGQPS-----------------------LFPESSMKISHTSPDIQLEAKTSTKE-NCVHNV---RMHRQITEPTS------SARHNSIRRQLNLSN
Acarolinensissyt9       NYTDTET---NDNEYSEDYLGQPA-----------------------TYPDSSMKISHTSPDIPLDAKAGTKE-NCAHNV---RMHRQITEPTP------SARHNSFRRQLNLSN
GgallusSYT9             NYTDTET---NDHDYSEDYLGQPT-----------------------TYPESSMKISHTSPDIPVDAQAGTKE-NCVHNA---RMHRQITEPTS------SARHNSIRRQLNLSN
TguttataSYT9            HYADADT---NERDYSEDYLGQPA-----------------------AYPEASMKISHTSPDLPLDTQEGAKE-NCVPNV---RMQRQTTEPTS------SARHNSLRRQLNLSN
OanatinusSyt9           NYTDTET---NDHENSEDYLGHPT-----------------------SFPEPSMKISHTSPDIQLDAQAGTKE-NCVHSV---RVQRQITEPTS------STRHNSIRRQLNLSN
MdomesticaSyt9          NYTETET---NDPEYSEDYQEQPS-----------------------PYPDAAMKISHTSPDIPLASQAENKE-NCVHGA---RMQRQVTEPTP------SARHNSIRRQLNLSN
MmusculusSyt9var1       NYTDTET---NEQENSEDFLDPPT-----------------------PCPDSSMKISHTSPDIPLSTQPGGQE-NCAHAV---RVQRQVTEPTP------SARHNSIRRQLNLSN
MmusculusSyt9var2       NYTDTET---NEQENSEDFLDPPT-----------------------PCPDSSMKISHTSPDIPLSTQPGGQE-NCAHAV---RVQRQVTEPTP------SARHNSIRRQLNLSN
MmusculusSyt9var3       -------------------------------------------------------------------------------------------------------------------
MmusculusSyt9var4       -------------------------------------------------------------------------------------------------------------------
HsapiensSYT9var1        NYMDTET---NEQENSEDFLDPPT-----------------------PCPDSSMKISHTSPDIPLSTQTGIQE-NCAHGV---RVQRQVTEPTS------SARHNSIRRQLNLSN
HsapiensSYT9var2        NYMDTET---NEQENSEDFLDPPT-----------------------PCPDSSMKISHTSPDIPLSTQTGIQE-NCAHGV---RVQRQVTEPTS------SARHNSIRRQLNLSN
```

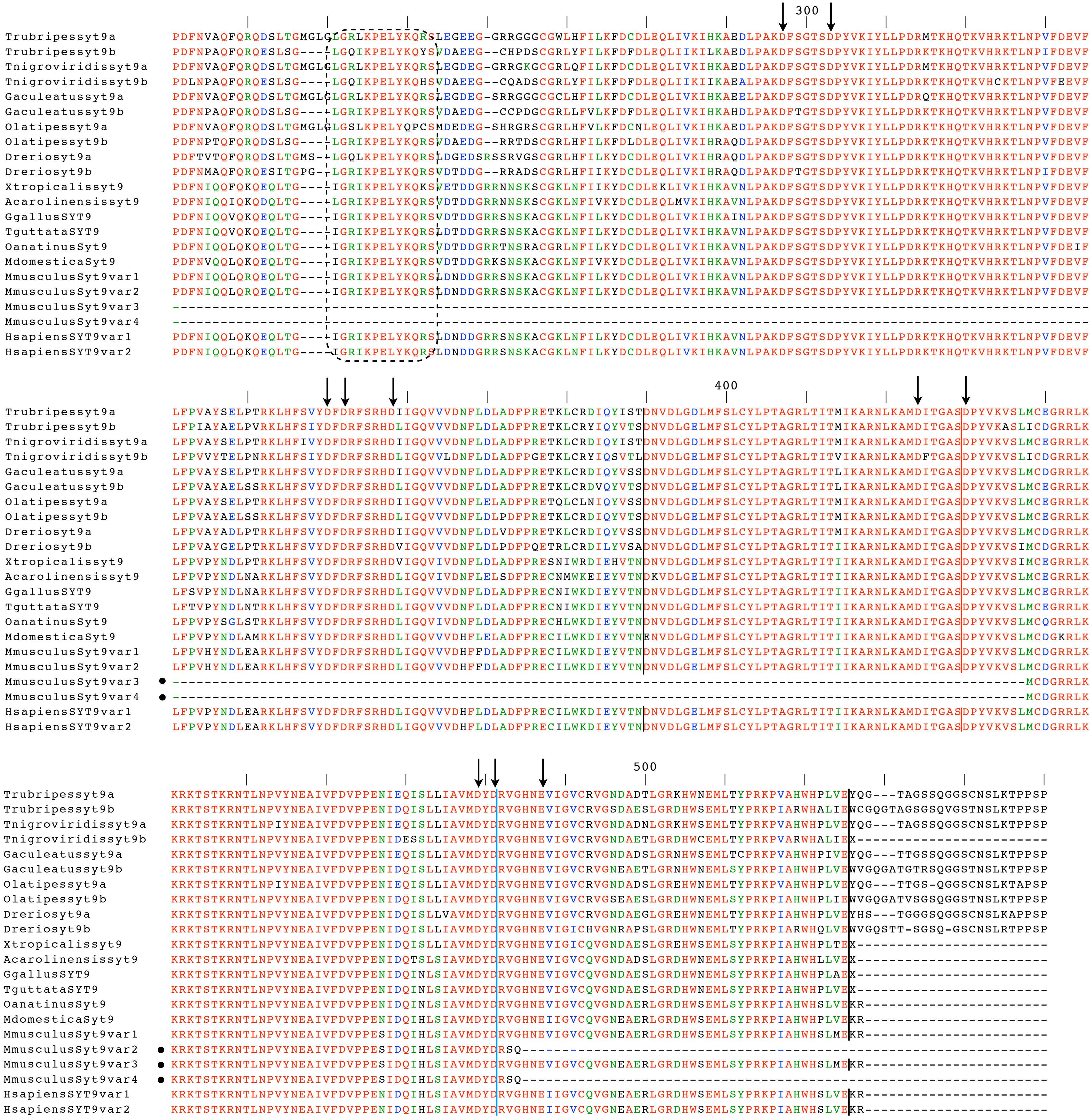
300
400
500
Trubripessyt9a
Trubripessyt9b
Tnigroviridissyt9a
Tnigroviridissyt9b
Gaculeatussyt9a
Gaculeatussyt9b
Olatipessyt9a
Olatipessyt9b
Dreriosyt9a
Dreriosyt9b
Xtropicalissyt9
Acarolinensissyt9
GgallusSYT9
TguttataSYT9
OanatinusSyt9
MdomesticaSyt9
MmusculusSyt9var1
MmusculusSyt9var2
MmusculusSyt9var3
MmusculusSyt9var4
HsapiensSYT9var1
HsapiensSYT9var2
