# Supplementary material for: A manual collection of Syt, Esyt, Rph3a, Rph3al, Doc2, and Dblc2 genes from 46 metazoan genomes - an open access resource for neuroscience and evolutionary biology
Source: BMC Genomics. 2010 Jan 15;11:37. doi: 10.1186/1471-2164-11-37 (PMC2823689; doi:10.1186/1471-2164-11-37)
Supplement: Additional file 21 — Alignment of the vertebrate Syt3 sequences. Amino acid position is marked every hundred amino acids approximately, at the top of each page of the alignment. Intron position and phase is indicated with a coloured bar between amino acids. Black bars indicate phase 0 introns. Red bars indicate phase +1 introns. Blue bars indicate phase +2 introns. The widely conserved motif of unknown function, just upstream of the C2A domain, is indicated. The five conserved acidic amino acids in each C2 domain are indicated by black arrows at the top of the alignment. X residues indicate where a portion of sequence is missing. [file 1471-2164-11-37-S21.PDF]

```
                                                                                                                      100
                             |         |         |         |         |         |         |         |         |         |
Trubripessyt3       MSGDWDEDVCKKAMELVEELCFNSPRGYSQSERCQEFSYLL--------------RGRNRQLDTEISVSLLSVIVTFCGIVLLSVSLFVSWKLCWLPWRDKEGGGLS
Tnigroviridissyt3   MSGDWDEDLCKKALVLVEELCFNSPRDYSQSERCQEFSYLL--------------RGRNRQLDSEISVSLLSVIVTFCGVVLLSVSLFVSWKLCWLPWRDKEGGGLS
Gaculeatussyt3      MSGDWDEDLCKKALALVEELCFNSPRGYSQSARCQEFSYLL--------------RGRIGQLDTEISVSLLSVIVTFCGIVLLSVSLFVSWKLCWLPWRDNEGGGLS
Olatipessyt3        MSGDWEEDLCKKALMFVEELCLNSPKGYSQSERCQEFRYLL--------------GGRSSRVDTEISVSLLSVIVTFCGIVLLSVSLFVSWKLCWLPWRDQEGGGLS
Dreriosyt3          MSGDWDDDICKRAMVLVEELCFRSG-GLSQSASCQEFSYMM--------------RGPNRQINTEISVSLLSVIVTFCGIVLLSVSLFVSWKLCWLPWRDKEGGGLS
Xtropicalissyt3     MSGDYEEDLCRSTMMLMRELCSSM-QSYAENEKCLEFYDLL------------RSQGMTHIRNTDVSVSLLSVIVTFCGIVLLGVSLFVSWKLCWIPWRDKGS-SQT
Acarolinensissyt3   MSGDYDEDRHRRAMELLHVLCANT-CAAQEREKCQELTGHVGPMNSGESPKERERKSQGKGARPEILVSLLSVIVTFCGIVLLGVSLFVSWKLCWIPWRDKGAASQS
TguttataSYT3        -----------------------------------------------------------------------------------------------------------
OanatinusSyt3       MSGVYENDLCRRALTLVSDLCSRL-RDSETSDKCQEFNDLR-------------IRGYPRASDADISVSLLSVIVTFCGIVLLGVSLFVSWKLCWVPWRDKGGSPWG
MmusculusSyt3       MSGDYEDDLCRRALILVSDLCARV-RDADTNDRCQEFNELR-------------IRGYPRGPDADISVSLLSVIVTFCGIVLLGVSLFVSWKLCWVPWRDKGGSAVG
HsapiensSYT3        MSGDYEDDLCRRALILVSDLCARV-RDADTNDRCQEFND-R-------------IRGYPRGPDADISVSLLSVIVTFCGIVLLGVSLFVSWKLCWVPWRDKGGSAVG

                                                                                                                      200
                             |         |         |         |         |         |         |         |         |         |
Trubripessyt3       LTSGLLPGSAGVG---GLGGGSLLPPLMQRKEGHSSSSLYPSLGQ---HHPHFSDMVGLERGEG---GGVVGGPHETQEPSYLDMDTYPNNAGTLKLSQTSPDVPNS
Tnigroviridissyt3   LTSGLLPGSAGIG---GLGGGSLLPPLMQRKEGHSSSSLYPSLGQ---HHPHFSDLVGLERGE---VGGVVGGPHDTQEPSYLDMDSYPNNAGTLKLSQTSPDVPNS
Gaculeatussyt3      LTSGLLPGSAGVGSLGGTGGSSLLPPLLQRKEGHSSSSLYPSLGQQGQHHPHFSDLVGLERGE---VGGVVGGPQESQEPSYLDMDSYPNNAGTLKLSQTSPDVPNS
Olatipessyt3        LTSGLLPGCAGVGSLGGTGGSSLLPQALQRKDGHSSSSLYPSLGQQ--HHPHFSDLVGLERGE---VGGVVGGPQETQEHSYLDMDSYPNNAGTLKLSQTSPDVPNS
Dreriosyt3          LTSGLLPGGAGLGGVGGGGGASLLPPLLQRKDTHSSSSLYPTLSQQGQHHPHFSDVMGVEGGGGLVVGGVVGGPQEMQEHSYLDMDSYPNNAATLKLSQTSPELPPA
Xtropicalissyt3     ----------------------------QRKDHGHHGHL---------HHSHYGDLLADR---------VELGP-EMPERSYLDMDSYPE--TNIKISQTSPDIPVD
Acarolinensissyt3   ----------------------------QRKEHLLHAHL---------HHSPFGDLLAER---------VELGP-EMPERSYLDMDSYPE--ANLKVSQTSPDLPVE
TguttataSYT3        -----------------------------------------------------------------------------------------------------------
OanatinusSyt3       G----------------------------------------------------------------------------------------------------------
MmusculusSyt3       GGPLRKDLAPGVGLAGLVGGGGHHLGASLGGHPLLGGPHHHGHTA---HHPPFAELLEPGGLG----GSEPPEPSYLDMDSYPEAAVASVVAAGVKPSQTSPELPSE
HsapiensSYT3        GGPLRKDLGPGVGLAGLVGGGGHHLAAGLGGHPLLGGPHHHAHAA---HHPPFAELLEPGSLG----GSDTPEPSYLDMDSYPEAAAAAV-AAGVKPSQTSPELPSE

                                                                                                                      300
                             |         |         |         |         |         |         |         |         |         |
Trubripessyt3       EVGAHPHKDLPNAHS--QQQV------------------------TARAKPMTHQLSSPDFHE----EK--EQVTCIGQIKPELYRPK-GDQGEGKQGDN-------
Tnigroviridissyt3   EVGAHPQKDLPNAHS--QQQV------------------------TSRAKPMTHQLSSPDFH-----EEK-EQVTCIGQIKPELYRPK-GDQGDGKQGDT-------
Gaculeatussyt3      EVGAQPHKDLPNAHS--QQQV------------------------TSRPKPMTHQLSSPDFHG----EEK-EQVTSIGQIKPELYRPK-GDQGEGKQGDN-------
Olatipessyt3        EGGAQPQKDLPNAHS--QQQV------------------------TPRPKPMTHQLSSPDFHG----EEK-EQVTCIGQIKPELYRPK-GDQGEGKQGDN-------
Dreriosyt3          TEGGSAAKDLPNAHS--QQQV------------------------TTRPKPMTHQLSSPDFHG----EEKSEQLTSIGQIKPELYRLRQGDQGDGKQGNN-------
Xtropicalissyt3     SQP-PKESQIPYAQS--HQQVANIAPANVAPANVAPANVAPANRYSTLPRPMTQQLSSPDFSGHV--DEKPEQATSIGQIKPELYKQRSADAEMKKQ---------E
Acarolinensissyt3   GQTGTKEGTIPNAHS--HQQVASLAPTP---------------RYNTLPRPVTQQLSSPDTGVHIGGEEKVEQVTSIGQIKPELYKQRSGESDSKKG---------E
TguttataSYT3        ----------------------------------------------------------------------XEPPAHLGQIQPELYGPAGDPEPFGARGGPGGGPGGP
OanatinusSyt3       --------------------------------------------------------SSPQFRH-CSLPRGERP--LIGQIKPELYQRGGEGGRRGGPGGPGVGAGGA
MmusculusSyt3       GGTGSGLLLLPPSGGGLPSAQSHQQVTSLAPTTRYPALPRPLTQQTLTTQADPSTEERPPALP-LPLPGGEEKAKLIGQIKPELYQGTGPGGRRGG-G---SGEAG-
HsapiensSYT3        GGAGSGLLLLPPSGGGLPSAQSHQQVTSLAPTTRYPALPRPLTQQTLTSQPDPSSEERPPALP-LPLPGGEEKAKLIGQIKPELYQGTGPGGRRSG-GGPGSGEAGT
```

```
                                                                                              400
Trubripessyt3         -------------CGKISFLLRYAFNTEQLVVKILKALDLPAKDANGFSDPYVKIYLLPDRKKKFQTKVHRKTLNPVFNETFQFGVPLNELHSRKLHFSVYDFDRFS
Tnigroviridissyt3     -------------CGKISFLLRYAFNTEQLVVKILKALDLPAKDANGFSDPYVKIYLLPDRKKKFQTKVHRKTLNPVFNETFQFGVPLNELHSRKLHFSVYDFDRFS
Gaculeatussyt3        -------------CGKISFLLRYAFNTEQLVVKILKALDLPAKDANGFSDPYVKIYLLPDRKKKYQTKVHRKTLNPVFNETFQFGVPLNELHSRKLHFSVYDFDRFS
Olatipessyt3          -------------CGKISFLLRYAFNTEQLVVKILKALDLPAKDANGFSDPYVKIYLLPDRKKKFQTKVHRKTLNPVFNETFQFGVPLNELHSRKLHFSVYDFDRFS
Dreriosyt3            -------------CGKISFLLRYAFNTEQLVVKILKALDLPAKDANGFSDPYVKIYLLPDRKKKFQTKVHRKTLNPVFNETFQFGVPLAELHARKLHFSVYDFDRFS
Xtropicalissyt3       A----------TSCGRISFILRYAYNSEQLVVKILKALELPAKDANGFSDPYVKMYLLPDRKKKFQTKVHRKTLNPIFNETFHFNVPFNELQNRKLHFSIYDFDRFS
Acarolinensissyt3     A----------QSCGRLSFALRYAYSTEQLVVRILRALDLPAKDANGFSDPYVKMYLLPDRKKKFQTKVHRKTLNPVFNETFNFNVPFAELPSRKLHFSVYDFDRFS
TguttataSYT3          T----------PSCGRLNVSLRYSYGSQQLLVRVLRARDLPAKDSNGFSDPYVKIYLLPDRKKKFQTKVLRRTLNPDWDETFSFGVPFAELPARRLHFSVYDFDRFS
OanatinusSyt3         GDGGDGDGEGGAPCGRFSFALRYLYGSDQLVVRILQALDLPAKDSNGFSDPYVKIYLLPDRKKKFQTKVHRKTLNPVFNETFQFSVPLAELPHRKLHFSIYDFDRFS
MmusculusSyt3         -----------APCGRISFALRYLYGSDQLVVRILQALDLPAKDSNGFSDPYVKIYLLPDRKKKFQTKVHRKTLNPIFNETFQFSVPLAELAQRKLHFSVYDFDRFS
HsapiensSYT3          G----------APCGRISFALRYLYGSDQLVVRILQALDLPAKDSNGFSDPYVKIYLLPDRKKKFQTKVHRKTLNPVFNETFQFSVPLAELAQRKLHFSVYDFDRFS

                                                                                              500
Trubripessyt3         RHDLIGQVVVDNLLDFSEGSGDKPIWRDIVEGTAEKADLGELNFSLCYLPTAGRLTVTIX-----------------------------------------------
Tnigroviridissyt3     RHDLIGQVVVDNLLDFSEGSGDKPIWRDIVEGTAEKADLGELNFSLCYLPTAGRLTVTIIKATNLKAMDLTGFSDPYVKASLVCDGRRLKKRKTSIKKNTLNPTYNE
Gaculeatussyt3        RHDLIGQVVVDNLLDFSEGSGDKPVWRDIVEGTAEKADLGELNFSLCYLPTAGRLTATVIKATNLKAMDLTGFSDPYVKASLICDGRRLKKRKTSIKKNTLNPTYNE
Olatipessyt3          RHDLIGQVVVDNLLDFSEGSGDKPIWRDIVEGTAEKADLGELNFSLCYLPTAGRLTATIIKANNLKAMDLTGFSDPYVKASLICDGRRLKKRKTSIKKNTLNPTYNE
Dreriosyt3            RHDLIGQVVVDNLLDFSEGTGEKPIWRDIVEGTAEKADLGELNFSLCYLPTAGRLTVTIIKATNLKAMDLTGFSDPYVKASLVCEGRRLKKRKTSIKKNTLNPTYNE
Xtropicalissyt3       RHDLIGQVVLDNLLEFSNATDETPIWRDILEASSEKADLGEINFSLCYLPTAGRLTATIIKATNLKAMDLTGFSDPYVKASLICEGRRLKKRKTSIKKNTLNPTYNE
Acarolinensissyt3     RHDLIGQVVLDNLLEIAERDNDTPLWRDIMEASSEKADLGELNFSLCYLPTAGRLTVTIIKATNLKAMDLTGFSDPYVKASLMCEGRRLKKRKTSIKKNTLNPSYNE
TguttataSYT3          RHDLIGQVVLDNLLEAAEARPEMVIWRDIQEGTGEKADLGEVNFSLCYLPTAGRLTVTVIRASNLRAMDLTGYSDPYVKASLMAEGRRLKKRKTSIKKNTLNPSYNE
OanatinusSyt3         RHDLIGQVVLDNLLELAEQPPDRPLWRAIVEGSSEKADLGEVNFSLCYLPTAGRLTVTIIKASNLKAMDLTGFSDPYVKASLICEGRRLKKRKTSIKKNTLNPTYNE
MmusculusSyt3         RHDLIGQVVLDNLLELAEQPPDRPLWRDILEGGSEKADLGELNFSLCYLPTAGRLTVTIIKASNLKAMDLTGFSDPYVKASLISEGRRLKKRKTSIKKNTLNPTYNE
HsapiensSYT3          RHDLIGQVVLDNLLELAEQPPDRPLWRDIVEGGSEKADLGELNFSLCYLPTAGRLTVTIIKASNLKAMDLTGFSDPYVKASLISEGRRLKKRKTSIKKNTLNPTYNE

Trubripessyt3         ------------------------XIGHNEVIGMCRVGSDAEGP-GREHWTAMLGNPRKPIEHWHQLVEEKAIGTFVSKSETASSPKPHIVVDSPHSD
Tnigroviridissyt3     ALVFDIPNENIESVSIVIAVMDYDCIGHNEVIGMCRVGSDADGP-GREHWTAMLGNPRKPIEHWHQLVEEKAIGTFVSKSETASSPKPHIVVDSPHSD
Gaculeatussyt3        ALVFDIPNENIESVSIIIAVMDYDCIGHNEVIGMCRVGSEADGP-GREHWTAMLANPRKPIEHWHQLVEEKAIGTFVSKTATASSPKPNIVVDSPHSD
Olatipessyt3          ALVFDIPNENIESVSIIIAVMDYDCIGHNEVIGMCRVGSDADGP-GREHWAAMLANPRKPIEHWHQLVEEKAIGTFVSKSATTPTSKPHIVVDSPHSD
Dreriosyt3            ALVFDIPNENIESVSLIIAVMDYDCIGHNEVIGMCRMGSDADGP-GREHWTAMLANPRKPIEHWHQLVEEKSINTYVSKSA-APSPKPNIVVDSPHSE
Xtropicalissyt3       ALVFDIPNENMDHVSLTIAVMDYDCIGHNEVIGMCRVGSDADMQ-GREHWNEMLANPRKPIEHWHQLVEEKALNSFMTKSP-PPRDKPSIVVDNTQSD
Acarolinensissyt3     ALVFDIPQDSMEHVSITLAVMDYDCIGHNEVIGMCRVGSDADAP-GRDHWAEMLANPRKPIEHWHQLVEEKTLNIYINKNP-PARDKPSIVVETVHSD
TguttataSYT3          ALVFDVPHESVHHVSLTIAVVDYDX-------------------------------------------------------------------------
OanatinusSyt3         ALVFDVAPESVESVGLSIAVMDYDCIGHNEVIGVCRVGSDAADPHGREHWAEMLANPRKPVEHWHTLVEX-----------------------------
MmusculusSyt3         ALVFDVAPESVENVGLSIAVVDYDCIGHNEVIGVCRVGPEAADPHGREHWAEMLANPRKPVEHWHQLVEEKTLSSFTKGGKGLSEKENSE--------
HsapiensSYT3          ALVFDVAPESVENVGLSIAVVDYDCIGHNEVIGVCRVGPDAADPHGREHWAEMLANPRKPVEHWHQLVEEKTVTSFTKGSKGLSEKENSE--------
```
