# Supplementary material for: A manual collection of Syt, Esyt, Rph3a, Rph3al, Doc2, and Dblc2 genes from 46 metazoan genomes - an open access resource for neuroscience and evolutionary biology
Source: BMC Genomics. 2010 Jan 15;11:37. doi: 10.1186/1471-2164-11-37 (PMC2823689; doi:10.1186/1471-2164-11-37)
Supplement: Additional file 22 — Alignment of the vertebrate Syt6 sequences. Amino acid position is marked every hundred amino acids approximately, at the top of each page of the alignment. Splice variants are included and highlighted with black dots where they differ. Intron position and phase is indicated with a coloured bar between amino acids. Black bars indicate phase 0 introns. Red bars indicate phase +1 introns. Blue bars indicate phase +2 introns. The widely conserved motif of unknown function, just upstream of the C2A domain, is indicated. The five conserved acidic amino acids in each C2 domain are indicated by black arrows at the top of the alignment. X residues indicate where a portion of sequence is missing. [file 1471-2164-11-37-S22.PDF]

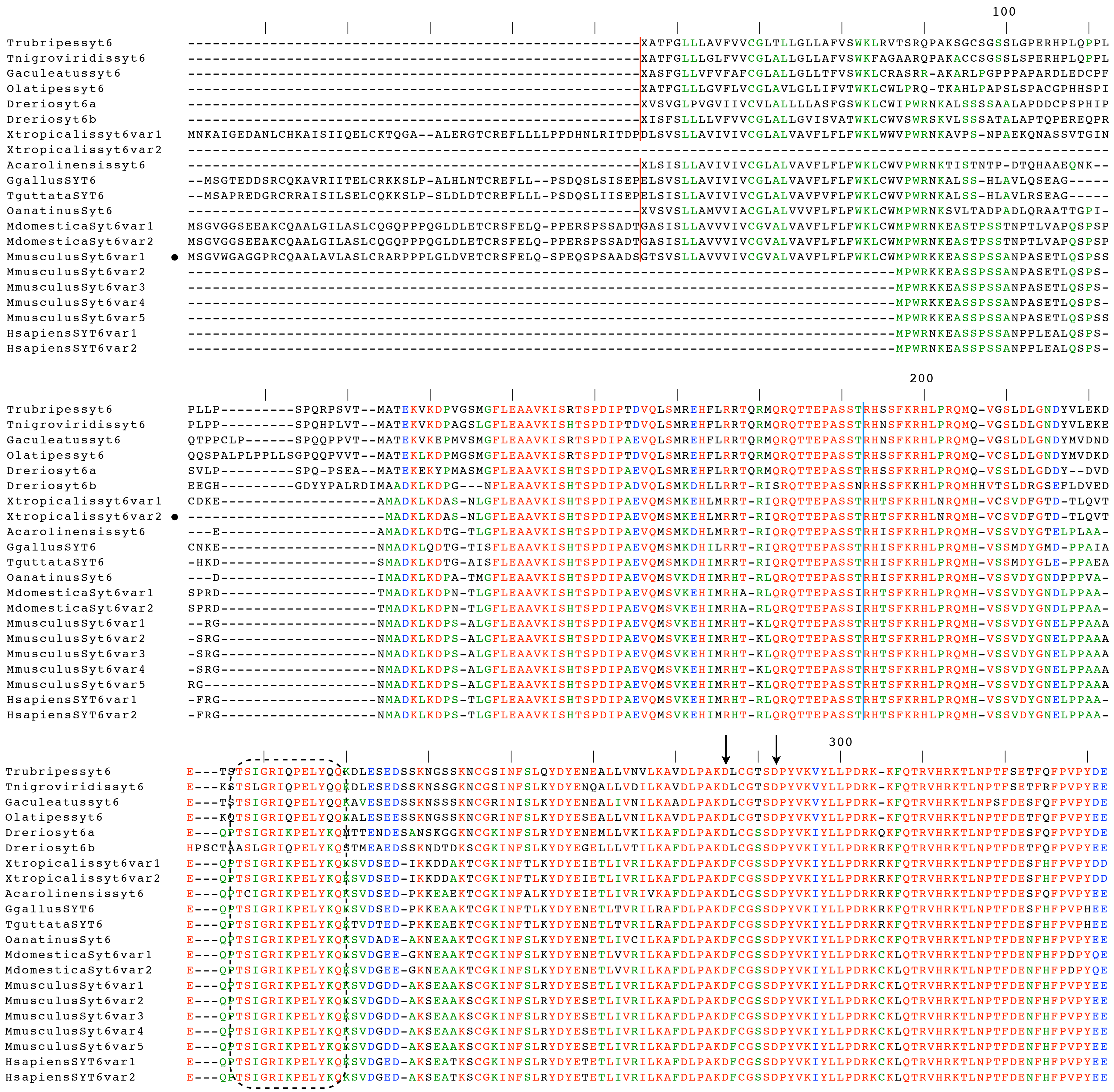
100
200
300
Trubripessyt6
Tnigroviridissyt6
Gaculeatussyt6
Olatipessyt6
Dreriosyt6a
Dreriosyt6b
Xtropicalissyt6var1
Xtropicalissyt6var2
Acarolinensissyt6
GgallusSYT6
TguttataSYT6
OanatinusSyt6
MdomesticaSyt6var1
MdomesticaSyt6var2
MmusculusSyt6var1
MmusculusSyt6var2
MmusculusSyt6var3
MmusculusSyt6var4
MmusculusSyt6var5
HsapiensSYT6var1
HsapiensSYT6var2

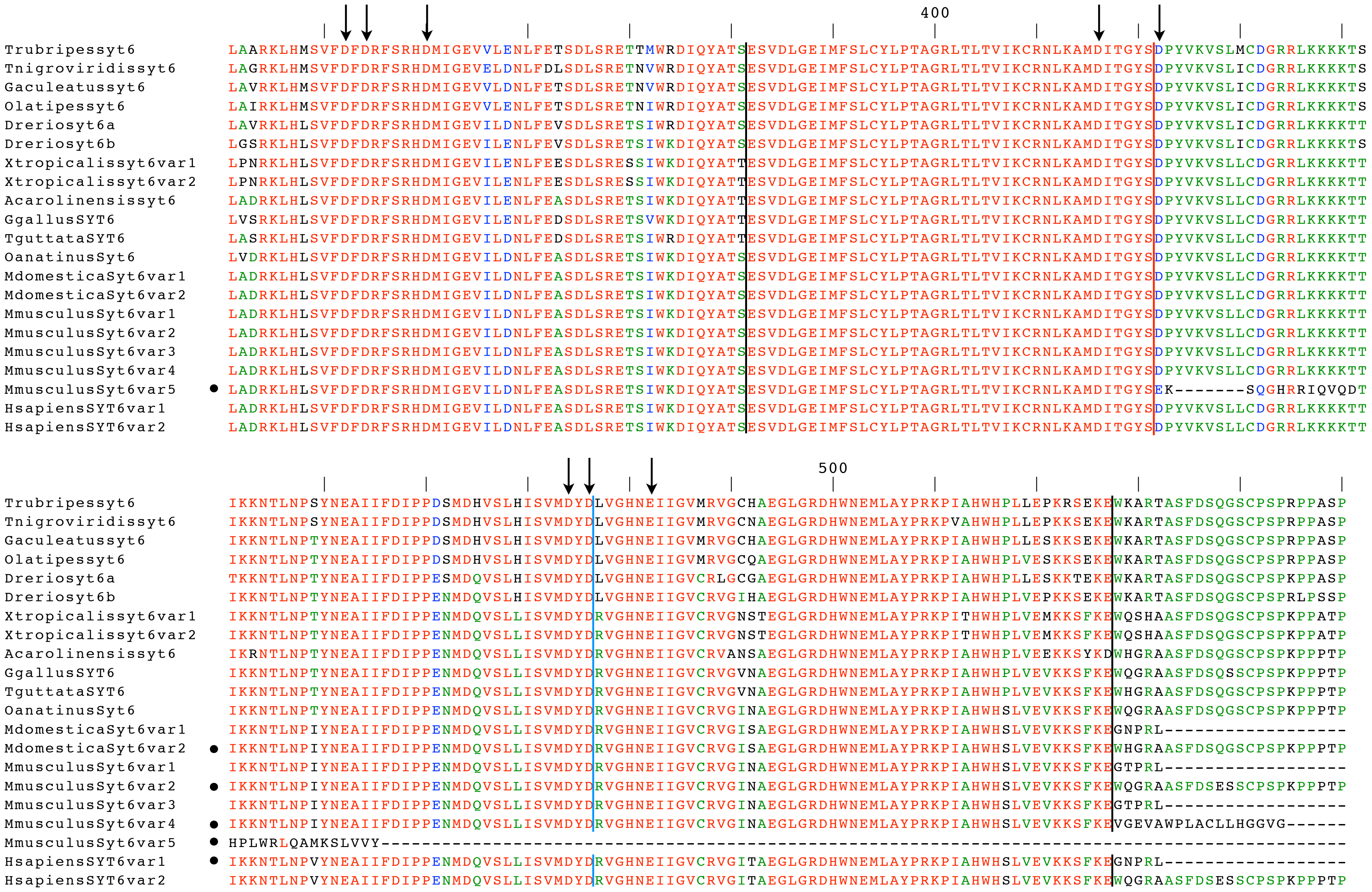
400
Trubripessyt6 LAARKLHMSVFDFDRFSRHDMIGEVVLENLFETSDLSRETTMWRDIQYATSESVDLGEIMFSLCYLPTAGRLTLTVIKCRNLKAMDITGYSDPYVKVSLMCDGRRLKKKKTS
Tnigroviridissyt6 LAGRKLHMSVFDFDRFSRHDMIGEVELDNLFDLSDLSRETNVWRDIQYATSESVDLGEIMFSLCYLPTAGRLTLTVIKCRNLKAMDITGYSDPYVKVSLICDGRRLKKKKTS
Gaculeatussyt6 LAVRKLHMSVFDFDRFSRHDMIGEVVLDNLFETSDLSRETNVWRDIQYATSESVDLGEIMFSLCYLPTAGRLTLTVIKCRNLKAMDITGYSDPYVKVSLICDGRRLKKKKTS
Olatipessyt6 LAIRKLHMSVFDFDRFSRHDMIGEVVLENLFETSDLSRETNIWRDIQYATSESVDLGEIMFSLCYLPTAGRLTLTVIKCRNLKAMDITGYSDPYVKVSLICDGRRLKKKKTS
Dreriosyt6a LAVRKLHLSVFDFDRFSRHDMIGEVILDNLFEVSDLSRETSIWRDIQYATSESVDLGEIMFSLCYLPTAGRLTLTVIKCRNLKAMDITGYSDPYVKVSLICDGRRLKKKKTT
Dreriosyt6b LGSRKLHLSVFDFDRFSRHDMIGEVILENLFEVSDLSRETSIWKDIQYATSESVDLGEIMFSLCYLPTAGRLTLTVIKCRNLKAMDITGYSDPYVKVSLICDGRRLKKKKTS
Xtropicalissyt6var1 LPNRKLHLSVFDFDRFSRHDMIGEVILENLFEESDLSRESSIWKDIQYATTESVDLGEIMFSLCYLPTAGRLTLTVIKCRNLKAMDITGYSDPYVKVSLLCDGRRLKKKKTT
Xtropicalissyt6var2 LPNRKLHLSVFDFDRFSRHDMIGEVILENLFEESDLSRESSIWKDIQYATTESVDLGEIMFSLCYLPTAGRLTLTVIKCRNLKAMDITGYSDPYVKVSLLCDGRRLKKKKTT
Acarolinensissyt6 LADRKLHLSVFDFDRFSRHDMIGEVILENLFEASDLSRETSIWKDIQYATTESVDLGEIMFSLCYLPTAGRLTLTVIKCRNLKAMDITGYSDPYVKVSLLCDGRRLKKKKTT
GgallusSYT6 LVSRKLHLSVFDFDRFSRHDMIGEVILENLFEDSDLSRETSVWKDIQYATTESVDLGEIMFSLCYLPTAGRLTLTVIKCRNLKAMDITGYSDPYVKVSLLCDGRRLKKKKTT
TguttataSYT6 LASRKLHLSVFDFDRFSRHDMIGEVILDNLFEDSDLSRETSIWRDIQYATTESVDLGEIMFSLCYLPTAGRLTLTVIKCRNLKAMDITGYSDPYVKVSLLCDGRRLKKKKTT
OanatinusSyt6 LVDRKLHLSVFDFDRFSRHDMIGEVILDNLFEASDLSRETSIWKDIQYATSESVDLGEIMFSLCYLPTAGRLTLTVIKCRNLKAMDITGYSDPYVKVSLLCDGRRLKKKKTT
MdomesticaSyt6var1 LADRKLHLSVFDFDRFSRHDMIGEVILDNLFEASDLSRETSIWKDIQYATSESVDLGEIMFSLCYLPTAGRLTLTVIKCRNLKAMDITGYSDPYVKVSLLCDGRRLKKKKTT
MdomesticaSyt6var2 LADRKLHLSVFDFDRFSRHDMIGEVILDNLFEASDLSRETSIWKDIQYATSESVDLGEIMFSLCYLPTAGRLTLTVIKCRNLKAMDITGYSDPYVKVSLLCDGRRLKKKKTT
MmusculusSyt6var1 LADRKLHLSVFDFDRFSRHDMIGEVILDNLFEASDLSRETSIWKDIQYATSESVDLGEIMFSLCYLPTAGRLTLTVIKCRNLKAMDITGYSDPYVKVSLLCDGRRLKKKKTT
MmusculusSyt6var2 LADRKLHLSVFDFDRFSRHDMIGEVILDNLFEASDLSRETSIWKDIQYATSESVDLGEIMFSLCYLPTAGRLTLTVIKCRNLKAMDITGYSDPYVKVSLLCDGRRLKKKKTT
MmusculusSyt6var3 LADRKLHLSVFDFDRFSRHDMIGEVILDNLFEASDLSRETSIWKDIQYATSESVDLGEIMFSLCYLPTAGRLTLTVIKCRNLKAMDITGYSDPYVKVSLLCDGRRLKKKKTT
MmusculusSyt6var4 LADRKLHLSVFDFDRFSRHDMIGEVILDNLFEASDLSRETSIWKDIQYATSESVDLGEIMFSLCYLPTAGRLTLTVIKCRNLKAMDITGYSDPYVKVSLLCDGRRLKKKKTT
MmusculusSyt6var5 ● LADRKLHLSVFDFDRFSRHDMIGEVILDNLFEASDLSRETSIWKDIQYATSESVDLGEIMFSLCYLPTAGRLTLTVIKCRNLKAMDITGYSEK-------SQGHRRIQVQDT
HsapiensSYT6var1 LADRKLHLSVFDFDRFSRHDMIGEVILDNLFEASDLSRETSIWKDIQYATSESVDLGEIMFSLCYLPTAGRLTLTVIKCRNLKAMDITGYSDPYVKVSLLCDGRRLKKKKTT
HsapiensSYT6var2 LADRKLHLSVFDFDRFSRHDMIGEVILDNLFEASDLSRETSIWKDIQYATSESVDLGEIMFSLCYLPTAGRLTLTVIKCRNLKAMDITGYSDPYVKVSLLCDGRRLKKKKTT
500
Trubripessyt6 IKKNTLNPSYNEAIIFDIPPDSMDHVSLHISVMDYDLVGHNEIIGVMRVGCHAEGLGRDHWNEMLAYPRKPIAHWHPLLEPKRSEKEWKARTASFDSQGSCPSPRPPASP
Tnigroviridissyt6 IKKNTLNPSYNEAIIFDIPPDSMDHVSLHISVMDYDLVGHNEIIGVMRVGCNAEGLGRDHWNEMLAYPRKPVAHWHPLLEPKKSEKEWKARTASFDSQGSCPSPRPPASP
Gaculeatussyt6 IKKNTLNPTYNEAIIFDIPPDSMDHVSLHISVMDYDLVGHNEIIGVMRVGCHAEGLGRDHWNEMLAYPRKPIAHWHPLLESKKSEKEWKARTASFDSQGSCPSPRPPASP
Olatipessyt6 IKKNTLNPTYNEAIIFDIPPDSMDHVSLHISVMDYDLVGHNEIIGVMRVGCQAEGLGRDHWNEMLAYPRKPIAHWHPLVESKKSEKEWKARTASFDSQGSCPSPRPPASP
Dreriosyt6a TKKNTLNPTYNEAIIFDIPPESMDQVSLHISVMDYDLVGHNEIIGVCRLGCGAEGLGRDHWNEMLAYPRKPIAHWHPLLESKKTEKEWKARTASFDSQGSCPSPKPPASP
Dreriosyt6b IKKNTLNPTYNEAIIFDIPPENMDQVSLHISVMDYDLVGHNEIIGVCRVGIHAEGLGRDHWNEMLAYPRKPIAHWHPLVEPKKSEKEWKARTASFDSQGSCPSPRLPSSP
Xtropicalissyt6var1 IKKNTLNPTYNEAIIFDIPPENMDQVSLLISVMDYDRVGHNEIIGVCRVGNSTEGLGRDHWNEMLAYPRKPITHWHPLVEMKKSFKEWQSHAASFDSQGSCPSPKPPATP
Xtropicalissyt6var2 IKKNTLNPTYNEAIIFDIPPENMDQVSLLISVMDYDRVGHNEIIGVCRVGNSTEGLGRDHWNEMLAYPRKPITHWHPLVEMKKSFKEWQSHAASFDSQGSCPSPKPPATP
Acarolinensissyt6 IKRNTLNPTYNEAIIFDIPPENMDQVSLLISVMDYDRVGHNEIIGVCRVANSAEGLGRDHWNEMLAYPRKPIAHWHPLVEEKKSYKDWHGRAASFDSQGSCPSPKPPPTP
GgallusSYT6 IKKNTLNPTYNEAIIFDIPPENMDQVSLLISVMDYDRVGHNEIIGVCRVGVNAEGLGRDHWNEMLAYPRKPIAHWHPLVEVKKSFKEWQGRAASFDSQSSCPSPKPPPTP
TguttataSYT6 IKKNTLNPTYNEAIIFDIPPENMDQVSLLISVMDYDRVGHNEIIGVCRVGVNAEGLGRDHWNEMLAYPRKPIAHWHPLVEVKKSFKEWHGRAASFDSQGSCPSPKPPPTP
OanatinusSyt6 IKKNTLNPTYNEAIIFDIPPENMDQVSLLISVMDYDRVGHNEIIGVCRVGINAEGLGRDHWNEMLAYPRKPIAHWHSLVEVKKSFKEWQGRAASFDSQGSCPSPKPPPTP
MdomesticaSyt6var1 IKKNTLNPIYNEAIIFDIPPENMDQVSLLISVMDYDRVGHNEIIGVCRVGISAEGLGRDHWNEMLAYPRKPIAHWHSLVEVKKSFKEGNPRL------------------
MdomesticaSyt6var2 ● IKKNTLNPIYNEAIIFDIPPENMDQVSLLISVMDYDRVGHNEIIGVCRVGISAEGLGRDHWNEMLAYPRKPIAHWHSLVEVKKSFKEWHGRAASFDSQGSCPSPKPPPTP
MmusculusSyt6var1 IKKNTLNPIYNEAIIFDIPPENMDQVSLLISVMDYDRVGHNEIIGVCRVGINAEGLGRDHWNEMLAYPRKPIAHWHSLVEVKKSFKEGTPRL------------------
MmusculusSyt6var2 ● IKKNTLNPIYNEAIIFDIPPENMDQVSLLISVMDYDRVGHNEIIGVCRVGINAEGLGRDHWNEMLAYPRKPIAHWHSLVEVKKSFKEWQGRAASFDSESSCPSPKPPPTP
MmusculusSyt6var3 IKKNTLNPIYNEAIIFDIPPENMDQVSLLISVMDYDRVGHNEIIGVCRVGINAEGLGRDHWNEMLAYPRKPIAHWHSLVEVKKSFKEGTPRL------------------
MmusculusSyt6var4 ● IKKNTLNPIYNEAIIFDIPPENMDQVSLLISVMDYDRVGHNEIIGVCRVGINAEGLGRDHWNEMLAYPRKPIAHWHSLVEVKKSFKEVGEVAWPLACLLHGGVG------
MmusculusSyt6var5 ● HPLWRLQAMKSLVVY---
HsapiensSYT6var1 ● IKKNTLNPVYNEAIIFDIPPENMDQVSLLISVMDYDRVGHNEIIGVCRVGITAEGLGRDHWNEMLAYPRKPIAHWHSLVEVKKSFKEGNPRL------------------
HsapiensSYT6var2 IKKNTLNPVYNEAIIFDIPPENMDQVSLLISVMDYDRVGHNEIIGVCRVGITAEGLGRDHWNEMLAYPRKPIAHWHSLVEVKKSFKEWQGRAASFDSESSCPSPKPPPTP
