# Supplementary material for: A manual collection of Syt, Esyt, Rph3a, Rph3al, Doc2, and Dblc2 genes from 46 metazoan genomes - an open access resource for neuroscience and evolutionary biology
Source: BMC Genomics. 2010 Jan 15;11:37. doi: 10.1186/1471-2164-11-37 (PMC2823689; doi:10.1186/1471-2164-11-37)
Supplement: Additional file 23 — Alignment of the vertebrate Syt10 sequences. Amino acid position is marked every hundred amino acids approximately, at the top of each page of the alignment. Intron position and phase is indicated with a coloured bar between amino acids. Black bars indicate phase 0 introns. Red bars indicate phase +1 introns. Blue bars indicate phase +2 introns. The widely conserved motif of unknown function, just upstream of the C2A domain, is indicated. The five conserved acidic amino acids in each C2 domain are indicated by black arrows at the top of the alignment. [file 1471-2164-11-37-S23.PDF]

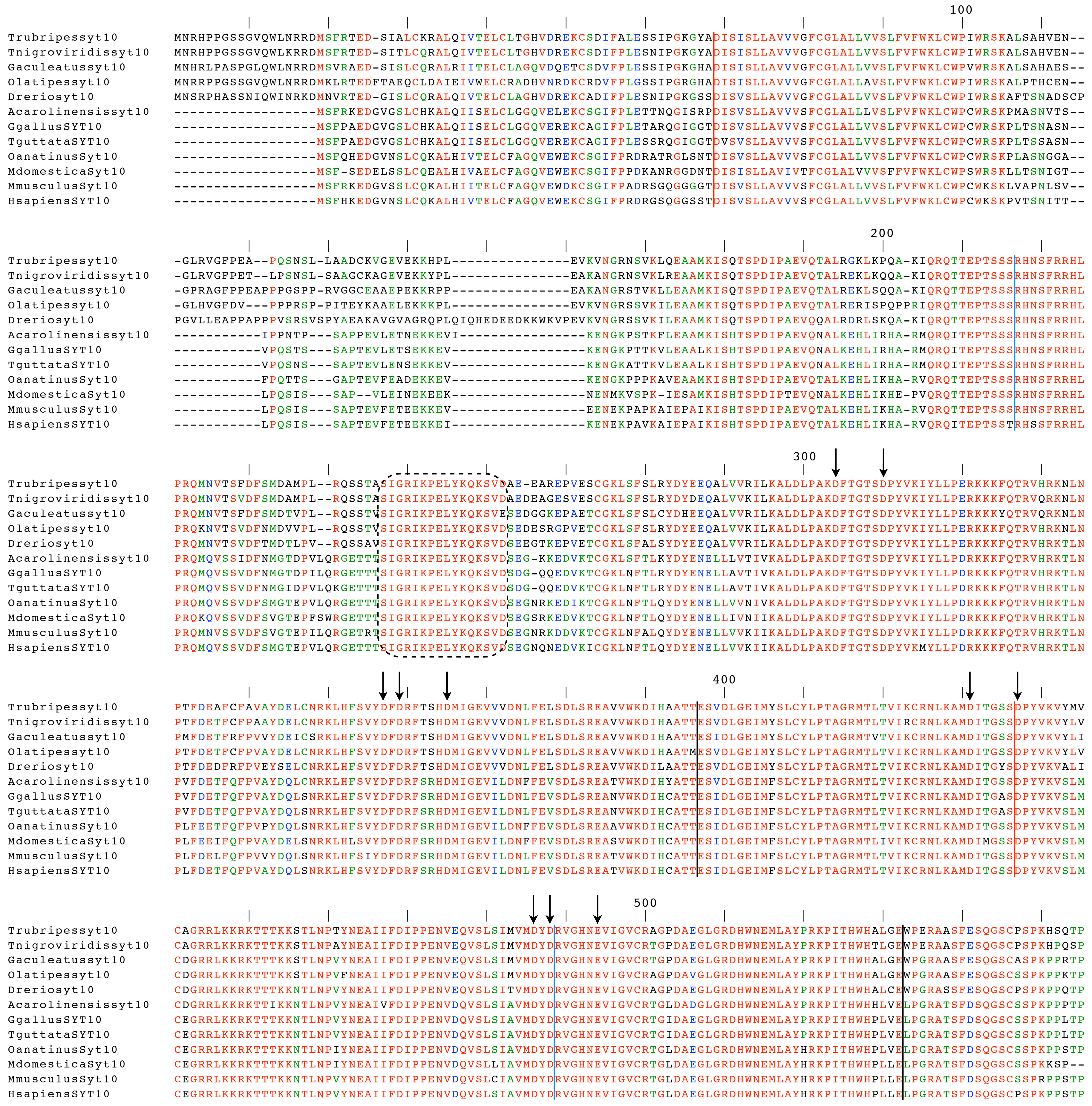
100
Trubripessyt10 MNRHPPGSSGVQWLNRRDMSFRTED-SIALCKRALQIVTELCLTGHVDREKCSDIFALESSIPGKGYADISISLLAVVVGFCGLALLVVSLFVFWKLCWPIWRSKALSAHVEN--
Tnigroviridissyt10 MNRHPPGSSGVQWLKRRDMSFRTED-SITLCQRALQIVTELCLTGHVDREKCSDIFPLESNIPGKGYADISISLLAVVVGFCGLALLVVSLFVFWKLCWPIWRSKALSAHVEN--
Gaculeatussyt10 MNHRLPASPGLQWLNRRDMSVRAED-SISLCQRALRIITELCLAGQVDQETCSDVFPLESSIPGKGHADISISLLAVVVGFCGLALLVVSLFVFWKLCWPVWRSKALSAHAES--
Olatipessyt10 MNRRPPGSSGVQWLNRRDMKLRTEDFTAEQCLDAIEIVWELCRADHVNRDKCRDVFPLGSSIPGRGHADISISLLAVVVGFCGLALLAVSLFVFWKLCWPIWRSKALPTHCEN--
Dreriosyt10 MNSRPHASSNIQWINRKDMNVRTED-GISLCQRALQIVTELCLAGHVDREKCADIFPLESNIPGKGSSDISVSLLAVVVGFCGLALLVVSLFVFWKLCWPIWRSKAFTSNADSCP
Acarolinensissyt10 ------------------MSFRKEDGVGSLCHKALQIISELCLGGQVELEKCSGIFPLETTNQGISRPDISVSLLAVVVSFCGLALLLVSLFVFWKLCWPCWRSKPMASNVTS--
GgallusSYT10 ------------------MSFPAEDGVGSLCHKALQIISELCLGGQVEREKCAGIFPLETARQGIGGTDISVSLLAVVVSFCGLALLVVSLFVFWKLCWPCWRSKPLTSNASN--
TguttataSYT10 ------------------MSFPAEDGVGSLCHKALQIISELCLGGQVEREKCAGIFPLESSRQGIGGTDVSVSLLAVVVSFCGLALLVVSLFVFWKLCWPCWRSKPLTSSASN--
OanatinusSyt10 ------------------MSFQHEDGVNSLCQKALHIVTELCFAGQVEWEKCSGIFPRDRATRGLSNTDISVSLLAVVVSFCGLALLVVSLFVFWKLCWPCWRSKPLASNGGA--
MdomesticaSyt10 ------------------MSF-SEDELSSLCQEALHIVAELCFAGQVEWEKCSGIFPPDKANRGGDNTDISISLLAVIVTFCGLALVVVSFFVFWKLCWPSWRSKLLTSNIGT--
MmusculusSyt10 ------------------MSFRKEDGVSSLCQKALHIITELCFAGQVEWDKCSGIFPADRSGQGGGGTDISVSLLAVVVSFCGLALLVVSLFVFWKLCWPCWKSKLVAPNLSV--
HsapiensSYT10 ------------------MSFHKEDGVNSLCQKALHIVTELCFAGQVEWEKCSGIFPRDRGSQGGSSTDISVSLLAVVVSFCGLALLVVSLFVFWKLCWPCWKSKPVTSNITT--
200
Trubripessyt10 -GLRVGFPEA--PQSNSL-LAADCKVGEVEKKHPL---------------EVKVNGRNSVKLQEAAMKISQTSPDIPAEVQTALRGKLKPQA-KIQRQTTEPTSSSRHNSFRRHL
Tnigroviridissyt10 -GLRVGFPET--LPSNSL-SAAGCKAGEVEKKYPL---------------EAKANGRNSVKLREAAMKISQTSPDIPAEVQTALREKLKQQA-KIQRQTTEPTSSSRHNSFRRHL
Gaculeatussyt10 -GPRAGFPPEAPPPGSPP-RVGGCEAAEPEKKRPP---------------EAKANGRSTVKLLEAAMKISQTSPDIPAEVQTALREKLSQQA-KIQRQTTEPTSSSRHNSFRRHL
Olatipessyt10 -GLHVGFDV---PPPRSP-PITEYKAAELEKKKPL---------------EVKVNGRSSVKILEAAMKISQTSPDIPAEVQTALRERISPQPPRIQRQTTEPTSSSRHNSFRRHL
Dreriosyt10 PGVLLEAPPAPPPVSRSVSPYAEAKAVGVAGRQPLQIQHEDEEDKKWKVPEVKVNGRSSVKILEAAMKISQTSPDIPAEVQQALRDRLSKQA-KIQRQTTEPTSSSRHNSFRRHL
Acarolinensissyt10 -----------IPPNTP---SAPPEVLETNEKKEVI----------------KENGKPSTKFLEAAMKISHTSPDIPAEVQNALKEHLIRHA-RMQRQITEPTSSSRHNSFRRHL
GgallusSYT10 -----------VPQSTS---SAPTEVLETSEKKEV-----------------KENGKPTTKVLEAALKISHTSPDIPAEVQNALKEHLIRHA-RMQRQITEPTSSSRHNSFRRHL
TguttataSYT10 -----------VPQSNS---SAPTEVLENSEKKEV-----------------KENGKATTKVLEAALKISHTSPDIPAEVQNALKEHLIRHA-RMQRQITEPTSSSRHNSFRRHL
OanatinusSyt10 -----------FPQTTS---GAPTEVFEADEKKEV-----------------KENGKPPPKAVEAAMKISHTSPDIPAEVQTALKEHLIKHA-RVQRQTTEPTSSSRHNSFRRHL
MdomesticaSyt10 -----------LPQSIS---SAP--VLEINEKEEK-----------------NENMKVSPK-IESAMKISHTSPDIPTEVQNALKEHLIKHE-PVQRQTTEPTSSSRHNSFRRHL
MmusculusSyt10 -----------LPQSIS---SAPTEVFETEEKKEV-----------------EENEKPAPKAIEPAIKISHTSPDIPAEVQTALKEHLIKHA-RVQRQTTEPTSSSRHNSFRRHL
HsapiensSYT10 -----------LPQSIS---SAPTEVFETEEKKEI-----------------KENEKPAVKAIEPAIKISHTSPDIPAEVQTALKEHLIKHA-RVQRQITEPTSSTRHSSFRRHL
300
Trubripessyt10 PRQMNVTSFDFSMDAMPL--RQSSTASIGRIKPELYKQKSVDAE-EAREPVESCGKLSFSLRYDYEEQALVVRILKALDLPAKDFTGTSDPYVKIYLLPERKKKFQTRVHRKNLN
Tnigroviridissyt10 PRQMNVTSVDFSMDAMPL--RQSSTASIGRIKPELYKQKSVDAEDEAGESVESCGKLSFSLRYDYDEQALVVKILKALDLPAKDFTGTSDPYVKIYLLPERKKKFQTRVQRKNLN
Gaculeatussyt10 PRQMNVTSFDFSMDTVPL--RQSSTVSIGRIKPELYKQKSVESEDGGKEPAETCGKLSFSLCYDHEEQALVVRILKALDLPAKDFTGTSDPYVKIYLLPERKKKYQTRVQRKNLN
Olatipessyt10 PRQKNVTSVDFNMDVVPL--RQSSTVSIGRIKPELYKQKSVDSEDESRGPVETCGKLSFSLRYDYEEQALVVKILKALDLPAKDFTGTSDPYVKIYLLPERKKKFQTRVHRKNLN
Dreriosyt10 PRQMNVTSVDFTMDTLPV--RQSSAVSIGRIKPELYKQKSVDSEEGTKEPVETCGKLSFALSYDYEEQALVVRILKALDLPAKDFTGTSDPYVKIYLLPERKKKFQTRVHRKTLN
Acarolinensissyt10 PRQMQVSSIDFNMGTDPVLQRGETTTSIGRIKPELYKQKSVDSEG-KKEDVKTCGKLSFTLKYDYENELLLVTIVKALDLPAKDFTGTSDPYVKIYLLPDRKKKFQTRVHRKTLN
GgallusSYT10 PRQMQVSSVDFNMGTDPILQRGETTTSIGRIKPELYKQKSVDSDG-QQEDVKTCGKLNFTLRYDYENELLAVTIVKALDLPAKDFTGTSDPYVKIYLLPDRKKKFQTRVHRKTLN
TguttataSYT10 PRQMQVSSVDFNMGIDPVLQKGETTTSIGRIKPELYKQKSVDSDG-QQEDVKTCGKLNFTLRYDYENELLAVTIVKALDLPAKDFTGTSDPYVKIYLLPDRKKKFQTRVHRKTLN
OanatinusSyt10 PRQMQVSSVDFSMGTEPVLQRGETTTSIGRIKPELYKQKSVDSEGNRKEDIKTCGKLNFTLQYDYENELLVVNIVKALDLPAKDFTGTSDPYVKIYLLPDRKKKFQTRVHRKTLN
MdomesticaSyt10 PRQKQVSSVDFSVGTEPFSWRGETTTSIGRIKPELYKQKSVDSEGSRKEDVKTCGKLNFTLQYDYENELLIVNIIKALDLPAKDFTGTSDPYVKIYLLPDRKKKFQTRVHRKTLN
MmusculusSyt10 PRQMNVSSVDFSVGTEPILQRGETRTSIGRIKPELYKQKSVDSEGNRKDDVKTCGKLNFALQYDYENELLVVKIIKALDLPAKDFTGTSDPYVKIYLLPDRKKKFQTRVHRKTLN
HsapiensSYT10 PRQMQVSSVDFSMGTEPVLQRGETTTSIGRIKPELYKQKSVDSEGNQNEDVKICGKLNFTLQYDYENELLVVKIIKALDLPAKDFTGTSDPYVKMYLLPDRKKKFQTRVHRKTLN
400
Trubripessyt10 PTFDEAFCFAVAYDELCNRKLHFSVYDFDRFTSHDMIGEVVVDNLFELSDLSREAVVWKDIHAATTESVDLGEIMYSLCYLPTAGRMTLTVIKCRNLKAMDITGSSDPYVKVYMV
Tnigroviridissyt10 PTFDETFCFPAAYDELCNRKLHFSVYDFDRFTSHDMIGEVVVDNLFELSDLSREAVVWKDIHAATTESVDLGEIMYSLCYLPTAGRMTLTVIRCRNLKAMDITGSSDPYVKVYLV
Gaculeatussyt10 PMFDETFRFPVVYDEICSRKLHFSVYDFDRFTSHDMIGEVVVDNLFELSDLSREAVVWKDIHAATTESIDLGEIMYSLCYLPTAGRMTVTVIKCRNLKAMDITGSSDPYVKVYLI
Olatipessyt10 PTFDETFCFPVAYDELCNRKLHFSVYDFDRFTSHDMIGEVVVDNLFELSDLSREAVVWKDIHAATMESVDLGEIMYSLCYLPTAGRMTLTVIKCRNLKAMDITGSSDPYVKVYLV
Dreriosyt10 PTFDEDFRFPVEYSELCNRKLHFSVYDFDRFTSHDMIGEVVVDNLFELSDLSREAVVWKDILAATTESVDLGEIMYSLCYLPTAGRMTLTVIKCRNLKAMDITGYSDPYVKVALI
Acarolinensissyt10 PVFDETFQFPVAYDQLCNRKLHFSVYDFDRFSRHDMIGEVILDNFFEVSDLSREATVWKDIHYATTESVDLGEIMFSLCYLPTAGRMTLTVIKCRNLKAMDITGSSDPYVKVSLM
GgallusSYT10 PVFDETFQFPVAYDQLSNRKLHFSVYDFDRFSRHDMIGEVILDNLFEVSDLSREANVWKDIHCATTESIDLGEIMFSLCYLPTAGRMTLTVIKCRNLKAMDITGASDPYVKVSLM
TguttataSYT10 PVFDETFQFPVAYDQLSNRKLHFSVYDFDRFSRHDMIGEVILDNLFEVSDLSREANVWKDIHCATTESIDLGEIMFSLCYLPTAGRMTLTVIKCRNLKAMDITGASDPYVKVSLM
OanatinusSyt10 PLFEETFQFPVPYDQLSNRKLHFSVYDFDRFSRHDMIGEVILDNFFEVSDLSREAAVWKDIHCATTESIDLGEIMFSLCYLPTAGRMTLTVIKCRNLKAMDITGSSDPYVKVSLM
MdomesticaSyt10 PLFEEIFQFPVAYDELSNRKLHLSVYDFDRFSRHDMIGEVILDNFFEVSDLSREASVWKDIHCATTESIDLGEIMFSLCYLPTAGRMTLIVIKCRNLKAMDIMGSSDPYVKVSLM
MmusculusSyt10 PLFDELFQFPVVYDQLSNRKLHFSIYDFDRFSRHDMIGEVILDNLFEVSDLSREATVWKDIHCATTESIDLGEIMFSLCYLPTAGRMTLTVIKCRNLKAMDITGSSDPYVKVSLM
HsapiensSYT10 PLFDETFQFPVAYDQLSNRKLHFSVYDFDRFSRHDMIGEVILDNLFEVSDLSREATVWKDIHCATTESIDLGEIMFSLCYLPTAGRMTLTVIKCRNLKAMDITGSSDPYVKVSLM
500
Trubripessyt10 CAGRRLKKRKTTTKKSTLNPTYNEAIIFDIPPENVEQVSLSIMVMDYDRVGHNEVIGVCRAGPDAEGLGRDHWNEMLAYPRKPITHWHALGEWPERAASFESQGSCPSPKHSQTP
Tnigroviridissyt10 CAGRRLKKRKTTTKKSTLNPAYNEAIIFDIPPENVEQVSLSIMVMDYDRVGHNEVIGVCRTGPDAEGLGRDHWNEMLAYPRKPITHWHTLGEWPERAASFESQGSCPSPKHPQSP
Gaculeatussyt10 CDGRRLKKRKTTTKKSTLNPVYNEAIIFDIPPENVEQVSLSIMVMDYDRVGHNEVIGVCRTGPDAEGLGRDHWNEMLAYPRKPITHWHALGEWPGRAASFESQGSCASPKPPRTP
Olatipessyt10 CDGRRLKKRKTTTKKSTLNPVFNEAIIFDIPPENVEQVSLSIMVMDYDRVGHNEVIGVCRAGPDAVGLGRDHWNEMLAYPRKPITHWHALGEWPGRAASFESQGSCSSPKPPKTP
Dreriosyt10 CDGRRLKKRKTTTKKNTLNPVYNEAIIFDIPPENVEQVSLSITVMDYDRVGHNEVIGVCRAGPDAEGLGRDHWNEMLAYPRKPITHWHALCEWPGRASSFESQGSCPSPKPPQTP
Acarolinensissyt10 CDGRRLKKRKTTIKKNTLNPVYNEAIVFDIPPENVDQVSLSIAVMDYDRVGHNEVIGVCRTGLDADGLGRDHWNEMLAYPRKPITHWHHLVELPGRATSFDSQGSCSSPKPPPTP
GgallusSYT10 CEGRRLKKRKTTTKKNTLNPVYNEAIIFDIPPENVDQVSLSIAVMDYDRVGHNEVIGVCRTGIDAEGLGRDHWNEMLAYPRKPITHWHPLVELPGRATSFDSQGSCSSPKPPLTP
TguttataSYT10 CEGRRLKKRKTTTKKNTLNPVYNEAIIFDIPPENVDQVSLSIAVMDYDRVGHNEVIGVCRTGIDAEGLGRDHWNEMLAYPRKPITHWHPLVELPGRATSFDSQGSCSSPKPPLTP
OanatinusSyt10 CEGRRLKKRKTTTKKNTLNPIYNEAIIFDIPPENVDQVSLSIAVMDYDRVGHNEVIGVCRTGLDAEGLGRDHWNEMLAYHRKPITHWHPLVELPGRATSFDSQGSCSSPKPPSTP
MdomesticaSyt10 CEGRRLKKRKTTTKKNTLNPIYNEAIIFDIPPENVDQVSLLIAVMDYDRVGHNEVIGVCRTGLDAEGLGRDHWNEMLAYHRKPITHWHPLLELPGRATSFDSQGSCSSPKKSP--
MmusculusSyt10 CEGRRLKKRKTTTKKNTLNPVYNEAIIFDIPPENVDQVSLCIAVMDYDRVGHNEVIGVCRTGLDAEGLGRDHWNEMLAYHRKPITHWHPLLELPGRATSFDSQGSCSSPRPPSTP
HsapiensSYT10 CEGRRLKKRKTTTKKNTLNPVYNEAIIFDIPPENVDQVSLSIAVMDYDRVGHNEVIGVCRTGLDAEGLGRDHWNEMLAYHRKPITHWHPLLELPGRATSFDSQGSCPSPKPPSTP
