# Supplementary material for: A manual collection of Syt, Esyt, Rph3a, Rph3al, Doc2, and Dblc2 genes from 46 metazoan genomes - an open access resource for neuroscience and evolutionary biology
Source: BMC Genomics. 2010 Jan 15;11:37. doi: 10.1186/1471-2164-11-37 (PMC2823689; doi:10.1186/1471-2164-11-37)
Supplement: Additional file 24 — Alignment of the invertebrate Syt12 sequences. Amino acid position is marked every hundred amino acids approximately, at the top of each page of the alignment. Intron position and phase is indicated with a coloured bar between amino acids. Black bars indicate phase 0 introns. Red bars indicate phase +1 introns. Blue bars indicate phase +2 introns. Because of their differing positions, TM domains are highlighted in blue. X residues indicate where a portion of sequence is missing. [file 1471-2164-11-37-S24.PDF]

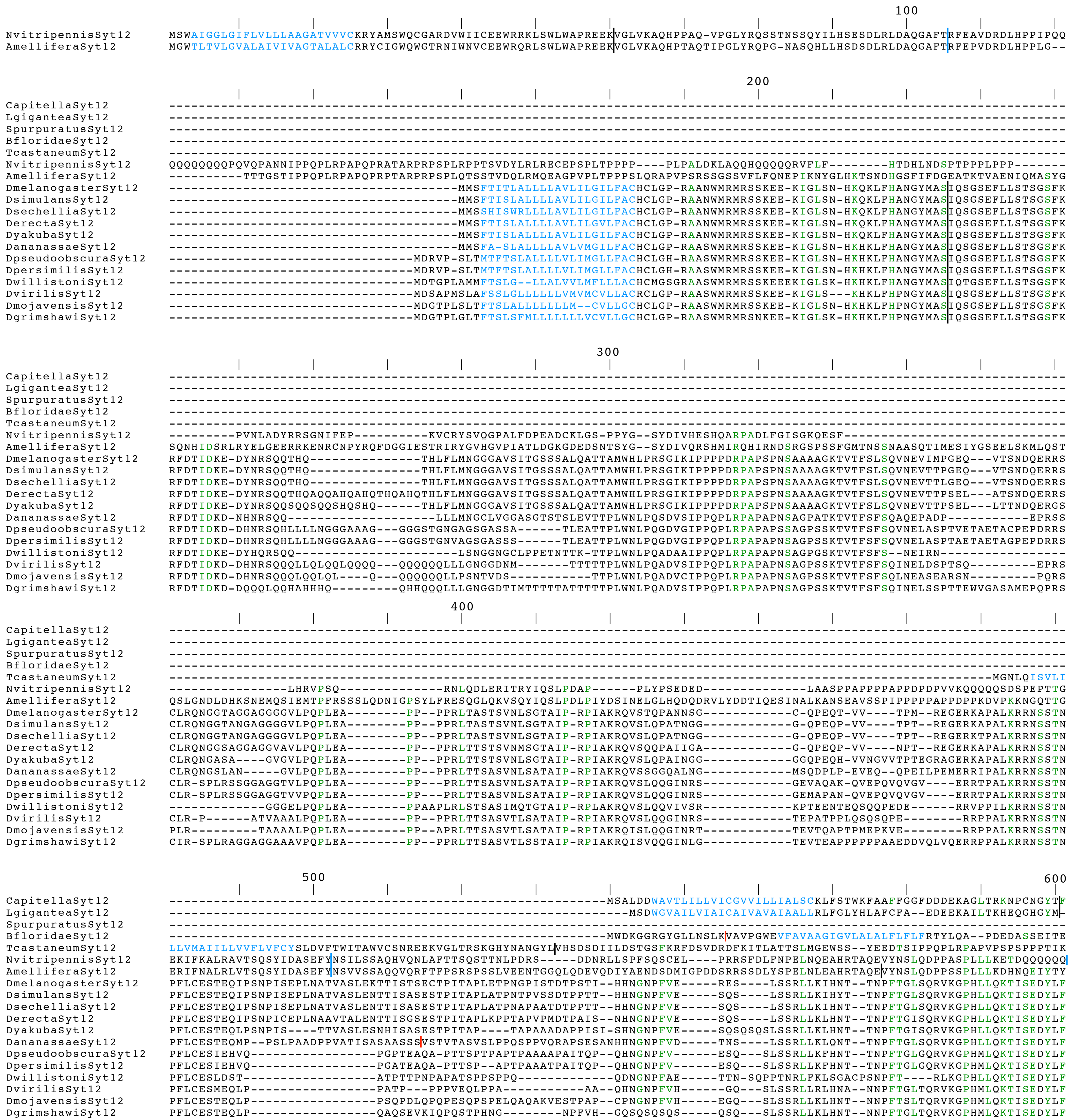
100
NvitripennisSyt12
AmelliferaSyt12
200
CapitellaSyt12
LgiganteaSyt12
SpurpuratusSyt12
BfloridaeSyt12
TcastaneumSyt12
NvitripennisSyt12
AmelliferaSyt12
DmelanogasterSyt12
DsimulansSyt12
DsechelliaSyt12
DerectaSyt12
DyakubaSyt12
DananassaeSyt12
DpseudoobscuraSyt12
DpersimilisSyt12
DwillistoniSyt12
DvirilisSyt12
DmojavensisSyt12
DgrimshawiSyt12
300
400
500
600

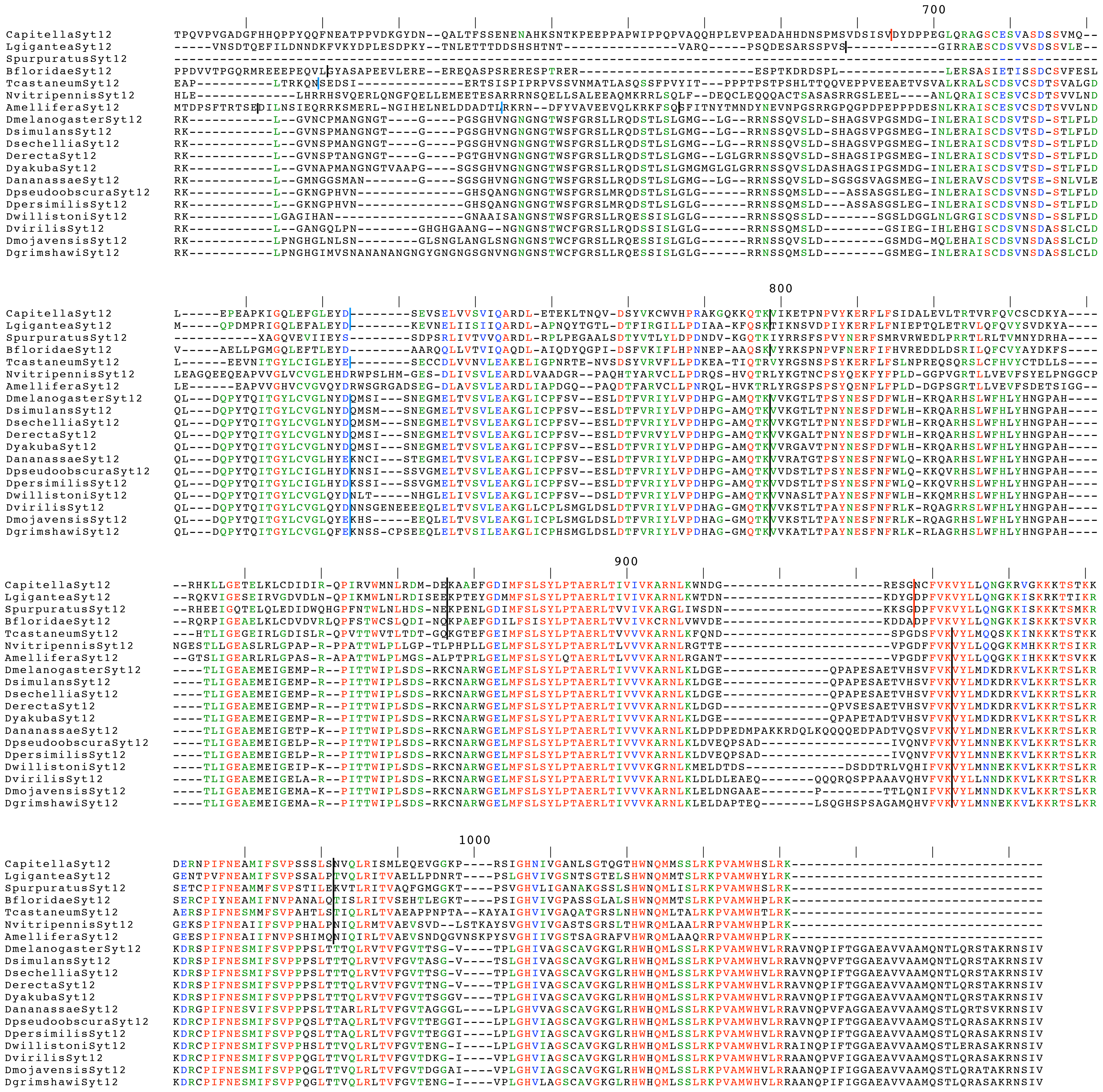
700
800
900
1000
CapitellaSyt12
LgiganteaSyt12
SpurpuratusSyt12
BfloridaeSyt12
TcastaneumSyt12
NvitripennisSyt12
AmelliferaSyt12
DmelanogasterSyt12
DsimulansSyt12
DsecheliaSyt12
DerectaSyt12
DyakubaSyt12
DananassaeSyt12
DpseudoobscuraSyt12
DpersimilisSyt12
DwillistoniSyt12
DvirilisSyt12
DmojavensisSyt12
DgrimshawiSyt12
