# Supplementary material for: A manual collection of Syt, Esyt, Rph3a, Rph3al, Doc2, and Dblc2 genes from 46 metazoan genomes - an open access resource for neuroscience and evolutionary biology
Source: BMC Genomics. 2010 Jan 15;11:37. doi: 10.1186/1471-2164-11-37 (PMC2823689; doi:10.1186/1471-2164-11-37)
Supplement: Additional file 25 — Alignment of the vertebrate Syt12 sequences. Amino acid position is marked every hundred amino acids approximately, at the top of each page of the alignment. The H. sapiens splice variant is included and highlighted with a black dot where it differs. Intron position and phase is indicated with a coloured bar between amino acids. Black bars indicate phase 0 introns. Red bars indicate phase +1 introns. X residues indicate where a portion of sequence is missing. [file 1471-2164-11-37-S25.PDF]

```
                                                                                                   100
Trubripessyt12          -------------------------------MSSPQSGDISGYHLSVVRNPPGWEVGIYLAGFFVLLAVAGLNIWKLWKSGTFPTPSPFPNFDYRYLQEKYGTSFSEVRQKRVAANNH
Tnigroviridissyt12      -------------------------------MSSPQSGDISGYHLSVVRNPPGWEVGIYLAGFFVLLAVAGLNIWKLWKSGRFPTPSPFPNFDYRYLQEKYGTSFSEVRQKRVAANNH
Gaculeatussyt12         -------------------------------MSSALSEDVSGYHLSVVRNPPGWEVGIYLVGFFVLLCVAGINIWKLWKSGTFPAPSPFPNFDYRYLQEKYGTSFSEVRQKRVAANNH
Olatipessyt12           -------------------------------MSSAQSGDISSYQLSVVRNPPGWEVGIYLVGFLVLLGAAGLNIWKLWKSGTFPAPSPFPNFDYRYLQEKYGTSFSEVRQKRVAASNH
Dreriosyt12             --------------------------------MSVQGQDISEYHLSVVLNPPGWEVCLFVFGFLVLFAVVIVNLWRLYKSGTFPTPSPFPNFHYRYLQEKYGSSHSEVRQKRVAACNQ
Xtropicalissyt12        ----------------------------------------------XIRSPPGWEIGIYIAGALTLLGVTGLYLWKLYKSGNYPSPSPFPNYDYRYLKQKYGTSHSDVRKKRQPA---
Acarolinensussyt12      ----------------------------------------------XIKNPPGWEIGVYITGALALLGIAGVNLWKLWRSGSYPAPSPFPNYDYRYLEQKYGTTYSQIKQKRA-ANNF
GgallusSYT12            ---------------------------------------MGRYRLSAAASPPRWEVGIYAAGALALLGIAAVNLWKLWRSGSYPAPSPFPNYDYRYLEQKYGTAYSDIKHKHG-A---
TguttataSYT12           ----------------------------------MDTGHVSRSRFSVASSPPRWEIGLYAAGALALLGIAAINLWKLWRSGSYPAPSPFPNYDYRYLEQKYGAACPDIRNKR------
OanatinusSyt12          ----------------------------------------------XIKSPPGWEVGIYVTGALVLLGIAAVNLWKLWKSGSFPIPSPFPNYDYRYLQQKYGMTYSETRRKRSTAWAP
MdomesticaSyt12         -----------------------------------MAVDVTEYHLSVIKSPPGWEIGIYVTGALALLGVAAVNLWRLCKSGSFPIPSPFPNYDYRYLQKKYGVTYSEARQKRTMAWSS
MmusculusSyt12          -----------------------------------MAVDVTEYHLSVIKSPPGWEVGVYAAGALALLGIAAVSLWKLWTSGSFPSPSPFPNYDYRYLQQKYGEAYVEAKLKRVPPWND
HsapiensSYT12var1     ● MSHNTQTNIIFHSPGNEMKTKAPPVGEVTVTAADIMAVDVAEYHLSVIKSPPGWEVGVYAAGALALLGIAAVSLWKLWTSGSFPSPSPFPNYDYRYLQQKYGESCAEAREKRVPAWNA
HsapiensSYT12var2       -----------------------------------MAVDVAEYHLSVIKSPPGWEVGVYAAGALALLGIAAVSLWKLWTSGSFPSPSPFPNYDYRYLQQKYGESCAEAREKRVPAWNA

                                                                                         200
Trubripessyt12          RRTSA--TSSRKPSLALCDTPDAFRDLGHLELMSRELDPTGMAQLNRSISTDSLSSIS--------SIANNFGHDFTVGQLEVTLEFEPSRHPNQGTGLLHMTLHQGKDLLE-KEEGD
Tnigroviridissyt12      RRTST--TSSRKPSLALCDTPDGFRDLGHLELMSRELDPTGAAQLNRSISTDSLSSIS--------SIANNFGHDFTVGQLEVTLEFEPFRHRGQGAGLLHVTLHQGKDLLE-KEEGD
Gaculeatussyt12         RRTST--TSSRKPSLALGDTPDCFRDLGHLELMSRELDPTGVAQLNRSVSTDSLSSIS--------SIANNFGHDFTVGQLEVTLEFEPSRHAGQGPGLLHIALHQGKDLLE-REEGD
Olatipessyt12           RRTST--TSSRKPSLALGDAPDAFRDLGHLELMSRELDATGLAQLNRSVSTDSLSSIS--------SIAHNFGHDFTVGQLEVTLEFEPSRQPGHGPGLLHISLHQGKDLLE-KEEGD
Dreriosyt12             RRASS---ASRKPSLQLLDTPDGLRDLGTLELMSRELDQSG-GSLNRSVSSESLCSIS--------SVAQTFGHDFTVGQLEVTLELDTR------ASLLLVALHQGKDLLE-KEEEN
Xtropicalissyt12        -------AKPANNMANLNDTYESINEFGPIELINKDLEMTPYGMLRKSISTDSLSSVS--------SIGNNFGQDITVGQIEVTMDYDVH------SNTLHVALIQGKDLLERKEDGN
Acarolinensussyt12      RKAGDRTSPVRKPSLKMDDTFENINELGTLELMHKELDLAPYGPLKKSISTDSLSSIS--------SIGNNFGQEFTVGQVEVNMEYNVH------SNTLHVTLLQGKDLME-KEDIN
GgallusSYT12            -AVGSKPLPSRKASLRGADTCESINELGSLELMSRDLGLASYGPLKKSVSADSLNSVS--------SIGNNFGQDFTVGQVEVSMEYDGK------AAALHVTLLQGKDLLE-KEDAR
TguttataSYT12           -------------------TPWRASGAGQLELMGRELGLAHYGPLRKSISADSLNSIS--------SIGNNFGQDFTVGQVEVSMEYDGR------AAALHVTLLQGKDLLE-KEDAR
OanatinusSyt12          YQGSERTPPSLKAGLQIDDSLENVTELGTLELMNRDPDLAPFGPLRRSLSADSLSSISRRLAQLHLSITNNFGQDFALGQVEVTLEYEVA------VHALHVTVLAGKDLTE-KEDAS
MdomesticaSyt12         RKSGERGSPSRKSSLRMDDAFESISELGPLELMNRDLDLAPYGPLKKSQSADSLNSIS--------SVNNNLGQDFLLGQVEVNITYEPR------SHLLRVDVVQGKDLME-KEDPA
MmusculusSyt12          QRTTTRGPPSRKGSLSIEDTFESISELGPLELMGRELDLAPYGTLRKSQSADSLNSIS--------SVSNTFGQDFTLGQVEVSMDYDGA------SHTLHVAVLQGKDLLE-REEAT
HsapiensSYT12var1       QRASTRGPPSRKGSLSIEDTFESISELGPLELMGRELDLAPYGTLRKSQSADSLNSIS--------SVSNTFGQDFTLGQVEVSMEYDTA------SHTLNVAVMQGKDLLE-REEAS
HsapiensSYT12var2       QRASTRGPPSRKGSLSIEDTFESISELGPLELMGRELDLAPYGTLRKSQSADSLNSIS--------SVSNTFGQDFTLGQVEVSMEYDTA------SHTLNVAVMQGKDLLE-REEAS

                                                                               300
Trubripessyt12          FPGCFIRVSLGPEEINIGVTR-VQTNAFTVIFDEHYTIPMDLSALEEYTLRFAAFGIDADERNISAGIADLKLSDLDLTVRPFNAWLYLQDVNKAVDAVGEILLSLSYLPTAERLTVV
Tnigroviridissyt12      FPGCFIRVSLGPEEINVGVTR-VQTNAFTVMFDEHYTVPVDLSTLEEYSLRFAAFGIDADERNISAGIADLKLSDLDLTIRPFNAWLYLQDVNKAVDAVGEILLSLSYLPTAERLTVV
Gaculeatussyt12         FPGCFIRVSLGPDEISVGVTR-IQTNAYTVLFDERFSIPMDASLLEEYSLRCAAFGIDADERNISAGVADLKLSDLDLTVRPFNAWLYLQDVNKAVDAVGEILLSLSYLPTAERLTVV
Olatipessyt12           FPGCFIRVSLGPEELSVGVTR-VQTNAFTVIFDERFSVPMDLSSLEEYSLRFAAFGIDSDERNISAGVAELKLLDLDLTIRPFNAWLYLQDVNKAVDAVGEILLSLSYLPTAERLTVV
Dreriosyt12             FPGCFITVTLVPQQINLGATQ-VQRNAFTVVFDERFSVPLESVNLEENSLRFSTFGVDSDERNITAGVAELKLSDLDLPYRPFNAWLYLQDINKAVDAVGEILLSLSYLPTAERLTVV
Xtropicalissyt12        FESCFVHISLLPDEQIVGISR-IQGNAYSVMFDETFSIPLDPLALEENSLKFSIFGIDGDDRKVSTGVAELKLSDLDLALRPFNTWIYLQDVNKATDTVGEILLSLSYLPTAERLTVV
Acarolinensussyt12      FESCFVRISLLPDEQIVGISR-IQRSAYSVFFDERFSIPLDPSALEENSLRFSVFGIDEDERNISTGVAELKLSDLDLSIRPFNAWLYLQDMNKARDSVGEILLSLSYLPTAERLTVV
GgallusSYT12            FESCFMRISLLPAEQIVGISR-IQRSAYAVAFDERFSIPLDPAALEEDSLRFSVFGIDEDERNVSTGVAELKLSDLDLTVRPFNAWLYLQDMNKTIDTVGEILLSLSYLPTAERLTVV
TguttataSYT12           FESCFMRISLLPAEQIVGISR-IQRSSYSVAFDERFSVPLDPVALEENSLRFSVFGIDEDERSVSTGVAELKLSDLDLATRPFNAWLYLQDTNKAVDTVGEILLSLSYLPTAERLTVV
OanatinusSyt12          FQSCFVRVSLLPDEQIVGISRGIPCRRKAFCFDERFSIPLDPSASEENSLRFSVFGIDEDERNVSTGVVELKLSVLDLTVQPFTGWLYLQDLNKAADAVGEILLSLSYLPTAERLTVV
MdomesticaSyt12         FDSCFLRVSLLPDEQIVGISR-IQRNAYSIFFDEKFSIYVDPIAMEDSSLRFSVFGIDDDDRNISTGAVDLKLSVLELAQQPFSGWLYLQDLNKASDAVGEILLSLSYLPTAERLTVV
MmusculusSyt12          FESCFMRVSLLPDEQIVGISR-IQRNAYSIFFDEKFSVPLDPTALEEKSLRFSVFGIDEDERNVSTGVVELKLSVLDLPLQPFSGWLYLQDQNKAADAVGEILLSLSYLPTAERLTVV
HsapiensSYT12var1       FESCFMRVSLLPDEQIVGISR-IQRNAYSIFFDEKFSIPLDPTALEEKSLRFSVFGIDEDERNVSTGVVELKLSVLDLPLQPFSGWLYLQDQNKAADAVGEILLSLSYLPTAERLTVV
HsapiensSYT12var2       FESCFMRVSLLPDEQIVGISR-IQRNAYSIFFDEKFSIPLDPTALEEKSLRFSVFGIDEDERNVSTGVVELKLSVLDLPLQPFSGWLYLQDQNKAADAVGEILLSLSYLPTAERLTVV

                                                                     400
Trubripessyt12          VAKCKNLVWTDSKTTADPFVKVYLLQDGKKISKKKTSTKRDDTNPIFNEAMIFSVPSNVLQELSLRVTVAETTEDGRGENLGHVIIGPEASGMGITHWNQMLATLRKPVSMWHPLRRI
Tnigroviridissyt12      VAKCKNLVWTDSKTTADPFVKVYLLQDGRKISKKKTSTKRDDTNPIFNEAMIFSVPSNVLQELSLRVTVAETTDDGRGENLGHVIIGPEASGMGITHWNQMLATLRKPVSMWHPLRRI
Gaculeatussyt12         VAKCKNLVWTNSKNTADPFVKVYLLQDGRKISKKKTSTKRDDTNPIFNEAMIFSVPSLVLQELSLRVTVAEATDDGRGENLGHVIIGPEASGIGITHWNQMLATLRKPVSMWHPIRRI
Olatipessyt12           VAKCKNLVWTNEKNTADPFVKVYLLQDGRKISKKKTSTKRDDTNPIFNEAMIFSVPSLVLQELSLRVTVAEATDDGRGENLGHVIIGPEASGMGITHWNQMLATLRKPVSMWHPLRRI
Dreriosyt12             IAKAKNLVWTNGKTTADPFVKVYLLQDGRKISKKKTSIKRDDTNPIFNEAMIFSVPAIVLQDLSLRVTVAESTEDGRGENVGHVIIGPEASGMGITHWNQMLATLRKPVSMWHPLRRT
Xtropicalissyt12        VVKVRNLLWTEGKTTADPFVKVYLLQDGRKISKKKTAVKRDDRNPVFNEAMIFSVPAIVLQELSLRITVAECCEDGRAENIGHVIIGPSTSGMGMTHWNQMLATLRKPVSMWHQIRQN
Acarolinensussyt12      VVKAKNLVWTNGKMSADPFVKVYLLQDGRKISKKKTAVKRDEANPVFNEAMIFSVPAIVLQELSLRVTVAECCDDGRAENIGHVVIGPAAGGMGITHWNQMLATLRKPVSMWHPLRRS
GgallusSYT12            VVKAKNLVWSNGKGTADPFVKVYLLQDGRKISKKKTAVKRDDTNPVFNEAMIFSVPAIVLQDLSLRVTVAESGEDGRADNTGHVLIGPAASGMGITHWNQMLATLRKPVSMWHPLRRN
TguttataSYT12           VVKAKNLVWSNGKVTADPFVKVYLLQDGRKISKKKTAVKRGDTNPVFNEAMIFSVPAIVLQELSLRVTVAESGEDGRGDNTGHVLIGPAASGMGTTHWNQMLATLRKPVSMWHPLRRN
OanatinusSyt12          VVKAKNLVWTGGKTSADPFVKVYLLQDGRKMSKKKTAVKRDDPNPVFNEAMIFSVPAIVLQDLSLRVTVAERCGDGRADSIGHVVIGPATSGMGITHWNQMLATLRRPVSMWHAVRRS
MdomesticaSyt12         VVKAKNLVWTNDKATADPFVKVYLLQDGRKISKKKTAVKKDDPHPVFNEAMIFSVPAIVLQX--------------------------------------------------------
MmusculusSyt12          VVKAKNLIWTNEKSTADPFVKVYLLQDGRKMSKKKTAVKRDDPNPVFNEAMIFSVPAIVLQDLSLRVTVAESSSDGRGDNVGHVIIGPGVSGMGTTHWNQMLATLRRPVSMWHPVRRN
HsapiensSYT12var1       VVKAKNLIWTNDKTTADPFVKVYLLQDGRKMSKKKTAVKRDDPNPVFNEAMIFSVPAIVLQDLSLRVTVAESSSDGRGDNVGHVIIGPSASGMGTTHWNQMLATLRRPVSMWHAVRRN
HsapiensSYT12var2       VVKAKNLIWTNDKTTADPFVKVYLLQDGRKMSKKKTAVKRDDPNPVFNEAMIFSVPAIVLQDLSLRVTVAESSSDGRGDNVGHVIIGPSASGMGTTHWNQMLATLRRPVSMWHAVRRN
```
