# Supplementary material for: A manual collection of Syt, Esyt, Rph3a, Rph3al, Doc2, and Dblc2 genes from 46 metazoan genomes - an open access resource for neuroscience and evolutionary biology
Source: BMC Genomics. 2010 Jan 15;11:37. doi: 10.1186/1471-2164-11-37 (PMC2823689; doi:10.1186/1471-2164-11-37)
Supplement: Additional file 26 — Alignment of the invertebrate Syt13 sequences. Amino acid position is marked every hundred amino acids approximately, at the top of each page of the alignment. Splice variants which specify an alternative N-terminus lacking a TM domain, are indicated on top, with a dotted line indicating where they join the common C-terminal portion. Intron position and phase is indicated with a coloured bar between amino acids. Black bars indicate phase 0 introns. Red bars indicate phase +1 introns. Blue bars indicate phase +2 introns. The widely conserved motif of unknown function, upstream of the C2A domain, is indicated. X residues indicate where a portion of sequence is missing. [file 1471-2164-11-37-S26.PDF]

```
                                                                                      100
                           |         |         |         |         |         |         |         |         |         |         |
NvitripennisSyt13var2      ------XVVLEALKCVKVPDKLSEVLGDVLELACGQSQSQHQQQDAATASAVTTSSSSSTTA-SSACSRISAITGVAIIRGRWTQRQARVA-SSASSSTTQDSQDSSE----------------
AmelliferaSyt13var2        -------XVLDTF--VKVPGKLSEVLGDVLELACGGGGGSGGSSGGGSGRGNEAGGKSGNSSIGNAASRIGVVAGVATIRDKWAQRGHRSPLSSVANTDSADSTTSTSSE---------------
DmelanogasterSyt13var2     MSGLGPAACWLAHKRIESWSRMAKERVGAVRRVTLDRNSADEGTPSGSG----ASSTAGGNGVSVVHILPI-----GEGSTTPPP-QPMTPPPSASSVTSTTSGIGSVSAGSVSDSCDLPTDSE
DsimulansSyt13var2         MSGLGPAACWLAHKRIESWSRMAKERVGAVRRVTLDRNSADEGTPSGSG----ASSTAGGNGVSVVHLLPM-----GEGSTTPPP-QPMTPPPSASSVTSTTSGIGSVSAGSVSDSCDLPTDSE
DsecheliaSyt13var2         MSGLGPAACWLAHKRIESWSRMAKERVGAVRRVTLDRNSADEGTPSGSG----ASSTAGGNGVSVVHILPM-----GEGSTTPPP-QPMTPPPSASSVTSTTSGIGSVSAGSVSDSCDLPTDSE
DerectaSyt13var2           MSGLGPAACWLAHKRIESWSRMAKERVGAVRRVTLDRNSADEGTASGSGSGSGASAAAGGNGASAVHILPM-----GEGSTTPPP-QPMTPPPSASSVTSTTSGIGSVSAGSVSDSCDLPTDSE
DyakubaSyt13var2           MSGLGPAACWLAHKRIESWSRMAKERVGAVRRVTLDRNSADEGTPSGSG----ASTTAGGNGVSVVHILPM-----GEGSTTPPP-QPMTPPPSASSVTSTTSGIGSVSAGSVSDSCDLPTDSE
DananassaeSyt13var2        MSGLGPAACWLAHKRIESWSRMAKERVGAVRRVTLDRNSADESSASGSGAASTAGATGGGNGVA--HTLPMIGDGGSGGGTTPPP-QPMTPPPSASSVTSTTSGIGSVSAGSVSDSCDLPTDSE
DpseudoobscuraSyt13var2    MSGLGPAACWLAHKRIESWSRMAKERVGAVRRVTIDRNSGDESSASGSSTAGSSTIQTLPTGETAPAAAAAAAAAPSSGPSTTPPPQPMTPPPSASSVTSTTSGIGSVSAGSGSDSCDLTTDSE
DpersimilisSyt13var2       MSGLGPAACWLAHKRIESWSRMAKERVGAVRRVTIDRNSGDESSASGSSTAGSSTIQTLPTGETAPAAAAAAAA-PSSGPSTTPPPQPMTPPPSASSVTSTTSGIGSVSAGSGSDSCDLTTDSE
DwillistoniSyt13var2       MSGLGPAACWLAHKRIETWSRMAKERVGAVRRVTIDRNSADSADGSANG------LQTLLTGELSTCS--------ATGSSTTPPPQPMTPPPSASSVTSTTSGIGSVSAGSVSDSCDLPTDSE
DvirilisSyt13var2          MSGLGPAACWLAHKRIESWSRIAKERVGAVRRVTLDRNSADESSGSG-----------GSNGCT--QVLPG---SEGASSTTPPP-QPMTPPPSASSVTSTTSGIGSVSAGSVSDSCDLPTDSE
DmojavensisSyt13var2       MSGLGPAACWLAHKRIESWSRMAKERVGAVRRVTLDRNSADETTGSGSG------SGSGCGGCT--QMLPG---SEGAASTTPPPLQPMTPPPSASSVTSTTSGIGSVSAGSGSDSCDLPTDSE
DgrimshawiSyt13var2        MSGLGPAACWLAHKRIESWSRMAKERVGAVRRVTLDRNSADESSGSGSGGSGSGSGGS-GSGSSNGCTQILPGSDGAASSTTPPPPQQMTPPPSASSVTSTTSGIGSVSAGSVSDSCDLPTDSE
```

```
                                                                                      100
                           |         |         |         |         |         |         |         |         |         |         |
IscapularisSyt13           ----------------------------------------------------------------------------------------------------------------------------
DpulexSyt13                --MPTAAVLGAAVGVGGALFLTLTLLIYRYHAAQKKEAADQHYPYHHGGKGAGYNSLSNSWSTRDSWAPPASPTPPPPYKTSNCSLIDEAHGAHNGKRRPLAKLYLPLTTHHQNQYTRSPSPS
TcastaneumSyt13            --MVASAVLGAAAGTGMALFVAMTIVVYRYYSHRRK---------------------LKEWNSLDRMPFP-----TTPTRIINKP---------------IYAVAQTVSLHFHHAEFNV----
NvitripennisSyt13var1      --MVSSAVLGAAAGTGLALVIAVTIVVYRYYSTGRK---------------------VKSWSSLDRWPEPPKCPSLNQSKVVRQQLPSHHQDNNDPEACRVLNQSKTCYYSVQKTELSV----
AmelliferaSyt13var1        --MVSSAIFGAAAGTGLALVVAMTIVVYRYYAVKRK---------------------GKYWSSLDRWPDPP---GTKKGEGRQEQCSEHCHDSSSSHVLNCWRKQQKNYYAVQSREFNV----
AgambiaeSyt13              MVFVSSAVLGAAAGTGLALLVAVTIVMYRYYVVRRK---------------------GKEWAELDRL---------EEKKAARK---------------------------INLQDCNV----
DmelanogasterSyt13var1     MVMVSSAVLGAAAGTGLALILAVTIVMYRYYLLRKR---------------------GKEWDELDRW---------EETRIMRK---------------------------LQMKEVAI----
DsimulansSyt13var1         MVMVSSAVLGAAAGTGLALILAVTIVMYRYYLLRKR---------------------GKEWDELDRW---------EETRIMRK---------------------------LQMKEVAI----
DsecheliaSyt13var1         MVMVSSAVLGAAAGTGLALILAVTIVMYRYYLLRKR---------------------GKEWDELDRW---------EETRIMRK---------------------------LQMKEVAI----
DerectaSyt13var1           MVMVSSAVLGAAAGTGLALILAVTIVMYRYYLLRKR---------------------GKEWDELDRW---------EETRIMRK---------------------------LQMKEVAI----
DyakubaSyt13var1           MVMVSSAVLGAAAGTGLALILAVTIVMYRYYLLRKR---------------------GKEWDELDRW---------EETRIMRK---------------------------LQMKEVAI----
DananassaeSyt13var1        MVMVSSAVLGAAAGTGLALLLAVTIVMYRYYLLRRR---------------------GKEWDELDRW---------EETRIMRK---------------------------LQMKEVAI----
DpseudoobscuraSyt13var1    MVMVSSAVLGAAAGTGLALLLAVTIVMYRYYLLRRR---------------------GKEWEELDRW---------EETRIMRK---------------------------LQMKEVNI----
DpersimilisSyt13var1       MVMVSSAVLGAAAGTGLALLLAVTIVMYRYYLLRRR---------------------GKEWEELDRW---------EETRIMRK---------------------------LQMKEVNI----
DwillistoniSyt13var1       MVLVSSAVLGAAAGTGLALLVAVTIVMYRYYLLRKR---------------------GKEWEELDRW---------EATRIMRK---------------------------LQLKEVNI----
DvirilisSyt13var1          MVMVSSAVLGAATGTGLALLLAVTIVMYRYYLLRRR---------------------GKEWDELDRW---------EETRIMRK---------------------------LKLKEVNI----
DmojavensisSyt13var1       MVMVSSAVLGAAAGTGLALLLAVTIVMYRYYLLRRR---------------------GKEWDELDRW---------EETRIMRK---------------------------LQMKEVNI----
DgrimshawiSyt13var1        MVMVSSAVLGAAAGTGLALILAVTIVMYRYYLLRKR---------------------GKEWDELDRW---------EETRIMRK---------------------------LKMKEVNI----
```

```
                                                                            200
                           |         |         |         |         |         |         |         |         |         |         |
IscapularisSyt13           ----------------------------------------------------------------------------------------------------------------------------
DpulexSyt13                CRHRLASNPTPLFGGGGGGGTTTLASSTSGHHNSMNVSSNNLNLTPYPSPMVSPTPSFSSLLNSLASVTSSTCNSPVPTTTTTPASPLVRRNRSPML--------------------------
TcastaneumSyt13            AKEQHIIQPPQNT--SAQALTAELMKMPLELCSAPQGSH----SLQHQSKSYPGGQ---PLLCRTPSVSSQS------SLDSSASR-QGHRGSSPQIRTFAPDGKTPVHQEAIHTSSYPLRNS
NvitripennisSyt13var1      AKEQHAVQSSSAA---SESSAGQSRSSHMGLV----MGR----TLTSLSRRPSSGSQE-----QLPATPPSG------EQQHHHHT-SASATPSPQIRAYGPDGRHHNHELLHAASSYPPSRG
AmelliferaSyt13var1        AKEQHAVQPCSSVVVESGSLPHQSRSYPGGTTGSGSMGR----LFTRNSSVSSQSSLEGPSGPSGPSGPSGS------SGSSGPSS-SHRSTTTTQIRSFGPDGRYHRPDPLHAASSYPPSRS
AgambiaeSyt13              TSGSVPIQPASS---------VVHHSISSET-----LGTGAVEVTPSPLQPTLSATG------STKKSYGTS------DTRTGPGCRPAHAAASTASRNNSMESVHSRSSK------------
DmelanogasterSyt13var1     AKECQPIQPASQLHGMHGGMGLNHHHMGTVSATGTDILRGPAEVFHSPLHLHHQSRSFPPRLQRTPSISSQS------SVDSAPSRHSGHRGSSPQIRTFGPDGRSSLPSE----PGFP-HHL
DsimulansSyt13var1         AKECQPIQPASQLHGMHGGLGLNHHHMGTVSATGTDILRGPAEVFHSPLHLHHQSRSFPPRLQRTPSISSQS------SVDSAPSRHSGHRGSSPQIRTFGPDGRSSLPSE----PGFP-HHL
DsecheliaSyt13var1         AKECQPIQPASQLHGMHAGMGLNHHHMGTVSATGTDILRGPAEVFHSPLHLHHQSRSFPPRLQRTPSISSQS------SVDSAPSRHSGHRGSSPQIRTFGPDGRSSLPSE----PGFP-HHL
DerectaSyt13var1           AKECQPIQPASQLHGMHGGMGLNHHHMGTVSATGTDILRGPAEVFHSPLHLHHQSRSFPPRLQRTPSISSQS------SVDSAPSRNSGHRGSSPQIRTFGPDGRSSLPSE----PGFP-HHL
DyakubaSyt13var1           AKECQPIQPASQLHGMHGGMGLNHHHMGTVSATGTDILRGPAEVFHSPLHLHHQSRSFPPRLQRTPSISSQS------SVDSAPSRNSGHRGSSPQIRTFGPDGRSSLPSE----PGFP-HHL
DananassaeSyt13var1        AKECQPIQPASQMHGM-GGMGLNHHHMGTVSATGTEILRGPAEVFHSPLHLHHQSRSFPPRLQRTPSISSQS------SVDSAPSRHSGHRGSSPQIRTFGPDGRSSLPSE----PAFP-HHL
DpseudoobscuraSyt13var1    AKESQPIQPPTQ---MHGLAMGVNHHMGPGTVAGSDILRGPVEVFHSPLHLHHQSRSFPPRLQRTPSISSQS------SVDSAPSRHSGHRGSSPQIRTFGPDGRSSLPSE----PAFP-HHL
DpersimilisSyt13var1       AKESQPIQPPTQ---MHGLAMGVNHHMGPGTVAGSDILRGPVEVFHSPLHLHHQSRSFPPRLQRTPSISSQS------SVDSAPSRHSGHRGSSPQIRTFGPDGRSSLPSE----PAFP-HHL
DwillistoniSyt13var1       AKESQPIQPASH--GLHGLVGGSNQAMATGT----EILRGPAEVFHSPLHLHHQSRSFPPRLQRTPSISSQS------SVDSAPSRHSGHRGSSPQIRTFGPDGRSSLPSE----PAFP-HHL
DvirilisSyt13var1          SKESHPIQPATQ---LHGIPCVPNHPIPA------DILRGPAEVFHSPLHLHHQSRSFPPRLQRTPSISSQS------SVDSAPSRQSGHRGSSPQIRTFGPDGRSSLPSE----AAFP-HHL
DmojavensisSyt13var1       SKESHTIQPATQ---LHGIPYVPTNPIPS------DILRGPAEIFHSPLHLHHQSRSFPPRLQRTPSISSQS------SMDSAPSRQSGHRGSSPQIRTFGPDGRSSLPSD----SAFP-HHL
DgrimshawiSyt13var1        SRETHPVQPATQ---LHGIPCVPNHPLSA------DAIRGPPELFHSPLHLHHQSRSFPPRLQRTPSISSQS------SVDSAPSRQSGHRGSSPQIRTFGPDGRSSLPSE----SAFP-HHL
```

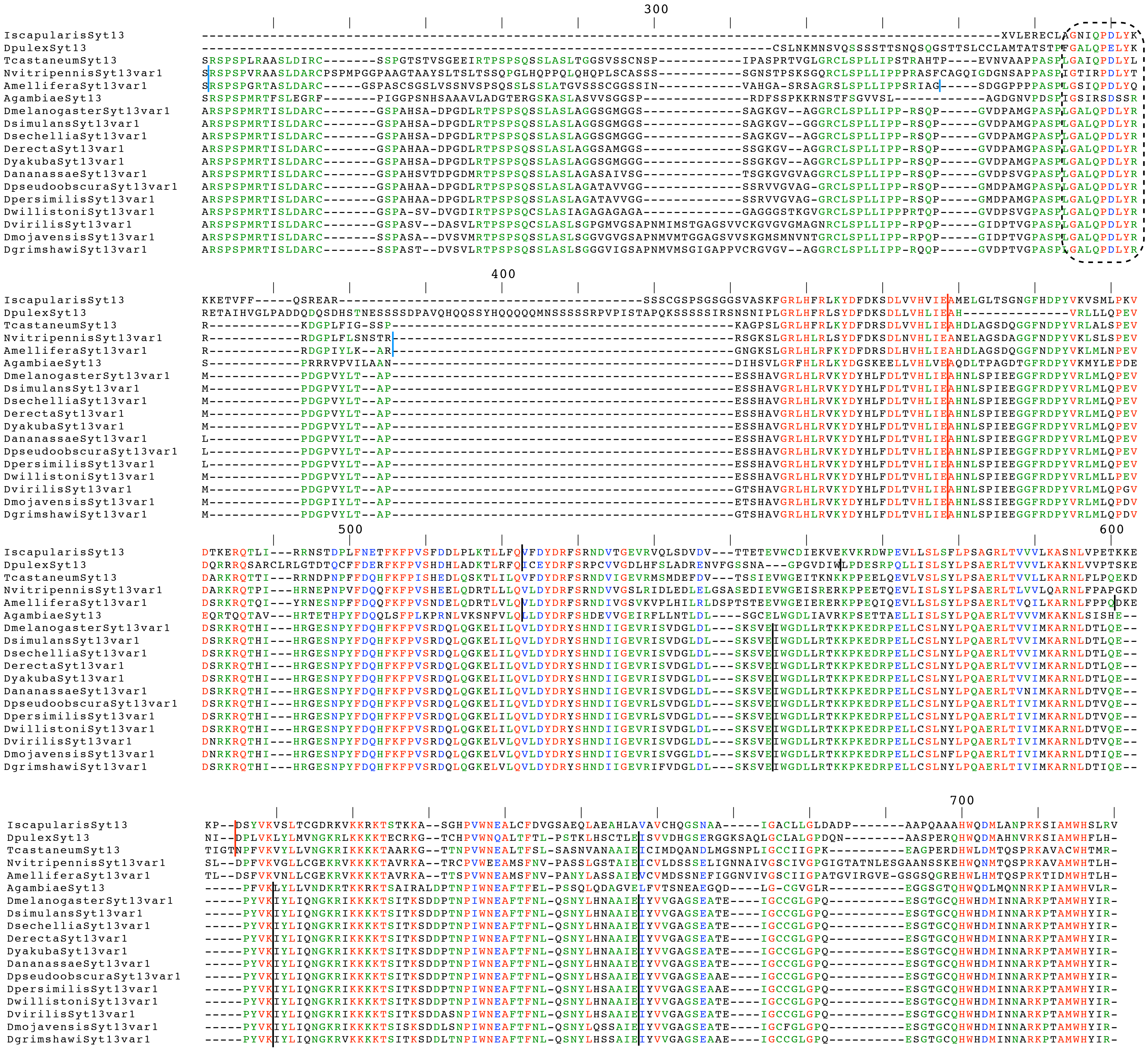
300
400
500
600
700
IscapularisSyt13
DpulexSyt13
TcastaneumSyt13
NvitripennisSyt13var1
AmelliferaSyt13var1
AgambiaeSyt13
DmelanogasterSyt13var1
DsimulansSyt13var1
DsechelliaSyt13var1
DerectaSyt13var1
DyakubaSyt13var1
DananassaeSyt13var1
DpseudoobscuraSyt13var1
DpersimilisSyt13var1
DwillistoniSyt13var1
DvirilisSyt13var1
DmojavensisSyt13var1
DgrimshawiSyt13var1
