# Supplementary material for: A manual collection of Syt, Esyt, Rph3a, Rph3al, Doc2, and Dblc2 genes from 46 metazoan genomes - an open access resource for neuroscience and evolutionary biology
Source: BMC Genomics. 2010 Jan 15;11:37. doi: 10.1186/1471-2164-11-37 (PMC2823689; doi:10.1186/1471-2164-11-37)
Supplement: Additional file 27 — Alignment of the vertebrate Syt13 sequences. Amino acid position is marked every hundred amino acids approximately, at the top of each page of the alignment. The H. sapiens splice variant is included and highlighted with a black dot where it differs. Intron position and phase is indicated with a coloured bar between amino acids. Black bars indicate phase 0 introns. Red bars indicate phase +1 introns. The widely conserved motif of unknown function, just upstream of the C2A domain, is indicated. X residues indicate where a portion of sequence is missing. [file 1471-2164-11-37-S27.PDF]

```
                                                                                                          100
                            |         |         |         |         |         |         |         |         |         |
Dreriosyt13             -MLVSATALLGATLGTVSGV-LTLCGLSLLCKSC--KKGKLESGDEADPEKAKPSILHTLTQFSVHKCTEPIQPQASLKFPQIYRPKPSVTSQEVINYKEHGASN-----
Xtropicalissyt13        ---MLPIIAVGVTTGTVLGIFLALCSSVFLYSCLKRKKYTWAG--GDGTPVTMANLFQPGQPMSIYKCTELVHPQAKLRFPYIYRSKE--PCPTIGRGGDKEETSKEN--
Acarolinensissyt13      MVLSIPVIALGATLGTATSI-LALCGLTCLCKCKRAGKADSDKEKGLCAENAKPSVLQTVQQFNLEKTAEPVQPRTILKFSHIYGPKPVVTSSEIANYGDYTSKTTKEP-
GgallusSYT13            MVLSAPIIALGATLGTATSI-LALCGLTCFCKCKQPGKGLSEKDQDEDTENTKPSVLQPAQQFNVKKTAEPVQPRALLKFPNIYGPKPVVTSPEIVNYTQYSLKTTEEP-
TguttataSYT13           MVLSAPVIALGATLGTATSI-LALCGLTCFCKCKQPGKGLSEKEQEDETENTKPSVLQPVQQFNIKKTAEPVQPRALLKFPNIYGPKPEVTSPEVVNYTQYSLKTTEEP-
OanatinusSyt13          MVLSVPVIALGATLGTATSI-LALCGFTCLCRHMHPKKGVLAKEQDDAEKRPGPACSGPSRQFNVKKSTEPVQPRALLKFPDIYGPKPAVTAPEVINYTDYSLRAAEEA-
MdomesticaSyt13         -------------------------------------------------------------XFNIKKSTEPVQPRALLKFPDIYGPKPAVTAPEVINYADYSLKTTEEEE
MmusculusSyt13          MVLSVPVIALGATLGTATSI-LALCGVTCLCRHMHPKKGLLPRDREPDPEKARPGVLQAAQQFNIKKSTEPVQPRPLLKFPDIYGPRPAVTAPEVINYADYTLETTEES-
HsapiensSYT13var1       MVLSVPVIALGATLGTATSI-LALCGVTCLCRHMHPKKGLLPRDQDPDLEKAKPSLLGSAQQFNVKKSTEPVQPRALLKFPDIYGPRPAVTAPEVINYADYSLRSTEEP-
HsapiensSYT13var2       --------------------------------------------------------------------------------------------------------------

                                                                                                200
                            |         |         |         |         |         |         |         |         |         |
Dreriosyt13             ---DTSAAELDTCNQATEREEVFSLPRQASADEIPCSSEQTGAMTTSSSILYPKLHFSISLHKESGELHINIVEAE----NISVEAGCEGYISGCVSVSEEQKHAHTAVH
Xtropicalissyt13        --KDEKSGETEPQGKVETISEKINSEKNE--ESL-APENEYVEKFSQCLNHIPKLRYSLGYDHQKRELCVSFLEAVGCPLTKEEDSGSHSYIVGTLTSNGGQTEAQTSLM
Acarolinensissyt13      S-KGKSESLDEKRVNIQVNEELFAISQNGGMKDV-CVTERLSPEKAAGCNQMPELHYSLGYNRQKNALCVTLLEILYETTLGDQNTGCDCYILGTLVSKSGTTEAQTVLK
GgallusSYT13            A-TVKHAVLDENRMKIQVNEELFVLPQNGVVKDV-CVTEHLKPERAASCNQVPELRYSLTYDQQKAELCVALLEAMHGKMSSDQDTGCHCYILGTLVSKSGMTEAQTELK
TguttataSYT13           P-TGKHTALDENRMKIQVNEELFVLPQNGVVKDV-CVTEHLKPERAGSGRQAPELHYSLLYEPQQAQLCVSLLQAMHDGMNGDQDTGCHCYILGTLESKSGIAEAQTELK
OanatinusSyt13          T-PVSAPALNDSRLKRQVTEELFILPQNGVVEDV-CVIETWNPEKAASWNQAPKLQYTLNYDGQKAKLSVSLLEAV----TGDQDAGCDCYIQGCVTSKTGTTEAQTVLK
MdomesticaSyt13         SVPASTQALNDSRLKRQVTEELFILPQNGVVEDV-CVIETWNPEKAASWNQAPKLHYSLGYDQQKAQLSVTLLEAV----TGDHDVGCDCYIQGSMASRSGTVEAQTALK
MmusculusSyt13          AAPASPQAQSDSRLKRQVTEELSIRPQNGVVEDV-CVMETWNPEKAASWNQAPKLHFRLDYDQKKAELFVTSLEAV----TSDHEGGCDCYIQGSVAVKTGSVEAQTALK
HsapiensSYT13var1       TAPASPQPPNDSRLKRQVTEELFILPQNGVVEDV-CVMETWNPEKAASWNQAPKLHYCLDYDCQKAELFVTRLEAV----TSNHDGGCDCYVQGSVANRTGSVEAQTALK
HsapiensSYT13var2     ● -------------------------------------METWNPEKAASWNQAPKLHYCLDYDCQKAELFVTRLEAV----TSNHDGGCDCYVQGSVANRTGSVEAQTALK

                                                                                      300
                            |         |         |         |         |         |         |         |         |         |
Dreriosyt13             KLAVHVQWGEELVFALPMESTEDTDSLDGEVALSLHCCDRFSHNSTLGMMRFKLADVSMMLDADCWVDLQPPKQEVTSSTGELLLSLSYLPAANRLGVVVMKARGLQSDK
Xtropicalissyt13        NRTPHTVWDEALLFPLSEE-----ERVEAELTLTLRHCDRYSRHQVAGEITLSLANLGVPFGAARWVDLRPPEKELE-GSGEVLLSLSYLPAASRLIVVVIKARNIHCDQ
Acarolinensissyt13      KKQLHVAWEDVLLFPVKEE-----ELPEGTLTLTLRNCDKFSRHIIVGEVKPKLASVGEPYGPVQSEKMKAFDKEPDAGYGEVLLSISYLPAANRLLVVLIKAKNLHSKQ
GgallusSYT13            KKVLHTLWEEALRFPLTEE-----EMQEGTLTLTLRNCDKFSRHSIVGELKLSLANMED-FGMAQWERLKTPEKEPSTGHGEVLLSISYLPAANRLLVVIIKAKNLHSKQ
TguttataSYT13           KKVLHTLWEEVLQFPLTEE-----EMPGGTLTLTLRNCDKFSRHSIVGELKLNLAEMEESFGKAQWERLKSPEKEPSTGHGEVLLSISYLPAANRLLVVIIKAKNLHSKQ
OanatinusSyt13          KRLPRTAWEEALAFPLAEE-----ELAAASLTLTLRNCDRFSRHSVAGEMKLSLAGPGVSWGVAQCGELKTSEKELAAGTGEVLLSISYLPAANRLLVVLIKAKNLHSNQ
MdomesticaSyt13         KRLPHTSWEEALVFPLPEE-----ERPAATLTLTLRTCDRFSRHSVVGELQLPLDGVSLPLGMAQWGELKTSAKDLATGSGEVLLSISYLPAANRLLVVLIKAKNLHSNQ
MmusculusSyt13          KRQLHTTWEEGLALPLGEE-----ELPTATLTLTLRTCDRFSRHSVIGELRLGLDGASVPLGAAQWGELKTTAKEPSAGAGEVLLSISYLPAANRLLVVLIKAKNLHSNQ
HsapiensSYT13var1       KRQLHTTWEEGLVLPLAEE-----ELPTATLTLTLRTCDRFSRHSVAGELRLGLDGTSVPLGAAQWGELKTSAKEPSAGAGEVLLSISYLPAANRLLVVLIKAKNLHSNQ
HsapiensSYT13var2       KRQLHTTWEEGLVLPLAEE-----ELPTATLTLTLRTCDRFSRHSVAGELRLGLDGTSVPLGAAQWGELKTSAKEPSAGAGEVLLSISYLPAANRLLVVLIKAKNLHSNQ

                                                                    400
                            |         |         |         |         |         |         |         |         |
Dreriosyt13             LKDNI--DLSVKLTLKHQNAKLKKKQTRRVKHKMNPVWNEMMMLELPSELLAKSSVDLEVLN-LASPGTLLPLGRCMLGLQTSGTGLQHWKQMLDNPRKQIAMWHPLYT
Xtropicalissyt13        YNNLLGKDLSIKVILKHQSQKLKKKQTKRTKHMINPVWNEMVMFEVPQELLGDVYVELQMVCVKPDRSGNHILGTCNLGAEWTGTGKNHWLEMMNNPRRQIAFWHRLNT
Acarolinensissyt13      LKELLGKDISVKVTLKHQALKLKKKQTKRAKHKINPVWNEMIMFEVPHDLLCASSVELEMLS-QDGDGQNHLLGKCSLGLHATGTERSHWEEMLRNPRKQIAMWHQLHM
GgallusSYT13            LKDLLGNDVSVKVTLRHQSLKLKKKQTKHAKHKINPVWNEMIMFEVPHELLRASSVELEMLS-QDGAGQSQVLGKCSLGLHVTGTERNHWEEMLRNPRRQIAMWHQLHM
TguttataSYT13           LKDLLGSDVSVKVTLRHQSLKLKKKQTKRAKHKINPVWNEMIMFEVPHELLRASSVELEMLS-QDGAGQSHVLGKCSLGLHVTGTERNHWEEMLRNPRKQIAMWHQLHM
OanatinusSyt13          SKDLLGKDVSVKVTLKHQALKLKKKQTKRAKHKINPVWNEMIMFEVPQDLLRASSVELEMLG-QDEAGQTLVLGRCSLGLHASGSMLSHWEEMLKNPRRQIAMWHPLHP
MdomesticaSyt13         SKDVLGKDVSVKVTLKHQALKLKKKQTKRAKHKLNPVWNEMIMFEVPDDLLRASSVELEMLG-QDGAGQSHVLGRCSLGLHSSGTQRSHWEEMLKNPRRQIAMWHQLQM
MmusculusSyt13          SKELLGKDVSVKVTLKHQAQKLKKKQTKRAKHKINPVWNEMIMFELPDDLLRASSVELEVLG-QGEEGPSCELGHCSLGLHASGSERSHWEEMLKNPRRQIAMWHQLHL
HsapiensSYT13var1       SKELLGKDVSVKVTLKHQARKLKKKQTKRAKHKINPVWNEMIMFELPDDLLQASSVELEVLG-QDDSGQSCALGHCSLGLHTSGSERSHWEEMLKNPRRQIAMWHQLHL
HsapiensSYT13var2       SKELLGKDVSVKVTLKHQARKLKKKQTKRAKHKINPVWNEMIMFELPDDLLQASSVELEVLG-QDDSGQSCALGHCSLGLHTSGSERSHWEEMLKNPRRQIAMWHQLHL
```
