# Supplementary material for: A manual collection of Syt, Esyt, Rph3a, Rph3al, Doc2, and Dblc2 genes from 46 metazoan genomes - an open access resource for neuroscience and evolutionary biology
Source: BMC Genomics. 2010 Jan 15;11:37. doi: 10.1186/1471-2164-11-37 (PMC2823689; doi:10.1186/1471-2164-11-37)
Supplement: Additional file 28 — Alignment of the invertebrate Syt15 sequences. Amino acid position is marked every hundred amino acids approximately, at the top of each page of the alignment. Intron position and phase is indicated with a coloured bar between amino acids. Black bars indicate phase 0 introns. Red bars indicate phase +1 introns. Blue bars indicate phase +2 introns. Because of their differing positions, TM domains are highlighted in blue. The widely conserved motif of unknown function, just upstream of the C2A domain, is indicated. X residues indicate where a portion of sequence is missing. [file 1471-2164-11-37-S28.PDF]

```
                                                                                                                    100
CapitellaSyt15        ----------------------------------------------------------------------------------------------------------------------
HrobustaSyt15a        ----------------------------------------------------------------------------------------------------------MMSFGQLQKSCS
HrobustaSyt15b        ----------------------------------------------------------------------------------------------------------------------
LgiganteaSyt15a       MRIISRRLVRTSGHGEEMDHGKKDHGHRDHGEEDHGHRDNGEEDRGHRDRDETNHGHRNHHDKHHEVRDHGKRGDDNSGYKTTYAAQEINAEEGVSNPSAKHNDYIFGNITVTSLGII
LgiganteaSyt15b       ----------------------------------------------------------------------------------------------------------------------
CsavignyiSyt15        -------------------------------------------------------------------------------MITLKRAFQQLWEDNL--TEAEEKK--IPPMVVVPNTFI
CintestinalisSyt15    -------------------------------------------------------------------------------MLSLKRAFQQFREESMPGSTTDEKS--VTTSVVVPETLI
SpurpuratusSyt15      --MDGGFGYADTTTGRDVG-GRFGYAAAALGDINVDGFNVPVAGIIVGSLCGAVVLAGLAFLLYRVYRRKCGARMRYERLGGMQKQPAPAVQIDGKSLATPSVSLKTVPFTLPPAPIP
BfloridaeSyt15a       --------------------------------------------------------------------------------------------------------MIFSGYDVALA--V
BfloridaeSyt15b       ----------------------------------------------------------------------------------------------------------------------
IscapularisSyt15a     -------------------------------------------XSRAADVCLTLPEYQASDRLSSCPRSVNCLDCALRLWWGGDGATHCTRAGGAARMTPEILVWVIAGSSVAGLIVV
IscapularisSyt15b     ----------------------------------------------------------------------------------------------------------------------
DpulexSyt15           ----------------------------------------------------------------------------------------------------------------------
TcastaneumSyt15       ----------------------------------------------------MENQSWTRKLLSTWRKDQQWYADKLAILRSVRSSEPPPGDI--QSPPTGETFKEYVVSEQTPGELQ

                                                                                                           200
CapitellaSyt15        -------------------------------------------------------------------------------------------------------------------XAY
HrobustaSyt15a        NNIIFPSRITTIPFTVPNLIERRRHLWYANDVKSSRDESPSASTPTKPAHSQDPSNNHNNSSSNNTTNNNGNSSNSNGR----WSSEPFLVNSAVSRRSNKHLLQSADHVENNKSSDS
HrobustaSyt15b        -----------------------------------------------------------------------------------------------XLGTIDPSLYRHRRRSYNDMTSK
LgiganteaSyt15a       VGCSAAVLLIIILAIVIFKIRRKRRKFDLQFEDLNE---DTLFSRSCPSSRSSSPKLAKRRSCPDAYASFKPRDSVLIRSMTIERIPDFELPPERVQP-KVSYDRSLSIGPT-TYQQ-
LgiganteaSyt15b       ---------------------------------------------------------------MFRFLKQIPFTVPSQGTPPSGRRHDGRWDENNVGGGSEPASPTGKRPSVEIPGAY
CsavignyiSyt15        T--PVGPQRSPPMPIGGRSPVIGRSTKDNIIPFQLPPTIGSLQVKSESDNISP-TWFPRSPTLSRVGGLYQSCDRLSVDSWSQK-SPRGSPNGSSSQL--NSPQHSPSRSP--DQRTF
CintestinalisSyt15    TAPPSSPRKTTPIAI---SPVFTK--KDKIIPFQLPPTIESFQVQSDIINGSPITRYPRSPTLSRVS-FYQSNDRLSVDSWSQKPSPKGSLSSSNGDYLSNSPTCDVPRSPIEQSKNS
SpurpuratusSyt15      RERIGGFTFPPPNCMPDMLDQPDMRGSAERRPSVTMERRPSYAGCQVPIINQDQQKIMSSSSSAYDQEDGRRDSVGTLSLCSADSAEGGSPTHNTSSPRINNRRPSATEMMSPPVGAF
BfloridaeSyt15a       GACASVLVTVGAVGFLCTWLWRRKQGYHSNVDDLNNGGGDSWLECCWPTRSTSLDNLVPLPTMTTSPSDNHGNEMMARAAVPMETIPFTVPNTERIQP-SGPGEQVKPITTAYNYQQH
BfloridaeSyt15b       ---------------------------------------------------------------------XLPSLLCLRICAEDRRDSEAIYDW--ISSPSSDTSDY-IMSTLASPGMF
IscapularisSyt15a     ----SLVVWLVVAQVRGTKGICRKSGQGDSRPPLLGHDRKSILSKSLAGSPLSARPGDKCPDWVPSVPTLLTPESFNVQSASLTA-------AEPVQP-IDKPGFVRWNSFCSPSAEG
IscapularisSyt15b     ---------------------------------------------------------------------------------------------------------XTSLLGGLNPELY
DpulexSyt15           ------------------------------------------------------------------------------------------------------------------XVTS
TcastaneumSyt15       RHEIL-YSSVAVGVVLLVFISAAVLFFLRRRKTKNADEEHAASSILVYPSQQGTAPQFLTKEIHFSLPPLRSTSLGDLT----EPDDSDVDTESLLLPSVSNQRSNS-------FSCY

                                                                                                 300
CapitellaSyt15        SLGNIDPSLYK--MN--DEDDEYDIPEHHIGRIWFAVEYERESEKLQVTLMKAKNLPSRTLGQGNGCDPFVRIYLVPDDRRYLQSKFKKKTTNPKFEESFVFQVPNKAIDERVLKLTM
HrobustaSyt15a        LVYGIDPTLYD---DQQGVDVETNFEEDNIGRLWFAIQYNPEAERLAVTLIQMKNLPSRIRGINNGCDPYVRICLLPDEKRHLQSQFKKKNCNPKFDENFVFQVSLRNLKERSLKFTV
HrobustaSyt15b        TGGNNDCMEED--DD--DEDDNFDYGPDYLGKIRFGVSYSKESEKLTVVVMQANKLPSRNPGSADTCDPFIKLVLLPDLRSQVRTSFKKKTCNPKFDETFVFQVPVKDFEERILKLTA
LgiganteaSyt15a       CIGSIQPELYR--HPTVPEEDEDDLPPTEHGRLWFSIIYDVAVEQLCVNLIKVKNIPGRGVNHAPR-DPFVKIYLLPDERTCRTSKTRKKSLSPVYNESFTFQVSPDEILTRTLRFSV
LgiganteaSyt15b       ALGSIDPTLYR--IA--DEDDIYEVPRDHIGRVWFAVEYERETEKLLVTLIKARNLPSRSCGSDNGCDPFVRIYLIPDERRYLQSKFRKKTCNPKFEESFVFQVSLRSVEDRVLKLTV
CsavignyiSyt15        FVGKIQPELYK--YTENQDVD---LPISRCGRLWFSVVYDAAVESLTVKVIKVKQLPLRTTSNNPP-DSFVKLFILPDERISQQTKVKLRNCNPAFNETFVFQVSEEDLGKRSLRMSV
CintestinalisSyt15    FLGRIQPELYK--YTENQDID---LPISRCGRMWFSVVYDAAVETLTVKVVKVKQLPLRHPSNNPP-DSFVKIFLLPDERISQQTKVKLRNCNPTFNETFIFQVSEEELQKRSLRMSV
SpurpuratusSyt15      VLGGLNPELYK---LQDEEE-ESNYPDDHIGRIWFAVEYELESERLVVSLIKSKNLPSRTLGNANQCDPLVKVFLLPEERRHLQSKVKRKTCNPRFDESFVFQVTFKALQQRTLRLSV
BfloridaeSyt15a       FLGQLQPELYK--YTDEDDETEKHLPPSPSGRIWFSVVFDASVQTLTVSILKVKHLPPRDKNNASR-DTFVRVYLLPDERMSQQSKVRRKTLNPRFNETFVFQVSETEVRERMLRLSV
BfloridaeSyt15b       SLGAIDPDLYK--AT--QDDSETNFPEDHLGRVWFALEYQREAERLLVSLIKIKNLPSRTPGSSNACDPFVKIFLLPDERRFVQSKFKRKTTNPKFDESFVFQVSYKALQTRTLRLTV
IscapularisSyt15a     LVGNLLPRSYSCGYTWSDDEDTPTAPPSVHGRLWFSLTYRSDDQELDVNVIKAKYLPGRGLNNAPR-DPFVRVYLLPDDDTFRQTEVKRRTLSPKFEETVTFKIEEAEVRRRTLRLSV
IscapularisSyt15b     RVSSGD-GAFG--GP--DDGEERSFPESHRGRLWFALHYEDARESLELRVLKARNLPSRVCGSVNCCDPFVRIQLLPDERRYQQTKFKKKTCNPVFDESYIFQIPGKALSERVLKLTV
DpulexSyt15           NIGSINPDLYK--NPLIPEEDTSNYPEGHLGRFWFSINYDLEQEKLLINLIRIHNLPSRDKDSTTACDPLVRVALLPGERRYHQSSIQRKTCNPVFNEVFGFCVSHKELQEQTVKFTV
TcastaneumSyt15       GLGVIEPALYK---STLDLD-EIQWPEGHIGRIWFSLRYEPSTEKLLVSLLKAKNLPSRTVGTVNSCDPFVRLHLMPDERRYLQSKQKKKTCNPYFDETLVFQVSQKDMSDHILKLTV
```

```
                                                                      400
                            |         |         |         |         |         |         |         |         |         |         |
CapitellaSyt15          YDIDRHK-RHVVIGHALYPLREH-----NYESNERVVMWRDLERE--V----TEMNVERGEMNVSLSYNGHLERLTAVIIEGKAFKKVEPSSS------DFYVKVCLMQQGKVVKSKR
HrobustaSyt15a          FDVTRNK-SHVVIGSVTYPLKMYE-------SSSKVVLWRDLERD--DLSHPGHEGVDSMQLYVSLSYNGDLSRLTVGIYAGKNFKVDEMLTEEESHKTKMYAKVMLMQVNKVLKSKK
HrobustaSyt15b          CDVDRLR-RHNVIGHVIYPLKTSGVLADNAECGKKITVIRDLDLDDCI----ENSSEDKGELLISMTYNAGTERLNVGVYEGRNIKKIVESKNA-----NFYAKVALMKNHCIVKSKR
LgiganteaSyt15a         YDVDKRQ-IRHSLGHVMVAMKDVD-------LTRGEVIWSDLEPM--A---QAASSLG--DIQVSLTYIPQQEKIKIVVMRARNLRPSDYTSES-----GLYARVQFYYGRRAHKTKK
LgiganteaSyt15b         FDVDRHK-RHNVLGHALYPLRDH-----DCQSNERVVVWRDLEKE--V----AETATNKGELSVSTSYNNHLERITIGIFEAKDFKQFDGANSL-----DYYIKVCLMVQNKVIKTKK
CsavignyiSyt15          YDLDKKKSTRRLLGHSLVGLGDVD------PMKANEVLWRDLDDI--AHPGDSGTSLG--ELKISLTYLPGMSRLNVVVLGARSLRKMDL-DET-----GIYVKVLLSQGHRTVKTRR
CintestinalisSyt15      YDVDKKRSTRHLLGHALVGLNDVD------PIKVNEILWRDLDDI--AHPGDSGTSLG--ELKILLTYLPRMNRLNVVVQEARNLRKMDL-DAT-----GIYVKVSLTQGHNVIKSKR
SpurpuratusSyt15        YDVDRSK-KHRLIGHSTYPLKDMDY-----EAHQKVVMWLDLEKE--VTEQSASSETG--DIHFSLNYNNTNERLTVVMAEGKGFKMLDGFQHV-----DSYVKVSLMNIGKVVKAKK
BfloridaeSyt15a         YDMDRRR-VRHSLGHVILPLTEVD-------LAKDELMWRDLEDA--AHVGQDSCLVG--ELNVSLSYLPSMDRLKVVILRAKNFRRLEF-DDT-----AVYVKVQLMHGRKPVKTKK
BfloridaeSyt15b         YDVDRHK-KHRTLGNVLYPFKDF-----DYDDGQKIVLWKDLEEK--S----NMETSDLGEVQFSLTYNNHLERLTVVVLRARGVKKLAGMAAV-----DSCARVTLMNSNHVLKTKK
IscapularisSyt15a       YDIDRRK-VRHCLGHVMLNLEKEP-------LTPEKVIWMELDNV--P---KKSSQIG--EIQISLTCNPFTNRIKAAALQVRRFSRLEE-NST-----SVYVKLSLNHGRKSVKEKA
IscapularisSyt15b       LDNDRGK-RHNVIGHVLFPLKDL-----DPISGERVVAWRDLEAKADVFLMSLQATPEQGELLLALCYKASHERHSVTVLVARGLTAPSEIGPPE----NTQVKVSFLMENKPVKSKK
DpulexSyt15             IDNGRLR-KRQTIGHLLCSLKNI-----SIEDGKQQIITQDISKD-------PSVVGSPGEMHLSLCYHPSADRFTVTVLQARNIRAPNTATP------DSYVRLTLFNQTRAVKTKR
TcastaneumSyt15         IDGSRTK-RRSEIGHVTFPLKDLEIG----DGTEQQLFKLDLEK--------IKSNLG--ELLVSLLYNENLSRLTATVIEARGLKF-QGDKH------EAYVRLTLNQHYRSVKEKR

                                                            500
                            |         |         |         |         |         |         |         |         |         |         |
CapitellaSyt15          TDVAKRNV--SPGFNESFTFKLP-VASLDMASISLTAMQHV-----------------MGQR--DKIVGRLVIGSFMFARGKELEHWNEMLANQKEQISQWHTLT-----------
HrobustaSyt15a          SEMVK--ASNSPVFNEMFKMKLA-PFNLNSVSLSITVYLQT------------------SHSK--EALGRVVLGSFMFARGAELEHWTEMTSKEKEQTSQWHNLTQL---------
HrobustaSyt15b          TDTIKDDR--NPKFNESLSFKQPNENSLMNVCIVVSIFQNL-----------------HAQR--DKMVGRVVIGPYMFARGKELEHWNEVMTKPLENITYWHNIT-----------
LgiganteaSyt15a         TTMQRIHS--DPEIHESFTFSIA-GKQLDTCNVVLSIITSQ----------------TRGPFSREEEYGRVTVGPFMFARGEELIHWQEMVAQSKTPITRWHTLTPYVAPDLH---
LgiganteaSyt15b         SEIVRKTT--CPTFNESFNFKLP-VNSLDTASVTLTAMQHL-----------------TGHK--DKVVGRITLGSFMFARGKELEHWNEMIANQKEQITHWHVLT-----------
CsavignyiSyt15          TSIEDGAA--DPQYNESFSFKVT-AKDMENTCLLLNVVTTGGRGAVHKMTSTLKNTVTRGRYGSEHKYGQVSVGSFMYCRGEHLLHWQEMLAHPSKTIARWHALSEVVNNH-----
CintestinalisSyt15      TSTQDGAA--DPQYNESFSFKVT-SKDMEQTCLLLNVITTGGRGTLHKMTSTLKNTVTRGRYGAELKYGRVSVGSFMYCRGEQLLHWQEMMAQPSKSIARWHTLSEVVTNQ-----
SpurpuratusSyt15        TEIIK--KNPSPTFNESFHFKLP-PDGLDMYSISVAVMQHA------------------PGVKGDKQIGRVVVGPFMYARGKELEHWNDMVSHPKEPISQWHSLT-----------
BfloridaeSyt15a         TAPQQGHC--EQVYNESFSFTVT-AKQLEQTSVQVTIVATG----------------N-GRLSSDEEYGRFVLGPFMFARGEELLHWQEMLAEPRKVITRWHSLTTS---------
BfloridaeSyt15b         TSVVTNSS--DPLYNESFNFKLS-PTQLDTSSLSVAIVQVT-----------------PGDEGSERQVGRVVVGPFMYARGKELEHWTEMVNNQKDMVKRWHALTQ----------
IscapularisSyt15a       TEVREWRSADEVSFTDVFSFGVA-GRYFDSCSLCFTVMVSP----------------DGSDYSR---LGKTVLGPFMYARGEELRHWQDMLTNPRSAVVRWHALEVPTGRDEWKSD
IscapularisSyt15b       TSVCKRNS--EPKFMESFGFKVS-PAGLSAASVILKLSCLA-----------------PPHK--DRCLGRVVLGSFMFARGQAQQHWTDAMAAAPKQIHRWHKLS-----------
DpulexSyt15             TGIIRRSA--DPCYNESFHFRLE-AANVDTTNIVLAIHQPD-----------------NK----DRILGRLVLGSFMFARGRGMAHWNDMFARPREHVQLWHPLPDLN--------
TcastaneumSyt15         TGVARPSRDGAVTFTEGFNFKLA-PSQVDISSLAFHVFQAT------------------SGYGRDKLIGKCVLGSYMFARGKALIQWNTAIANPMEQSQQWHVLCE----------
```
