# Supplementary material for: A manual collection of Syt, Esyt, Rph3a, Rph3al, Doc2, and Dblc2 genes from 46 metazoan genomes - an open access resource for neuroscience and evolutionary biology
Source: BMC Genomics. 2010 Jan 15;11:37. doi: 10.1186/1471-2164-11-37 (PMC2823689; doi:10.1186/1471-2164-11-37)
Supplement: Additional file 29 — Alignment of the vertebrate Syt15 sequences. Amino acid position is marked every hundred amino acids approximately, at the top of each page of the alignment. Splice variants are included and highlighted with black dots where they differ. Intron position and phase is indicated with a coloured bar between amino acids. Black bars indicate phase 0 introns. Red bars indicate phase +1 introns. Blue bars indicate phase +2 introns. The widely conserved motif of unknown function, just upstream of the C2A domain, is indicated. X residues indicate where a portion of sequence is missing. [file 1471-2164-11-37-S29.PDF]

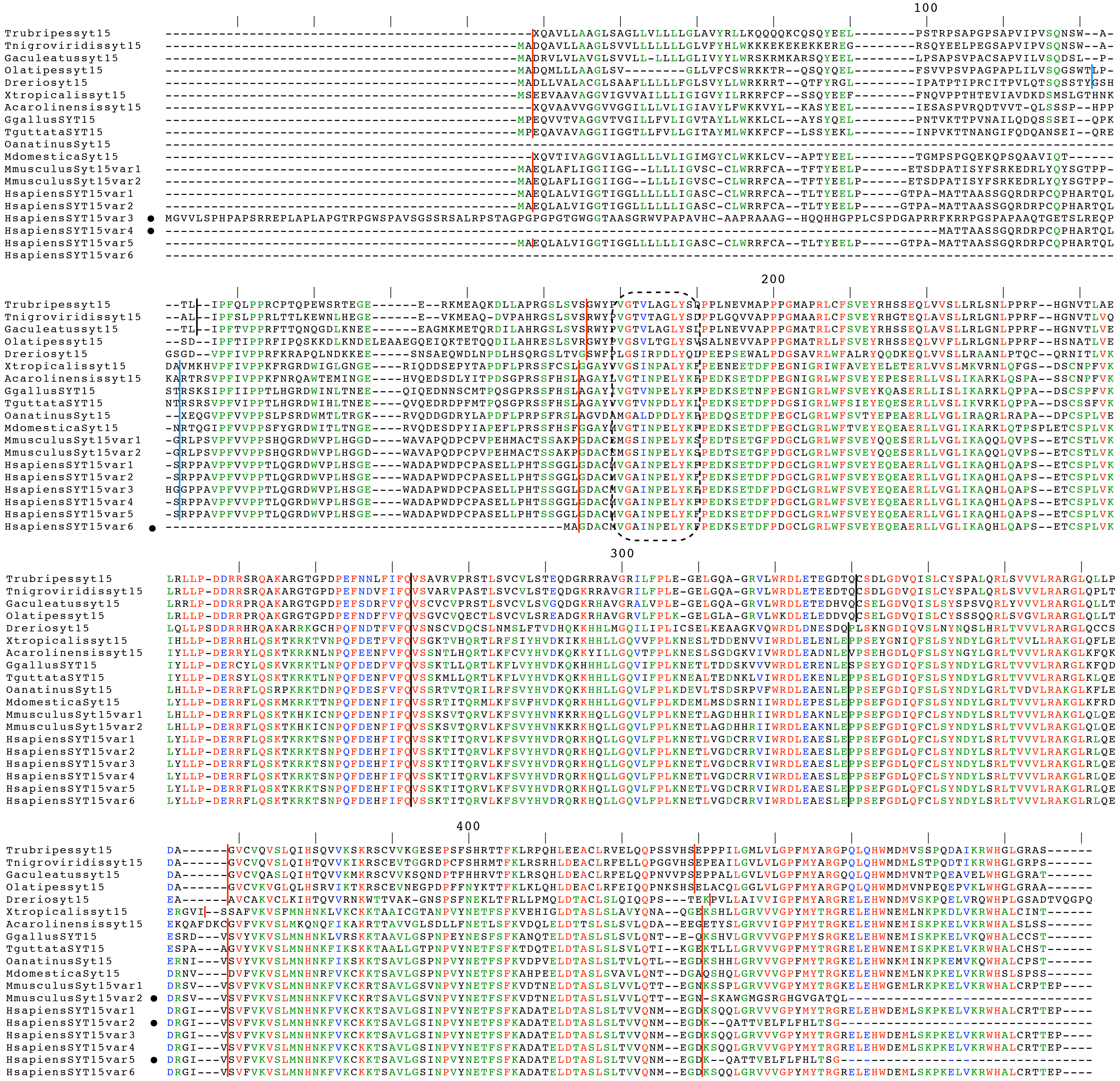
100
200
300
400
Trubripessyt15
Tnigroviridissyt15
Gaculeatussyt15
Olatipessyt15
Dreriosyt15
Xtropicalissyt15
Acarolinensissyt15
GgallusSYT15
TguttataSYT15
OanatinusSyt15
MdomesticaSyt15
MmusculusSyt15var1
MmusculusSyt15var2
HsapiensSYT15var1
HsapiensSYT15var2
HsapiensSYT15var3
HsapiensSYT15var4
HsapiensSYT15var5
HsapiensSYT15var6
