# Supplementary material for: A manual collection of Syt, Esyt, Rph3a, Rph3al, Doc2, and Dblc2 genes from 46 metazoan genomes - an open access resource for neuroscience and evolutionary biology
Source: BMC Genomics. 2010 Jan 15;11:37. doi: 10.1186/1471-2164-11-37 (PMC2823689; doi:10.1186/1471-2164-11-37)
Supplement: Additional file 30 — Alignment of the invertebrate Syt16 sequences. Amino acid position is marked every hundred amino acids approximately, at the top of each page of the alignment. Splice variants which specify alternative N-termini lacking TM domains, are included and highlighted with black dots where their sequences differ. TM domains are highlighted in blue. Intron position and phase is indicated with a coloured bar between amino acids. Black bars indicate phase 0 introns. Red bars indicate phase +1 introns. Blue bars indicate phase +2 introns. X residues indicate where a portion of sequence is missing. [file 1471-2164-11-37-S30.PDF]

```
                                                                                                                      100
                                        |         |         |         |         |         |         |         |         |         |         |         |
CapitellaSyt16                ---------------------------------------------------------------------------------------------------------------------------------
HrobustaSyt16                 ---------------------------------------------------------------------------------------------------------------------------------
LgiganteaSyt16                ----------------XPTEAVAFLGAVGFFLVVLILFFCYM-NKTLCFKNCGGFPCIDR--PTKHKKANSLGESISYSFTTMHKFLIERYHQFAPYEI-------SVRRKTAASLAEEGQCLSDDLEK
CsavignyiSyt16var1            ---------------------------------------------------------------------XVSTAAVVFLSVIVGITAIIVVFWMFFTK-K-----ACFKQCGGCDGFLEPQRHS--ADA
CsavignyiSyt16var2          ● -------MSTAMCCFHALFAHMGSGGEAFSRDLVATVKKDHAKK--KRTNSINSNDSLNDPT-YTDGWGDETNQARLEITVKENNESQPSVSTRIRQS-M-----RKLRS-SLLDLRASTTTVN--TNN
CintestinalisSyt16var1        ------------------------------------------------------------MVAIASTTHTVSTAAVIFLSVIVGITAFLVVFYMFFTK-K-----ACFKNCGGCDGLLEPGRYS--GDE
CintestinalisSyt16var2      ● -------MSTAMCCFHGLFAHMGSGGSSFSRDVVAVLKTDRAAKNRKQTVSVSSDDSLNDPDEIVENWNDSSSKPRLSITADADEKKEAPRFARVSTRIK-----NSMRAIRSSLSDLRASTTSINTNN
SpurpuratusSyt16var1          ---------------------------------------------------------------------------------------------------------------------------------
SpurpuratusSyt16var2          ---------------------------------------------------------------------------------------------------------------------------------
SpurpuratusSyt16var3          ---------------------------------------------------------------------------------------------------------------------------------
BfloridaeSyt16                ---------------------------------------------------------------------------------------------------------------------------------
IscapularisSyt16              ----------------XPTEATAFLGAVGMILVVLVVFYLYL-NKKLCFTTVGGFPCCDVAPTPREKEKKVAARDLGEVFVFVHWFYTAGCMREGITNH-------RIQKPHSNYPQTEGPLHFLEEVE
DpulexSyt16                   ----XGSWMEETEPWWPGLLVAVVSSLILLLFLVTLLVHHLPPLQKWLLPVLTRIAPPTTNNKTAQTANPTQQQTDGYDQVDAGSSSQQPHHPLYPSSRDH----HSKESEQQRRQQHRSSAEAVLRQR
TcastaneumSyt16               ------MLVISEHSYLGGTDGGTVLGAIAGMGALLLVFLLYIKNKRWWWGSVGGMGCCDEACPP------VSSRPVPSPPVPATSFTPAPLKHKLVKAY-------SFDGGDSSSESEMGGSKFRQNST
NvitripennisSyt16             -------------------------------------------------------------------------------------------------------------------MTLAGPDHFLAV--
AmelliferaSyt16               -------------------------------------------------------------------------------------------------------------------MILAGSDHFLAVPV
AgambiaeSyt16                 MRNDLKRPEGSTPCDNAHMELTTFFGLALGFVALILVLFLYV-HKIRCFGAAPPFLPFDEQLRAEKTFHRIRNRFAYDGNNSSDSEDDTLRRLKLDP--------CGAGYGGGVGVGGGGSISCYHANE
DmelanogasterSyt16            MIVTSASGLDSTPTLGT-IEVTTLLGAFFGVLVLLLLLFLFI-SRKCCFHYRHAINCCDERHLAVKCVQKITRKRRYE-NTSSDSEEDILRRLRWHQQHQL----GEKPGGYGLGISQANPLTAQSFNY
DsimulansSyt16                MIVTSASGLDSTPTLGT-IEVTTLLGAFFGVLVLLLLLFLFI-SRKCCFHYRHAINCCDERHLAVKCVQKITRKRRYE-NTSSDSEEDILRRLRWHQQHQL----GEKPGGYGLGISQANPLTAQSFNY
DsecheliaSyt16                MIVTSASGLDSTPTLGT-IEVTTLLGAFFGVLVLLLLLFLFI-SRKCCFHYRHAINCCDERHLAVKCVQKITRKRRYE-NTSSDSEEDILRRLRWHQQHQL----GEKPGGYGLGISQANPLTAQSFNY
DerectaSyt16                  MIVTSASGLDSTPTLGT-VEVTTLLGAFFGVLVLLLLLFLFI-SRKCCFHYRHAINCCDERHLAVKCVQKITRKRRYE-NTSSDSEEDILRRLRWHQQHQL----GEKPGGYGLGISQDNPLTAQSFSY
DyakubaSyt16                  MIVTSASGLDSTPTLGT-VEVTTLLGAFFGVFVLLLLLFFFI-SRRCCFHYRHAINCCDERHLAIKCVQKITRKRRYE-NTSSDSEEDILRRLRWHHQHQL----GEKPGGYGLGISQANPLTAQSFNY
DananassaeSyt16               MIVTSASGLDSTPTLGT-VEVTTLLGVFFGVLVLLLLLFLYI-SRKCCFHYRHAINCCDERHLAAKCVQKITRKRRYE-NTSSDSEEDILRRLRWHQQHQL----GDKHGNYGMGINQANPLTAHSFNY
DpseudoobscuraSyt16           MIVTSASGVDSTPTLGT-VEVTTLLGVFFGVLVLLLLLFLYI-SRKCCFHHRHGINCCDERHLAAKCVQKITRKRRYE-NTSSDSEEDILRRLRWHQQNQH----SDKPIGYGMSISQANPLTAHSFNY
DpersimilisSyt16              MIVTSASGVDSTPTLGT-VEVTTLLGVFFGVLVLLLLLFLYI-SRKCCFHHRHGINCCDERHLAAKCVQKITRKRRYE-NTSSDSEEDILRRLRWHQQNQH----SDKPIGYGMSISQANPLTAHSFNY
DwillistoniSyt16              MIVTSASGVDSTPTLGT-VEVTTLLGAFFGVLVLLLLLFLYI-SRKCCFHHRHGINCCDERHLAAKCVQKITRKRRYE-NTSSDSEEDILRRLRWHQQSQQ----NDKLAGYGMNSNQINPLTAHSFNY
DvirilisSyt16                 MIVAS--GVDSTASLGT-VEVTTLLGAFFGVLVLLLLLFLYI-SRRCCFHHRHGINCCDERHLAAKCVQKITRKRRYE-NTSSDSEEDMLRRLRWHQQNNNLLP-NGKFIGYGMGIDQRNPLTGHNFNY
DmojavensisSyt16              MIVTS--GVDSTVTLGT-VEVTTLLGAFFGVLVLLLLLFLYI-SRRCCFHHRHGINCCDERHLAAKCVQKITRKRRYE-NTSSDSEEDMLRRLRWHQQNNNILP-NGKFVGYGMGIDQRNPLTGHSFNY
DgrimshawiSyt16               MIVTS--GVDSTASLGT-VEVTTLLGAFFGVLVLLLLLFLYI-SRRCCFHHRHGINCCDERHLAAKCVQKITRKRRYE-NTSSDSEEDMLRRLRWHQQNNNLLPPNGKLVGYGMGSEQMNPLTGHSFNY

                                                                                                    200
                                        |         |         |         |         |         |         |         |         |         |         |         |
CapitellaSyt16                ---------------XVPVEAYAFMGAVVVFLVLLVLFFLYLNKMVCFHEIGGFPCIDRR---------------HGDPEKDRHEVLKEEDDDEAICVDDSEGHR-----------KDYGSVRTGRLGS
HrobustaSyt16                 ---------------------------------------------------------------------------------------------------------------------------------
LgiganteaSyt16                DKLEV-SDGEGVEADEED--NLAEGG-----------------VEDAHQ----------------------------SPTVEETAALVPADKAGATAVSKPVFENRG------------Y-----EGER
CsavignyiSyt16var1            ALAKSYDDSDSDSSSDSEEETLRQMRKTIRHKEHSSDNISGSHMKTSKSDLGPQPKSA-------------------SPTLKGDNEVVGVNESFSSQDLLKLDSESP---------ESEAAPPQGGDDK
CsavignyiSyt16var2            SNSKSYDDSDSDSSSDSEEETLRQMRKTIRHKEHSSDNISGSHMKTSKSDLGPQPKSA-------------------SPTLKGDNEVVGVNESFSSQDLLKLDSESP---------ESEAAPPQGGDDK
CintestinalisSyt16var1        ALAKSFDDSDSDSSDDSEEETMRQMRKTVRRSSARKNNGSSSDII---DDQQPSTSNI-------------------DTTLNTNPSTPSSGEKQMNEQQTVMDGTSPNE--APKQMESPASHVSTNDGK
CintestinalisSyt16var2        NNTKSFDDSDSDSSDDSEEETMRQMRKTVRRSSARKNNGSSSDII---DDQQPSTSNI-------------------DTTLNTNPSTPSSGEKQMNEQQTVMDGTSPNE--APKQMESPASHVSTNDGK
SpurpuratusSyt16var1          ----------------XPLAALIFLGVIFIFLTFLVLLFMYLNRIACFASCGGCP-CMEE---------------KKKKKEKQAILGESFGYEDEGSSSDSEDETILRYQQSLLPPKSKQPTISTKLKR
SpurpuratusSyt16var2        ● ------------------------------------------------------------------------------------MVAKLFLLCCDGDSDSNEDQH-------LVVVKNVKSCKKVHFXR
SpurpuratusSyt16var3        ● -----------------------------------------XTRPGFSSLIGFAPRSVRE---------------PTEVKDPDEDLGEDEGHYSDPSIAQSVNES---------------DVASIANSK
BfloridaeSyt16                -------------------------------------------------------------------------------------------MASELNKEGRGGLS-----------SGEESVATFS-GE
IscapularisSyt16              EPFSL-TGKGAGQLPQPDLVSLVESGRGA--------SSGADTADEEDG----------------------------ASHRSRTVGGMPSQVSTSATVRSALHYENG------------A-FLKAEDSP
DpulexSyt16                   AANRSQDLAHHQQQQQRLLLDHEDIQRLDEWTAHHQPSAANSQRQSSLAETDCDVSNQLQLQRCPVATAIESHDPDVNSSIRPVGPDVTGHHHPTTTDDVVLDMPSA-----RCTLNKPSSRTLSRSLA
TcastaneumSyt16               S--GT-TGPYYPPRQVHDLISLVEKGRVGVSSNSSCCSSTTSSVGEKHG----------------------------VPATRVTAQINDNHGRLSTDLRETKIDNQG------------IDYQKIETSS
NvitripennisSyt16             ETTAFLGACVGFVVLLVVVALYLSRKWYFTPAATTTSLFGAVCVPLCESSNSSAAQSLGTLSPLVIAPVEAEAELRVSARTGKNYPFSDPETSSDSEEDALRRLSSRSQPPPDTLTIQAEDGLPSIKQN
AmelliferaSyt16               EATAFLGAIAGFVVLLLALFLYLSRKWCFTPPSTTT-LFGGVCVPLCESNNGSTSQ--------------------ITKNIGKAFSYSDPETSSDSEED-VRRLNSH--PPPDTLTIQAEDG-PTILDA
AgambiaeSyt16                 AVAGLDQSVVAHRHKSRDPLAMAEGGKIGMPHSSNDCSSGSSNEGNLDYGGTRLSLDAHLSNDRLAAGAA------PARLDGRLTGSAELKLRGEPPLLPELTKESD-------LERDLVPGYRKKTTK
DmelanogasterSyt16            HQAGASADL-QRVTVSRDPLAIAERGKVGIPSSHSECSSNDSMEVSVDSHTGVLTGLSKATTTVPHPATAF--RNPCGEHRAKTQ-ALRYQHRVNVAAPPKLDKERD-----NVLVVPMVNAYSINNNN
DsimulansSyt16                HQAGAGADL-QRVTVSRDPLAIAERGKVGIPSSHSECSSNDSMEVSVDSHTGVLAGLSKATTTVPHPATAF--RNPCAEHRAKTQ-ALRYQHRVNVAAPLKMDKERD-----NVLVVPMVNAYSINNNN
DsecheliaSyt16                HQAGAGADL-QRVTVSRDPLAIAERGKVGIPSSHSECSSNDSMEVSVDSHTGVLGGLSKATTTVPHPATAF--RNPCAEHRAKTQ-ALRYQHRVNVAAPLKPDKERD-----NVLVVPMVNAYSINNNN
DerectaSyt16                  HQAGVGADL-QRVTVTGDPLAIAERGKVGIPSSHSECSSNDSMEVSVDSHIGFLTGLSKATTTVPHPATAF--RNPCAEHRVKTQ-ALRYQHRVDVAAPPKLDKERD-----NVLV----NDYSINNNN
DyakubaSyt16                  HQAGAGADL-QRVTVTRDPLAIAERGKVGIPSSHSECSSNDSMEVSVDSHISILTGLSKGTTTVPHPATAF--RNPCAGHRPTTQ-ALKYQHRVDVAAAPKLDKERD-----NVLVVPMVNGYSINNNN
DananassaeSyt16               HHAG--SDL-QRVTVTRDPLAVAERGKVGIPSSHSECSSNDSMEASVDSHTGILTGESKGS----HPASSY--RNPYADHRAKTQ-ALRYQHRVEVVMPPKHDKERD----SNSVVVPMSNSCNNNNSS
DpseudoobscuraSyt16           HQSD--ADL-QRVTVTRDPLAIAERGKVGIPSSHSECSSNDSMEASVDSHTGILTGETKA-----HPVTAF--RNPYTDNRAKTHHSQRYHHRVEV-LPPKLEKDNA------VVVMPVANSFINNNN-
DpersimilisSyt16              HQSD--ADL-QRVTVTRDPLAIAERGKVGIPSSHSECSSNDSMEASVDSHTGILTGETKA-----HPVTAF--RNPYTDNRAKTHHSQRYHHRVEV-LPPKLEKDNA------VVVMPVANSFINNNN-
DwillistoniSyt16              HQPD--ADL-QRVTVTRDPLAIAERGKVGIPSSHSECSSNDSMEASVDSHTGILTGERETKGFPITALRQS---SYPSYHRSKILHTQRYQQHKVEVLAPKQDKIQDNRDRDNVVVVPITNTLSNNNNN
DvirilisSyt16                 HQ----ADL-QRVTVTRDPLAIAERGKVGIPSSHSECSSNDSIEASVDSHTGILTGESRAVP--VASLRNSYAKSQKYQHKVDVQPPKPERERDSVVVVPIA-SLTNNNN--NITANNNYNQSCNNNNN
DmojavensisSyt16              HQ----ADL-QRVTVTRDPLAIAERGKVGIPSSHSECSSNDSIEASVDSQTGILTADSRAVP-AVSALRHSYAKSQKYQHKVDVLPQKVEKDRDSVVVVPITTSLNNNNN--NNSSITNHNYNQNNNNN
DgrimshawiSyt16               HQ-Q--ADL-QRVTVTRDPLAIAERGKVGIPSSHSECSSNDSIEASVDSHTGILTGESRAVP--MSSLRSSYAKSQKYQHKVDVLPQKSEKDRDSVVVVPITSIQQQNNN--NISTANN---NCNHSNS
```

```
300
CapitellaSyt16              DVSERSQTKKSRVNKGSVRSLAKSEQLFDVSDLQDREATFISK-------------------C------GSLEVTFDYDMGSGKMAVTVHQARDIPSKER-GGASSTQVRLMLLPTKKIRQKTKVKIGD
HrobustaSyt16               -----------------------------------------------------------------------------------XLTLIIHEARDLPSRER-GGAIQTQVRMLLLPIKKHRHKTKVVVAA
LgiganteaSyt16              TPSQSFTSSNPSLSTERLSNDEHLFDVSD---LQNEP---PLISKCGN-----------------------LEVTFRYEAKRGKIHIKILQALEIPAKDR-GGASHVQVRAMLLPTKRQKHKTKVK-TG
CsavignyiSyt16var1          QWTIDDTGSVYSRDMSSSMLEEDEVAVAIAAKPKSLDGESISLSMDSGL-----PISACGM----------IDIALQYSLSDRKLQITIIEAKELPSKDR-GGASIIQVRAVMLPAKRKRYKTKVQHIN
CsavignyiSyt16var2          QWTIDDTGSVYSRDMSSSMLEEDEVAVAIAAKPKSLDGESISLSMDSGL-----PISACGM----------IDIALQYSLSDRKLQITIIEAKELPSKDR-GGASIIQVRAVMLPAKRKRYKTKVQHIN
CintestinalisSyt16var1      EWNIDDSESVYSRELSSSLMEEDEVAVAIAAKPSPLDGDSVDISMEDTT-----PISACGV----------IDIAVQYSISDRKLQITIIEAKELPSKDR-GGASIIQVRAVMLPAKRKRYKTKVQHIN
CintestinalisSyt16var2      EWNIDDSESVYSRELSSSLMEEDEVAVAIAAKPSPLDGDSVDISMEDTT-----PISACGV----------IDIAVQYSISDRKLQITIIEAKELPSKDR-GGASIIQVRAVMLPAKRKRYKTKVQHIN
SpurpuratusSyt16var1        NTTITIGGVRTDDVEQGDISLDQEGHDYPVESEFVDDLPLVGSDDDDDLVVEQLPPSDAGPSI------GDLELAFQYNGEARRMNVTIIKCHNLPTKDE-GGASSFRLRLLLLPSKRQRAKTHIRESK
SpurpuratusSyt16var2        KNSL-FQGVRTDDVEQGDISLDQEGHDYPVESEFVDDLPLVGSDDDDDLVVEQLPPSDAGPSI------GDLELAFQYNGEARRMNVTIIKCHNLPTKDE-GGASSFRLRLLLLPSKRQRAKTHIRESK
SpurpuratusSyt16var3        SESVILESVRTDDVEQGDISLDQEGHDYPVESEFVDDLPLVGSDDDDDLVVEQLPPSDAGPSI------GDLELAFQYNGEARRMNVTIIKCHNLPTKDE-GGASSFRLRLLLLPSKRQRAKTHIRESK
BfloridaeSyt16              VVSADEMSVISSAPDPNWKNKAQDRGYYDVDQPYEEDSQLISK-------------------C------GVLEVAFAYDAPNRKMTVSIVQARDVPSKDR-GGTGGYQVHMVLLPTKKQRCKTKVRQGN
IscapularisSyt16            LSLMACGGTDVSCLPRTTVPGLQLRCVGAT-GLRGDVGPSESTTRCGA-----------------------LELTFNYSPASRKLTVGVLRAHDLPSRDR-GGANSVQVRLLLLPHKRQKFKTKIRQAS
DpulexSyt16                 VLTATGMDRIRSMIHLDTGSRTRAESMIDMTDMSSTFGESESVTGSVIL-----PGPNRTANK------AVIEMGFSYNSVSGKLVVRLVELRQLTSASR---NTSVQLRLLLLPDRKQRCKSKLRYTN
TcastaneumSyt16             TIVQTDINSNRDCIVVMNTDTDERNDLMCC--IRSEP-ETQLVNKCGQ-----------------------LEIALLYDAPMRKMTVHVLQARDIPSRDR-GQPTHTQVRLILLPSKKQKHKTKIR-SG
NvitripennisSyt16           GTEEQPEERPEERPEERPEEQPEEQPEEEQEQQQQQQQQQCVVEVGSPSLLIEESHAASSSLAVSGEEMASLEVAFLYDAPMREMTVHVLQGRNYPA-----GIGGSQVRLVLLPSKKQRRKTRVRQGS
AmelliferaSyt16             GTTSS------SCTEEHSSNDQQQCVVEVGASGLDNREQEVEVEQNGST-----TNDVITTMGTITEEVGSLEVAFLYDAPMREMTVHVLQGRNYPE-----GIGGSQVRLVLLPSKKQRRKTRVRQGT
AgambiaeSyt16               GATNGSRGE-GLVVAAASTASSPLYDTSDLKSLKSDAGGAVVPGPAGYD-----QPAGSGAHPPATGSNGTLEISLLYDAPMRKMTVHVLQARGIASRGDKGQLTHTQVRLLMLPAKRQKHKTKIR-SG
DmelanogasterSyt16          N--SITTS--NNHPSSNNNDDEPLFDTSDLRSIKSDD---LLVGVDQKE-----PAVQRGPIE--------LELSLLYDAPMRKMTVHVMQAKNLPPLGS-GQTTHTQVRMLMLPSKKQKLKTKIR-SG
DsimulansSyt16              NN-SITTS--NNHPSSNNNDDEPLFDTSDLRSIKSDD---LLVGVDQKE-----PPVQRGPIE--------LELSLLYDAPMRKMTVHVMQAKNLPPLGS-GQTTHTQVRMLMLPSKKQKLKTKIR-SG
DsechelliaSyt16             NNNSITTS--NNHPSSNNNDDEPLFDTSDLRSIKSDD---LLVGVDQKE-----PPVQRGPIE--------LELSLLYDAPMRKMTVHVMQAKNLPPLGS-GQTTHTQVRMLMLPSKKQKLKTKIR-SG
DerectaSyt16                ---SITTS--NNNPSSNNNDDEPLFDTSDLRSIKSDD---LLVGVDQKE-----SPVQRGPIE--------LELSLLYDAPMRKMTVHVMQAKNLPPLGS-GQTTHTQVRMLMLPSKKQKLKTKIR-SG
DyakubaSyt16                ---SITAS--NNNPSSNNNDDEPLFDTSDLRSVKSDD---LLVGVDHKE-----SPVQRGPIE--------LELSLLYDAPMRKMTVHVMQAKNLPPLGI-GQATHTQVRMLMLPSKKQKLKTKIR-SG
DananassaeSyt16             VANNNNTI--STNNNNNNNDDEPLFDTSDLRSIKSDD---MLVGGDAKV-----TP--RGPIE--------LELSLLYDAPMRKMTVHVMQARSLPPLAS-GQPTHTQVRMLMLPTKKQKLKTKIR-SG
DpseudoobscuraSyt16         -----TTI--PNNNNNN--DDEPLFDTSDLRSVKSDD---MLVG--DSK-----APASRGPIE--------LEMSLLYDAPMRKMTVHVMQARCLPPLGN-GQQTHTQVRMLMLPNKKQKHKTKIR-SG
DpersimilisSyt16            -----TTI--PNNNNNNNNNDDEPLFDTSDLRSVKSDD---MLVG--DSK-----APASRGPIE--------LEMSLLYDAPMRKMTVHVMQARCLPPLGN-GQQTHTQVRMLMLPNKKQKHKTKIR-SG
DwillistoniSyt16            NNNNNNTSTINNNNNNNTNDDEPLFDTSDLRSIKSDD---MLVNDAKGT-----PP--RGPIE--------LEMSLLYDAPMRKMTIHVMQARCLPPLAT-GQQTHTQVRMLMLPNKKQKHKTKIR-SG
DvirilisSyt16               SNNNNTTTNSSNNNNNNNNDDEPLFDTSDLRSIKSDD---ILVSDPKST-----AP--RGPIE--------LEMSLLYDAPMRKMTVHVMQARSLPPLGN-GQQTHTQVRLLMLPNKKQKHKTKIR-SG
DmojavensisSyt16            SNTINSTATNSNNNNNNN-DDEPLFDTSDLRSIKSDD---MLVSDPKLT-----AP--RGPIE--------LEMSLLYDAPMRKMTVHVMQARSLPPLPN-GQQTHTQVRMLMLPNKKQKHKTKIR-SG
DgrimshawiSyt16             SSSNINSNNPNNNNNPNNNDDEPLFDTSDLRSIKSDD---IPVSDSKIT-----AP--RGPIE--------LEMSLLYDAPMRKMTVHVMQARSLPPLAN-GQQTHTQVRMLMLPNKKQKHKTKIR-SG

400
CapitellaSyt16              C----PDFQEMFTF-KVPPEDVNNFGVRFRLY-GCERMRRERMIGETVMGFASLRHDTATTHWLTL--EPRSNLSHADSKFDVSS-LSKSDSAS--STQSMQH-GGMPELLIGLSYNGTTGRLLVEVIK
HrobustaSyt16               ESN--PQFNETFDF-KVPTDDLTSMGLRMRLY-GCERLKKDRLIGETIVNFNRLNTN---NVPFWTPLDPRSNLMHGDSMSSVSSNLTHSESDS--STHSLNHAGTL-ELMLGVAYNGVTGRMAVEVIK
LgiganteaSyt16              DN---PVFNEDIVFNKIFPDNVQSMGVRFRLY-GVERMRRERMIGESIVGFASLNLD--EEQTHTVVLEPRSNLS-GDSGFDVAS-LSKSDSAS--STQSLQHGG-MPELLLGLGYNGTTGRLAVEVIK
CsavignyiSyt16var1          A----PRFAETFKISQVSPEDLRRMGLRLRLY-GIGKVR-DRLIGETTVRFDELNLIREPQLTVTLQLEPRTNVNRGDNYELSGM-KEYVDSS---SVATLTHGGSLPEILLALAYNQMTGRLSVEVVK
CsavignyiSyt16var2          A----PRFAETFKISQVSPEDLRRMGLRLRLY-GIGKVR-DRLIGETTVRFDELNLIREPQLTVTLQLEPRTNVNRGDNYELSGM-KEYVDSS---SVATLTHGGSLPEILLALAYNQMTGRLSVEVVK
CintestinalisSyt16var1      S----SRFAETFKVSQVSPEELRRLGLRVRLY-GIGKVR-DRLIGEATVRFDELNLIRDPQLTVTLQLEPRTDVNRGDQLDVTGS-QGYIDTS---SVATLAHGGSLPELLLSLSYNEMTGRLCVEVVK
CintestinalisSyt16var2      S----SRFAETFKVSQVSPEELRRLGLRVRLY-GIGKVR-DRLIGEATVRFDELNLIRDPQLTVTLQLEPRTDVNRGDQLDVTGS-QGYIDTS---SVATLAHGGSLPELLLSLSYNEMTGRLCVEVVK
SpurpuratusSyt16var1        E----PLFKELFRFSRIFPHEVVSTALRCRLY-GCERMRKEKLIGESIIKFSSLNIANKQIIKVRIDPRSDVSKNGSSPYSSTSD-LSDSNSSS--SLQSMSQ-GSVPEILIGLAYNATTGRLDVEVIK
SpurpuratusSyt16var2        E----PLFKELFRFSRIFPHEVVSTALRCRLY-GCERMRKEKLIGESIIKFSSLNIANKQIIKVRIDPRSDVSKNGSSPYSSTSD-LSDSNSSS--SLQSMSQ-GSVPEILIGLAYNATTGRLDVEVIK
SpurpuratusSyt16var3        E----PLFKELFRFSRIFPHEVVSTALRCRLY-GCERMRKEKLIGESIIKFSSLNIANKQIIKVRIDPRSDVSKNGSSPYSSTSD-LSDSNSSS--SLQSMSQ-GSVPEILIGLAYNATTGRLDVEVIK
BfloridaeSyt16              N----PVFKESFRFSRVNQNELPDMGVRFRLY-ACERMKKERLIGEKLVGFSNLKLDQRESMAVTLPLEPRSNLSGGDSQFSLSG-LSRSDSGS--SSQSLTHAGGVPELLVGLAYNATTGRLSVEVIK
IscapularisSyt16            QD---TRFDESFAFSRISPEEVNTMGLRVRLY-GCERMRREHLVGETVVPFSFLGLD--QQTTLWLVLEPRSNLAHWDSKCEISS-LARSDSTG--STQSMQHGG-LPELLLGLAYNGTTGRLAAEVIK
DpulexSyt16                 EDGSTVSLMETFVFSRIEPGELSSTGIRLRLY-CSERLRRERLLGEAFVGLASMTLDHQREQSLLVALETKSGPFKKSLAALGRS-TSSSSSRGDISPTSIGPNG-IPELLLGLAYNGTTGRLSVEILR
TcastaneumSyt16             EN---PQYMESFVLHRVNPEDVNSMGIRLRLY-GCERMRRERLIGEAVVGFTHINLE--LENNFWLNLEPRVDTTLTGCAGELTS-LARSDSTG--STHSMQHGG-VPELLLGLCYNATTGRLSIEVVK
NvitripennisSyt16           S----PQYMESFLLPRVNPEDVNAMGVRVRVYLWGGRMRRERLLGEARVSFDRINLR--LETTLWLALQPPPSSSVSDRVHRRLS-LTRSDSTG--SQHSMK-GGSVAEILIGLTYNGVTGRLSVEIIK
AmelliferaSyt16             S----PQYMESFLLPRVNPEDVNAMGVRLRVYLWGGRMRRERLLGEARVSFDQINLQ--LETTLWLTLQPPPFSSVQDW-GTTSS-LTRSDSTG--SQHSMK-GGSVAEILIGLAYNGTTGRLSVEIIK
AgambiaeSyt16               EC---PQFMESFLLHRVNPEEVNSMGLRIRVY-GCERMRRERLIGETIVSFANIDLE--LETNLWLPLESRSSSTDTASTSDLLS-IARSDSAG--STTSMQHGG-VPELLLGLGYNGITGRLTVEIVK
DmelanogasterSyt16          EN---PQYMESFLLHRVNPEDVNNMGLRVRLY-GCERLRKERLIGEAYVSFATVDLE--LETNLWLPLEPRNTSSGLGSTSDLLS-LARSESAG--STSSMQHGG-VSELLLGLSYNGVTGRLSVEIIK
DsimulansSyt16              EN---PQYMESFLLHRVNPEDVNNMGLRVRLY-GCERLRKERLIGEAYVSFATVDLE--LETNLWLPLEPRNTSSGLGSTSDLLS-LARSESAG--STSSMQHGG-VSELLLGLSYNGVTGRLSVEIIK
DsechelliaSyt16             EN---PQYMESFLLHRVNPEDVNNMGLRVRLY-GCERLRKERLIGEAYVSFATVDLE--LETNLWLPLEPRNTSSGLGSTSDLLS-LARSESAG--STSSMQHGG-VSELLLGLSYNGVTGRLSVEIIK
DerectaSyt16                EV---PQYMESFLLHRVNPEDVNNMGLRVRLY-GCERLRKERLIGEAYVSFATVDLE--LETNLWLPLEPRNTSSGLGSTSDLLS-LARSESAG--STSSMQHGG-VSELLLGLSYNGVTGRLSVEIIK
DyakubaSyt16                ET---PQYMESFLLHRVNPEDVNNMGLRVRLY-GCERLRKERLIGEAYVSFATVDLE--LETNLWLPLEPRNTSSGLGSTSDLLS-LARSESAG--STSSMQHGG-VSELLLGLSYNGVTGRLSVEIIK
DananassaeSyt16             EN---PQYMESFLLHRVNPEDVNNMGLRVRLY-GCERLRKERLIGEAYVSFATVDLE--LETNLWLPLEPRNTSSVLGSTSDLLS-LARSESAG--STSSMQHGG-VSELLLGLSYNGVTGRLSVEVIK
DpseudoobscuraSyt16         EN---PQYMESFLLHRVNPEDVNNMGLRVRLY-GCERLRKERLIGEAYVSFATVDLE--LETNLWLPLEPRNTSSVLGSTSDLLS-LARSESAG--STSSMQHGG-VSELLLGLSYNGVTGRLSVEIIK
DpersimilisSyt16            EN---PQYMESFLLHRVNPEDVNNMGLRVRLY-GCERLRKERLIGEAYVSFATVDLE--LETNLWLPLEPRNTSSVLGSTSDLLS-LARSESAG--STSSMQHGG-VSELLLGLSYNGVTGRLSVEIIK
DwillistoniSyt16            EN---PQYMESFLLHRVNPEDVNNMGLRVRLY-GCERLRKERLIGEAYVSFATVDLE--LETNLWLPLEPRNTSSVLGSTSDLLS-LARSDSAG--STSSMQHGG-VSELLLGLSYNGVTGRLSVEIIK
DvirilisSyt16               EN---PQYMESFLLHRVNPEDVNNMGLRVRLY-GCERLRKERLIGEAYVSFATVDLE--LETNLWLPLEPRNTTSLLGSTSDLLS-LARSDSAG--STSSMQHGG-VSELLLGLSYNGVTGRLSVEIIK
DmojavensisSyt16            EN---PQYMESFLLHRVNPEDVNNMGLRVRLY-GCERLRKERLIGEAYVSFATVDLE--LETNLWLPLEPRVTTSLLGSTSDLLS-LARSDSAG--STSSMQHGG-VSELLLGLSYNGVTGRLSVEIIK
DgrimshawiSyt16             EN---PQYMESFLLHRVNPEDVNNMGLRVRLY-GCERLRKERLIGEAYVSFATVDLE--LETNLWLPLEPRNTSSLLGSTSDLLS-LARSDSAG--STSSMQHGG-VSELLLGLSYNGVTGRLSVEIIK

500                                                                                                                      600
CapitellaSyt16              GSNFKNMAMS--RAPDTYVKLTLMAPNGQEVSRSKTSTRRGQPN-PLYKETSVFQVALFQLPEVTLMVSVYNKRSM-----KRKEMIGWFSLGYSNSSEEELNHWNDMRES--RAEQVCRWHMLLEP
HrobustaSyt16               GSNFKNMAAK--RPPDTYVKLALMAPNGQEISRSKTSIRRGQPN-PLYKETFMFQVPQFQLPDVSLMVSVFVQKSL-----KRKEMIGWFTMGNTNSSEDDASHWTAMLES--KGEQVCRWHVLLEP
LgiganteaSyt16              GSNFRNMAMN--RAPDTYVKLTLMSPSGQELQRCKTSVRRGQPN-PLFKETFMFQVAQFQLPEVTLMVSVYNKKNM-----KKKEMIGWFSLGMNSSGEEEQGHWSDMRESK-GGDQVCRWHVLLES
CsavignyiSyt16var1          GSHFRNLALP--KPPDTFVKLVLLNSSCQEMAQSKTTVRRSQPN-PVFKETFYFQIALFQLSEVSLMVSVYCSKTI-----KKKEMLGWISLGQNSSSEADEQHWVEMKEA--KGSTVSRWHTLIES
CsavignyiSyt16var2          GSHFRNLALP--KPPDTFVKLVLLNSSCQEMAQSKTTVRRSQPN-PVFKETFYFQIALFQLSEVSLMVSVYCSKTI-----KKKEMLGWISLGQNSSSEADEQHWVEMKEA--KGSTVSRWHTLIES
CintestinalisSyt16var1      GSHFRNLAMS--KPPDTFVKLVLLNSSCQEMAQSKTTIRRSQPN-PVFKEPFYFQVALFQLVEVSLMVSVYSSKTI-----KKKEMLGWISLGQNSSSEADEQHWLEMKEA--KGNTVSRWHTLIES
CintestinalisSyt16var2      GSHFRNLAMS--KPPDTFVKLVLLNSSCQEMAQSKTTIRRSQPN-PVFKEPFYFQVALFQLVEVSLMVSVYSSKTI-----KKKEMLGWISLGQNSSSEADEQHWLEMKEA--KGNTVSRWHTLIES
SpurpuratusSyt16var1        GSHFKQRAAG--RAPDSYAKVALMSSSGFEISKSKTSVRRGQSS-PTFKETFFFQVAQFQLPEMTLLFTVFNKKGM-----KRKETIGWFSMGLSNSSEEEIGHWNEMRES--KGHQVCRWHALLES
SpurpuratusSyt16var2        GSHFKQRAAG--RAPDSYAKVALMSSSGFEISKSKTSVRRGQSS-PTFKETFFFQVAQFQLPEMTLLFTVFNKKGM-----KRKETIGWFSMGLSNSSEEEIGHWNEMRES--KGHQVCRWHALLES
SpurpuratusSyt16var3        GSHFKQRAAG--RAPDSYAKVALMSSSGFEISKSKTSVRRGQSS-PTFKETFFFQVAQFQLPEMTLLFTVFNKKGM-----KRKETIGWFSMGLSNSSEEEIGHWNEMRES--KGHQVCRWHALLES
BfloridaeSyt16              GSHFRNMAMN--RAPDTYVKICLMASNGQELSRSKTSIRRGQPN-PVYKETFIFQVALFQLSDVTLMFSVYNKRGM-----KRKEMIGWFSLGLNNSGEEELNHWQDMRES--KGQQVCRWHVLLES
IscapularisSyt16            GSHFRNVAMN--RAPDTFVKLTLMSSSGQEIARSKTSVRRGQPN-PLFKETFVFQVALFQLPDVTLMVSVYNKRSM-----KRKEMIGWFSLGLNSSGEEELSHWSDMRESK-G-EQVCRWHVLLET
DpulexSyt16                 AASLRSWTSN--RSPDACVKVLLLTQTGQELGRAKTAPKRNSDGGPTWMETFAFQVTLFQLAEVTLLISVVNKRSL-----KRRELLGWISLGFDSSVDQQTEHWQEMREAT--GEQICHWHALLPP
TcastaneumSyt16             GSHFRNLALN--RAPDTYVKLNLVSSTGQELAHSKTTVRRGQPN-PLFKETFVFQVALFQLADVTLMVSVYNRKGVT----KKKEMVGWFSLGLNSSGAEELAHWMDMKESQ-Q-QQICRWHILVQS
NvitripennisSyt16           GSHFRGGCGNDTKPPDTYVKLALVDSNGHEMGHSKTGLRRAQPN-PLYKETFIFQVALFQLGDVTLYLSVYNRRRTAVMGKKGREMIGWLCLGLSSSGADELQHWNDMRSAR-QPTQVQRWHSLLRP
AmelliferaSyt16             GSHFRGGGGNDTKPPDTYVKLSLVDSNGHEMERSKTGLKRAQPN-PLYKETFIFQVALFQLGDVTLFLSVYNRRRGG-MGRKGREMIGWLSLGLNSSGSEELQHWNDMRAAR-QPTQVQRWHSLLRP
AgambiaeSyt16               GSHFRNHTLP--KVPDTYVKLCLVSSMGQEIARAKTSTRRGQSN-PLFKETFIFQVAMFQLNDVTLIVSVYAKRNM-----KRNEMVGWFSMGLNSSGPEEMIHWNEMRESSSRSELITRWHVLVDS
DmelanogasterSyt16          GSQFRSLSLN--KAPDTYVKMVMVSSIGQEISRAKTSIRRGQPN-PLFKETFAFQVALFQLNDVTLMISVYAKRHM-----KKNEMVGWFSLGLNSSGSEEVAHWADVCEMP-KGEMLARWHVLVDS
DsimulansSyt16              GSQFRSLTLN--KAPDTYVKMVMVSSIGQEISRAKTSIRRGQPN-PLFKETFAFQVALFQLNDVTLMISVYAKRHM-----KKNEMVGWFSLGLNSSGSEEVAHWADVCEMP-KGEMLARWHVLVDS
DsechelliaSyt16             GSQFRSLTLN--KAPDTYVKMVMVSSIGQEISRAKTSIRRGQPN-PLFKETFAFQVALFQLNDVTLMISVYAKRHM-----KKNEMVGWFSLGLNSSGSEEVAHWADVCEMP-KGEMLARWHVLVDS
DerectaSyt16                GSQFRSLSLN--KAPDTYVKMVMVSSIGQEISRAKTSIRRGQPN-PLFKETFAFQVALFQLNDVTLMISVYAKRHM-----KKNEMVGWFSLGLNSSGSEEVAHWADVCEMP-KGEMLARWHVLVDS
DyakubaSyt16                GSHFRNLSLN--KAPDTYVKMVMVSSIGQEISRAKTSIRRGQPN-PLFKETFAFQVALFQLNDVTLMISVYAKRHM-----RKNEMVGWFSLGLNSSGSEEVAHWADVCEMP-KGEMLARWHVLVDS
DananassaeSyt16             GSQFRSLSLN--KAPDTYVKMVMVSSIGQEISRAKTSIRRGQPN-PLFKETFAFQVALFQLNDVTLMISVYAKRHM-----KKNEMVGWFSMGLNSSGPEEVAHWTDVCEMP-KGEMLARWHVLVDS
DpseudoobscuraSyt16         GSQFRSLSLN--KAPDTYVKMVMVSSIGQEISRAKTSIRRGQPN-PLFKETFAFQVALFQLNDVTLMISVYAKRHM-----KKNEMVGWFSLGLNSSGPEEVAHWADVCEMP-KGEMLARWHVLVDS
DpersimilisSyt16            GSQFRSLSLN--KAPDTYVKMVMVSSIGQEISRAKTSIRRGQPN-PLFKETFAFQVALFQLNDVTLMISVYAKRHM-----KKNEMVGWFSLGLNSSGPEEVAHWADVCEMP-KGEMLARWHVLVDS
DwillistoniSyt16            GSQFRSLSLN--KAPDTYVKLCMVSSIGQEISRAKTSIRRGQPN-PLFKETFAFQVALFQLNDVTLMISVYAKRHM-----KKNEMVGWFSLGLNSSGPEEVAHWADVCEMP-KGEMLARWHVLVDS
DvirilisSyt16               GSQFRSLMLN--KAPDTYVKLCMVSSIGQEISRAKTSIRRG-PN-PLFKETFAFQVALFQLNDVTLMISVYAKRHM-----KKNEMVGWFSLGLNSSGPEECAHWADVCEMP-KGEMLARWHVLVDS
DmojavensisSyt16            GSQFRSLTLN--KAPDTYVKLCMVSSIGQEISRAKTSIRRG-PN-PLFKETFAFQVALFQLNDVTLMISVYAKRHM-----KKNEMVGWFSLGLNSSGPEECAHWADVCEMP-KGEILARWHVLVDS
DgrimshawiSyt16             GSQFRSLTLN--KAPDTYVKLCMVSSIGQEISRAKTSIRRG-PN-PLFKETFAFQVALFQLNDVTLMISVYAKRHM-----KKNEMVGWFSLGLNSSGPEECAHWADVCEMP-KGEILARWHVLVDS
```
