# Supplementary material for: A manual collection of Syt, Esyt, Rph3a, Rph3al, Doc2, and Dblc2 genes from 46 metazoan genomes - an open access resource for neuroscience and evolutionary biology
Source: BMC Genomics. 2010 Jan 15;11:37. doi: 10.1186/1471-2164-11-37 (PMC2823689; doi:10.1186/1471-2164-11-37)
Supplement: Additional file 31 — Alignment of the vertebrate Syt16 sequences which have TM domains. Amino acid position is marked every hundred amino acids approximately, at the top of each page of the alignment. Splice variants are included and highlighted with black dots where they differ. Intron position and phase is indicated with a coloured bar between amino acids. Black bars indicate phase 0 introns. Red bars indicate phase +1 introns. X residues indicate where a portion of sequence is missing. [file 1471-2164-11-37-S31.PDF]

```
                                                                                                                   100
                                          |         |         |         |         |         |         |         |         |         |
Trubripessyt16             MASDITPEAIGFLSTVGLVIVLLTIFLLFINKKLCFSRVGGLPCLEQNGHRKK--RP--GMRQGLVNSFGNEEDNGT--TSSDSDGEILKQFEIS--RSQTFR----AQASVRRER
Tnigroviridissyt16         ----XTPEAIGFLTGVGLVAVLLAIFLLFINKKLCFSRVGGLSCLEQRGSRKK--HP--GPATSL------EEDGGT--TSSDSDGEILKQFEISASRSHTLR----AQTPERHER
Gaculeatussyt16            ----XPPEAIGFLSAVGVFIVALAVLFLFINKKLCFSRVGGLPCLEHRHAHRK--KTR----QGLLSSYGDEDEDGGGVSSSGSEEEVLKQFEISVSRSQSFRTSTAANLIELQAQ
Olatipessyt16              ----XTPEAIGFLSAVGVFIVALAILFLFINKKLCFSRVGGLPCLEQHGRHKR--RRQ-GVLQGLVSSYGYDGDAAS--SSSDSEDEVLKQFEISASRPQSFRVAPASGGPETSRQ
Dreriosyt16                MASDITPEAVGFLSAVGIFVVLLAILFLFINKKLCFARVGGLPCLEQYSRRKR--RDRAGIHQGLVNSYGDDGEIS---SSSDSDDELAKHFEISVSRSQSFR----SGVTEINPQ
Xtropicalissyt16var1       MATDITPEAIWFLSAVGVLIVLLAVLFLIINKKLCFENIGDLPCLDQRGKKKQLPKKKSGVLQSLVNSYGEDEDLT---SSSDSDEEVFKQFQISVSRSQSFRSTVSEKATQAGSE
Xtropicalissyt16var4       MATDITPEAIWFLSAVGVLIVLLAVLFLIINKKLCFENIGDLPCLDQRGKKKQLPKKKSGVLQSLVNSYGEDEDLT---SSSDSDEEVFKQFQISVSRSQSFRSTVSEKATQAGSE
Acarolinensissyt16var1     ----XTPEAIGFLSAVGVFIVLLGVLFLFINKKLCFEKIGGLPCLEQPGKRRRD-KEKSGVREGLVNSYGEDEDLT---TSSDSDDEVIKQFEISVSRSQSFRSGVSEKGTQAGLE
GgallusSYT16var1           -----XYFCGRWVFACGIFVILLPVFLLSINKKLYFEKRGGLSCLEREGRRKHY-KEKSRIHDGLX--------------------------------------------------
TguttataSYT16var1          ----XSPDTAGFLSAAGPFIILPAVLFHFINKKREVDS----KCLEQ-GEENHC-EEKSRAHEGLVNSYKEEDEQA---ASSGNNHDMVRQFETSMSWSHSFDQKYLNK---VGVG
MdomesticaSyt16var1        ----XTSEAIGFLSAVGVFIVLLAILFLFINKKLCFEKIGGLPCLEHRGKRKHS-RGKSGVHEGLVNSYGEDE-LS---TSSDSDEEIIKQFEISVSRSQSFRSGPYEKGTQASSE
MmusculusSyt16var1         MATDITPEAIGFLSAIGVFVVLLAVLLLFINKKLCSENLRGHPYPEQRGKRKHS-RDKTGGHTGTVNSFGDDEEPS---TSSESDEDVTKQFKISVSRSQSFRSGVSEKGKTTELE
MmusculusSyt16var4         MATDITPEAIGFLSAIGVFVVLLAVLLLFINKKLCSENLRGHPYPEQRGKRKHS-RDKTGGHTGTVNSFGDDEEPS---TSSESDEDVTKQFKISVSRSQSFRSGVSEKGKTTELE

                                                                                                         200
                                          |         |         |         |         |         |         |         |         |         |
Trubripessyt16             P-----RSVQTCSIDQEQEPVGSELSDRE-----GGDRRSNHVHL---FLLSEMDAPSQH------SGQDSLSAATAQREATPTPSLDRSVSLQLST-TAESPPPSVLQQAPESIK
Tnigroviridissyt16         P-----RLDLRPSVKREQETVGPELSDREDAHKHAADGRKLHRLLSNSFIFAEMEAPHRP------STQDSLSAAPVQGEGTSTPSFHRSASLLLPPGTPESSSLSDVQPAPESRE
Gaculeatussyt16            T--ALGRRHKFSRL-SDQEEGSTEPSDCEEMAAQGHQGG----------------------------FKDPLTAALEESEWAAPDALE--AAG---A--SDGPAQGANRQAADGSE
Olatipessyt16              THLPLSRRNKFTRL-SEQEEGSTEPSDCEERFKQLPDGSNFLPRLLCLLPAEAEASGRQ-------TFQDPLCAVEDKSEKMSAPLLNSPAAT---A--DASPILSPTRPAAEGSE
Dreriosyt16                N--APGQHHKFNRLLSDQEEGSTEPSDCEEAERKSVQSFQDPLTSRRRVGSEPRDPLSQSPLPEGRTPTDPLSLMAEQISVGSQDTGDGLEAL---PRDDGSRLGSVGRQTPQRSL
Xtropicalissyt16var1       ------SRQKFSRLLSNHEELSTEASECED-------------------LDVLCYQSN----------EEDDHVSYRSRQELAA-----------------TEHKNATGQEADARP
Xtropicalissyt16var4       ------SRQKFSRLLSNHEELSTEASECED-------------------LDVLCYQSN----------EEDDHVSYRSRQELAA-----------------TEHKNATGQEADARP
Acarolinensissyt16var1     ------RRPKFNRLLSNHEEYSSEISECEGILIRNVLRTGKREMVYLDGFSQLSYQDNL-------SYHEDDRLSFDSRTASESRGSGQTKDPEMEVGAKPSLRRQETEGSLEMET
GgallusSYT16var1           ---------------------------------------------XLDGLSQLSYQDNL-------SCHEDDHVSVDSRTT-ERRSAGQHTGPRMESSIADGFSQQATEGRLELEA
TguttataSYT16var1          ------CSPKFSHFPCSHKEHRTEVSRCRD----------------LDGLSQLSYQDNL-------SCREDDHVSVDSRTTTRSRGTGQHKGPRVEPRIGDGVSQSHREGCLEMET
MdomesticaSyt16var1        ------RKPKLKHLLSSHEENSTEASECED----------------LDGLSQPRCLNNV-------SYGEDDHNSVDSRITSESRDFGEQRTNRREAIFTHSFGEEATGRNLETET
MmusculusSyt16var1         ------QKIKCKRLLCTHQEDSAEGSACE----------------DLDRTSQLSYSEIL-------SY-EDRPISILPQSPFESRNVRHHGPCRPEMGMVRSLGRPCADGVLETET
MmusculusSyt16var4       ● ------QKIKCKRLLCTHQEDSAEGSACEGIHC-SALHCRQGQVNDLDRTSQLSYSEIL-------SY-EDRPISILPQSPFESRNVRHHGPCRPEMGMVRSLGRPCADGVLETET

                                                                                              300
                                          |         |         |         |         |         |         |         |         |         |
Trubripessyt16             GAEAVDLDKNNDTDSLSTLSPENPPPDEVSSLQDVASS--AADISKCGDLLLSLEYRPNAEKLVVTTIAAQDVPDKARSGMDTWQVRMVLLPYKKQRKKTSVQKGSLPHFNETFHF
Tnigroviridissyt16         GAEVADEDGNNDTDSASTWSPENPPQDERSPQQDAACA--PAEIPKCGDLLLSLEYRPNAEKLVVTTIAAEDVPDKARSGMDTWQVHLVLLPYKKQRKKTSMQRGSLPHFNETFHF
Gaculeatussyt16            EME-NG-GGSAAVECSPVWSPEPDQPQPASSSPRL-------PISTCGDLVLSLEYRPDAEKLLVSVIAARDVPDKARSGMDSWQVHMVLLPAKKQRHRTLVQKGSLPHFNETFRF
Olatipessyt16              DKPVDQ-DKNEAADSSSTWSPEQERTLHSSASSQLLCSSSRPPICKCGDLVLSLEYRPSLRKLLVSVLSARGVPDKDRSGLDSWQVHMLLLPTKKQRHKTSVQKGPCPHFNETLRF
Dreriosyt16                AMDNQGYDNSDVTESFPTWSPEHDQSLSHPLSSSP-----RGHISVCGDLIIALDYRAETHRLLVTVIMAQGIPDKARSGMDSWQVHVVLLPGKRQRHKTAVQKGSMPMFEETFRF
Xtropicalissyt16var1       GVNNDGFEDQDATDNSSVCSPEDQAESTITPPVPYV------PISKCGDLDVTLEYKPSSQKLTVTVLEAKDIPDKERSGVDSWQVHLVVLPSKKQRGKTTTQKGSLPVFNETFTF
Xtropicalissyt16var4       GVNNDGFEDQDATDNSSVCSPEDQAESTITPPVPYV------PISKCGDLDVTLEYKPSSQKLTVTVLEAKDIPDKERSGVDSWQVHLVVLPSKKQRGKTTTQKGSLPVFNETFTF
Acarolinensissyt16var1     AFSNQGFEMNDATDSSSAWTPEEHEGANVLPAHPSP----REPICKCGDLDIIFDYRPSTQKLLVTVLEAKDIPDKDRSGVSTWQVHVVLMPSKKQRGKTSVQRGPSPAFKDKVTF
GgallusSYT16var1           AFTNQGFEGNDATDSSSAWSPEETDEVNSEPAHHSA----HQPICKCGDLDVIFSYKASSQKLVVTVLEARDIPDKDRSGVNTWQVHTVLMPSKKQRGKTRVQRGPIPMFKDNITF
TguttataSYT16var1          AFVNQGLEVNDATDSSSAWSPEEHNEVNSAPAHHCA----HEPICKCGDLDVIFTYKPSSQKLIATVLAARDIPDKDRSGVNTWQVHAVLMPGKKQRGKTSVQRGPIPIFQDKITF
MdomesticaSyt16var1        SFSNPGFEEPYSTDTSSAWSPEEQDGLKVPHFPSSA----PEPISKCGDLDVIFEYNSSSQKLAVTIVKAQNVPDKDRSGVNAWQVHTVLLPRKKQRGKTSVQRGPTPVFKEKVFF
MmusculusSyt16var1         AFVSRGFEDSYATHSSSLWSPEEQDGTSL-QVPHRL----LEPISKCGDLDVIFEYRAVTQKLTVTIVRAQGLPDKDRSGVNSWQVHIVLLPSKKQRGKTNIQRGPNPVFKEKVTF
MmusculusSyt16var4         AFVSRGFEDSYATHSSSLWSPEEQDGTSL-QVPHRL----LEPISKCGDLDVIFEYRAVTQKLTVTIVRAQGLPDKDRSGVNSWQVHIVLLPSKKQRGKTNIQRGPNPVFKEKVTF
```

```
                                                                      400
Trubripessyt16             SGLHPSELHKMAIRFRLYALGG-RVLRDRMIGEKVLRLDELQPEGGTTEMTLSLEPRSNLKTLSAQQS----PFPDGALSSQSLAHGGVPELLLGLSYSATTGRLSVEIIKGSHFR
Tnigroviridissyt16         SGLHPSELHKMAIRFRLYALGG-RVLRDRMIGEKVLRLDQLPPEGGTTEMTLSLEPRSNLKTLNAPPSSSHVPLSSSAASIQSLAHGGVPELLLGLSYNATTGRLSVEVIKGSHLR
Gaculeatussyt16            SRLEPAELQASAVRFRLYALGASRMSRERMMGEKVLRLGGLDPEGGTMETTLVLEPRSNLKSLDSQLSLSAVSQSDSASSTQSLTHGGVPELLVGLTYNATTGRMSVELIKGSHFR
Olatipessyt16              SHVEPTDLHTSAVRFRLYALGG-RMFRERMVGEKVLCLGGLDPQGGTTETTVVLEPRSNLKHVGSQLSLSAASQSDIASFTQSLTHGGVPELLLGLSYNATTGRMSVELIRGSLFR
Dreriosyt16                SHLEPGDLPSSALRFRLYALG--KMNRERMMGETLYRLSRLK-HTGRFETTLVLEPRSNLKTVDAQTSLS--TQTDSASSSLSVSHAGNPELLLGLSYNATTGRLSVEIIKGSHFR
Xtropicalissyt16var1       NKLVPEEMPNHALRFRLYAVH--KMNRVKMMGEKLYHLRNIRPD-SENNLTLVLEPRSNLTSGDSQLSFTPVSHSESDSSTQSLSHGGVPELLVGLSYNPTTGRLSVEMIKGSHFR
Xtropicalissyt16var4       NKLVPEEMPNHALRFRLYAVH--KMNRVKMMGEKLYHLRNIRPD-SENNLTLVLEPRSNLTSGDSQLSFTPVSHSESDSSTQSLSHGGVPELLVGLSYNPTTGRLSVEMIKAYRLH
Acarolinensissyt16var1     TKLSPEELSHYAIRFRLYSVR--KMIKERMMGEQLFYLSNLNQE-GEMKVTLVLEPRSNLCSGGSQISLSAISHSDSASSNQSLSHGGVPELLVGLSYNATTGRLSVEMIKGSHFR
GgallusSYT16var1           SKLEPHELGGHAVRFRLYAVH--KVIGEKMMGEQLFYLSNISQE-EEVKVTLVLEPRSNLTSADSQLSLSAISHSDSASSTQSLSHGGVPELLVGLSYNATTGRLSVEMIKGSHFR
TguttataSYT16var1          SKLEPQELSSHAVRFRLYAVH--KVVGEKMMGEQLFYLRGISQE-GEVKVTLLLEPRSNLSSADSQLSLSAISHSDSASSTQSLSHGGVPELLVGLSYNATTGRLSVEMIKGSHFR
MdomesticaSyt16var1        SKLQPEDLGRHAVQFRLYAAL--KMNREKMMGEKLFHLSQLHQG-GKMKVTLVLEPRSNMSSGESQLSLSAVSHSDSASSTQSLSHGGVPELLVGLSYNATTGRLSVEIIKGSHFR
MmusculusSyt16var1         TKLEPRDVASCAVRFRLYAAR--KMTRERMMGEKLFCLSHLHPE-GEMKVTLVLEPRSNLSSGESPLSPSVVSHSDSASSTQSLSHGGVPELLVGLSYNATTGRLSVEMIKGSHFR
MmusculusSyt16var4         TKLEPRDVASCAVRFRLYAAR--KMTRERMMGEKLFCLSHLHPE-GEMKVTLVLEPRSNLSSGESPLSPSVVSHSDSASSTQSLSHGGVPELLVGLSYNATTGRLSVEMIKGSHFR

                                                              500
Trubripessyt16             NLAITKPPDTYARLS--LLNSVGQETSRCKTSVRRGQPNPVYKETFVFQVALFQLSDVTLLVSIYNRRSMKRKEMVGWVSLGQNSSGEEERLHWEDMKEGRGQQVGRWHVLLEA
Tnigroviridissyt16         NLAATRPPDTYVRLS--LLNSVGQETSRCKTSVRRGQPNPVYKETFVFQAALFQLSDVTLLVSVYNRRSMKRKEMVGWIALGQSSSGEEERLHWQDMREAGGPQVSRWHVLLEA
Gaculeatussyt16            NLAVNRPPDTYGRLT--LLNSVGQEISRCKTSVRRGQPNPVYKETFVFQVALFQLSDVTLLVSVYNRRSLKRKETVGWIAMGQNSSGEEEQLHWQDMKDSRGQQVCRWHVLLEA
Olatipessyt16              NLAVSRPPDTYGRLT--LLNSVGQEIARCKTSVRRGQPNPVYKETFVFQVALFQLSDVTLLVSINTRRSMKRKEMVGWIALGQNSSGKEEQLHWQVMKESRGQQVCRWHVLLEA
Dreriosyt16                NFAVNRPPDTFGKLT--LLNSMGQEISRCKTSIRRSQPNPVFKETFVFQVALFQLSDVTLMVSIYNRRNMKRKEMIGWVALGQNSSGEEELLHWQDMKESGNQQVCRWHTLLDA
Xtropicalissyt16var1       NLAINRPPDTYGKLS--LLDSVGHEISRCKTSVRRGQPNPVYKETFIFQVALFQLTDFTLMISIYNRRSMKRKEMIGWISLGKNSSGEEEQNHWLEMKESKGQQVCHWHTLLES
Xtropicalissyt16var4     ● TNDFHLQPAQHEKEGDDWMDFPGEKQ----------------------------------------------------------------------------------------
Acarolinensissyt16var1     NLAVNRPPDTYGKLC--LLNSIGQEMSRCKTSVRRGQPNPVYKETFVFQVALFQLSDVTLMISIYNRRSIKRKEMIGWLSMGQNSSGEEEQNHWHEMKESKSQQICRWHTLLES
GgallusSYT16var1           NLAINRPPDTYGKLC--LLNSVGQEMSRCKTSIRRGQPNPVYKETFIFQVALFQLSDVTLMISIYNKRSMKRKEMIGWISMGQNSSGEEEQSHWQEMKESKGLQVCRWHTLLES
TguttataSYT16var1          NLAINRPPDTYGKLC--LRNSVGQEMSRCKTSIRRGQPNPVYKETFIFQVALFQLSDVTLLISIYNRRSMKRREMVGWISMGQSSSGEEEQSHWQEMRESQGTQVCRWHTLLES
MdomesticaSyt16var1        NLAINRPPDSYGKLF--LLNSVGQEMSRCKTSIRRGQPNPVYKETFVFQVALFQLSDVTLMISIYNRRAMKRKEMIGWISLGQNSSGEEEQDHWQEMKETKGQQICRWHTLLES
MmusculusSyt16var1         NLAANRAPDTYGKLF--LLNCVGQEMSRCKTSIRRGQPNPVYKETFVFQVALFQLSDVTLMISIYSRRTMKRKEMIGWVALGQNSSGEEEQEHWEEMKESKGQQTCRWHTLLES
MmusculusSyt16var4         NLAANRAPDTYGKLF--LLNCVGQEMSRCKTSIRRGQPNPVYKETFVFQVALFQLSDVTLMISIYSRRTMKRKEMIGWVALGQNSSGEEEQEHWEEMKESKGQQTCRWHTLLES
```
