# Supplementary material for: A manual collection of Syt, Esyt, Rph3a, Rph3al, Doc2, and Dblc2 genes from 46 metazoan genomes - an open access resource for neuroscience and evolutionary biology
Source: BMC Genomics. 2010 Jan 15;11:37. doi: 10.1186/1471-2164-11-37 (PMC2823689; doi:10.1186/1471-2164-11-37)
Supplement: Additional file 32 — Alignment of the vertebrate Syt16 sequences which lack TM domains. Amino acid position is marked every hundred amino acids approximately, at the top of each page of the alignment. Splice variants are included and highlighted with black dots where they differ. Intron position and phase is indicated with a coloured bar between amino acids. Black bars indicate phase 0 introns. Red bars indicate phase +1 introns. X residues indicate where a portion of sequence is missing. [file 1471-2164-11-37-S32.PDF]

```
                                                                                                                 100
Xtropicalissyt16var2      MVQPMASQ---RFFQPVSSWISRMCEAVSLAGDSISGSLNNLNK--AQPSVDSKQDSQEYQEEFEYHIEDFPEENPCSSDNDDFENVNNVVTSEYNEANGQAL--VTSDNTESPGCVGSS
Xtropicalissyt16var3      ------------------------------------------------------------------------------------------------------------------------
Acarolinensissyt16var2    ----MASQDVVNFFQPFSSWVSQFFEALQEAGDTISSSFTSLGN--RAIEEDGDKDLENSDGSYEHHSEGSDNESTFSQDSEESDLFPSSCHDELESSSASSL--GPYWYEGKRIHPQEE
GgallusSYT16var2          ----MTSEAAQSFLQPLSSWLSQIYEAIQQAGDSISASLLNLNGVGRESEEDRNRGLEGNGVAYEDYSVESEDERHIDLWDEEYICPREDGHIAGRDLASEALPCGSDMGMQRQKWVKNT
TguttataSYT16var2         ----MASEAPQNFLQPLSSWMSQIYEAIQQAGDSISASLLNLSGVGRKPEGERTQDLENNGGVYEDYSEESGDERDLDLWDEDYFYPREDSYIGGPGLASQNLPHSSDTGPQRQNRVKTT
MdomesticaSyt16var2       MVLTMASQDTQNFFQSLSSWISRVYEAVQQVGDTLSASIVNLSK--HNPQFNGELDPELIDGDVQE----SDLQDGEKQNHAQIKDDMEPLLLEANQFPTGTY--NTDLGDMKQSP----
MmusculusSyt16var2        MVLTMASQDVQNFFQPLSSWLSRVYEALQQAGDALSASLVLLSK--HDSALSDKPEQDLDAAQIQQ----TYLED-EEQDHDGSPEEASSLFLEEDHFSLSNS--DLQDSVQTASPTLGQ
MmusculusSyt16var3        ------------------------------------------------------------------------------------------------------------------------
HsapiensSYT16var1         MVLAMASQDVQNFFQPFSSWISRVYEALQQAGDMLSASLVNISK--QDSKLSDKLDQDLDNIQIQE----TYFED-EEQDNDWSQEDANSLFLEVDHFSCCNS--DLQDSAQNSSPSLSQ
HsapiensSYT16var2a        MVLAMASQDVQNFFQPFSSWISRVYEALQQAGDMLSASLVNISK--QDSKLSDKLDQDLDNIQIQE----TYFED-EEQDNDWSQEDANSLFLEVDHFSCCNS--DLQDSAQNSSPSLSQ
HsapiensSYT16var2b        ------------------------------------------------------------------------------------------------------------------------
HsapiensSYT16var3         ------------------------------------------------------------------------------------------------------------------------

                                                                                   200
Xtropicalissyt16var2      LCHAETSIVSTSGAIVDEDEDTIETLSTTTEEEDIDNQIRTLLRDLDSPTFSSVGITNGKEFWEGKICVVGVLKPLHFFCSLTSDVQMNSYGEDEDLTSSSDSDEEVFKQFQISVSRSQS
Xtropicalissyt16var3      ------------------------------------------------------------------------------------------------------------------------
Acarolinensissyt16var2    YLDDDDSLASYGACQSYKGYQVMETLPTIPEEEEHLL--------------------------------------------------LNSYGEDEDLTTSSDSDDEVIKQFEISVSRSQS
GgallusSYT16var2          TSLAGQCVERTGHLVANGSHWINEDLQPL---PTISEDESEPSVRMRGHDHSISMDGSLE------------------INKDKASFAX--------------------------------
TguttataSYT16var2         STLPEQCVESSRTLTSNGSYWMSEDLQPLDRLPPIAEEEGEPSWRMGGHEHSLSLGGSLN------------------SANGTARCAVNSYKEEDEQAASSGNNHDMVRQFETSMSWSHS
MdomesticaSyt16var2       -LGDSSSIVSQLSYPTCDNPEQPSRLLPVLEEDCHL----EKLKKNLQHDFENQVPESLE--------------------S-NG-EQVNSYGEDE-LSTSSDSDEEIIKQFEISVSRSQS
MmusculusSyt16var2        QAEDSSSVIPPWPSKIPGAPKPQPVLSSIAEEDHHS----ERQRCGRQHG-----SGTLE--------------------KVNGRKQVNSFGDDEEPSTSSESDEDVTKQFKISVSRSQS
MmusculusSyt16var3      ● -----------------------------------------------------------------------MTSKEGGGEGEEIIHNVNSFGDDEEPSTSSESDEDVTKQFKISVSRSQS
HsapiensSYT16var1         HAKDSCSTMSQWPNWASDDRKLPHVLSSIAEEEHHL----EKQRSGLQHGFDSQLPGTLE--------------------TVNGKKQVNSFGDDEELSTSSDSDEEVIKQFEISVSRSQS
HsapiensSYT16var2a        HAKDSCSTMSQWPNWASDDRKLPHVLSSIAEEEHHL----EKQRSGLQHGFDSQLPGTLE--------------------TVNGKKQVNSFGDDEELSTSSDSDEEVIKQFEISVSRSQS
HsapiensSYT16var2b        ------------------------------------------------------------------------------------------------------------------------
HsapiensSYT16var3         ------------------------------------------------------------------------------------------------------------------------

                                                              300
Xtropicalissyt16var2      FRSTVSEKATQAGSESRQKFSRLLSNHEELSTEASECED-------------------LDVLCYQSN---EEDDHVSYRSR--QELAATEHKNATGQEADARPG---------------
Xtropicalissyt16var3      -----------------------------------------------------------------------------------------------------------------------
Acarolinensissyt16var2    FRSGVSEKGTQAGLERRPKFNRLLSNHEEYSSEISECEGILIRNVLRTGKREMVYLDGFSQLSYQDNLSYHEDDRLSFDSRTASESRGSGQTKDPEMEVGAKPSLRRQETEGSLEMETA
GgallusSYT16var2          -------------------------------------------------------LDGLSQLSYQDNLSCHEDDHVSVDSRTTER-RSAGQHTGPRMESSIADGFSQQATEGRLELEAA
TguttataSYT16var2         FDQKYLNK---VGVGCSPKFSHFPCSHKEHRTEVSRCRD----------------LDGLSQLSYQDNLSCREDDHVSVDSRTTTRSRGTGQHKGPRVEPRIGDGVSQSHREGCLEMETA
MdomesticaSyt16var2       FRSGPYEKGTQASSERKPKLKHLLSSHEENSTEASECED----------------LDGLSQPRCLNNVSYGEDDHNSVDSRITSESRDFGEQRTNRREAIFTHSFGEEATGRNLETETS
MmusculusSyt16var2        FRSGVSEKGKTTELEQKIKCKRLLCTHQEDSAEGSACED----------------LDRTSQLSYSEILSY-EDRPISILPQSPFESRNVRHHGPCRPEMGMVRSLGRPCADGVLETETA
MmusculusSyt16var3        FRSGVSEKGKTTELEQKIKCKRLLCTHQEDSAEGSACED----------------LDRTSQLSYSEILSY-EDRPISILPQSPFESRNVRHHGPCRPEMGMVRSLGRPCADGVLETETA
HsapiensSYT16var1         FRSVTSEKGKQTGLEQKPKFSRSLLTHGEDGTEVSACED----------------LDGASQRRYSENLSYGEDDHIPAHSQSPCERGDAKHHGTSHQESSVVQSLRRQSTEGSLEMETA
HsapiensSYT16var2a        FRSVTSEKGKQTGLEQKPKFSRSLLTHGEDGTEVSACED----------------LDGASQRRYSENLSYGEDDHIPAHSQSPCERGDAKHHGTSHQESSVVQSLRRQSTEGSLEMETA
HsapiensSYT16var2b        -----------------------------------------------------------------------------------------------------------------------
HsapiensSYT16var3       ● -------------------------------------------------------------------------------------------------------------------META

                                                   400
Xtropicalissyt16var2      VNNDGFEDQDATDNSSVCSPEDQAESTI--TPPVPYVPISKCGDLDVTLEYKPSSQKLTVTVLEAKDIPDKERSGVDSWQVHLVVLPSKKQRGKTTTQKGSLPVFNETFTFNK
Xtropicalissyt16var3      -----------------------------------------------------------------------------------------------------------------
Acarolinensissyt16var2    FSNQGFEMNDATDSSSAWTPEEHEGANVLPAHPSPREPICKCGDLDIIFDYRPSTQKLLVTVLEAKDIPDKDRSGVSTWQVHVVLMPSKKQRGKTSVQRGPSPAFKDKVTFTK
GgallusSYT16var2          FTNQGFEGNDATDSSSAWSPEETDEVNSEPAHHSAHQPICKCGDLDVIFSYKASSQKLVVTVLEARDIPDKDRSGVNTWQVHTVLMPSKKQRGKTRVQRGPIPMFKDNITFSK
TguttataSYT16var2         FVNQGLEVNDATDSSSAWSPEEHNEVNSAPAHHCAHEPICKCGDLDVIFTYKPSSQKLIATVLAARDIPDKDRSGVNTWQVHAVLMPGKKQRGKTSVQRGPIPIFQDKITFSK
MdomesticaSyt16var2       FSNPGFEEPYSTDTSSAWSPEEQDGLKVPHFPSSAPEPISKCGDLDVIFEYNSSSQKLAVTIVKAQNVPDKDRSGVNAWQVHTVLLPRKKQRGKTSVQRGPTPVFKEKVFFSK
MmusculusSyt16var2        FVSRGFEDSYATHSSSLWSPEEQDGTSL-QVPHRLLEPISKCGDLDVIFEYRAVTQKLTVTIVRAQGLPDKDRSGVNSWQVHIVLLPSKKQRGKTNIQRGPNPVFKEKVTFTK
MmusculusSyt16var3        FVSRGFEDSYATHSSSLWSPEEQDGTSL-QVPHRLLEPISKCGDLDVIFEYRAVTQKLTVTIVRAQGLPDKDRSGVNSWQVHIVLLPSKKQRGKTNIQRGPNPVFKEKVTFTK
HsapiensSYT16var1         FNSRGFEDSYATDSSSMWSPEEQDRTNL-QVPSGVSEPISKCGDLDVIFEYRAASQKLTVTIVRAQGLPDKDRSGVNSWQVHVVLLPGKKHRGRTNIQRGPNPVFREKVTFAK
HsapiensSYT16var2a      ● FNSRGFEDSYATDSSSMWSPEANLNLK-----RKGYLLDVYCLSSPLLFTTSLHRRNRTGPICRCHPGSQSPSQSVVT-----------------------------------
HsapiensSYT16var2b        -----------------------------------------------------------------------------------------------------------------
HsapiensSYT16var3         FNSRGFEDSYATDSSSMWSPEEQDRTNL-QVPSGVSEPISKCGDLDVIFEYRAASQKLTVTIVRAQGLPDKDRSGVNSWQVHVVLLPGKKHRGRTNIQRGPNPVFREKVTFAK
```

```
                                                          500
Xtropicalissyt16var2      LVPEEMPNHALRFRLYAVHKMNRVKMMGEKLYHLRNIRPDSENNLTLVLEPRSNLTSGDSQLSFTPVSHSESDSSTQSLSHGGVPELLVGLSYNPTTGRLSVEMIKGSHFRNL
Xtropicalissyt16var3    ● -----MPNHALRFRLYAVHKMNRVKMMGEKLYHLRNIRPDSENNLTLVLEPRSNLTSGDSQLSFTPVSHSESDSSTQSLSHGGVPELLVGLSYNPTTGRLSVEMIKGSHFRNL
Acarolinensissyt16var2    LSPEELSHYAIRFRLYSVRKMIKERMMGEQLFYLSNLNQEGEMKVTLVLEPRSNLCSGGSQISLSAISHSDSASSNQSLSHGGVPELLVGLSYNATTGRLSVEMIKGSHFRNL
GgallusSYT16var2          LEPHELGGHAVRFRLYAVHKVIGEKMMGEQLFYLSNISQEEEVKVTLVLEPRSNLTSADSQLSLSAISHSDSASSTQSLSHGGVPELLVGLSYNATTGRLSVEMIKGSHFRNL
TguttataSYT16var2         LEPQELSSHAVRFRLYAVHKVVGEKMMGEQLFYLRGISQEGEVKVTLLLEPRSNLSSADSQLSLSAISHSDSASSTQSLSHGGVPELLVGLSYNATTGRLSVEMIKGSHFRNL
MdomesticaSyt16var2       LQPEDLGRHAVQFRLYAALKMNREKMMGEKLFHLSQLHQGGKMKVTLVLEPRSNMSSGESQLSLSAVSHSDSASSTQSLSHGGVPELLVGLSYNATTGRLSVEIIKGSHFRNL
MmusculusSyt16var2        LEPRDVASCAVRFRLYAARKMTRERMMGEKLFCLSHLHPEGEMKVTLVLEPRSNLSSGESPLSPSVVSHSDSASSTQSLSHGGVPELLVGLSYNATTGRLSVEMIKGSHFRNL
MmusculusSyt16var3        LEPRDVASCAVRFRLYAARKMTRERMMGEKLFCLSHLHPEGEMKVTLVLEPRSNLSSGESPLSPSVVSHSDSASSTQSLSHGGVPELLVGLSYNATTGRLSVEMIKGSHFRNL
HsapiensSYT16var1         LEPRDVAACAVRFRLYAARKMTRERMMGEKLFYLSHLHPEGEMKVTLVLEPRSNISSGGSPLSPSAVSHSDSTSSTQSLSHGGAPELLVGLSYNATTGRLSVEMIKGSHFRNL
HsapiensSYT16var2a        -----------------------------------------------------------------------------------------------------------------
HsapiensSYT16var2b      ● --------------------MTRERMMGEKLFYLSHLHPEGEMKVTLVLEPRSNISSGGSPLSPSAVSHSDSTSSTQSLSHGGAPELLVGLSYNATTGRLSVEMIKGSHFRNL
HsapiensSYT16var3         LEPRDVAACAVRFRLYAARKMTRERMMGEKLFYLSHLHPEGEMKVTLVLEPRSNISSGGSPLSPSAVSHSDSTSSTQSLSHGGAPELLVGLSYNATTGRLSVEMIKGSHFRNL

                                                600
Xtropicalissyt16var2      AINRPPDTYGKLSLLDSVGHEISRCKTSVRRGQPNPVYKETFIFQVALFQLTDFTLMISIYNRRSMKRKEMIGWISLGKNSSGEEEQNHWLEMKESKGQQVCHWHTLLES
Xtropicalissyt16var3      AINRPPDTYGKLSLLDSVGHEISRCKTSVRRGQPNPVYKETFIFQVALFQLTDFTLMISIYNRRSMKRKEMIGWISLGKNSSGEEEQNHWLEMKESKGQQVCHWHTLLES
Acarolinensissyt16var2    AVNRPPDTYGKLCLLNSIGQEMSRCKTSVRRGQPNPVYKETFVFQVALFQLSDVTLMISIYNRRSIKRKEMIGWLSMGQNSSGEEEQNHWHEMKESKSQQICRWHTLLES
GgallusSYT16var2          AINRPPDTYGKLCLLNSVGQEMSRCKTSIRRGQPNPVYKETFIFQVALFQLSDVTLMISIYNKRSMKRKEMIGWISMGQNSSGEEEQSHWQEMKESKGLQVCRWHTLLES
TguttataSYT16var2         AINRPPDTYGKLCLRNSVGQEMSRCKTSIRRGQPNPVYKETFIFQVALFQLSDVTLLISIYNRRSMKRREMVGWISMGQSSSGEEEQSHWQEMRESQGTQVCRWHTLLES
MdomesticaSyt16var2       AINRPPDSYGKLFLLNSVGQEMSRCKTSIRRGQPNPVYKETFVFQVALFQLSDVTLMISIYNRRAMKRKEMIGWISLGQNSSGEEEQDHWQEMKETKGQQICRWHTLLES
MmusculusSyt16var2        AANRAPDTYGKLFLLNCVGQEMSRCKTSIRRGQPNPVYKETFVFQVALFQLSDVTLMISIYSRRTMKRKEMIGWVALGQNSSGEEEQEHWEEMKESKGQQTCRWHTLLES
MmusculusSyt16var3        AANRAPDTYGKLFLLNCVGQEMSRCKTSIRRGQPNPVYKETFVFQVALFQLSDVTLMISIYSRRTMKRKEMIGWVALGQNSSGEEEQEHWEEMKESKGQQTCRWHTLLES
HsapiensSYT16var1         AVNRAPDTYGKLFLLNSVGQEMSRCKTSIRRGQPNPVYKETFVFQVALFQLSDVTLMISVYNRRTMKRKEMIGWIALGQNSSGEEEQDHWEEMKETKGQQICRWHTLLES
HsapiensSYT16var2a        --------------------------------------------------------------------------------------------------------------
HsapiensSYT16var2b        AVNRAPDTYGKLFLLNSVGQEMSRCKTSIRRGQPNPVYKETFVFQVALFQLSDVTLMISVYNRRTMKRKEMIGWIALGQNSSGEEEQDHWEEMKETKGQQICRWHTLLES
HsapiensSYT16var3         AVNRAPDTYGKLFLLNSVGQEMSRCKTSIRRGQPNPVYKETFVFQVALFQLSDVTLMISVYNRRTMKRKEMIGWIALGQNSSGEEEQDHWEEMKETKGQQICRWHTLLES
```
