# Supplementary material for: A manual collection of Syt, Esyt, Rph3a, Rph3al, Doc2, and Dblc2 genes from 46 metazoan genomes - an open access resource for neuroscience and evolutionary biology
Source: BMC Genomics. 2010 Jan 15;11:37. doi: 10.1186/1471-2164-11-37 (PMC2823689; doi:10.1186/1471-2164-11-37)
Supplement: Additional file 33 — Alignment of the vertebrate Syt14 sequences. Amino acid position is marked every hundred amino acids approximately, at the top of each page of the alignment. Splice variants are included and highlighted with black dots where they differ. Intron position and phase is indicated with a coloured bar between amino acids. Black bars indicate phase 0 introns. Red bars indicate phase +1 introns. X residues indicate where a portion of sequence is missing. [file 1471-2164-11-37-S33.PDF]

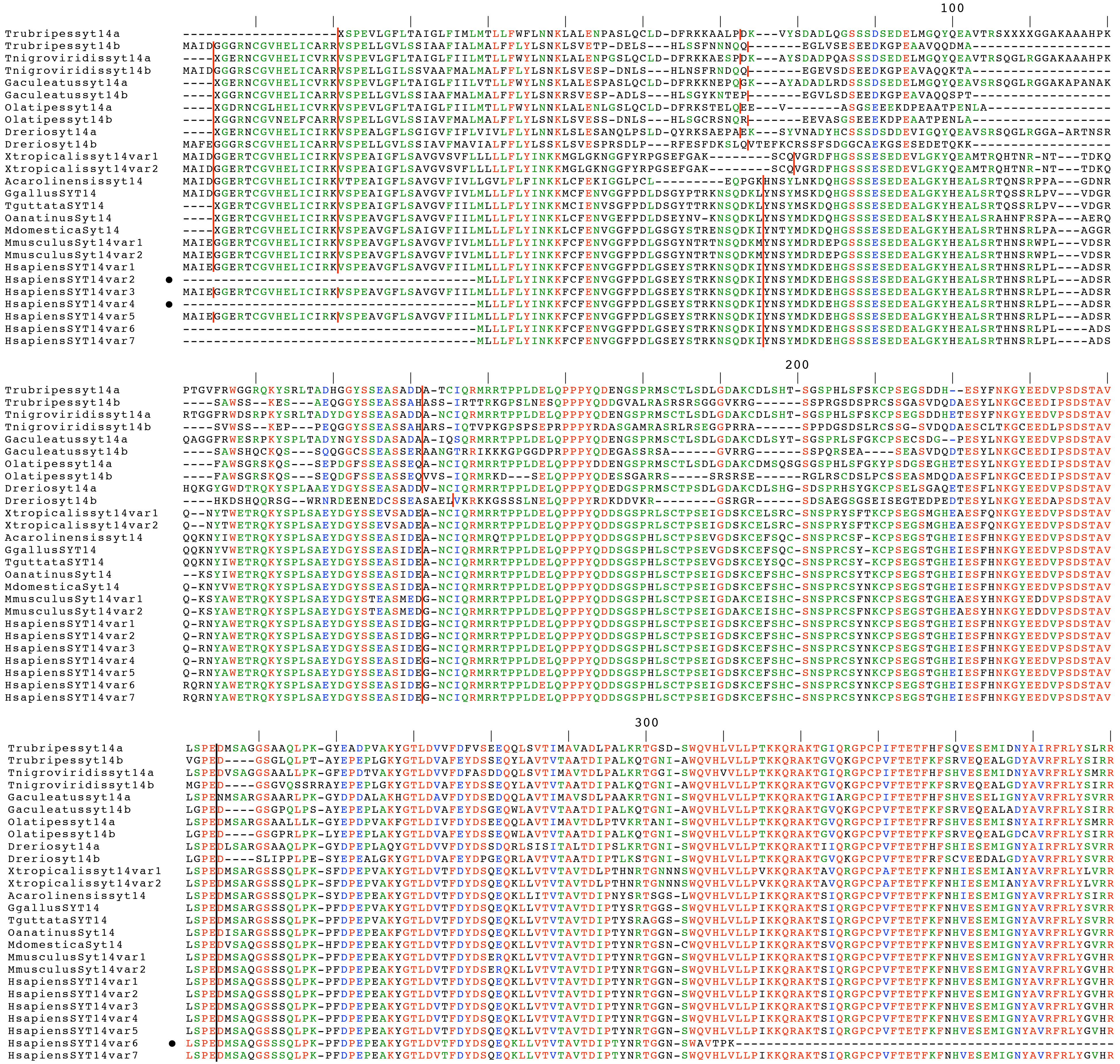
100
200
300
Trubripessyt14a
Trubripessyt14b
Tnigroviridissyt14a
Tnigroviridissyt14b
Gaculeatussyt14a
Gaculeatussyt14b
Olatipessyt14a
Olatipessyt14b
Dreriosyt14a
Dreriosyt14b
Xtropicalissyt14var1
Xtropicalissyt14var2
Acarolinensissyt14
GgallusSYT14
TguttataSYT14
OanatinusSyt14
MdomesticaSyt14
MmusculusSyt14var1
MmusculusSyt14var2
HsapiensSYT14var1
HsapiensSYT14var2
HsapiensSYT14var3
HsapiensSYT14var4
HsapiensSYT14var5
HsapiensSYT14var6
HsapiensSYT14var7

```
                                                                 400
                                                                  |
Trubripessyt14a           MKKEKVFGEKVFYLTKFNLQGKMSMPVMLEPCCALPAGGSQVSLSDITCSESASSFQSVNQTSTPEILVGLAYNATTGRLSVEIIRGIHFRNLAANKPP-------NGLFCCLKHFIGGQ
Trubripessyt14b           MKKEKVLGEKVFYLTKLNLQGKIALPVTLEPGSELAGCGSLVSVSR---SAGALSYRSSEDSSLPEILLGLIYNSATGQLSAEVIKGSYFKTAVSDKPV-------NGLFCCVKHFVGGQ
Tnigroviridissyt14a       MKKEKVFGEKVFYLTKLNLQGKMSMPVMLDPCCALPGGESQGSLSDMTCSESASSFQSVGQSSTPEILVGLVYNATTGRLSVEIIKGIHFKNLAANKPP-------NGLFCCLKHFIGGQ
Tnigroviridissyt14b       MKKEKVLGEKVFYLTKLNLQGKIALPVTLEPGSELAGCGSLVSVSR---SAGALSYRSAEDTSLPEILLGLVYNSATGQLSAEVIKGSYFKTAVSDKPV-------NGLFCCVKHFVGGQ
Gaculeatussyt14a          MKKEKVLGEKVFYLTKLNLQGKVSVPVMLDPCCALPGGESQVSLSDTTCSESASSFQSVSQTSTPEILVGLVYNATTGRLSVEIIKGIHFKN-AANKPP-------NGLFCCLKHLIGGQ
Gaculeatussyt14b          MKKEKVLGEKVFYLTKLNLQGKIALPVTLEPGSELTGCGSVVSVSR---SAGALSHRSTEDSSMPEILLGLIYNSATGQLSAEVIQGSHFKTTASDKPV-------NGLFCCVKHFVGGQ
Olatipessyt14a            MKKEKVFGEKVFYLTKLNLQGKMSVPVIILDPCCPIPGGESQVSLSDMTCSESASSFQSVSQASTPEILVGLVYNATTGRLSVEIIKGIHFKNLAANKPP-------NGLFCCLKHLIGGQ
Olatipessyt14b            MKKEKVLGEKVFYLTKLNLQGKIALPVTLEPGSELPGCGSLVSVSR---SAGALSYRSTED-SLPEILLGLIYNSATGRLSAEVIQGNHFKTAASDKPI-------SDLFCCVKHFVGGQ
Dreriosyt14a              MKKEKALGEKVFYLTKLNLQGKMSVPIILDPYCNIPGSDSQASMSDVSCSETASSYPSAAQGSAPEILLGLVYNATTGRLSVEVIKGSHFKNLAANKPP-------NGLFCCLKHLIGGQ
Dreriosyt14b              MKKEKVLGEKVFYLTKLNLQGKLALPVTLEPGTSVPGCGSVVSVSR---SAGALSCRSTGESSIPELLLGLLYNSTTGRLSAEVIKGSHFKNSASDKLP-------IGLFCCVKHFISGQ
Xtropicalissyt14var1      MKKERMVGEKIFYLTKLNLQGKLSLPVVLEPAYSLSGCDSQMSMSEMSCTESTSSCQSLVHGSVPEILIGLLYNATTGRLSAEVIKGSHFKNLSANRPP--------RLFCCLKHLIGGQ
Xtropicalissyt14var2  ●   MKKERMVGEKIFYLTKLNLQGKLSLPVVLEPAYSLSGCDSQMSMSEMSCTESTSSCQSLVHGSVPEILIGLLYNATTGRLSAEVIKGSHFKNLSANRPP---------------------
Acarolinensissyt14        VKKERILGEKTFYLTKLNLQGKMSVPVTLEPAYNISGCDSQMSMSEMSCSESTSSCQSLAHGSAPEILVGLLYNATTGRLAAEVIKGSHFKNLAANRPP-------NGLFCCLKHLIGGQ
GgallusSYT14              MKKERIVGEKIFHLTKLNLQGKMSVPVILEPSYSLSGCDSQMSMSEVSCSESTSSCQSLVHGSAPEILVGLLYNATTGRLSAEVIKGSHFKNLAANRPP-------NGLFCCLKHLIGGQ
TguttataSYT14             MKKERIVGEKIFYLTKLNLQGKMSVPVILEPSYSLPGCDSQMSMSEVSCSESTSSCQSLVHGSAPEILIGLLYNATTGRLSAEVIKGSHFKNLAANRPP-------NGLFCCLKHLIGGQ
OanatinusSyt14            MKKEKIVGEKIFYLTKLNLQGKMSLPVILEPSY-LSGCDSQMSMSEISC-ESTSSCQSLEHGSVPEILIGLLYNATTGRLSAEVIKGSHFKNLAANRPP-------NGLFCCLKHLIGGQ
MdomesticaSyt14           MKKEKIVGEKIFYLTKLNLQGKMSLPVILEPSYSISGCGSQMSMSEMSCSESTSSCQSLEHGSTPEILIGLLYNATTGRLSAEVIKGSHFKNLAANRPP-------NGLFCCLKHLIGGQ
MmusculusSyt14var1        MKKEKIVGEKIFYLTKLNLQGKMSLPVILEPSYNPSGCDSQVSLSEASCGDSTSSCQSLQHGSVPEILIGLLYNATTGRLSAEVIKGSHFKNLAANRPP---------------------
MmusculusSyt14var2    ●   MKKEKIVGEKIFYLTKLNLQGKMSLPVILEPSYNPSGCDSQVSLSEASCGDSTSSCQSLQHGSVPEILIGLLYNATTGRLSAEVIKGSHFKNLAANRPP-------NGLFCCLKHLIGGQ
HsapiensSYT14var1         MKKEKIVGEKIFYLTKLNLQGKMSLPVILEPSYNHSGCDSQMSVSEMSCSESTSSCQSLEHGSVPEILIGLLYNATTGRLSAEVIKGSHFKNLAANRPP-------NGLFCCLKHLIGGQ
HsapiensSYT14var2         MKKEKIVGEKIFYLTKLNLQGKMSLPVILEPSYNHSGCDSQMSVSEMSCSESTSSCQSLEHGSVPEILIGLLYNATTGRLSAEVIKGSHFKNLAANRPP-------NGLFCCLKHLIGGQ
HsapiensSYT14var3     ●   MKKEKIVGEKIFYLTKLNLQGKMSLPVILEPSYNHSGCDSQMSVSEMSCSESTSSCQSLEHGSVPEILIGLLYNATTGRLSAEVIKGSHFKNLAANRPP---------------------
HsapiensSYT14var4         MKKEKIVGEKIFYLTKLNLQGKMSLPVILEPSYNHSGCDSQMSVSEMSCSESTSSCQSLEHGSVPEILIGLLYNATTGRLSAEVIKGSHFKNLAANRPP---------------------
HsapiensSYT14var5     ●   MKKEKIVGEKIFYLTKLNLQGKMSLPVILEPSYNHSGCDSQMSVSEMSCSESTSSCQSLEHGSVPEILIGLLYNATTGRLSAEVIKGSHFKNLAANRPPTFHPLLSDGLFCCLKHLIGGQ
HsapiensSYT14var6         ----------------------------------------------------------------------------------------------------------------------------
HsapiensSYT14var7     ●   MKKEKIVGEKIFYLTKLNLQGKMSLPVILEPSYNHSVSISSNGLNTVYVHGL------------------------------------------------------------------------

                                           500
                                            |
Trubripessyt14a           VYIIRDTYVKLTLLNSMGHQMSKCKTSICRGQPNPTFKETFLFQVALFQLSDVTLILSVYSRRS-MKRKEMIGWISLGLNSSGEEELTHWTLMKESKGQQVCRWHSLLES
Trubripessyt14b           LYIIRDTYVKLTMLDSKGKEMSKCKTAVCRGQPNPTYKETFVFQVALFQLSEVSLVLTVFCSRSSMRPRERLGWVSLGLNSSSEEQQAHWTEMKEAEGQQVCHWHTLAVT
Tnigroviridissyt14a       VYIIRDTYVKLTLLNSMGHEMSKCKTSVCRGQPNPTFKETFVFQVALFQLSDVTLILSVYSKRS-MKRKEMIGWISLGLNSSGEEELTHWTQMKESKGQQVCRWHSLLES
Tnigroviridissyt14b       LYILRDTYVKLTMLDSKGKEMSKCKTAVCRGQPNPTYKETFLFQVALFQLSEVSLVLTVFCSRSSMRPRERLGWVSLGLNSSSEEQQAHWTEMKEAEGQQVCHWHTLTIT
Gaculeatussyt14a          VYIIRDTYVKLTLLNSMGHEMSKCKTSICRGQPNPTYRETFVFQVALFQLSDVTLILSVYNKRS-MKRKEMIGWISLGLNSSGEEELTHWTQMKESKGQQVCRWHSLLES
Gaculeatussyt14b          LYIMRDTYVKLTMLDSKGKEMSKYKTAVCRGQPNPTYKETFLFQVALFQLSEVSLVLSVFCRRSSMRPRERLGWVSLGLSSTCEEQQSHWTEMKEAEGRQVCHWHTLTDT
Olatipessyt14a            VYIIRDTYVKLTLLNSMGHEMSKCKTSICRGQPNPTYKETFVFQVALFQLSDVTLILSVYNKRS-MKRKEMIGWISLGLNSSGEEELTHWTQMKESKGQQVCHWHSLLES
Olatipessyt14b            LYIMRDTYVKLTMLDSKGKEMSKCKTATCRGHPNPTYKETFVFQVALFQLSEVSLVVSVFCRRSSMRPRERLGWVSLGLNSTSEEQLVHWTEMREAEGQQICHWHTLTDT
Dreriosyt14a              VYIIRNTYVKLTLLNSMGQEMSKCKTSVCRGQPNPTYKETFVFQVALFQLSDVTLILSVYNKRS-MKRKEMIGWISLGLNSSGEEELTHWTEMKESKGQQVCRWHSLLES
Dreriosyt14b              LYIMRDTYVKLTMLDSKGKEMSKCKTSVCRGQPNPTYKETFVFQVALFQLSEVTLQVSVYSRRSSMKRRERVGWVALGLNSTTEEQEEHWTQMKESEGQQVCHWHTLIDS
Xtropicalissyt14var1      VYIIRDTYVKLTLMNNSMGQEMSKCKTSIRRGQSNPLYKETFIFQVALFQLSEVTLILSVYNKRS-MKRKELIGWISLGLNSSGDEQLNHWTEMKESKGQQVCRWHTLLES
Xtropicalissyt14var2      -----NTYVKLTLMNSMGQEMSKCKTSIRRGQSNPLYKETFIFQVALFQLSEVTLILSVYNKRS-MKRKELIGWISLGLNSSGDEQLNHWTEMKESKGQQVCRWHTLLES
Acarolinensissyt14        VYIIRDTFVKLTLLNSMGQEMSKCKTSIRRGQPNPVYKETFIFQVALFQLSDVTLILSVYNKRS-MKRKEMIGWIALGLNSSGEEELNHWTEMKESKGQQVCRWHALLET
GgallusSYT14              VYIIRDTYVKLTLLNSMGQEMSKCKTSIRRGQPNPVYKETFVFQVALFQLSDVTLILSVYNKRS-MKRKEMIGWISLGLNSSGEDELNHWTEMKESKGQQVCRWHTLLES
TguttataSYT14             VYIIRDTYVKLTLLNSMGQEMSKCKTSIRRGQPNPVYKETFVFQVALFQLSDVTLILSVYNKRS-MKRKEMIGWISLGLNSSGEDELNHWTEMKESKGQQVCRWHTLLES
OanatinusSyt14            VYIIRDTYVKLTLLNSMGQEMSKCKTSIRRGQPNPIYKETFVFQVALFQLSDVTLILSVYNKRS-MKRKEMIGWISLGLNSSGEEELNHWTEMKESKGQQVCRWHSLLES
MdomesticaSyt14           VYIIRDTYVKLTLLNSMGQEMSKCKTSIRRGQPNPIYKETFVFQVALFQLSDVTLILSVYNKRS-MKRKEMIGWISLGLNSSGEEELNHWTEMKESKGQQVCRWHSLLES
MmusculusSyt14var1        -----NTYVKLTLLNSMGQEMSKCKTSTRRGQPNPVYKETFVFQVALFQLSDVTLILSVYNRRS-MKRKEMIGWISLGLNSSGEEELRHWTAMKESKGQQVCRWHALLES
MmusculusSyt14var2        VYIIRDTYVKLTLLNSMGQEMSKCKTSTRRGQPNPVYKETFVFQVALFQLSDVTLILSVYNRRS-MKRKEMIGWISLGLNSSGEEELRHWTAMKESKGQQVCRWHALLES
HsapiensSYT14var1         VYIIRDTYVKLTLLNSMGQEMSKCKTSIRRGQPNPVYKETFVFQVALFQLSDVTLILSVYNKRS-MKRKEMIGWISLGLNSSGEEELNHWTEMKESKGQQVCRWHALLES
HsapiensSYT14var2         VYIIRDTYVKLTLLNSMGQEMSKCKTSIRRGQPNPVYKETFVFQVALFQLSDVTLILSVYNKRS-MKRKEMIGWISLGLNSSGEEELNHWTEMKESKGQQVCRWHALLES
HsapiensSYT14var3         -----NTYVKLTLLNSMGQEMSKCKTSIRRGQPNPVYKETFVFQVALFQLSDVTLILSVYNKRS-MKRKEMIGWISLGLNSSGEEELNHWTEMKESKGQQVCRWHALLES
HsapiensSYT14var4         -----NTYVKLTLLNSMGQEMSKCKTSIRRGQPNPVYKETFVFQVALFQLSDVTLILSVYNKRS-MKRKEMIGWISLGLNSSGEEELNHWTEMKESKGQQVCRWHALLES
HsapiensSYT14var5         VYIIRDTYVKLTLLNSMGQEMSKCKTSIRRGQPNPVYKETFVFQVALFQLSDVTLILSVYNKRS-MKRKEMIGWISLGLNSSGEEELNHWTEMKESKGQQVCRWHALLES
HsapiensSYT14var6         --------------------------------------------------------------------------------------------------------------
HsapiensSYT14var7         --------------------------------------------------------------------------------------------------------------
```
