# Supplementary material for: A manual collection of Syt, Esyt, Rph3a, Rph3al, Doc2, and Dblc2 genes from 46 metazoan genomes - an open access resource for neuroscience and evolutionary biology
Source: BMC Genomics. 2010 Jan 15;11:37. doi: 10.1186/1471-2164-11-37 (PMC2823689; doi:10.1186/1471-2164-11-37)
Supplement: Additional file 35 — Alignment of the vertebrate Syt17 sequences. Amino acid position is marked every hundred amino acids approximately, at the top of each page of the alignment. Splice variants are included and highlighted with black dots where they differ. Intron position and phase is indicated with a coloured bar between amino acids. Black bars indicate phase 0 introns. Red bars indicate phase +1 introns. Blue bars indicate phase +2 introns. A possible motif just upstream of the C2A domain, is indicated. X residues indicate where a portion of sequence is missing. [file 1471-2164-11-37-S35.PDF]

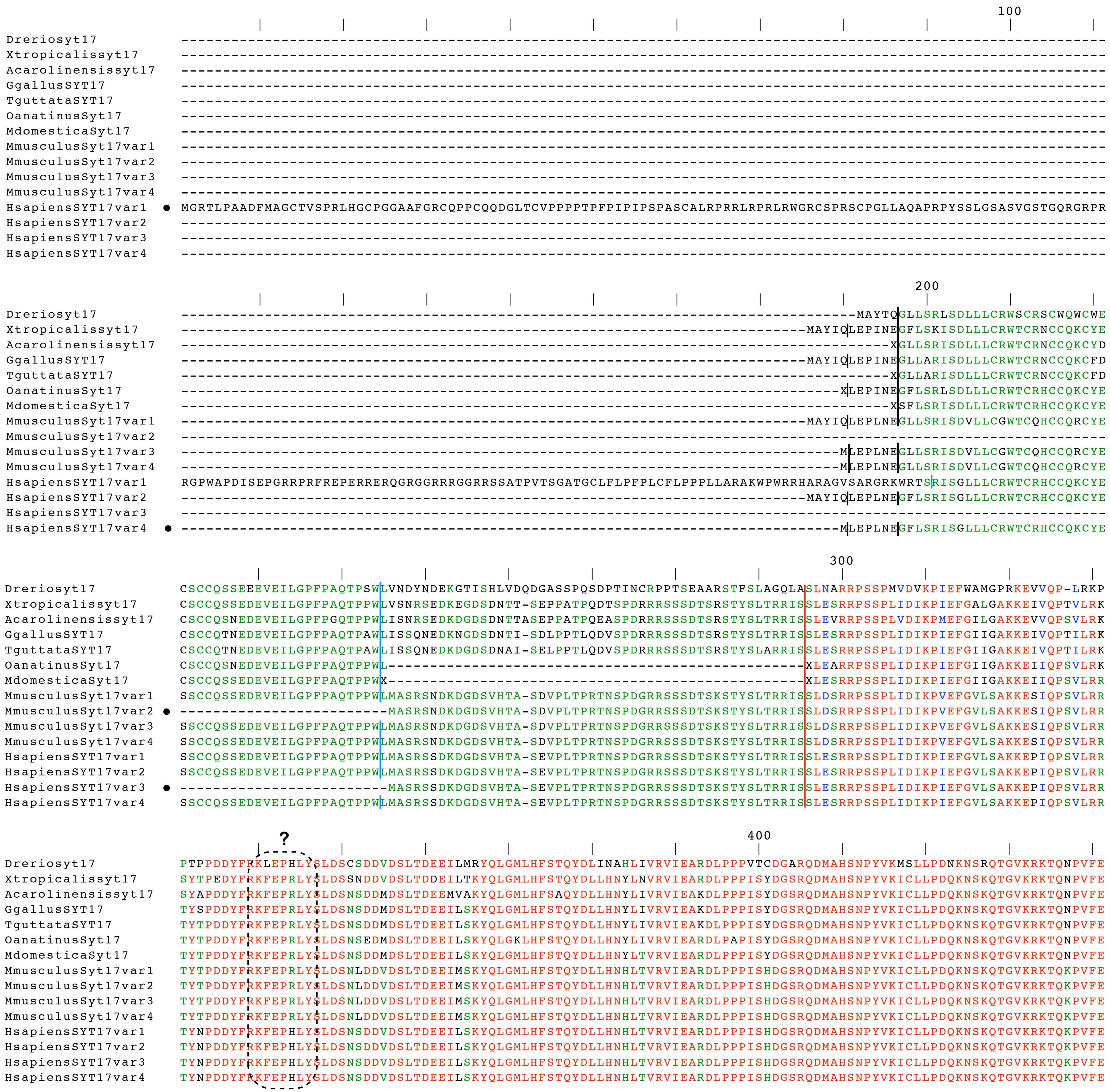
100
200
300
400
?
Dreriosyt17
Xtropicalissyt17
Acarolinensissyt17
GgallusSYT17
TguttataSYT17
OanatinusSyt17
MdomesticaSyt17
MmusculusSyt17var1
MmusculusSyt17var2
MmusculusSyt17var3
MmusculusSyt17var4
HsapiensSYT17var1
HsapiensSYT17var2
HsapiensSYT17var3
HsapiensSYT17var4

```
                                                                              500
Dreriosyt17               ERFTFDLPFLEAQRRTLLLSVVDFDKFSRHCVIGKVALPLSEVDLVKGGHWWKALVPSSQNEVELGELLLSLNYLPSAGRLNVDIIRAKQLLQTDMCQGSDPFVKVQLVTG
Xtropicalissyt17          ERYTFEIQFLEAQRRTLLLTIVDFDKFSRHCVIGKVAMPLNEVDLVKGGHWWKAIIPSSQNEVELGELLLSLNYLPSAGRLNVDIIRAKQLLQTDMSQGSDPFVKIQLVHG
Acarolinensissyt17        ERYTFEIPFLEAQRRTLLLNIVDFDKFSRHCVIGKVSMPLSDVDLVKGGHWWKALIPSSQNEVELGELLLSLNYLPSAGRLNVDIIRAKQLLQTDMSQGSDPFVKIQLVHG
GgallusSYT17              ERYTFEIPFLEAQRRTLLLTVVDFDKFSRHCVIGKVSMPLGDVDLVKGGHWWKALVPSSQNEVELGELLLSLNYLPSAGRLNVDIIRAKQLLQTDMSQGSDPFVKIQLVHG
TguttataSYT17             ERYTFEIPFLEAQRRTLLLTVVDFDKFSRHCVIGKVSMPLSDVDLVKGGHWWKALVPSSQNEVELGELLLSLNYLPSAGRLNVDIIRAKQLLQTDMSQGSDPFVKIQLVHG
OanatinusSyt17            ERYTFEIPFLEAQRRTLLLTMVDFDKFSRHCVIGKVSMPLSEVDLVKGGHWWKALVPSSQNEVELGELLLSLNYLPSAGRLNVDVIRAKQLLQTDMSQGSDPFVKVQLVHG
MdomesticaSyt17           ERYTFEIPFLEAQRRTLLLTIVDFDKFSRHCVIGKVSVPLSEVDLVKGGHWWKALVPSSQX---------------------------------------XPFVKIQLVHG
MmusculusSyt17var1        ERYTFEIPFLEAQRRTLLLTVVDFDKFSRHCVIGKVAVPLCEVDLVKGGHWWKALIPSSQNEVELGELLLSLNYLPSAGRLNVDIIRAKQLLQTDVSQGSDPFVKIQLVHG
MmusculusSyt17var2        ERYTFEIPFLEAQRRTLLLTVVDFDKFSRHCVIGKVAVPLCEVDLVKGGHWWKALIPSSQNEVELGELLLSLNYLPSAGRLNVDIIRAKQLLQTDVSQGSDPFVKIQLVHG
MmusculusSyt17var3        ERYTFEIPFLEAQRRTLLLTVVDFDKFSRHCVIGKVAVPLCEVDLVKGGHWWKALIPSSQNEVELGELLLSLNYLPSAGRLNVDIIRAKQLLQTDVSQGSDPFVKIQLVHG
MmusculusSyt17var4        ERYTFEIPFLEAQRRTLLLTVVDFDKFSRHCVIGKVAVPLCEVDLVKGGHWWKALIPSSQNEVELGELLLSLNYLPSAGRLNVDIIRAKQLLQTDVSQGSDPFVKIQLVHG
HsapiensSYT17var1         ERYTFEIPFLEAQRRTLLLTVVDFDKFSRHCVIGKVSVPLCEVDLVKGGHWWKALIPSSQNEVELGELLLSLNYLPSAGRLNVDVIRAKQLLQTDVSQGSDPFVKIQLVHG
HsapiensSYT17var2         ERYTFEIPFLEAQRRTLLLTVVDFDKFSRHCVIGKVSVPLCEVDLVKGGHWWKALIPSSQNEVELGELLLSLNYLPSAGRLNVDVIRAKQLLQTDVSQGSDPFVKIQLVHG
HsapiensSYT17var3         ERYTFEIPFLEAQRRTLLLTVVDFDKFSRHCVIGKVSVPLCEVDLVKGGHWWKALIPSSQNEVELGELLLSLNYLPSAGRLNVDVIRAKQLLQTDVSQGSDPFVKIQLVHG
HsapiensSYT17var4         ERYTFEIPFLEAQRRTLLLTVVDFDKFSRHCVIGKVSVPLCEVDLVKGGHWWKALIPSSQNEVELGELLLSLNYLPSAGRLNVDVIRAKQLLQTDVSQGSDPFVKIQLVHG

                                                                    600
Dreriosyt17               LKLMKSKKTSCMRGTIDPCYNESFSFRVPQEDLCEVSLVLTVYGHNVKSSNDFVGRIVIGQFSSGPQETTHWRRLLSSQRTPVEQWHSLRSRAECDRVSPASLEVT
Xtropicalissyt17          LKLAKTKKTSCMRGTIDPFYNESFSFKVPQEELENVSLVFTVYGHNMKTSNDFIGRIVIGQYASGSPESNHWRRMLNSNRTAVEQWHSLRSRAECDRVSPASLEVT
Acarolinensissyt17        LKLAKTKKTSCMKATIDPFYNESFSFKVPQEELENASLVFTVYGHNVKSSNDFIGRIVIGQYATGSPESKHWRRMLGSHRTAVEQWHSLRSRAECDRVSPASLEVT
GgallusSYT17              LKLTKTKKTSCMRGTIDPFYNESFSFKVPQEELENASLVFTVYGHNVKSSNDFIGRIVIGQYSTGAPESNHWRRMLNAHRTAVEQWHSLRSREECDRVSPASLEVT
TguttataSYT17             LKLTKTKKTSCMRGTIDPFYNESFSFKVPQEELENASLVFTVYGHNVKSSNDFIGRIVIGQYSTGAPESNHWRRMLSAHRTAVEQWHSLRSREDCDRVSPASLEVT
OanatinusSyt17            LKLAKTKKTSFMRGTIDPFYNESFSFKVPQEELENASLVFTVYGHNVKSSNDFVGRIVIGQYSSGSSESNHWRRMLNAHRTAVEQWHSLRSRDECDRVSPASLEVT
MdomesticaSyt17           LKLVKSKKTSFMRGTIDPFYNESFSFKVPQEELENASLVFTVYGHNMKSSNDFIGRIVIGQYSTGSSESNHWRRMLNTHRTAVEQWHSLRSRAECDRVSPASLEVT
MmusculusSyt17var1        LKLVKTKKTSFLRGTIDPFYNESFSFKVPQEELENASLVFTVFGHNMKSSNDFIGRIVIGQYSSGPSESNHWRRMLNTHRTAVEQWHSLRSRAECDRVSPASLEVT
MmusculusSyt17var2        LKLVKTKKTSFLRGTIDPFYNESFSFKVPQEELENASLVFTVFGHNMKSSNDFIGRIVIGQYSSGPSESNHWRRMLNTHRTAVEQWHSLRSRAECDRVSPASLEVT
MmusculusSyt17var3        LKLVKTKKTSFLRGTIDPFYNESFSFKVPQEELENASLVFTVFGHNMKSSNDFIGRIVIGQYSSGPSESNHWRRMLNTHRTAVEQWHSLRSRAECDRVSPASLEVT
MmusculusSyt17var4      ● LKLVKTKKTSFLRGTIDPFYNESFSFKVPQEELENASLVFTGKVTYMLMKP-------------------------------------------------------
HsapiensSYT17var1       ● LKLVKTKKTSFLRGTIDPFYNESFSFKVPQEELENASLVFTGSNSPIPACELSSHPAHGISPWIPSPGNEHFHGIKKQVKAIKVE---------------------
HsapiensSYT17var2         LKLVKTKKTSFLRGTIDPFYNESFSFKVPQEELENASLVFTVFGHNMKSSNDFIGRIVIGQYSSGPSETNHWRRMLNTHRTAVEQWHSLRSRAECDRVSPASLEVT
HsapiensSYT17var3         LKLVKTKKTSFLRGTIDPFYNESFSFKVPQEELENASLVFTVFGHNMKSSNDFIGRIVIGQYSSGPSETNHWRRMLNTHRTAVEQWHSLRSRAECDRVSPASLEVT
HsapiensSYT17var4         LKLVKTKKTSFLRGTIDPFYNESFSFKVPQEELENASLVFTVFGHNMKSSNDFIGRIVIGQYSSGPSETNHWRRMLNTHRTAVEQWHSLRSRAECDRVSPASLEVT
```
