# Supplementary material for: A manual collection of Syt, Esyt, Rph3a, Rph3al, Doc2, and Dblc2 genes from 46 metazoan genomes - an open access resource for neuroscience and evolutionary biology
Source: BMC Genomics. 2010 Jan 15;11:37. doi: 10.1186/1471-2164-11-37 (PMC2823689; doi:10.1186/1471-2164-11-37)
Supplement: Additional file 36 — Alignment of the invertebrate Sytalpha sequences. Amino acid position is marked every hundred amino acids approximately, at the top of each page of the alignment. Intron position and phase is indicated with a coloured bar between amino acids. Black bars indicate phase 0 introns. Red bars indicate phase +1 introns. Blue bars indicate phase +2 introns. The widely conserved motif of unknown function, just upstream of the C2A domain, is indicated. X residues indicate where a portion of sequence is missing. [file 1471-2164-11-37-S36.PDF]

```
                                                                                                           100
CapitellaSytalpha           ---------MSSAEGPPEYMYAVYVTVPLLALVGVGLLVYICCAHKYR--FNWFETSLLQESSSSGEQRSFNFYDEKSCFNTQFSPNFYAFGPRSSLVAGTLLPSHANTGCGLA
LgiganteaSytalpha           ----------MSRPSPSAYLIAVYVAVPAVVLLLAIIVVYIYCTKRHR--LNWYQRTLLEE------------YDRGT------------LGPLLSEGAKTGLMS-GKGGRGMR
CsavignyiSytalpha1          ------------------------------------------------------------------------------------------------------------------
CsavignyiSytalpha2          ------------MAALSGGAVAGICIVSVLGAALLAWVIYYVIKKRQR------DTEYLDRCGRLWKKYGHRERPEMMPKYPYAGATFIRAPVPGQQFTSRELKASTSTSQEFV
CintestinalisSytalpha1      -----------------MLMYCIQCIVVVFFALFLVSLTACIKTYK-R------------------------------------------------------------------
CintestinalisSytalpha2      ------------MPAISGAAIAGIVIVSALGIALLVWGIYYIIQKRKR------DAAYLDRCGRLWNKYGNRDRPEMMPKYPYAGATFVRTPVPGQQFTSRELKSSMGTSQEFV
SpurpuratusSytalpha         -----------------MEVYLIIASISAALTLGIVLAVLVCVCYIQS--PGKLRKEMEDDS---------DFEAQPAMFLPEPPNSDDEFTPGMKLPSFRRFDSSID------
BfloridaeSytalpha           ------------------------------------------------------------------------------------------------------------------
NvitripennisSytalpha        ------------MDPHGPEIVAILGAIGATAGAVSAVVVYALCARRRRVPLNWFEKDLLDRAEEAEKSSREEHALAS-----------------LHPTLGILGKQASSGAASIK
AgambiaeSytalpha            MKSPPIASWFSQSDGVNPTQIAIYGSVSFLVVSLLGLLIYITCSKRYR--LNWYEKNLLESAKEREAAQRGSPRQSTGACGPSPV--------LGETTGEDSGDSACSSSRSVI
DmelanogasterSytalpha       ------MDIVIREEDISLAQIGVYASVSFLVVSAVGAALYTTCSKRYR--LNWFEQNLLESANEKDEDQQ-REALVAGAVGYN-VDNVNE-VPRGKYSSGNAGNLSPTSLKSED
DsimulansSytalpha           ------MDIVIREEDISLAQIGVYASVSFLVVSAVGAALYTTCSKRYR--LNWFEQNLLESANEKDEDQQ-REALVAGAVGYN-VDNVNE-VPRGKYSSGNAGNLSPTSLKSED
DsecheliaSytalpha           ------MDIVIREEDISLAQIGVYASVSFLVVSAVGAALYTTCSKRYR--LNWFEQNLLESANEKDEDQQ-REALVAGAVGYN-VDNVNE-VPRGKYSSGNAGNLSPTSLKSED
DerectaSytalpha             ------MDIVIREEDISLAQIGVYASVSFLVVSAVGAALYTTCSKRYR--LNWFEQNLLESANEKDEDQQ-REALVAGAVGYN-VDNVNE-VPRGKYSSGNAGNLSPTSLKSED
DyakubaSytalpha             ------MDIVIREEDISLAQIGVYASVSFLVVSAVGAALYTTCSKRYR--LNWFEQNLLESANEKDEDQQ-RESLVAGAVGYN-VDNVNE-VPRGKYSSGNAGNISPTSLKSED
DananassaeSytalpha          ------MDIVIREEDISLAQIGVYASVSFLVVSAVGAALYTTCSKRYR--LNWFEQNLLESANEKEEDQQ-REALVAGAVGYN-VDNVNE-VPRGKFGTGNAGNLSPTSLKSED
DpseudoobscuraSytalpha      ------MDILIREEDISLAQIGVYASVSFLVVSAVGAALYTTCSRRYR--LNWFEQNLLESANEKDEDQQ-REALVAGAAGYN-VDNLNE-CSRG--------NLSPTSLKNDE
DpersimilisSytalpha         -----------------------------------------------------------------------XEALVAGAAGYN-VDNLNE---------CSRGNLSPTSLKNDE
DwillistoniSytalpha         ------MDIVIREEDISLAQIGVYASVSFLVVSAVGAALYTTCSKRYR--LNWFEQNLLESASEKDEDQQ-RQALVSGAVGYN-VDNINE-CARGNKTNG--GNLSPTSIKSED
DvirilisSytalpha            ------MDIVIREEELSLTQIGVYASVSFLVVSAVGAALYTTCSKRYR--LNWFEKNLLESASEKDEEQQSREALVAGAVGYN-VDNLHEGVPRGKLTHNTAGNLSPTSLKSED
DmojavensisSytalpha         ------MDIVIREEELSLTQIGIYASVSFLVVSAVGAALYTTCSRRYR--LNWFEQNLLESASEKDEEQQSREALVAGTVGYN-VDNLNECVARGKLASSNGGNMSPTSLKSED
DgrimshawiSytalpha          ------MDIVIREEELSLTQIGVYASVSFLVVSAVGAALYTTCSKRYR--LNWFEQNLLESASENDEEQQSREALVSGAVGYNNVDNVHGGKFN---PHNNVGNLSPTSLKSED

                                                                                                200
CapitellaSytalpha           NPVTPTVTCVYQNKGGTPYQALPREDTPLSPSEKFW---VPPTVLNKKRAQSLIPERLSESVMLVQ-VPSGPAHR---RRRASMQDALDHTKINASLYIDRPVALQRQQSIGNI
LgiganteaSytalpha           SSSLKSEWMIYVKKMGSSPSS-PTNDA----SEKFW---VPPNVLERKRAQSLVPEKIVQPIMSVDGLPSTPPPAYTSRRRASMHDAIDLTKIDARLYEKKP--LVRQTSVSSI
CsavignyiSytalpha1          ----------------------------------------------------------------XLVVSPAHASQHLGRRRSSMQMPIDLRQIDPRLYKVK--DISLIVVDSTD
CsavignyiSytalpha2          VPNRKATDPNER----------------------------------------------------------------DWEKKSSMYAPIDPGSINPRLYSD-----DFSGQEDSP
CintestinalisSytalpha1      ------------------------------------------------------------------SLNVAPGGQNLNRRRSSMQIPIDLRQIDPKLYKVS---LSADGVDSTE
CintestinalisSytalpha2      LPQRKTAEGAD----------------------------------------------------------------DADNKNLSMYAPIDPDLINPRLYSN-----DFTAQQDSH
SpurpuratusSytalpha         ------------------------GSES---TTSSS---TPP--LSLYPANFTIPRAPSSTISDLSIEKAQPPIS---PRTPSMSRKLDPAQLDPTSH------MYKEMSDGS-
BfloridaeSytalpha           ------------------------------------------------------------------------------------------------------------------
NvitripennisSytalpha        TEDEWQQPREGIFNNRVKETRDVGRHLS----------ECSCDEAAGTSMSPLAPPLPGGGALVASDERMVILRS---LPLTSCTSNGSESSCSVSAHARLSSTDSDDISCPRS
AgambiaeSytalpha            AAPSGAVAIASTG-------KHVVLVTS-----------SPAKPTTSMVATVAGGSRSSTTGAAAS--------------KVDTSQG---TKIDMLDSASTC--STVSRDE-LG
DmelanogasterSytalpha       NDPAFWVPASVTS--TAAIQQQVSNTTE----ESAP--PT--SPTGSLKSNTLSYCSTTSVPIARSDKHVVLAMHPSRPRVSSMNAKLDHTKIDMTLYRSHS--QPKTINP-VS
DsimulansSytalpha           NDPAFWVPASVTS--TAAIQQQVSNTTE----ESAP--PTPTSPTGSLKSNTLSYCSTTSVPIARSDKHVVLAMHPSRPRVSSMNAKLDHTKIDMTLYRSHS--QPKTLNP-VS
DsecheliaSytalpha           NDPAFWVPASVTS--TAAIQQQVSNTTE----ESAP--PTPTSPTGSLKSNTLSYCSTTSVPIARSDKHVVLAMHPSRPRVSSMNAKLDHTKIDMTLYRSHS--QPKTLNP-VS
DerectaSytalpha             NDPAFWVPATVNS--TAAIQQQVSNTTE----ESAP--PTPTSPTGSLKSNSLSYCSTTSVPIARSDKHVVLAMHPSRPRVSSMNAKLDHTKIDMTLYRSHS--QPKTLNP-VS
DyakubaSytalpha             NDPAFWVPSSVTS--TAAIQQQVSNTTE----ESAP--PTPTSPTGSLKSNTLSYCSTTSVPIARSEKHVVLAMHPSRPRVSSMNAKLDHTKIDMTLYRSHS--QPKTLNP-VS
DananassaeSytalpha          NDPAFWVPASVTS--TSAIQQQVSNTTE----ESAP--PTPTSPTGSLKSNTLSYCSTTSVPIARSDKHVVLAMHPGRPRVSSMNAKLDHTKIDMTLYRSHS--QPKTINP-VT
DpseudoobscuraSytalpha      NDPAFWVPASVAS--TAAIQQQVSNTTE----ESAP--PTPTSPTGSLKSNTLSLCSTASVPIARSDKHVVLAMHPTRPRVSSMNAKLDHTKIDMTLYRSHA--QPKTLDP-AP
DpersimilisSytalpha         NDPAFWVPASVTS--TAAIQQQVSNTTE----ESAP--PTPTSPTGSLKSNTLSLCSTASVPIARSDKHVVLAMHPTRPRVSSMNAKLDHTKIDMTLYRSHA--QPKTLDP-AP
DwillistoniSytalpha         NDPAFWVPASVAT--TAAN----AVAQD----ESAP--PTPTSPTGSLKSNTLSYCSTTSVPIARSDKHVVLAMHPGRPRVSSMNAKLDHTKIDMTLYRSHS--QPKAMSP-LP
DvirilisSytalpha            ADPAFWVPASVTSS-TAAVQQQVSNTTE----ESAP--PTPTSPTGSLKSNTLSLCSTTSVPIARSDKHVVLAMHPSRPRVSSMNAKLDHTKIDMTLYRSHA--QPKPVCPAAP
DmojavensisSytalpha         ADPAFWVPASVTSS-TAAVQQQVSNTTE----DSAP--PTPTSPTGSLKSNTLSYCSSTSVPIARSDKHVVLAMHPSRPRVSSMNAKLDHTKIDMTLYRSHA--QPKTVCPSAT
DgrimshawiSytalpha          ADPAFWVPASVTSSSTAAVQQQVSNATNTTELDSAPATPTPTSPTGSLKSNTLSYCSTTSVPIARSEKHVVLAMNPTRPRVSSMNAKLDHTKIDMTLYRSNS--QPKHACPAAP
```

```
                                                                                                    300
                              |         |         |         |         |         |         |         |         |         |         |
CapitellaSytalpha          EEENLGSINFSLLYDQEQGLITVRLIQACDLVPRDFSGTADPYCRLCLLPVRRTQIQSKVHRKTLNPEFNEEFIFDANPN-EISDMSLQILLYDFDQFSRDECIGEVVQPLGSL
LgiganteaSytalpha          QEDNNGNIHFSLEYNKETSILTVHLIQAQDLVPRDCSSTLDPYCRVSLLPDRRGDLQSKIQRKTLNPIFEEPLMFELGAN-KLPSTTLEILVFDYDQFSQDECIGQLHVSLDTV
CsavignyiSytalpha1         GDDDTPMLHFSLAYNSEFEVLNVKLIQARSLATQVFSGTADPYCTVALVPG-FNPRRSKVHKKTSNPEFEECFTFSVSSD-SLEETILEVKTFDFDQFSRDECTGVMQMKMKEI
CsavignyiSytalpha2         SHKRPPQVFFTIEYDRKFNILKIFVNQARYLKPQNHASMFDPYCTVNILPG-HTEKQSQVIRRTADPLFQEHFSYEVPAH-EIPKKTVQIKFFDYDQFSRDECTGVVEQRLDEL
CintestinalisSytalpha1     AEADTPMIHFDVSYNGDLEVLNVKLIQARNLATQDFSGTSDPYCTVALVPG-FNPRRSKVHKKTSNPEFGESFVFSVSSD-NLEDKVLQVKTYDFDQFSRDECTGVMELNLKEI
CintestinalisSytalpha2     SSKRPPQVFFSIEFDKNFNILKIFVNQARYLKPRTYTSMCDPYCTVNLLPG-HTEKQSQVMRRTMDPLFQEHFSFGVPIQ-ELPKKKLQIKFFNYDQFSRDEFTGIVEQKLDEI
SpurpuratusSytalpha        ---TLGIMNFSLKYNSEMGLLTVRLIQARNLQPRDFSGTADPYCKVCVIPHASKTLQSKVHRKTVLPEFRESFVFEIPEI-EIHRQTVRVYLYDYDQFSRDECIGVVELPLAHV
BfloridaeSytalpha          --XYAGELHFALHYNKDMGILTIRLIQARDLQPREFSGTADPYFKISVLPDEPRTLQSKIHRKTLDPEFEEKFAFEIPPT-DLPNRTIRFLLFDYDQFSRDECVGQVLLPLENV
NvitripennisSytalpha       ANETRGELQMSITYDAPDGILNVKLVEARDLRARDLSETADPYAKIRLLPDRSTVKQTRIHKKTLNPEFDEDFVFQVAPNCQLAERTLEVLLYDFDASSKHRGLGYVQIPLSTV
AgambiaeSytalpha           GEDARGAIHLTLSYDPAAGILNVKLVEAQDLQPQDFSGTADPYAKIRLLPDRNNMWQTRIHKKTLNPVFDEDFVFEVRSA-TIGRRTLEILLFDFDAYSRHVCIGGVQFPLAQV
DmelanogasterSytalpha      LNEVRGNLHVSLGYDPVGGLLNVRLLEAQNLQPRQFSGTADPYAKVRLLPDKKNFWQTRIHKRTLNPVFDEQFVFEVTAG-VIDKRTVEILLYDFDAYSRHVCIGGSKLHLANL
DsimulansSytalpha          LNEVRGNLHVSLGYDPVRGLLNVRLLEAQNLQPRQFSGTADPYAKVRLLPDKKNFWQTRIHKRTLNPVFDEQFVFEVTAG-VIDKRTVEILLYDFDAYSRHVCIGGSKLHLANL
DsecheliaSytalpha          LNEVRGNLHVSLGYDPVGGLLNVRLLEAQNLQPRQFSGTADPYAKVRLLPDKKNFWQTRIHKRTLNPVFDEQFVFEVTAG-VIDKRTVEILLYDFDAYSRHVCIGGSKLHLANL
DerectaSytalpha            LNEVRGNLHVSIGYDPVGGLLNVRLLEAQNLQPRQFSGTADPYAKVRLLPDKKNFWQTRIHKRTLNPVFDEQFVFEVTAG-VIDKRTVEILLYDFDAYSRHVCIGGSKLHLANL
DyakubaSytalpha            INEVRGNLHVSIGYDPVGGLLNVRLLEAQNLQPRQFSGTADPYAKVRLLPDKKNFWQTRIHKRTLNPVFDEQFVFEVTAG-VIDKRTVEILLYDFDAYSRHVCIGGSKLHLANL
DananassaeSytalpha         INEVRGNLHLSIGYDPVGGLLNVRLLEAQNLQPRQFSGTADPYAKVRLLPDKKNFWQTRIHKKTLNPVFDEQFVFEVTAG-VIDKRTVEILLYDFDAYSRHVCIGGSKIHLASL
DpseudoobscuraSytalpha     AIEVRGNLHVGISYDPVGGLLNVRLLEAQNLQPRQFSGSADPYAKVRLLPDKKNFWQTRIHKKTLNPVFDEHFVFEVAAG-VIDKRTVEILLYDFDAYSRHVCIGGTKLHLANI
DpersimilisSytalpha        AIEVRGNLHVGISYDPVGGLLNVRLLEAQNLQPRQFSGSADPYAKVRLLPDKKNFWQTRIHKKTLNPVFDEHFVFEVAAG-VIDKRTVEILLYDFDAYSRHVCIGGTKLHLANI
DwillistoniSytalpha        ANETQGNLHVGIGYDPIGGLLNVRLLEAQNLQPRQFSGTADPYAKIRLLPDKKNFWQTRIHKKTLNPVFDENFIFEVSAG-VLDKRSIEILLYDFDAYSRHVCIGGTKLHLANL
DvirilisSytalpha           ISELRGNLHICLSYDPVGGLLNVRLLEAQNLQPRQFSGTADPYAKIRLLPDKKNFWQTRIHKKTLNPVFDEHFVFEVTAG-VIDKRSVEILLYDFDAYSRHVCIGGTKLHLANL
DmojavensisSytalpha        GNEVRGNLHVCLSYDPVGGLLNVRLLEAQNLQPRQFSGTADPYAKIRLLPDKKNFWQTRIHKKTLNPVFDENFVFEVSAG-VIDKRTVEILLYDFDAYSRHVCIGGTKLHLAQL
DgrimshawiSytalpha         LNELRGNLHVCLSYDPVGGLLNVRLLEAQNLQPRQFSGTADPYAKIRLLPDKKNFWQTRIHKKTLNPVFDEQFVFEVNAG-VIDKRSVEILLYDFDAYSRHVCIGGTKLQLATL

                                                                                        400
                              |         |         |         |         |         |         |         |         |         |         |
CapitellaSytalpha          D---LGEKVSLWKGITPR--SNSHVEEEALGDLMFSMAYLPSAERLTVVVVKGRHLQP--KDDGKTTYSPFVKVSLI-SSGKRVKKKKTSAKRNSANPVWNEALVFNMPRDSV-
LgiganteaSytalpha          D---FSEKTILWKGLMPP--DKPKDHEE-IGDIMFSMSYLQSAERLTVAVTKARNLRH--PEDGKLSLDPYTKVTLI-LSNRKTKKRKTSTSRGDCNPEWNEALVFNLPNEYL-
CsavignyiSytalpha1         DFV-MTPSIDLWRKIKPPKCSDISKSSETYGDLMLALSYLRSAEKLTVAVQKARNLRE--PESRKGLPDSYVKVALY-KGKQRLKKKKTDTVHSTCNPVFNQALTFSVPHETLQ
CsavignyiSytalpha2         N---LDSKVDLWRKIRSFE-QLGEKSPRKAGDILLRLGYLPSAEKLTVVLLKARHLKEIGNDGDSRPPDPYIRVAVW-HDGTILKKKKTSTKRRTCNPTYNQAINFAVPLDVL-
CintestinalisSytalpha1     DFV-MTPNIDLWRKMKFP---DDHETSETFGDLMLALSYLRSAEKLTVAVQKARNLKE--PESRKGLPDPYVKVALY-KGKQRIKKKKTDTIHSTCNPVFNQALTFSVPFDTLQ
CintestinalisSytalpha2     N---LDAKVDLWRKIQNVD-ETENKS-KKNGDILLRLGYLPSAEKLTVVLLKARNLLESRPEKEARLPDPYIRVTVW-HDGNILKKKKTSTKRRTCNPTYNQAINFAVPLDVL-
SpurpuratusSytalpha        D---LTEKLEVWKGIRAP--PSCPSHV--LGDLMFSLSYLPSAERLTVVILKARNLRTVSTAEGKQTADPYVKVSIF-YMSKRLKKKKTSTQHGTRSPVFNEALVFNVDTDFL-
BfloridaeSytalpha          D---LSERVELWKMIESYKIRTPEKKPD-LGDLLMTASYLPTAERLTIVILKARGLENT---PHNKRPDPYVKVSIL-YAGKRLKKKKTSTRHSTTNPVYNEALVFDVGREFM-
NvitripennisSytalpha       TDLGLEPKTLTKSVMRYG--AEGRFRAPPLGELMVSLSYQPTAERLTVIVIRARNLPIND-ETGTATFEPYVQVNIV-REDKSLKKKKTSIRREGTSPVWSESLNFDLTPDVL-
AgambiaeSytalpha           C---LAERIDIWRPLLPY--TEQDNKQD-LGDLMVSLSYQPAAEKLTVVIIKARNLRVID--GTRNSSDPYVKVCLYNADGRRLKKRKTTVARNTTAPVYNEALTFDISRETL-
DmelanogasterSytalpha      D---LSEQLKLWTPLSSA--SAQDMKVD-LGDIMVSLAYLPSAERLMVVLIKARNLRIVD--DARNSSDPYVKVTLLGPGGKKIKKRKTGVQRGTLNPVYNEALAFDVAKETL-
DsimulansSytalpha          D---LSEQLKLWTPLSSA--SAQDMKVD-LGDIMVSLAYLPSAERLMVVLIKARNLRIVD--DARNSSDPYVKVTLLGPGGKKIKKRKTGVQRGTLNPVYNEALAFDVAKETL-
DsecheliaSytalpha          D---LSEQLKLWTPLSSA--SAQDMKVD-LGDIMVSLAYLPSAERLMVVLIKARNLRIVD--DARNSSDPYVKVTLLGPGGKKIKKRKTGVQRGTLNPVYNEALAFDVAKETL-
DerectaSytalpha            D---LSEQLKLWTPLSSA--SAQDMKVD-LGDIMVSLAYLPSAERLMVVLIKARNLRIVD--DARNSSDPYVKVTLLGPGGKKIKKRKTGVQRGTLNPVYNEALAFDVAKETL-
DyakubaSytalpha            D---LSEQVKLWTPLSSA--SAQDMKVD-LGDIMVSLAYLPSAERLMVVLIKARNLRIVD--DARNSSDPYVKVTLLGPGGKKIKKRKTGVQRGTLNPVYNEALAFDVAKETL-
DananassaeSytalpha         D---LSEQVKLWTPLSSA--SAQDMKVD-LGDLMVSLAYLPSAERLMVVLIKARNLRIVD--DARNSSDPYVKVTLLGPGGKKMKKRKTGVQRGTLNPVYNEALAFDVGKETL-
DpseudoobscuraSytalpha     D---LSEQLQLWTPLSSA--SAQDMKVD-LGDIMVSLAYLPSAERLMVVLIKARNLRIVD--DARNSSDPYVKVTLLGPVGKKMKKRKTGVQRSTVNPVYNEALAFDVNKETL-
DpersimilisSytalpha        D---LSEQLQLWTPLSSA--SAQDMKVD-LGDIMVSLGLPSLGRRLMVVLIKARNLRIVD--DARTPSDPYVX----------------------------------------
DwillistoniSytalpha        D---LSEQLKLWTPLTSA--SAQDMKVD-LGDIMVSLAYLPSAERLMVVLIKARNLRIVD--DSRNSSDPYIKVSLLGPGGKKMKKRKTGVQRNTVNPVYNEALAFDVAKETL-
DvirilisSytalpha           D---LSEQLKLWTPLTSA--SAQDMKVD-LGDIMVSLAYLPSAERLMVVLIKARNLRIVD--DARNSSDPYVKVTLLGPGGKKLKKRKTGVQRNTVNPVYNEALAFDVGKETL-
DmojavensisSytalpha        D---LSEQLKLWTPLTSA--SAQDMKMD-LGDIMVSLAYLPSAERLMVVLIKARNLRIVD--DARNSSDPYVKVSLLGPGGKKLKKRKTGVQRNTVNPVYNEALAFDVGKETL-
DgrimshawiSytalpha         D---LSEQLSLWTPLTSA--SAQDMKVD-LGDIMVSLAYLPSAERLMVVLIKARNLRIVD--DARNSSDPYVKVSLLGPSGKKIKKRKTGVQRNTVNPVYNEALAFDVGKETL-

                                                                             500
                              |         |         |         |         |         |         |         |         |         |         |
CapitellaSytalpha          -GDVQVEFVVYTDNLLGNN-----EALGKVIVGSKSS-GEEQAHWKDVL-SS-KKAMAMWHRIRAVDSTS----------------------------------------
LgiganteaSytalpha          -DTICIDVSVFHENKIGND-----ELLGRVRLSGDSD-GDENIHWQDLV-SS-KCARARWHHLS----------------------------------------------
CsavignyiSytalpha1         SPDARLICHVVHDFKLGYN-----EYLGQLDLGPNSTDSEESNHWSEMMTQHINRPIARWHRLKQSTTPAQRLSTDENMSGSTHGRRKSFAVPEALQLHGLAELGRKSFS
CsavignyiSytalpha2         -PQVEMQFHVVHETGVKLNRLDRKEAIGYLEIGPHS-SGDEFEHWRDLMS---NKPQARWHHLTDVTESDSKPASGSSLTNLDLTDTGRGTI------------------
CintestinalisSytalpha1     AADTKLICHVVHDFKLGYN-----EDLGQVEIGPNCMDSEERRHWSEMMAQHINRPIARWHHLKQPSPPTQRLSTDENLPSSS-GRRKSFAVPEALQLHGLADVSRRSFS
CintestinalisSytalpha2     -PQVEIQFHVVNEIGVKLNRAERKEAIGYLEIGPHS-NGDEFEHWRDLMS---NKPQARWHHLVDITDVDIKP-TGSSISHLDL-DSGISQDKL----------------
SpurpuratusSytalpha        -QHLTIEFQVIHENRFGPN-----EVLGKAVVGPGTG-GEELAHWNDMIRSG-NKPVASWHCLLP---------------------------------------------
BfloridaeSytalpha          -DHVYIELVIVHENRFGQN-----QGMGKVVLSAESE-GEELEHWKSLAAS--RKGTARWHCLQPLQPD-----------------------------------------
NvitripennisSytalpha       -AECILDFSIFR----ANG-----ELLARCEVSE-----LRQRELFHRVLAG-AGASAQWLPLSEPEKLAHDGQPELQHQLSKSS-------------------------
AgambiaeSytalpha           -RDCSIEFQVLHDSLLGKN-----ETLGRATVGSGPEFRQENRKFFDELFRT-RAATAQWISLCE---LKLKSDTPSKKK------------------------------
DmelanogasterSytalpha      -KNCVLEFTVVHDGLLGSS-----EILGRTLIGNSPEVRTEEKIFFEEVFRA-KNATAQWVPLQEP-ANNLATSA--KSSKN----------------------------
DsimulansSytalpha          -KNCVLEFTVVHDGLLGSS-----EILGRTLIGNSPEVRTEEKIFFEEVFRA-KNATAQWVPLQEP-ANNLATVA--KSSKN----------------------------
DsecheliaSytalpha          -KNCVLEFTVVHDGLLGSS-----EILGRTLIGNSPEVRTEEKIFFEEVFRS-KNATAQWVPLQEP-ANNLATVA--KSSKN----------------------------
DerectaSytalpha            -KNCVLEFTVVHDGLLGSS-----EILGRTLIGNSPEVRTEEKIFFEEVFRA-KNATAQWVPLQEP-ANNLATAA--KSSKN----------------------------
DyakubaSytalpha            -KNCVLEFAVVHDGLLGSS-----EILGRTLIGNSPEVRTEEKIFFEEVFRA-KNATAQWVPLQEP-ANNLATAA--KSSKN----------------------------
DananassaeSytalpha         -KNCVLEFTVVHDGLLGSS-----EILGRAIIGNSPEVRTEEKIFFEEIFRA-KNATAQWVPLQEP-ATNLANTA--KSTTNRN--------------------------
DpseudoobscuraSytalpha     -KNCVLEFTVVHDGLLGSS-----EILGRTLIGNSSEVRTEEKIFFEEMFRA-KNATAQWVPLQEP-ATNLANAA--KSTTNKN--------------------------
DpersimilisSytalpha        --------------------------------------------------------------------------------------------------------------
DwillistoniSytalpha        -KNCVLEFTVVHDGLLGSS-----EILGRTLIGNSPDVRCEEKMFFDEMFRA-KNATAQWVPLQEP-ANNLANTATAKTTTNKN--------------------------
DvirilisSytalpha           -KNCVLEFNVVHDGLLGSS-----EILGRALVGNSPEVQHDEKIFFEEMFRA-KNATAQWVSLQEP-ANNLSATA--KTTTKN---------------------------
DmojavensisSytalpha        -KNCVLEFNVVHDGLLGSS-----EILGRALIGNSPEVQHEEKIFFEEMFRA-KNATAQWVPLQEPTANNLGATA--KATTKN---------------------------
DgrimshawiSytalpha         -KNCLLEFTVIHDGVLGSN-----EILGRALIGSSAEVPHDEKIFFEEMFRA-KNATAQWVPLHEP-ANNLGATA--KTTTKH---------------------------
```
