# Supplementary material for: A manual collection of Syt, Esyt, Rph3a, Rph3al, Doc2, and Dblc2 genes from 46 metazoan genomes - an open access resource for neuroscience and evolutionary biology
Source: BMC Genomics. 2010 Jan 15;11:37. doi: 10.1186/1471-2164-11-37 (PMC2823689; doi:10.1186/1471-2164-11-37)
Supplement: Additional file 37 — Alignment of the Syt18 sequences. Amino acid position is marked every hundred amino acids approximately, at the top of each page of the alignment. Intron position and phase is indicated with a coloured bar between amino acids. Black bars indicate phase 0 introns. Red bars indicate phase +1 introns. X residues indicate where a portion of sequence is missing. [file 1471-2164-11-37-S37.PDF]

```
                                                                                                                      100
                             |         |         |         |         |         |         |         |         |         |         |
LgiganteaSyt18          -----------------------------------------------------------------------------------------------------------------------
SpurpuratusSyt18        ------------------------------MRKLKVTFVSIIFFKFTVTLIFAVSTALAVSISFGLLIFICCCMRARKTQKR-------------------------------------
BfloridaeSyt18          -------MEINVHFSSFLRLGSAGCTKTKVSVVPVECFRSRNVLATRQAYAQTLIAVGAAVLCFLLLLGCTICWRLRAWNR--------------------------------------
Trubripessyt18          MPYHDEEYPGQPLWQSVVLFCCKGMIEGIMVILFFWLLVQVLFTKQLEVHLQVLLLVGLIVFCLCLVLGCILCWRNSQMYPAKVKDPV-----TSAPVSAEPVTLSKTPTPSPTAAAAR
Tnigroviridissyt18      MPYQDEEYPGQPLWQSVTLFFCKGMIEGIMVILFFWLLVQVLFTKQLEVHLQVLLLVGLVVFCLCLLLGCILCWRNRQAYPAKVKDPV-----TSAAAPAELVTLSKTPPPLPTPALSR
Gaculeatussyt18a        -----------------------------------------MSQYALAVHLQILLAVGLAVVCCCLVLSCVFCCRRRKSVSSEDKEAVF-LSPHRVERVTVTLTP--SPCTQPVKQQY-
Gaculeatussyt18b        MPYHDEEYPGQPLWQSVLLFCCKGMIEGIMIILFFWLLVQVLFTKQLEVHLQILLLVGLIVFCLCLVLGCILCCRTSRICAVRDKDPVSSDPVTSAPAHAEPVTFAQIPPPSASMTASA
Olatipessyt18a          -----------------------------------------MSQDSPEVNLQILLAVGLAVFFYCLVLGCIFWCCRQKTVSLEDKEAAF-LPPHLASGVAASSSSH-SPCTQHVKQQY-
Olatipessyt18b          MPYQDEECPDQPVWQSVLLFCCKGMIEGIMFILFFWLLVQVLFTKQLEVHLQILLLVGLIVFCLSLVLGCVVCWRKSQFFSGKKQTP---------ATASAPVDLPESSHPA--VPASQ
Dreriosyt18a            ------------------------------------------------XHLQILLAVGLAVLCFCLLIGCIFCYYHSKLHPKDDKEAELSVPPPSADHVTITLSPSPSIITQPIKQQY-
Dreriosyt18b            MATEEEENSGQPLWQSVLLFGCKGMIEGIIVLLFFWLLVQVLFTKQLEVHLQILLSVGLVVLCCSLFLGCIICWLKTREKPPENKKAVRT---SRPPDSVDEVTVSLSPSPSSGTTFTK
Xtropicalissyt18        ------------------------------------------------XYLQVLLGAALALFCFCLLLGCAICWQQRSRSQ--------------------------------------
Acarolinensissyt18      ------------------------------------------------XHLQVFLGVGLALLCFCLLLGCAICWHQRKKSRC-------------------------------------
MdomesticaSyt18         -----------------------------------------------------------------------------------------------------------------------

                                                                                                            200
                             |         |         |         |         |         |         |         |         |         |         |
LgiganteaSyt18          ----------------------XISDKRYVLLNNQE-VEIEGYKALDIISRKSQIACTSTAPDAFGKSDIYADSKPPSYPRPIIGIIHGAKLSEARSFEDISFDPRSEPI--------K
SpurpuratusSyt18        QKRREKLEHALAITPLKADFFRKRDEDCLKTTPVPR-QDPIEDNPFSDVQTDDQGVETPNDQPDNYGRSQSWAPA-PSTKLIAVDPQRRQTLAVLSLTPSSSDSSLASCSS-------E
BfloridaeSyt18          ---RAKARQPSERLSVSFQGTPFGPTSSSSIKYTLD-PADLRTGPTFSSVTSSPFLPPSTPDPNEARVKRFSCPDPPSAELLG----RRDNLRRSKTLESVEDGPIEE-----------
Trubripessyt18          RRYEELDGDTLEYPSTFSSSVSSEGELVTQPLSNRA---ASEQKEQPKSYFSLRRFSTPALTSPLYRPIDPGHASLPSFPRFGL-SKTCKALKRRCTVTGSTISYNEHSRLT-----SP
Tnigroviridissyt18      QQYQELDGDILDYPSTFTSSVSSQVEFVAQPDTNRAPAAAAEQKEQPKSYFSLRRFRTPPLTSPLYKPIDPGHASLPSFPRFGL-SKTCKALKRRCTVTGSTISHNEHSRLT-----SP
Gaculeatussyt18a        ---EELDGDVLEFPSSKSSSPPSEDDLTSLRFDRSP-TGSAELVPSPRSSFPMRRLSTPAVPCSANKSPRHGRASLPSLTKLGLASMSRRAMGRRSTVSGESLLYGESSGLTVAGSPGQ
Gaculeatussyt18b        HKYGEQDGDIVEYPSTFPSPTPSEREFASLSFSKRA-RAGSEQKEQPKSYFSPRRLSTPPLISPLYKPIHHSHTSLPSFPKLGLMSKTYKALQRRCMVTGGKLSYNEHSRLT-----SP
Olatipessyt18a          ---EELDGDVLEFVASS----PSGDDLSCAT---DP-KRSAGRVEAPLSFFPLRRLSSPAVPCSPRKTLRHGRASLPSLTKISLVSKSRYVMGRLSTGNSEIFAHFKGSRLT-----GQ
Olatipessyt18b          PLCQELRRDTLDYPSTFSSPARSQGEISSFPFGNQA-KVATEEHERAAPYFSLRRLSSPLLPGPAYKPMHPGRASLPSLPKLGLLTKN--APQRRCTVSGDCRSHTERSRLT-----SP
Dreriosyt18a            ---EELDGDVLDFPSINSS---SEDDISM-----------TSLPGPQKSRFSLRRLSSPVITCKLGKPAIRGRSSLPTIPKLSLSVKSHSRVDDDSNSENSKIKVYNDR----------
Dreriosyt18b            LQYEELDGEVLDYPCTFESSTPSDEEFTVFS-----------EVNKQKSCFQMRRLSSPTVATSLYKPMSRGRSSLPSLPRLGLLSKTRKVVERHCAVTGDNYSFSEHRRLTTPNPRSP
Xtropicalissyt18        ---SIQSLDDTDKELAEISVRSAAVPIPTSYQEIDD-MDE--------DFIAQSGDNVEDAPIRSIH----SRPSRHGLHRISS--KTKRILHRRSTLAVDYSQEGDSVKL--------
Acarolinensissyt18      DEAKAQAADRTVMDLGLVLPSRTITPVAIQQQYVEI-EGELLESPSPTAVIDSPGSSGSESPPRNLHRGRASLPSIPISQKLSLPVKAQRGRERRCTISGDESSLLSRPIL-------G
MdomesticaSyt18         -----------------------------------------------------------------------------------------------------------------------

                                                                                300
                             |         |         |         |         |         |         |         |         |         |         |
LgiganteaSyt18          RCTSLTTLYKDVPETNNT-----------DSLTFMTLFINLDTDHNCLTISLDKLYNVLK-IDLYNDIHIWLRVFPDHPEGFHSQPVHAAKNLEF-IDIFRLKDQTVENLIKSTFRLTV
SpurpuratusSyt18        NDLEQLGRESEWTVYQGG-----------NEKPVVHFSLYFSTQDSTLFVCLKSVSGLARKYYPGCSSFLKVSLLPKYRDGFRTDIVRKSLNPHF-NESFQFCKVTLAEAQGSFLKIKL
BfloridaeSyt18          -SSSVEIDPPRGPLPTVS-----------GGKTTLHFSLFHSAFDHTLTVNILGVSNLPRTFSMESAAFVKVWLLPSHQEAVQTTIRRKSFNPQF-NESFKFKSMEREDIDNVHLRFAV
Trubripessyt18          AVACPLMPEEPIPLAPLNYGSSISCQQPLSPKPCLHFTMAFSPEERTLAVSVLRLSGTPHRL---EDVSVLGRLPPLYPCPLQAS-AQKSLSPEADSLLLLLKVGSVEELQRCVLKLTV
Tnigroviridissyt18      SMARPLPPEEPIPLLPLNYRSTVSCQQPLSPKPCLHFTLAFSPEERTLAVTVLGLSGTSHGL---EDVSVLGRLPPLYPCPLQAS-AHKSPSPEAPSLLLLLKVSSVQELQRCVLKLTV
Gaculeatussyt18a        HGEPRLSQYGSNSLSISS-----------KPAALLHFSLLFSSACGSLIVNVLGVSGASRR---RTGVFVRASLPPLCTTPQQIAYRRRSLSPDLHSQSFVLQVGAVEDLRGCTLRLAV
Gaculeatussyt18b        SSVFASSPEEPIPLAPLSYGSGASCQMPVSSKPCLHFTMAFSPEQQTLAVTVLDLTGTTHRL---QDASVLGSLPPLYPCPVQAS-TQ---SPESRSLVLLLRVSSVMELQKCDLRIEV
Olatipessyt18a          QGEPCSTQYGSGSLSIS--------------APLLHFSLLFSSASGTLVINILGLSG--HR---RSGVIVRVSLPPICPSAQQITLRRRSLSPDLQGQSRVLQVGSVEELRACTLRLAV
Olatipessyt18b          RAISSSIAENPIALTPLSYGSHTNLS---SANPCLHFTLAFNPQQQILTVTILNLTETSHRL---ENVSVLGSLLPLHPSPTQMS-SHSSLSRDASRLELLLKVRSMKELQRCELRLAL
Dreriosyt18a            -------HYGSSRSTPS-----------------ISFTLLYSSSNSRLTVSVLGVFRGSRR---LSGMQVTACLPPLCPETLQ-AGRKQSLSAECPAQVFSLQVWSVQELQTCTLRLSI
Dreriosyt18b            CTLSPLQTQGLLQEEPLFSNYGSNVYSE-TSDPFLHFTLTFSSMQNTLTISSVSITSVVHCL---EEMIIQIRLPPLCPGPLTLS-AQDYNLSSGLYKTLVVSVGSLEGLKSCILRLAV
Xtropicalissyt18        VRMSASRTDPSGLSKTKH-----------KSHPFLHFTLHYSVEEETLTVTVTGLSNLPKKFRHKRQSLVRVYLMPGFIEP----LPAQNEGPEQ-GQKFLFCKYSSEQLKELTLRLTV
Acarolinensissyt18      TSLPPSYTIPRGLSGATT-----------KPRPHLHFTLFYSEAEALLTVTVVGVSRLSKGLRSSRNSYVKVYLLPKFVEPQRTSLCRKSLNPEF-HEQFHFGRYSLEELRSLTLRFTV
MdomesticaSyt18         -----------------------------------------------------------------------------------------------------------------------
```

```
                                                                                  400
                                |         |         |         |         |         |         |         |         |         |         |
LgiganteaSyt18        LGKDSKKKSHRESIICEGFVNGRDIDWK-----------SAQNNEIKVYFKRKWMKKMSTSNKFLESDLGELFVLLQYQSMAKRMKVLIRKANNLPKSDK---LIGVPGHSVVINLYKD
SpurpuratusSyt18      YVRE--KWNRKDRFSGEVFAECHQLLTKGDSLLELQRTFSIKRTKLPKKNLQRMRSKSTSEETSNRPYLGDLFVSLQYQPLADRLKVMIRKAENLLVDSA---LPGNGDVYVTVRLLKQ
BfloridaeSyt18        YVKQ--LWDKKDGFVGEVLFPGAEWD-RRDVPTTFSRELKSSKTKGEK--LRKISSSIDLEELDEKAKSCELFVLLQYQAMSNRMKVLIRKAENLTKKKMSLPVPGTADHYIIVRLLHR
Trubripessyt18        YTQK--PPSLRSSALGELEAECGGREWRGENPFFFRKELNQKKWKLQEQDQSSNNSATCKA---SSSPP-QIFVLLQYQTLTHRIKATLLRADNLDTL-AG--TSAPAEFQVLVALHHE
Tnigroviridissyt18    YTQK--NPSLKSTVLGDLETECRGRDWRVQKPFLFKKELNQIKWKLQE-DQVSDNSA--RT---SSSPP-QIFVLLQYQTLAHRIKAGLLQAHNLDRL-HG--TSAPAELQVLVTLRHE
Gaculeatussyt18a      HSRD--FSGLREAALGVVELPCEQMDWEPDTTITYTRQLSPTKSKLKKSVSSQETLGRRKSSVCAPRVLGQLFVLLQYQTPAQRVKVMVRKAENLTKLTR---IPGAADHYVVINLRQD
Gaculeatussyt18b      YTRE--AP----TALGELEVECGGRDWRAEHPFHLAKELNINKWKLKKIGGMEMNVARPTLILFACISL-LIFILRQYQPLVRHIKVKVFRAYYLVHLVHS--LAATVHFYAQRTTFGF
Olatipessyt18a        YSRD--FSGLREAALGWLELPCEPVDWESEITNTYSKQLTATKGKLKKSVSSQEWLGRSKSSVGAPVTLGKVLILLQYQPAAQRLKVMVRKAENLTKLTR---IPGAPDHCVVIKLHQN
Olatipessyt18b        YTRG--VQSPRDTPLGEVGLECGNIDWISEHPLQFMKALKRNQGNYEKX----------------------------------------------------------------------
Dreriosyt18a          SSRD--FSGLRETPLGELEMSCAEIQWESDHTFSLTRQLNPARRKLRKSQSCQAAVG---APVSLLRSLGQILILLQYQSLAHRIKVMVRKAQNLPKLSR---MPGTPDHSVIINLLQD
Dreriosyt18b          CKQN--NP--KTSLFEELEVCCSRLDWTLDRPVNCVRELRPVVDKTKRX----------------------------------------------------------------------
Xtropicalissyt18      FAQE--KQSLKERFIGEVLFPGPEIDWNSQGSCVYTKELSGIKAKLKK--SMSTTDVIS-PTTAQPKSLGQIFILLQYQCSANRIKVMVQKAENLSKRAR---IHGPADHFVSIRLIKE
Acarolinensissyt18    YAKE--FHNLKDSFLGEVMFPCSQATWNPRVPSVYTQELSTTKTKLKK--SLSSQDISSSPSFSQPKSMGQLFLLLQYQALANRIKVLVRKAENLGRLTR---IPGTPDHYVVIQLYHD
MdomesticaSyt18       ------------------------------------------------------------------------------------------------------------XHSVIIELYQA

                                                                        500
                                |         |         |         |         |         |         |         |         |         |         |
LgiganteaSyt18        GEIVSSRETQVQGGLHPVWNQPFLFTIPNE--HHDSYNLEFIIKRNRRYTKDNIIGHVLVGPGAPKSGGDQWQEAMKPRGLECALWHNITPIFTYRN--------------------
SpurpuratusSyt18      SEMVGEYSTQRRSGTSPIWNQPFLFHPKSDTIQHHSILL--AIFRGRRLATDVMVGKVEVGIGAGETGVAHWTEMLQPLGIEVAKWHRIKPPFS-----------------------
BfloridaeSyt18        GNVKETKETKSASGSSPVWNQPFLFDIPSDQVED--YSLEFVVMRGRIYTRDGVIGSVLIGPAAPPSGVSHWNETLQPRARESARWHTIQPASGKFSPKRKLSKESYTKAEFNFSSN
Trubripessyt18        GAVISSRETKGAS-----WNSSFVFDLPPGDISKLPLMLEFVIVQHQAPAQRKVLGRVQIGAGAADAGRAHWRDVC-ILHVEQPRWHVVQPEVL-----------------------
Tnigroviridissyt18    GSVISSRETRGASCSS--WNSSFLFDLPPGDINTLQLMIEFVIVQHQAHSQGQVLGRVQIGAEAAGPGRAHWRDMC-ILHVEQTRWHAVQPETL-----------------------
Gaculeatussyt18a      GKVIVTKETKGASGPNPVWNAPFLFDLPSGDITQLPLVLEFIVMQGRLYTKSSVLGRVSIGGDASGAGQAHWEEMCSRGQTETARWHTIQSDVP-----------------------
Gaculeatussyt18b      NTYVTV------------WNTSVLFDPPPGDINQLPLMLEFIIVQX-----------------------------------------------------------------------
Olatipessyt18a        ANVISSKETKGVSGPNPIWNAPFLFDLPSGDITQLPLTLEFVIMQGRLYTRGSVLGRVLIGSNASEVGQGHWREMCSLQQTETTHWHTIQSEAV-----------------------
Olatipessyt18b        ---------------------------------------------------------------------------------------------------------------------
Dreriosyt18a          GTVISTKETKCSSGLNPVWNAPFLFDLPAGDVSTL--MLEFIIMQGRLYAKSCVLGRVLIGCEVSEAGNQHWREMRNTPQVETACWHLIQQDTP-----------------------
Dreriosyt18b          ---------------------------------------------------------------------------------------------------------------------
Xtropicalissyt18      NVVKERKETRTVTGSSPVWNAPFLFDTPVEALEEDTIALEFVVMQGRAYNRARTLGRVRIGAGGSKDGLAHWKEMQNKEPKECARWHTLQPDV------------------------
Acarolinensissyt18    GKVLDTKETKSVAGCNPVWNTPFLFSIPAGDIQEQQLALEFTIMQARLYTRSCLVGRVLIGPNAPEMGQVHWKEMCSRGNVESARWHSIQPPAFQLSP-------------------
MdomesticaSyt18       GQLLDSRETQAVAGCSPTWNAPFLFSLPAGDLREQNLFLQFTVTQSHLLTRTLTLGWVRIGPEASAAGRAHWWYMCQHGLQESARWHLLCP--GKPDPPQSATPGSGIRNS------
```
