# Supplementary material for: A manual collection of Syt, Esyt, Rph3a, Rph3al, Doc2, and Dblc2 genes from 46 metazoan genomes - an open access resource for neuroscience and evolutionary biology
Source: BMC Genomics. 2010 Jan 15;11:37. doi: 10.1186/1471-2164-11-37 (PMC2823689; doi:10.1186/1471-2164-11-37)
Supplement: Additional file 40 — Alignment of the N. vectensis Syt sequences. Amino acid position is marked every hundred amino acids approximately, at the top of each page of the alignment. Splice variants are included and highlighted with black dots where they differ. TM sequences are highlighted in blue. Intron position and phase is indicated with a coloured bar between amino acids. Black bars indicate phase 0 introns. Red bars indicate phase +1 introns. Blue bars indicate phase +2 introns. X residues indicate where a portion of sequence is missing. [file 1471-2164-11-37-S40.PDF]

```
                                                                                                   100
                             |         |         |         |         |         |         |         |         |         |         |         |
NvectensisSyt1       --------------------------------------------------------------------------------------------------------------------------
NvectensisSyt7       --------------------------------------------------------------------------------------------------------------------------
NvectensisSyt21      XALVAGLATGLPVVLLVFIYVAYCCCKRKQKDELLREPRLWRRDNRSSHDLFIPPYMPGIVQRFVQHSSKNSSETTSTNTSPVDDKHFFSEGSDPYRTAPDDMNGPDRDRTGSIKALQVPAP
NvectensisSyt24      --------------------------------------------------------------------------------------------------------------------------
NvectensisSyt25      --------------------------------------------------------------------------------------------------------------------------
NvectensisSyt26      --------------------------------------------------------------------------------------------------------------------------
NvectensisSyt27      --------------------------------------------------------------------------------------------------------------------------
NvectensisSyt28      --------------------------------------------------------------------------------------------------------------------------
NvectensisSyt29      ----------------------------------------------------------------MPTAPKRTRV-----PPPRVRKAWPKARGKPGGGEPGSYVVYQDESRMGLESVPVQPE
NvectensisSyt30      --------------------------------------------------------------------------------------------------------------------------
NvectensisSyt31      --------------------------------------------------------------------------------------------------------------------------
NvectensisSyt32      --------------------------------------------------------------------------------------------------------------------------
NvectensisSyt33      --------------------------------------------------------------------------------------------------------------------------
NvectensisSyt34var1  -------------------------------------------------------------------------------------------------------------------MFQSVSA
NvectensisSyt34var2  -------------------------------------------------------------------------------------------------------------------MFQSVSA
NvectensisSyt35      --------------------------------------------------------------------------------------------------------------------------
NvectensisSyt36      --------------------------------------------------------------------------------------------------------------------------
NvectensisSyt37      ----------------------------------------------------------------------------------------------MALAYFIRVFCFSRRQTTTVDGASDPGY
NvectensisSyt38      ---------------------------------------MPSEFGLTWCSLLLVVTSHRVMAVEIALNPRKVLIVVLLVVFSVSGKDIHRAQNPPKDDKFSGVPLPAIILLSVLGVCLLLVA
NvectensisSyt39var1  --------------------------------------------------------------------------------------------------------------------------
NvectensisSyt39var2  --------------------------------------------------------------------------------------------------------------------------
NvectensisSyt40      ---------------------------------------------------------------------------------------------------------MTDTASLGIVLGAVLGF
NvectensisSyt41      ---------------------------------------------------------MGLPLKFQDFDPRRSKQ----NTSYVSRMIWQNTGVTCVLGLLVFPVLVGVAIGYLLVLLGVTTY
NvectensisSyt42      --------------------------------------------------------------------------------------------------------------------------
NvectensisSyt43      --------------------------------------------------------------------------------------------------------------------------

                                                                                     200
                             |         |         |         |         |         |         |         |         |         |         |         |
NvectensisSyt1       --------------------------MVRPTLNLDVTKPTLPIWIIAVVGIGAAVIVIFCIYCCCCRKCGKKKKKDDKRGGKERVDFRAVQIGASYQEKVQ-------PSMDELD-------
NvectensisSyt7       -------------------------MVVLMVASGEVRHQNGEITWVIPAIASLVSLSMFIILCSWCRKCWKTSKAQETEIGTETFDSSSESLASTPKQKYQSLYTGKKPVGSTYDPIYSHRS
NvectensisSyt21      RRSSLMQPSTSSGDSSESTYEQATPKKGVGPRSPRSLPKDSRSFEFVRPIPVKPLITITRAESLSKDDMNARMESTNYDPESSRQTYSDSQVEARRYREGSSRSLDNIALDRQDSERRGSTD
NvectensisSyt24      --------------------------------MHDEFSFADSFTFAAITGCVAGCLVGVSLLVLCKTVCKGYLSSKLHWCFQTKQDKSEESSSEKSDAIPDAVASSTPYSSPSLCRGGKNTS
NvectensisSyt25      ------------------------------------XCTLRALPKVSLWGRPQSTLDPKACINLCLSGIMQTTKKEANAKSLEDVHHQRLQSFGTRKAPPGKLYTGIFTLNSGKTGFRQYRD
NvectensisSyt26      --------------------------------------------------------------------------------MLNLSEQAQIGIVSGAAGVLVLMVICMVCKLRRLNNTVYNDM
NvectensisSyt27      -------------------------------------------------------------------------------XCRSFCGVDLLPAHPFPTGGEVRHVRVQPLLIRRSVPDSPDSA
NvectensisSyt28      ------------------------------------------------------MPSVWVLIILGVTCAIILSAFAGLLITTVLHYRRSRRDADLATNKPELPVIKWPSLRVGALTAEYAQS
NvectensisSyt29      TRRPIRHPTDVYPPVRENDKEDDVLLESLDMDYNSSGVMPPEEMVERRGS--LEFLLHFYQLSSTLTLTMVRMSDLPRRPDTGHPPDPIVELELKYGHEGKVKSESYQNTCNPHMYES--FR
NvectensisSyt30      -----------------------------XPVVAIAVSVSAGVFLVAVIALVCVYKRRKRDEKYKQYSQVAKNTSLQLPPCNVRTEKQIVNEGSKGLPLRDTLGVKMLSASEESLSPAVSSD
NvectensisSyt31      --------------------------------------------------------------------------------------------------------------XKYEPSEQRRSS
NvectensisSyt32      ------------------------------------------MLSVDISTPIPTSPQPVTGWVFVVVIATGIFCGVLMAFGACALCRWRRRRMTKYREITDFF--EPIPRMASSPSPLYKSL
NvectensisSyt33      -------------------------MDTKSYQNLTLEGFQNPHYDTAESTSSGSDVKLKVAIGFGVVCAAVLIAVLIVLVYKIIVLRAITRKSQRPPERKIFHRGPSLKERDFSRAPQQRRH
NvectensisSyt34var1  HFVIFMVFPLIVSVPTPAIVFMAVIGSCCLLLLILYQCLKAGLFFKTEPPERPNEKDYAELGEAYATETDDDYLVGVSSKGTSRRSSFNTSGSKSDGELAL-------GDSASVCGYGPYSD
NvectensisSyt34var2  HFVIFMVFPLIVSVPTPAIVFMAVIGSCCLLLLILYQCLKAGLFFKTEPPERPNEKDYAELGEAYATETDDDYLVGVSSKGTSRRSSFNTSGSKSDGELAL-------GDSASVCGYGPYSD
NvectensisSyt35      -----------------------------------------MAFGYVPAISFLALILLLLVGLLGMYYCLTHNLCPCTQTEADKVAYEELNIGEETDEAEDLARSPRPVKKTPQIASDSEVD
NvectensisSyt36      ------XEYLVCIDYDTLHEFASENNKMSNPTFALWRCCLTGETKPKPKKKPTDEKKQNGGVEIAHEAKQDDCYVENKDIEYSKADDPTTPNEDRGSHDGV-ESGYDNSELDSSAAPSEVDA
NvectensisSyt37      HVAAEADKDLEYFDEEESHELNS-NRTSDGPFSFLGKRNESNIMLQVPSPTAFRRRYTLATASLGLTQGLSTSSLPNGRSLTGRASTRSAGGETYSDDETMTRSNINRNDLISQGIQRTRNP
NvectensisSyt38      ILYKLLSLDRCCHGDDDEKKGSKVDGAKYAELGQVDQPSDAEDEEEDLGGGYRPLTASSPSHTLNPGFPCAPGGRRLGKPGRSASMVTPRPIDPSYFLSVNNTPAAAKTSSALDVSRFRSEL
NvectensisSyt39var1  --------------------------------------------------------------------------------------------------------------------------
NvectensisSyt39var2  --------------------------------------------------------------------------------------------------------------------------
NvectensisSyt40      LFGAVAVCVSFLCYRRHAKGSYDTHDVDGRRRTIEYYSGTPQGYLPPAHTRPYPVQPNIYRLTKKSEEEATECEDGSREDDGERVRITDPYDSSVVEELAEGETDRDTIADLDTSTP---TK
NvectensisSyt41      RKVQARGKQEAIDREEYQKIKVVLGNEVIDSSFPDKVSMNVQDGTVRAPPEHVPHKYYYHRLINQTSNTNGYVKAHG-DDESAGSEDEDNKEKHAYLRDSRDESDSSLHASMEDNADR--TD
NvectensisSyt42      -------------------------------------MLTDAFLQAPIDQVMPVKSSVVLIIVVGVSTALASAFFIVFCKCIWVWWKYKRSRQPVAKDYETYAITHHTKWPAEYTYQTYENV
NvectensisSyt43      -------------------MENSVIHYVEIAVSVAALLFFILLAILCLLYIRKHYQERKRPKLLKRPSQYRYISPNEPPEFIIPPYSGGLLDEEAFEEKEDDNASHTSERDKLSVTGTTQVQ
```

```
                                                                   300
NvectensisSyt1        -YNSEDYHS-----------------------------------DLSSGVKIGRINFTLDYSFTDNTLTVGIIRAEDIPAKDF-SG-SSDPYVKIMLLPDKKKKYETK---VHRKTLNPVFN
NvectensisSyt7        GYDSPDYMRSTLKFNRPPSQEFEFQTPQLNISKVEVPPAPPPPPDASLKEQLGKIFFSLTYSSDSVLTLKVLKAQGLPAKDF-SG-TSDPFVKIMLLPDKKHKLETR---VKRKNLNPVWN
NvectensisSyt21       RSLALRPKSTPIRHHTQSEPTAAKIPINISSQVIKSKRGSKGKIAHAPVGSLGRIHVSLQYKKTTCDLFVKILQCRDLPAKDLRRNTSSPYVR-IYALPSRRHNHRTN---IVTNNLNPTFN
NvectensisSyt24       QGNDDD-------------------------------------DAVPISRPCKLQFSLYYNFHDMRLLVHIICALNIPTRWY-GK-APDSQVRLQLLPDTEHFYQTE---IRVNEAQPIFN
NvectensisSyt25       -FDATRRDIPITSFLHTKDTLQRPTATAPGSIEPDEYKNLDHTVTIGTDQNLGQLNFIVKYSDRTRKLTVHLIRCLDLPDTRNRKQTA---IHRRSTSPLYN
NvectensisSyt26       PLKPKPSLQ-----------PQCSDCVIERHRDPNESLEIQSASFSQPSKK-GTLKFTLIYQTEFNNLIIQVINGHEIVGRDF-WDNAVDSYVQLKLDPDPGNVYKGKT-SISRKTTEPYYN
NvectensisSyt27       QTTPKTPLA-----------KDLGSLDPNLYTFETQEEEDGDVFFSGGNHKLGQVHFTVQYDIRRTVLTVRLIKAINLSPLDGEWSKPCNPFAIVQLQPD--YQHQLQS-YVQRKTTNPNFD
NvectensisSyt28       PLDAQYVAI-----------QPRTYHDLQKSEEQLLSENRAGSTSEVPERRKAKLKFTLQYNAIEEALTVKLLQAKNLSILTG--TNDVTYYVKLILPLSHRSFQ-SS-AVKGETDTTTFE
NvectensisSyt29       FEVPVNDLGNQTLCFRVIDLMKTGIPEWVPPTPPPPPKGRRRQKDLPPPP-------EPPPPPPPTLIGNVTVPLASVPMKKLLSDTEITIVRDIIKLRRKRVPAEVE---PSGELGESMTS
NvectensisSyt30       DTFYDALERY-------DSRRSMSLSSRDPSPTSANEDEEDEEDEVYPESHIGRLWFNIQYSTTGQNLVVTLVKARNLPSRSK-TVRTCDPFVKIALLPKDKHVSQSR---CKRKTKRPTFN
NvectensisSyt31       SVVSSNIGAI---------DTTAYTQCNELAIALAQPPEPPPLGTLLFEARYQKYEEQLTIHLIGAKRLPVRHHVIDVGDCPHYDVMPCDPKVLVCLLPDEKPVLESY---VKQSTWDPNFD
NvectensisSyt32       GSLSGSLSTF-------PELLQPNTDQLEPR-LYETRDDNDNHVGGSPFMGPSRLCFSLNYSHYNEKLQVKLIRATRLTPKGN-NV-VATPYVKMCLLPDRKRKLQST---RRHRNSNPAFN
NvectensisSyt33       RPLMKTDSSA-------SDITIGAWRAANLSPSSDSSDCNGNPFEQS--LGCGKLEFSIMYDTTECELKVRVLKANNL------SVPEATTYVEVFLLPDKIEKYQTN---VKYKNSNPVFN
NvectensisSyt34var1   SEYGPSDGRRAMSAYGDGIDMRDEVLSTAGRIQISASYAPTAEKLSVTVV-----RAEDIPTKQRGGASSVQVRIVILPTKKQRFKTKAKPATNPNFHETFTFTRITQ---NELRNLCLRVR
NvectensisSyt34var2   SEYGPSDGRRAMSAYGDGIDMRDEVLSTAGRIQISASYAPTAEKLSVTVV-----RAEDIPTKQRGGASSVQVRIVILPTKKQRFKTKAKPATNPNFHETFTFTRITQ---NELRNLCLRVR
NvectensisSyt35       GRTPSERVENASEVHSDTEVGSIRSASTARAGGVRGGEGGEEALLPGKAW-----IV-AEYYPDVGRVGFSIVEAQNVPMKGKGGGTHAR--FHVVLLPAK-----KQ---RFKTKHRRTPH
NvectensisSyt36       G--PMEPSTMTIVQKGSSGGVSSTEIGAGGRLH--LKFSLNNNKLSVKIS-----SI-QFAPHQKWKNEDLEVSCMLLPVHKQSFRRRAT-----RLGETIVIQIPPR---DKTPS-SLRFR
NvectensisSyt37       SDSPRQTPTRGMSSGSSTSEMGENQIRGVGKLYVTLDFTMHTCKLSVTIT-----KM-DFPPYQQRDPQQLEVSVMLLPGKRQSFRAKPK-----EFNDPVAIYLYPK---DKVKDMSLRFR
NvectensisSyt38       DLTFGSSSNNSAKYGSWAHGLAAPPSKDRAMSAYGDLTSLRRPSASDPRRRFAKLQVSISYAPTAQRLEIEVLRVEDVSDALIRSGIAEVVVHASILPSKRSRFKTK----AKPITNAAFH
NvectensisSyt39var1   ----------------------------------------------------------------------------------------------------------------------------
NvectensisSyt39var2   ------------------------------------------------------------------MFSIQRKKGWNGTAVLH--GNVTVSEKTEEYNYKVPNAAIILLSFLAFS
NvectensisSyt40       AKKPKKPLLKQFSLPAKIGQLSEDLVHHSPEKFIRDKQTEMQECVLE---------ISLFYDTASKELHITVINVSGIPTSDETFLRPPNCSVKMRILPEMLHWQWTR---KVSRTLNPVFN
NvectensisSyt41       DEKGAKREKRQKRRQSSNNTMITNSPEKVNMEEVKDVIINIDENEKHTSG-LGRLRFALNYNATKTELQVTIIKASDLFVHDNKEGIN-PYVK-ISLLPQKFCWQKTR---IVEDSPDAVFN
NvectensisSyt42       PSDADSVES--------------------------------EDPYIIETPGDYGKLLLQLDYSTEDDVLIVGVVQGKGVICKT--SVQTAQIYPVVQIYDENDRMLDERKIEIQPMTFEPQWN
NvectensisSyt43       PQLGRSMSAEAASGERTPPGSRPQYRRALSSQGGSLAPRKNSASRRNSIAPYGKMQVSLHFVSNKSLLVIQINGTYDLPQLR--TTGVSTPYVRVHLLPGNGSKDQ------RTNFMNLASN

                                                 400
NvectensisSyt1        EQFVFKNIPYSEITNRILLMELFDFDRFSR----HDLIGEARLPLIDVDLASNINEWRVLTPPSGSGGAGHSKS---DLGDICFSLRYVPSSGKLQITIVEAKSLKSM----DLTGYS-DPY
NvectensisSyt7        EVFTFEGFPHNKLMGKTLYMQVLDYDRFSR----NDPIGEVEIPLENIDLGPV-----TLTFTKDLLPCKKDRV---PLGDLLVSLMYQPTNNRIIVVMKANKLKAM----DLTGSS-DPY
NvectensisSyt21       ELFIISGLTLMEVQQLALQILVIHNEVISR----NVVIGEVMVALSGLELTGDEISI----WRDLRPYHFQNI----LGE-LHISVCHQPLSSRLSVTVLQARNLPKI-----SHLNIGDPY
NvectensisSyt24       ETFEFVGYSERDLMDLTLRMGLFAYDKFSR----GKMLGFTSVPFRDVEWHPT--QATILWRDLDTMVRRSFSY---PRGELSVSLRYEAQANRISIIVLKATALPKV----SLVKST-APY
NvectensisSyt25       ETFVFRITADEDVYSMSLLFVTFYYDQYSH----SHVLGEEQVPLVAFHLGDE-----TMLRCFLREESSMSHA---LVGDILISLLYTTKEQTLNVSIMKASNLSTS----LAASNE-KLY
NvectensisSyt26       ETFQFS-IEMEKLADAILSLLVMEVDRYSR----HHVIGQIDLELGDVVLANQ-----MVAMEMEILEAYKSRL---GLGDLLVSLSYMPTAERLSVVIMKARNLNPPFSEWSSQPRDISPF
NvectensisSyt27       ETFEFE-VLVQELQRLSLCITLHSFDTFAS----HDIIGQALLPLADLDLNKE-----NIYITD--LRPSLKHV---DLGELMVSLGYLKSAERLTVVLIKARNLP------AVNIKGTDPY
NvectensisSyt28       DSFEFK-IPNGELPRQNIKFTVRCFDFSH----HEIIGELRVNLCEQEPRGL-----SLSREVR-WDVLGPDR---SVGEVMVSLGYLRLTEKLAVVLVRARDL---------QPLKSDPY
NvectensisSyt29       GAEATTGTSEFEDAIDTADETFADSSREVSEKFTNVDGSSEKGDEERDRVSDERVAI------PRPPVSFAADELGQEGQMLLVSLAYWKPSEKLTVVMKGKNLKFI-----DNSRKPDPF
NvectensisSyt30       ETFYIP-IAEDELDSSTLQFTVFDGYRMSQ----QAIIGEAMYPLIDMESTGIQELWRDLEKPIEVSQAK-RKLTTSELGDIHISLSYYPTLDRLTIIILRAANLRTI----GHKGT--DPF
NvectensisSyt31       ETFVFK-LNTNEVKEKTLRFSVYDCTRLHK----LCPIGHTLFSLKGQDLDGTMAVERAIKPQCQTYGHS--------RGEISLSLTYNPGTSRLVVGVRQARNILQF-----EGHAHKEYF
NvectensisSyt32       EDFVFS-VPAEELRMRTLKFTVCDFDRFSR----QQVIGHVHFQLDCIDNLISPDGTGEIWVDINEDDAK-VQL---EKGQLLFSLQYLPTACRLTVAILKGKDLRVE----GEETTEIDTY
NvectensisSyt33       ETFEFD-VAFSELPERTLQFCINDYDGYSR----HQALGEV-FHSFDVNVNMQEDTVVIYEKDIRRDIFTFREENQVRKGEVLLSLGYLRLTEKLAVVLVKARLS--------------NPF
NvectensisSyt34var1   LYGHERIGKDKLLGESKIELTEFELDGEGTPLWRTLTPGNAIGSSDSMYDLSDTGSV---HSFGSYGSSSSLAMVQSGAPELLVSLCYQSLTGRLTVEVLKASNLRNV-----AMQRAPDSY
NvectensisSyt34var2   LYGHERIGKDKLLGESKIELTEFELDGEGTPLWRTLTPGNAIGSSDSMYDLSDTGSV---HSFGSYGSSSSLAMVQSGAPELLVSLCYQSLTGRLTVEVLKASNLRNV-----AMQRAPDTY
NvectensisSyt35       PKFRETFVFDRLVKEDLYRMAVRLFDGVAKEKLVGEINLQLADIAQAPGYKLN---AWRDLRKARNPADP--------------------------------------------------
NvectensisSyt36       LYDHKTITKKYLIGQGVVHVEDLKLEAEEVTIMLDMTSPNTYGIDNR---------------YISGIKPAQDSITEDPIPEILLSLEYRGLTRKLVAEVVKTRNLGLW-----TESKPCDLY
NvectensisSyt37       LYEHGSMGARHLLGEGVLRLRNVNLDNEMVPVAVDLQPPGSYVPEGA---------------SGAIMYEGELAISEEDRPREILISLEYRRLTGKLLEVIKTRNLGML-----TDSKAKEMY
NvectensisSyt38       ERWVVRNVSKKELFMQQLRFRLYSHEFLGM----GRILGETVVDVMSFDLEEVANSKWLTFTPAPEIKKLLNL-----------------------------------------------
NvectensisSyt39var1 ● -------------MQTKLMEKLHTRERNYISLGLRSLISREARALINDGHECEEDEEEWVLNTTNEKIKQAQETP-STIARLQVSAAYSETTGRIAVNICQATQLATV------LQQHGYNT
NvectensisSyt39var2   LIFLAIFFLILRRMQTKLMEKLHTRERNYISLGLRSLISREARALINDGHECEEDEEEWVLNTTNEKIKQAQETP-STIARLQVSAAYSETTGRIAVNICQATQLATV------LQQHGYNT
NvectensisSyt40       ETFIVPGFVHNKLRECTAHFVVLDFDHIQDNVY---VIGEVFMPLSELRANRLEKIV---------KRTFGK---------LLIAMSHNPIDKKLTVRVDSARGLPEM-----SR-RTTNAY
NvectensisSyt41       ESFVISGFSKERIREYKLSFCVVNYHEYFKERYADDVIGEIHFPLSELKVPDDKSTVSLTRWTDLRPVASSKSDPVDLGE-VCVSLCFRPFSGRLIVTVTKIQGLSKV-----A-VERTDPY
NvectensisSyt42       EEVLFA-LSGRPVTSLNVQVRLYELDSFSN----QHVIGCIKLPLAEQSFEESRADWYDLEDEHKIECDY--------VGEVLLSLNYFPTTQRLTIVVLKARNLKID----NVSGLW-GPL
NvectensisSyt43       RICAFDRITLEEAKKATLKFVILDYDKFSR----SEFVADIMMPLADIDLEQ--------------------------GETLTRHLNSQSRPETDX-----------------------XFQ

                                      500                                                                                600
NvectensisSyt1        VKIALVQEGRKIKKKKTTVKK-RTLNPYYNETFTFTVAFEKIEQTSLIISVLDYD-RVGKSEMIGKCVVGEL--SS--GADLRHWADMLAS--PRRSVAQWHTLHN-------------
NvectensisSyt7        VKMYIMHKDRRLDKKKTTIKR-RTRDPVWNESFIFDVPLDKIRDLTFVFNVMDYD-RITQNELIGQVILGYR--TT--GSSLRHWTEMMNN--PRKPVAQWHRLQDAL------------
NvectensisSyt21       VKVELF-SSRSRVGKKKTRVKKKTVNPKFAQTFTFDLAADSVLDMMTMTFTVMVQDASGCHERIGQVVLSASS-DG--P-EFGHWSQVIAN--PCPKPIEQWHMIHE-------------
NvectensisSyt24       CKVILSIDDKVIESRRTKCK--RSLSPVWNQGFILDIDKSRVNDYVITLRVMNHD-LLMSDDIIGEVVISSR--AN--GFAREHWDEMMRNKHSRKEVAMTHALE--------------
NvectensisSyt25       VKAVFSHEGQKVGKRKTTVKRKTLNPVYNEILLFRVPRETLERATLTVSLNRYN-VVGKSSTIGEVAFSPM--SK--GPEAAHWNDMLTR--AGVPTAMWHVLRGFKFREKEGHKPISR
NvectensisSyt26       VKVVLLFDGQKVKKKKTSTRK-QEKNPVYNESMLFDIPPHFLHRVILLISVADKPSDSRRSDIIGRVVIGSP--SS--GEALSHWNQMLVS--PRRPIAAWHKLRL-------------
NvectensisSyt27       VKVSLLINGKRVKKKKTSTKK-SDLHPVFNEAVSFDISKELLSNIDLLLSVMHD------NDVIGSFLIGAH--AQ--GKQHEHWQDVLTA--DR-PIAHWHILQDPKKF----------
NvectensisSyt28       ADVSICHAGRYLRHKQSHTLL-QECNPIFNETLTFHIPHGILHEVSIVISIRHDSGEGEDDVILGQVLLGPE--TT--GIQFEQWNDMRLN--NK-PIARWHKLNAF------------
NvectensisSyt29       VKLYIM-MGAKRLKKKRTLNKKRDANPVWNEAFSFNIP-HRLLHRVSVMLLAKHHSERGRERLLGKLVIGATS-SD--E-TVDHWNAMCTT--GKSVARWHHLIEEK------------
NvectensisSyt30       VKIAFSIGNKVMKTKKTAVHQ-DTVNPMFNDAFNYTVTEDDLGTSSLLVSVWHSGGGVREDKLIGRVLLGGMMYAR--GKECEHWTEMMTN--PRKMIKYWHPLGP-------------
NvectensisSyt31       VKISRIHLNRKVKTKRTPSTD-GDASPIFKHDMSFNIPTEQLSDSCLVLSLMNHG-VVRGSTTIGRVVLGPYVFGS--GNERSHWGRMIRS--PLNTIEQWHSLYL-------------
NvectensisSyt32       VKVSMIMGGKAVKKRKTPVVK-KTCNPVFNQAFVFSMPPSYLENVSFVISVIATPKRGGNKIHVGRAVVGPYMYST--SPGLSHWTDMLGS--PRSAVAQWHSLI--------------
NvectensisSyt33       VKVSLMVSGRQIKKTKTSVMH-GTLRPVYNEAFVFDIPVERLGHVSVMLLHVMLHSDGKRCQT--IAKTVVGPDS-QT--SIGLHHWNCMMTS--PRKPIAQWHPLLNS------------
NvectensisSyt34var1   VKVTLVSSSGKQVAKSKTTIRKSMADPEYNESFVYEISEKDLPEMTLMFSVIAISKTRKKKDMIGWFALGKNY-SG--QEEIRQWNDMIQQ--KEQKISQWHTLADA------------
NvectensisSyt34var2 ● VKVSMFNSIGQQLSKSKTSIRRSMYDPEFNETFVFQIIEFELPSVSLMFSVVNIKKMRRK-EIMGWFSMGRDN-TG--EDEESHWKEMIEG--KGKAVKRWHVLSAVEY----------
NvectensisSyt35       --------------------------------------------------------------------------------------------------------------------------
NvectensisSyt36       VDLTLKDKDRRVLRVCRTSVKRHVIDIENNEMFMFRLNNERMKEVTLIFSVVRVSDVRKKEEVLGSCAFGRDT-SG--QQQAEHWDNMLKD--EARVEYRWHTLYK-------------
NvectensisSyt37       VSVKLIGSNGELISKGRTTPRRHMIDPEYNEMFLFHVPEQELNSVTVLIALTSVSKTLGRKLKVGQLALGQNY-SS--EEEYRHWFEMTRS--KEKSIVRWHRINEMTVP----------
NvectensisSyt38       --------------------------------------------------------------------------------------------------------------------------
NvectensisSyt39var1   LQVHVKMQPLATKFRYKTLSK-PAYRAIFDEQFAFDGFRKRDLRKCWFRFRLYSFTKFGKKKIVGQVNVSVTDFEEN-GVWGTLWLDVTPSDEMYRLIASANELGNY-------------
NvectensisSyt39var2   LQVHVKMQPLATKFRYKTLSK-PAYRAIFDEQFAFDGFRKRDLRKCWFRFRLYSFTKFGKKKIVGQVNVSVTDFEEN-GVWGTLWLDVTPSDEMYRLIASANELGNY-------------
NvectensisSyt40       IKVDIW-FTGQKLFSRVTKVQYKSKSPIFNEVFIFDVSDDKLPQITISFRVKHHGKLR--EKHIGRVDLGANA-LT--EIEYRHWEQVLDK--PHLEIDQWHDIRSYNGSE---------
NvectensisSyt41       IKLSLY-CDGVRLSKANTRVKRRTLNPIYNEKFNFNVTADQISHTTVALRVANHREAHS--PSLGLVLLGYNS-RG--S-GQEQWHGMLES--PSRHIEKWHKILPDDLA----------
NvectensisSyt42       VKVFFHVNKRRVGRKRTAMQK-KTFNPVYNEAFAFKVSQEAMPKITFRILVVSKT-SHGQDEVLGHVTLGQN--VI--GSGFSHWSHMLAT--LRKPVAMWHPIIPFT-----------
NvectensisSyt43       VKVLYYVQNKLVDKKKTKVAK-GSTNPVFNEHFVFEMKEEELKNSSLLCEVYQSDSKL-RVERIGVINLGVE--SY--GTEIRHWNEMMLQ--PTKLVAEWHHLHPVK------------
```
