# Supplementary material for: A manual collection of Syt, Esyt, Rph3a, Rph3al, Doc2, and Dblc2 genes from 46 metazoan genomes - an open access resource for neuroscience and evolutionary biology
Source: BMC Genomics. 2010 Jan 15;11:37. doi: 10.1186/1471-2164-11-37 (PMC2823689; doi:10.1186/1471-2164-11-37)
Supplement: Additional file 41 — Alignment of the invertebrate Dblc2 sequences. Amino acid position is marked every hundred amino acids approximately, at the top of each page of the alignment. Splice variants are included and highlighted with black dots where they differ. Intron position and phase is indicated with a coloured bar between amino acids. Black bars indicate phase 0 introns. Red bars indicate phase +1 introns. Blue bars indicate phase +2 introns. [file 1471-2164-11-37-S41.PDF]

```
                                                                                                          100
                             |         |         |         |         |         |         |         |         |
TadhaerensDblc2              -------------MGAGTSKEKNEDMDNYRKSSIDSLEAAVN-QGILTPEQKEKEERNRK-----FRARKNNLMVLKELYRLMDPSVKK--S-GDAQGEVQLSFKYNDE
NvectensisDblc2              MGTGASA----ARRQWAVSEEENTAAPNAEAANVDSAVRDMA-GMAKLMEAKQKFLKNRKKNTACFQKELASTDLIKEMYKRMNPTVTQADS-GDIKGDIEMSLKYNHR
CapitellaDblc2               MGAAYSR----ASRVVDTEQSLTINVDDSVAMGAGHPEKIKSIARLLTAENQKKNRRKEQNEVG---NTKENLNILKQMFKKMDPSVLKTSSFMD-HGDIHLSFKYDPD
LgiganteaDblc2               MGTGPST----VDQPVNTKP---------LSQDPAKIEKLTRFLEAKAARDKSVTVAELTDKYE---QDKQSMKMLQGLFKKLDPRYGMAKTVGEVTGEIQISFKYDVR
CsavignyiDblc2               -------------------------MGSGASKTPNVKPKMTSSTYDVTVKKTEEEKMAEKHEMN-----VSNMYMLKRMFKQLDPVV--MNSNGGVQGKVNVSIKYITE
CintestinalisDblc2var1       -------------------------MGSGASKVTQPKPKVTSSNYDVTPKSSDVQDKHASGV--------NNMYMLKRMFKQIDPVV--MKSNHGIQGKINAAVKYVSG
CintestinalisDblc2var2     ● -------------------------MGSGASKVTQPKPKVTSSNYDVTPKSSDVQDKHASGVVSHMVTVKNNMYMLKRMFKQIDPVV--MKSNHGIQGKINAAVKYVSG
SpurpuratusDblc2             MGSANSSTSGPAREPAPHESAQVEQNFRSDNERVMQLARAMTTAKKRRHQTPASLMASQGNDFE---TYNESIFILKQLFKRMDPAV--TKTLGDAKGEVKLSLKYDKR
BfloridaeDblc2var1           -------MGNGASEPAVGQRSP--DNDTKNPEVIEGMARVMTTAKERRTANRNVFTREEQEEEEEYNYKESAVILTQLFKKMDPAV--MKSIGDVMGELKVSIKYKSD
BfloridaeDblc2var2         ● -------MGNGASEPAVGQRSP--DNDTKNPEVIEGMARVMTTAKERRTANRNVFTREEQEEEEEYNYK---------------------SIGDVMGELKVSIKYKSD
BfloridaeDblc2var3           -------MGNGASEPAVGQRSP--DNDTKNPEVIEGMARVMTTAKERRTANRNVFTREEQEEEEEYNYKESAVILTQLFKKMDPAV--MKSIGDVMGELKVSIKYKSD

                                                                                                          200
                             |         |         |         |         |         |         |         |         |
TadhaerensDblc2              QSAVLVKIIRARDLAIKDISAGTSDPYFKVDLIPDRNKEGAKKTRTVKKSLNPIYNEVIAFNVPPEQLHETRLRLMAYDWDLLGKDDFMGECIINLSELDFDQ--MGNG
NvectensisDblc2              EQLLLVKVIRARDLVPRDLN-GKSDPYVVLELVPDRLEEGTKKTRFKQKTLNPVFNEIFQFKVPHNNLTDTKLCATIWDHDFFGEDDFNGEAVVDLSRVNFNNG-THTD
CapitellaDblc2               SSNLLVKVICCRDLDPKDLRGQSADPYVKLFLHSSSQPSHVNRTATVKNTLNPNFNEIFSFEVDSNAFAESHVVIQVWDYDVLDKDDFIGEVIVPLPGLDLDHQAVHTA
LgiganteaDblc2               QEILLVKIIKCRELRCGGIRSKTSSPYVKMDMYPDNYSQGSQVTQIVVEENNPVFSEIFKFRTSEIELIENNLVVQVWDYDAITQDDFLGEVIIRLQDFDFNKSIVHTA
CsavignyiDblc2               KSLLLVKIISARDLLTKDVRGASANPYIKVEMHSNENM-EERSTSVKQNTCNPIYNEVFMFQL--NIEESTKLRLSVWCHDALGEDDFMGEHVINVSELDCSD-VITNK
CintestinalisDblc2var1       KSVLLVKVVCAQDLVAKDLRGSASDPYIKVGIYVDGHMIDEKSTSVKRNTRDPIYNEVMMYEVTNDMMTSLKIRMSVWSDDGLGDDDVIGEHVIDVAEMDCYD-VIINK
CintestinalisDblc2var2       KSVLLVKVVCAQDLVAKDLRGSASDPYIKVGIYVDGHMIDEKSTSVKRNTRDPIYNEVMMYEVTNDMMTSLKIRMSVWSDDGLGDDDVIGEHVIDVAEMDCYD-VIINK
SpurpuratusDblc2             RSMLLVKVVSARDLSAKDIRGRTSDPFVQMELIPDLHDEGKKSTQYVKKTLNPTFNEIFTFKLSEEDIAETQLRVRVLSHDPFGKADFIGENIIQLGSMDFSD--IITS
BfloridaeDblc2var1           QSLLLVKVVGARDLSPKDLRGKVANAFVQVDLVPDPAGSGIKHTNVVKKSLNPTFNEIFAFPCSAGILAETKLRLTVWNHDTLGAGDFMGERIVELRELEPNQ--VLTN
BfloridaeDblc2var2           QSLLLVKVVGARDLSPKDLRGKVANAFVQVDLVPDPAGSGIKHTNVVKKSLNPTFNEIFAFPCSAGILAETKLRLTVWNHDTLGAGDFMGERIVELRELEPNQ--VLTN
BfloridaeDblc2var3           QSLLLVKVVGARDLSPKDLRGKVANAFVQVDLVPDPAGSGIKHTNVVKKSLNPTFNEIFAFPCSAGILAETKLRLTVWNHDTLGAGDFMGERIVELRELEPNQ--VLTN

                                                                                                          300
                             |         |         |         |         |         |         |         |         |
TadhaerensDblc2              WYPLQQATDLSISGAIEISLEYKLPSTMIVTIIQGRDLVSRD-ISGKSDPFIRCYVVDTPNRYKTSVKHST-----------LNPVWDETFEFDIPQEEFSSRTIIFSV
NvectensisDblc2              WFMLQLQTDFSITGELDVTVEYLEPDTLAVTIHKATNLKSSNSMANTSDAYVRCSVSGLDYHEQTKVIPGT-----------LNPEFAEIFEFEVPREELDHRAIQFHV
CapitellaDblc2               WYTLKSETNLTITGSLEVSLRFQMPESLFVSVHSGSGLTNLY-EDDLPNPVVKVQIPGIQTLYETTVQKNT-----------VDPVWNETFEFCVPIEEFSSRYVVLHA
LgiganteaDblc2               WYNLNTSTDLSITGEVEVSLNYQLPDQLTVTIHHCIGLSPPD-GQSSANPFIKVAIPGTKTVHSTGVMKNS-----------LDPKWEESFDFSVAQEEFAFRYIVLHV
CsavignyiDblc2               WLSLHPETDFSISGKINISMTYQSPQSLFIAVNDVTELRCK---SQKPQISIRVYIPGVPYVFETKPASDVKMGDDVSADDVVSCEWKESFEFPVAKEELSSRYVVIVA
CintestinalisDblc2var1       WFHLHPQTDFSIGGDVTITMTYVMPQTLCVVINDVTNLHCK---NQNPSLLIRAIIPGIPFVHKTTTKIPHQQGNDV-----ISCDWNESFEFPVARGELPSRYVVLVV
CintestinalisDblc2var2       WFHLHPQTDFSIGGDVTITMTYVMPQTLCVVINDVTNLHCK---NQNPSLLIRAIIPGIPFVHKTTTKIPHQQGNDV-----ISCDWNESFEFPVARGELPSRYVVLVV
SpurpuratusDblc2             WFDLQPETDLGIQGELEITLTYQLPDTLKVRVHSAGGLVCRD-AKKMPHPYVKVLIPGIHKVEETKVQKKM-----------LDPVWEETFDFTLAQEEFTNRYLVLHV
BfloridaeDblc2var1           WYPLQEETDLSISGTLEISLAYQLPQLLCVTVHRASGLVCWD-QSKLPHPFVRVMVPGITMVEQTDVKRGT-----------LDPEWEQTYEFPVPQEEFDYRYVVLNV
BfloridaeDblc2var2           WYPLQEETDLSISGTLEISLAYQLPQLLCVTVHRASGLVCWD-QSKLPHPFVRVMVPGITMVEQTDVKRGT-----------LDPEWEQTYEFPVPQEEFDYRYVVLNV
BfloridaeDblc2var3         ● WYPLQEETDLSISGTLEISLAYQLPQLLCVTVHRASGL-----------------------VEQTDVKRGT-----------LDPEWEQTYEFPVPQEEFDYRYVVLNV

                                                                                                          400
                             |         |         |         |         |         |         |         |         |
TadhaerensDblc2              FDYDLTGKNDPMGDVHIHLTNFDIDNGLHEWFSLADLKNADRTRSQWAATAVVQEFREALTAHAIFGYPSFLFSKNSQIGLKTYSVRSRVAGSTARVQVVDGLLAK
NvectensisDblc2              FGKETLVQDVSLGQAYFDLKSLDLDEPERTSLPLADLKNECLKRAEWAQNAVAQEFREAMYAHAMYKHPTFIYSMHNK-GRKMYSVSSRNAGSSAKVFLVNGIPV-
CapitellaDblc2               IDEGGSGENESLGQIIIELNNLNTDAGFHGSFQLADLRNSDRLRAQWFQHATLQEFREALVAHAAFRQPKFLFQ--SHSGTKMLRVVNKKAKSEARLRIVNGIPVF
LgiganteaDblc2               IDDNNTEGNCSLGQVIIDLDTFNPVEEHRQTYKLADLKNSEHLKNKTMQSSISQELRESFLAHAAVQHPKFLFQGDSHTGKRIVTVTCRKAKNRAKIRVVDGILVA
CsavignyiDblc2               HD---VTNE-YLGECHIDLDNFDCDVGYSGLFRLSDMRGNAMVRSRWSRNAVSSEFQDALLAHAQYKKPNFVFS--RAGTNKAVSLRVPKAGAESRIRVVNGILVH
CintestinalisDblc2var1       CD---VTEDNYLGECQIDLDNFDCDVGYSGTFRLSDMRGNAKVRSRWSSDAVSEELKQAMAAHAVYKMPQFIFT--RVGDNKAISLRVPKVGTESRIRVVNGILVR
CintestinalisDblc2var2       CD---VTEDNYLGECQIDLDNFDCDVGYSGTFRLSDMRGNAKVRSRWSSDAVSEELKQAMAAHAVYKMPQFIFT--RVGDNKAISLRVPKVGTESRIRVVNGILVR
SpurpuratusDblc2             LDKALVGDSEDMGQVFIDLDNLDISQGFSGKFPLADLKNSERTRTKWSQTATVQEFREAMYAHSVYRYPGLVFQ--KQRGNKVLTVHSRKAGSSAKVRIVNGVPT-
BfloridaeDblc2var1           CDK--FRDGESMGEVHIDLCNFDPEEGYTXSFPLADMRNSDRVRTKWAQRALLQEYKEAMYAHSAYQFPKFLFH--KHQGHKVVSVSSRKAGCQAKVRIIGGVPIS
BfloridaeDblc2var2           CDK--FRDGESMGEVHIDLCNFDPEEGYTXSFPLADMRNSDRVRTKWAQRALLQEYKEAMYAHSAYQFPKFLFH--KHQGHKVVSVSSRKAGCQAKVRIIGGVPIS
BfloridaeDblc2var3           CDK--FRDGESMGEVHIDLCNFDPEEGYTXSFPLADMRNSDRVRTKWAQRALLQEYKEAMYAHSAYQFPKFLFH--KHQGHKVVSVSSRKAGCQAKVRIIGGVPIS
```
