# Supplementary material for: A manual collection of Syt, Esyt, Rph3a, Rph3al, Doc2, and Dblc2 genes from 46 metazoan genomes - an open access resource for neuroscience and evolutionary biology
Source: BMC Genomics. 2010 Jan 15;11:37. doi: 10.1186/1471-2164-11-37 (PMC2823689; doi:10.1186/1471-2164-11-37)
Supplement: Additional file 42 — Alignment of the invertebrate Esyt2 sequences. Amino acid position is marked every hundred amino acids approximately, at the top of each page of the alignment. Splice variants are included and highlighted with black dots where they differ. Intron position and phase is indicated with a coloured bar between amino acids. Black bars indicate phase 0 introns. Red bars indicate phase +1 introns. Blue bars indicate phase +2 introns. X residues indicate where a portion of sequence is missing. [file 1471-2164-11-37-S42.PDF]

```
                                                                                                                                  100
TadhaerensEsyt2                -----------------------------------------------------------------------------------------------------------------------------------------
NvectensisEsyt2a               --------------------------------------------------------------------------MSELGE--------KISLFRK-EFFSHFFTFLG-WFVLAWIAWAFGALHLSFA--WLVLFLFI
NvectensisEsyt2b               -----------------------------------------------------------------MSEGIEDVQPLPFYK--------RIAINIR-NMSRDEVMELGRYFAVTLALWLVGYMHFSFA--WIVMILMI
NvectensisEsyt2cvar1           ---------------------------------------------MAAQREGSSIKTGEEKAPLTTNQNQNDPKAAEEPE--------KKAKPTEIP-IVTFVLKYLKFVGVALGIWFMGWMGLSYV--WVLCGLLV
NvectensisEsyt2cvar2         ● ---------------------------------------------MGETQKASSLENSVKQ----DEQLSQDVPPSDMPS--------APPKSEESQLIVKVIMTFLKYTGATLLVWFVGWFGFGYL--WVVLGTLI
CapitellaEsyt2                 ----------------------------------------------------------------MADSQPKPPTKPPNQQ------INGAGSQTQGVQLLPLITKYFKTAGVLLLAWSAGYFRFSST--WVLIGMF-
HrobustaEsyt2a                 -------------------------------------------------------------------------MSAVDDYHTLLLAKREKLNKGNDGSLISLILKGFKTTGIAIAVWCWGYLGMSVA--WIFVLLF-
HrobustaEsyt2bvar1             ---------------------------------------------------------------MSEISEMTPVKSDIATVDTDSQRNLKKTTRSQTVQLLPLIVKSATTAGFLFAVWGAGFYRFSVS--WVIGAST-
HrobustaEsyt2bvar2             ---------------------------------------------------------------MSEISEMTPVKSDIATVDTDSQRNLKKTTRSQTVQLLPLIVKSATTAGFLFAVWGAGFYRFSVS--WVIGAST-
LgiganteaEsyt2var1             ------------------------------------------------------------------MPPPAKPEEKPAES------KAKRTAVLEDTLLFNAVKNYFKYAGVVLGVWGFGYFSFSPS--WLLLGLV-
LgiganteaEsyt2var2             ------------------------------------------------------------------MPPPAKPEEKPAES------KAKRTAVLEDTLLFNAVKNYFKYAGVVLGVWGFGYFSFSPS--WLLLGLV-
CsavignyiEsyt2                 ----------------------------------------------------------------------------------------------XISDTAALFKLGVTRFFFSLVIWLLGYFNFSIL--WLVIGVWL
CintestinalisEsyt2var1         MPVESRRTDSGFTDEDNLDGEFENSNEEVFNFMNPNEKGVDDAPSVSDVSSTTES-----------DGTRPQSVGDRIQ----------TVVKARIDNTIALVKLGVTRFFIAVFIWLLGYFNFSIL--WIVIGVWL
CintestinalisEsyt2var2       ● MPVESRRTDSGFTDEDNLDG------------------GVDDAPSVSDVSSTTES-----------DGTRPQSVGDRIQ----------TVVKARIDNTIALVKLGVTRFFIAVFIWLLGYFNFSIL--WIVIGVWL
CintestinalisEsyt2var3         MPVESRRTDSGFTDEDNLDGEFENSNEEVFNFMNPNEKGVDDAPSVSDVSSTTES-----------DGTRPQSVGDRIQ----------TVVKARIDNTIALVKLGVTRFFIAVFIWLLGYFNFSIL--WIVIGVWL
CintestinalisEsyt2var4         MPVESRRTDSGFTDEDNLDGEFENSNEEVFNFMNPNEKGVDDAPSVSDVSSTTES-----------DGTRPQSVGDRIQ----------TVVKARIDNTIALVKLGVTRFFIAVFIWLLGYFNFSIL--WIVIGVWL
SpurpuratusEsyt2               ----------------------------------------------------------------MVPGNSETVGDETTTE------PVRNIKVEESTDLLSSIRKHGFILGILLMVWLVGYLGFSVL--WLLLIVV-
BfloridaeEsyt2a                ----------------------------------------------------------------------MEPEEPKVET-----DRSWRDLWDTYHPTTDSIRIYVLSLGVLALVWLVGYWGFSVT--WVMLGLFI
BfloridaeEsyt2b                -------------------------------------------MADGSESGAGSAGTQAGREAGSAAGGDGQAPGGDTQP-----PPAAPSRLQVNEDLLVLIKKYAMQLGLLVVVWMVGYWAFSVS--WIMLGLFV
IscapularisEsyt2               ----------------------------------------------MDANRPSTTETPADAEPLLPAEKPPAEKPASTEP-------VKQARAILRTTLGSLVYKFAKGMLIAGSLYIMGWLNLSPA--WLLMGVAS
DpulexEsyt2var1                ---------------------------------MTDTKSSDDVIAVSREMPPSASKTP--------SPEKEIVPANQKKK---------KPPPTLGKSLVGLCKIAAKHLPLVVGVYAMGYFNFSIA--WILGIVG-
ApisumEsyt2                    ----------------------------------------------------------------MADRQGEELRLRDVDNDSSALVEEAADSTLEKPTVRQTVWRVTKGLVPVVTIYALGFWRVASASVWVLMPFFF
TcastaneumEsyt2avar1           ----------------------------------------------------------------------MSVSKDGSQA---------MEKRSEGSSILSVIFSAVKKASIVGIVYFAGYMQWSVA--WFIGPIV-
TcastaneumEsyt2b               --------------------------------------------------------------------MSRDLTASEVSV-------PLSE----TILNVETVVKLIKKLGFFFLIYLLGYYEFSVVLPYALLTFVV
TcastaneumEsyt2c               -----------------------------------------------------------------MTESDNLIPPLEPEI-------QLGQLNGSAMKKRFFVIKIFYKAILIFVGYAISYMQWSFILLFVTAGTLI
NvitripennisEsyt2var1          ---------------------------------------------MGEGTTETASKIKSWPVMCMMALASGITENDPNGV-----EEIEAPKTWPYMTFASLGKSLFMKLATVGVIWGVGYMNWNFA--WLIPPIA-
AmelliferaEsyt2var1            -------------------------------------------------------------------------MEDKCED-----NKISKSNPWPYMNIGSLTISFLTKLATAGIIWSWGYLNLNIA--WLIGPIA-
AgambiaeEsyt2var1              ----------------------------------------------------------------MAGASKELSPEKQSAP-----PESSEVAKTKDDSIMTLLYSFAKKVVTVGIIYFVGYMGWSVA--WLITPVI-
DmelanogasterEsyt2var1         ----MSDNSPSVPLAEFTIPPDPT-ETEPFVPLVEVKKMNESP--VVTPTTPTGNGTPP-----TQT-----TNGNSIVA---------TKSVSDDNSIFSVFYTLGKKVAIVGSIYLVGYMGWSVA--WLIAPVI-
DmelanogasterEsyt2var4         -----------------------------------------------------------------------------------------------------------------------------------------
DmelanogasterEsyt2var5         ----MSDNSPSVPLAEFTIPPDPT-ETEPFVPLVEVKKMNESP--VVTPTTPTGNGTPP----------TQTTNGNSIVA---------TKSVSDDNSIFSVFYTLGKKVAIVGSIYLVGYMGWSVA--WLIAPVI-
DmelanogasterEsyt2var6         -----------------------------------------------------------------------------------------------------------------------------------------
DmelanogasterEsyt2var7         ----MSDNSPSVPLAEFTIPPDPT-ETEPFVPLVEVKKMNESP--VVTPTTPTGNGTPP----------TQTTNGNSIVA---------TKSVSDDNSIFSVFYTLGKKVAIVGSIYLVGYMGWSVA--WLIAPVI-
DmelanogasterEsyt2var8       ● --------------------------------------MNESP--VVTPTTPTGNGTPP-----TQT-----TNGNSIVA---------TKSVSDDNSIFSVFYTLGKKVAIVGSIYLVGYMGWSVA--WLIAPVI-
DsimulansEsyt2var1             ----MSDNSPSVPLAEFTIPPDPT-ETESFVPLAEAKKMSESPNPPVTPTTPTGNGTPP-----TQT-----TNGNSTVA---------TKSVSDDNSIFSVFYTLGKKVAIVGSIYLVGYMGWSVA--WLIAPVI-
DsechelliaEsyt2var1            ----MSDNSPSVPLAEFTIPPDPT-QTESFVPLAEAKKMNESP--VVTPTTPTGNGTPP-----TQT-----TNGNSTVA---------TKSVSDDNSIFSVFYTLGKKVAIVGSIYLVGYMDWSVA--WLIAPVI-
DerectaEsyt2var1               ----MSDNSPSVPLAEFTIPPDPT-ETEPFVPLVEAKKMSESP--VVAPATPTGNGTPP-----TQT-----ANGNSTVA---------TKSVSDDDSIFSVFYTLGKKVAIVGSIYLVGYMGWSVA--WLIAPVI-
DyakubaEsyt2var1               ----MSDNTPSVPLAEFTIPPDPT-ETEPFVPLVEAKRMNESP--VVAPATPTGNGTPP-----TQT-----ANGNSTVA---------TKSVSDDNSIFSVFYTLGKKVAIVGSIYLVGYMGWSVA--WLIAPVI-
DananassaeEsyt2var1            ----MSDNTPNVPLAEFVDPPNPA-EDEPFVPLAEPAKKMSEPL-VAAPATPTGNGTPT-----EPTFGNGNGNGNKAVA---------TRSTSGEDSIFSILYTVGKKVAIVGSIYLVGYMGWSVA--WLIAPVI-
DpseudoobscuraEsyt2var1        -----XDNTPSVPLADFIEPPNPT-EDEPFVPLAEPRKMNDTPVAAAATPTPTTATTAP-----ISNGTPTEAAAASAAA-----TTTVATSGSSENSIFSILYTIGKKVAIVGSIYLVGYMGWSVA--WLIAPVI-
DpersimilisEsyt2var1           -----XDNTPSVPLADFIEPPNPT-EDEPFVPLAEPRKMNDTPVAVAATPTPTTATTAP-----ISNGTPTEAAAASAAAASAAATTTVATSGSSENSIFSILYTIGKKVAIVGSIYLVGYMGWSVA--WLIAPVI-
DwillistoniEsyt2var1           -----XDNTRSVPLADFVDPPKPE-EEESFVPLAEVKKMNDLP---TTPTTPTGPVTPS-----TQSTPNSTPNDGVAKP---------IASQSNDNSIFSIIYGVGKKVAIVGSIYLVGYMGWSVA--WLIAPVI-
DvirilisEsyt2var1              MLSDKNAAESSTPLADFIVPPDPERVEEPIVPLAQLPKMTETTA-LPSIATPTA--TPAA----TSTATPTTANGNTTVA---------RSRSSDEDGIFSVLYSVGKKVAIVGSIYLVGYMGWSVA--WLIAPVI-
DmojavensisEsyt2var1           --XDTNAAEPTVPLAEFVVPPNSD-LEEPIVPLAQPPKMNETPA-ATPAATPTAAATPTTEATTTTTATPSAGNDSAAVA---------KPSSSNEDSIFSVIYSVGKKVAIVGSIYLVGYMGWSVA--WLIAPVI-
DgrimshawiEsyt2var1            MQSDKNEAVPSVPLADFIVPPDPQ-QEEPILKLAEPPTMSETPV-SNPATRTTTA---------TTAATVNGNATNQVVA---------SSRNSDENSIFSMIYTVVKKVAIVGSIYLVGYMGWSVA--WLIAPVI-
Celegansesyt_2var1             ------------------------------------------------------------------------------------------------MSWQSYLVPLVGSAILSTFTFFLGKWDYSFV--WVLIIII-
Celegansesyt_2var2             ------------------------------------------------------------------------------------------------MSWQSYLVPLVGSAILSTFTFFLGKWDYSFV--WVLIIII-
Cbrenneriesyt_2var1            ------------------------------------------------------------------------------------------------MTWQSYVVPLVGSALISTFTFFLGKWDYSFV--WVLIIVM-
Cbrenneriesyt_2var2            ------------------------------------------------------------------------------------------------MTWQSYVVPLVGSALISTFTFFLGKWDYSFV--WVLIIVM-
Cbriggsaeesyt_2var1            ------------------------------------------------------------------------------------------------MTWQSYVLPLIGSALLSTFTFFLGKWDYSVV--WVLVIVI-
Cbriggsaeesyt_2var2            ------------------------------------------------------------------------------------------------MTWQSYVLPLIGSALLSTFTFFLGKWDYSVV--WVLVIVI-
Cremaneiesyt_2var1             ------------------------------------------------------------------------------------------------MTWQSYVVPLVGSALLSALTFFLGKWDYSFV--WVLIIVL-
Cremaneiesyt_2var2             ------------------------------------------------------------------------------------------------MTWQSYVVPLVGSALLSALTFFLGKWDYSFV--WVLIIVL-
Cjaponicaesyt_2                ------------------------------------------------------------------------------------------------MSWQSYVVPIVGSALLSTFTFFLGKWDYSFL--WIIIIVI-
```

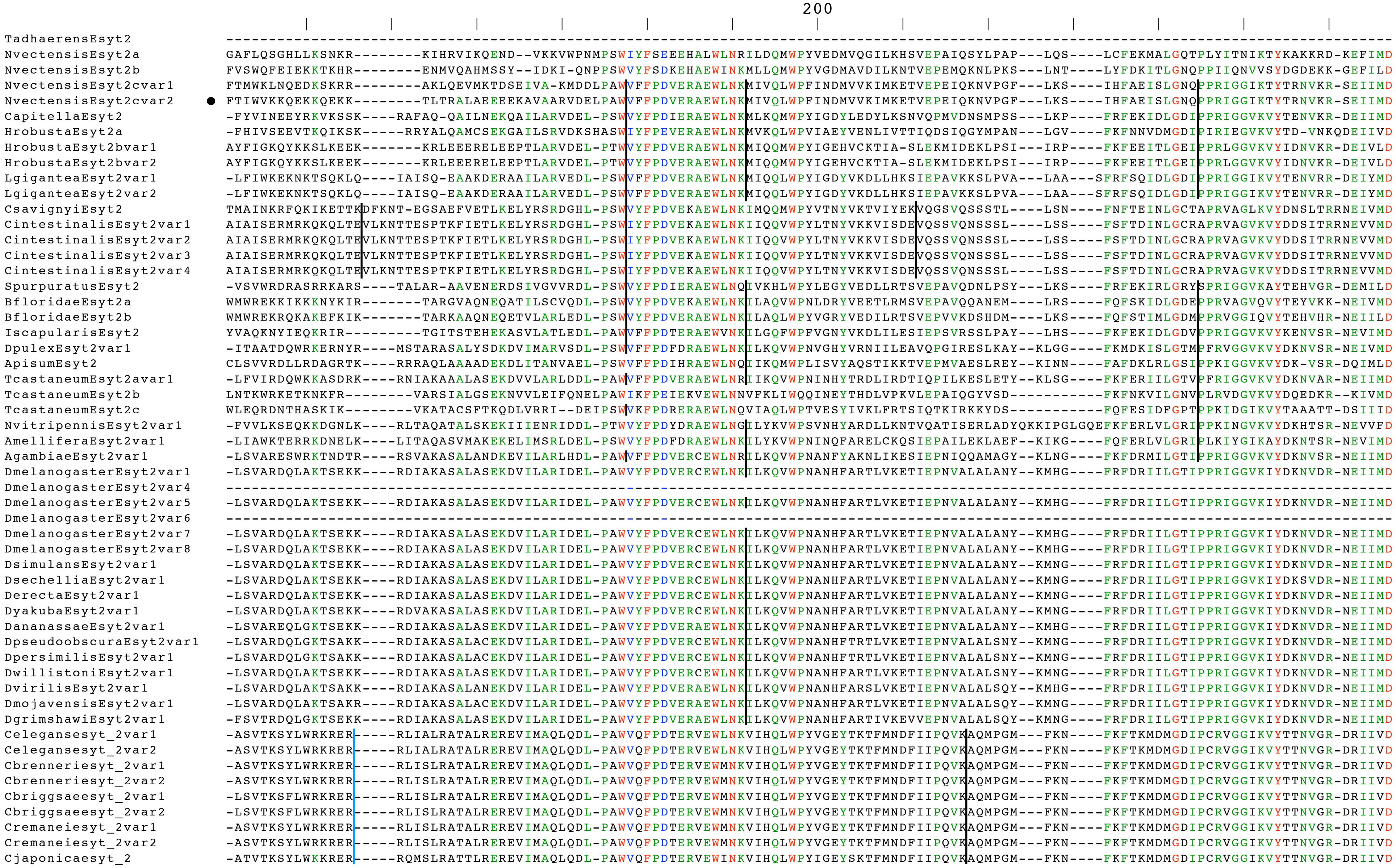
200
TadhaerensEsyt2
NvectensisEsyt2a
NvectensisEsyt2b
NvectensisEsyt2cvar1
NvectensisEsyt2cvar2
CapitellaEsyt2
HrobustaEsyt2a
HrobustaEsyt2bvar1
HrobustaEsyt2bvar2
LgiganteaEsyt2var1
LgiganteaEsyt2var2
CsavignyiEsyt2
CintestinalisEsyt2var1
CintestinalisEsyt2var2
CintestinalisEsyt2var3
CintestinalisEsyt2var4
SpurpuratusEsyt2
BfloridaeEsyt2a
BfloridaeEsyt2b
IscapularisEsyt2
DpulexEsyt2var1
ApisumEsyt2
TcastaneumEsyt2avar1
TcastaneumEsyt2b
TcastaneumEsyt2c
NvitripennisEsyt2var1
AmelliferaEsyt2var1
AgambiaeEsyt2var1
DmelanogasterEsyt2var1
DmelanogasterEsyt2var4
DmelanogasterEsyt2var5
DmelanogasterEsyt2var6
DmelanogasterEsyt2var7
DmelanogasterEsyt2var8
DsimulansEsyt2var1
DsechelliaEsyt2var1
DerectaEsyt2var1
DyakubaEsyt2var1
DananassaeEsyt2var1
DpseudoobscuraEsyt2var1
DpersimilisEsyt2var1
DwillistoniEsyt2var1
DvirilisEsyt2var1
DmojavensisEsyt2var1
DgrimshawiEsyt2var1
Celegansesyt_2var1
Celegansesyt_2var2
Cbrenneriesyt_2var1
Cbrenneriesyt_2var2
Cbriggsaeesyt_2var1
Cbriggsaeesyt_2var2
Cremaneiesyt_2var1
Cremaneiesyt_2var2
Cjaponicaesyt_2

```
                                       300
TadhaerensEsyt2              -----------------------------------------------------------------------------------------------------------------------------------------
NvectensisEsyt2a             LDVVYNGDAHFTLGIKKVQ---LGISDLKIHGPLRVILKPLLSDYNPVGGVTVFFLNRPKISFDLTNLLSVLDIPGLKGTLLDIVEDVVASFVVLPNRIAVPLSASVDAGDLQ--------------YPIPDGVLRV
NvectensisEsyt2b             LDLKYEGDAQVKLSVKNVK---LGLTKFKLNGILRVIFKPLVSLYNPIGGVTVFFLNRPKTKFDLTNLLNVLDFPGLNSTLRRIVDDTIASFVVLPNRVAIPLAEGVDASDLQ--------------YPIPQGVLRV
NvectensisEsyt2cvar1         VDLIYAGDADIQLSVKGIS---VGIEDLQLRGTLRVIMSPLVPSAPLVGGISVFFLNRPDIDFDLTNLLNILDIPGLSDILRGVVGDVVASFVVLPNRICIPLTD-VDPYKLK--------------YPLPDGVLRI
NvectensisEsyt2cvar2         VDLIYAGDADIQLSVKGIS---VGIEDLQLRGTLRVIMSPLVPSAPLVGGISVFFLNRPDIDFDLTNLLNILDIPGLSDILRGVVGDVVASFVVLPNRICIPLTD-VDPYKLK--------------YPLPDGVLRI
CapitellaEsyt2               LELFYAGDCQVTVAVRGMN---AGIRDFTLHGTVRIVMKPLVNIIPIIGGMQIFFLNQPNIDFDLTNAANILDIPLLSQSLRTVVEDYVSQFMVLPNKIPVTLAANVQSAMLR--------------NIMPQGVIRI
HrobustaEsyt2a               VNVRYAGDANIKCTVKGIN---FGIKSLRIYGNLRCVINPLISDVPIIGGLTVFFLNCPEVTFDMTAAANILDLPVIKKTIKDIVCNVLSDLCVLPRRIPIALHGSVDSTMIK--------------YPPPEGLVII
HrobustaEsyt2bvar1           AEITYAGDCDINLSMRGLH---AGIKDILLHGMIRIIFRPLINKIPLIGGLEFCFINLPNLDFDLTNLANIFDVPFLNDSLRKIITDQVSNFLVLPNVIRVDLVSPSDLLNTKPSGHRGVTVGGIGGITLPYGVLRI
HrobustaEsyt2bvar2           AEITYAGDCDINLSMRGLH---AGIKDILLHGMIRIIFRPLINKIPLIGGLEFCFINLPNLDFDLTNLANIFDVPFLNDSLRKIITDQVSNFLVLPNVIRVDLVSPSDLLNTKPSGHRGVTVGGIGGITLPYGVLRI
LgiganteaEsyt2var1           LEIIYSSDSEITVQAKGIN---AGIKDLQIHGTMRVIFKPLISRIPLFGGLSVFFLNNPTVDFNLTSLANAFDLPGLSDMLKNIVQEQIANIMVLPNRIPVSMVKGLDLNKLR--------------YPQPQGVLRI
LgiganteaEsyt2var2           LEIIYSSDSEITVQAKGIN---AGIKDLQIHGTMRVIFKPLISRIPLFGGLSVFFLNNPTVDFNLTSLANAFDLPGLSDMLKNIVQEQIANIMVLPNRIPVSMVKGLDLNKLR--------------YPQPQGVLRI
CsavignyiEsyt2               IQIVYDSECNCGVSVNRLQ---AGISNFSVRGLLRVEFHPLIEQIPLVGAVSLSFVNDPCIDFNLTELANLFDLPGFNHLLRGAISDGVCGMMVLPDKYVIKLHPDVDISRIR--------------FPLPQGVIRI
CintestinalisEsyt2var1       IQIVYDSECNCGVSVNRLQ---AGICDLRLRGLLRVEFHPLIEDLPLIGAVSVGFVNDPFIDFDLTDLANLFDLPGFNSLLRGAISDSVCGMMVLPDKYVIKLCPDIDISRLR--------------FPLPQGVIRI
CintestinalisEsyt2var2       IQIVYDSECNCGVSVNRLQ---AGICDLRLRGLLRVEFHPLIEDLPLIGAVSVGFVNDPFIDFDLTDLANLFDLPGFNSLLRGAISDSVCGMMVLPDKYVIKLCPDIDISRLR--------------FPLPQGVIRI
CintestinalisEsyt2var3       IQIVYDSECNCGVSVNRLQ---AGICDLRLRGLLRVEFHPLIEDLPLIGAVSVGFVNDPFIDFDLTDLANLFDLPGFNSLLRGAISDSVCGMMVLPDKYVIKLCPDIDISRLR--------------FPLPQGVIRI
CintestinalisEsyt2var4       IQIVYDSECNCGVSVNRLQ---AGICDLRLRGLLRVEFHPLIEDLPLIGAVSVGFVNDPFIDFDLTDLANLFDLPGFNSLLRGAISDSVCGMMVLPDKYVIKLCPDIDISRLR--------------FPLPQGVIRI
SpurpuratusEsyt2             LEIFYAGDCDIEISVKTVKRLKAGIQDLQLHGTLRVEMRPLVNKMPLIGGMSIYFLNRPAIDFNLTNLADLLDVPGLSNMLHGILEDQFACFLVLPNRIPLTFMDTTDINELK--------------YPMPKGVLRI
BfloridaeEsyt2a              MDLMYSGDCDIQIRIKRFL---AGVQDLQVQGTVRVVMKPLMSQHPLVGGITVFFLNRPDINFNLSNIGEVLNFPGLSSMLKGVVADQVAAFMVLPNRFPIPLVPDLDISRLR--------------YPMPKGVLRI
BfloridaeEsyt2b              MEIMYAGDCDIQIRMKRFL---AGIQDLQIHGTLRVVMKPLVKFSPLIGGITVFFLNRPEIDFNLTNLADVFDFPGLSSLLKGIVADQVSNFMVLPNRYPMPLIPDLEVAKLK--------------YPMPVGVLRI
IscapularisEsyt2             LELFYSGDCKFSIKVKGFK---AGIRDLQVHGHLRVVMRPLTKEMPIVGGVTVFFLRPPAIDFQLTNLGQVLEVPGLNDLLKKAVSDQVAAMMVLPNKFSMKLQEHVSTQSLR--------------FSLPCGVLRL
DpulexEsyt2var1              MDICYAGDCDIRFSIKGLK---GGIKDFQMSGMLRVIMKPLISQIPLFGGIQIFFLNSPSVDFNLIGVVDVLDMPGLNGILRRVIIEQIGAFLVLPNKLSFTLSDVVSPIVVK--------------IPEPSGVLRV
ApisumEsyt2                  IDVIFASDSDISFYVSGIP---CGIKDFQIRGMMRVVMRPLLTTSPLVGGMQIFFLNQPDIDYDLMGVADVLDMPGLNDVLRKVISQQVAALMVLPNKLPIVLSNEIAAHVVK--------------LPEPEGVLRV
TcastaneumEsyt2avar1         LDIFYAGDCDITFYLAGIK---GGIRDFQLHGMLRVVMKPLITTIPLVGGLQVFFLNNPDIDFDLIGIADLLDMPGLSDILRRIVVETVASMMVLPNKFPIKLSDDVDAMELK--------------APEPEGVLRV
TcastaneumEsyt2b             LNISYAGDCYVTFH-TFRFT--GGIEKIQFHGTVRVVLTPLISKMPLIGGLQVYFMDEPHIDFDLIKATSILDLPYVRNKIKNTTMNVINSMFMYPNVYSINLTEGINMSKLT--------------VFRTEGILRV
TcastaneumEsyt2c             FDVFYDGDCDINFSFSGAEI--GGIRDFQLSVEVRVVLKPLLPKVPLIGGIQIYFLNTPDINFTL---EGLSGIPGLSSFIRSKIEEKITKKIVFPNKITKRFSKSVAPSELK--------------ALEPAGVLRV
NvitripennisEsyt2var1        MDIMYAGDCDITFSMGTFK---AGIKDFQMRGMLRVTLKPLIPIIPIAGGVQVFFLNCPIIDFNLVGVADILDLPGFSDVLRKIITEQIAAIAVLPNKFSMPLTDEVPAEVMK--------------TPEPEGVLRI
AmelliferaEsyt2var1          ADIMYAGDCDITFSVGNIK---GGIRDFQIRGMMRIVMKPLLPVMPIVGGVQAFFLNPPAINFNLIGVADVLDLPGFNEILRKTIVEQIAAFVVLPNKIVIPLSEAVPIESLK--------------IPEPEGVLRI
AgambiaeEsyt2var1            LDLFYAGDCDISFALSGLR---GGIKDFQIHGTVRVIMKPLISQMPLIGGLQIFFLNNPNIDFNLVGVVDLLDMPGLSDILRKIIVEQVAAIMVLPNKLPIVLSDGVPALSLK--------------MPEPEGVLRI
DmelanogasterEsyt2var1       LDLFYASDCDINFYLGGMK---GGIKDFQIHGWVRVVMKPLIRSMPLVGGLQIFFLNNPNIDFNLVGVIDFMDMPGLSDLLRRIIVEQIGNVMVLPNKLPISLSEEVSAVALK--------------MPEPEGILRI
DmelanogasterEsyt2var4       -----------------------------------------------------------------------------------------------------------------------------------------
DmelanogasterEsyt2var5     ● LDLFYASDCDINFYLGGMK---GGIKDFQIHGWVRVVMKPLIRSMPLVGGLQIFFLNNPNIDFNLVGVIDFMDMPGLSDLLRRIIVEQIGNVMVLPNKLPISLSEEVSAVALK--------------MPEPE-----
DmelanogasterEsyt2var6       -----------------------------------------------------------------------------------------------------------------------------------------
DmelanogasterEsyt2var7       LDLFYASDCDINFYLGGMK---GGIKDFQIHGWVRVVMKPLIRSMPLVGGLQIFFLNNPNIDFNLVGVIDFMDMPGLSDLLRRIIVEQIGNVMVLPNKLPISLSEEVSAVALK--------------MPEPEGILRI
DmelanogasterEsyt2var8       LDLFYASDCDINFYLGGMK---GGIKDFQIHGWVRVVMKPLIRSMPLVGGLQIFFLNNPNIDFNLVGVIDFMDMPGLSDLLRRIIVEQIGNVMVLPNKLPISLSEEVSAVALK--------------MPEPEGILRI
DsimulansEsyt2var1           LDLFYASDCDINFYLGGMK---GGIKDFQIHGWVRVVMKPLIRSMPLVGGLQIFFLNNPNIDFNLVGVIDFMDMPGLSDLLRRIIVEQIGNVMVLPNKLPISLSEEVSAVALK--------------MPEPEGILRI
DsecheliaEsyt2var1           LDLFYASDCDINFYLGGMK---GGIKDFQIHGWVRVVMKPLIRSMPLVGGLHIFFLNNPNIYSNLVGVIDXXXXXXXXXXXXRIIVEQIGNVMVLPNKLPISLSEEVSAVALK--------------MPEPEGILRI
DerectaEsyt2var1             LDLFYASDCDINFYLGGMK---GGIKDFQIHGWVRVVMKPLIRSMPLVGGLQIFFLNNPNIDFNLVGVIDFMDMPGLSDLLRRIIVEQIGNVMVLPNKLPISLSEEVSAVALK--------------MPEPEGILRI
DyakubaEsyt2var1             LDLFYASDCDINFYLGGMK---GGIKDFQIHGWVRVVMKPLIRSMPLVGGLQIFFLNNPNIDFNLVGVIDFMDMPGLSDLLRRIIVEQIGNVMVLPNKLPISLSEEVSAVALK--------------MPEPEGILRI
DananassaeEsyt2var1          LDLFYASDCDINFYLGGFK---GGIKDFQIHGWVRVVMKPLIRSMPLVGGLQIFFLNNPNIDFNLVGALDFMDMPGLSDLLRRIIVEQIGNVMVLPNKLPISLSEEVSAVALK--------------MPEPEGILRI
DpseudoobscuraEsyt2var1      LDLFYASDCDINFYLGGMK---GGIKDFQIHGWVRVVMKPLIRSMPLVGGLQIFFLNNPNIDFNLVGVIDFMDMPGLSDLLRRIIVEQIGAVMVLPNKLPISLSEEVSAVALK--------------MPEPEGILRI
DpersimilisEsyt2var1         LDLFYASDCDINFYLGGMK---GGIKDFQIHGWVRVVMKPLIRSMPLVGGLQIFFLNNPNIDFNLVGVIDFMDMPGLSDLLRRIIVEQIGAVMVLPNKLPISLSEEVSAVALK--------------MPEPEGILRI
DwillistoniEsyt2var1         LDLFYASDCDINFYLGGMK---GGIKDFQIHGWVRVVMKPLIRSMPLVGGLQIFFLNNPNIDFNLVGVIDFMDMPGLSDLLRRIIVEQIGNVMVLPNKLPISLSEEVSAVALK--------------MPEPEGILRI
DvirilisEsyt2var1            LDLFYASDCDINFYLGGMK---GGIKDFQIHGWVRVVMKPLIRSMPLVGGLQIFFLNNPNIDFNLVGVIDFMDMPGLSDLLRRIIVEQIGNVMVLPNKLPISLSEEVSAVALK--------------MPEPEGLLRI
DmojavensisEsyt2var1         LDLFYASDCDINFYLAGMK---GGIKDFQIHGWVRVVMKPLIRSMPLVGGLQIFFLNNPNIDFNLVGVIDFMDMPGLSDLLRRIIVEQIGNVMVLPNKLPISLSDEVSAVSLK--------------MPEPEGLLRI
DgrimshawiEsyt2var1          LDLFYASDCDINFYLGGMK---GGIKDFQIHGWVRVVMKPLIRSMPLVGGLQIFFLNNPNIDFNLVGVIDFMDMPGLSDLLRRIIVEQIGNVMVLPNKLPISLSDEISAVSLK--------------MPEPEGLLRI
Celegansesyt_2var1           MDVAYAGDADFTVSCCGFT---GGMNNIQFSGKLRAILKPLLPYPPMVGGVSGTFLEMPKMDFNLTGMGEMVELPGLIDAIRSVINSQIAALCVLPNEIVVPLAPDVDVTQLY--------------FPEPDGVVRL
Celegansesyt_2var2           MDVAYAGDADFTVSCCGFT---GGMNNIQFSGKLRAILKPLLPYPPMVGGVSGTFLEMPKMDFNLTGMGEMVELPGLIDAIRSVINSQIAALCVLPNEIVVPLAPDVDVTQLY--------------FPEPDGVVRL
Cbrenneriesyt_2var1          MDVAYAGDADFTVSCCGFT---GGMNNIQFSGKLRAILKPLLPYPPMVGGVSGTFLEMPKMDFNLTGMGEMVELPGLIDAIRSVINSQIAALCVLPNEIVVPLAPDVDVTQLY--------------FPEPDGVVRL
Cbrenneriesyt_2var2          MDVAYAGDADFTVSCCGFT---GGMNNIQFSGKLRAILKPLLPYPPMVGGVSGTFLEMPKMDFNLTGMGEMVELPGLIDAIRSVINSQIAALCVLPNEIVVPLAPDVDVTQLY--------------FPEPDGVVRL
Cbriggsaeesyt_2var1          MDVAYAGDADFTVSCCGFT---GGMNNIQFSGKLRAILKPLLPYPPMVGGVSGTFLEMPKMDFNLTGMGEMVELPGLIDAIRSVINSQIAALCVLPNEIVVPLAPDVDVTKLY--------------FPEPDGVVRL
Cbriggsaeesyt_2var2          MDVAYAGDADFTVSCCGFT---GGMNNIQFSGKLRAILKPLLPYPPMVGGVSGTFLEMPKMDFNLTGMGEMVELPGLIDAIRSVINSQIAALCVLPNEIVVPLAPDVDVTKLY--------------FPEPDGVVRL
Cremaneiesyt_2var1           MDVAYAGDADFTVSCCGFT---GGMNNIQFSGKLRAILKPLLPYPPMVGGVSATFLEMPKMDFNLTGMGEMVELPGLIDAIRSVINSQIAALCVLPNEVVVPLAPDVDVTKLY--------------FPEPDGVVRL
Cremaneiesyt_2var2           MDVAYAGDADFTVSCCGFT---GGMNNIQFSGKLRAILKPLLPYPPMVGGVSATFLEMPKMDFNLTGMGEMVELPGLIDAIRSVINSQIAALCVLPNEVVVPLAPDVDVTKLY--------------FPEPDGVVRL
Cjaponicaesyt_2              MDVAYAGDADFAVSCCGFT---GGMNNIQFSGKLRAILKPLLPYPPMVGGVSGTFLEMPKMDFNFTGMGEMVELPGLIDAIRSVINSQIAALCVLPNEIVVPLAPDVDVTQLY--------------FPEPDGVVRF
```

```
                                 400                                                                                           500
TadhaerensEsyt2                 -------------------------------------------------------XFIVHIRYGQSLIVNVQDHDT--VTEDDFLGTVKLDVNFLAQKKRTNTWFPLKNVDKGEVRL------DIVWLSISDSVEDL
NvectensisEsyt2a                EVIEAKDLIAADMALLS-KPTSDPY--CIVEVG-AQKYRTKTKKSNCDPVWKETFEAFIDNTEGQELFCKVYDEDI--AGKDTEIGEVDVQVASAFENGKTDLWLHLEGVEEGRIHL------GLKWFSLSPNPSDL
NvectensisEsyt2b                KVVEARDLVAKDFGVVK-KGKSDPY--AILEIG-AQKFRTKVKKNDLNPTWNETFEAFVDNSEGQDIDMFLWDEDK--AGKDSKLGFLSTQIASAVEQGQRDVWLPLQGAKTGRAHL------HLSWFPLSDQASDL
NvectensisEsyt2cvar1            EVTEAKDLVAKDIAVFK-KGTSDPY--AMVKVG-AQTFRTETKKETLNPKWNEVFEVFVDNSQGQKIKIQLFDEDR--ASDDEALGSVEADISTVVQQGSADLWLPLENVASGQINL------HCTWYTFTNSPDDL
NvectensisEsyt2cvar2            EVTEAKDLVAKDIAVFK-KGTSDPY--AMVKVG-AQTFRTETKKETLNPKWNEVFEVFVDNSQGQKIKIQLFDEDR--ASDDEALGSVEADISTVVQQGSADLWLPLENVASGQINL------HCTWYTFTNSPDDL
CapitellaEsyt2                  QCVAARELKKADISVFG-KGKSDPY--LKVYVG-ATTFKTKCIEDTVNPVWNDYFEAPVDQKYGQFVELECLDKDP--G-DDDELGTASIDIDSVAKTGSMDTWLPLENVKTGMVHV------RALWLHLSKDPEDL
HrobustaEsyt2a                  HMVECKALKSADINIIG-KGKSDPY--CTIGVG-NLIQRTKTIHATLDPVWDQYFQFVVDQTRGQWIDVQVFDEDA-GSTNDDPLGNVTLDIEMVASKKNVDIWLPLSNAPTGAIHM------RALWLAASKKMDDY
HrobustaEsyt2bvar1              KMVEADDLKRADAGKIFGKGKSDPY--AVVTVG-GTTYKTKYIASTLSPRWDEVFEFAVDDVKTQEIEIKVYDHDD--HASDDFIGNTYIRLEEV-EKGHMDEWRRLSNVKTGRVHV------VCDLLYLTFDADD-
HrobustaEsyt2bvar2              KMVEADDLKRADAGKIFGKGKSDPY--AVVTVG-GTTYKTKYIASTLSPRWDEVFEFAVDDVKTQEIEIKVYDHDD--HASDDFIGNTYIRLEEV-EKGHMDEWRRLSNVKTGRVHV------VCDLLYLTFDADD-
LgiganteaEsyt2var1              NIIEAKELKKADIGITG-KGKSDPY--VICSVG-AQKFQTKVIDNTVEPVWNEDYEAIVDVADGQLLTLDVNDRDP--GNDDDHLGGLSVDISQAKSQGIVDEWLPLEDVKKGMVHI------KLTWLYLANDPLEL
LgiganteaEsyt2var2              NIIEAKELKKADIGITG-KGKSDPY--VICSVG-AQKFQTKVIDNTVEPVWNEDYEAIVDVADGQLLTLDVNDRDP--GNDDDHLGGLSVDISQAKSQGIVDEWLPLEDVKKGMVHI------KLTWLYLANDPLEL
CsavignyiEsyt2                  HVIEARKLEEKDKKILGFGGGSDPY--VTVKVGQQHKFTTSIIKNNVNPRWNEVFDALVHDVPTTQIQFALFDDDG-ALNKSDNLGMVSIPVKSVFELGIIDEWVQLSDASTGAIHI------RLEFYELSEDPADL
CintestinalisEsyt2var1          HVIEARNLEEKDKKVLGFGGGSDPY--VTVQVGHRQKFKTAVVTHNLNPVWNEVFDVVVPDVPTTQIQFSLFDDDG-ALNKSDNLGMCSIPVKSVFKQGIIDEWVQLSDVSTGAIHV------RLEFYELSDNPKDL
CintestinalisEsyt2var2          HVIEARNLEEKDKKVLGFGGGSDPY--VTVQVGHRQKFKTAVVTHNLNPVWNEVFDVVVPDVPTTQIQFSLFDDDG-ALNKSDNLGMCSIPVKSVFKQGIIDEWVQLSDVSTGAIHV------RLEFYELSDNPKDL
CintestinalisEsyt2var3          HVIEARNLEEKDKKVLGFGGGSDPY--VTVQVGHRQKFKTAVVTHNLNPVWNEVFDVVVPDVPTTQIQFSLFDDDG-ALNKSDNLGMCSIPVKSVFKQGIIDEWVQLSDVSTGAIHV------RLEFYELSDNPKDL
CintestinalisEsyt2var4          HVIEARNLEEKDKKVLGFGGGSDPY--VTVQVGHRQKFKTAVVTHNLNPVWNEVFDVVVPDVPTTQIQFSLFDDDG-ALNKSDNLGMCSIPVKSVFKQGIIDEWVQLSDVSTGAIHV------RLEFYELSDNPKDL
SpurpuratusEsyt2                TAVEARNLVRADMGLLK-KGKSDPY--LIINVG-MQKFKTKTINNNLNPKWNQTFEALVYEEHGQTLDVDCWDEDP--GSKDDPLGNLSIDIHYISKMGTFDSWLPLEDIKHGDLHL------HLEWLVPSENFDII
BfloridaeEsyt2a                 QLKEAKQLMSADPDFFTKKGKSDPY--CTLHVG-AQFFKSKVIQRTLDPKWNQYFEAVVYEVEGQTMQVNVFDEDP--GVKDDPLGNAAVSIGQVAKEGFTDVWLPLEDATSGQVRL------RMTWLGLSSQREAL
BfloridaeEsyt2b                 HLKEAKELMRADVGFM-KKGKSDPY--CTLQVG-AQSFRSKTIENSLEPRWNEYYEAVVDQLEGQTMQVNMFDEDP--GSKDDPLGNAAVSISEVVKMGFSDMWLPLEDATTGQVHL------RMSWLSLSSQMEAL
IscapularisEsyt2                EVVAAKDLVKADIGMLG-LGKSDPY--AIITVG-AQEFRTQVIPSTVNPKWNFYCEX---------------------------------------------XWLTLDDTKSGKIRL------RTFWLSLTTDTDDL
DpulexEsyt2var1                 RVIEAKQLMKMD-RVLG-IGKSDPY--AIITVG-SQEFRTKTIYNTVNPKWDFYCEYVVSERRSQLCFLRMFDRDE-TGGEDDPLGKATIDIYSIAKVGKKDMWVTLEDVKSGMIHL------ELTWFSLMDDPVML
ApisumEsyt2                     HIFQAKNLVAKDMSLIR-KGKSDPY--VIVTLG-AQQYKTHTINNELNPKWDYWCEFASFSPRGQVLKLKLYDEDEMVGKKHSNLGRASIQIGNVAKTGYFDKWINLEDTKHGMIHV------RMLWLDLTLEQSAL
TcastaneumEsyt2avar1            HVVEAKHLMKKDIGVLG-KGKSDPY--AVVTLG-AQEFKTKVIDNSVDPKWDFWCEFNVLESDGQQLYIHLWDKDE--TSDDETLGRATIEVSNIVKKGQDDLWVTLEQAKHGMVHL------RLTWLTLSDNYSDL
TcastaneumEsyt2b                HVVEAKNLVNRDLI-----GKSDPY--VVLSCG-SIRVETPVVENCLNPKWDFWTNFEI-EPNSE-LKIEVWDKDE--GSKDDSLGHAKINVAQVAKIGQSDMVSTQILVTHILQIILANRLARILKFDKSPKYCTF
TcastaneumEsyt2c                HVFEAKDLMAKDIT-----GKSDPY--VILYVG-AQERKSNTVNQCLNPKWDYWCEFVIIDPKAQHLGFKLYDRDN--VNEDDFLGSGEVDIASVLK-GQTDQWITLDSAKHGAIHL------RFTWLSLSSDLEDL
NvitripennisEsyt2var1           HVVQAKHLMKKDIGMLG-KGKSDPY--AVITVG-AQEFKTKTIDNTVDPKWDYWCECTVTSAIAQQLNIQVWDFDD--TKNDENLGRATIEVSRVKKKGTIDTWVSLELAKHGMVHL------RLVWLKLTTNPADL
AmelliferaEsyt2var1             HVVEAKHLMKKDIGMLG-KGKSDPY--AIINIG-AQEFRTKTIDNTVNPKWDFWCECAVTSAIAQQITVLLWDYDD--TKGDESLGRATIEVSRVKKKGNIDTWISXEQAKHGMIHL------RLTWFQLSKNIVDL
AgambiaeEsyt2var1               HVVEAKDLMKKDISVLG-KGKSDPY--AIISVG-AQQFRTQTIDNTVNPKWDYWCEAFIHAESGQTLQVVINDED---AGEDELLGRATVEISSVTKNGEIDTWLTLEQAKHGLVHL------RMTWFKLSSEKSDL
DmelanogasterEsyt2var1          HVVEAKDLMKKDISVLG-KGKSDPY--AIINVG-AQEFKTQIIDNNVNPKWDYWCEATVFIEMGQFVEIQLKDSDD--SKKDENLGRASIDIASVIKKGVVDSWLTLEDAKHGLLHV------RLQWYKLTADPNDL
DmelanogasterEsyt2var4          -----------------------------------------------------------------------------------------------------------------------------------------
DmelanogasterEsyt2var5          -----------------------------------------------------------------------------------------------------------------------------------------
DmelanogasterEsyt2var6        ● --------------------------------------------------------------MGQFVEIQLKDSDD--SKKDENLGRASIDIASVIKKGVVDSWLTLEDAKHGLLHV------RLQWYKLTADPNDL
DmelanogasterEsyt2var7          HVVEAKDLMKKDISVLG-KGKSDPY--AIINVG-AQEFKTQIIDNNVNPKWDYWCEVRIPWLIPAGIPRRGIPC-------ASCLAFCSLPMLSIDCCMKQSNTKKNHRFIA-------------------------
DmelanogasterEsyt2var8          HVVEAKDLMKKDISVLG-KGKSDPY--AIINVG-AQEFKTQIIDNNVNPKWDYWCEATVFIEMGQFVEIQLKDSDD--SKKDENLGRASIDIASVIKKGVVDSWLTLEDAKHGLLHV------RLQWYKLTADPNDL
DsimulansEsyt2var1              HVVEAKDLMKKDISVLG-KGKSDPY--AIINVG-AQEFKTQIIDNNVNPKWDYWCEATVFIEMGQFVEIQLKDSDD--SKKDENLGRASIDIASVIKKGVVDSWLTLEDAKHGLLHV------RLQWYKLTADPNDL
DsechelliaEsyt2var1             HVVEAKDLMKKDISVLG-KGKSDPY--AIINVG-AQEFKTQIIDNNVNPKWDYWCEATVFIEMGQFVEIQLKDSDD--SKKDENLGRASIDIASVIKKGVVDSWLTLEDAKHGLLHV------RLQWYKLTADPNDL
DerectaEsyt2var1                HVVEAKDLMKKDISVLG-KGKSDPY--AIINVG-AQEFKTQIIDNNVNPKWDYWCEATVFIQMGQFVEIQLRDSDD--SKKDENLGRASIDIASVIKKGVVDSWLTLEDAKHGLLHV------RLQWYKLTADPNDL
DyakubaEsyt2var1                HVVEAKDLMKKDISVLG-KGKSDPY--AIINVG-AQEFKTQIIDNNVNPKWDYWCEATVFIQMGQFVEIQLKDSDD--SKKDENLGRASIDIASVIKKGVVDSWLTLEDAKHGLLHV------RLQWYKLTADPNDL
DananassaeEsyt2var1             HVVEAKDLMKKDISVLG-KGKSDPY--AIINVG-AQEFRTQTIDNNVNPKWDYWCEATVFIEMGQYLEIQLMDKDE-LSKKDESLGRASIDISSVIKKGVVDSWLTLEDAKHGLLHV------RLQWYKLTADPNDL
DpseudoobscuraEsyt2var1         HVVEAKDLMKKDISVLG-KGKSDPY--AIINVG-AQEFKTQIIDNNVNPKWDYWCEAPVFIEMGQFVEIQLKDSDD--SKKDENLGRASIDIASVIKKGVVDSWLTLEDAKHGLLHV------RLQWYKLTADPNDL
DpersimilisEsyt2var1            HVVEAKDLMKKDISVLG-KGKSDPY--AIINVG-AQEFKTQIIDNNVNPKWDYWCEAPVFIEMGQFVEIQLKDSDD--SKKDENLGRASIDIASVIKKGVVDSWLTLEDAKHGLLHV------RLQWYKLTAELNDL
DwillistoniEsyt2var1            HVVEAKDLMKKDISVLG-KGKSDPY--AIINVG-AQEFKTQIIDNNVNPKWDYWCEATVFIEMGQFVEIQLKDSDD--SKKDENLGRASIDIASVIQKGVVDSWLTLEDAKHGLLHV------RLQWYKLTADPNDL
DvirilisEsyt2var1               HVVEAKNLMKKDISVLG-KGKSDPY--AIINVG-AQEFRTQIIDNNVNPKWDYWCEAPVFIEMGQWVDILLKDSDD--SKKDENLGRASIDISSVIKKGVLDTWLTLEDAKHGDLHV------RLQWYRLTADPNDL
DmojavensisEsyt2var1            HVVEAKNLMKKDISVLG-KGKSDPY--AIVNVG-AQEFRTQIIDNNVNPKWDYWCEATVFIEMGQFVNIQLKDSDD--SKQDENLGRATIDISSVIKKGVLDTWLALEDAKHGDLHV------RLQWYKLTADPNDL
DgrimshawiEsyt2var1             HVVEAKNLMKKDISVLG-KGKSDPY--AIINVG-SQEFRTQIIDNNVNPKWDYWCEAPVFIEMGQWVDMQLKDSDD--SKKDESLGRASIDIASVIKKGVLDTWLTLEDAKHGDLHV------RLQWYKLTADPNDL
Celegansesyt_2var1              KIIEAKNLENRDISFIK-KGKSDPY--AEIQVG-SQFFKTRTIDDDLNPIWNEYFEAVVDQADGQKLRIELFDEDQ---GKDEELGRLSVDLKLVQAKGTIDKWYPLEGCKHGDLHI------KATWMNLSTELRHL
Celegansesyt_2var2              KIIEAKNLENRDISFIK-KGKSDPY--AEIQVG-SQFFKTRTIDDDLNPIWNEYFEAVVDQADGQKLRIELFDEDQ---GKDEELGRLSVDLKLVQAKGTIDKWYPLEGCKHGDLHI------KATWMNLSTELRHL
Cbrenneriesyt_2var1             KIIEARNLENRDISFIK-KGKSDPY--AEIQVG-SQFFKTRTIDDDLNPIWNEYFEAVVDQADGQKLRIELFDEDQ---GKDEELGRLSVDLKLVQARGTIDKWYPLEGCKHGDLHI------KATWMDLSTNLKHL
Cbrenneriesyt_2var2             KIIEARNLENRDISFIK-KGKSDPY--AEIQVG-SQFFKTRTIDDDLNPIWNEYFEAVVDQADGQKLRIELFDEDQ---GKDEELGRLSVDLKLVQARGTIDKWYPLEGCKHGDLHI------KATWMDLSTNLKHL
Cbriggsaeesyt_2var1             KVIEAKNLENRDISFIK-KGKSDPY--AEIQVG-SQFFKTRTIDDDLNPIWNEYFEAVVDQADGQKLRIELFDEDQ---GKDEELGRLSVDLKMVQAKGTVDKWYPLEGCKHGDLHL------KATWMSLSTDLKHL
Cbriggsaeesyt_2var2             KVIEAKNLENRDISFIK-KGKSDPY--AEIQVG-SQFFKTRTIDDDLNPIWNEYFEAVVDQADGQKLRIELFDEDQ---GKDEELGRLSVDLKMVQAKGTVDKWYPLEGCKHGDLHL------KATWMSLSTDLKHL
Cremaneiesyt_2var1              KVIEAKNLENRDISFIK-KGKSDPY--AEIQVG-SQFFKTRTIDDDLNPIWNEYFEAVVDQADGQKLRIELFDEDQ---GKDEELGRLSVDLKLVQAKGTIDKWYPLEGCKHGDLHI------KATWMSLSTDLKHL
Cremaneiesyt_2var2              KVIEAKNLENRDISFIK-KGKSDPY--AEIQVG-SQFFKTRTIDDDLNPIWNEYFEAVVDQADGQKLRIELFDEDQ---GKDEELGRLSVDLKLVQAKGTIDKWYPLEGCKHGDLHI------KATWMSLSTDLKHL
Cjaponicaesyt_2                 KIIEAKNLENRDISFIK-KGKSDPY--AEIQVG-SQFFKTRTIDDDLNPIWNEYFEAVVDQADGQKLRIELFDEDQ---GKDEELGRLSVDLKLVRARGTIDKWYPLEGCKHGDLHI------RTTWMNLSTQLKDL
```

```
                                                                                            600
                                      |         |         |         |         |         |         |         |         |         |         |         |         |
TadhaerensEsyt2            -------------QLEAGDCKEPKQQFPHALLLTNVLNG----------KLATERRTYCKVTVGNSTFTSKPTKRKTKVPRWDVQYKFLITNPQKEEAIFEM-----------------------------FEFDSN
NvectensisEsyt2a           R--------------QAT-----PRNPTVAALFVKIINAIDLPKD--MTSIETPTWLLCKVNV-GKTSKDTFQVSSA-APTWSQGLRFLISDPRTQNVKISI-----------------------------LEGGDK
NvectensisEsyt2b           K--------------APQ-----EPHASVAALFVKVVSAESLPQP--HKKAHLKS-VFCEVSI-AEQTNKTFIVYGE-KSEWNQGLRFFVRDPANDEVEVKV-----------------------------IESKGN
NvectensisEsyt2cvar1       L--------------PPEKAVQGEEMLATSALFVKLDSAKNLPVT--NAARGTTS-AFCKLTV-GNKTKNSKTITDSISPVWEEPFRFLIHDPKYQELNIEV-----------------------------FDSEKE
NvectensisEsyt2cvar2       L--------------PPEKAVQGEEMLATSALFVKLDSAKNLPVT--NAARGTTS-AFCKLTV-GNKTKNSKTITDSISPVWEEPFRFLIHDPKYQELNIEV-----------------------------FDSEKE
CapitellaEsyt2             GKT--------EAMNTADTADAEMLLSSA-ILRVSVDSAKALPR---QKKSMGEPSPFARLRV-GNEEKKTSIKLKTTDPRWEESFLFLINNPNQQDLYIDV----------------------------IDSNKGE
HrobustaEsyt2a             K-------------PQSEVTGTVSDTFATSILLVVIDSAKNIP----KSLNTGKPRNTYVKAKLLHDSRRTTIKYGTGDPVWGENFNFFITNPQKDQLELELVHKGSSKETTLATMMVPINSVMLTKNLSLIQEYPL
HrobustaEsyt2bvar1         ------------------PGRIKSRSGSTCLLMVGVESAKDLPS---HSKGLLEPSPCVSLQL-GNKLVKTTPKLRTIHPKWDEVFSFMVTEPDNEILFVQV----------------------------LDTSKND
HrobustaEsyt2bvar2         ------------------PGRIKSRSGSTCLLMVGVESAKDLPSTFSHSKGLLEPSPCVSLQL-GNKLVKTTPKLRTIHPKWDEVFSFMVTEPDNEILFVQV----------------------------LDTSKND
LgiganteaEsyt2var1         DRERKRHRKNQVVQKMEEDRQTDEKFSSC-ILLVNLDSARDLPR---GKKSLNEPSPFCQISV-GTLKKESWVKWNTNEPRWEQNFRFLIHNPNFQNLDVDV----------------------------MDKKTG-
LgiganteaEsyt2var2       ● DR---------VVQKMEEDRQTDEKFSSC-ILLVNLDSARDLPR---GKKSLNEPSPFCQISV-GTLKKESWVKWNTNEPRWEQNFRFLIHNPNFQNLDVDV----------------------------MDKKTG-
CsavignyiEsyt2             I-------------EVLEHSNSDEKIYSS-LLNIYIDGAQNLPD---SCESSYDASPQLTIMMPGRESFKTQTASHTCNPVWEESFNCLVSNPNLDNVSFKI---------------------------TNQHGTKQ
CintestinalisEsyt2var1     K-------------GALNHSHMNDKLFSS-FLNIYVDGAQNLPE---FNQECYDANPQLKITLPGKEPLKTRVAMHTNNPVWEENFHVLISHPELDLVTFQIKRKDGIKSTLMQHSDALRKVLLMKTEPSMENDHGN
CintestinalisEsyt2var2     K-------------GALNHSHMNDKLFSS-FLNIYVDGAQNLPE---FNQECYDANPQLKITLPGKEPLKTRVAMHTNNPVWEENFHVLISHPELDLVTFQIKRKDGIKSTLMQHSDALRKVLLMKTEPSMENDHGN
CintestinalisEsyt2var3   ● K-------------GALNHSHMNDKLFSS-FLNIYVDGAQNLPE---FNQECYDANPQLKITLPGKEPLKTRVAMHTNNPVWEENFHVLISHPELDLVTFQ-----------------------------MENDHGN
CintestinalisEsyt2var4     K-------------GALNHSHMNDKLFSS-FLNIYVDGAQNLPE---FNQECYDANPQLKITLPGKEPLKTRVAMHTNNPVWEENFHVLISHPELDLVTFQIKRKDGIKSTLMQHSDALRKVLLMKTEPSMENDHGN
SpurpuratusEsyt2           HDQ--------VADCIQVSSPTSESLHSCALLVVKLDSAKDLPV---SSRSTSMPSPVCTLKV-GQTMQKSHVQQKTMRPVWEETYHFLVMNPAMQSLDIEV----------------------------TDSKKGN
BfloridaeEsyt2a            EKM--------YTQMENMKRVTDMDDMSSALLFVRVDSASGLPS----KKKVEDMNTYVELTM-GKKHEKSWIQWGTDKPVWGQGFTFLVKDPHSEELLIEI-----------------------------KDEKSK
BfloridaeEsyt2b            DKS--------LDQMEKVKRIADADALSSALLVVRVDSAKNLPT---VKKSGSDPNPYVELSV-AQKHWKTKPQYNTYEPVWEEAFNFLIQNPLHQELLAEV-----------------------------KDEKTK
IscapularisEsyt2           PL-----------QLERVKSISTKTPLSTAVLIVFLDSAKHLPN---ASRAAGEPSPQVQLVL-GHVERWSSIKHSTNDPVWEEIFYLLLANPEVQEMEIKX---------------------------XVVDNKTG
DpulexEsyt2var1            K------------MHAAETQSM---GLSSALLIVYVDSATSLP----SARTSSKPDPYVIVTA-GNRSEQTSARMRTCDPTWEQALVFLVCNPESDDLYLKV----------------------------MDQKTG-
ApisumEsyt2                K------------RALTETQELRITNLSSAVVMVYVDSAINLP----NARAQSKPDPLVRVTV-GQTTQTTVGKLRTERPVYEQGFTFLVSNPETDTIEFKV----------------------------IDQKTN-
TcastaneumEsyt2avar1       K------------AALEETQQLRVTSMSTALLTIFLDSAKNLP----QARASTKPDPYAVLKV-GNTTKETKVLERTIHPVWEQGFSFLVANPESDTLYLTI----------------------------IDRKTT-
TcastaneumEsyt2b           L------------KIMKETELLS-PNLHTALLMIYLESSLNLP----KFSK-TSPNPYAELEV-ENETKTTDPEQQTCEPLWETGFTFLLRDPKKAVLNLRI-----------------------------IDAESK
TcastaneumEsyt2c           D------------EISRETKLLQVDHISTALLTIYVDTATKLP----EAKRLVKPHPYFILTL-RDQKEKSRVKKHTNDPCWEQGFVMLVPNPLEDSLHMAI-----------------------------LDKPTG
NvitripennisEsyt2var1      A------------AALKETQELRVTAMSTAILILYIDSAKNLP----CVKGSKQPDVYLEASV-GGKLERTGTMLRSCNPVWEQGFTLLVANPETGTLHIKI----------------------------HDEKSV-
AmelliferaEsyt2var1        K------------AALVETQELRVTSMSTALLILYIDSAKNLP----CVRGNKQPDVYLEASI-GGNTKRTATMLRSCDPVWEQGFTFLVSNPETGILHIKI----------------------------TDEKTN-
AgambiaeEsyt2var1          K------------QALEETQHLRVTSMSTALLTVFIDSAKNLP----QARQQSQPDPYLVLSV-GKKNEQTSVQMRTDAPVWEQGFTFLVGNPDNDTLQLKV----------------------------IDQKTG-
DmelanogasterEsyt2var1     Q------------QILLETQLLRVTSMSSAVLSVFIDSARHLK----QARSSSKPDPYLVCSV-NKQKQQTAMIMRDDSPVWEQGFTFLVSNPDNESLNIKI----------------------------YDQKTG-
DmelanogasterEsyt2var4   ● --------------------------MSSAVLSVFIDSARHLK----QARSSSKPDPYLVCSV-NKQKQQTAMIMRDDSPVWEQGFTFLVSNPDNESLNIKI-----------------------------YDQKTG
DmelanogasterEsyt2var5     -----------------------------------------------------------------------------------------------------------------------------------------
DmelanogasterEsyt2var6     Q------------QILLETQLLRVTSMSSAVLSVFIDSARHLK----QARSSSKPDPYLVCSV-NKQKQQTAMIMRDDSPVWEQGFTFLVSNPDNESLNIKI-----------------------------YDQKTG
DmelanogasterEsyt2var7     -----------------------------------------------------------------------------------------------------------------------------------------
DmelanogasterEsyt2var8     Q------------QILLETQLLRVTSMSSAVLSVFIDSARHLK----QARSSSKPDPYLVCSV-NKQKQQTAMIMRDDSPVWEQGFTFLVSNPDNESLNIKI----------------------------YDQKTG-
DsimulansEsyt2var1         Q------------QILLETQLLRVTSMSSAVLSVFIDSARHLK----QARSSSKPDPYLVCSV-NKQKQQTAMIMRDDSPVWEQGFTFLVSNPDNESLNIKI----------------------------YDQKTG-
DsechelliaEsyt2var1        Q------------QILLETQLLRVTSMSSAVLSVFIDSARHLK----QARSSSKPDPYLVCSV-NKQKQQTAMIMRDDSPVWEQGFTFLVSNPDNESLNIKI----------------------------YDQKTG-
DerectaEsyt2var1           Q------------QILLETQLLRVTSMSSAVLSVFIDSARHLK----QARSSSKPDPYLVCSV-NKQKQQTAMIMRDDSPVWEQGFTFLVSNPDNESLNIKI----------------------------YDQKTG-
DyakubaEsyt2var1           Q------------QILLETQLLRVTSMSSAVLSVFIDSARHLK----QARSSSKPDPYLVCSV-NKQKQQTAMIMRDDSPVWEQGFTFLVSNPDNECLNLKI----------------------------YDQKTG-
DananassaeEsyt2var1        Q------------QILLETKLLRVTTMSSAVLSVFIDSARHLK----QARSSSKPDPYLVCSV-NKQKQQTAMIMRDDSPVWEQGFTFLVSNPDNESLNIKI----------------------------YDQKTG-
DpseudoobscuraEsyt2var1    Q------------QILLETQLLRVTSMSSAVLSVFIDSARHLK----QARSSSKPDPYLVCSL-NKQKQQTAMIMRDDSPVWEQGFTFLVSNPDNESLNIKI----------------------------YDQKTG-
DpersimilisEsyt2var1       Q------------QILLETQLLRVTSMSSAVLSVFIDSARHLK----QARSSSKPDPYLVCSL-NKQKQQTAMIMRDDSPVWEQGFTFLVSNPDNESLNIKI----------------------------YDQKTG-
DwillistoniEsyt2var1       Q------------QILLETKLLRITTMSSAVLSVFIDSARHLK----QARSSSKPDPYLVCSL-NKQKQQTAMIMRDDSPVWEQGFTFLVSNPDNESLNIKI----------------------------YDQKTG-
DvirilisEsyt2var1          Q------------QILLETQLLRVTTMSAAVLSVFIDSARHLK----QARSNSKPDPYLVCSV-NKQKKQTAMILRDDSPVWEQGFTFLVNNPDNECLNIKI----------------------------YDQKTG-
DmojavensisEsyt2var1       Q------------QILLETQLLRVTTMSSAVLSVFIDSARHLK----QARANSKPDPYLVCSV-NKQKKQTAMILRDDSPVWEQGFTFLVTNPNNESLNIKI----------------------------YDQKTG-
DgrimshawiEsyt2var1        Q------------QILLETKLLRVSTMSSAVLSVFIDSAKHLK----QARANSKPDPYLVCSV-NKQKKQTAMIFRDDSPVWEQGFTFLVSNPENECLNIKI----------------------------YDQKTG-
Celegansesyt_2var1         EK-----------QEWEAEWGQADKPIHSALLMVYIDSVADLP----YPKSKLEPSPFVEVSL-GKETQRTPVKVKTVNPLFQSKFLFFVRHLEGQELKFEA----------------------------VDDGTR-
Celegansesyt_2var2         EK-----------QEWEAEWGQADKPIHSALLMVYIDSVADLP----YPKSKLEPSPFVEVSL-GKETQRTPVKVKTVNPLFQSKFLFFVRHLEGQELKFEA----------------------------VDDGTR-
Cbrenneriesyt_2var1        EK-----------QEWEAEWGQADKPIHSALLMVYIDSVADLP----YPKSKLEPSPFVEVSL-GKEAQRTPVKVKTVNPLFQSKFLFFVRHLEGQELKFEA----------------------------IDDGTR-
Cbrenneriesyt_2var2        EK-----------QEWEAEWGQADKPIHSALLMVYIDSVADLP----YPKSKLEPSPFVEVSL-GKEAQRTPVKVKTVNPLFQSKFLFFVRHLEGQELKFEA----------------------------IDDGTR-
Cbriggsaeesyt_2var1        ER-----------QEWEAEWGQADKPIHSALLMVYIDSVADLP----YPKSKLEPSPFVEVSL-GKETQRTPVKVKTVNPLFQSKFMFFVRHLEGQELKFEA----------------------------VDDGTR-
Cbriggsaeesyt_2var2        ER-----------QEWEAEWGQADKPIHSALLMVYIDSVADLP----YPKSKLEPSPFVEVSL-GKETQRTPVKVKTVNPLFQSKFMFFVRHLEGQELKFEA----------------------------VDDGTR-
Cremaneiesyt_2var1         EK-----------QEWEAEWGQADKPIHSALLMVYIDSVADLP----YPKSKLEPSPFVEVSL-GKETQRTPVKVKTVNPLFQSKFMFFVRHLEGQELKFEA----------------------------VDDGTR-
Cremaneiesyt_2var2         EK-----------QEWEAEWGQADKPIHSALLMVYIDSVADLP----YPKSKLEPSPFVEVSL-GKETQRTPVKVKTVNPLFQSKFMFFVRHLEGQELKFEA----------------------------VDDGTR-
Cjaponicaesyt_2            EK-----------QEWEAEWGQADKPIHSAILMVYIDSVADLP----YPKSKLEPSPFVEVSL-GKETQRTPVKVKTVNPLFQSKFLFFVRHLEGQELKFEA----------------------------VDDGTR-
```

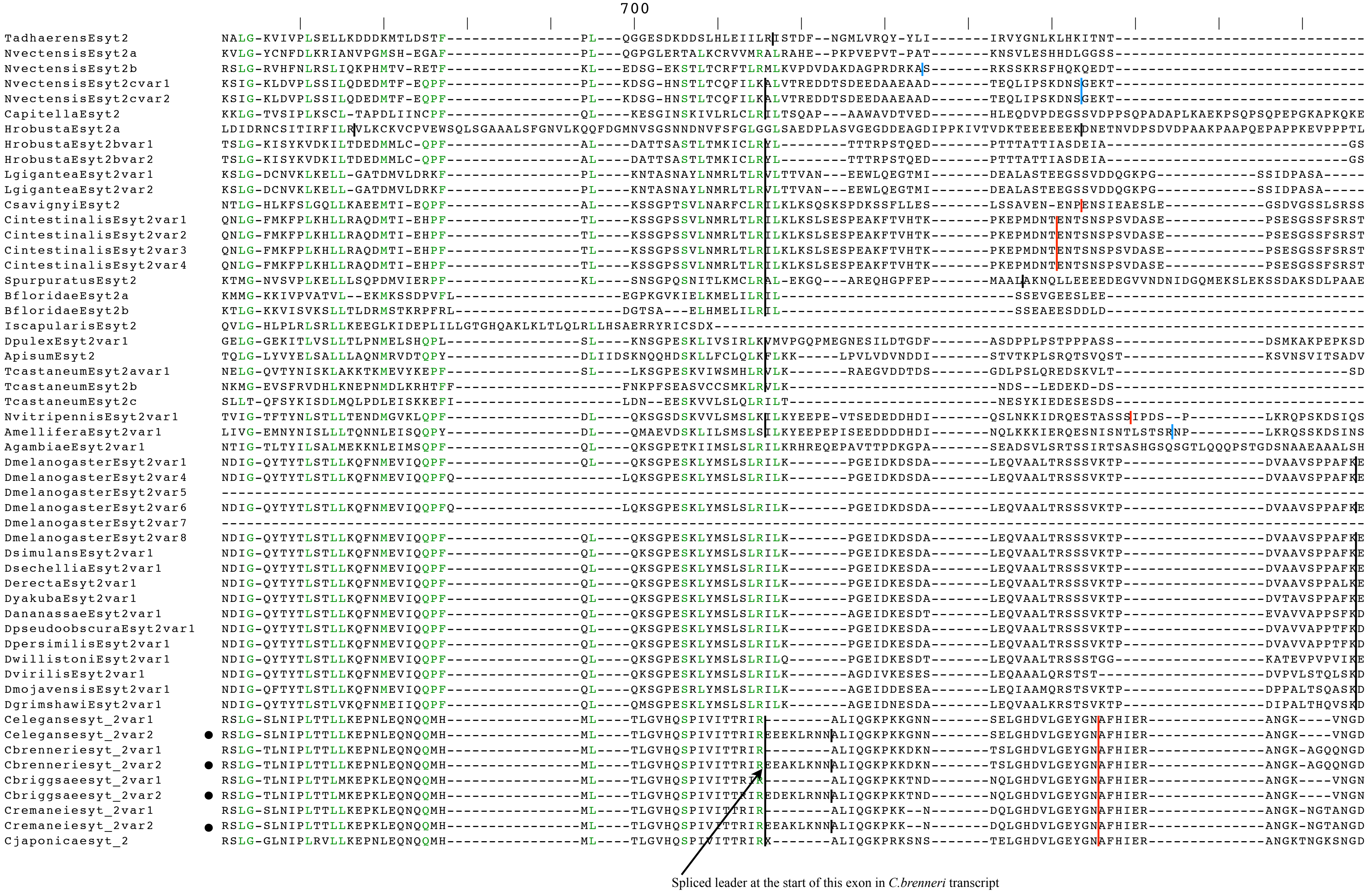
700
Spliced leader at the start of this exon in *C.brenneri* transcript

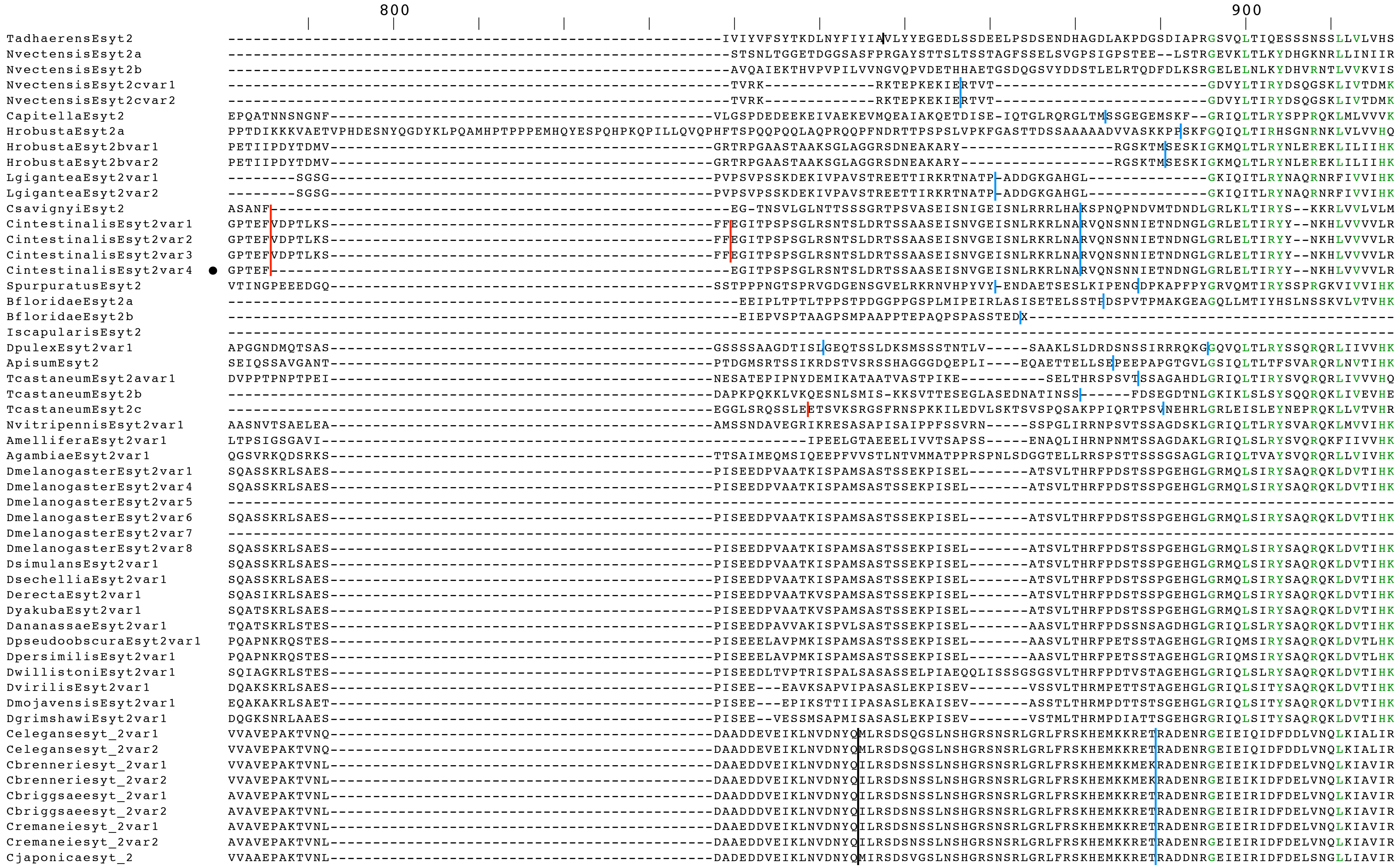
800
900
TadhaerensEsyt2
NvectensisEsyt2a
NvectensisEsyt2b
NvectensisEsyt2cvar1
NvectensisEsyt2cvar2
CapitellaEsyt2
HrobustaEsyt2a
HrobustaEsyt2bvar1
HrobustaEsyt2bvar2
LgiganteaEsyt2var1
LgiganteaEsyt2var2
CsavignyiEsyt2
CintestinalisEsyt2var1
CintestinalisEsyt2var2
CintestinalisEsyt2var3
CintestinalisEsyt2var4
SpurpuratusEsyt2
BfloridaeEsyt2a
BfloridaeEsyt2b
IscapularisEsyt2
DpulexEsyt2var1
ApisumEsyt2
TcastaneumEsyt2avar1
TcastaneumEsyt2b
TcastaneumEsyt2c
NvitripennisEsyt2var1
AmelliferaEsyt2var1
AgambiaeEsyt2var1
DmelanogasterEsyt2var1
DmelanogasterEsyt2var4
DmelanogasterEsyt2var5
DmelanogasterEsyt2var6
DmelanogasterEsyt2var7
DmelanogasterEsyt2var8
DsimulansEsyt2var1
DsechelliaEsyt2var1
DerectaEsyt2var1
DyakubaEsyt2var1
DananassaeEsyt2var1
DpseudoobscuraEsyt2var1
DpersimilisEsyt2var1
DwillistoniEsyt2var1
DvirilisEsyt2var1
DmojavensisEsyt2var1
DgrimshawiEsyt2var1
Celegansesyt_2var1
Celegansesyt_2var2
Cbrenneriesyt_2var1
Cbrenneriesyt_2var2
Cbriggsaeesyt_2var1
Cbriggsaeesyt_2var2
Cremaneiesyt_2var1
Cremaneiesyt_2var2
Cjaponicaesyt_2

```
                                                                                             1000
                                     |         |         |         |         |         |         |         |         |         |         |         |         |
TadhaerensEsyt2             ASNLPA--MNQSGSIDSYVRAYLLPDR--GKDDRKRTSTVNSDQNPAFGETFEFPVTYEEAKSRTLEVAVKSVTPNKT---------VGRVLINLSTVNIAAT--TTAWYALSPP--------------------
NvectensisEsyt2a            AERLRS--RYPENKTNPFVRLYLLPDR--TKKTRRRTGAVRGSLAPSFNETIEYCVGLDQLKDRDLDIEVKNARPGLSMRRGWNS--IGRTTIQLSELSLSAG--ITMTCELKK---------------------
NvectensisEsyt2b            ARNLRS--GDAENKTNPFVRLYLLPDR--SKKTRRVTKTVRGAYSGMFNEVFEYAIGLDLLGDRQLACAVKSDR-GLRNRL------IGETTVDLGELELTDG--VTLHRDLIKMH-------------------
NvectensisEsyt2cvar1        ARDLMP--CDSDGLADPYMRSYVLPDK--SKSNRRRTDIAKNTLSPSFDEKFEWMIPEAQLKDRTLDVTVKNDVSFFSKSK--TS--MGQVLIDLGKLDLSKP--ISAWYMLRDEKDDE----------------
NvectensisEsyt2cvar2        ARDLMP--CDSDGLADPYMRSYVLPDK--SKSNRRRTDIAKNTLSPSFDEKFEWMIPEAQLKDRTLDVTVKNDVSFFSKSK--TS--MGQVLIDLGKLDLSKP--ISAWYMLRDEKDDE----------------
CapitellaEsyt2              CMNLIP--CDEDNLADPYVRIYMNPEK-S----KRKTQIIKNNLNPIFDETFEWDVTMQELALKTLEISVKNKN-SM-FSTKREH--MGQLTLALGQTDLSKA--LTQWYDLE--DPDSK---------------
HrobustaEsyt2a              CKNLIP--CDKDNLADPYVRMYLLPDR--TADTKRKTKLVKNTLNPTFDETFEWDLGVMEFPFRTLEVAVKNDV-GV-FSKSRTD--MGVVAVELAKLGNLSSA-LTQWFDLEDPKGH-----------------
HrobustaEsyt2bvar1          CINLVA--YDKDNFSDPYVRMYLLPDR--SNASKRKTNVMKNNLNPVWDETFEWPLKKFELPNRTLEIMVKNFI-GVFEKKTQTE--MGVVNLRLSDFDDLAQP-VSDWFDLQ---DPTSLTASISTVL------
HrobustaEsyt2bvar2          CINLVA--YDKDNFSDPYVRMYLLPDR--SNASKRKTNVMKNNLNPVWDETFEWPLKKFELPNRTLEIMVKNFI-GVFEKKTQTE--MGVVNLRLSDFDDLAQP-VSDWFDLQ---DPTSLTASISTVL------
LgiganteaEsyt2var1          CTNLLA--LDSNNLSDPYIRLYLLPDK-SSDS-KRKTKVIKDNLNPVFDETLEFPVSPSDVKLKLLEVAIKNKN-SL-FSSSKKM--MGIVTIDLANFDIYKA--LTDWYDLQ--PEDSDERVSLETEI------
LgiganteaEsyt2var2          CTNLLA--LDSNNLSDPYIRLYLLPDK-SSDS-KRKTKVIKDNLNPVFDETLEFPVSPSDVKLKLLEVAIKNKN-SL-FSSSKKM--MGIVTIDLANFDIYKA--LTDWYDLQ--PEDSDERVSLETEI------
CsavignyiEsyt2              AENLIA-CDED--TSDPYVRIYIIPDK---RT-RKKTKVVKRDLLPVWDQRLEFDIPKKEVKQKKLHISVKNQT-GF-LSSEKVL--MGQVIIDLRELDLLQP--NTEWYXXQLAA-------------------
CintestinalisEsyt2var1      AANLIV-CDDDEKTSDPYVRVYILPDK---RS-RKKTKVIKNNLNPVWDQRLEFDVSKSEVMHKKLHVSVKNQT-GF-LSSEKVL--MGQVIVDLSKLDLHQP--TTEWYNLQVAT-------------------
CintestinalisEsyt2var2      AANLIV-CDDDEKTSDPYVRVYILPDK---RS-RKKTKVIKNNLNPVWDQRLEFDVSKSEVMHKKLHVSVKNQT-GF-LSSEKVL--MGQVIVDLSKLDLHQP--TTEWYNLQVAT-------------------
CintestinalisEsyt2var3      AANLIV-CDDDEKTSDPYVRVYILPDK---RS-RKKTKVIKNNLNPVWDQRLEFDVSKSEVMHKKLHVSVKNQT-GF-LSSEKVL--MGQVIVDLSKLDLHQP--TTEWYNLQVAT-------------------
CintestinalisEsyt2var4      AANLIV-CDDDEKTSDPYVRVYILPDK---RS-RKKTKVIKNNLNPVWDQRLEFDVSKSEVMHKKLHVSVKNQT-GF-LSSEKVL--MGQVIVDLSKLDLHQP--TTEWYNLQVAT-------------------
SpurpuratusEsyt2            ASQLTI--PDSEDMPDSYIRAYLLPDK-SKSG-KQKTKVIKDTRDPVFDHTFEFSCTSTELSERVLDICIKNSH-SF-LPLNNPT--IGQVDIDLATLDLSKA--TTEWYNLKRISPDSVSRLSMSFTS------
BfloridaeEsyt2a             ARNLLP--PRGSKICDAYVRMRFCAGQ--KKSEIKKTGVVKQNLNPAWDETLGFPASLAEAKQGRLEVAVKHKRSLLKSFRGTDAWFLGEVEVDLSKVERLEIKGQPQWFDLKRDAGAKLSRYASFMSTRSGAES
BfloridaeEsyt2b             ---------------------------------------------------------------------------------------------------------------------------------------
IscapularisEsyt2            -XNLAH--KEGDDLPDPYVKLHLLPDK--LKENKRKTQTCRNSCNPVFDETX-----------------------------------------------------------------------------------
DpulexEsyt2var1             VSQLPG--GDQPDPPDPYVKLYLLPDR-STNS-KRKTEIVKDTVNPVFDETFEYTVAPIDLPSRELEVSVINRK-GR-FA-RSPL--MCSCVVILGHHDLTQA--VTQWFEMK--PSE-----------------
ApisumEsyt2                 VVNLPI--KEASDIPDPYVKLYVLPKKENSNT-KRKTETYKDNCNPVYEETFEYIMGVAELNSKQLEVTVLTKK-TW----HSPV--LGQIVLNISDYVNVNTPSFTGWFDLE--TEVKEGTVG-----------
TcastaneumEsyt2avar1        IANIPL--KDPSNIPDPYVKLYLLPER-AKDT-KRKTHVVKDNCNPIFDESFEYILSQGELNTKQLEVTVASQK-QL-FYSSSNI--LGMVIIDFEKLNVSQP--YNAWFDLT--PESDRNHR------------
TcastaneumEsyt2b            VDLVRK--R-------AQIYVKLYLQT-DKQRNHRKKTKIAKTKDAVFNESFDYLISNADLNWTNLLVMVKTDKGLLKKS-------LGRTVISLQACGNLTEP-FTDWFDLRSKHGEHVYHLIK----------
TcastaneumEsyt2c            VSNLPL--KDPSDIPDPYVRIKMYSQG-HTTGPTYRTKVVTDNCNPVYEETFEYLFSKSDAYEQTLVATVKSKKFLHNNT-------MGQVEINLKYV-NLSER-YREWFDLCPKSS------------------
NvitripennisEsyt2var1       VANLPLPANDPSNIPDPYVKLYLLPDK-HKET-KRKTAVMKDNCNPTFDEQFEYIVSQGDINTRILELSVCTQK-GW-LSTGSNC--MGQVLINLSELDFTQA--VTSWYDLQSESKD-----------------
AmelliferaEsyt2var1         IANLPLPQNDPHNIPDPYVKLYLLPDR-HKET-KRKTAVMKDNCNPIFDEQFEYVVSQADLNSRILEVSVCTQK-GW-LSTGSNV--MGQVHINLNEIDVTKS--FTSWYDLQPETKD-----------------
AgambiaeEsyt2var1           INNIPL--KDPNNIPDPYVKLYLLPGR-SKES-KRKTNVVKDNCDPVFDTTFEYIISNAELVNSELEVTVCTQK-GF-F--GSPV--IGMQKLSLSDPDISSGQGIKAWYDLLPESKFE----------------
DmelanogasterEsyt2var1      IQKIPL--RDPSNIPDPYVKLYLLPGR-TKES-KRKTSVIKDNCNPVYDASFEYLISIAELRQTELEVTVCTQK-GF-LSGGSPI--IGMLKIPLDDAEITTQTGLNSWFDLQPEIRHE----------------
DmelanogasterEsyt2var4      IQKIPL--RDPSNIPDPYVKLYLLPGR--TKESKRKTSVIKDNCNPVYDASFEYLISIAELRQTELEVTVCTQKGFL--SGGSPI--IGMLKIPLDDAEITTQTGLNSWFDLQPEIRHE----------------
DmelanogasterEsyt2var5  ●   --KIPL--RDPSNIPDPYVKLYLLPGR--TKESKRKTSVIKDNCNPVYDASFEYLISIAELRQTELEVTVCTQKGFL--SGGSPI--IGMLKIPLDDAEITTQTGLNSWFDLQPEIRHE----------------
DmelanogasterEsyt2var6      IQKIPL--RDPSNIPDPYVKLYLLPGR--TKESKRKTSVIKDNCNPVYDASFEYLISIAELRQTELEVTVCTQKGFL--SGGSPI--IGMLKIPLDDAEITTQTGLNSWFDLQPEIRHE----------------
DmelanogasterEsyt2var7      ---------------------------------------------------------------------------------------------------------------------------------------
DmelanogasterEsyt2var8      IQKIPL--RDPSNIPDPYVKLYLLPGR-TKES-KRKTSVIKDNCNPVYDASFEYLISIAELRQTELEVTVCTQK-GF-LSGGSPI--IGMLKIPLDDAEITTQTGLNSWFDLQPEIRHE----------------
DsimulansEsyt2var1          IQKIPL--RDPSNIPDPYVKLYLLPGR-TKES-KRKTSVIKDNCNPVYDASFEYLISIAELRQTELEVTVCTQK-GF-LSGGSPI--IGMLKIPLDDAEITTQTGLNSWFDLQPEIRHE----------------
DsechelliaEsyt2var1         IQKIPL--RDPSNIPDPYVKLYLLPGR-TKES-KRKTSVIKDNCNPVYDASFEYLISIAELRQTELEVTVCTQK-GF-LSGGSPI--IGMLKIPLDDAEITTQTGLNSWFDLQPEIRHE----------------
DerectaEsyt2var1            IQKIPL--RDPSNIPDPYVKLYLLPGR-TKES-KRKTSVIKDNCNPVYDASFEYLISIAELRQTELEVTVCTQK-GF-LSGGSPI--IGMLKIPLDDAEITTQTGLNSWFDLQPEIRHE----------------
DyakubaEsyt2var1            IQKIPL--RDPSNIPDPYVKLYLLPGR-TKES-KRKTSVIKDNCNPVYDASFEYLISIAELRQTELEVTVCTQK-GF-LSGGSPI--IGMLKIPLDDAEITTQTGLNSWFDLQPEIRHE----------------
DananassaeEsyt2var1         IAKIPL--RDPSNIPDPYVKLYLLPGR-SKES-KRKTSVIKDNCNPVYDASFEYLISIAELRQTQLEVTVCTQK-GF-LSGSSPI--IGMLKIPLDDSEITTQSGLNSWFDLQPEMRHD----------------
DpseudoobscuraEsyt2var1     IVKIPL--RDPSNIPDPYVKLYLLPGR-TKES-KRKTGVIKDNCNPVYDASFEYLISIAELRQTELEVTVCTQK-GF-LSGSSPI--IGMLKIPLDDSEITTPAGLNTWFDLQPEMRHE----------------
DpersimilisEsyt2var1        IVKIPL--RDPSNIPDPYVKLYLLPGR-TKES-KRKTGVIKDNCNPVYDASFEYLISIAELRQTELEVTVCTQK-GF-LSGSSPI--IGMLKIPLDDSEITTPAGLNTWFDLQPEMRHE----------------
DwillistoniEsyt2var1        IAKIPL--RDPSNIPDPYVKLHLLPGR-SKES-KRKTSVIKDNCNPVYDASFEYLISIAELRQTALEVTVCTQK-GF-LSGGSPI--IGMLKIPLDDSEITTQTGLNTWFDLQPEMRHE----------------
DvirilisEsyt2var1           IMNIPL--RDPSSIPDPYVKLYLLPGR-SKES-KRKTSIIKDNCNPVYDASFEYLISIAELRHTALEVTVCTQK-GF-LSSGSPI--IGMLKLPLDDSEITTPAGLKSWFDLQPELKHE----------------
DmojavensisEsyt2var1        IMNIPL--RDPSSIPDPYVKLYLLPGR-SKES-KRKTSVIKDNCNPVYDASFEYLISIAELRHTELEVTVCTQK-GF-LSSGSPI--IGMLKIPLDDSEITTPAGLKSWFDLQPEQKHE----------------
DgrimshawiEsyt2var1         IMNIPL--RDPSNIPDPYVKLYLLPAR-SKES-KRKTGVIKDNCNPVYDASFEYLISISELRHTELEVTVCSQK-GF-LSHGSPI--IGMLKVQLDDSEITTPIGLKTWFDLEPESKHE----------------
Celegansesyt_2var1          CRDLMT--FDKKDQCNPYVSVKLVALDGNKEVFKKKTPTAKNTRHPHFDNHVEIDINPSDLLNHKVVINVKDDT-NYGTFVAKPV--LGCLEIRLDSLMNRQL--SQRWIPLSVERK------------------
Celegansesyt_2var2          CRDLMT--FDKKDQCNPYVSVKLVALDGNKEVFKKKTPTAKNTRHPHFDNHVEIDINPSDLLNHKVVINVKDDT-NYGTFVAKPV--LGCLEIRLDSLMNRQL--SQRWIPLSVERK------------------
Cbrenneriesyt_2var1         CRDLMT--FDKKDQCNPYVSVKLVALDGHKEVFKKKTPTAKNTRNPHFDNHVEIDVNPSDLLNHKVVINVKDDT-NYGTFVAKPV--LGAVEIRLDSLLNRQL--AQRWIPLSAERK------------------
Cbrenneriesyt_2var2         CRDLMT--FDKKDQCNPYVSVKLVALDGHKEVFKKKTPTAKNTRNPHFDNHVEIDVNPSDLLNHKVVINVKDDT-NYGTFVAKPV--LGAVEIRLDSLLNRQL--AQRWIPLSAERK------------------
Cbriggsaeesyt_2var1         CRDLMT--FDKKDQCNPYVSVKLVALDGHKEVFKKKTPTAKNTRNPHFDNHVEIDVNPSDLLNHKVVINVKDDT-NYGTFVAKPV--LGCLEIRLDSLLNRQL--SQRWIPLSVERK------------------
Cbriggsaeesyt_2var2         CRDLMT--FDKKDQCNPYVSVKLVALDGHKEVFKKKTPTAKNTRNPHFDNHVEIDVNPSDLLNHKVVINVKDDT-NYGTFVAKPV--LGCLEIRLDSLLNRQL--SQRWIPLSVERK------------------
Cremaneiesyt_2var1          CRDLMT--FDKKDQCNPYVSIKLVALDGHKEVFKKKTPTAKNTRNPHFDNHVEIDVNPSDLLNHKVVINVKDDT-NYGTFVAKPV--LGCLEIRLDSLLNRQL--SQRWIPLSVERK------------------
Cremaneiesyt_2var2          CRDLMT--FDKKDQCNPYVSIKLVALDGHKEVFKKKTPTAKNTRNPHFDNHVEIDVNPSDLLNHKVVINVKDDT-NYGTFVAKPV--LGCLEIRLDSLLNRQL--SQRWIPLSVERK------------------
Cjaponicaesyt_2             CRDLMT--FDKKDVCNPYVSVKMVALDGNKEVFKKKTPTAKNTRNPHFDNHVEIDVNPSDLLNHKVVLNVKDDT-NYGTFAAKPI--LGSLEIRLDSLLNRQL--AQRWIPLSVERK------------------
```
