# Supplementary material for: A manual collection of Syt, Esyt, Rph3a, Rph3al, Doc2, and Dblc2 genes from 46 metazoan genomes - an open access resource for neuroscience and evolutionary biology
Source: BMC Genomics. 2010 Jan 15;11:37. doi: 10.1186/1471-2164-11-37 (PMC2823689; doi:10.1186/1471-2164-11-37)
Supplement: Additional file 43 — Alignment of the mutually exclusive alternative Esyt2 exons. In addition to the alternatively coded exon which is highlighted, the exon upstream and the exon downstream are shown. Intron position and phase is indicated with a coloured bar between amino acids. Black bars indicate phase 0 introns. Blue bars indicate phase +2 introns. [file 1471-2164-11-37-S43.PDF]

```
                                                                                                        100
DpulexEsyt2var1              GVLRVRVIEAKQLMKMD-RVLGIGKSDPYAIITVGSQEFRTKTIYNTVNPKWDFYCEYVVSERRSQL---CFLRMFDRDETGG---EDDPLGKATIDIYSIAKVGKKDM
TcastaneumEsyt2avar1         GVLRVHVVEAKHLMKKDIGVLGKGKSDPYAVVTLGAQEFKTKVIDNSVDPKWDFWCEFNVLESDGQQ---LYIHLWDKDETS----DDETLGRATIEVSNIVKKGQDDL
NvitripennisEsyt2var1        GVLRIHVVQAKHLMKKDIGMLGKGKSDPYAVITVGAQEFKTKTIDNTVDPKWDYWCECTVTSAIAQQ---LNIQVWDFDDT----KNDENLGRATIEVSRVKKKGTIDT
AmelliferaEsyt2var1          GVLRIHVVEAKHLMKKDIGMLGKGKSDPYAIINIGAQEFRTKTIDNTVNPKWDFWCECAVTSAIAQQ---ITVLLWDYDDT----KGDESLGRATIEVSRVKKKGNIDT
AgambiaeEsyt2var1            GVLRIHVVEAKDLMKKDISVLGKGKSDPYAIISVGAQQFRTQTIDNTVNPKWDYWCEAFIHAESGQT---LQVVINDED-----AGEDELLGRATVEISSVTKNGEIDT
DmelanogasterEsyt2var1       GILRIHVVEAKDLMKKDISVLGKGKSDPYAIINVGAQEFKTQIIDNNVNPKWDYWCEATVFIEMGQF---VEIQLKDSDD----SKKDENLGRASIDIASVIKKGVVDS
DsimulansEsyt2var1           GILRIHVVEAKDLMKKDISVLGKGKSDPYAIINVGAQEFKTQIIDNNVNPKWDYWCEATVFIEMGQF---VEIQLKDSDD----SKKDENLGRASIDIASVIKKGVVDS
DsecheliaEsyt2var1           GILRIHVVEAKDLMKKDISVLGKGKSDPYAIINVGAQEFKTQIIDNNVNPKWDYWCEATVFIEMGQF---VEIQLKDSDD----SKKDENLGRASIDIASVIKKGVVDS
DerectaEsyt2var1             GILRIHVVEAKDLMKKDISVLGKGKSDPYAIINVGAQEFKTQIIDNNVNPKWDYWCEATVFIQMGQF---VEIQLRDSDD----SKKDENLGRASIDIASVIKKGVVDS
DyakubaEsyt2var1             GILRIHVVEAKDLMKKDISVLGKGKSDPYAIINVGAQEFKTQIIDNNVNPKWDYWCEATVFIQMGQF---VEIQLKDSDD----SKKDENLGRASIDIASVIKKGVVDS
DananassaeEsyt2var1          GILRIHVVEAKDLMKKDISVLGKGKSDPYAIINVGAQEFRTQTIDNNVNPKWDYWCEATVFIEMGQY---LEIQLMDKDEL---SKKDESLGRASIDISSVIKKGVVDS
DpseudoobscuraEsyt2var1      GILRIHVVEAKDLMKKDISVLGKGKSDPYAIINVGAQEFKTQIIDNNVNPKWDYWCEAPVFIEMGQF---VEIQLKDSDD----SKKDENLGRASIDIASVIKKGVVDS
DpersimilisEsyt2var1         GILRIHVVEAKDLMKKDISVLGKGKSDPYAIINVGAQEFKTQIIDNNVNPKWDYWCEAPVFIEMGQF---VEIQLKDSDD----SKKDENLGRASIDIASVIKKGVVDS
DwillistoniEsyt2var1         GILRIHVVEAKDLMKKDISVLGKGKSDPYAIINVGAQEFKTQIIDNNVNPKWDYWCEATVFIEMGQF---VEIQLKDSDD----SKKDENLGRASIDIASVIQKGVVDS
DvirilisEsyt2var1            GLLRIHVVEAKNLMKKDISVLGKGKSDPYAIINVGAQEFRTQIIDNNVNPKWDYWCEAPVFIEMGQW---VDILLKDSDD----SKKDENLGRASIDISSVIKKGVLDT
DmojavensisEsyt2var1         GLLRIHVVEAKNLMKKDISVLGKGKSDPYAIVNVGAQEFRTQIIDNNVNPKWDYWCEATVFIEMGQF---VNIQLKDSDD----SKQDENLGRATIDISSVIKKGVLDT
DgrimshawiEsyt2var1          GLLRIHVVEAKNLMKKDISVLGKGKSDPYAIINVGSQEFRTQIIDNNVNPKWDYWCEAPVFIEMGQW---VDMQLKDSDD----SKKDESLGRASIDIASVIKKGVLDT

DpulexEsyt2var2              GVLRVRVIEAKQLMKMD-RVLGIGKSDPYAIITVGSQEFRTKTIYNTVNPKWDFYCEAKVESLRAQN---CFIQVWDYDAGFPGIQNDDYLGRATIDIYSIAKVGKKDM
TcastaneumEsyt2avar2         GVLRVHVVEAKHLMKKDIGVLGKGKSDPYAVVTLGAQEFKTKVIDNSVDPKWDFWCEAQISSYESQL---LTVNVWDWDPGVPGAQNHDYLGRATIEVSNIVKKGQDDL
NvitripennisEsyt2var2        GVLRIHVVQAKHLMKKDIGMLGKGKSDPYAVITVGAQEFKTKTIDNTVDPKWDYWCEAVICSVIRQE---VQLTVWDWDPNVPGVQLDDFLGRATIEVSRVKKKGTIDT
AmelliferaEsyt2var2          GVLRIHVVEAKHLMKKDIGMLGKGKSDPYAIINIGAQEFRTKTIDNTVNPKWDFWCEAVICSCIMQN---IMVFLWDRDVL--TIPYDDFLGRATIEVSRVKKKGNIDT
AgambiaeEsyt2var2            GVLRIHVVEAKDLMKKDISVLGKGKSDPYAIISVGAQQFRTQTIDNTVNPKWDYWCEAEVNAILRQE---IELNLWDYDPGFPGVQNDDFLGRATVEISSVTKNGEIDT
DmelanogasterEsyt2var2       GILRIHVVEAKDLMKKDISVLGKGKSDPYAIINVGAQEFKTQIIDNNVNPKWDYWCEACIFTTIGHY---IGFSLWDYDQTMPGVQSDDVLGRASIDIASVIKKGVVDS
DsimulansEsyt2var2           GILRIHVVEAKDLMKKDISVLGKGKSDPYAIINVGAQEFKTQIIDNNVNPKWDYWCEACIFTTIGHY---IGFSLWDYDQTMPGVQSDDVLGRASIDIASVIKKGVVDS
DsechelliaEsyt2var2          GILRIHVVEAKDLMKKDISVLGKGKSDPYAIINVGAQEFKTQIIDNNVNPKWDYWCEACIFTTIGHY---IGFSLWDYDQTMPGVQSDDVLGRASIDIASVIKKGVVDS
DerectaEsyt2var2             GILRIHVVEAKDLMKKDISVLGKGKSDPYAIINVGAQEFKTQIIDNNVNPKWDYWCEACIFTTIGHY---IGFSLWDYDQTMPGVQSDDVLGRASIDIASVIKKGVVDS
DyakubaEsyt2var2             GILRIHVVEAKDLMKKDISVLGKGKSDPYAIINVGAQEFKTQIIDNNVNPKWDYWCEACIFTTIGHY---IGFSLWDYDQTMPGVQSDDVLGRASIDIASVIKKGVVDS
DananassaeEsyt2var2          GILRIHVVEAKDLMKKDISVLGKGKSDPYAIINVGAQEFRTQTIDNNVNPKWDYWCEACIFTTIGHY---IGFSLWDYDQTMPGVQNDDELGRASIDISSVIKKGVVDS
DpseudoobscuraEsyt2var2      GILRIHVVEAKDLMKKDISVLGKGKSDPYAIINVGAQEFKTQIIDNNVNPKWDYWCEACIFTTIGHL---IGFSLWDYDQTMPGVLNDDVLGRASIDIASVIKKGVVDS
DpersimilisEsyt2var2         GILRIHVVEAKDLMKKDISVLGKGKSDPYAIINVGAQEFKTQIIDNNVNPKWDYWCEACIFTTIGHL---IGFSLWDYDQTMPGVLNDDVLGRASIDIASVIKKGVVDS
DwillistoniEsyt2var2         GILRIHVVEAKDLMKKDISVLGKGKSDPYAIINVGAQEFKTQIIDNNVNPKWDYWCEACIFTTIGHF---VGFSLWDYDQTMPGVLADDLLGRASIDIASVIQKGVVDS
DvirilisEsyt2var2            GLLRIHVVEAKNLMKKDISVLGKGKSDPYAIINVGAQEFRTQIIDNNVNPKWDYWCEACIFTTINHL---VGFSLWDYDQSMPGVQADDVLGRASIDISSVIKKGVLDT
DmojavensisEsyt2var2         GLLRIHVVEAKNLMKKDISVLGKGKSDPYAIVNVGAQEFRTQIIDNNVNPKWDYWCEAGIFTGINHF---VGFSLWDYDQSMPGVQADDVLGRATIDISSVIKKGVLDT
DgrimshawiEsyt2var2          GLLRIHVVEAKNLMKKDISVLGKGKSDPYAIINVGSQEFRTQIIDNNVNPKWDYWCEASIFTTINHL---VGFSLWDYDQNMPGVNSDDVLGRASIDIASVIKKGVLDT

TcastaneumEsyt2avar3         GVLRVHVVEAKHLMKKDIGVLGKGKSDPYAVVTLGAQEFKTKVIDNSVDPKWDFWCESILESTKSTW---LHLHLYDKD----KGHRDDDLGRATIEVSNIVKKGQDDL
NvitripennisEsyt2var3        GVLRIHVVQAKHLMKKDIGMLGKGKSDPYAVITVGAQEFKTKTIDNTVDPKWDYWCECIMDEGSGPC-SKVIAHLFDKD----TTGPDDPLGRATIEVSRVKKKGTIDT
AmelliferaEsyt2var3          GVLRIHVVEAKHLMKKDIGMLGKGKSDPYAIINIGAQEFRTKTIDNTVNPKWDFWCEFIVEKSLGAYYNTVVAHLFDKD----NAGQDDPLGRATIEVSRVKKKGNIDT
AgambiaeEsyt2var3            GVLRIHVVEAKDLMKKDISVLGKGKSDPYAIISVGAQQFRTQTIDNTVNPKWDYWCEACVDVTHQTL---IGIKLFDWD----RTGDHDPLGRATVEISSVTKNGEIDT
DmelanogasterEsyt2var3       GILRIHVVEAKDLMKKDISVLGKGKSDPYAIINVGAQEFKTQIIDNNVNPKWDYWCEAVVEVSQHAI---LVLRLFDWD----RTSDDESLGRASIDIASVIKKGVVDS
DsimulansEsyt2var3           GILRIHVVEAKDLMKKDISVLGKGKSDPYAIINVGAQEFKTQIIDNNVNPKWDYWCEAVVEVSQHAI---LVLRLFDWD----RTSDDESLGRASIDIASVIKKGVVDS
DsechelliaEsyt2var3          GILRIHVVEAKDLMKKDISVLGKGKSDPYAIINVGAQEFKTQIIDNNVNPKWDYWCEAVVEVSQHAI---LVLRLFDWD----RTSDDESLGRASIDIASVIKKGVVDS
DerectaEsyt2var3             GILRIHVVEAKDLMKKDISVLGKGKSDPYAIINVGAQEFKTQIIDNNVNPKWDYWCEAVVEVSQHAI---LVLRLFDWD----RTSDDESLGRASIDIASVIKKGVVDS
DyakubaEsyt2var3             GILRIHVVEAKDLMKKDISVLGKGKSDPYAIINVGAQEFKTQIIDNNVNPKWDYWCEAVVEVSQHAI---LVLRLFDWD----RTSDDESLGRASIDIASVIKKGVVDS
DananassaeEsyt2var3          GILRIHVVEAKDLMKKDISVLGKGKSDPYAIINVGAQEFRTQTIDNNVNPKWDYWCEAVVEVSQHAI---LVLRLFDWD----RTSDDESLGRASIDISSVIKKGVVDS
DpseudoobscuraEsyt2var3      GILRIHVVEAKDLMKKDISVLGKGKSDPYAIINVGAQEFKTQIIDNNVNPKWDYWCEAAVEVSQHAI---LVLRLFDWD----RTSDDESLGRASIDIASVIKKGVVDS
DpersimilisEsyt2var3         GILRIHVVEAKDLMKKDISVLGKGKSDPYAIINVGAQEFKTQIIDNNVNPKWDYWCEAAVEVSQHAI---LVLRLFDWD----RTSDDESLGRASIDIASVIKKGVVDS
DwillistoniEsyt2var3         GILRIHVVEAKDLMKKDISVLGKGKSDPYAIINVGAQEFKTQIIDNNVNPKWDYWCEAVVEVSQHAM---LVLRLFDWD----RTSDDESLGRASIDIASVIQKGVVDS
DvirilisEsyt2var3            GLLRIHVVEAKNLMKKDISVLGKGKSDPYAIINVGAQEFRTQIIDNNVNPKWDYWCEAVVEVSQRAI---LVLRLFDWD----RTSDDESLGRASIDISSVIKKGVLDT
DmojavensisEsyt2var3         GLLRIHVVEAKNLMKKDISVLGKGKSDPYAIVNVGAQEFRTQIIDNNVNPKWDYWCEAVVEVSQRAI---LVLRLFDWD----RTSDDESLGRATIDISSVIKKGVLDT
DgrimshawiEsyt2var3          GLLRIHVVEAKNLMKKDISVLGKGKSDPYAIINVGSQEFRTQIIDNNVNPKWDYWCEVVVEVSQRSI---LVLRLFDWD----RTSDDESLGRASIDIASVIKKGVLDT
```
