# Supplementary material for: A manual collection of Syt, Esyt, Rph3a, Rph3al, Doc2, and Dblc2 genes from 46 metazoan genomes - an open access resource for neuroscience and evolutionary biology
Source: BMC Genomics. 2010 Jan 15;11:37. doi: 10.1186/1471-2164-11-37 (PMC2823689; doi:10.1186/1471-2164-11-37)
Supplement: Additional file 44 — Alignment of the vertebrate Esyt1 sequences. Amino acid position is marked every hundred amino acids approximately, at the top of each page of the alignment. Splice variants are included and highlighted with black dots where they differ. The middle portion is only present in the fish esyt1b sequences. A pink dot in this portion, marks an intron loss in T. rubripes and T. nigroviridis. Intron position and phase is indicated with a coloured bar between amino acids. Black bars indicate phase 0 introns. Blue bars indicate phase +2 introns. X residues indicate where a portion of sequence is missing. [file 1471-2164-11-37-S44.PDF]

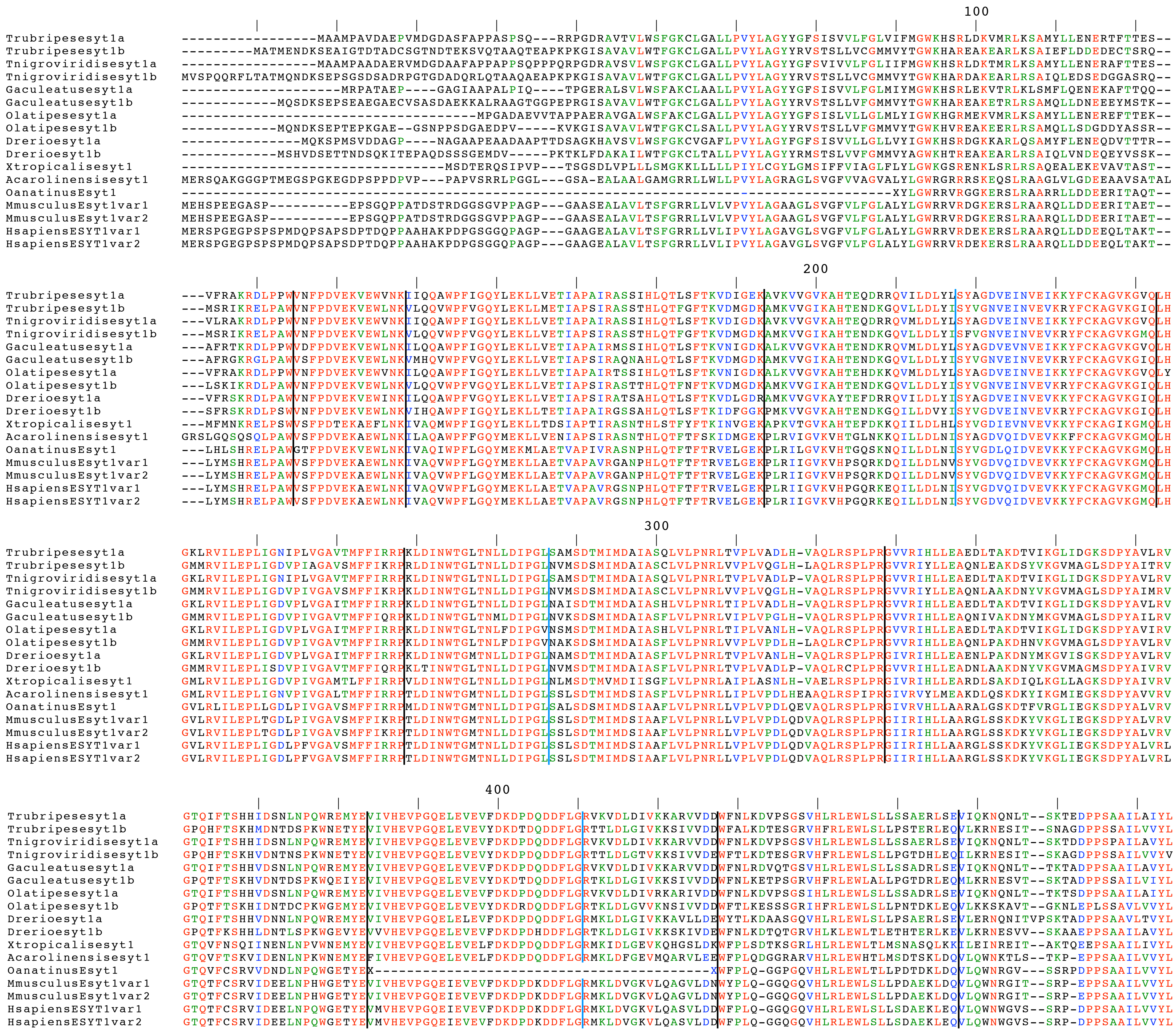
100
200
300
400
Trubripesesyt1a
Trubripesesyt1b
Tnigroviridisesyt1a
Tnigroviridisesyt1b
Gaculeatusesyt1a
Gaculeatusesyt1b
Olatipesesyt1a
Olatipesesyt1b
Drerioesyt1a
Drerioesyt1b
Xtropicalisesyt1
Acarolinensisesyt1
OanatinusEsyt1
MmusculusEsyt1var1
MmusculusEsyt1var2
HsapiensESYT1var1
HsapiensESYT1var2

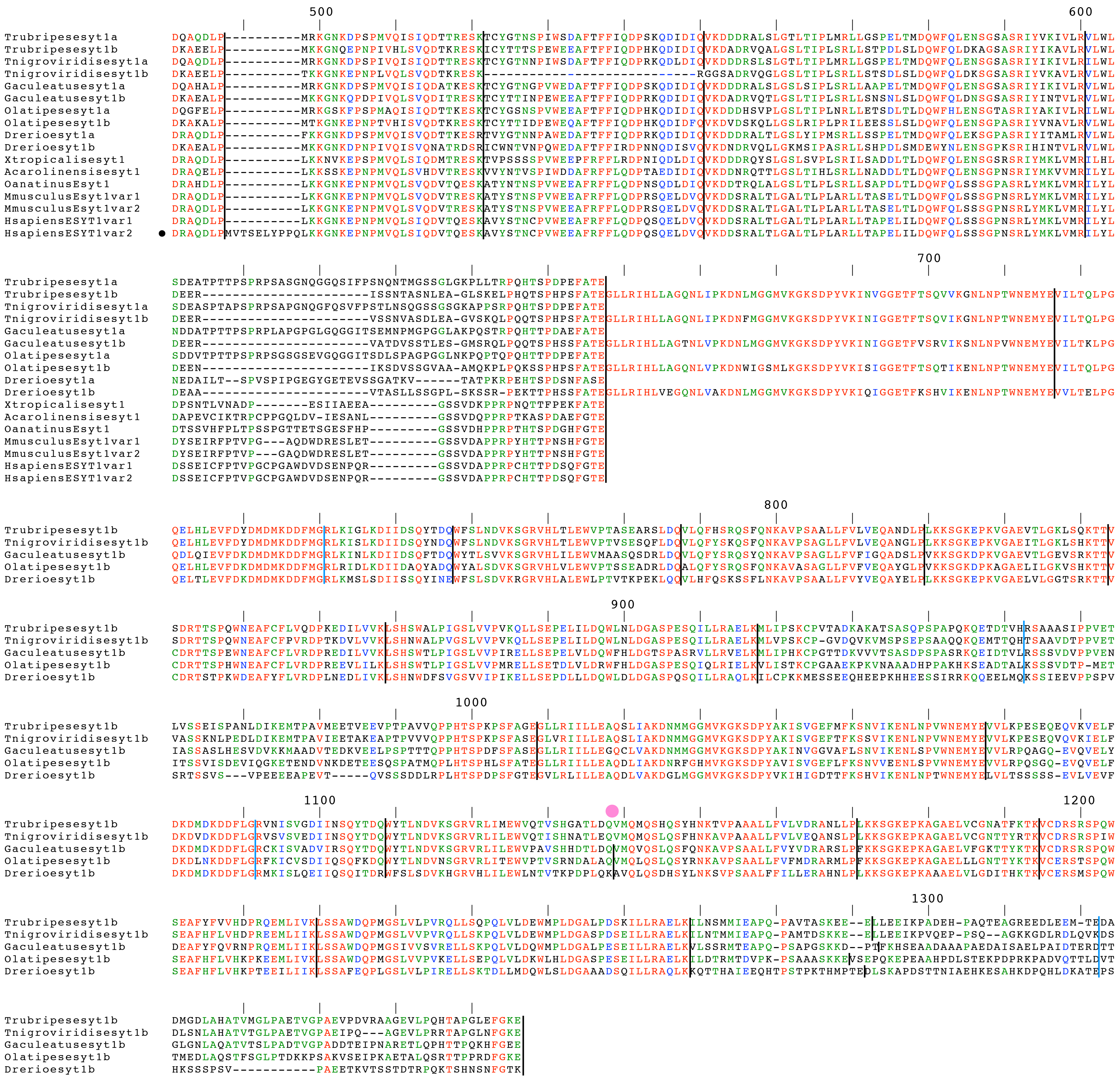

500
600
700
800
900
1000
1100
1200
1300
Trubripesesyt1a
Trubripesesyt1b
Tnigroviridisesyt1a
Tnigroviridisesyt1b
Gaculeatusesyt1a
Gaculeatusesyt1b
Olatipesesyt1a
Olatipesesyt1b
Drerioesyt1a
Drerioesyt1b
Xtropicalisesyt1
Acarolinensisesyt1
OanatinusEsyt1
MmusculusEsyt1var1
MmusculusEsyt1var2
HsapiensESYT1var1
HsapiensESYT1var2

```
                                1400
Trubripesesyt1a           GVLRIHLMEAQNLIAKDNFMGGMVKGKSDPYVKIRVAGITYRSHTIKENLNPTWNELYEVILTQLPGQEIQFELFDKDIDQDDFLGRFKLNLRDIISAQF
Trubripesesyt1b           GVLRIHLLEAQNMVAKDNLMGGMVKGKSDPYVKINIGGTVFKSHVIKENLNPTWNEMYELVLRGNRDHEIKFEAYDKDLDNDDFLGRFSVRLNEVIRSQY
Tnigroviridisesyt1a       GVLRIHLVEAQNLIAKDNFMGGMVKGKSDPYVKIRVAGITYRSHTIKENLNPIWNELYEVILTQLPGQEIQFELFDKDIDQDDFLGRFKLSLRDIISAQF
Tnigroviridisesyt1b       GVLRIHLLEAQNLVAKDNLMGGMVKGKSDPYVKISIGGAVFKSHVIKENLNPTWNEMYELVLNGHTDHEIKIEAYDKDLDNDDFLGRFSVRLNEVIRSQY
Gaculeatusesyt1a          GVLRIHLVEAQNLIAKDNFMGGMVKGKSDPYVKIRVAGITFRSHTIKENLNPTWNELYEVILTQLPGQEIQFELFDKDIDQDDFLGRFKLNLREIISAQF
Gaculeatusesyt1b          GLLRIHLLEAQHLVAKDNLMGGMVKGKSDPYVKINIGGVTFKSHVIKENLNPTWKEMYELVLSEHSVQEIKVEAFDKDLDADDFLGRFSIKLNEVIRSQY
Olatipesesyt1a            GVLRIHLVEAQNLIAKDNFMGGMVKGKSDPYVKIKVAGITFRSHTIKENLNPVWNELYEVILTQLPGQEIQFELFDKDIDQDDFLGRFKLSLRDIINGQF
Olatipesesyt1b            GVLRIHLLEAKNLVAKDIVLG---KGKSDPYVKINIGGFMFKSHVIKENLNPTWNEMYEVVLSGNHDQDIKFEAFDKDLNSDDFLGRFSVRLNEVMSAQY
Drerioesyt1a              GVLRIHLVEAQSLVAKDNLMGGMMKGKSDPYVKIRVGGLAFKSQVIKENLNPVWNELYEVILTQLPGQEVEFDLFDKDIDQDDFLGRVKVSLRDLISAQF
Drerioesyt1b              GLLRLHLLEAQDLVAKDGLMGGMMKGKSDPYVKIHIGDTTFKSHVIKENLNPTWNEMYELILSPDPNLEVKFEVYDKDVDSDDFLGRFKLRLGDIIKSQY
Xtropicalisesyt1          KLLRIFVLEAENLIAKDNLMGGLVKGKSDPYTVISSGGKKVRTRVIDNNLNPCWNQAFEVLVTDIPGQDIVFEVFDKDVDKDDFLGSCQISVKDAVKQKF
Acarolinensisesyt1        SVIRIHLLEAENLIAKDNFMGGMIKGKSDPYVKVRLGGQKFRSRVIKEDLNPRWSEIYEVVVSDIPGQEVEFDLYDKDVDKDDFLGRCKIPLRQVLSSKF
OanatinusEsyt1            SVLRIHVLEAQGLIAKDKFLGGLVRGKSDPYVKLRVGGLSFRTRVVREELSPRWNEVFEVIVTTVPGQELEVEVFDKDLDKDDFLGRCKVSLGPVLSSGF
MmusculusEsyt1var1        NVLRIHVLEAQDLIAKDRFLGGLVKGKSDPYVKLKVAGKSFRTHVVREDLNPRWNEVFEVIVTSIPGQELEIEVFDKDLDKDDFLGRYKVSLTTVLNSGF
MmusculusEsyt1var2        NVLRIHVLEAQDLIAKDRFLGGLVKGKSDPYVKLKVAGKSFRTHVVREDLNPRWNEVFEVIVTSIPGQELEIEVFDKDLDKDDFLGRYKVSLTTVLNSGF
HsapiensESYT1var1         HVLRIHVLEAQDLIAKDRFLGGLVKGKSDPYVKLKLAGRSFRSHVVREDLNPRWNEVFEVIVTSVPGQELEVEVFDKDLDKDDFLGRCKVRLTTVLNSGF
HsapiensESYT1var2         HVLRIHVLEAQDLIAKDRFLGGLVKGKSDPYVKLKLAGRSFRSHVVREDLNPRWNEVFEVIVTSVPGQELEVEVFDKDLDKDDFLGRCKVRLTTVLNSGF

                              1500                                                                                  1600
Trubripesesyt1a           IDTWYTLNDVKSGQVHLVLEWLPRVSELNRLEQILQYQAQQSYQNKVVPSSAMLFVYVERAHGLPLKKNGKEPKVGADVLLKNVSHRTKVCERSTSPRWDEGFHFLVRDPKEETLTVKLSHSWG
Trubripesesyt1b           TDQWYTLNDVKSGKVHLILEWVPAVSHPVRLDEVLQLQSLQSFQNKAVPAAALLFIHLEGAHSLPLKKSGKEPKAGAELVLGETTYKTQLCDRSTSPQWNESFYFLVHDPKLQMLIVKLSSGWD
Tnigroviridisesyt1a       IDTWYTLNDVKSGRVHLVLEWLPRVSDLKRLEPILQYQVQQSYQNKVVPSAAMLFVYVERAHGLPLKKSGKEPKVGADVLLRNVSHRTKVCERSTSPRWDEGFHFLVRDPKEETLTVKLSHSWG
Tnigroviridisesyt1b       TDQWYTLNDVKSGKVHLILEWVPAVSHPARLDQVLQLQALQSFQNKAAPAAALLFVYVDRAHSLPLKKSGKEPKAGAELVLGETRYKTQLCDRSTSPQWNESFYFLVHDPKHQMLIVKLSSGWD
Gaculeatusesyt1a          IDTWYTLNDVKSGRVHLELEWLPRVADLPRLEQILQYQSQQSYHNKLVPSSALLFVYVERAHGLPLKKNGKEPKVGAEITLKGVSHRTKICERSTSPRWDEAFHFLVRDPRDETLSVKLSHSWG
Gaculeatusesyt1b          TDQWYTLNDVKSGRVHLILEWVPTVSHSVRLDQVLQLQSLQSYQNKAVPAAALLFVHLERAHSLPLKKSGKDPKAGAELVLGETTYKTKLCERSTNPQWNECFYFLVHNPKDQMLVVKLSSGWD
Olatipesesyt1a            IDSWYTLNDVKSGRVHLVLEWLPRVSDLIRLEQVLQYQSQQLYQNKAVPSAAVLFVYVERAHGLPLKKNGKEPKAGVEVALKGVSFKTKICERSTSPRWDEAFHFLVRDPTDETLTVKLSHSWG
Olatipesesyt1b            TDQWFTLKDVKSGQVHVILEWVPTVSSSIRLDQVMQLESLQSYQNKAVPSAALLFVYIQAAQSLPLKKSGKEPRAGAELVLGEASSKTKLCDRSANPQWNESFHFVVRDPKRQTLIVKLSSGWD
Drerioesyt1a              TDQWYTLNDVKTGRIHLVLEWVPKISDPIRLEQILQYNYRQSYLNKIVPSAALLFVYIERAHGLPLKKSGKEPKAGAEVSLKNVSYRTKVVNRSTSPQWDEALHFLIHNPTEDTLIVKVSHSWG
Drerioesyt1b              NDEWFTLNDIKHGRVHLVVEWLPTVTQRDKLEQVMQMQSSQSYQNKSVASAALLFILLDRAHQLPLKKSGKEPKAAAELTLGGTSYKSKVCERSSSPHWNETFDFLVHDPKKDVLVIKLSSAWD
Xtropicalisesyt1          IDEWLPLEKVKSGKLHVKLECLSLLADSSQIDQVLMMNSLNQPAHSDNFSAALLYVFIERANGLQMRKGDKNPSPSAELKIRKDIYKTKVAQNTNAPAWEESFVFLLKTPHSEELELMIRDEGK
Acarolinensisesyt1        VDEWLPLEDVKSGRLHVKLECLPPTYSAAELEQVLIVNSLIQTPKSEELSSALLSVFLDRAADLPMRKGSKPPSPFVSLSVRGISYKTKVSSQTADPVWDEAFSFLIKKPHAESLELQVKDDGH
OanatinusEsyt1            MDEWLYLEDVSSGRLHLRLERLSPRPSTAELEQVLQVNSLIQTNKSTELAAALLSVYLEHAADLPX-----------------------------------------------------------
MmusculusEsyt1var1        LDEWLTLEDVPSGRLHLRLERLTPRPTAAELEEVLQVNSLIQTQKSSELAAALLSVFLERAEDLPLRKGTKPPSPYATITVGETSHKTKTVSQSSAPVWEESASFLIRKPHAESLELQVRGEGT
MmusculusEsyt1var2      ● LDEWLTLEDVPSGRLHLRLERLTPRPTAAELEEVLQVNSLIQTQKSSELAAALLSVFLERAEDLPLRKGTKPPSPYATITVGETSHKTKVGGD--APLLRTGGRRLQDPVPASHPNPSVSNRLS
HsapiensESYT1var1         LDEWLTLEDVPSGRLHLRLERLTPRPTAAELEEVLQVNSLIQTQKSAELAAALLSIYMERAEDLPLRKGTKHLSPYATLTVGDSSHKTKTISQTSAPVWDESASFLIRKPHTESLELQVRGEGT
HsapiensESYT1var2         LDEWLTLEDVPSGRLHLRLERLTPRPTAAELEEVLQVNSLIQTQKSAELAAALLSIYMERAEDLPLRKGTKHLSPYATLTVGDSSHKTKTISQTSAPVWDESASFLIRKPHTESLELQVRGEGT

                                                                                                   1700
Trubripesesyt1a           QALGSLTLPLRDVLLEPGMVLDRWINLDGALPESQILLRVMLKILDTQ----------LVACRR---NADGEISGDDVIPRWVPSHLDIRQRTAFSMGSDNMNPTS--QVKLTIGYSTEEARLF
Trubripesesyt1b           QPMGSLVLPVKNLLAAPQLVMDQWFHLDGALPESQVLLRAELKILDSK----------MLDL--------------------------ISAGTLPCAAPIWGSTNG--QVKLTLSYSLQERQLM
Tnigroviridisesyt1a       QALGSLTLPLRDVLLDPGMVLDRWFNVDGALPESQILLRVMLKILDTQ----------LVTCRR---DADGEVGGDDVIPRWVPSHLDIRQRNAFSMGSDNANTAG--QVKLTIGYSAEESRLF
Tnigroviridisesyt1b       QPMGSLVISVKSLLAEPQLLTDQWFRLDGALPDSQVLLRAELKILDSK----------MVDM--------------------------ISSGTLPCTAPIWGSGNG--QVEVTLSYSPQERQLM
Gaculeatusesyt1a          QALGSFTIPLREVLFEPGLVLDRWINLDGALPESQILLRATLKVLDTQ----------LALCRRAPGDVGNDGGGEEGLPRWVPSHLDLRHR--FSM-AESTNSAG--QVKLTVGYSVEESRLF
Gaculeatusesyt1b          QPMGSLVIPVNELLAEPHLVLDQWFHLDGATPDSQILLRAELKVLHSK----------MVDM--------------------------IGPGTLPCVA----SGCG--QVKLSLSYALQERHLT
Olatipesesyt1a            QALGSLTLPLKEVLSESGLVLDRWLSLDGALPESQILLRVTLKVLDTQ----------LAVCRSGPVGAANDADEETVFPKWDPSHLDLRRRSRFSVVSDNANTAG--QVKLTIGYSTEENRLF
Olatipesesyt1b            EPMGSLVVPVRELLSEPELVLDRWFHLDGASAYSQILLRAELKILNTK----------MLHL--------------------------IGTEALPCAA----ASAG--QLKMSLTYAPQQKKLV
Drerioesyt1a              QALGSLVLPVRELLEEKDLTIDRWFSLNGAMPESQILLRAELKLLDSK----------LAQCS----DEEDASHVIPSAEPTVAANSELRHRNVPVQGGEDASSTGKAQIKLSISYSSEEHRLI
Drerioesyt1b              QPMGSLVLPIRELLLKPDLLLDQWLSLDGASAQSQILLRAQLKILDSK----------MAAL--------------------------VAMGSGPVLSNKQTATTG--QIQLSMSF---QKKLT
Xtropicalisesyt1          GSLGSLTVPLVDLLKKEDLTMDGWFPLNTSGTYSEVLIRLQMRVLVSP------------SPLKESEVDPKQPTIEDISLNKQSTEGELRQRQQLTNSDMESSASPFGQLCLTLYYPAKDGRLV
Acarolinensisesyt1        -VLGSLSLPLTQLLVAEGLVLDQWFQLNNASPTTQILMRVQLGLLVSQHSGVEAFHAVMGEDNEESSLKGSETELYIQEDGDSRSMQELRQRMPHANSNLELSDSPLGQLHLTVWYNIDARKLI
OanatinusEsyt1            -------------------------------------------------------------------------------------------------------XPQRHRLGKLGQVKLTVWYYTEEQKLV
MmusculusEsyt1var1        GTLGSVSLPLSELLQEDQLCLDHWFALS-GQG--QVLMRAQLGILVSQHSGVEA-HSHSYSHSHSSSSLNDEPEALGGPTHPASPVLEVRHRLTHGDSPSEAPVGPLGQVKLTVWYHSDEQKLI
MmusculusEsyt1var2      ● PNLRPLSGRRVPLFSSGSHTLRAWSCRFGVKGPAPWALYPFLSLSSSRRTS-SAWTTGLHSVVRARC---------------------------------------------------------
HsapiensESYT1var1         GVLGSLSLPLSELLVADQLCLDRWFTLSSGQG--QVLLRAQLGILVSQHSGVEA-HSHSYSHS--SSSLSEEPELSGGPPHITSSAPELRQRLTHVDSPLEAPAGPLGQVKLTLWYYSEERKLV
HsapiensESYT1var2         GVLGSLSLPLSELLVADQLCLDRWFTLSSGQG--QVLLRAQLGILVSQHSGVEA-HSHSYSHS--SSSLSEEPELSGGPPHITSSAPELRQRLTHVDSPLEAPAGPLGQVKLTLWYYSEERKLV

                                                                                     1800
Trubripesesyt1a           ITVHACRALAACSKDGADPYVSFILLPDKATTKRRTATKKRDLNPEFNERFDFDFSLEESYQRRLDLSVKN-SVSFMSRERELIGKLQLDLEEIDLKACVTQWYDLVAELN----
Trubripesesyt1b           VVVHACRGLLPQSKDAVDSYVSLMLLPDKNKATKKKTAVKKRDQNPEYNERFEYDLPLDELRFRRLSVTVKNNSASF--RSRDVIGQVQIELAQINLVAGVTDWFTLRDDAE----
Tnigroviridisesyt1a       ITVHACRVLAACSKDGADPYVSFILLPDKKATTKRRTATKKRDLNPEFNERFDFDFSLEESLQRRLDLSVKN-SVSFMSRERELIGKLLLDLEEIDLKACLTQWYDLVAELN----
Tnigroviridisesyt1b       VVVHACRGLLPQSKDGVDSYVSLLLLPDKNKATKKKTAVKKRDHNPEYNERFRYDLPPDELRFRRLSVTVKNNSASF--RSRDVIGQVQIELAQVDLTAGVTDWFTLRDDDE----
Gaculeatusesyt1a          ITIHSCRALAACSKDGADPYVSFILLPDKKATTKRRTATKKRDLNPEFNERFDFDFSLEESTQRRLDLSVKN-SVSFMSRERELIGKLQLDLDQIDLKAGITQWYDLVAETN----
Gaculeatusesyt1b          VIVHACRGLLSQSKDGVDSYVSLMLLPDKSKATKRKTAVKKRDLNPEYNERFEYELSVDEMRFKRLILSVKNNSASF--RSRDIIGQVQIELAHVDLVSGVTAWFTLKVEAE----
Olatipesesyt1a            ITVHSCRGLAPRSKDGADPYVSFILLPDKKATTKRRTTTKRKDLNPEFNERFEFDFSLEESTQRKLYLSVKN-SVSFMSREKELIGKLQLDLDQIDLKAGVAQWFDLVAETN----
Olatipesesyt1b            VLVHACRGLLAQSKDGVDSYVSLMLLPDKTKATKRKTAVKKKDLNPEYNERFEFDLPEQEVPFKCLSASVKNNS--F--RGKDVIGQVQVELAQMDLKSGITQWFALSDEAE----
Drerioesyt1a              IKVHACRNLPSSSKEPPDSYISFILLPDKNRNTKKKTSVKKKSLKPEFNEIFEFDMSLEEAKQKHLEVSVKN-SVSFMSREKELLGKLQIDFSPLDLKTGVSQWYDLSHETN----
Drerioesyt1b              LLVHNCRGLVTSSKDSLDTYVSIILLPDKSKATKRKTSVKKKTLNPEFNEKFEFDMSMEEVQRRELSVCVKNASSSFMNRDKDVIGQVQIDLGHIDLISGVTQWFDLKEEQN----
Xtropicalisesyt1          AIVSKGRNLCTSAKEPPDPYLSFVLLPDKSRSTKKKTSVKKRTHNPEFNEKFEWEMSLEEARKRKLEISVKN-SVSFMSREKELLGKVLIDLSDVDLATGQNGWYDVGPENTSV--
Acarolinensisesyt1        AIIHACRNLKSSAKDIPDPYVSLILLPDKSRVTKRKTAVRKKTLNPEFNEKFEWDLTLEEAQRRKLEAYIKN-SISFMSRG-EPIGKVHIDLSQKDLTQGAAQX------------
OanatinusEsyt1            SLVHSCRALRQSGRDTPDPYVSLLLLPDKNRASKRKTSPKKKTLSPEFNERFEWDMSLDEAMRRKLEVSVKS-NVSFMTREREL X-----------------------------
MmusculusEsyt1var1        SIIHSCRALRQNGRDLPDPYVSVLLLPDKNRSTKRKTPQKKRTLNPEFNERFEWDLPLDGTLRRKLDVSVKS-NSSFMSRERELLGKVQLDLAEIDLSQGAAQWYDLMDDRDKGGS
MmusculusEsyt1var2        -----------------------------------------------------------------------------------------------------------------------
HsapiensESYT1var1         SIVHGCRSLRQNGRDPPDPYVSLLLLPDKNRGTKRRTSQKKRTLSPEFNERFEWELPLDEAQRRKLDVSVKS-NSSFMSRERELLGKVQLDLAETDLSQGVARWYDLMDNKDKGSS
HsapiensESYT1var2         SIVHGCRSLRQNGRDPPDPYVSLLLLPDKNRGTKRRTSQKKRTLSPEFNERFEWELPLDEAQRRKLDVSVKS-NSSFMSRERELLGKVQLDLAETDLSQGVARWYDLMDNKDKGSS
```
