# Supplementary material for: A manual collection of Syt, Esyt, Rph3a, Rph3al, Doc2, and Dblc2 genes from 46 metazoan genomes - an open access resource for neuroscience and evolutionary biology
Source: BMC Genomics. 2010 Jan 15;11:37. doi: 10.1186/1471-2164-11-37 (PMC2823689; doi:10.1186/1471-2164-11-37)
Supplement: Additional file 45 — Alignment of the vertebrate Esyt2 sequences. Amino acid position is marked every hundred amino acids approximately, at the top of each page of the alignment. Splice variants are included and highlighted with black dots where they differ. Intron position and phase is indicated with a coloured bar between amino acids. Black bars indicate phase 0 introns. Blue bars indicate phase +2 introns. X residues indicate where a portion of sequence is missing. [file 1471-2164-11-37-S45.PDF]

```
                                                                                                 100
Trubripesesyt2a              --------------------------------------MEGPGVGTNGPLPAPRNSVSSTTGPPLPPDQLHEEPQSSLTDLIHMWIKFGKTFAIIFPIYVLGYLEFSFSWVLVGLAML
Trubripesesyt2b              ------------------------MSRDSMTGVRGAHSRNAST---KEKGHAAPSSAKPNFPHPSAGGTSGDTPLSCTSELTQTWIQLAKTFVLIFPIYALGYFEFSFSWLLIGLAIF
Tnigroviridisesyt2a          --------------------------------------MEGSSVGTNGALPAPRNSGSSTPGPPLPPGQVEAEQQSSLTELIQMWIKFGKTFAIIFPIYVLGYFEFSFSWVLIGLATL
Tnigroviridisesyt2b          ------------------------MSDDSMAGVRGAHPRHWSP---KENGHAA----RANFPHPSAGGTSGDTPLCLTSELTQTWIQLAKTFVLIFPIYALGYFEFSFSWLLIGLAIF
Gaculeatusesyt2a             -----------------------------------MEGSVGPDAKANGPIAAPSSPVRSTVGPPLAPQV---EPQSSMTDVTQMWIKFGKTFALILPIYILGYFEFSFSWVLVGLAAL
Gaculeatusesyt2bvar1         -----------------------------MTDPRGARL-------PKENGHVAPS---PNFPRASPGDTADDAPVSSAAALTRTWLDVAKTFVLIFPIYALGYFEFSFSWLLIGLVIF
Gaculeatusesyt2bvar2         -----------------------------MTDPRGARL-------PKENGHVAPS---PNFPRASPGDTADDAPVSSAAALTRTWLDVAKTFVLIFPIYALGYFEFSFSWLLIGLVIF
Olatipesesyt2a               -----------------------------------MEGSAQPGARSNGPSAAPSSQVDSPLSPPLSPQEPTEEDQSSLANVTQMGIEFAKSFAVIFPIYVLGYFEFSFSWILIGLAMV
Olatipesesyt2b               ------------------------MSRDSMTAVRGAQSQSASPAAPKENGHTAASAAQPNFPPPSPASAPDDAPLSSTGELTQTWVHFAKTFVFIFPVYALGYFEFSFSWLLIGLMIF
Drerioesyt2a                 ----------------------------MSASVNGVEPKPAPAVSQNGPGSPPQTPKDVTPPELELPDE---EPQSSVTEATQMGIKFAKTFLLIFPIYLLGYLEFSFSWVLIGLGLV
Drerioesyt2b                 ----------------------MGFSEDVMTAVRGNLSQTAVASGQKDNGQAEPT--NGAIPCSSSPVELSEEPLSCSSELTRTWLQFAKTFVVIFPIYVLGYFEFSFSWLLIALTIF
Xtropicalisesyt2             ----------------------------------------MATESSAQKGPPPSPAENGQPGGPPAAAVPAEEQGMISVDIAGLFYQFSKTFVLIFPVYVLGYFGLSFSWLLIALVLL
Acarolinensisesyt2           -------------------------------------MSGAGAQARAGPGPGPSGDSEPPPPPPPPPSSS---SQPMISVDLAGLVSQFARSFALVFPVYVLGYLGLSFSWILLALLGL
GgallusESYT2                 -------------------------------------MSGAAEKQSPG-AGTPAAAEKAAAGGDAGPEPP--PPSMLSVDVTGLVSQFARSFVLIFPVYVLGYLGLSFSWVLIALCGL
TguttataESYT2                ----------------------------------------------------------XAAAADPAPESP--SP-LLSVDVTGLVSQFARSFVLIFPVYVLGYLGLSFSWVLITLCGL
OanatinusEsyt2               ----------------------------------------------------------------------------------------------------------------------
MdomesticaEsyt2              -------------------------------------MSGSGDRTPGGDAASKAAGDLSTTAAKGPGAEP--PPAMLSVDLPGLCAQFARSFVLLFPVYVLGYLGLSFLWILLGLLLL
MmusculusEsyt2var1           ------------------------------------------------MSSAGGEGPEAGPGRAGGRSEPEAPGSALSVDLPGLLGQLARSFALLLPVYALGYLGLSFSWVLLALGLL
MmusculusEsyt2var2           ------------------------------------------------MSSAGGEGPEAGPGRAGGRSEPEAPGSALSVDLPGLLGQLARSFALLLPVYALGYLGLSFSWVLLALGLL
MmusculusEsyt2var3           ----------------------------------------------------------------------------------------------------------------------
MmusculusEsyt2var4           ----------------------------------------------------------------------------------------------------------------------
HsapiensESYT2var1            ----------------------------------------------------------------------------------------------------------------------
HsapiensESYT2var2            ----------------------------------------------------------------------------------------------------------------------
HsapiensESYT2var3          ● MTPPSRAEAGVRRSRVPSEGRWRGAEPPGISASTQPASAGRAARHCGAMSGARGEGPEAGAGGAGGRAAPENPGGVLSVELPGLLAQLARSFALLLPVYALGYLGLSFSWVLLALALL
HsapiensESYT2var4            ----------------------------------------------------------------------------------------------------------------------

                                                                                           200
Trubripesesyt2a              FYWRKNHGNKDYRINRALAFLEHEEKAVKQSVPTTDLPPWVHYPDVERTEWLNKTVKQMWPFICQFVDKLFRETIEPAVKGANPHLSSFCFTKIDMGQKPLRVNGVKVYTENVDKRQI
Trubripesesyt2b              FWWRRHTGGKRSRVSRAFAFFEQAERSVTQSLSTSDLPPWVHFPDVERVEWLNKTVGQMWPYICQFVEKLLHEALEPAVKASDPHLSTFCFSKIDIGDKPLRVNGVKVYTENVDKRQI
Tnigroviridisesyt2a          FYWRKNHGNKDYRINRALKYLEHEDKAVKQSVPTTDLPPWVHYPDVERVEWLNKTVKQMWPFICQFVDKLFRETIEPAVKGANPHLSSFCFSKIDMGQKPLRVNGVKVYTENVDKRQV
Tnigroviridisesyt2b          FWWRRRTGGKHSRVSRAFAFFEQAERSVTQSLTTSDLPPWVHFPDVERVEWLNKTVGQMWPYVCQFVEKLLETVEPAVKASDPHLSTFCFSKIDMGDKPLRVNGVKVYTENVDKRQI
Gaculeatusesyt2a             CYWKRNHGGKDYRINRAMAFLEHEDKTATQSLATSELPPWVHYPDVERVEWLNKTVKQMWPFICQFVDKLFRETIEPAVKGANPHLASFCFTKIDMGDKPLRVDGVKVYTENVDKRQI
Gaculeatusesyt2bvar1         FWWRRNAAGKHGRLGRAFAFLEQEERGVEQSLATSDLPPWVHFPDIERVEWLNKTVKQMWPYICQFAEKLFHDTIEPAVKQSNAHLSTFCFSKIDMGDKPLRVNGVKVYTENVDKRQI
Gaculeatusesyt2bvar2         FWWRRNAAGKHGRLGRAFAFLEQEERGVEQSLATSDLPPWVHFPDIERVEWLNKTVKQMWPYICQFAEKLFHDTIEPAVKQSNAHLSTFCFSKIDMGDKPLRVNGVKVYTENVDKRQI
Olatipesesyt2a               FYWKKNYGKRDYRINRALAYLEHKDKVVKLSLPTTEMPPWVHYPDVERVEWLNKTVKQMWPFICQFVDKLFRETIEPAVKGANPHLSSFCFTKIDMGDKPLRVNGVKVYTENIDKRQV
Olatipesesyt2b               FLWRRNTGGKHSRLSRALAFFDQEELTAKPGLTTSDLPPWVHFPDVERVEWLNKTVKQMWPYICQFVEKLFRETIEPAVKETHAHLSTFCFTKIDMGDKPLRINGVKVYTENVDKRQI
Drerioesyt2a                 FWLKRNQGSRFARVNQAMAFLEQEERAVRQTIRSSELPPWVHFPDVERVEWLNKTVQQMWPYICQFVEKIFKETIEPAVQGANTHLSTFTFSKIDMGDKPLRVDGVKVYTENVDKRQI
Drerioesyt2b                 FFWKRNTNSKNTRLSRAMSIFDPDD-AVKQELDATELPSWVHYPDVERVEWLNKTVNQMWPYVCQFVDKLFKETIEPAIKESNAHLSTFSFTKIDLGDKPLRINGVKVYSENVDKRQI
Xtropicalisesyt2             VWWRKNKGNKNSRLYRALAFLESEEKSVKHHIASIDLPAWVHFPDIERAEWLNKTVKHMWPYICQFIEKLFRETIEPAVRGANAHLSTFNFTKIDMGSQPLRVNGVKVYTENVDKRQI
Acarolinensisesyt2           FWVQRHRGGKASRLGRALAFLQDEERAVKLTVATGDLPAWVHFPDTERAEWLNKTVKHMWPFICQFIEKLFRETIEPAVRGANNHLSTFSFTKIDIGHQPLRINGVKVYTENVDKRQI
GgallusESYT2                 FWIRRHRGGKTSRLGRALAFLEDEEEAVRLSVSSADLPAWVHFPDTERAEWLNKTVKQMWPFICQFIEKLFRETIEPAVRGANNHLSTFSFTKIDIGHQPLRINGVKVYTENVDKRQI
TguttataESYT2                FWIRRHRGGKSSRLSRALAFLEDEEEAVRLSVSSADLPAWVHFPDTERAEWLNKTVKQMWPFICQFIEKLFRETIEPAVRGANNHLSTFSFTKIDIGHQPLRINGVKVYTENVDKRQI
OanatinusEsyt2               ---------------------------------------XVHFPDTERAEWLNKTVKHMWPFICQFIEKLFRETIEPAVRGANTHLSTFSFTKIDMGHQPLRINGVKVYTENVDKRQI
MdomesticaEsyt2              FWCRRNKGHKTSRFYRALAFLENEEQAVRLSICTSDLPAWVHFPDTERAEWLNKTVKHMWPFICQFIEKLFRETIEPAVRGANAHLSTFSFTKIDMGHQPLRINGVKVYTENVDKRQI
MmusculusEsyt2var1           AWCRRSRGLKASRLCRALALLEDEEQAVRLGVRACDLPAWVHFPDTERAEWLNKTVKHMWPFICQFIEKLFRETIEPAVRGANAHLSTFSFTKVDVGQQPLRVNGVKVYTENVDKRQI
MmusculusEsyt2var2           AWCRRSRGLKASRLCRALALLEDEEQAVRLGVRACDLPAWVHFPDTERAEWLNKTVKHMWPFICQFIEKLFRETIEPAVRGANAHLSTFSFTKVDVGQQPLRVNGVKVYTENVDKRQI
MmusculusEsyt2var3           ----------------------------------------------------------------------------------------------------------------------
MmusculusEsyt2var4         ● ------------------------------------------------------------------------------------------------MWTPLRVNGVKVYTENVDKRQI
HsapiensESYT2var1          ● ----------------------------------------------------------MWPFICQFIEKLFRETIEPAVRGANTHLSTFSFTKVDVGQQPLRINGVKVYTENVDKRQI
HsapiensESYT2var2            ----------------------------------------------------------MWPFICQFIEKLFRETIEPAVRGANTHLSTFSFTKVDVGQQPLRINGVKVYTENVDKRQI
HsapiensESYT2var3            AWCRRSRGLKALRLCRALALLEDEERVVRLGVRACDLPAWVHFPDTERAEWLNKTVKHMWPFICQFIEKLFRETIEPAVRGANTHLSTFSFTKVDVGQQPLRINGVKVYTENVDKRQI
HsapiensESYT2var4            ----------------------------------------------------------------------------------------------------------------------
```

```
                                                                                 300
                                   |         |         |         |         |         |         |         |         |         |         |
Trubripesesyt2a           IMDLQISFVGNTEIDVDIKKYYCRAGIKSIQLHGTLRVVMEPLLGDMPLVGALSVFFLKKPLLDINWTGLTNVLDIPGVNGLCDNIIQDIICTYLVLPNRISIPLVGESQLAQLRFPV
Trubripesesyt2b           IMDLHISFVGNTEIEIDIKRYYCKAGIKSIQMHGVLRVVMEPLLGDLPLVGALSVFFLKKPLLDVNWTGLTNILDIPGLSGFSDSLIQDLINSYLVLPNRITVPLVGDVQLAQLRFPM
Tnigroviridisesyt2a       IMDLQISFVGNTEIDVDIKKYYCRAGIKSIQLHGTLRVVMEPLLGDMPLVGALSVFFLKKPLLDINWTGLTNILDIPGVNGLCDNIIQDIICTYLVLPNRISIPLVGESQLAQLRFPI
Tnigroviridisesyt2b       IMDLHISFVGNTEIEIDIKRYYCKAGIKSIQMHGVLRVVLEPLLGDLPLVGALSAFFLKKPLLDVNWTGLTNILDIPGLSGFSDSLIQDLIYSYLVLPNRVTVPLVGEVELAQLRFPM
Gaculeatusesyt2a          IMDLQISFVGNTEIDVHIKKYYCRAGIQSIQLHGMVRVVMEPLLGDMPLIGALSVFFLKKPLLDINWTGLTNMLDIPGVNGLFDNVIQDIIASQMVLPNRISIPLVQESQLAQLRFPI
Gaculeatusesyt2bvar1      IMDLQISFVGNTEIDIDIKRYYCKAGIKSIQIHGVLRVVMEPLLGDMPLIGALSVFFLKKPLLDINWTGLTNILDIPGLNGLSDNLIQDIIYNYLVLPNRITIPLVGDVELALLRFPM
Gaculeatusesyt2bvar2      IMDLQISFVGNTEIDIDIKRYYCKAGIKSIQIHGVLRVVMEPLLGDMPLIGALSVFFLKKPLLDINWTGLTNILDIPGLNGLSDNLIQDIIYNYLVLPNRITIPLVGDVELALLRFPM
Olatipesesyt2a            IMDLQISFVGNTEIDVDIKKYYCRAGIKSIQLHGTLRVVMEPLLGDMPLIGALTVFFLQKPLLDINWTGLTNILDIPGLNGLCDGIIQDIIQGYLVMPNQIRIPLVGEAELSRIRFPT
Olatipesesyt2b            IMDLQISFVGNTEIDVDVKRYYCKAGIKSIQIHGVLRVVMEPLLGEMPLIGALSLFFLKKPLLDINWTGLTNILDIPGLHGFSDSLIQDIICSYLVLPNRVTVPLVSEMELAKLRFPV
Drerioesyt2a              IMDLQINFVGNTEIDVDIKKYYCRAGIKSIQLNGVLRVIMEPLLGDMPLVGALSVFFLKKPFLDINWTGLTNMLDIPGVNSLCDNVIQDIINGCLVNPNKITIPLADDALISKLRFPM
Drerioesyt2b              IMDLQISYVGNTEIDVDVKRYYCRAGIKSIQLHGVLRVILEPLLGNMPLVGAFSLFFLKKPLLDINWTGLTNILDIPGLNGFSDQMIQDIISAYMVLPNRITVPLIGEVELAQLRFPM
Xtropicalisesyt2          ILDLQISFVGETEIDLEVKRYFCRAGVKSIQLHGTMRVVLEPLIGDVPIVGALSIFFLRKPLLEINWTGLTNMLDMPGLNGLSDTIILDIISNYLVLPNRITVPLVSDVQIAQLRFPI
Acarolinensisesyt2        ILDLQISFVGNCEIDLEIKRYFCRAGVKSIQIHGTMRVILEPLLGDMPLIGALSLFFLRKPLLEINWTGLTNLLDIPGLNGLSDTIILDIISNYLVLPNRITX---------------
GgallusESYT2              ILDLQISFAGNCEIDLEIKRYFCRAGVKSIQIHGTMRVILEPLIGDMPLIGALSLFFLRKPLLEINWTGLTNLLDVPGLNGLSDTIILDIISNYLVLPNRITVPLVSEVQIAQLRFPI
TguttataESYT2             ILDLQISFVGNCEIDLEIKRYFCRAGVKSIQIHGTMRVILEPLIGDMPLIGALSLFFLRKPLLEINWTGLTNLLDVPGLNGLSDTIILDIISNYLVLPNRITVPLVSEVQIAQLRFPI
OanatinusEsyt2            ILDLQISYVGNCEVDLEIKRYFCRAGVKSMQIHGTMRVILEPLIGDMPLVGALSIFFLRKPLLEINWTGLTNLLDVPGLNGLSDTIILDIISNYLVLPNRITVPLVSEVQIAQLRFPI
MdomesticaEsyt2           ILDLQISFVGNCEIDLEIKRYFCRAGVKSIQIHGTMRVILEPLIGDMPLVGALSIFFLRKPLLEINWTGLTNLLDIPGLNGLSDTIILDIISNYLVLPNRITVPLVSEVQIAQLRFPI
MmusculusEsyt2var1        ILDLQISFVGNCEIDLEIKRYFCRAGVKSIQIHGTMRVILEPLIGDMPLVGALSIFFLRKPLLEINWTGLTNLLDIPGLNGLSDTIILDIISNYLVLPNRITVPLVSEVQIAQLRFPI
MmusculusEsyt2var2        ILDLQISFVGNCEIDLEIKRYFCRAGVKSIQIHGTMRVILEPLIGDMPLVGALSIFFLRKPLLEINWTGLTNLLDIPGLNGLSDTIILDIISNYLVLPNRITVPLVSEVQIAQLRFPI
MmusculusEsyt2var3        ----------------------------------------------------------------------------------------------------------------------
MmusculusEsyt2var4        ILDLQISFVGNCEIDLEIKRYFCRAGVKSIQIHGTMRVILEPLIGDMPLVGALSIFFLRKPLLEINWTGLTNLLDIPGLNGLSDTIILDIISNYLVLPNRITVPLVSEVQIAQLRFPI
HsapiensESYT2var1         ILDLQISFVGNCEIDLEIKRYFCRAGVKSIQIHGTMRVILEPLIGDMPLVGALSIFFLRKPLLEINWTGLTNLLDVPGLNGLSDTIILDIISNYLVLPNRITVPLVSEVQIAQLRFPV
HsapiensESYT2var2         ILDLQISFVGNCEIDLEIKRYFCRAGVKSIQIHGTMRVILEPLIGDMPLVGALSIFFLRKPLLEINWTGLTNLLDVPGLNGLSDTIILDIISNYLVLPNRITVPLVSEVQIAQLRFPV
HsapiensESYT2var3         ILDLQISFVGNCEIDLEIKRYFCRAGVKSIQIHGTMRVILEPLIGDMPLVGALSIFFLRKPLLEINWTGLTNLLDVPGLNGLSDTIILDIISNYLVLPNRITVPLVSEVQIAQLRFPV
HsapiensESYT2var4         ----------------------------------------------------------------------------------------------------------------------

                                                                      400
                                   |         |         |         |         |         |         |         |         |         |         |
Trubripesesyt2a           PKGVLRIHFLEAQDLLGKDKFLGGLIKGKSDPYGVLRFGTELFQSKVIHETVNPKWNEVYEALIYENTGKNLEIELFDEDTDKDDFLGCLMIDLAQIQQQKIDEWFSLEDVPQGKLH
Trubripesesyt2b           PKGVLRIHFLEALDLEGKDQFLGGLIKGKSDPYGVLQIGNQLFQSKTVKESLHPKWNEVYEALVYEHSGQHLEIELFDEDPDKDDFLGSLMIDLTELHKEQKVDEWFDLEETSTGKLH
Tnigroviridisesyt2a       PKCILRIHFVEAQDLVGKDRFLGGLIKSKSDPYGVLRVGTELFKSKVIHETVNPKWNEVYEALIYDNSGKNLVIELYDEDTDKDDFLGCLTIDLAEIEKQQKVDEX-----------
Tnigroviridisesyt2b       PKGVLRIHFLEALDLEGKDKFLGGLIKGKSDPYGVLQIGNQLFQSKTVKESLHPKWNEVYEALVYEHSGQHLEIELFDEDPDKDDFLGSLMIDMTELHKEQKVDEWFDLEETSTGKLH
Gaculeatusesyt2a          PKGILRIHFMEAQDLLGKDKFLGGLIKGKSDPYGFVRIGTQVFQSKVIHENLNPKWNEVYEAFIYDYTTHNLEIELFDEDTDKDDFLGSLTIDLAEVQKERKVDEWFVIENVDTGKLH
Gaculeatusesyt2bvar1      PKGVLRIHFLEAQDLEGKDTFLGGLIKGKSDPYGVLQIGNQVFHSKVVKESLNPKWNEVYEALVYEHSGQHLEIELFDEDPDKDDFLGSLMIDMSELYKEQKVDEWFDLEETPTGKLH
Gaculeatusesyt2bvar2      PKGVLRIHFLEAQDLEGKDTFLGGLIKGKSDPYGVLQIGNQVFHSKVVKESLNPKWNEVYEALVYEHSGQHLEIELFDEDPDKDDFLGSLMIDMSELYKEQKVDEWFDLEETPTGKLH
Olatipesesyt2a            PKAVLRIHFIEAQELMSKDRLLGGLIKGKSDPYGVIQVGTVLFQSKIINESLNPKWNEVYEALIYDNMPNEVKFELFDKDNNQDDFLGGLSLDLVELQKVLMVDQWFPLDDARTGKLH
Olatipesesyt2b            PKGVLRIHFLEAQDLEGKDKFLGGLIMGKSDPYGVLQIGNQVFQSKTVKQTLNPKWNEVYEALVYEHSGEHLEIELFDEDPDKDDFLGSLMIDLAELHKHQRVDEWFELEEAPTGKLH
Drerioesyt2a              PRGILRVHFLEGQDLLSKDTYMGGLIKGKSDPYGVIQINNQLFRSKIIKDSLNPRWNEVYEAIVYDGQGQVVFIELFDEDTDHDDFLGSLTMEIDEIQKQQKVDEWFDLIGVPNGKLH
Drerioesyt2b              PKGVLRIYFIEAQNLEVKDTYLGGLIKGKSDPYGMLLVSNQLFRSKTIKECLHPKWNEVYEALVYEPSGQHLEIELFDEDPDKDDFLGSLMIDLTELHKEQKVDEWFDLEEVTTGKLH
Xtropicalisesyt2          PKGVLRIHFLEAQDLMWKDTYMKGLVKGKSDPYGVVRLGNQVFQSKVIKENLNPKWNEVYEALVHEHPGQELEIELFDEDTDKDDFLGSLLIDLVEVEKERVVDEWFTLDEATSGKLH
Acarolinensisesyt2        -XGVLRIHFIEAQDLEGKDTYLKGIIKGKSDPYGIIRVGNQIFQSKVIKENLNPKWNEVYEX-----------------------------------------XWFTLDEVSKGRLH
GgallusESYT2              PKGVLRIHFIEAQDLEGKDTYLKGIVKGKSDPYGIIRVGNQIFQSKVIKENLNPKWNEVYEALVYEHPGQELEIELFDEDPDKDDFLGSLMIDLIEVEKERLLDEWFTLDEVSKGKLH
TguttataESYT2             PKGVLRIHFIEAQDLEGKDNYLKGIVKGKSDPYGIIRVGNQIFQSKVIKENLNPKWNEVYEALVYEHPGQELEIELFDEDPDKDDFLGSLMIDLIEVEKERLLDEWFTLDEVSKGKLH
OanatinusEsyt2            PKGVLRIHFIEAQDLQGKDTYLRGLVKGKSDPYGVIRVGNQIFQSKVIKENLSPKWNEVYEALVYEHPGQELEIELFDEDPDKDDFLGSLMIDLIEVEKERLLDEWFTLDEVPKGKLH
MdomesticaEsyt2           PKGVLRIHFIEAQDLQGKDTYLKGLVKGKSDPYGVIRVGNQIFQSKVIKENLSPKWNEVYEALVYEHPGQELEIELFDEDPDKDDFLGSLMIDLIEVEKERLLDEWFTLDEVPKGKLH
MmusculusEsyt2var1        PKGVLRIHFIEAQDLQGKDTYLKGLVKGKSDPYGIIRVGNQIFQSKVIKENLSPKWNEVYEALVYEHPGQELEIELFDEDPDKDDFLGSLMIDLIEVEKERLLDEWFTLDEVPKGKLH
MmusculusEsyt2var2        PKGVLRIHFIEAQDLQGKDTYLKGLVKGKSDPYGIIRVGNQIFQSKVIKENLSPKWNEVYEALVYEHPGQELEIELFDEDPDKDDFLGSLMIDLIEVEKERLLDEWFTLDEVPKGKLH
MmusculusEsyt2var3        ---------------------------------------------------------------------------------------------------------------------
MmusculusEsyt2var4        PKGVLRIHFIEAQDLQGKDTYLKGLVKGKSDPYGIIRVGNQIFQSKVIKENLSPKWNEVYEALVYEHPGQELEIELFDEDPDKDDFLGSLMIDLIEVEKERLLDEWFTLDEVPKGKLH
HsapiensESYT2var1         PKGVLRIHFIEAQDLQGKDTYLKGLVKGKSDPYGIIRVGNQIFQSRVIKENLSPKWNEVYEALVYEHPGQELEIELFDEDPDKDDFLGSLMIDLIEVEKERLLDEWFTLDEVPKGKLH
HsapiensESYT2var2         PKGVLRIHFIEAQDLQGKDTYLKGLVKGKSDPYGIIRVGNQIFQSRVIKENLSPKWNEVYEALVYEHPGQELEIELFDEDPDKDDFLGSLMIDLIEVEKERLLDEWFTLDEVPKGKLH
HsapiensESYT2var3         PKGVLRIHFIEAQDLQGKDTYLKGLVKGKSDPYGIIRVGNQIFQSRVIKENLSPKWNEVYEALVYEHPGQELEIELFDEDPDKDDFLGSLMIDLIEVEKERLLDEWFTLDEVPKGKLH
HsapiensESYT2var4         ---------------------------------------------------------------------------------------------------------------------
```

```
                                                                   500
Trubripesesyt2a         LRLDWLTPLATPDKLDQALISIKADRTKANDGLSAALLVVFLDSARNLPHNPLEFNQTGLRKASINKAIKSGKKVTSDPSPFVQFRVGHKSFESKTRYKTNEPVWEETHTFLIHNPKT
Trubripesesyt2b         LKLEWLSLLSTPEKLEQVLRSVRADRSLANDGLSSALLVVYLDSAQNLPSNLSDFTYDGSKQVSVFKVLKSAKKSSSEPNPFVQLTVGHKTLDSKIRFKSKDPLWEDCFSFLVHNPRR
Tnigroviridisesyt2a     ------------------------------------------------XRNPLEFNQTGLKKASINKAIKSGKKATSDPSPLVKLTVGHKSYDSKTKYKTNEPVWEETHAFLIQNPRA
Tnigroviridisesyt2b     LKLEWLSLLSTPEKLEQVLRSVRADRSLANDGLSSALLVVYLDSAQNLPSNLSDFSYDGLKQVSVFKVLKSAKKSSSEPNPFVQLTVGHKTLESKIRFKTKDPLWEDCFSFLVHNPRR
Gaculeatusesyt2a        LKLEWLSLLPTPEKLDQALASIKAERGQANDGLSAAVLVVFLDSAKNLPRNPLEFNQAGLRKASLSKAIKSGKKVTSSPSPFVQFTVGHKPLESKIRYKTNEPVWEEAFTFLIHNPKT
Gaculeatusesyt2bvar1    LKLEWLSLLSTPEKLNQVLQSVRAD-SLANDGLSSALLVVHLDSAKNLPSNLSDFTYDGLKQVSVFKALKCAKKSSSEPCPYVQLTVGHKSVESKIRYKTKEPLWEDCFSFFIHNPRR
Gaculeatusesyt2bvar2 ● LKLEWLSLLSTPEKLNQVLQSVRAD-SLANDGLSSALLVVHLDSAKNLP---------------------CAKKSSSEPCPYVQLTVGHKSVESKIRYKTKEPLWEDCFSFFIHNPRR
Olatipesesyt2a          LKLEWLSLLQTPDKLNQVMADIGADRGQANDGPSSAVLIIFLDSAKNLPRNPLEFHPAGPRKASVSELIKT-KKVTSDPNPFVQFRVGHKSFESKTKYKTIQPLWEENFTFLIHNPKK
Olatipesesyt2b          LKLEWLSLFSSPEKLDQVLRSVRADRSLANEGLSSALLVVYLDSAKNLPSNLSDFSYDGLKQVSVFKVLKSAKKNISEPSPYVQFTVGHKTIESKVRYKTKEPLWEDCFSFLVHNPNR
Drerioesyt2a            VKAEWLSLHPTPDKLDEVLSSIKADKGQANDGLSSALLLVHLDSAKNLPRNPLEFNSAGLKKGAVNKAVKSGKKVTSVPNPFVQFTVGHRSFESKTRFKTIEPVWEETFTFLIHNPKC
Drerioesyt2b            LRLEWLSLYSSAEKLDQVQKSIRT-----NDNLSSALLIVNLDSASNLPTNPFVFNPDGPKPP--YKGLKSGKKVSIDPNPFVKLTVGQKTCTSKVRYKTSEPLWEETFPFLINNPQT
Xtropicalisesyt2        LKLEWLTPKSTTENLDQVLKSIKADKDQANDGLSAALLILYLDSARSLPNNPLEINHDGMKKAAVEKAKKAGKKIGSSPNPYVLFSVGHTVQESKVKYKTAEPVWEQTFTFFVHNPKR
Acarolinensisesyt2      LKLEWLTLMPTAENLDQVLKSIRADKDQANDGLSSALLILYLDSARNLPNNPLDYNPDALKKPAVQKALKSGKKINSNPNPLVLLTVGHKAQESKIRYKTNEPVWEENFTFFVHNPRR
GgallusESYT2            LKLEWLTLMPTAENLDKVLTSIRADKDQANDGLSSALLILYLDSARNLPHNPLEFNPDALKKSAVQKALKSGKKLNSNPNPLVLLSVGHKAQESKIRYKTNEPVWEENFTFFVHNPKR
TguttataESYT2           LKLEWLTLMPTADNLDKVLTSIRADKDQANDGLSSALLILYLDSARNLPNNPLEFNPDGLKKAAVQKALKSGKKINSNPNPLVLLSVGHKAQESKIRYKTNEPVWEENFTFFVHNPKR
OanatinusEsyt2          LKLEWLTLMPNAENLDKVLTSIKADKDQANDGLSSALLILYLDSARNLPNNPLEFNHDGLKKAAVQKALKSGKKINSNPNPIVQMSVGHTAQESKIRYKTNEPVWEENFTFFIHNPKR
MdomesticaEsyt2         LKLEWLTLMPNVLNLDKVLTGIKADKNQANDGLSSALLILYLDSARNLPSNPLEFNHDGLKKAAVQKALKSGKKINSNPNPLVQISVGHKAQESKIRYKTNEPVWEENFTFFVHNPKR
MmusculusEsyt2var1      LKLEWLTLMPDAANLDKVLADIRADKDQASDGLSSALLILYLDSARNLP---------------------SGKKINSNPNPLVQMSVGHKAQESKIRYKTSEPVWEENFTFFIHNPRR
MmusculusEsyt2var2    ● LKLEWLTLMPDAANLDKVLADIRADKDQASDGLSSALLILYLDSARNLPSNPLDFNPGVLKKSAVQRALKSGKKINSNPNPLVQMSVGHKAQESKIRYKTSEPVWEENFTFFIHNPRR
MmusculusEsyt2var3      ------------------------------------------------------------------------------------MSVGHKAQESKIRYKTSEPVWEENFTFFIHNPRR
MmusculusEsyt2var4      LKLEWLTLMPDAANLDKVLADIRADKDQASDGLSSALLILYLDSARNLP---------------------SGKKINSNPNPLVQMSVGHKAQESKIRYKTSEPVWEENFTFFIHNPRR
HsapiensESYT2var1       LRLEWLTLMPNASNLDKVLTDIKADKDQANDGLSSALLILYLDSARNLPSNPLEFNPDVLKKTAVQRALKSGKKISSNPNPVVQMSVGHKAQESKIRYKTNEPVWEENFTFFIHNPKR
HsapiensESYT2var2       LRLEWLTLMPNASNLDKVLTDIKADKDQANDGLSSALLILYLDSARNLP---------------------SGKKISSNPNPVVQMSVGHKAQESKIRYKTNEPVWEENFTFFIHNPKR
HsapiensESYT2var3       LRLEWLTLMPNASNLDKVLTDIKADKDQANDGLSSALLILYLDSARNLP---------------------SGKKISSNPNPVVQMSVGHKAQESKIRYKTNEPVWEENFTFFIHNPKR
HsapiensESYT2var4     ● --------------------------------------------------------------------------------------------------------------MILVMPVF

                                                        600
Trubripesesyt2a         QELEVEVKDEKHDCSLGTLTLPLTRLLEAEDMTLNQRFPLKNSGPSCTLKMKIALRVLYFD-TDSSPSSEVPSSPLKTQNSS-NPTPRPSVSSDPHRLPEVSGAP-------SVSPQD
Trubripesesyt2b         QELEVEVKDDKHKCTLGNLTVPLGILLEEEDMTLTQGFPLKNSGPSSIIKLKMALRILSLEKQVSS---DQPSSVQV-R---KSSLAQPPPTAAASPRRSVSD-----------SPLP
Tnigroviridisesyt2a     QKLEVEVKDQKHDCSLGTLTLPLSRLLEAEDMTLNQRFPLKNSGPSCTLKLKIALRVLYYETDSSSPS-EVPSSPAKTQSSS-NPTPRPSVCSDTQRLPSTTSELP---GAASLSPQD
Tnigroviridisesyt2b     QELEVEVKDDKHKCTLGNLTVPLSILLEEEDMTLTQGFPLKNSGPSSIIKLKMALRILCLEKLVSS---DQPSSVQV-R---KSSMPQPPPAAAACPRRSVSD-----------SPLP
Gaculeatusesyt2a        QELQVEVKDKKHDCSLGTLTFPLSRLLEAEDMTLNQRFPLRNSGPNCTLQMKMALRVLCLDKSPSSGSNPAPSSAQVGKNSSASRNPRPSVSPESFVPPASSSSSPPTPSSSSMSAQD
Gaculeatusesyt2bvar1    QELELEVKDDKHKCTLGSLAVSLRGLLDEEGMTLTRCFPLKNSGPSSTIKLKMALRILSMEKQVSS---DQPSSVTV-R---KSSVPLQAPAAPAKRGSASVG-----------SPQP
Gaculeatusesyt2bvar2    QELELEVKDDKHKCTLGSLAVSLRGLLDEEGMTLTRCFPLKNSGPSSTIKLKMALRILSMEKQVSS---DQPSSVTV-R---KSSVPLQAPAAPAKRGSASVG-----------SPQP
Olatipesesyt2a          QELEVEVKDAKHECSMGTISVPLSRLVEAKNMMLNEHFPMKNQGPGSTVKMKMALRVLSLEKDTSPGTRSNPSAVQVHKSSSSSPMPSPSVSSESAMPTPNDPSIP---RSASVSTQD
Olatipesesyt2b          QELEVEVKDGKHKSTLGNLTVPLSSLLSEEDMTLTQCFPLKNSGPSSTVKLKMALRILSLEKNVFS---DLPSAVQV-R---KSSLPQP--ATASSLRPSVSD-----------SLLP
Drerioesyt2a            QDLEVEVKDEKHECSLGTITLPLSQLLKEKQMTMSQRFPLKNSGPGSTLKMKMALRILSLDKLAAS---DKPSSAQVHRAGSVRKTSNATPQRPAVSEPAKTSK---------TQQPA
Drerioesyt2b            QELEIEVKDSKHKCSLGSLQVSLASLLNEVDMTLNQQFPLQSSGPNSTLKLKMALRILSLEKEVTS---SHPSSVRV-RITSQSNTCVPPKPQATSSPTDIQP-----------SQNP
Xtropicalisesyt2        QDLEVEVKDENHQSSMGNLKIPLSQILASEDLTLNQRFHLNNSGPNSSLKMKIALRILPVEKPVRSP--DEQHTSQVKRPSIFKGKQ---PPTPQMP-SPSPA-------------VA
Acarolinensisesyt2      QELEVEVKDEQHQCSLGNFKLPLNQLLASEDLTMHQRFHLSNSGPNSTVNMKIALRILSLEKQERPP--DHQHTAQVKRPSVSKDARK-GSFKPQVPVSPAA--------------SL
GgallusESYT2            QDLEVEVRDEQHQCSLGNFKLPLSQLLESEDLTMHQRFQLSNSGPNSTINMKIALRVLSLEKQARSP--DHQHSAQVKRPSVSKDARK-SSFKPQVPVSPPLD-------------SS
TguttataESYT2           QDLEVEVRDEQHQCSLGNFKLPLSQLLESEDLTMHQRFHLSNSGPNSTINMKIALRVLSLEKPTRSP--DHQHSAQVKRPSLSKDARK-SSFKPQVPVSPTSD-------------PN
OanatinusEsyt2          QDLEVEVKDEQHQCSLGNLKIPLSQLLASDDLTMNQRFQLSNSGPNSTIKMKIALRVLHLEKQERPA--DYQHSAQVKRPSVSKEGRK-ISVKSQVPTPTTAD-------------SS
MdomesticaEsyt2         QDLEVEVKDEQHQCSLGNLKIPLSQLLASDDLTMNQRFQLSNSGPNSTIKMKIALRILYLEKQERSP--DHQHSAQVKRPSVSKEGRKTSSVKSHITAPAALD-------------SS
MmusculusEsyt2var1      QDLEVEVKDEQHQCSLGSLRIPLSQLLTSDNMTINQRFQLSNSGPNSTLKMKIALRVLHLEKQERPP--DYQHSAQVKRPSVSKEGRK-MPIKSQMSASPGTG-------------GA
MmusculusEsyt2var2      QDLEVEVKDEQHQCSLGSLRIPLSQLLTSDNMTINQRFQLSNSGPNSTLKMKIALRVLHLEKQERPP--DYQHSAQVKRPSVSKEGRK-MPIKSQMSASPGTG-------------GA
MmusculusEsyt2var3      QDLEVEVKDEQHQCSLGSLRIPLSQLLTSDNMTINQRFQLSNSGPNSTLKMKIALRVLHLEKQERPP--DYQHSAQVKRPSVSKEGRK-MPIKSQMSASPGTG-------------GA
MmusculusEsyt2var4      QDLEVEVKDEQHQCSLGSLRIPLSQLLTSDNMTINQRFQLSNSGPNSTLKMKIALRVLHLEKQERPP--DYQHSAQVKRPSVSKEGRK-MPIKSQMSASPGTG-------------GA
HsapiensESYT2var1       QDLEVEVRDEQHQCSLGNLKVPLSQLLTSEDMTVSQRFQLSNSGPNSTIKMKIALRVLHLEKRERPP--DHQHSAQVKRPSVSKEGRK-TSIKSHMSGSPGPG-------------GS
HsapiensESYT2var2       QDLEVEVRDEQHQCSLGNLKVPLSQLLTSEDMTVSQRFQLSNSGPNSTIKMKIALRVLHLEKRERPP--DHQHSAQVKRPSVSKEGRK-TSIKSHMSGSPGPG-------------GS
HsapiensESYT2var3       QDLEVEVRDEQHQCSLGNLKVPLSQLLTSEDMTVSQRFQLSNSGPNSTIKMKIALRVLHLEKRERPP--DHQHSAQVKRPSVSKEGRK-TSIKSHMSGSPGPG-------------GS
HsapiensESYT2var4     ● PPCVLQVRDEQHQCSLGNLKVPLSQLLTSEDMTVSQRFQLSNSGPNSTIKMKIALRVLHLEKRERPP--DHQHSAQVKRPSVSKEGRK-TSIKSHMSGSPGPG-------------GS
```

```
                                                 700
                     |         |         |         |         |         |         |         |         |         |         |
Trubripesesyt2a        LQARQRDTEDPFMSSGSAERSV--GNLGDNLADIGRSTGNLA--SSGSQQYQPGGKEPTPSIASDISNPYAAQELQQRLQQLQNGSGPGYFPLGQIQLTVRHSSQRNKLIVVVHSCRN
Trubripesesyt2b        PPTPPPPTD------LSAPTRPQKEGEPYS-ASPRRSTSSLSSYMSSSQKHLSH-KESTPSLVSDISLPFATLELQQRLRQLQNGSAPGQFPLGEVQLTVRHSPQRNKLIVVVHACRN
Tnigroviridisesyt2a    LLAQQRDSGDLLMSPGSADSGKAGGNVGDNPADVGRSTANLA--NSGSQQYQTGAKEPTPSIASDISNPYAAQELQQRLQQLQNGSGPGYFPLGQIQMTIRHSSQRNKLIVVVHACRN
Tnigroviridisesyt2b    PPTPPPPID------VSTLTLPQKDGEPYS-ASPHRSRSSLSGFMSGSQKHLSH-KESTPSLASDISLPFATLELQQRLRQLQNGSASSQFPLGEVQLTVRHSPQRNKLIVVVHGCRN
Gaculeatusesyt2a       LLNRRRESEEPFRSPGGMDMGP--GGGGRGLADTGRSTSNLA--ISGSQQHLAGGKEPTPSIASDISYPYAAQELQQRLQQLQNGSGPSHYPLGEIQLTVRLASQRNKLVVVVHSCRN
Gaculeatusesyt2bvar1   PPTPPPPTPPPPPVAASTLTLQRGDGEPYS-AGPRRSVSDLGACMSGSQKHLPH-KESTPSLASDISLPFATMELHQRLRQLQNGSAPGQFPLGEVQLTIRHSSQRNRLIVVAHACRN
Gaculeatusesyt2bvar2   PPTPPPPTPPPPPVAASTLTLQRGDGEPYS-AGPRRSVSDLGACMSGSQKHLPH-KESTPSLASDISLPFATMELHQRLRQLQNGSAPGQFPLGEVQLTIRHSSQRNRLIVVAHACRN
Olatipesesyt2a         -QQREPTRPTDYKDTGSNLMRK--GGRG-GLAENGRSTSNLA--LSGSQLYLADGKESTPSIASDISNPYATQELQQRLQQLYNGSGLSHPPLGEIRLTIRYSMQRNKLVVIVHSCRD
Olatipesesyt2b         T-TPQHPTK------SSTFTLHHEDGEPYS-ASPCCSTSNLSTCISGSQKHLRH-KESTPSLASDISLPIATLELQQRLRQLQNGSGLGQFPLGEVQLTVRHSSQRNKLIVVVHACRN
Drerioesyt2a           PAPRTQPTPTPSPRVEPVTDRKPLEESPPHLAKSGKSMSNLA--ISGSNLHL-NSKEQTPSIASDISNLAATQELQKTIQHLHNGASPGFAPLGEIELTIRHSPQRNKLIVVVHKCRN
Drerioesyt2b           PCTPPPDTP---------QGKRGSDGSPVRLADAGRSLSSLG--IGSSHKRQSH-RDSTPSLASDISLPCATLELQQRLQQLQNGSGLNLYPLGEVQLTVRHSSQRNKLIVVVHACRN
Xtropicalisesyt2       HKPPPTPKLDTNKKLENGNKGSTPSASPKRPTELHKSSSSLSGSSFTHSPSHPSAKEPTPSIASDISLPVATQELRERLRQLQNGTTLGQSPLGQIQLTIRHSSQRNKLIVVVHSCRN
Acarolinensisesyt2     NKPPAPAAADTDKKTEAVEKGPPSNPSPQWPTDLSRSSSSLLASNLSYSPSHLSVKEPTPSIASDISLPIATQELRQRLRQLENGTTLGQSPLGQIQLTIRHSSQRNKLMVVVHTCRN
GgallusESYT2           KHAPASPVADSDKKTDVAEKSQPPNASPQWPTDLSRSSSSLHASNFNYSPSHLSVKEPTPSIASDISLPIATQELRQRLRQLENGTTLGQSPLGQIQLTIRHSSQRNKLIVVVHSCRN
TguttataESYT2          KPVPASPVTDSDKKTDTADKNQPPNASPQWPTDLSRSSSSLHASNFSCSPSHLSVKEPTPSIASDISLPIATQELRQRLRQLENGTTLGQSPLGQIQLTIRHSSQRNKLIVVVHSCRN
OanatinusEsyt2         KTVPTTPVTDS-RKADLGEKGHPANASPQWPTELSRSSSSLHASNFAYSPSHLSVKEPTPSIASDISLPIATQELRQRLRQLENGTTLGQSPLGQIQLTIRHSSQRNKLIVVVHSCRN
MdomesticaEsyt2        KMVSAPPLSENPRKSDVDEKGQPPNASPQWPSDLSQSSSSLPTSNVTSSPSHLSIKEPTPSIASDISLPIATQELRQRLRQLENGTTLGQSPLGQIQLTIRHSSQRNKLIVVVHSCRN
MmusculusEsyt2var1     NTAPSTPVMGVDDKPAMEEKPQPPEASPLGHRDLGRSSSSLLAS-----PSHIAAKEPTPSIASDISLPIATQELRQRLRQLENGTTLGQSPLGQIQLTIRHSSQRNKLIVVVHSCRN
MmusculusEsyt2var2     NTAPSTPVMGVDDKPAMEEKPQPPEASPLGHRDLGRSSSSLLAS-----PSHIAAKEPTPSIASDISLPIATQELRQRLRQLENGTTLGQSPLGQIQLTIRHSSQRNKLIVVVHSCRN
MmusculusEsyt2var3     NTAPSTPVMGVDDKPAMEEKPQPPEASPLGHRDLGRSSSSLLAS-----PSHIAAKEPTPSIASDISLPIATQELRQRLRQLENGTTLGQSPLGQIQLTIRHSSQRNKLIVVVHSCRN
MmusculusEsyt2var4     NTAPSTPVMGVDDKPAMEEKPQPPEASPLGHRDLGRSSSSLLAS-----PSHIAAKEPTPSIASDISLPIATQELRQRLRQLENGTTLGQSPLGQIQLTIRHSSQRNKLIVVVHSCRN
HsapiensESYT2var1      NTAPSTPVIGGSDKPGMEEKAQPPEAGPQGLHDLGRSSSSLLAS-----PGHISVKEPTPSIASDISLPIATQELRQRLRQLENGTTLGQSPLGQIQLTIRHSSQRNKLIVVVHACRN
HsapiensESYT2var2      NTAPSTPVIGGSDKPGMEEKAQPPEAGPQGLHDLGRSSSSLLAS-----PGHISVKEPTPSIASDISLPIATQELRQRLRQLENGTTLGQSPLGQIQLTIRHSSQRNKLIVVVHACRN
HsapiensESYT2var3      NTAPSTPVIGGSDKPGMEEKAQPPEAGPQGLHDLGRSSSSLLAS-----PGHISVKEPTPSIASDISLPIATQELRQRLRQLENGTTLGQSPLGQIQLTIRHSSQRNKLIVVVHACRN
HsapiensESYT2var4      NTAPSTPVIGGSDKPGMEEKAQPPEAGPQGLHDLGRSSSSLLAS-----PGHISVKEPTPSIASDISLPIATQELRQRLRQLENGTTLGQSPLGQIQLTIRHSSQRNKLIVVVHACRN

                                            800
                     |         |         |         |         |         |         |         |         |         |         |
Trubripesesyt2a        LIAFTDHGSDPYVRLYLLPDKRRSGRRKTHTLKRNLNPVYDQTFEFTVSLVELHRRTLDVAVKNGGSLLSKHKGLLGKVLVDLTHEDISKGFTQWYDLSEDGLRKPH-----------
Trubripesesyt2b        LIAFTKDGSDPFIRLYLLPDKSRTGKRKTTTVKRTLNPIYDQTFEFSVSVVELHRRTLDVAVKNGGGLLSKHKGLLGKVLVDLSSEDISKGWTQWYDLSEDGCSSQL-----------
Tnigroviridisesyt2a    LIAFTDHGSDPYVRLYLLPDKRRSGRRKTHMRKRSLNPLYDETFEFTVSLVELHRRTLDVAVKNGGSLLSKHKGLLGKVLVDLTHEDISKGFTQWYDLSEDGLMKLH-----------
Tnigroviridisesyt2b    LIAFTKDGSDPFIRLYLLPDKSRPGRRKTTTAKRTLNPVYDQTFEFSVSVVELHRRTLDVAVKNGGSILSKHKGLLGKVLVDLSSVDIAKGWTHWYDLSEDGSSSHL-----------
Gaculeatusesyt2a       LIAFTDHGSDPYVRLYLLPDKRRSGRRKTHTIKKTLNPVYDQTFEFNVSLVELHRRTLDVAVKNGGGLLSKHKGLLGKVLVDLTHEESNKSLTQWYELSEDGLTKSHQL---------
Gaculeatusesyt2bvar1   LIAFTKDGSDPFIRLYLLPDKRRTGRRKTSTMKRTLNPVYDQTFEFSVSMVELHRRTLDVAVKNGGNILSKHKGLLGKVLVDLSGDDISRGYTQWYELSEGGPSSQSIRSIPDSLLTQ
Gaculeatusesyt2bvar2   LIAFTKDGSDPFIRLYLLPDKRRTGRRKTSTMKRTLNPVYDQTFEFSVSMVELHRRTLDVAVKNGGNILSKHKGLLGKVLVDLSGDDISRGYTQWYELSEGGPSSQSIRSIPDSLLTQ
Olatipesesyt2a         LISFTDHGSDPYVRLYLLPDKRRSGRRKTHTIKKSLSPVYDKPFEFDVSLVELHRRILDVAVKNGGGILSKHRGLLGKVQVDLNFEEIHKGLTLX-----------------------
Olatipesesyt2b         LIAFTKDSSDPFIRLYLLPDKSRTGRRRTSTMKKTLNPVYDQTFEFSVSMVELHRRTLDVAVKNGGSILFKHKGLLGKVLVDLSGDDILKGCTQWYNLSEDGLASELRRSSQEAVPS-
Drerioesyt2a           LISASQNGSDPYVRLYLLPDKRRSGRRKTSTAKKTVNPVFDQTFEFTVSIVELQKRTLDVAVKNGGGILAKHRGLLGKVIVEFNLEDPSKSSTQWYELSVDGFKRPSP----------
Drerioesyt2b           LIALNKDGSDPYIRLYLLPDKSRSGRRKTSTLKKTLNPIYDQSFEFSVSMVELHRRTLDIAVKNGGGLLSKNKLLLGKX---------------------------------------
Xtropicalisesyt2       LIAFSEEGSDPYVRMYLLPDKRRSGRRKTHVYKKTLNPVYDQTFEFSVSLPELQRRTLDIAVKNSGGFLSRDKGLLGKLLLELNAEDAVKGWTLWYDLTEDGTRPAVSS---------
Acarolinensisesyt2     LIAFSEEGSDPYVRMYLLPDKKRSGRRKTSVSKKNLNPVFDQAFDYSVSLADLQKRTLDVAVKNSGGFLSKDKGLLGKVLIPLASEELSKNFTQWYDLTEDGTRPHVSP---------
GgallusESYT2           LIAFSEEGSDPYVRMYLLPDKRRSGRRKTHVSKKTLNPVFDQIFDFSVSLPEVQRRTLDVAVKNSGGFLSKDKGLLGKVLIPLTSEELAKGWTQWYDLTEDGTRPHGAS---------
TguttataESYT2          LIAFSEEGSDPYVRMYLLPDKRRSGRRKTHVSKKTLNPVFDQMFDFSVSLPEVQRRTLDVAVKNSGGFLSKDKGLLGKLLIPLASEELAKGWTQWYDLTEDGTKPNGAS---------
OanatinusEsyt2         LIAFSEDGSDPYVRMYLLPDKRRSGRRKTHVSKKTLNPVFDQSFDFSVSLPEVQRRTLDVAVKNSGGFLSKDKGLLGKLLIALASEELAKGWTQWYDLTEDGSRPHVVN---------
MdomesticaEsyt2        LIAFSEDGSDPYVRLYLLPDKRRSGRRKTHVSKKTLNPVFDQSFDFSVSLPEVQRRTLDVAVKNSGGFLSKDKGLLGKLLVALASEDLAKGWTQWYDLTEDGTRPQAVN---------
MmusculusEsyt2var1     LIAFSEDGSDPYVRMYLLPDKRRSGRRKTHVSKKTLNPVFDQSFDFSVSLPEVQRRTLDVAVKNSGGFLSKDKGLLGKVLVVLASEELAKGWTQWYDLTEDGTRPQVIT---------
MmusculusEsyt2var2     LIAFSEDGSDPYVRMYLLPDKRRSGRRKTHVSKKTLNPVFDQSFDFSVSLPEVQRRTLDVAVKNSGGFLSKDKGLLGKVLVVLASEELAKGWTQWYDLTEDGTRPQVIT---------
MmusculusEsyt2var3     LIAFSEDGSDPYVRMYLLPDKRRSGRRKTHVSKKTLNPVFDQSFDFSVSLPEVQRRTLDVAVKNSGGFLSKDKGLLGKVLVVLASEELAKGWTQWYDLTEDGTRPQVIT---------
MmusculusEsyt2var4     LIAFSEDGSDPYVRMYLLPDKRRSGRRKTHVSKKTLNPVFDQSFDFSVSLPEVQRRTLDVAVKNSGGFLSKDKGLLGKVLVVLASEELAKGWTQWYDLTEDGTRPQVIT---------
HsapiensESYT2var1      LIAFSEDGSDPYVRMYLLPDKRRSGRRKTHVSKKTLNPVFDQSFDFSVSLPEVQRRTLDVAVKNSGGFLSKDKGLLGKVLVALASEELAKGWTQWYDLTEDGTRPQAMT---------
HsapiensESYT2var2      LIAFSEDGSDPYVRMYLLPDKRRSGRRKTHVSKKTLNPVFDQSFDFSVSLPEVQRRTLDVAVKNSGGFLSKDKGLLGKVLVALASEELAKGWTQWYDLTEDGTRPQAMT---------
HsapiensESYT2var3      LIAFSEDGSDPYVRMYLLPDKRRSGRRKTHVSKKTLNPVFDQSFDFSVSLPEVQRRTLDVAVKNSGGFLSKDKGLLGKVLVALASEELAKGWTQWYDLTEDGTRPQAMT---------
HsapiensESYT2var4      LIAFSEDGSDPYVRMYLLPDKRRSGRRKTHVSKKTLNPVFDQSFDFSVSLPEVQRRTLDVAVKNSGGFLSKDKGLLGKVLVALASEELAKGWTQWYDLTEDGTRPQAMT---------
```
