# Supplementary material for: A manual collection of Syt, Esyt, Rph3a, Rph3al, Doc2, and Dblc2 genes from 46 metazoan genomes - an open access resource for neuroscience and evolutionary biology
Source: BMC Genomics. 2010 Jan 15;11:37. doi: 10.1186/1471-2164-11-37 (PMC2823689; doi:10.1186/1471-2164-11-37)
Supplement: Additional file 46 — Alignment of the vertebrate Esyt3 sequences. Amino acid position is marked every hundred amino acids approximately, at the top of each page of the alignment. Splice variants are included and highlighted with black dots where they differ. Intron position and phase is indicated with a coloured bar between amino acids. Black bars indicate phase 0 introns. Red bars indicate phase +1 introns. Blue bars indicate phase +2 introns. Some intron positions are marked with dotted lines. In these cases, transcript sequence covers a gap in the genomic sequence and the intron presence is assumed. X residues indicate where a portion of sequence is missing. [file 1471-2164-11-37-S46.PDF]

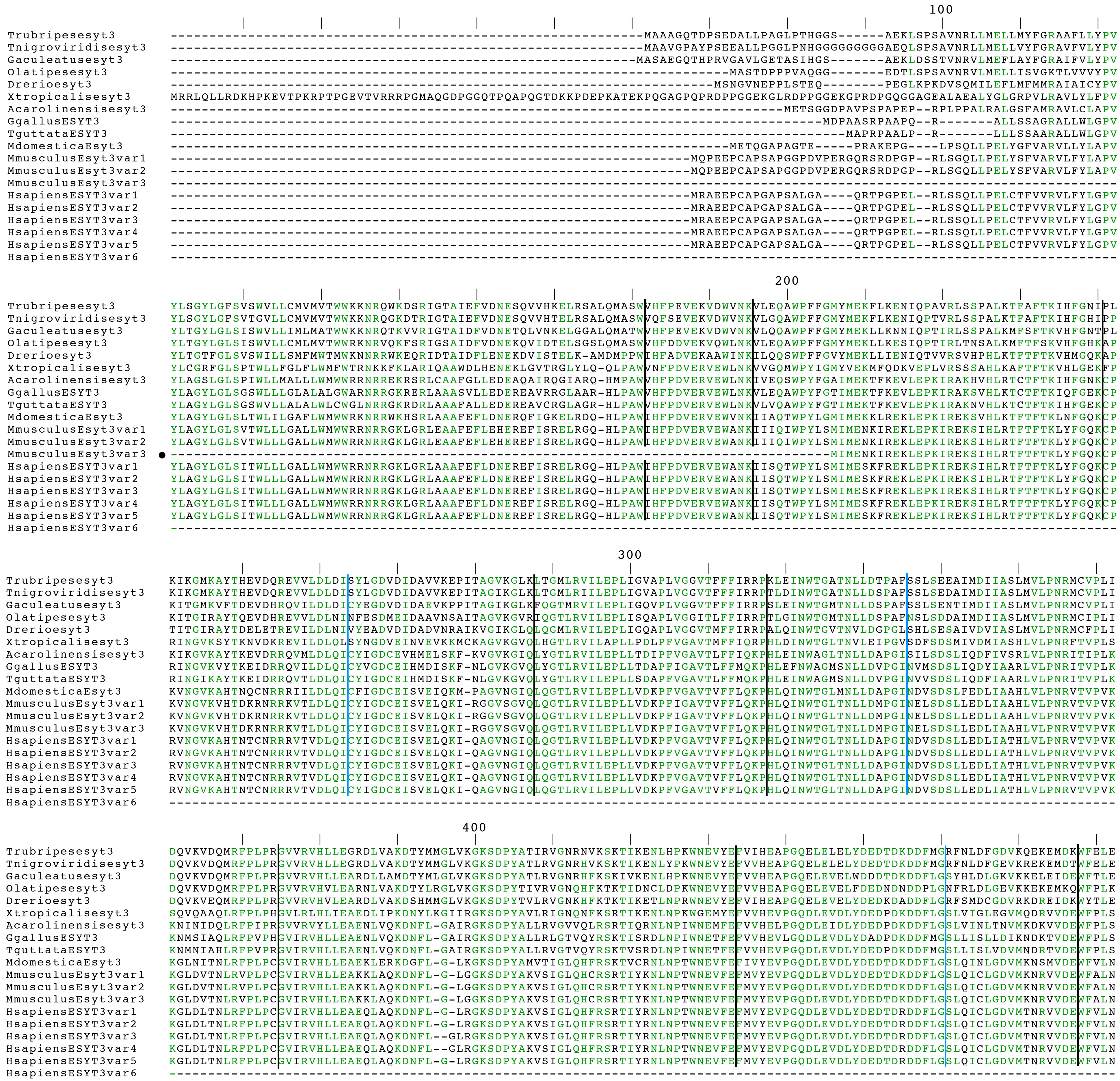
100
200
300
400
Trubripesesyt3
Tnigroviridisesyt3
Gaculeatusesyt3
Olatipesesyt3
Drerioesyt3
Xtropicalisesyt3
Acarolinensisesyt3
GgallusESYT3
TguttataESYT3
MdomesticaEsyt3
MmusculusEsyt3var1
MmusculusEsyt3var2
MmusculusEsyt3var3
HsapiensESYT3var1
HsapiensESYT3var2
HsapiensESYT3var3
HsapiensESYT3var4
HsapiensESYT3var5
HsapiensESYT3var6

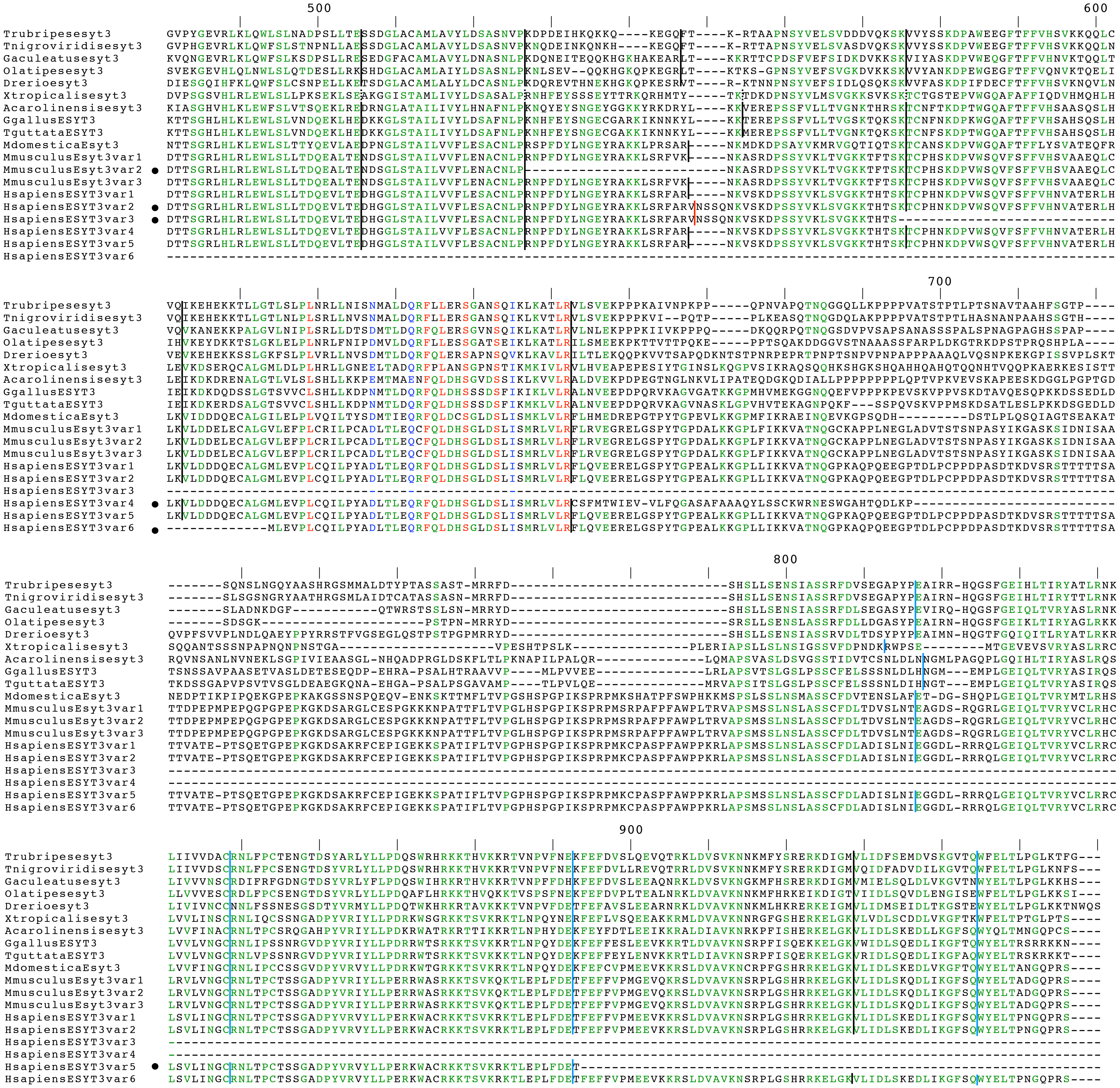
500
600
700
800
900
Trubripesesyt3
Tnigroviridisesyt3
Gaculeatusesyt3
Olatipesesyt3
Drerioesyt3
Xtropicalisesyt3
Acarolinensisesyt3
GgallusESYT3
TguttataESYT3
MdomesticaEsyt3
MmusculusEsyt3var1
MmusculusEsyt3var2
MmusculusEsyt3var3
HsapiensESYT3var1
HsapiensESYT3var2
HsapiensESYT3var3
HsapiensESYT3var4
HsapiensESYT3var5
HsapiensESYT3var6
