# Supplementary material for: A manual collection of Syt, Esyt, Rph3a, Rph3al, Doc2, and Dblc2 genes from 46 metazoan genomes - an open access resource for neuroscience and evolutionary biology
Source: BMC Genomics. 2010 Jan 15;11:37. doi: 10.1186/1471-2164-11-37 (PMC2823689; doi:10.1186/1471-2164-11-37)
Supplement: Additional file 47 — Alignment of the invertebrate Rabphilin sequences. Amino acid position is marked every hundred amino acids approximately, at the top of each page of the alignment. Splice variants are indicated. Intron position and phase is indicated with a coloured bar between amino acids. Black bars indicate phase 0 introns. Red bars indicate phase +1 introns. Blue bars indicate phase +2 introns. The five conserved acidic amino acids in each C2 domain are indicated by pink dots at the top of the alignment. X residues indicate where a portion of sequence is missing. [file 1471-2164-11-37-S47.PDF]

```
                                                                                                           100
Celegansrbf_1var1     MTKSTKLRHCKQKKKKPEKTPKGNPPILITEK-SIEDAATTTSTTDALLGSPE------GSRSKSRKLKL---CCCTAQAATLSPLDPTSYGGIASTSAHNGMVGGLSRDSRAASRTSKRGSSKS--
Cbrennerirbf_1var1    MTKSTKLRHCKPKKKKPEKPEKNKPPILVTERPSTDDVATTSSTTDALLGSTDE----GGSRSKSRSRKLGGLCCCTAQTATLSPLDPTGYGGIASTNANNGMVGGLSRESRAPSRTSRKGSSRSQ-
Cbriggsaerbf_1var1    MTKSTKLRHCKQKKKKPEKAEKSKTPIVITEKPSFEDVAITSSTTDGLLGSTDS----GGSRSKSRNRKLGGLCCCTAQTATLSPIEPTDYGGIASTN-NNGMIGSLCRDSRAPSRNSRSGSSRSQQ
Cremaneirbf_1var1     MTKSTKLRHCKQKKKKPEKAEKNKPPILVTERASSDDVATTSSTTDGLLGSTDGEAYGGASRSKSRTRKLGGLCCCTAQTATLSPLDPIDYGGIASTSANNGMVGGLSRDSRAASRTSRRGSSRSQ-
Cjaponicarbf_1var1    MTKSTKLRHASNKKDKKEKKRKKS-------KLAPPDEPPHTRT---------------GEESK---RRMGGTLCCCSKPSVLSPLDHSSYGGIASTSGAQNGMGGISRESRANSRASRRGSSRS--

                                                                                           200
TadhaerensRph         --------------------------------------MTDRNRK-ASTNAWICPNDRQLCLRAKLNIGWSYHSDS--------------------P--RGSGN----ELAQNEKKQIMQVLQKAEN
NvectensisRph         --------------------------------------MGDIEVE-AE-NRWTCPNDRQLVLRAKLNAGWSFHSGG--------------------PAKKPAKT----TISEAEQEIIKNVIQRADR
CapitellaRph          ------------------------------------------MADVNRMDRWVCPNDRQLALRAKLGSGWSVHTNK--------------------MQGFRREE----QLNCDEQECIMRVIKRAEM
HrobustaRph           ------------------------------------------------METWVCPNDRQLALRAKLGSGWSIHTNR--------------------KTPCSKQE----SLTSEEMDRITKVIKQAEK
LgiganteaRph          ------------------------------------------------MDRWVCPNDRQLALRAKLGTGWSVHTNR--------------------LSSFQKAK----QLNLDEQEQIMRVMQRAEY
CsavignyiRph          ------------------------------------------MAEDAV-HTWVCPSDRQLTLRSKLRAGWSVRASQ--------------------SKRHKRVS----AISGDELKHIRNVMLRAEN
CintestinalisRph      --------------------------------------MLTGNAEDAAQHAWVCPSDRQLTLRSKLRAGWSVRAAQ--------------------TDRQRRSS----TLSDEELKKIRQVIERAEN
SpurpuratusRph        ---------------------------------------MGEFAGGNAVDRWTCPNDRQLALRAKLGTGWSFHTNS--------------------AKKFQKSE----GLNRDEQDTILKVIEKAEK
BfloridaeRph          ---------------------------------------MGEMASGGRIDRWVCPNDRQLALRAKLNAGWSFHTDK--------------------LTSFQKQP----PLSKEEQDAIRKVMEKADF
IscapularisRph        ------------------------------------------MGDYGEKDKWVCPNDRELALRAKLQTGWSVKTGA--------------------MSTFTRQE----QLNDSEQERIVGVIKRADM
DpulexRphl            -----------------------------------------MEDDANAGDAWVCPNDRQLALRAKLHFGWSSAKSKRIDSNPTTATHCRPTQQQQSTGRPAGSTSTIEPLTQEEQERVFQVIQRAEE
TcastaneumRph         --------------------------------------MVDCGAN-DATNRFVCPNDRQLALRAX--------------------------------------------------------------
AmelliferaRph         --------------------------------------MDMTVKDISGGTRWVCPNDRHLSLRAKLQTGWSVKTAS---------LDSKWGTFSNSYISGTNRCAQSFVLSEEEQQTIIQVIQRAEA
DmelanogasterRph      --------------------------------------MDFQNRN-NTANKFVCPSDRQLALRAKLKAGWSSS--------------------------KTSEP----LR-PEEQEAIISVIRRNEE
DsimulansRph          --------------------------------------MDFQNRN-NTANKFVCPSDRQLALRAKLKAGWSSS--------------------------KTSEP----LR-PEEQEAIISVIRRNEE
DsecheliaRph          --------------------------------------MDFQNRN-NTANKFVCPSDRQLALRAKLKAGWSSS--------------------------KTSEP----LR-PEEQEAIISVIRRNEE
DerectaRph            --------------------------------------MDFQNRN-NTANKFVCPSDRQLALRAKLKAGWSSS--------------------------KTSEP----LR-PEEQEAIISVIRRNEE
DyakubaRph            --------------------------------------MDFQNRN-NTANKFVCPSDRQLALRAKLKAGWSSS--------------------------KTSEP----LR-PEEQEAIISVIRRNEE
DananassaeRph         --------------------------------------MDFQNRN-NTANKFVCPSDRQLALRAKLKAGWSSS--------------------------KSSEP----LR-PEEQEAIISVIRRNEE
DpseudoobscuraRph     --------------------------------------MDFQNRN-NTANKFVCPSDRQLALRAKLRAGWSSS--------------------------KTSEP----LR-PEEQEAIISVIRRNEE
DpersimilisRph        --------------------------------------MDFQNRN-NTANKFVCPSDRQLALRAKLRAGWSSS--------------------------KTSEP----LR-PEEQEAIISVIRRNEE
DwillistoniRph        --------------------------------------MDFQNRN-NTANKFVCPSDRQLALRAKLKAGWSSS--------------------------KTSEP----LR-PEEQEAIISVIRRNEE
DvirilisRph           --------------------------------------MDFANRN-NTANKFVCPSDRQLALRAKLKAGWSSS--------------------------KTNEP----LR-PDEQEAIISVIRRNEE
DmojavensisRph        --------------------------------------MDFANRN-NTANKFVCPSDRQLALRAKLKAGWSSS--------------------------KTNEP----LR-PEEQEAIISVIRRNEE
DgrimshawiRph         --------------------------------------MDFANRN-NTANKFVCPSDRQLALRAKLKAGWSSS--------------------------KTNEP----LR-PDEQEAIISVIRRNEE
Celegansrbf_1var1     LNRPQIDADEPSTSGTNPDRRPST-HFVLDLPVVSTRYLMNDWEIGGTQNKWVCPSDRHLHLRAQLKSGWSVRTAT--------------------ARSPTNSKAQTGSITAAEQEHIQKVLAKAEE
Cbrennerirbf_1var1    TKQPPIDPDEPSTSNT-AERRPST-HFMLDIPVVSNRYLMNDWEIGGTQNKWVCPSDRHLQLRAQLKSGWSVRTAT--------------------ARSPTNSKAQTGSITAAEQEHIQRVLAKAEE
Cbriggsaerbf_1var1    AKHQPIDPDEPSTSGT-TDRRPST-HFMLDLPVVSTRYLMNDWEIGGTQNKWVCPSDRHLHLRAQLKSGWSVRTAT--------------------ARSPTNSKAQTGSITAAEQEQIQRVLAKAEE
Cremaneirbf_1var1     AKQP-IDPDEPSTSGT-AERRPST-HFMLDLPVVSTRYLMNDWEIGGTQNKWVCPSDRHLHLRAQLKSGWSVRTAT--------------------ARSPTNSKAQTGSITAAEQEHIQRVLAKAEE
Cjaponicarbf_1var1    --TARHDADEPSTSN--ADRRPSTSHFMLDLPVVSTRYLMNDWEIGGTQNKWVCPSDRHLHLRAQLKSGWSVRTAT--------------------ARSPTNSKSQTGAITAAEQEHIQRVLAKAEE
                                               ↑
                                    var2 forms begin here
                                                                         300
TadhaerensRph         LEKMEQERIGRLVEKLDNMKNHAVG-DGERSCILCGVTFS-TFGTTPTPC-----NDCKKGVCSK-CGVDTY---------------------NSRKQPLWLCKLCSEQRE-LLKRSGAWFYKSM
NvectensisRph         IQLQEEGRVGRLVEKLENLRNNAIG-NGRDTCLLCASKFG-LLKTIDKEC-----DICEKNVCEK-CGVDTH---------------------NSVGQPIWLCVLCSEQRE-LWKKTGAWFFKSI
CapitellaRph          IDNLEMERVGRLVDRLENMKKNSIG-NGNSQCVLCADEFGLLAASP-TYC-----DDCKKAVCTK-CGVDTFNSHH---------------------QPLWLCKICSENRE-LWKRSGAWFFKGI
HrobustaRph           VEQFEMYRIGYLVDKLETMKMNALG-DGRNQCVLCADQFK-ILSKSPSVC-----SDCNKNVCSK-CSVETV---------------------NSKSKVIDLCKICSEKRE-LWRKSGAWFYKGL
LgiganteaRph          LDDSEQERIGRLVEKLENMKKNSIG-NGTTQCVLCGDEFG-LLGSSPTFC-----DDCKKAVCSK-CGVDTVNSM---------------------RQPLWLCKICSEQRE-VWKRSGAWFFKAI
CsavignyiRph          MEKAEQKRVGKLVHRLEDMRNNSAG-DGQSQCILCAEVFGKFIGATPYVC-----VDCKKNVCNK-CCVEFSPLLHHSGDFKADGGRGRGRETPSPGPNKWLCKICSEGRE-LWKRSGAWFFSSL
CintestinalisRph      MDQAEQKRVGKLVHRLDDMRSNSAG-DGKLRCILCAEAFGKLMGASSFVC-----VDCKKNVCSK-CSVEFSPHLIR-GSHSMDSSRGRSSNRKS-STTKWLCKICSEGRE-LWKRSGAWFFQSL
SpurpuratusRph        LEVSEQERIGRLVDKLDNMKKNSLG-NGTTQCILCGDEFG-LLGASPMTC-----YDCYKAVCSK-CGVDTTNSI---------------------KQPIMLCKLCSETRE-LWKRSGAWFFKAL
BfloridaeRph          MEQMEQERVGCLVDRLDNMKKAAKG-NGTTQCVLCGDAFR-MLGSSPLTC-----CDCKKHVCSK-CGVELTTKF---------------------GSQSYLCKICSESRE-MWKRSGAWFFKGL
IscapularisRph        LEQMEQQRVGRLVERVENMKRNALG-NGTTQCVLCANEFG-LLSGSPLLC-----NDCRKAVCSK-CSVDTF---------------------SAQREQIWLCKICSETRE-MWKKSGAWFYRAL
DpulexRphl            VSENEQLRVGKLVDRVRNIQKNAVGGDGANQCVLCGDYASVFLSWSLTSARHVTCKDCLKAVCQK-CSVDTSV---------------------NPREPTYLCKLCSERRE-TWKKSGAWFYKIT
TcastaneumRph         --------VGRLVERLENMRRNALG-RSPSQCLLCGDGFG-MLGAQKVLC-----IDCRRLVCQK-CAIETY---------------------TKAARNYWLCMICAETRE-IWKKSGAWFFKSI
AmelliferaRph         LNLSEQERIGRLVEKLENMKRNV--------CIVTTTRLN-EKKRCSSRC-----FNSAHCVCS--CALCGE----------------------KFGARQYLCRICAETRE-MWKKSGAWFIKGM
DmelanogasterRph      IEVAERQRVGRLVERVEKIKQHAVE-RGPNCCRLCGDTFG-ILRPQRILC-----EDCRQSVCTK-CSVDINIRYH----------------TSERSREIWLCRICSETRE-MWKKSGAWFFKGL
DsimulansRph          IEVAERQRVGRLVERVEKIKQHAVE-RGPNCCRLCGDTFG-ILRPQRILC-----EDCRQSVCAK-CSVDINIRYH----------------TSERSREIWLCRICSETRE-MWKKSGAWFFKGL
DsecheliaRph          IEVAERQRVGRLVERVEKIKQHAVE-RGPNCCRLCGDTFG-ILRPQRILC-----EDCRQSVCAK-CSVDINIRYH----------------TSERSREIWLCRICSETRE-MWKKSGAWFFKGL
DerectaRph            IEVAERQRVGRLVERVEKIKQHAVE-RGPNCCRLCGDAFG-ILRPQRILC-----EDCRQSVCAK-CSVDINIRYH----------------TSERSREIWLCRICSETRE-MWKKSGAWFFKGL
DyakubaRph            IEVAERQRVGRLVERVEKIKQHAVE-RGPNCCRLCGDTFG-ILRPQRILC-----EDCRQSVCAK-CSVDINIRYH----------------TSERSREIWLCRICSETRE-MWKKSGAWFFKGL
DananassaeRph         IEVAERLRVGRLVERVEKIKQHAVE-RGPNCCRLCGDTFG-LLRGHRIIC-----EDCRQAVCTK-CSLDINIRYH----------------TSEKSREIWLCRICSETRE-MWKKSGAWFFKGL
DpseudoobscuraRph     IEIAERQRVGRLVERVEKIKQHAVE-RGPNCCRLCGDTFG-LLRPHRIIC-----EDCRQSVCTK-CSVDINIRYH----------------TSERTREIWLCRICSETRE-MWKKSGAWFFKGL
DpersimilisRph        IEIAERQRVGRLVERVEKIKQHAVE-RGPNCCRLCGDTFG-LLRPHRIIC-----EDCRQSVCTK-CSVDINIRYH----------------TSERTREIWLCRICSETRE-MWKKSGAWFFKGL
DwillistoniRph        IEIAERQRVGRLVERVEKIKQHAVE-RGPNCCRLCGDAFG-LLRPHRIIC-----EDCRQAVCTK-CSVDINIRYH----------------TSERTREIWLCRICSETRE-MWKKSGAWFFKGL
DvirilisRph           IEIAERQRVGRLVERVEKIKLHAVE-RGPNCCRLCGDAFG-LLRPQRIIC-----EDCRKSVCTK-CSVDINIRYH----------------NSERTREIWLCRICSETRE-MWKKSGAWFFKGL
DmojavensisRph        IEIAERQRVGRLVERVEKIKLHAVE-RGPNCCRLCGDAFG-LLRPQRILC-----EDCRKAVCTK-CSVDINIRYH----------------NSERTREIWLCRICSETRE-MWKKSGAWFFKGL
DgrimshawiRph         IEIAERQRVGRLVERVEKIKLHAVE-RGPNCCRLCGDAFG-LLRPQRILC-----EDCRKSVCAK-CSVDINIRYH----------------NSERTREIWLCRICSETRE-MWKKSGAWFFKGL
Celegansrbf_1var1     SKSKEQQRIGKMVDRLEKMRRRATG-NGVTHCLLCHTEFGLLASKSYAAMC----VDCRKYVCQRNCGVETTDVNQ----------------TTGKVETVFLCKICSEAREVLWKKSGAWFYKEM
Cbrennerirbf_1var1    SKSKEQQRIGKMVDRLEKMRRRATG-NGVTHCLLCHTEFGLLASKSYAAMC----VDCRKYVCQRNCGVETTDVNQ----------------STGKVETVFLCKICSEAREVLWKKSGAWFYKEM
Cbriggsaerbf_1var1    SKNKEQQRIGKMVDRLEKMRRRATG-NGVTHCLLCHTEFGLLAAKSYAAMC----TDCRKYVCQRNCGVETTDVQ------------------SGKTETVFLCKICSEAREVLWKKSGAWFYKEM
Cremaneirbf_1var1     SKTKEQQRIGKMVDRLEKMRRRATG-NGVTHCLLCHTEFGLLASKSYAAMC----VDCRKYVCQRNCGVETTDVSQ----------------TTGKVETVFLCKICSEAREVLWKKSGAWFYKEM
Cjaponicarbf_1var1    SKNKESQRIGKMVDRLEKMRRRATG-NGVTHCLLCHTEFGLLASKSYAAMC----VDCRKYVCQRNCGVETTDVNQ----------------TTGKVETVFLCKICSEAREVLWKKSGAWFYKEM
```

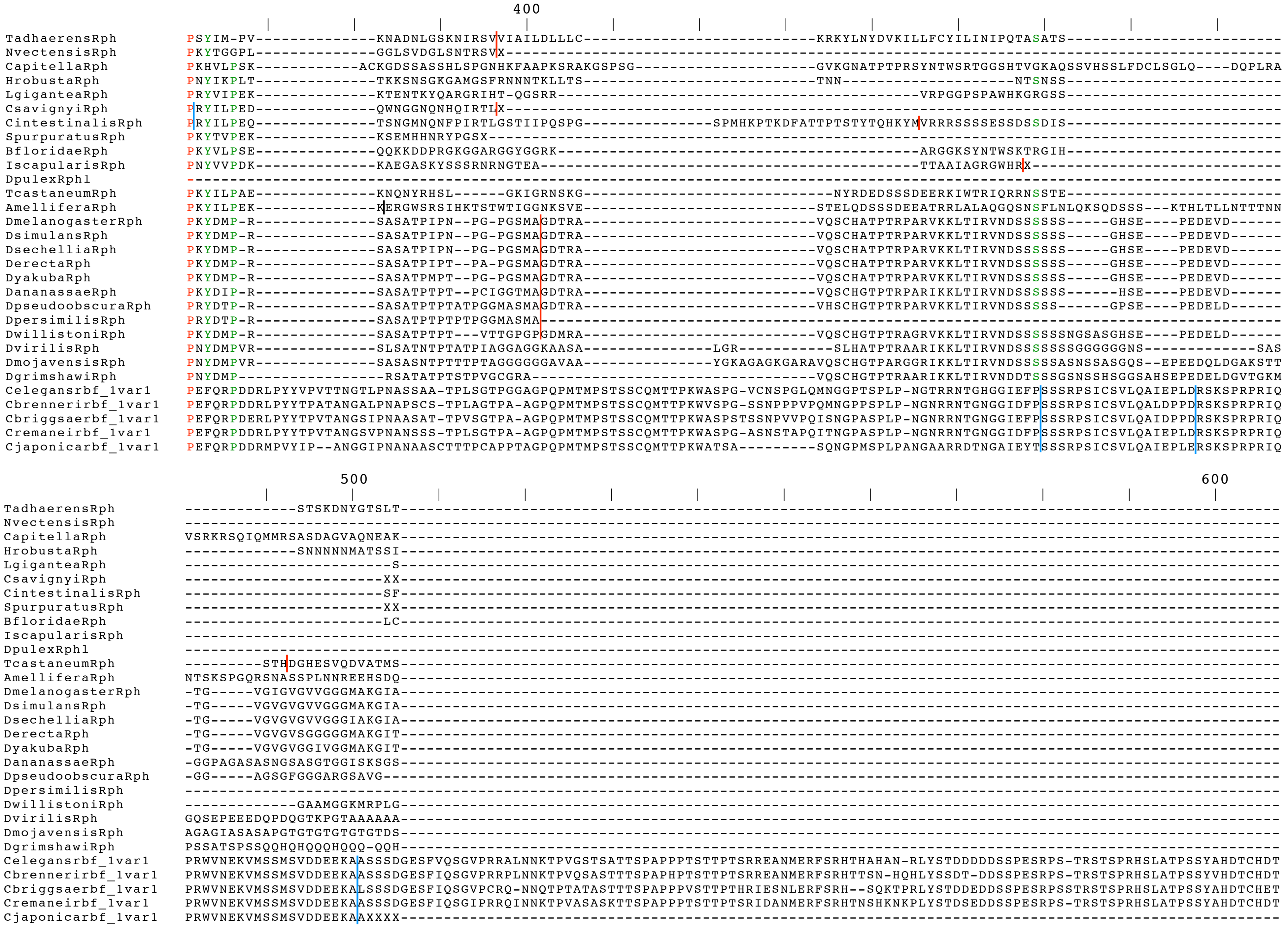

400
TadhaerensRph
NvectensisRph
CapitellaRph
HrobustaRph
LgiganteaRph
CsavignyiRph
CintestinalisRph
SpurpuratusRph
BfloridaeRph
IscapularisRph
DpulexRphl
TcastaneumRph
AmelliferaRph
DmelanogasterRph
DsimulansRph
DsechelliaRph
DerectaRph
DyakubaRph
DananassaeRph
DpseudoobscuraRph
DpersimilisRph
DwillistoniRph
DvirilisRph
DmojavensisRph
DgrimshawiRph
Celegansrbf_1var1
Cbrennerirbf_1var1
Cbriggsaerbf_1var1
Cremaneirbf_1var1
Cjaponicarbf_1var1
500
600

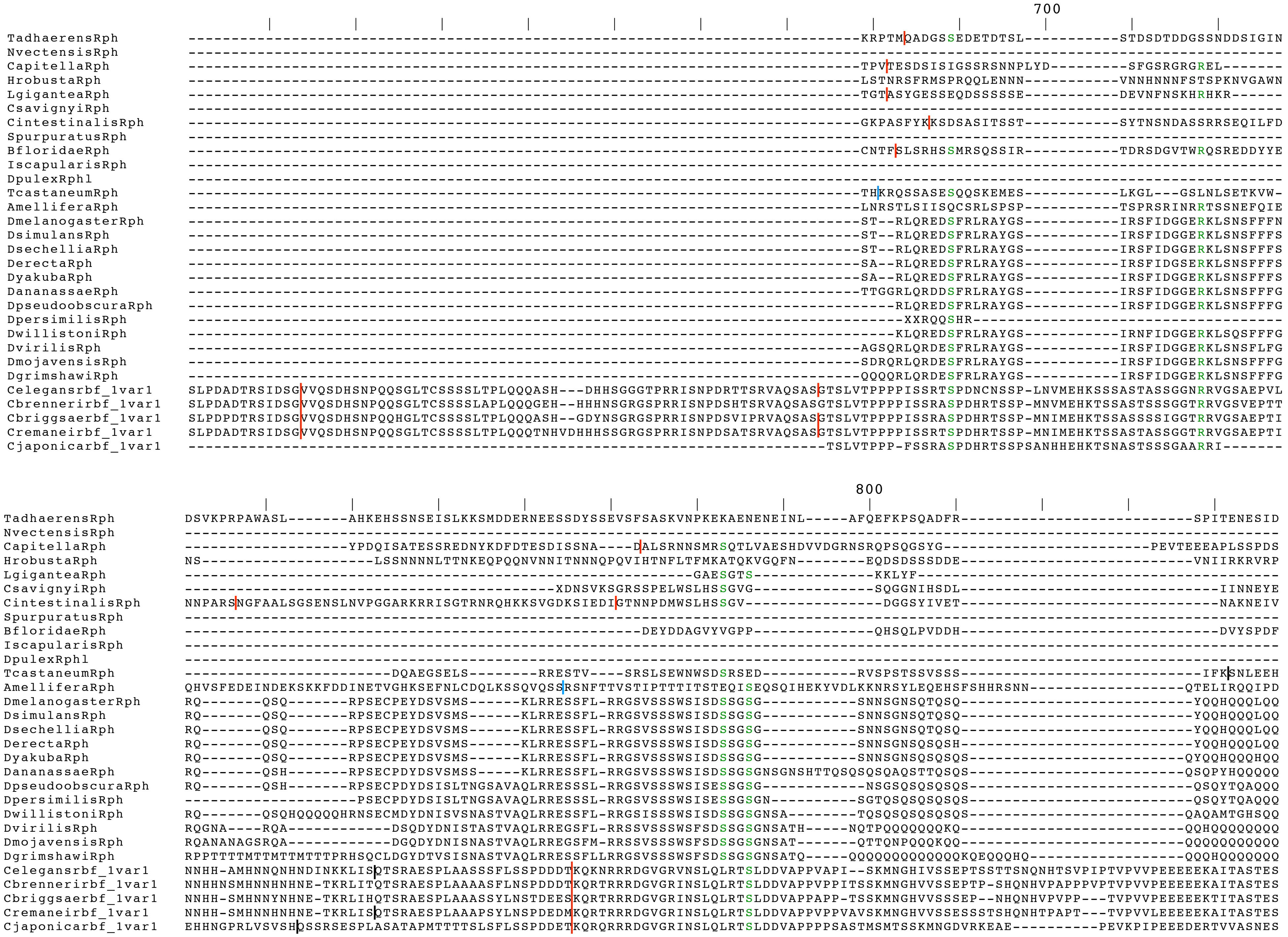

700
TadhaerensRph
NvectensisRph
CapitellaRph
HrobustaRph
LgiganteaRph
CsavignyiRph
CintestinalisRph
SpurpuratusRph
BfloridaeRph
IscapularisRph
DpulexRphl
TcastaneumRph
AmelliferaRph
DmelanogasterRph
DsimulansRph
DsechelliaRph
DerectaRph
DyakubaRph
DananassaeRph
DpseudoobscuraRph
DpersimilisRph
DwillistoniRph
DvirilisRph
DmojavensisRph
DgrimshawiRph
Celegansrbf_1var1
Cbrennerirbf_1var1
Cbriggsaerbf_1var1
Cremaneirbf_1var1
Cjaponicarbf_1var1
800
TadhaerensRph
NvectensisRph
CapitellaRph
HrobustaRph
LgiganteaRph
CsavignyiRph
CintestinalisRph
SpurpuratusRph
BfloridaeRph
IscapularisRph
DpulexRphl
TcastaneumRph
AmelliferaRph
DmelanogasterRph
DsimulansRph
DsechelliaRph
DerectaRph
DyakubaRph
DananassaeRph
DpseudoobscuraRph
DpersimilisRph
DwillistoniRph
DvirilisRph
DmojavensisRph
DgrimshawiRph
Celegansrbf_1var1
Cbrennerirbf_1var1
Cbriggsaerbf_1var1
Cremaneirbf_1var1
Cjaponicarbf_1var1

```
                                                                                                  900
TadhaerensRph         STGNEGLGSLDFSVLFEPIDFKLHVTIIKAKNLKGMGKHGV-DPYVKLQLLP---GSSKSNKMRTKQS-KTCNPEFNETLTYLGVTKDDI----ARKTLRVYVMNHDRFSSDTVIGIYNLKLKDIG-
NvectensisRph         -----XLGLLEFTLRYQKLDSRLDVVLHDAKGLKPMDHGGTSDPYVKLHLLP---GASKSNKLRSHTKYKTLNPVFNETLTYYGITEEEI----SSKTLRLQVFDEDTIGRNDFIGETSINLKLLNS
CapitellaRph          DSGT-SLGTLEFSLLYDSSNNALHCTISRARGLRAMDKNGFSDPYCKLHLLP---GASKSFKLRTKTLPKTLNPDWNETLTYYGITEDDC----RRKTLRLSILDEDTFGSD-FIGETRVPLKLLKT
HrobustaRph           KESATTHGIIEFNLHYINKEKLLKCTIVRAKNLPAMNKDGTCDAFVKLNMLP---GLSKATKRRTKTKIKTRNPEFDETVEYHGVEEEDL----QRKMLRITVFDETRLSSK-FIGETMIMLKSLVT
LgiganteaRph          ---SASLGCLEFSLLYDSVNNALYCTIVRAKGLKAMDSNGLSDPYVKLHLLP---GANKSTKLRTKTIHKTLNPEWNETLTYYGITEEDV----VKKTLRLSVLDEDHFGF-DFIGETRVPLKKLKP
CsavignyiRph          TDNAAGLGTLEFTLLYDSHKLALYVTVIRGQGLKAMDINGFSDPYVKLHLFP---GSKKSTKMRTKTKQKTLNPIFDESLTYWGVTEADL----QRKTLRLTVLDEDRFGQNEKIGEVRVLLKNFNL
CintestinalisRph      TENTAGLGSLEFTLLHDSHKLALYVTVIRARGLKAMDINGFSDPYVKLHLLP---GSKKSTKMRTKTKQKTLNPTFDETLTYWGVTDADI----QKKTLRLTVLDEDRLGDNEFIGEVRIQLKNFNL
SpurpuratusRph        ----------------------------------------------------------XSTKLRTKTVAKTLNPDFNETLTYYGVTEDDL----SRKILRLSVLDEDRFGHNDFIGEYRLPLRKLTP
BfloridaeRph          EEEDTTLGSIEFSMLYDPHNAALHVNIIRARGIKPMDHNGMSDPYVKLHLLP---GASKANKLRTKTSYKTLNPIFNETLTYYGMRDDDI----LRKTLRLTVFDEDRFGHNEFIGETRVQLKRLKP
IscapularisRph        -------------------------------------------------------------------------------------------------------------------------------
DpulexRphl            -------------------------------------------------------------------------------------------------------------------------------
TcastaneumRph         KPSDHSLGTIELSLTYDPITSTLHCTVYRAKNLIPMDINGLSDPFCKLNILP---NAKPSTRLRTKTVHKTRNPEFNENLTFYDISESDL----SKKALHVLVVDDDKFGHD-YMGETRIGLAKLKQ
AmelliferaRph         HETRQNYGTLEISLRYDPIIQCLQCKVERARGLQPMDIYDLADPFCKLNIL-PVNSIAITKRLRTKTIHKTRDPEFNETLNFYGTTETDVRKIWNGKALHILILQDDPAGQD-FLGEARFLLRELQP
DmelanogasterRph      HCRDPLLGWLEIAISYREAFHSLDCTMVRARDLPAMDAAGLADPYCKLNIITPEAHTKYTRWQRTKTVHKTRNPEFNETLQFVGVEPEEL----GNSLIYVALFDDDKYGHD-FLGAAKVCLSTVHS
DsimulansRph          HCRDPLLGWLEIAISYREAFHSLDCTMMRARDLPAMDAAGLADPYCKLNIITPEAHTKYTRWQRTKTVHKTRNPEFNETLQFVGVEPEEL----GNSLLYVALFDDDKYGHD-FLGAAKVCLSTVHS
DsechelliaRph         HCRDPLLGWLEIAISYREAFHSLDCTMVRARDLPAMDAAGLADPYCKLNIITPEAHTKYTRWQRTKTVHKTRNPEFNETLQFVGVEPEEL----GNSLIYVALFDDDKYGHD-FLGAAKVCLSTVHS
DerectaRph            HCRDPLLGWLEIAISYREAFHSLDCTMVRARDLPAMDAAGLTDPYCKLNIITPEAHTKYTRWQRTKTVHKTRNPEFNETLQFVGVEPEEL----GNSLIYVALFDDDKYGHD-FLGAAKVCLSTVHS
DyakubaRph            HCRDPLLGWLEIAITYREAFHSLDCTMVRARDLPAMDAAGLTDPYCKLNIITPEAHTKYTRWQRTKTVHKTRNPEFNETLQFVGVEPEEL----GNSLIYVALFDDDKYGHD-FLGAAKVCLSTVHS
DananassaeRph         HCRDPLLGWLEIAISYREAFHSLDCTMVRARDLPAMDAAGLTDPYCKLNIITPEAHTKYTRWQRTKTVHKTRNPEFNETLQFVGVEPEEL----GNSLLYVALFDDDKYGHD-FLGAAKVCLSTVHS
DpseudoobscuraRph     HCRDPLLGWLEIAISYRENFHSLDCTMVRARDLPAMDASGLTDPYCKLNIITPEALTKYTRWQRTKTVHKTRNPEFNETLQFVGVEPEEL----GNSLLYVALFDDDKYGHD-FLGAAKVCLSTVHS
DpersimilisRph        HCRDPLLGWLEIAISYRENFHSLDCTMVRARDLPAMDASGLTDPYCKLNIITPEALTKYTRWQRTKTVHKTRNPEFNETLQFVGVEPEEL----GNSLLYVALFDDDKYGHD-FLGAAKVCLSTVHS
DwillistoniRph        HCRDPLLGWLEISISYRESFHSLDCTMVRARDLPAMDSAGLADPYCMLNIITPEAHAKYTRWQRTKTVHKTRNPEFNETLQFVGVEPEEL----GNSLLYVALFDDDKYGHD-FLGAAKVCLSTVHS
DvirilisRph           HCRDPLLGWLEIAISYRESFHSLDCTMVRARDLPAMDAAGLADPYCKLNIVTPEGHTKYTRWQRTKTVHKTRNPDFNETLQFVGVEPEEL----GNSLLYVALFDDDKYGHD-FLGAAKICLSTVHS
DmojavensisRph        HCRDPLLGWLELAISYRESFHSLDCTLVRARDLPAMDASGLADPYCKVNIVTPEGHTKYTRWQRTKTVHKTRNPDFNETLQFVGVEPEEL----GNSLLYVALFDDDKYGHD-FLGAAKVCLSTVHS
DgrimshawiRph         HCRDPLLGWLEIAISYRESYHSLDCTMVRARDLPAMDAAGLADPYCKLNIVTPEGHTKYTRWQRTKTVHKTRNPDFNETLQFVGVEPEEL----GNSLLYVALFDDDKYGHD-FLGAAKVCLSTVHS
Celegansrbf_1var1     ASEPGSLGSITLTLTYHSADKKLKMHLIRAKNLKAMDSNGFSDPYVKFHLLP---GNTKATKLTSKTIEKTLNPEWNEEMSYYGITEDDK----EKKILRVTVLDRDRIGSD-FLGETRIALKKLND
Cbrennerirbf_1var1    ASEPGSLGSVTLTLTYHSADKRLKIHLIRGKNLKAMDSNGFSDPYVKFHLLP---GNTKATKLTSKTIEKTLNPEWNEEMSYYGITEEDK----EKKILRVTVLDRDRIGSD-FLGETRIALKKLND
Cbriggsaerbf_1var1    ASEPGSLGSINLTLTYTSSDKKLRIHLIRAKNLKAMDSNGFSDPYVKFHLLP---GNTKATKLTSKTIEKTLNPEWNEEMAYYGITEEDK----EKKILRVTVLDRDRIGSD-FLGETRIALKKLND
Cremaneirbf_1var1     ASEPGSLGSISLTLTYHSSDKKLKIHLIRAKNLKAMDSNGFSDPYVKFHLLP---GNTKATKLTSKTIEKTLNPEWNEEMSYYGITEEDK----EKKILRVTVLDRDRIGSD-FLGETRIALKKLND
Cjaponicarbf_1var1    ASEPGSLGSITLSLTYHSADKKLKIHLIRSKNLKAMDSNGFSDPYVKFHLLP---GNTKATKLTSKTIEKTLNPEWNEEMSYYGITEEDK----EKKILRVTVLDRDRIGSD-FLGETRIALKKLQD

                                                1000
TadhaerensRph         DVMKRFNDVP-LHKI-----SQVLEIHVNSIER--------GRIEISLKYVTATRTLEVGIIQCIGLKAMDASGYSDPYVKCYLRPDKYKI----TKQK--------------------------TS
NvectensisRph         NPTQTFKRSL-IPKA-----LVIYRATLTSLPLQDSPHSPLGRIQLALKYNTQRGMLFVTVMRCSGLASMDANGYSDPYVKCYLKPDPQKK----SKRR--------------------------TN
CapitellaRph          EQTKNFSVYLERK-Q-------------IDEKQDENDPDERGRILLNLRFSSQKQKFTVRVVRCSALAAMDSNGYSDPYVKVYLKPDKGKK----SKYK--------------------------TT
HrobustaRph           DRTKDYS-VY-LSGL-----QEEEMRKVSDHDR--------GRILVGMMYNSSRNQLHIAIKRCANLVAMDSNGFSDPFVKLYLRPDKDKK----SKFK--------------------------TK
LgiganteaRph          HQTKAFNVYLEKTFP---------------LEKDDDLIAIRGKILLSLRYATSKQSLIVRIIRCAELASMDSNGYSDPYVKVYLKPDKDKK----SKYK--------------------------TS
CsavignyiRph          TQTMRFNMGLVMQKE----------------MKEDEDDSERGRIMITLSYQPRDSLLLVTINRCSSLLPMARTSSIDPQVKVCLRADGNSTESSFSKTE--------------------------RR
CintestinalisRph      TQTNTYNMGLTEHQD----------------IKEDEDSSERGRIMVTLSYQPRDTMLVATINRCSSLLPVARTNTIDPQVKLCLKSEANPSEFTFKKTE--------------------------RR
SpurpuratusRph        YQTKSLSVYLEKPLP---------------LEKDDELAGERGKLMVGLKYVSTRQCLVVSIIRGAGLAAMDSNGYSDPYVKVYLKPDAGKR----TKHK--------------------------TA
BfloridaeRph          NQTRQFNVFLEKMMPNLPFIVTVQIYYIEELEKDDDLMYERGRILLSLMYRSQRQQLVVGIMRCAHLAAMDPNGYSDPYVKVYLKPDYKKA----TKHK--------------------------TR
IscapularisRph        -------------------------------------------------------------------------------------------------------------------------------
DpulexRphl            -------------------------------------------------------------------------------------------------------------------------------
TcastaneumRph         QNTIYVAASLEEMRH-----EQKGPLPAPELNQWFDDLWSRGQILLSLCYSTKRRALLVMVCRCANLLPMDNNGFSDPFVKLQLRPDPC-H----RKHK--------------------------TS
AmelliferaRph         HQIKHYNVPL--QDH-----YPV----DNEEAAWGVFSSGRGQIQVSLSYCTRRRALIVTIHRAINLLPMDSNGFSDPFVKLCLIENVTNN----HRQRIFDYSIARITGKKLIPKKITSRNAQNTT
DmelanogasterRph      TSQYRISVPLGVEDQ-----YSN----AAEMAQ----NWPNGKMLLSLCYNTKRRALVVNVKQCINLMAMDNNGSSDPFVKIQLKPDAHKN----KKHK--------------------------TS
DsimulansRph          TSQYRISVPLGVEDQ-----YSN----AAEMAQ----NWPNGKMLLSLCYNTKRRALVVNVKQCINLMAMDNNSSSDPFVKIQLKPDAHKN----KKHK--------------------------TS
DsechelliaRph         TSQYRISVPLGVEDQ-----YSN----AAEMAQ----NWPNGKMLLSLCYNTKRRALVVNVKQCINLMAMDNNGSSDPFVKIQLKPDAHKN----KKHK--------------------------TS
DerectaRph            TSQYRISVPLGVEDQ-----YSN----AAEMAQ----NWPNGKMLLSLCYNTKRRALVVNVKQCINLMAMDNNGSSDPFVKIQLKPDAHKN----KKHK--------------------------TS
DyakubaRph            TSQYRISVPLGVEDQ-----YSN----AAELAQ----NWPNGKMLLSLCYNTKRRALVVNVKQCINLMAMDNNGSSDPFVKIQLKPDAHKN----KKHK--------------------------TS
DananassaeRph         TTQYRISVPLGAEDQ-----YSN----AAEMSQ----NWPNGKMLLSLCYNTKRRALVVNVKQCINLMPMDNNGSSDPFVKIQLKPDAHKN----KKHK--------------------------TS
DpseudoobscuraRph     TSQYRISVPLGVEDQ-----YSS----AAEMAQ----EWPNGKILISLCYNTKRRALVVNVKQCINLMAMDNNGSSDPFVKLQLKPDAHKN----KKHK--------------------------TS
DpersimilisRph        TSQYRISVPLGVEDQ-----YSS----AAEMAQ----EWPNGKILISLCYNTKRRALVVNVKQCINLMAMDNNGSSDPFVKLQLKPDAHKN----KKHK--------------------------TS
DwillistoniRph        TSQYRISVPLGAEDQ-----YSN----VAEMSQ----NWPNGKMLLSLCYNTKRRALVVDVKQCINLMAMDSNGSSDPFVKLQLKPDAHKN----KKHK--------------------------TS
DvirilisRph           TSQYRISVPLGVEDQ-----YST----EAELAQ----SWPNGKMLISLCYNTKRRALVVNVKQCINLLAMDNNGSSDPFVKLQLKPDVHKN----KKHK--------------------------TS
DmojavensisRph        TSQYRISVPLGAEDQ-----YSA----EAEMSQ----SWPNGKMLISLCYNTKRRALVVNVKQCINLLPMDNNGSSDPFVKLQLKPDAHKN----KKHK--------------------------TS
DgrimshawiRph         TTQYRISVPLGVEDQ-----YSN----EAELAQ----SWPNGKILISLCYNTKRRALVVNVKQCINLLAMDNNGSSHPFVKLQLRPDAHKN----KKHK--------------------------TS
Celegansrbf_1var1     NEMKKFNLYLESALP-------------VPQQTKEEENEDRGKINVGLQYNIQQGSLFININRCVELVGMDSTGFSDPYCKVSLTPITSKA----HRAK--------------------------TS
Cbrennerirbf_1var1    NEMKKFNLYLESALP-------------VPVQTKEEENEERGKINVGLQYNIQQGSLFININRCVELIGMDSTGFSDPYCKVSLTPITSKA----HRGK--------------------------TS
Cbriggsaerbf_1var1    NEMKKFNLYLESALP-------------VPVQTKEEESVERGKINVGLQYNIQQGSLFININRCVELIGMDSTGFSDPYCKVTMTPITSKA----HRGK--------------------------TT
Cremaneirbf_1var1     NEMKKFNLYLESALP-------------VPVQTKEEENEDRGKINVGLQYNIQQGSLFININRCVELVGMDSTGFSDPYCKVSLTPITSKA----HRGK--------------------------TS
Cjaponicarbf_1var1    NEMKRFNLYLESALP-------------VP---KEEETDERGKIHVGLQYNIQQGSLFINVNRCVELIGMDSTGFSDPYCKVSLTPITSKA----HRAK--------------------------TA
```

```
                        1100
TadhaerensRph           VKRRTLNPKFNA--VFKYKYAHSE-LAGKTLDIQVWDRDIGRKNDFIGGVYLGKD-SSGDQLRHWFQTLKTPNAKFTHFHTLTDELRHPAE---------------------------
NvectensisRph           IRKKTLNPEFNE--EFVYDIAHHE-LAKKSLEVTVWDYDVGKSNDFIGGVILNIN-AEGSALKHWYDMLKGPNQLHTQWHTLENVNAHDD----------------------------
CapitellaRph            TKKRTLNPEYNE--EFHYDISQPE-LDKKTLEITVWDKDIGKQNDYIGGVQLGNQ-AKGDRLRHWNEVLKIPDRNHEHWHPLSADGLPEVS---------------------------
HrobustaRph             VQSKTLNPEFNE--EFVYSVKTNE-LINKTLDVSVWDKDFAR-NEYIGGIMLGAE-SGAKEKSHWMEVINNNNQFFERWHVLKNIHHH------------------------------
LgiganteaRph            VKKRTLNPEFNE--EFVYEIKHNE-LPKKTLEVTVWDKDIGKANDFIGGVQIGIN-AKGERLRHWFNALKNPDREFRQWHILSAELTPEVYTSS------------------------
CsavignyiRph            KNTKSCNPVYNEVLRLPIPLHAKE-VVNCSLDVSVWDKDTFGKEHLIGAICFGIH-SKGDKLKHWFNCVKKPLDAHEMWHALAVPGD-DKMASLAESARRHRTPRLHRKGKNKEFFGK
CintestinalisRph        KNTKTSNPAFNETMKLPLPAQARE-LVNCSLDVSVWDKDTFGKEHLIGAVCFGIH-SKGDKLKQWFNCVKKPQEAHEMWHSLTLPNDPEKISSLAEAGRRSRTPRFHRKLHS------
SpurpuratusRph          VKKRTLNPEFNE--EFYYEVKHPE-LAKKTLEITVWDKDIAKANDYIGGVQLGIT-SKGERLRHWFETLKGIDKKYERWHTLSDESFGDE----------------------------
BfloridaeRph            MLKKTLNPEFNE--EFVYDVKLNE-LAKKTLEISVWDYDYGKPNDFIGAVQLGIQ-SKGERLKHWFDCLKYPDRRHERWHVLIEEFDPITTPTDTVRD--------------------
IscapularisRph          -------------XEFAFELKENDNLEKKFLQISVWDKDQGRVDEYLGGLELGLH-CKAAQLQHWLDVIRVPEQSFECWHALVPVPLLP-----------------------------
DpulexRphl              ----------------------------------------------------------------------------------------------------------------------
TcastaneumRph           IKWKNLNPVYNE--EFAFETRPTE-LATQSLYITVYDKDYGKSNDYLGGLILGGTGSKGLRLKQWLDMIRYPDHRHEAWHNLTEEILD------------------------------
AmelliferaRph           IKWKTLNPEWNE--EFTFTARLTD-LMKLTLYLTIWDKDFGKNNDYLGGLELSRN-SKGARLRHWIDVIKFPDHRHQAWHNLIDTILPIE----------------------------
DmelanogasterRph        VKWRTLNPIYNE--EFYFEASPHD-LNKEMLILTVWDKDLGKSNDFLGSLQLGAQ-SKGERLQQWLDCIRLPDHFHEKWHCLAPDNPAH-----------------------------
DsimulansRph            VKWRTLNPIYNE--EFYFEASPHD-LNKEMLILTVWDKDLGKSNDFLGSLQLGAQ-SKGERLQQWLDCIRLPDHFHEKWHCLAPDNPAH-----------------------------
DsecheliaRph            VKWRTLNPIYNE--EFYFEASPHD-LNKEMLILTVWDKDLGKSNDFLGSLQLGAQ-SKGERLQQWLDCIRLPDHFHEKWHCLAPDNPAH-----------------------------
DerectaRph              VKWRTLNPIYNE--EFYFEASPHD-LNKEMLILTVWDKDLGKSNDFLGSLQLGAQ-SKGERLQQWLDCIRLPDHFHEKWHCLAPDNPAH-----------------------------
DyakubaRph              VKWRTLNPIYNE--EFYFEASPHD-LNKEMLILTVWDKDLGKSNDFLGSLQLGAQ-SKGERLQQWLDCIRLPDHFHEKWHCLAPDNPAH-----------------------------
DananassaeRph           VKWRTLNPIYNE--EFYFEASPHD-LNKEMLILTVWDKDLGKSNDFLGSLQLGAQ-SKGDRLQQWLDCIRLPDHFHEKWHCLAPDNPAH-----------------------------
DpseudoobscuraRph       VKWRTLNPIYNE--EFYFEASPHD-LNKEMLIVTVWDKDLGKSNDFLGSLQLGAQ-SKGERLQQWHDCIRLPDHFHEKWHCLAPDNPAH-----------------------------
DpersimilisRph          VKWRTLNPIYNE--EFYFEASPHD-LNKEMLIVTVWDKDLGKSNDFLGSLQLGAQ-SKGERLQQWHDCIRLPDHFHEKWHCLAPDNPAH-----------------------------
DwillistoniRph          VKWRTLNPIYNE--EFYFEASPHD-LNKEMLILTVWDKDLGKSNDFLGSLQLGAQ-SKGERLQQWLDCIRLPDHFHEKWHCLAADNPTH-----------------------------
DvirilisRph             VKWRTLNPIYNE--EFYFEASPHD-LNKEMLILTVWDKDLGKSNDFLGSLQLGAQ-SKGDRLQQWLDCIRLPDHFHEKWHCLAPDNPTH-----------------------------
DmojavensisRph          VKWRTLNPVFNE--EFYFEASPHD-LNKEMLILTVWDKDLGKSNDFLGSLQLGAQ-SKGDRLQQWLDCIRLPDHFHEKWHCLASDNPTH-----------------------------
DgrimshawiRph           VKWRTLNPIYNE--EFYFEASPHD-LNKEMLILTVWDKDLGKSNDFLGSLQLGAQ-SKGDRLQQWLDCIRLPDHFHEKWHCLAPENPTH-----------------------------
Celegansrbf_1var1       TKKRTLNPEWNE--QLQFVVPFKD-LPKKTLQIGVYDHDLGKHDDYIGGILLSTS-AKDERGRQWIKCIENPGTLVEAWHRLELDS--------------------------------
Cbrennerirbf_1var1      IKKRTLNPEWNE--QLQFVVPFKD-LPKKTLQIGVYDHDLGKNDDYIGGILLSTS-AKDERGRQWIKCIENPGTLVEAWHRLELDS--------------------------------
Cbriggsaerbf_1var1      TKKRTLNPEWNE--QLQFVVPFKD-LPKKTLRIGVYDHDLGKHDDYIGGILLSTS-AKEERGRQWIKCIENPGTLVEAWHRLELDS--------------------------------
Cremaneirbf_1var1       TKKRTLNPEWNE--QLQFVVPFKD-LPKKTLQIGVYDHDLGKHDDYIGGILLSTS-AKDERGRQWIKCIENPGTLVEAWHRLELDS--------------------------------
Cjaponicarbf_1var1      TKKRTLNPEWNE--HIQFVVPFKD-LPKKTLQIGVFDHDLGKHDDYIGGILLSTS-AKDERGRQWIKCIENPGTLVEAWHRLELDS--------------------------------
```
