# Supplementary material for: A manual collection of Syt, Esyt, Rph3a, Rph3al, Doc2, and Dblc2 genes from 46 metazoan genomes - an open access resource for neuroscience and evolutionary biology
Source: BMC Genomics. 2010 Jan 15;11:37. doi: 10.1186/1471-2164-11-37 (PMC2823689; doi:10.1186/1471-2164-11-37)
Supplement: Additional file 48 — Alignment of the vertebrate Rabphilin (Rph3a) sequences. Amino acid position is marked every hundred amino acids approximately, at the top of each page of the alignment. Splice variants are included and highlighted with black dots where they differ. Intron position and phase is indicated with a coloured bar between amino acids. Black bars indicate phase 0 introns. Red bars indicate phase +1 introns. Blue bars indicate phase +2 introns. The five conserved acidic amino acids in each C2 domain are indicated by black arrows at the top of the alignment. X residues indicate where a portion of sequence is missing. [file 1471-2164-11-37-S48.PDF]

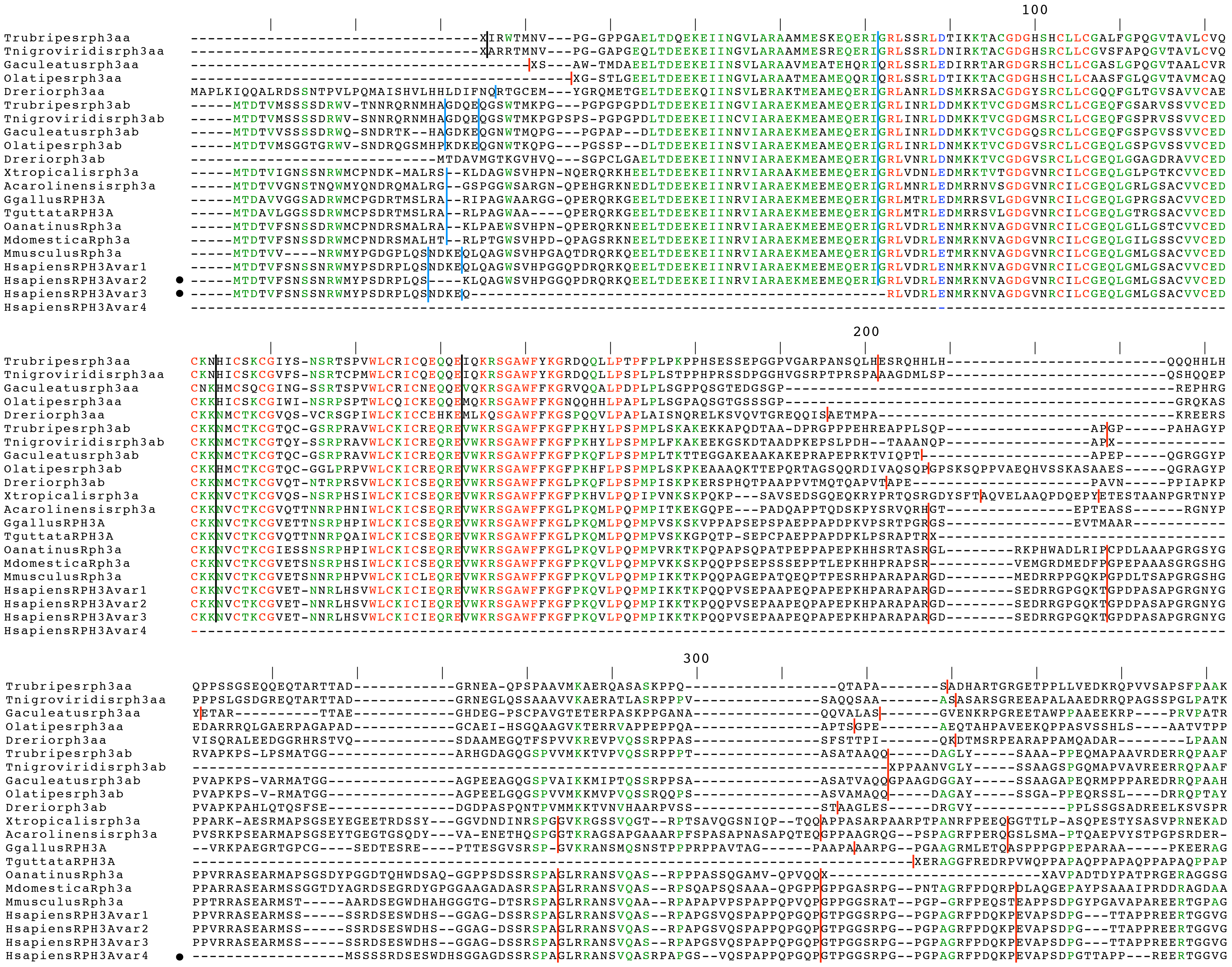
100
200
300
Trubripesrph3aa
Tnigroviridisrph3aa
Gaculeatusrph3aa
Olatipesrph3aa
Drreriorph3aa
Trubripesrph3ab
Tnigroviridisrph3ab
Gaculeatusrph3ab
Olatipesrph3ab
Drreriorph3ab
Xtropicalisrph3a
Acarolinensisrph3a
GgallusRPH3A
TguttataRPH3A
OanatinusRph3a
MdomesticaRph3a
MmusculusRph3a
HsapiensRPH3Avar1
HsapiensRPH3Avar2
HsapiensRPH3Avar3
HsapiensRPH3Avar4

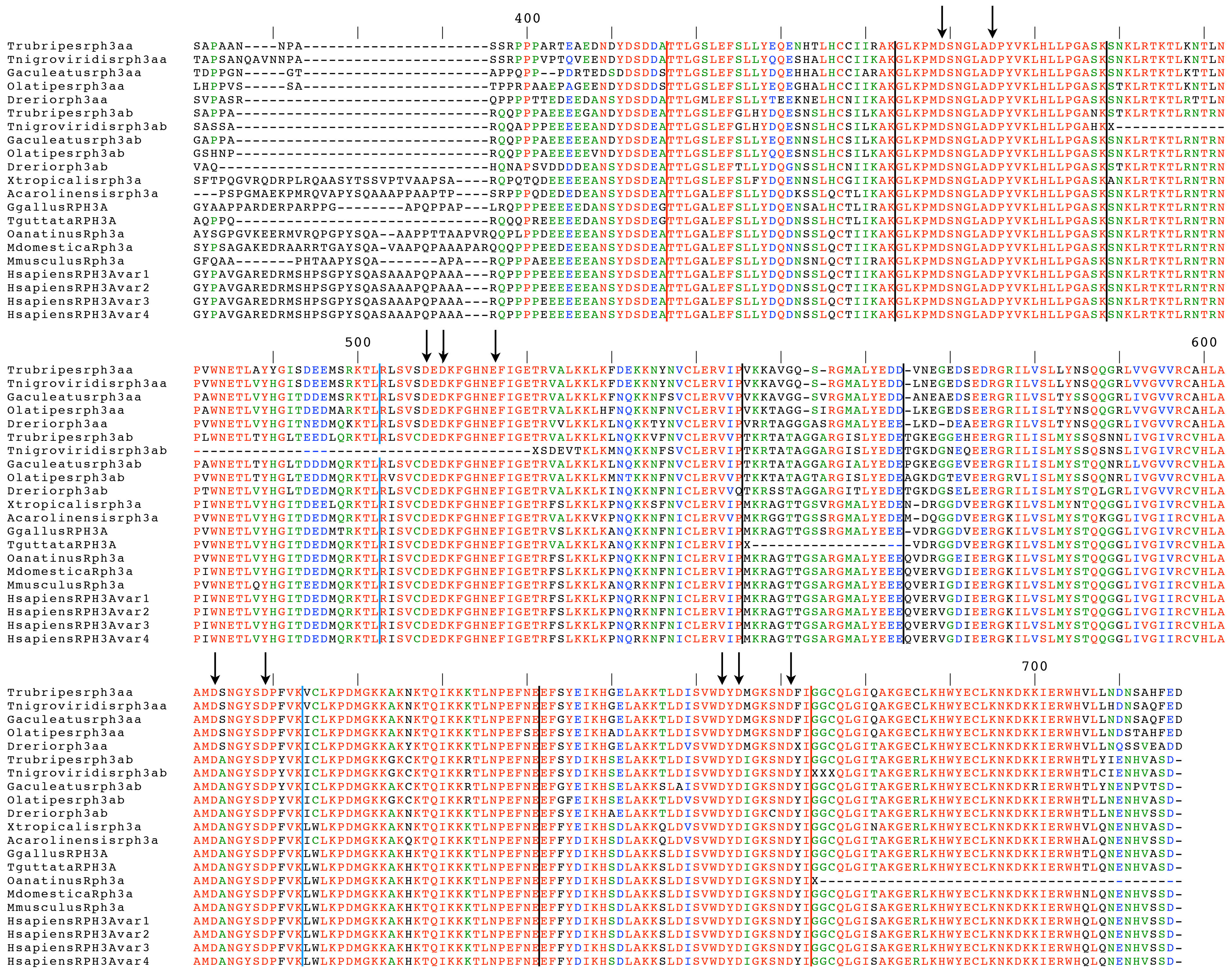

400
500
600
700
Trubripesrph3aa
Tnigroviridisrph3aa
Gaculeatusrph3aa
Olatipesrph3aa
Dreriorph3aa
Trubripesrph3ab
Tnigroviridisrph3ab
Gaculeatusrph3ab
Olatipesrph3ab
Dreriorph3ab
Xtropicalisrph3a
Acarolinensisrph3a
GgallusRPH3A
TguttataRPH3A
OanatinusRph3a
MdomesticaRph3a
MmusculusRph3a
HsapiensRPH3Avar1
HsapiensRPH3Avar2
HsapiensRPH3Avar3
HsapiensRPH3Avar4
