# Supplementary material for: A manual collection of Syt, Esyt, Rph3a, Rph3al, Doc2, and Dblc2 genes from 46 metazoan genomes - an open access resource for neuroscience and evolutionary biology
Source: BMC Genomics. 2010 Jan 15;11:37. doi: 10.1186/1471-2164-11-37 (PMC2823689; doi:10.1186/1471-2164-11-37)
Supplement: Additional file 49 — Alignment of the vertebrate Rph3al sequences. Amino acid position is marked every hundred amino acids approximately, at the top of each page of the alignment. Splice variants are included and highlighted with black dots where they differ. Intron position and phase is indicated with a coloured bar between amino acids. Black bars indicate phase 0 introns. Red bars indicate phase +1 introns. Blue bars indicate phase +2 introns. X residues indicate where a portion of sequence is missing. [file 1471-2164-11-37-S49.PDF]

```
                                                                                                                      100
Dreriorph3al                 MTDTMFGSENEQWVCPNDRQLMLRAKLHTGWSIHTFQSERQRKAQALEKRELDLIMSVIHRAEQLELIEQHRIGRLVERLDNMRSSAMGNGLSQCLLCGEVFGLLGSSSVLCL
Xtropicalisrph3alvar1        MTDTIFNNGSDQWVCPNDRQLALRAKLRAGWSVHTFQTEKQRKNQSLNQNEIDVILQVIRRAEKVDTLEQQRIGRLVERLENMRKNVMGNGMSQCLLCGELLGFLGTTSVFCQ
Xtropicalisrph3alvar2        MTDTIFNNGSDQWVCPNDRQLALRAKLRAGWSVHTFQTEKQRKNQSLNQNEIDVILQVIRRAEKVDTLEQQRIGRLVERLENMRKNVMGNGMSQCLLCGELLGFLGTTSVFCQ
Xtropicalisrph3alvar3        MTDTIFNNGSDQWVCPNDRQLALRAKLRAGWSVHTFQTEKQRKNQSLNQNEIDVILQVIRRAEKVDTLEQQRIGRLVERLENMRKNVMGNGMSQCLLCGELLGFLGTTSVFCQ
Acarolinensisrph3al          MADTIFGNGNDKWVCPNDRQLALRAKLQTGWSVHTFQTDRQRRDASLNSKEMEIILEVIHRAEKLDIVEQQRIGRLVERLENMQKNVMGNGLSQCLLCGEALGLLGSTAVFCQ
GgallusRPH3AL                MADTIFGSGTGPWVCPNDRQLALRAKLQTGWSVHTFQTEKQRKMQALSPQELEVILEVIRKAEKLDVVEQQRIGRLVERLENMRKNAMGNGLSQCLLCSELLGLLGSTSVFCQ
TguttataRPH3AL               MADMIFGSGTGPWVCPNDRQLALRAKLQTGWSVHTFQIEKQRKMQALSPKELEVILGVIRKAEHLDAVEQQRIGRLVERLENMRKNAMGNGLSQCLLCGEMLGLLGSSSVFCQ
OanatinusRph3al              MADTIFGNGSDQWVCPNDRQLALRAKLQSGWSVHTYQTDKQRKSQSLNPAELSTILEVIQRAEKLDVIEQHRIGGLVERLENMRKNVMGNGLSQCLLCGEPLGLLGGTAVFCR
MdomesticaRph3al             MADTIFGSGKDQWVCPNDRQLALRAKLQSGWSVHTYQTDKQRKSQSLNADEVEAILEVIQRAEKLDIMEQQRIGRLVERLENMRKNVMGNGLSQCFLCGEPLGLLGSSSVFCQ
MmusculusRph3alvar1          MADTIFSSGNDQWVCPNDRQLALRAKLQTGWSVHTYQTEKQRRSQCLSPGELEIILQVIQRAERLDILEQQRIGRLVERLETMQRNVMGNGLSQCLLCGEVLGFLGSSSVFCK
MmusculusRph3alvar2          MADTIFSSGNDQWVCPNDRQLALRAKLQTGWSVHTYQTEKQRRSQCLSPGELEIILQVIQRAERLDILEQQRIGRLVERLETMQRNVMGNGLSQCLLCGEVLGFLGSSSVFCK
HsapiensRPH3ALvar1           MADTIFGSGNDQWVCPNDRQLALRAKLQTGWSVHTYQTEKQRRKQHLSPAEVEAILQVIQRAERLDVLEQQRIGRLVERLETMRRNVMGNGLSQCLLCGEVLGFLGSSSVFCK
HsapiensRPH3ALvar2           MADTIFGSGNDQWVCPNDRQLALRAKLQTGWSVHTYQTEKQRRKQHLSPAEVEAILQVIQRAERLDVLEQQRIGRLVERLETMRRNVMGNGLSQCLLCGEVLGFLGSSSVFCK

                                                                                                             200
Dreriorph3al                 DCCMKVCTKCGIETTAGGQKRTQWLCKICSEQREVWKRSGAWFYKALPKHIRPIK--DSILDN----RKPVIERGEQLP-VARSAPISYTWAQSRVYLSESDGSDAELSECSS
Xtropicalisrph3alvar1        DCKKKVCTKCGIETV-GSQKRQVWLCKICSEQREVWKRSGAWFYKGLPKYILPMK--FSNKTGELHLRPLQPDHPTQEA-KNS-SNRTYTWAKGKVVSSDSE-SDSEHSSSSL
Xtropicalisrph3alvar2        DCKKKVCTKCGIETV-GSQKRQVWLCKICSEQREVWKRSGAWFYKGLPKYILPMK--FSNKTGELHLRPLQPDHPTQEA-KNS-SNRTYTWAKGKVVSSDSE-SDSEHSSSSL
Xtropicalisrph3alvar3        DCKKKVCTKCGIETV-GSQKRQVWLCKICSEQREVWKRSGAWFYKGLPKYILPMK--FSNKTGELHLRPLQPDHPTQEA-KNS-SNRTYTWAKGKVVSSDSE-SDSEHSSSSL
Acarolinensisrph3al          DCKKKVCTKCGIETL-NAQKRPVWFCKICSEHREVWKRSGAWFYKGLPKYILPLKNSSSSKTGELHLRPHHAEMPTLEA-KGASPSRTYTWARGKX-----------------
GgallusRPH3AL                DCKKKVCTKCGIETF-GAQKRPLWLCKICSEQREVWKRSGAWFYKGLPKYITPLK--SSSRAGEVPSQPWQSEAAVLEA-GSVGTGRSFTWARGKVVSSDSD-SE--LSSSSQ
TguttataRPH3AL               DCKKKVCTKCGIETV-GAQKRPLWLCKICSEQREVWKRSGAWFYKGLPKYLVPLK--SSIKALELQCQPRQDEEPEPESVRS---SHSFTWARGKX-----------------
OanatinusRph3al              DCKKKVCTKCGIETS-GGQKRPLWLCKICSEQREVWKRSGAWFYRGLPKYILPLK--TAGRTHEAHLRPLQVELPPYQTTKSPLASRSYTWARGKX-----------------
MdomesticaRph3al             DCKKKVCTKCGIETS-GSQKRTLWLCKICSEQREVWKRSGAWFYKALPKYILPLK--MAGPASEPHFRPPPLEPAASDP-RSIGTSRSYTWARGKVVSSDSD-SDSTLSSSSL
MmusculusRph3alvar1          DCRKKVCTKCGIEAS-PGQKRPLWLCKICSEQREVWKRSGAWFYKGLPKYILPLK--TPGRADDPHFRPLPVEPTETQP-PSAETSRVYTWARGRVVSSDSD-SDSDLSSSSL
MmusculusRph3alvar2        ● DCRKLGIRWLQHFLESLLILNRDLTCSL-------------------------------------------------------------------------------------
HsapiensRPH3ALvar1           DCRKKVCTKCGIEAS-PGQKRPLWLCKICSEQREVWKRSGAWFYKGLPKYILPLK--TPGRADDPHFRPLPTEPAEREP-RSSETSRIYTWARGRVVSSDSD-SDSDLSSSSL
HsapiensRPH3ALvar2         ● DCRK------------------------------VWKRSGAWFYKGLPKYILPLK--TPGRADDPHFRPLPTEPAEREP-RSSETSRIYTWARGRVVSSDSD-SDSDLSSSSL

                                                                                 300
Dreriorph3al                 DRKTFASETNQRRDSES---SGGQVQTPQSSFTSPTRGNVHIE-SPSSLVSEQSSSSSFNPGLEDEVIDHSRTLLSFCKLSMRRFIFLPSM--------------------
Xtropicalisrph3alvar1        DGKMV-----DPKGDKNRKHSGSSTEPSMTPVVSHMKNLEGLSGSQSSFGS-ERGSATESSQGDLIEGYNSDIPSCTTEEKITKYLKRRNAARLVNGSHFLSFVGNLTTTC
Xtropicalisrph3alvar2        DGKMV-----DPKGDKNRKHSGSSTEPSMTPVVSHMKNLEGLSGSQSSFGS-ERGSATESSQGDLIEGYNSDIPSCTTEEKITKYLKRRNAARLVNGKRRGHTNLWH----
Xtropicalisrph3alvar3        DGKMV-----DPKGDKNRKHSGSSTEPSMTPVVSHMKNLEGLSGSQSSFGS-ERGSATESSQGDLIEGYNSDIPSCTT------------------GKRRGHTNLWH----
Acarolinensisrph3al          ---------------------------------------------------------------------------------------------------------------
GgallusRPH3AL                EDRPSSAGSKGSPGGKQQAGQVLSGEPGR---------PMRVSGSRGSAGSGQGESCHSDQAADGHNSGGGRAP----------------------GKRGTHSTTRC----
TguttataRPH3AL               ---------------------------------------------------------------------------------------------------------------
OanatinusRph3al              ---------------------------------------------------------------------------------------------------------------
MdomesticaRph3al             EEKLLSSGTKSPKGSKSRTELGGSSDLPRA-AQGQALPPSHPSGSWTSLGSSEAGGVQSGPESGPG----ELQG----------------------GKRHTWMSPRF----
MmusculusRph3alvar1          EDRPLPSGVKGTKGDKPRGDSGASMESPR---LGPARPPSHLSGSQSSLGS-EAGTGATEPQGGTPAQPEPRVP----------------------GKRHTWATPRY----
MmusculusRph3alvar2          ---------------------------------------------------------------------------------------------------------------
HsapiensRPH3ALvar1           EDRLPSTGVRDRKGDKPWKESGGSVEAPR---MGFTHPPGHLSGCQSSLASGETGTGSADPPGGPRPGLTRRAPVKDTPGRAPAADAAPAGPSSCLG--------------
HsapiensRPH3ALvar2           EDRLPSTGVRDRKGDKPWKESGGSVEAPR---MGFTHPPGHLSGCQSSLASGETGTGSADPPGGPRPGLTRRAPVKDTPGRAPAADAAPAGPSSCLG--------------
```
