# Supplementary material for: A manual collection of Syt, Esyt, Rph3a, Rph3al, Doc2, and Dblc2 genes from 46 metazoan genomes - an open access resource for neuroscience and evolutionary biology
Source: BMC Genomics. 2010 Jan 15;11:37. doi: 10.1186/1471-2164-11-37 (PMC2823689; doi:10.1186/1471-2164-11-37)
Supplement: Additional file 50 — Alignment of the vertebrate Doc2 sequences. Amino acid position is marked every hundred amino acids approximately, at the top of each page of the alignment. Splice variants are included and highlighted with black dots where they differ. Intron position and phase is indicated with a coloured bar between amino acids. Black bars indicate phase 0 introns. Red bars indicate phase +1 introns. Blue bars indicate phase +2 introns. Some intron positions are marked with dotted lines. In these cases, transcript sequence covers a gap in the genomic sequence and the intron presence is assumed. The five conserved acidic amino acids in each C2 domain are indicated by black arrows at the top of the alignment. X residues indicate where a portion of sequence is missing. [file 1471-2164-11-37-S50.PDF]

```
                                                                                                                        100
                               |         |         |         |         |         |         |         |         |         |         |         |         |
Trubripesdoc2a          ---------------------------------------------MTVRK-GKSLAITIQEHMAIDVCPGPIRPIRQISAYFPRLSPTSSFSEPLSPNQVAAPTALSPGSVGHSGGAIGGGGSGGGGSSSLL
Tnigroviridisdoc2a      ---------------------------------------------MTVRK-GKSLAITIQEHMAIDVCPGPIRPIRQISAYFPRLSPTSSFSEPLSPNQVAAPTTLSPGGAGHSGGAAGGGGSGGGGSGGLL
Gaculeatusdoc2aa        ---------------------------------------------MTVRK-GKSLAISIQEHMAIDVCPGPIRPIRQISAYFPRLSPTSSFSEQLSPNQAAAPTSLSPGAAA-------------GRSGGLL
Gaculeatusdoc2ab        --------------------------------------------MMALRG-GKKLSISIQEHMAIDVCPGPIRPIRQISAYFPRLSPT-------SPGPTSSPLALSPGVGG----------------AHLL
Olatipesdoc2aa          ---------------------------------------------MTVRQ-GNRLGISIQEHMAINVCPGPIRPIRQISSYFPRLSPTSSFSEPLSPNQLTAPAPLSPGGSGQDGGAVGGGGEGGG-SRSFL
Olatipesdoc2ab          ---------------------------------------------MTVQR-GKKLSISIQEHMAIDVCPGPIRPIRQISAYFPRLSPTSPTPLSPNPSLSPPPNTSPVALSPPV---------ASAMGGAHL
Dreriodoc2a             ---------------------------------------------MTVRK-GKKLTISIQEHMAIDVCPGPIRPIRQISAYFPRLSPTA---EPVSPNP-ASPLSLSPGLGA-----------SGGASGGLL
Xtropicalisdoc2a        ------------------------------------------------------------------------------------------------------------------------------------
Acarolinensisdoc2a      ---------------------------------------------MTVRR-GDRAPVPIREHMAIDVSPGPIRPIAQISAYFPHPGL--------EGVLSTRD-----------------------------
OanatinusDoc2a          ---------------------------------------------MTLRK-GEKMTISIQEHMAIDVCPGPIKPIKQISDYFPRFPRGLPAPVPRGHCPRPPAAQGSGSPSD--------------PPREDD
MdomesticaDoc2a         ---------------------------------------------MRGRR-GDRMTINIQEHMAINVCPGPIRPIRQISDYFPRLGP--------EGGGSGADPQAQLSPGA-------------GAAATPS
MmusculusDoc2a          ---------------------------------------------MRGRR-GDRMTINIQEHMAINVCPGPIRPIRQISDYFPRRGP--------GPEGGG------GGGT--------------GCGEAPA
HsapiensDOC2Avar1       ---------------------------------------------MRGRR-GDRMTINIQEHMAINVCPGPIRPIRQISDYFPR-GP--------GPEGGG------SGG------------------EAPA
HsapiensDOC2Avar2       ------------------------------------------------------------------------------------------------------------------------------------
HsapiensDOC2Avar3     ● -----------MCAHECVPVRVPLRECARASTRVCARSRALPSGPRVGVR-DPAPSPAPAPLPVPRRRAGCILASTSLTPSSPHFLG--------APSLSARTARLRALLAG-------------RRRPACA
HsapiensDOC2Avar4       ------------------------------------------------------------------------------------------------------------------------------------

Trubripesdoc2b          ---------------------------------------------MTQRK-GEKPTISIQEHMAIDVCPGPIQPIKQISDYFPRYPRGLPPAVPQQAGRTGSLCSALSSSSAAS------ESDRSNEDKPDR
Tnigroviridisdoc2b      ---------------------------------------------MTQRK-GEKPTISIQEHMAIDVCPGPIQPIKQISDYFPRYPRGLPPALAHPAGRTGSLCSALSSSPAAS------ESERPNEDKPER
Gaculeatusdoc2b         ---------------------------------------------MTQRK-GEKPTISIQEHMAIDVCPGPIQPIKQISDYFPRYPRGLPPAAPQPAGHAGPPSPALSTSTSTSAASSTAESDRTNEDKPER
Olatipesdoc2b           ---------------------------------------------MTQRK-GEKPTISIQEHMAIDVCPGPIQPIKQISDYFPRYPRGLPAAAPH-VGPPRSPLSTPSTSVSAA------ESERPDEDSPER
Dreriodoc2b             ---------------------------------------------MTQRK-GEKTTISIQEHMAIDVCPGPIQPIKQISDYFPRYPRGLPPTAPPALSRAGSLRSSSTSQKSEGPQ---ADANRDEDDLDQA
Xtropicalisdoc2b        ---------------------------------------------MTLRK-GEKMTISIQEHMAIDVCPGPIKPIKQISDYFPRFPRGIPAAVTRA-----N--SLKSNVSQPSVSPCETSK-EEEEDVDKL
Acarolinensisdoc2b      ---------------------------------------------MTLRK-GEKTTISIQEHMAIDVCPGPIKPIKQISDYFPRFPRGLPATVSRSNSLRSSSSQAAVSPSETAPR-------EDDEDVDQL
GgallusDOC2B            ---------------------------------------------MTLRK-GEKTTISIQEHMAIDVCPGPIKPIKQISDYFPRFPRGLPAAVGRS-----G--ALRPAVSQPSVGPTETPR-EDDEDVDQL
TguttataDOC2Bvar1       ---------------------------------------------MTLRK-GEKMTISIQEHMAIDVCPGPIKPIKQISDYFPRFPRGLPAAGGRS-----SGSAPRPTPRQPTGDPAEHPRHEEEEDVDQL
TguttataDOC2Bvar2       ---------------------------------------------MTLRK-GEKMTISIQEHMAIDVCPGPIKPIKQISDYFPRFPRGLPAAGGRS-----SGSAPRPTPRQPTGDPAEHPRHEEEEDVDQL
OanatinusDoc2b          ---------------------------------------------MTLRK-GEKMTISIQEHMAIDVCPGPIKPIKQISDYFPRFPRGLPAPVPR-------GHCPRPPAAQGSGSPSDPPR-EDDEDVDQL
MdomesticaDoc2b         ---------------------------------------------MTLRR-GEKTTISIQEHMAIDVCPGPIKPIKQISDYFPRFPRGLPTSVSR-------AGSLQAGGSQASVSPSDPPR-DDEEDVDQL
MmusculusDoc2b          ---------------------------------------------MTLRRRGEKATISIQEHMAIDVCPGPIRPIKQISDYFPRFPRGLPPTAAPRAPAPPDAPARSPAASASPRSPSDGAR-DDDEDVDQL
HsapiensDOC2B           ---------------------------------------------MTLRRRGEKATISIQEHMAIDVCPGPIRPIKQISDYFPRFPRGLPPDAGPRAAAPPDAPARPAVAGAGRRSPSDGAR-EDDEDVDQL

Trubripesdoc2d          MSVSKPPPPPS-------TSHLAIPPSSTSCPS-SSSSSPSATPHTTSMPPASPVKISMQEHFAINVCPGPILPIPQISDFFPRFHDYPCTPPPPREKKILKEETFNGETGG------------GYRDGERR
Tnigroviridisdoc2d      MSVSKPPPPPS-------TAHLAIPPSSTSSPS-PSASSPSATPHATSMPPASPVKISMQEHFAINVCPGPILPIPQISDFFPRFHDYPCTPPPPREKKILKEETFNGETGG------------GYRDGERR
Gaculeatusdoc2d         MSVSKPPPPPS-------TSHLAIPPSSTSAPS-SSATSPSATPHPAGPPPPSPVKISMQEHFAINVCPGPILPIPQISDFFPRFHDYPCTPPPPREKKILKEETFNGETGV------------GYKDGERR
Olatipesdoc2d           MSVSKPPPPPS-------TSHLTIPPPSASAPS-SSASSPLATPHSTAPPPPSPVKISMQEHFAINVCPGPILPIPQISDFFPRFHDYPCTPPPPREKKILKEETFNGETGG------------GHLDGERR
Dreriodoc2dvar1         MSVSKPPPPQSHFPPNNTTSHLPLPPSSSSTPSPSTPPSAAGPPSPAVPPPPSPVKISMQEHFAINVCPGPILPIPQISDFFPRFHDFPITPPPPREKQVLKDKDKDGDRD-------------GEKDRKRE
Dreriodoc2dvar2         ------------------------------------------------------------------------------------------------------------------------------------

MmusculusDoc2gvar1      --------------------------------------------MACAGPASGRQRVSMQEHMAIDVSPGPIRPIRLISNYFPHFYPFLEPVLRAPDRQAMLAPAIPSAPQ---------------------
MmusculusDoc2gvar2      ------------------------------------------------------------------------------------------------------------------------------------
Hsapiensdoc2g1          -------------------------------------------MAGTAAAGGQPPRVSMQEHMAIDVSPGPIRPIRLISHYFPHFYPFAEPALHPPNLRPAAASAVRSAPQ---------------------
Hsapiensdoc2g2          ------------------------------------------------------------------------------------------------------------------------------------
```

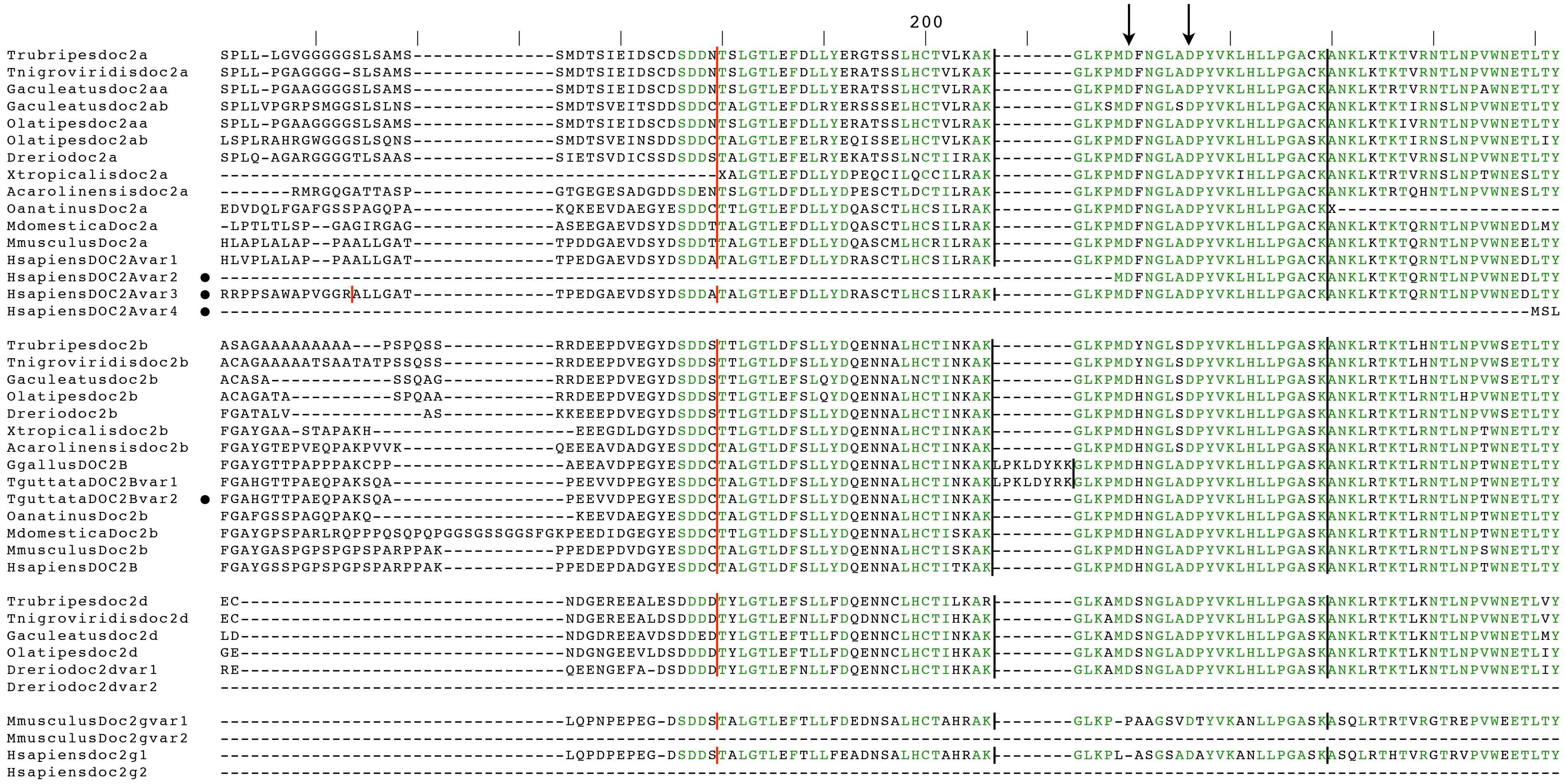
200
Trubripesdoc2a
Tnigroviridisdoc2a
Gaculeatusdoc2aa
Gaculeatusdoc2ab
Olatipesdoc2aa
Olatipesdoc2ab
Dreriodoc2a
Xtropicalisdoc2a
Acarolinensisdoc2a
OanatinusDoc2a
MdomesticaDoc2a
MmusculusDoc2a
HsapiensDOC2Avar1
HsapiensDOC2Avar2
HsapiensDOC2Avar3
HsapiensDOC2Avar4
Trubripesdoc2b
Tnigroviridisdoc2b
Gaculeatusdoc2b
Olatipesdoc2b
Dreriodoc2b
Xtropicalisdoc2b
Acarolinensisdoc2b
GgallusDOC2B
TguttataDOC2Bvar1
TguttataDOC2Bvar2
OanatinusDoc2b
MdomesticaDoc2b
MmusculusDoc2b
HsapiensDOC2B
Trubripesdoc2d
Tnigroviridisdoc2d
Gaculeatusdoc2d
Olatipesdoc2d
Dreriodoc2dvar1
Dreriodoc2dvar2
MmusculusDoc2gvar1
MmusculusDoc2gvar2
Hsapiensdoc2g1
Hsapiensdoc2g2

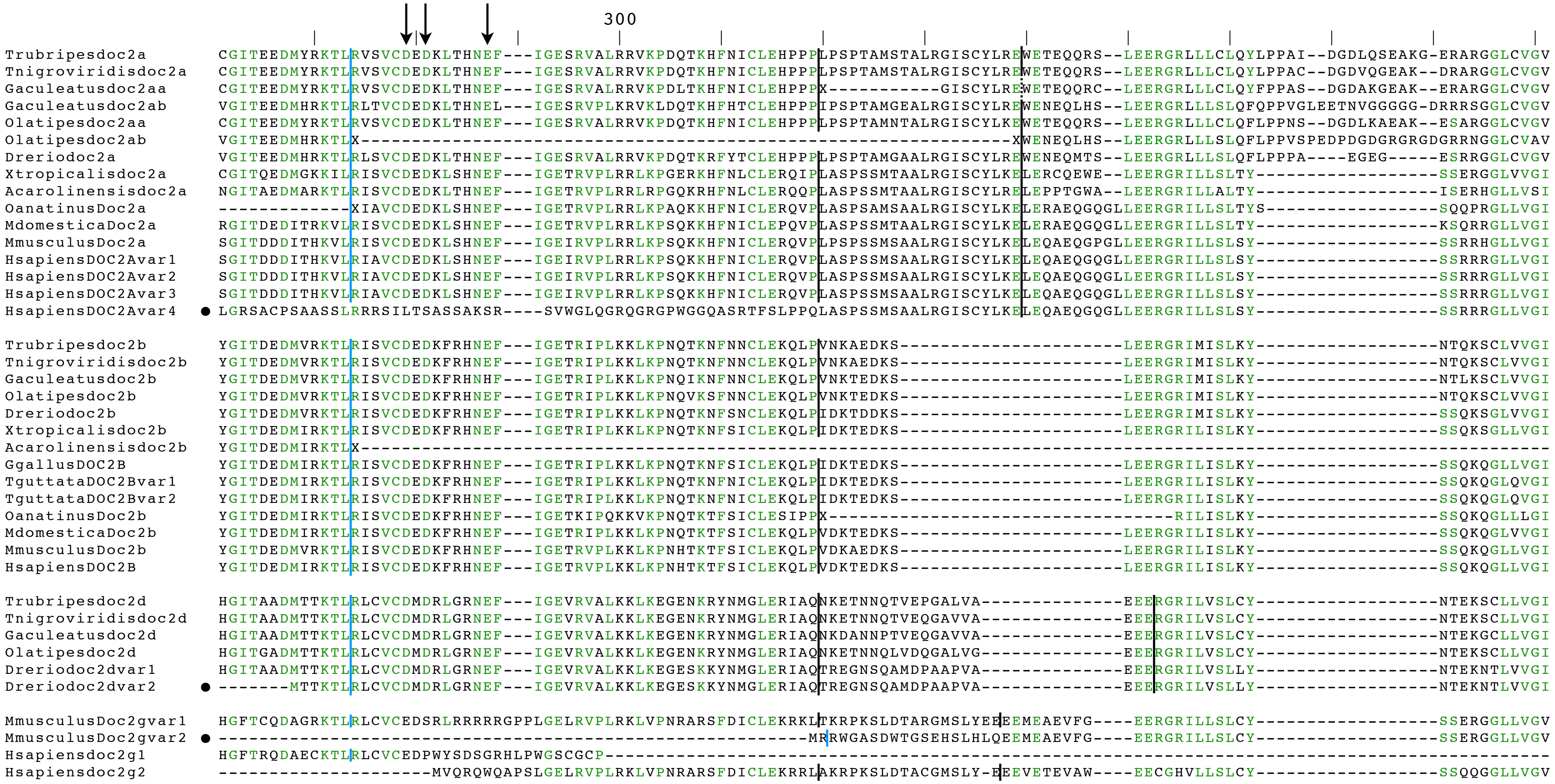

300
Trubripesdoc2a CGITEEDMYRKTLRVSVCDEDKLTHNEF---IGESRVALRRVKPDQTKHFNICLEHPPPLPSPTAMSTALRGISCYLREWETEQQRS--LEERGRLLLCLQYLPPAI--DGDLQSEAKG-ERARGGLCVGV
Tnigroviridisdoc2a CGITEEDMYRKTLRVSVCDEDKLTHNEF---IGESRVALRRVKPDQTKHFNICLEHPPPLPSPTAMSTALRGISCYLREWETEQQRS--LEERGRLLLCLQYLPPAC--DGDVQGEAK--DRARGGLCVGV
Gaculeatusdoc2aa CGITEEDMYRKTLRVSVCDEDKLTHNEF---IGESRVALRRVKPDLTKHFNICLEHPPPX-----------GISCYLREWETEQQRC--LEERGRLLLCLQYFPPAS--DGDAKGEAK--ERARGGLCVGV
Gaculeatusdoc2ab VGITEEDMHRKTLRLTVCDEDKLTHNEL---IGESRVPLKRVKLDQTKHFHTCLEHPPPIPSPTAMGEALRGISCYLREWENEQLHS--LEERGRLLLSLQFQPPVGLEETNVGGGGG-DRRRSGGLCVGV
Olatipesdoc2aa CGITEEDMYRKTLRVSVCDEDKLTHNEF---IGESRVALRRVKPDQTKHFNICLEHPPPLPSPTAMNTALRGISCYLKEWETEQQRS--LEERGRLLLCLQFLPPNS--DGDLKAEAK--ESARGGLCVGV
Olatipesdoc2ab VGITEEDMHRKTLX---XWENEQLHS--LEERGRLLLSLQFLPPVSPEDPDGDGRGRGDGRRNGGLCVAV
Dreriodoc2a VGITEEDMHRKTLRLSVCDEDKLTHNEF---IGESRVALRRVKPDQTKRFYTCLEHPPPLPSPTAMGAALRGISCYLREWENEQMTS--LEERGRLLLSLQFLPPPA----EGEG-----ESRRGGLCVGV
Xtropicalisdoc2a CGITQEDMGKKILRISVCDEDKLSHNEF---IGETRVPLRRLKPGERKHFNLCLERQIPLASPSSMTAALRGISCYLKELERCQEWE--LEERGRILLSLTY------------------SSERGGLVVGI
Acarolinensisdoc2a NGITAEDMARKTLRISVCDEDKLTHNEF---IGETRVPLRRLRPGQKRHFNLCLERQQPLASPSSMTAALRGISCYLRELEPPTGWA--LEERGRILLALTY------------------ISERHGLLVSI
OanatinusDoc2a -------------XIAVCDEDKLSHNEF---IGETRVPLRRLKPAQKKHFNICLERQVPLASPSAMSAALRGISCYLKELERAEQGQGLLEERGRILLSLTYS-----------------SQQPRGLLVGI
MdomesticaDoc2a RGITDEDITRKVLRISVCDEDKLSHNEF---IGETRVPLRRLKPSQKKHFNICLEPQVPLASPSSMTAALRGISCYLKELERAEQGQGLLEERGRILLSLTY------------------KSQRRGLLVGI
MmusculusDoc2a SGITDDDITHKVLRISVCDEDKLSHNEF---IGEIRVPLRRLKPSQKKHFNICLERQVPLPSPSSMSAALRGISCYLKELEQAEQGPGLLEERGRILLSLSY------------------SSRRHGLLVGI
HsapiensDOC2Avar1 SGITDDDITHKVLRIAVCDEDKLSHNEF---IGEIRVPLRRLKPSQKKHFNICLERQVPLASPSSMSAALRGISCYLKELEQAEQGQGLLEERGRILLSLSY------------------SSRRRGLLVGI
HsapiensDOC2Avar2 SGITDDDITHKVLRIAVCDEDKLSHNEF---IGEIRVPLRRLKPSQKKHFNICLERQVPLASPSSMSAALRGISCYLKELEQAEQGQGLLEERGRILLSLSY------------------SSRRRGLLVGI
HsapiensDOC2Avar3 SGITDDDITHKVLRIAVCDEDKLSHNEF---IGEIRVPLRRLKPSQKKHFNICLERQVPLASPSSMSAALRGISCYLKELEQAEQGQGLLEERGRILLSLSY------------------SSRRRGLLVGI
HsapiensDOC2Avar4 ● LGRSACPSAASSLRRRSILTSASSAKSR----SVWGLQGRQGRGPWGGQASRTFSLPPQLASPSSMSAALRGISCYLKELEQAEQGQGLLEERGRILLSLSY------------------SSRRRGLLVGI
Trubripesdoc2b YGITDEDMVRKTLRISVCDEDKFRHNEF---IGETRIPLKKLKPNQTKNFNNCLEKQLPVNKAEDKS----------------------LEERGRIMISLKY------------------NTQKSCLVVGI
Tnigroviridisdoc2b YGITDEDMVRKTLRISVCDEDKFRHNEF---IGETRIPLKKLKPNQTKNFNNCLEKQLPVNKAEDKS----------------------LEERGRIMISLKY------------------NTQKSCLVVGI
Gaculeatusdoc2b YGITDEDMVRKTLRISVCDEDKFRHNHF---IGETRIPLKKLKPNQIKNFNNCLEKQLPVNKTEDKS----------------------LEERGRIMISLKY------------------NTLKSCLVVGI
Olatipesdoc2b YGITDEDMVRKTLRISVCDEDKFRHNEF---IGETRIPLKKLKPNQVKSFNNCLEKQLPVNKTEDKS----------------------LEERGRIMISLKY------------------NTQKSCLVVGI
Dreriodoc2b YGITDEDMVRKTLRISVCDEDKFRHNEF---IGETRIPLKKLKPNQTKNFSNCLEKQLPIDKTDDKS----------------------LEERGRIMISLKY------------------SSQKSGLVVGI
Xtropicalisdoc2b YGITDEDMIRKTLRISVCDEDKFRHNEF---IGETRIPLKKLKPNQTKNFSICLEKQLPIDKTEDKS----------------------LEERGRILISLKY------------------SSQKSGLLVGI
Acarolinensisdoc2b YGITDEDMIRKTLX--
GgallusDOC2B YGITDEDMIRKTLRISVCDEDKFRHNEF---IGETRIPLKKLKPNQTKNFSICLEKQLPIDKTEDKS----------------------LEERGRILISLKY------------------SSQKQGLLVGI
TguttataDOC2Bvar1 YGITDEDMIRKTLRISVCDEDKFRHNEF---IGETRIPLKKLKPNQTKNFNICLEKQLPIDKTEDKS----------------------LEERGRILISLKY------------------SSQKQGLQVGI
TguttataDOC2Bvar2 YGITDEDMIRKTLRISVCDEDKFRHNEF---IGETRIPLKKLKPNQTKNFNICLEKQLPIDKTEDKS----------------------LEERGRILISLKY------------------SSQKQGLQVGI
OanatinusDoc2b YGITDEDMIRKTLRISVCDEDKFRHNEF---IGETKIPQKKVKPNQTKTFSICLESIPPX---------------------------------RILISLKY------------------SSQKQGLLLGI
MdomesticaDoc2b YGITDEDMIRKTLRISVCDEDKFRHNEF---IGETRIPLKKLKPNQTKTFSICLEKQLPVDKTEDKS----------------------LEERGRILISLKY------------------SSQKQGLVVGI
MmusculusDoc2b YGITDEDMVRKTLRISVCDEDKFRHNEF---IGETRVPLKKLKPNHTKTFSICLEKQLPVDKAEDKS----------------------LEERGRILISLKY------------------SSQKQGLLVGI
HsapiensDOC2B YGITDEDMIRKTLRISVCDEDKFRHNEF---IGETRVPLKKLKPNHTKTFSICLEKQLPVDKTEDKS----------------------LEERGRILISLKY------------------SSQKQGLLVGI
Trubripesdoc2d HGITAADMTTKTLRLCVCDMDRLGRNEF---IGEVRVALKKLKEGENKRYNMGLERIAQNKETNNQTVEPGALVA-------------EEERGRILVSLCY------------------NTEKSCLLVGI
Tnigroviridisdoc2d HGITAADMTTKTLRLCVCDMDRLGRNEF---IGEVRVALKKLKEGENKRYNMGLERIAQNKETNNQTVEQGAVVA-------------EEERGRILVSLCY------------------NTEKSCLLVGI
Gaculeatusdoc2d HGITAADMTTKTLRLCVCDMDRLGRNEF---IGEVRVALKKLKEGENKRYNMGLERIAQNKDANNPTVEQGAVVA-------------EEERGRILVSLCY------------------NTEKGCLLVGI
Olatipesdoc2d HGITGADMTTKTLRLCVCDMDRLGRNEF---IGEVRVALKKLKEGENKRYNMGLERIAQNKETNNQLVDQGALVG-------------EEERGRILVSLCY------------------NTEKSCLLVGI
Dreriodoc2dvar1 HGITAADMTTKTLRLCVCDMDRLGRNEF---IGEVRVALKKLKEGESKKYNMGLERIAQTREGNSQAMDPAAPVA-------------EEERGRILVSLLY------------------NTEKNTLVVGI
Dreriodoc2dvar2 ● -------MTTKTLRLCVCDMDRLGRNEF---IGEVRVALKKLKEGESKKYNMGLERIAQTREGNSQAMDPAAPVA-------------EEERGRILVSLLY------------------NTEKNTLVVGI
MmusculusDoc2gvar1 HGFTCQDAGRKTLRLCVCEDSRLRRRRRGPPLGELRVPLRKLVPNRARSFDICLEKRKLTKRPKSLDTARGMSLYEEEMEAEVFG----EERGRILLSLCY------------------SSERGGLLVGV
MmusculusDoc2gvar2 ● --MRRWGASDWTGSEHSLHLQEEMEAEVFG----EERGRILLSLCY------------------SSERGGLLVGV
Hsapiensdoc2g1 HGFTRQDAECKTLRLCVCEDPWYSDSGRHLPWGSCGCP--
Hsapiensdoc2g2 ---------------------MVQRQWQAPSLGELRVPLRKLVPNRARSFDICLEKRRLAKRPKSLDTACGMSLY-EEEVETEVAW----EECGHVLLSLCY------------------SSQQGGLLVGV

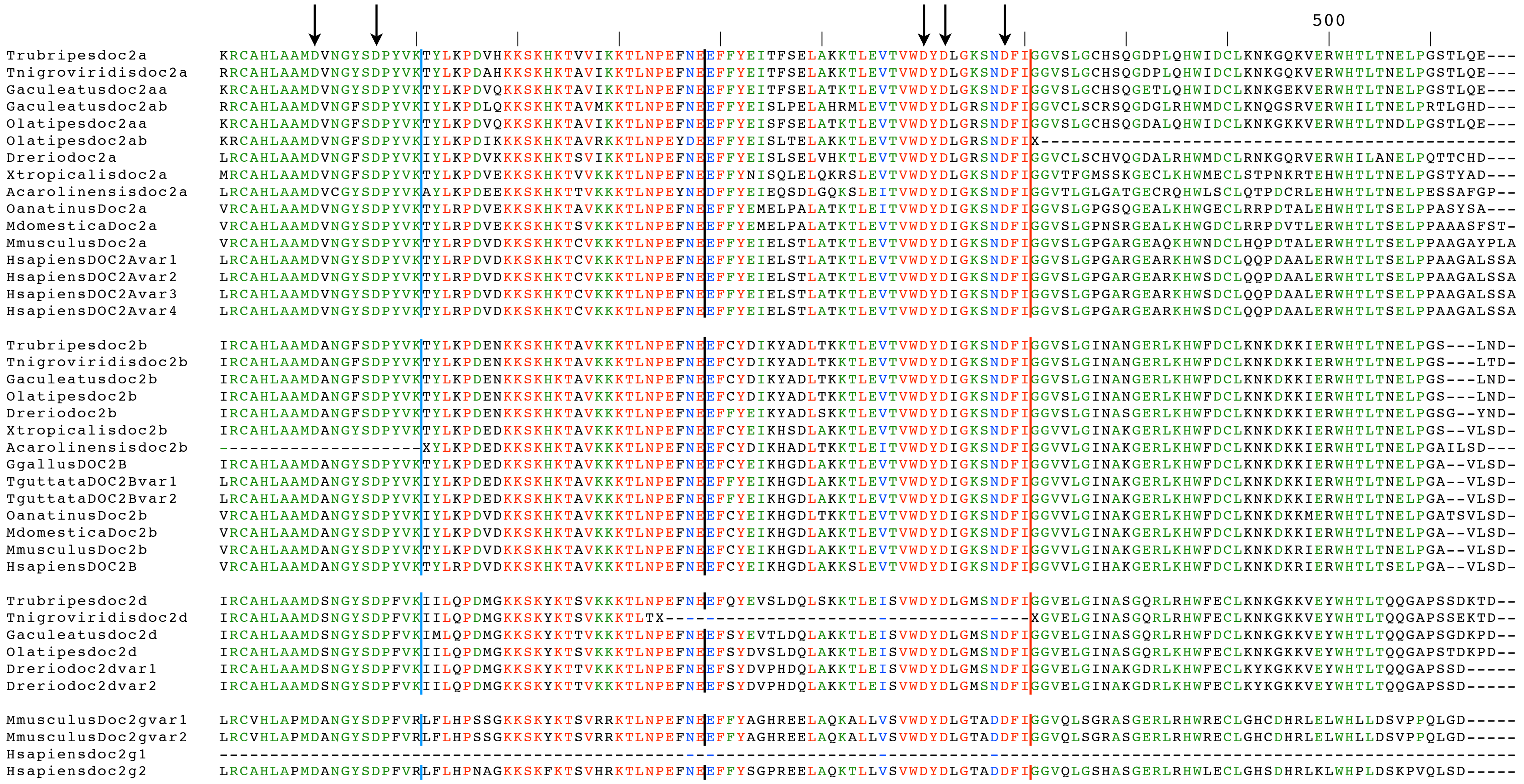

500
Trubripesdoc2a KRCAHLAAMDVNGYSDPYVKTYLKPDVHKKSKHKTVVIKKTLNPEFNEEFFYEITFSELAKKTLEVTVWDYDLGKSNDFIGGVSLGCHSQGDPLQHWIDCLKNKGQKVERWHTLTNELPGSTLQE---
Tnigroviridisdoc2a RRCAHLAAMDVNGYSDPYVKTYLKPDAHKKSKHKTAVIKKTLNPEFNEEFFYEITFSELAKKTLEVTVWDYDLGKSNDFIGGVSLGCHSQGDPLQHWIDCLKNKGQKVERWHTLTNELPGSTLQE---
Gaculeatusdoc2aa KRCAHLAAMDVNGYSDPYVKTYLKPDVQKKSKHKTAVIKKTLNPEFNEEFFYEITFSELATKTLEVTVWDYDLGKSNDFIGGVSLGCHSQGETLQHWIDCLKNKGEKVERWHTLTNELPGSTLQE---
Gaculeatusdoc2ab RRCAHLAAMDVNGFSDPYVKIYLKPDLQKKSKHKTAVMKKTLNPEFNEEFFYEISLPELAHRMLEVTVWDYDLGRSNDFIGGVCLSCRSQGDGLRHWMDCLKNQGSRVERWHILTNELPRTLGHD---
Olatipesdoc2aa KRCAHLAAMDVNGFSDPYVKTYLKPDVQKKSKHKTAVIKKTLNPEFNEEFFYEISFSELATKTLEVTVWDYDLGRSNDFIGGVSLGCHSQGDALQHWIDCLKNKGKKVERWHTLTNDLPGSTLQE---
Olatipesdoc2ab KRCAHLAAMDVNGFSDPYVKIYLKPDIKKKSKHKTAVRKKTLNPEYDEEFFYEISLTELAKKTLEVTVWDYDLGRSNDFIX---
Dreriodoc2a LRCAHLAAMDVNGFSDPYVKIYLKPDVKKKSKHKTSVIKKTLNPEFNEEFFYEISLSELVHKTLEVTVWDYDLGRSNDFIGGVCLSCHVQGDALRHWMDCLRNKGQRVERWHILANELPQTTCHD---
Xtropicalisdoc2a MRCAHLAAMDVNGFSDPYVKTYLKPDVEKKSKHKTVVKKKTLNPEFNEEFFYNISQLELQKRSLEVTVWDYDLGKSNDFIGGVTFGMSSKGECLKHWMECLSTPNKRTEHWHTLTNELPGSTYAD---
Acarolinensisdoc2a LRCAHLAAMDVCGYSDPYVKAYLKPDEEKKSKHKTTVKKKTLNPEYNEDFFYEIEQSDLGQKSLEITVWDYDIGKSNDFIGGVTLGLGATGECRQHWLSCLQTPDCRLEHWHTLTNELPESSAFGP--
OanatinusDoc2a VRCAHLAAMDVNGYSDPYVKTYLRPDVEKKSKHKTAVKKKTLNPEFNEEFFYEMELPALATKTLEITVWDYDIGKSNDFIGGVSLGPGSQGEALKHWGECLRRPDTALEHWHTLTSELPPASYSA---
MdomesticaDoc2a VRCAHLAAMDVNGYSDPYVKTYLRPDVEKKSKHKTSVKKKTLNPEFNEEFFYEMELPALATKTLEVTVWDYDIGKSNDFIGGVSLGPNSRGEALKHWGDCLRRPDVTLERWHTLTSELPPAAASFST-
MmusculusDoc2a VRCAHLAAMDVNGYSDPYVKTYLRPDVDKKSKHKTCVKKKTLNPEFNEEFFYEIELSTLATKTLEVTVWDYDIGKSNDFIGGVSLGPGARGEAQKHWNDCLHQPDTALERWHTLTSELPPAAGAYPLA
HsapiensDOC2Avar1 LRCAHLAAMDVNGYSDPYVKTYLRPDVDKKSKHKTCVKKKTLNPEFNEEFFYEIELSTLATKTLEVTVWDYDIGKSNDFIGGVSLGPGARGEARKHWSDCLQQPDAALERWHTLTSELPPAAGALSSA
HsapiensDOC2Avar2 LRCAHLAAMDVNGYSDPYVKTYLRPDVDKKSKHKTCVKKKTLNPEFNEEFFYEIELSTLATKTLEVTVWDYDIGKSNDFIGGVSLGPGARGEARKHWSDCLQQPDAALERWHTLTSELPPAAGALSSA
HsapiensDOC2Avar3 LRCAHLAAMDVNGYSDPYVKTYLRPDVDKKSKHKTCVKKKTLNPEFNEEFFYEIELSTLATKTLEVTVWDYDIGKSNDFIGGVSLGPGARGEARKHWSDCLQQPDAALERWHTLTSELPPAAGALSSA
HsapiensDOC2Avar4 LRCAHLAAMDVNGYSDPYVKTYLRPDVDKKSKHKTCVKKKTLNPEFNEEFFYEIELSTLATKTLEVTVWDYDIGKSNDFIGGVSLGPGARGEARKHWSDCLQQPDAALERWHTLTSELPPAAGALSSA
Trubripesdoc2b IRCAHLAAMDANGFSDPYVKTYLKPDENKKSKHKTAVKKKTLNPEFNEEFCYDIKYADLTKKTLEVTVWDYDIGKSNDFIGGVSLGINANGERLKHWFDCLKNKDKKIERWHTLTNELPGS---LND-
Tnigroviridisdoc2b IRCAHLAAMDANGFSDPYVKTYLKPDENKKSKHKTAVKKKTLNPEFNEEFCYDIKYADLTKKTLEVTVWDYDIGKSNDFIGGVSLGINANGERLKHWFDCLKNKDKKIERWHTLTNELPGS---LTD-
Gaculeatusdoc2b IRCAHLAAMDANGFSDPYVKTYLKPDENKKSKHKTAVKKKTLNPEFNEEFCYDIKYADLTKKTLEVTVWDYDIGKSNDFIGGVSLGINANGERLKHWFDCLKNKDKKIERWHTLTNELPGS---LND-
Olatipesdoc2b IRCAHLAAMDANGFSDPYVKTYLKPDENKKSKHKTAVKKKTLNPEFNEEFCYDIKYADLTKKTLEVTVWDYDIGKSNDFIGGVSLGINANGERLKHWFDCLKNKDKKIERWHTLTNELPGS---LND-
Dreriodoc2b IRCAHLAAMDANGFSDPYVKTYLKPDENKKSKHKTAVKKKTLNPEFNEEFFYEIKYADLSKKTLEVTVWDYDIGKSNDFIGGVSLGINASGERLKHWFDCLKNKDKKIERWHTLTNELPGSG--YND-
Xtropicalisdoc2b IRCAHLAAMDANGYSDPYVKTYLKPDEDKKSKHKTAVKKKTLNPEFNEEFCYEIKHSDLAKKTLEVTVWDYDIGKSNDFIGGVVLGINAKGERLKHWFDCLKNKDKKIERWHTLTNELPGS--VLSD-
Acarolinensisdoc2b --------------------XYLKPDEDKKSKHKTAVKKKTLNPEFNEEFCYDIKHADLTKKTLEITVWDYDIGKSNDFIGGVVLGINAKGERLKHWFDCLKNKDKKIERWHTLTNELPGAILSD---
GgallusDOC2B IRCAHLAAMDANGYSDPYVKTYLKPDEDKKSKHKTAVKKKTLNPEFNEEFCYEIKHGDLAKKTLEVTVWDYDIGKSNDFIGGVVLGINAKGERLKHWFDCLKNKDKKIERWHTLTNELPGA--VLSD-
TguttataDOC2Bvar1 LRCAHLAAMDANGYSDPYVKIYLKPDEDKKSKHKTAVKKKTLNPEFNEEFFYEIKHGDLAKKTLEVTVWDYDIGKSNDFIGGVVLGINAKGERLKHWFDCLKNKDKKIERWHTLTNELPGA--VLSD-
TguttataDOC2Bvar2 LRCAHLAAMDANGYSDPYVKIYLKPDEDKKSKHKTAVKKKTLNPEFNEEFFYEIKHGDLAKKTLEVTVWDYDIGKSNDFIGGVVLGINAKGERLKHWFDCLKNKDKKIERWHTLTNELPGA--VLSD-
OanatinusDoc2b VRCAHLAAMDANGYSDPYVKIYLKPDVDKKSKHKTAVKKKTLNPEFNEEFCYEIKHGDLTKKTLEVTVWDYDIGKSNDFIGGVVLGINAKGERLKHWFDCLKNKDKKMERWHTLTNELPGATSVLSD-
MdomesticaDoc2b VRCAHLAAMDANGYSDPYVKIYLKPDVDKKSKHKTAVKKKTLNPEFNEEFCYEIKHGDLAKKTLEVTVWDYDIGKSNDFIGGVVLGINAKGERLKHWFDCLKNKDKKIERWHTLTNELPGA--VLSD-
MmusculusDoc2b VRCAHLAAMDANGYSDPYVKTYLKPDVDKKSKHKTAVKKKTLNPEFNEEFCYEIKHGDLAKKTLEVTVWDYDIGKSNDFIGGVVLGINAKGERLKHWFDCLKNKDRIERWHTLTNELPGA--VLSD-
HsapiensDOC2B VRCAHLAAMDANGYSDPYVKTYLRPDVDKKSKHKTAVKKKTLNPEFNEEFCYEIKHGDLAKKSLEVTVWDYDIGKSNDFIGGVVLGIHAKGERLKHWFDCLKNKDRIERWHTLTSELPGA--VLSD-
Trubripesdoc2d IRCAHLAAMDSNGYSDPFVKIILQPDMGKKSKYKTSVKKKTLNPEFNEEFQYEVSLDQLSKKTLEISVWDYDLGMSNDFIGGVELGINASGQRLRHWFECLKNKGKKVEYWHTLTQQGAPSSDKTD--
Tnigroviridisdoc2d IRCAHLAAMDSNGYSDPFVKIILQPDMGKKSKYKTSVKKKTLTX------------------------------------XGVELGINASGQRLRHWFECLKNKGKKVEYWHTLTQQGAPSSEKTD--
Gaculeatusdoc2d IRCAHLAAMDSNGYSDPFVKIMLQPDMGKKSKYKTTVKKKTLNPEFNEEFSYEVTLDQLAKKTLEISVWDYDLGMSNDFIGGVELGINASGQRLRHWFECLKNKGKKVEYWHTLTQQGAPSGDKPD--
Olatipesdoc2d IRCAHLAAMDSNGYSDPFVKIILQPDMGKKSKYKTSVKKKTLNPEFNEEFSYDVSLDQLAKKTLEISVWDYDLGMSNDFIGGVELGINASGQRLKHWFECLKNKGKKVEYWHTLTQQGAPSTDKPD--
Dreriodoc2dvar1 IRCAHLAAMDSNGYSDPFVKIILQPDMGKKSKYKTTVKKKTLNPEFNEEFSYDVPHDQLAKKTLEISVWDYDLGMSNDFIGGVELGINAKGDRLKHWFECLKYKGKKVEYWHTLTQQGAPSSD-----
Dreriodoc2dvar2 IRCAHLAAMDSNGYSDPFVKIILQPDMGKKSKYKTTVKKKTLNPEFNEEFSYDVPHDQLAKKTLEISVWDYDLGMSNDFIGGVELGINAKGDRLKHWFECLKYKGKKVEYWHTLTQQGAPSSD-----
MmusculusDoc2gvar1 LRCVHLAPMDANGYSDPFVRLFLHPSSGKKSKYKTSVRRKTLNPEFNEEFFYAGHREELAQKALLVSVWDYDLGTADDFIGGVQLSGRASGERLRHWRECLGHCDHRLELWHLLDSVPPQLGD-----
MmusculusDoc2gvar2 LRCVHLAPMDANGYSDPFVRLFLHPSSGKKSKYKTSVRRKTLNPEFNEEFFYAGHREELAQKALLVSVWDYDLGTADDFIGGVQLSGRASGERLRHWRECLGHCDHRLELWHLLDSVPPQLGD-----
Hsapiensdoc2g1 --
Hsapiensdoc2g2 LRCAHLAPMDANGYSDPFVRLFLHPNAGKKSKFKTSVHRKTLNPEFNEEFFYSGPREELAQKTLLVSVWDYDLGTADDFIGGVQLGSHASGERLRHWLECLGHSDHRLKLWHPLDSKPVQLSD-----
