# Supplementary material for: A manual collection of Syt, Esyt, Rph3a, Rph3al, Doc2, and Dblc2 genes from 46 metazoan genomes - an open access resource for neuroscience and evolutionary biology
Source: BMC Genomics. 2010 Jan 15;11:37. doi: 10.1186/1471-2164-11-37 (PMC2823689; doi:10.1186/1471-2164-11-37)
Supplement: Additional file 15 — Alignment of the vertebrate Syt4 sequences. Amino acid position is marked every hundred amino acids approximately, at the top of each page of the alignment. The G. gallus splice variant is included and highlighted with a black dot where it differs. Intron position and phase is indicated with a coloured bar between amino acids. Black bars indicate phase 0 introns. Red bars indicate phase +1 introns. X residues indicate where a portion of sequence is missing. [file 1471-2164-11-37-S15.PDF]

```
                                                                                                                   100
Trubripessyt4          MAPMVEEGSQLVAVPTGVAVVSVFGLIFTASAFSWICCQRKSGKAQ-KTPPYKFVHMLKGVDIYPESLGGKKKFAAAAAMTTGDAGKTDINGNCHTTSLMSPASDGKLASSPSDARPALH
Tnigroviridissyt4      MAPMVEEGSQLVAVPVGVAVVSVFGLVFTASAFAWICCQRKSTKSQ-KTPPYKFVHMLKGVDIYPESLSGKKTFAAAA----GDAGKTDINGNCHTASLMS--------TAGGDARPALQ
Gaculeatussyt4         MAPMVEEGARLVAVPVGVAVVSVFGLVFTVSAFAWICCQRKNAKSQ-KTPPYKFVHMLKGVDIYPESLSNKKKFAAHATMTTSDTGKTDVNGNCHTVAPMSPTSNGKLASSPNGSRQALH
Olatipessyt4           MAPMMESGAEPVAVPVGVAVVSVFGLVFTVSAFTWICCQRKNTKSQ-KTPPYKFVHMLKGVDIYPESLSGKKKFAAAATTTVNDAGKTDVNGNCHTT-PMSPSSTSKLATSPNGSRPALH
Dreriosyt4             MAPVTTEEAHFAEVPVSVAVVSVFGLVFSVSIFAWICCQRKANKSSNKTPPYKFVHMLKGVDIYPESLSGKKKFGGEK----------------------TPEAHGKQTLSPTGGRPDLH
Xtropicalissyt4        MAPITSGRDAFDEIPSVVGLVSGFGLVFTVSLFSWICCQRKSAKSN-TTPPYKFVHVLKGVDIYPESLNSKKKFGADE--------------------------KTELAPKSLISKTSLH
Acarolinensissyt4      -----------XEIPTVVGIFSAFGLVFTVSLFAWICCQRKSSKSN-KTPPYKFVHVLKGVDIYPENLNSKKKFGAED--------------------------KGEAKNKSIIPKSSLH
GgallusSYT4var1        MAPIAASHQQFDEIPTVVGIFSAFGLVFSVSLFAWICCQRKSSKSN-KTPPYKFVHVLKGVDIYPENLNSKKKFGVDD--------------------------KSEAKNKSAMPKNSLH
GgallusSYT4var2        MAPIAASHQQFDEIPTVVGIFSAFGLVFSVSLFAWICCQRKSSKSN-KTPPYKFVHVLKGVDIYPENLNSKKKFGVDD--------------------------KSEAKNKSAMPKNSLH
TguttataSYT4           MAPIADSRQQFDEIPTVVGIFSAFGLVFSVSLFAWICCQRKSSKSN-KTPPYKFVHVLKGVDIYPENLNSKKKFGADD--------------------------KSEAKNKSATPKNSLH
OanatinusSyt4          MAPITASREELDEIPTVVGIFSAFGLVFTVSLFAWICCQRKSSKSN-KTPPYKFVHVLKGVDIYPENLNSKKKFGADD--------------------------KNEANSKSALPKNSLH
MdomesticusSyt4        MAPITSSREEFDEIPTVVGIFSAFGLVFTVSLFAWICCQRKSSKTN-KTPPYKFVHVLKGVDIYPENLNSKKKFGADD--------------------------KNEAKNKPVVPKTSLH
MmusculusSyt4          MAPITTSRVEFDEIPTVVGIFSAFGLVFTVSLFAWICCQRRSAKSN-KTPPYKFVHVLKGVDIYPENLSSKKKFGGDD--------------------------KSEVKGKAALPNLSLH
HsapiensSYT4           MAPITTSREEFDEIPTVVGIFSAFGLVFTVSLFAWICCQRKSSKSN-KTPPYKFVHVLKGVDIYPENLNSKKKFGADD--------------------------KNEVKNKPAVPKNSLH

                                                                                               200
Trubripessyt4          LDLEKRDLNGNFTTKPFHHHQQQ--TVRSSPDLELPSPHAGFTQPGTVDRRDLPSPSSSFSSQAPTPAVDKPLGEEKEGGVGTLHFSLEYQPERKAFIVHIKEAHGLAPTDEQSLTSDPY
Tnigroviridissyt4      LDLEKRDLNGNFTTKPFHHHHHQPQAVRSSPDLELPSPQAGFTQPRAADRPDLPSPSSSFSSQAPTPAVDKPPTEEKEGGVGTLHFSLEYQPERKAFIVHIKEANGLAPTDEQSLTSDPY
Gaculeatussyt4         LDLEKRDLNGNFTTKPFHHHSQK---VRSSPDLELPSPHAGFTQPGAMDRRDLPSPCSTLSSQAPTPAVEKPQVEDREGGLGTLHFSLEYQPEKKAFIVHIKEAHGLTPTDEQSLTSDPY
Olatipessyt4           LDLEKRDLNGNFTTKPFHHHHQK---VRCSPDLELPSPHAGFTQPGAMDRHDLPSPFSNLSSQAPTPGVERAHVEEKENGLGTLHFSLEYQQEKKAFIVHIKEANGLSPTDEQSLTSDPY
Dreriosyt4             LDLEKRDLNGNFTTKPPTLQLK----VRSSPDIDIPALQGGFAN------QEAGTPESVVSSHTPTPAVEKS--QDKEGGLGTLFFSVEYNFEKKAFMVHIKEAHGLSPTDEQSLTSDPY
Xtropicalissyt4        LDLEKRDLNGNFPKAN-K--------VRSSPDLEDFSQPQLPEK----D-KDSVSPESVKSNASVVS-------LDKQDKLGTLFFALEYNFEKKAFVVNIKEARSLPAMDEQSMTSDPY
Acarolinensissyt4      LDLEKRDLNGNFPKTVPK--------ITSSSEVENSIPKVFPER----E-KDSVSPDSLKSISSLSS-------EEKQDKLGTLFFSLEYNFEKKAFMVNIKEARGLPAMDEQSMTSDPY
GgallusSYT4var1        LDLEKRDLNGNFPKTTSK--------MQSSPDPENSSSKHFSEN----Q-KDSVSPDSLKSTTSLSS-------EEKQDKLGTLFLSLEYNFEKKAFVVSIKEARGLPAMDEQSMTSDPY
GgallusSYT4var2        LDLEKRDLNGNFPKTTSK--------MQSSPDPENSSSKHFSEN----Q-KDSVSPDSLKSTTSLSS-------EEKQDKLGTLFLSLEYNFEKKAFVVSIKEARGLPAMDEQSMTSDPY
TguttataSYT4           LDLEKRDLNGNFPKTTTK--------MQGSTDLENSSPKHFAER----K-KDSVSPDSLKSITSLSS-------EGKQDKLGTLFFSLEYNFEKKAFVVNIKEARGLPAMDEQSMTSDPY
OanatinusSyt4          LDLEKRDLNGNFPKTNAK--------VMSPSDLENLTPKHFSES----E-KDSISLDSLKSNTSLSL-------EEKQEKLGTLFLSLEYNFEKKAFVVNIKEARGLPAMDEQSMTSDPY
MdomesticusSyt4        LDLEKKDLNGNFPKANPK--------AGSPSDLENLTPKPFSER----EQKESASPDSLKSNSSLCS-------VEKQDKLGTLFFSLEYNFEKKAFIVNIKEARGLPAMDEQSMTSDPY
MmusculusSyt4          LDLEKRDLNGNFPKANPK--------AGSSSDLENVTPKLFTET----E-KEANSPESLKSSTSLTS-------EEKQEKLGTLFLSLEYNFEKKAFVVNIKEAQGLPAMDEQSMTSDPY
HsapiensSYT4           LDLEKRDLNGNFPKTNLK--------PGSPSDLENATPKLFLEG----E-KESVSPESLKSSTSLTS-------EEKQEKLGTLFFSLEYNFERKAFVVNIKEARGLPAMDEQSMTSDPY

                                                                       300
Trubripessyt4          IKLTLLPEKKHRVKTRVLRKTLDPAFDETFSFYGIPLARVSELALHFMVLSFDRFSRDEVIGETLVPLSGIDLSEGRVLMSREIIKRNSKKSSGRGELLLSLCYQSTTNTLTVVVLKARN
Tnigroviridissyt4      IKLTLLPEKKHRVKTRVLRKTLDPAFDETFSFYGLPLARVSELALHFMVLSFDRFSRDEVIGETLVPLSGIDLSEGRVLMSREIIKK--KKSSGRGELLLSLCYQSTTNALTVVVLKARN
Gaculeatussyt4         IKLTLLPEKKHRVKTRVLRKTLDPAFEETFSFYGIPLARVSELALHFMVLSFDRFSRDEVIGETLVPLSGIDLSEGRVLMSREIIKKNVKKSSCRGELLLSLCYQSTTNTLTVVVLKARH
Olatipessyt4           IKLTLLPEKKHRVKTRVLRKTLDPSFDETFSFYGISLSRVTELSLHFMVLSFDRFSRDEVIGETLVPLSGIDLSEGRVLMSREIIKRNIKKSSGRGELLLSLCYQSTTNTLTVVVLKARH
Dreriosyt4             IKLTLLPEKKHKVKTRVLRKTLDPAFDETFSFYGIPFARVSQLALHFMVLSFDRFSRDEVIGETLVPLADIDLSEGRVLMSRDIIKKNIRRSAGRGELLLSLCYQSTTSTLTVVVLKARH
Xtropicalissyt4        IKMTILPEKKHKVKTRVLRKTLDPAFDETFTFYGIPYSQVQDLVLHFIILSFDRFSRDDVIGEVSFPLSGIELSDGRVLMNREINKRNVRKSAGRGELLISLCYQSTTNTLTVVVLKARH
Acarolinensissyt4      IKMTVLPEKKHKVKTRVLRKTLDPAFDETFTFYGIPYTQVQDLSLHFMILSFDRFSRDDVIGEVLFPLSGIELSEGRLLMNKEIIKKNVRKSSGRGELLISLCYQSTTNTLTVVVLKARH
GgallusSYT4var1        IKMTILPEKKHKVKTRVLRKTLDPAFDETFTFYGIPYSQIQDLTLHFMILSFDRFSRDDVIGEVLIPLAGIELSEGRMLMDREIIKRNVRKSSGRGELLISLCYQSTTNTLTVVVLKARH
GgallusSYT4var2    ●   IKMTILPEKKHKVKTRVLRKTLDPAFDETFTFYGIPYSQIQDLTLHFMILSFDRFSRDDVIGEVLIPLAGIELSEGRMLMDREIIKRN--KSSGRGELLISLCYQSTTNTLTVVVLKARH
TguttataSYT4           IKMTILPEKKHKVKTRVLRKTLDPAFDETFTFYGIPYSQIQDLTLHFMVLSFDRFSRDDVIGEVLIPLAGTELSEGRLLMDREIIKRNVRKSSGRGELLVSLCYQSTTNTLTVVVLKARH
OanatinusSyt4          IKMTILPEKKHKVKTRVLRKTLDPAFDETFTFYGIPYSQIQDLVLHFMILSFDRFSRDDIIGEVLIPLSGIELSNGKMLMNREIIKRNVRKSSGRGELLISLCYQSTTNTLTVVVLKARH
MdomesticusSyt4        IKMTILPEKKHKVKTRVLRKTLDPAFDETFTFYGIPYSQIQDLVLHFIILSFDRFSRDDIIGEVLIPLSGIELADGRMLMNREIIKRNVRKSSGRGELLISLCYQSTTNTLTVVVLKARH
MmusculusSyt4          IKMTILPEKKHRVKTRVLRKTLDPVFDETFTFYGIPYPHIQELSLHFTVLSFDRFSRDDVIGEVLIPLSGIELSDGKMLMTREIIKRNAKKSSGRGELLVSLCYQSTTNTLTVVVLKARH
HsapiensSYT4           IKMTILPEKKHKVKTRVLRKTLDPAFDETFTFYGIPYTQIQELALHFTILSFDRFSRDDIIGEVLIPLSGIELSEGKMLMNREIIKRNVRKSSGRGELLISLCYQSTTNTLTVVVLKARH

                                             400
Trubripessyt4          LPKIENNGPTDPYVKVNMYQGKKRVCKKKTHVKKCSPNPVFNELFVFDLPSEEGLRDTSVELLLMDSDSGNSRCPNTVLGRLVLGMSATGTPGEHWREICEHPRRQIAKWHGLSEE
Tnigroviridissyt4      LPKTDNNGPTDPYVKVNMYQGKKRVCKKKTHMKKCSPNPVFNELFVFDLPSDEGLRDTSVELLLMDSASGTARCPSPVLGRLVLGTSSTGTPGEHWREICDHPRRQIAKWHGLSED
Gaculeatussyt4         LPKTENNGPTDPYVKVNMYQGKKRVCKKKTHVKKCSPNPVFNELFVFDLPSDEGLRDTSVELLLMDSDTGNSRCPNTVLGRLVLGTSVAGTPGEHWREICDHPRRQIAKWHGLSED
Olatipessyt4           IPRTENNGPTDAYVKVNMYHGKKRICKKKTHVKKCSPNPVFNELFYFDLPSEEGLRDTSIELLLMDSD--NSRSSNGAIGRLVLGTAAAGTAGEHWREICDHPRRQIAKWHDMSED
Dreriosyt4             LPKADSSGPSDPYVKVNLFQGKKRVCKKKTHVKKCAPNPVFNELFVFDLPSEDGLRDTSVELLLLDSDR-TSRTP--VIGRLVLGTSSPGTAGEHWREICDHPRRQIAKWHALSED
Xtropicalissyt4        LPKAEVSGLSDPYVKVNLYHAKKRISKKKTHVKKCTPNAVFNELFVFDIPC-EGLEDISVEFLVMDSDRGSR---NEVIGRLILGSSADGTGGEHWKEICEHPRRQIAKWHMLCDG
Acarolinensissyt4      LPKADVSGLSDPYVKVNLYHAEKRISKKKTHVKKCTPNAVFNELFVFDIPC-EGLQEISIEFLVLDSDRGSR---NEIIGRLTLGASAEGTAGEHWKEICEYPRRQIAKWHMLCDG
GgallusSYT4var1        LPKSDVSGLSDPYVKVNLYHVKKRISKKKTHVKKCTPNAVFNELFVFDIPC-EGLDDISIEFLVLDSDRGSR---NEVIGRLTLGSSAEGTGGEHWKEICEYPRRQIAKWHMLCDG
GgallusSYT4var2        LPKSDVSGLSDPYVKVNLYHVKKRISKKKTHVKKCTPNAVFNELFVFDIPC-EGLDDISIEFLVLDSDRGSR---NEVIGRLTLGSSAEGTGGEHWKEICEYPRRQIAKWHMLCDG
TguttataSYT4           LPKSDVSGLSDPYVKVNLYHAKKRISKKKTHVKKCTPNAVFNELFVFDIPC-EGLDDISIEFLVLDSDRGSR---NEVIGRLTLGSSAEGTGGEHWKEICEYPRRQIAKWHMLCDG
OanatinusSyt4          LPKADVSGLSDPYVKVNLYHAKKRISKKKTHVKKCTPNAVFNELFVFDIPC-EGLEDISVEFLVLDSDRGSR---NEVIGRLILGASAEGTGGEHWKEICEFPRRQIAKWHMLCDG
MdomesticusSyt4        LPKADVSGLSDPYVKVNLYHAKKRISKKKTHVKKCTPNAVFNELFVFDIPC-EGLEEISVEFLVLDSDRGSR---NEVIGRLVLGASAEGTGGEHWKEICEYPRRQIAKWHMLCDG
MmusculusSyt4          LPKSDVSGLSDPYVKVNLYHAKKRISKKKTHVKKCTPNAVFNELFVFDIPC-ESLEEISVEFLVLDSERGSR---NEVIGRLVLGATAEGSGGGHWKEICDFPRRQIAKWHMLCDG
HsapiensSYT4           LPKSDVSGLSDPYVKVNLYHAKKRISKKKTHVKKCTPNAVFNELFVFDIPC-EGLEDISVEFLVLDSERGSR---NEVIGQLVLGAAAEGTGGEHWKEICDYPRRQIAKWHVLCDG
```
