# Supplementary material for: A manual collection of Syt, Esyt, Rph3a, Rph3al, Doc2, and Dblc2 genes from 46 metazoan genomes - an open access resource for neuroscience and evolutionary biology
Source: BMC Genomics. 2010 Jan 15;11:37. doi: 10.1186/1471-2164-11-37 (PMC2823689; doi:10.1186/1471-2164-11-37)
Supplement: Additional file 16 — Alignment of the vertebrate Syt11 sequences. Amino acid position is marked every hundred amino acids approximately, at the top of each page of the alignment. Splice variants are included and highlighted with black dots where they differ. Intron position and phase is indicated with a coloured bar between amino acids. Black bars indicate phase 0 introns. Red bars indicate phase +1 introns. X residues indicate where a portion of sequence is missing. [file 1471-2164-11-37-S16.PDF]

```
                                                                                                   100
Trubripessyt11          MADMIELGPTYAMSPVLAGFLGAGVLVLVVAVLVLLWSFCQRRYMRLSGR-HKLHGDRYCDAEDPPYKFIHMLKGISIYPESLSSSKRIVRGARRAEHSDRG-------ATTGRGMVLVD
Tnigroviridissyt11      MAEMIELGPTYAMSPVLAGFLGAGALVLLVAVLVLLWSFCQRRYLHLSGR-RKLHGDRYCDAEEPPYKFIHMLKGISIYPESLSSSKRIVRGARRAQHGERG-------AA-GRGMVLVD
Gaculeatussyt11         MADVIELGPAYAMSPVLAGFLGAGVLVLVVVVLVLLWSFCQRRYLRVSGR-YKLHGDRYCDSEDPPYKFIHMLKGISIYPESLSSSKRIVRGIRRADRSKRDGERGFPTAGAGRGLVLVD
Olatipessyt11           MADMVELGPAYAMSPVLAGFLGAGVLVLVMVVLVLLWSFCQRRYLRISGR-YKLHGDRYCDAEDPPYKFIHMLKGISIYPESLSGSKRIVRGVRRADRSDADGERGGAAGPAGRGMVLVD
Dreriosyt11a            MAEMTNLKPTYDVSPVLAGFLGAGVLVVSVTVAVFLWTCCQRRYRQMTGA-YKLTSGPYDPPVDPPYKFIHMLKGISIYPETLSNSKKIVRVGRRSASNSLLREWG--RGSRNGDVLLVD
Dreriosyt11b            MAEMTEIGAAYDMSPVLAGFIGAGVLVVAVFALIYLWTCCQKHYNRTNYKLHGIQSEHSDPLTDPPYKFIHMLKGMSIYPDALTNSKRIIRVAREARAQTSDRV----QGSKGRPVVLVN
Acarolinensissyt11      -----------XVSPIVAGLIGATVLVVSVSVTVFVWTCCHQQAEKKH--------------KTPPYKFIHMLKGISIYPETLSNKKKIIRIRRDKTGAARK---------DGSGSLSGD
GgallusSYT11var1        MAEITSVHPGFDVSPVVAGLIGATVLVVSVSVTVFVWTCCHQQAEKKH--------------KTPPYKFIHMLKGISIYPETLSNKKKINRIRRDKNGAPKE---------TGRGNLLVD
GgallusSYT11var2        MAEITSVHPGFDVSPVVAGLIGATVLVVSVSVTVFVWTCCHQQAEKKH--------------KTPPYKFIHMLKGISIYPETLSNKKKINRIRRDKNGAPKE---------TGRGNLLVD
TguttataSYT11           -----------XVSPVVAGLIGATVLVVSVSVTVFVWTCCHQQAEKKH--------------KTPPYKFIHMLKGISIYPETLSNKKKINRIRRDKEG-------------GRGNLLVD
OanatinusSyt11          MAEITNLRPPYDVSPVVAGLVGATVLVVSVSVSVFVWTCCRRRAQKKH--------------QAPPYKFIHMLKGVSIYPEPLX------------------------------------
MdomesticaSyt11         -----------XVSPVVLGLIGATVLVVSVSVTVFVWTCCHQQAEKKH--------------KTPPYKFIHMLKGISIYPETLSNKKKIIRVRRDKGGPGRD---------GGRGNLLVD
MmusculusSyt11var1      MAEITNIRPSFDVSPVAAGLIGASVLVVCVSVTVFVWTCCHQQAEKKH--------------KTPPYKFIHMLKGISIYPETLSNKKKIIKVRRDKDGPRRE---------SGRGNLLIN
MmusculusSyt11var2      MAEITNIRPSFDVSPVAAGLIGASVLVVCVSVTVFVWTCCHQQAEKKH--------------KTPPYKFIHMLKGISIYPETLSNKKKIIKVRRDKDGPRRE---------SGRGNLLIN
HsapiensSYT11var1       MAEITNIRPSFDVSPVVAGLIGASVLVVCVSVTVFVWSCCHQQAEKKQ--------------KNPPYKFIHMLKGISIYPETLSNKKKIIKVRRDKDGPGRE---------GGRRNLLVD
HsapiensSYT11var2       MAEITNIRPSFDVSPVVAGLIGASVLVVCVSVTVFVWSCCHQQAEKKQ--------------KNPPYKFIHMLKGISIYPETLSNKKKIIKVRRDKDGPGRE---------GGRRNLLVD
```

```
                                                                                    200
Trubripessyt11          AENNILDVPGQLQMSHLIPPANSGPQ---GRLERALPVRTDYCC-LDSSSGGSSQTNSKTPSPFTPASSEPEPEPERSLGTLSLTVDYNFPKKALVVTIVGARGLPAMDEQVSSSDPYVK
Tnigroviridissyt11      AENNILDVPGQLQMSHLLPPAGAGPA---GRLERGLPVRSDYCC-LDSSSAGSSQTGSKTPSPFTPASLQPEPEP--SLGTLSLTVDYNFPKKALVVTIVGARGLPAVDEQVSSSDPYVK
Gaculeatussyt11         AENNILDVPGQLQMSHLVPPAGPGPAHGAGRTERALPVRADYCC-LDSSSAGSSQTSSKTASPFTPAPSEPEPDP--SLGTVSLTVDYNFPKKALVVTIVGARGLPAMDEQAGSSDPYVK
Olatipessyt11           AENNILDVPGQLQMSHLVPPAGAAAPQGQGRMERALPVRADYCC-LDSSSASSSQTSSKTASPFTPAGAEPEP----NLGSISLAVDYNFPKKALVVTIVGARGLPAMDEQAGSSDPYVK
Dreriosyt11a            ADPECGTT--HLQMNHLVPPVGPP------RLERALPIRADYCC-MESSSGSSSEAHSKTASPYSLNSP--------SLGTLSLAIDYNFPKKALVVTIIGAQGLPAVDEQAGSSDPYVK
Dreriosyt11b            MDSAAESGLLGEKQMRLEESGHEV-----SRLERELPVRADYCC-LDSSSASSSQTSSTTVSS-TATPFTPADEP--SRGDLSIAIDYNFPKKALVVTILEARGLPAVEGQTGSADPYVK
Acarolinensissyt11      EAEAGL-------AGAEKLSGGFSA----PPCVKQLPIKVDYGEGL-SPAQSLTPAGSKTPSPSSPEEEV-------MLGVLTFSVDYNFPKKALVVTIQEAHGLPVMDEQNQGSDPYIK
GgallusSYT11var1        AAESGL-------VGSDKAPDGPNT----APHIDQLPIKVDYGDEL-SPDQSLTPGGSKTSSPSSPGEDV-------LLGELTFSVDYNFPKKALVVTIQEAHGLPVMDEHTQSSDPYIK
GgallusSYT11var2        AAESGL-------VGSDKAPDGPNT----APHIDQLPIKVDYGDEL-SPDQSLTPGGSKTSSPSSPGEDV-------LLGELTFSVDYNFPKKALVVTIQEAHGLPVMDEHTQSSDPYIK
TguttataSYT11           AAESGL-------MGPTKAPDGP-------PQVDQLPIKVDYGDEL-SPDQSLTPGGSKTSSPSSPGDDV-------LLGDLTFSVDYNFPKKALVVTIQEAHGLPVMDEHTQSSDPYIK
OanatinusSyt11          --------------------------------------------------------------------------------------------XFLVVSIQEALVLPATDPQTQTADPYVK
MdomesticaSyt11         AAESGL-------LNREKSPGGLNT----SPCIDQLPIKVDYGDEL-SPVQSLTPGGSKTASPSSPEEDV-------MLGSLTFSVDYNFPKKALVVTIQEAHGLPVMDDQTQGSDPYIK
MmusculusSyt11var1      A-ESGL-------LSHDKDPRGPSP----ASCMDQLPIKRDYGEELRSPMTSLTPGESKATSPSSPEEDV-------MLGSLTFSVDYNFPKKALVVTIQEAHGLPVMDDQTQGSDPYIK
MmusculusSyt11var2      A-ESGL-------LSHDKDPRGPSP----ASCMDQLPIKRDYGEELRSPMTSLTPGESKATSPSSPEEDV-------MLGSLTFSVDYNFPKKALVVTIQEAHGLPVMDDQTQGSDPYIK
HsapiensSYT11var1       AAEAGL-------LSRDKDPRGPSS----GSCIDQLPIKMDYGEELRSPITSLTPGESKTTSPSSPEEDV-------MLGSLTFSVDYNFPKKALVVTIQEAHGLPVMDDQTQGSDPYIK
HsapiensSYT11var2       AAEAGL-------LSRDKDPRGPSS----GSCIDQLPIKMDYGEELRSPITSLTPGESKTTSPSSPEEDV-------MLGSLTFSVDYNFPKKALVVTIQEAHGLPVMDDQTQGSDPYIK
```

```
                                                             300
Trubripessyt11          MTILPEKKHRVKTRVLRKTLNPLFDETFTFYGVAYSSLPDLTLHFLVLSFDRFSRDDVIGESVVPLKGVDPSTGRVHISQQITKRNMQ-CDSRGELLVSLSYQPVSHRLSVVVLKARHLP
Tnigroviridissyt11      MTILPEKKHRVKTRVLRKTLDPLFDETFTFYGVAYSALPELTLHFLVLSFDRFSRDDVIGEAVVPLKGVDPSTGRVHLSQQISKRNMQ-CDSRGELLVSLSYQPVSHRLSVVVLKARHLP
Gaculeatussyt11         MTILPEKKHRVKTRVLRKTLDPLFDETFTFYGVAYSSLPELTLHFLVLSFDRFARDDVVGEAVVPLKGVDPSTGRVHLSQQISKRNMQ-CETRGELLASLSYQPVSHRLSVVVLKARHLP
Olatipessyt11           MTILPEX-----------------------------------------------------------------------------------------------------------------
Dreriosyt11a            MTILPEKKHRVKTRVLRKTLEPVFDETFTFYGVPYSSLPELTLHFLVLSFDRFARDDVIGEAVVPLEGVDPSTGRAHITQQISKRNTQ-CESRGELLVSLSYHPVTHRLNVVVLKAKHLP
Dreriosyt11b            MTILPEKKHRVKTRVLRKTLEPAFDETFTFYGVPYSSLSDLTLHFLVLSFDRFSRDDVIGEAMVPLAGVDPSTGRVHITQQITKRNMQ-CVSHGELLVSLSYQPVSHRLSVVVLKAKHLP
Acarolinensissyt11      MTILPDKRHRVKTRVLRKTLDPVFDETFTFYGIPYSQLQDLVLHFLVLSFDRFSRDDVIGEVMVPLAGVDPSTGKVHLTREIIKRNIQKCVSRGELQVSLSYQPVAQRMTVVVLKAKHLP
GgallusSYT11var1        MTILPDKRHRVKTRVLRKTLDPVFDETFTFYGIPYSQLQDLVLHFLVLSFDRFSRDDVIGEVMVPLAGVDPSTGKVQLTREILKRNIQKCISRGELQVSLSYQPVAQRMTVVVLKARHLP
GgallusSYT11var2        MTILPDKRHRVKTRVLRKTLDPVFDETFTFYGIPYSQLQDLVLHFLVLSFDRFSRDDVIGEVMVPLAGVDPSTGKVQLTREILKRNIQKCISRGELQVSLSYQPVAQRMTVVVLKARHLP
TguttataSYT11           MTILPDKRHRVKTRVLRKTLEPVFDETFTFYGIPYSQLQDLVLHFLVLSFDRFSRDDVIGEVMVPLAGVDPSTGKVQLTREILKRNIQKCISRGELQVSLSYQPVAQRMTVVVLKARHLP
OanatinusSyt11          MTVLPDRRHRVKTRVLRRTRNPVFDETFTFYGLPASQLQDLALHFLVLSFDRFSRDDVIGEVVLPLAGLDPATAKVQLTRDISQRSFQX-------------------------------
MdomesticaSyt11         MTILPDKRHRVKTRVLRKTLDPVFDETFTFYGIPYSQLQDLVLHFLVLSFDRFSRDDVIGEVMVPLAGVDPSTGKVQLTRDIIKRNIQKCISRGELQVSLSYQPVAQRMTVVVLKARHLP
MmusculusSyt11var1      MTILPDKRHRVKTRVLRKTLDPVFDETFTFYGIPYSQLQDLVLHFLVLSFDRFSRDDVIGEVMVPLAGVDPSTGKVQLTRDIIKRNIQKCISRGELQVSLSYQPVAQRMTVVVLKARHLP
MmusculusSyt11var2      MTILPDKRHRVKTRVLRKTLDPVFDETFTFYGIPYSQLQDLVLHFLVLSFDRFSRDDVIGEVMVPLAGVDPSTGKVQLTRDIIKRNIQKCISRGELQVSLSYQPVAQRMTVVVLKARHLP
HsapiensSYT11var1       MTILPDKRHRVKTRVLRKTLDPVFDETFTFYGIPYSQLQDLVLHFLVLSFDRFSRDDVIGEVMVPLAGVDPSTGKVQLTRDIIKRNIQKCISRGELQVSLSYQPVAQRMTVVVLKARHLP
HsapiensSYT11var2       MTILPDKRHRVKTRVLRKTLDPVFDETFTFYGIPYSQLQDLVLHFLVLSFDRFSRDDVIGEVMVPLAGVDPSTGKVQLTRDIIKRNIQKCISRGELQVSLSYQPVAQRMTVVVLKARHLP
```

```
                                             400
Trubripessyt11          KMDMTGLSA---NPYVKVNIFYGRKRIAKKKTHVKKCTLNPVFNESFIYDIPPELLPEISVEFLVVDFDRTTKNEVLGRLLLGLHSPAPTGASHWREVCESPRRQISKWHNLSEF
Tnigroviridissyt11      KMDMTGLSA---NPYVKVNVFYGRKRIAKKKTHVKKCTLNPVFNESFIYDIPPELLAEISVEFLVVDFDRTTKNEVLGRLLLGLHSPAPTGASHWREVCESPRRQISKWHNLSEY
Gaculeatussyt11         KMDITGLSA---NPYVKVNVFYGRKRIAKKKTHVKKCTLNPVFNESFIYDIPPELLPEISVEFLVVDFDRTTKNEVLGRLLLGLHGPAPSGASHWREVCENPRRQISKWHSLSEY
Olatipessyt11           ------------XPYVKVNVFYGRKRIAKKKTHVKKCTLNPVFNESFIYDIPPELLPEISVEFLVVDFDRTTKNEVLGRLLLGLHSPSPSGASHWREVCENPRRQISKWHNLSEY
Dreriosyt11a            TMDITGLSG---NPYVKVNVFYGRKRIAKKKTHVKKCTLNPVFNESFIYDVPAELMPDISVEFLVIDFDRTTKNEVVGRLVLGGQSPIPSGVTHWREVCDNPRRQIAKWHNLIEY
Dreriosyt11b            KLDITGLSA---NPYVKLNVFYGHKRIAKKKTHVKKCTLNPVFNESFIYDVPAELLPDISIEFLVMDFDRTTKNQALGRLVLGADSPCPSGAAHWQEVCQNPRRQISKWHTLN--
Acarolinensissyt11      KMDITGLS----DPYVKVNVYYGRKRIAKKKTHVKKCTLNPVFNESFIYDIPTELLPDISIEFLVIDFDRTTKNEVVGRLILGAHSVTAGGAEHWREVCESPRKQVAKWHSLSEY
GgallusSYT11var1        KMDITGLSG---NPYVKVNVYYGRKRIAKKKTHVKKCTLNPVFNESFIYDIPVDLLPDISIEFLVIDFDRTTKNEVVGRLILGAHSITAAGVEHWREVCENPRKPVAKWHSLSEY
GgallusSYT11var2     ●  KMDITGLS----DPYVKVNVYYGRKRIAKKKTHVKKCTLNPVFNESFIYDIPVDLLPDISIEFLVIDFDRTTKNEVVGRLILGAHSITAAGVEHWREVCENPRKPVAKWHSLSEY
TguttataSYT11           KMDITGLSG---NPYVKVNVYYGRKRIAKKKTHVKKCTLNPVFNESFIYDIPVDLLPDISIEFLVIDFDRTTKNEVVGRLILGAHSASAAGTEHWRQVCDSPGKAVAKWHSLSEY
OanatinusSyt11          -------------------------------------------------------------------------------------------------------------------
MdomesticaSyt11         KMDITGLSG---NPYVKMNLYYGRKRIAKKKTHVKKCTLNPVFNESFIYDIPTDLLPDISIEFLVIDFDRTTKNEVVGRLILGAHSVTAGGAEHWREVCESPRKPIAKWHSLSEY
MmusculusSyt11var1      KMDITGLSG---NPYVKVNVYYGRKRIAKKKTHVKKCTLNPVFNESFIYDIPTDLLPDISIEFLVIDFDRTTKNEVVGRLILGAHSVTTSGAEHWREVCESPRKPIAKWHSLSEY
MmusculusSyt11var2   ●  KMDITGLS----DPYVKVNVYYGRKRIAKKKTHVKKCTLNPVFNESFIYDIPTDLLPDISIEFLVIDFDRTTKNEVVGRLILGAHSVTTSGAEHWREVCESPRKPIAKWHSLSEY
HsapiensSYT11var1       KMDITGLSG---NPYVKVNVYYGRKRIAKKKTHVKKCTLNPIFNESFIYDIPTDLLPDISIEFLVIDFDRTTKNEVVGRLILGAHSVTASGAEHWREVCESPRKPVAKWHSLSEY
HsapiensSYT11var2    ●  KMDITGLSGRAPDPYVKVNVYYGRKRIAKKKTHVKKCTLNPIFNESFIYDIPTDLLPDISIEFLVIDFDRTTKNEVVGRLILGAHSVTASGAEHWREVCESPRKPVAKWHSLSEY
```
